# Asymmetric Organocatalyzed Transfer Hydroxymethylation of Isoindolinones Using Formaldehyde Surrogates

**DOI:** 10.1021/acs.orglett.4c00818

**Published:** 2024-03-19

**Authors:** David Svestka, Pavel Bobal, Mario Waser, Jan Otevrel

**Affiliations:** † Department of Chemical Drugs, Faculty of Pharmacy, Masaryk University, Palackeho 1, 612 00 Brno, Czechia; ‡ Institute of Organic Chemistry, Johannes Kepler University, Altenbergerstrasse 69, 4040 Linz, Austria

## Abstract

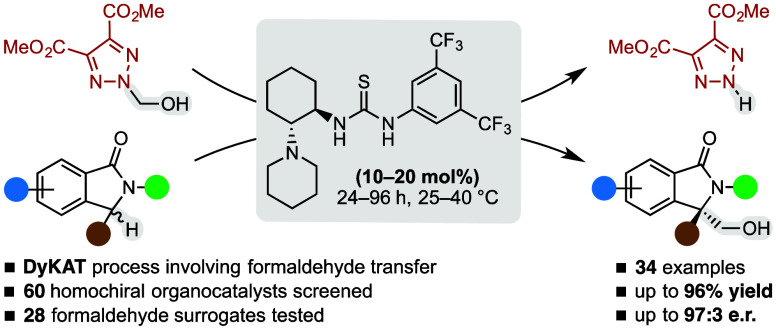

The piperidine-based Takemoto catalyst has been successfully employed in a novel asymmetric transfer hydroxymethylation of activated isoindolinones, allowing us to prepare the enantioenriched hydroxymethylated adducts in good to excellent yields (48–96%) and enantiopurities (81:19–97:3 e.r.). To increase the reaction rate without compromising the selectivity, carefully optimized formaldehyde surrogates were employed, providing a convenient source of anhydrous formaldehyde with a base-triggered release. The substrate scope, including 34 entries, showed the considerable generality of the asymmetric transformation, and most entries exhibited complete conversions in 24–48 h. A scale-up experiment and multiple enantioselective downstream transformations were also carried out, suggesting the prospective synthetic utility of the products.

The asymmetric cross-aldol reaction with formaldehyde, also known as enantioselective hydroxymethylation or methylolation, is one of the most efficient carbon chain extension methods, which is greatly rewarding in terms of atom economy and gaining molecular complexity.^1^ Because of the gaseous, reactive, and toxic nature of anhydrous formaldehyde, performing this reaction is far from straightforward, and thus, easier-to-handle formaldehyde sources are usually engaged. However, despite their practicality, aqueous formaldehyde solutions may cause incompatibility issues with many catalytic systems due to the presence of water and methanol as a stabilizer. As a cyclic formaldehyde trimer, trioxane is activated by means of acid, but it is chemically inert in a neutral or alkaline environment. Paraformaldehyde, a polymeric precursor of formaldehyde, has limited solubility in many organic solvents. The lower rate of its depolymerization, especially under mild conditions, may also slow the reaction down.^2^ For these reasons, some research on bench-stable, soluble, and reactive formaldehyde surrogates is highly desired. Such surrogates offer a convenient source of anhydrous formaldehyde, which can be generated *in situ* by using a base. Although some formaldehyde surrogates are mentioned in the literature,^3^ their use in asymmetric organocatalytic hydroxymethylations has not been systematically investigated so far. Moreover, all contemporary hydroxymethylation methods employing formaldehyde surrogates necessitate 1 equiv or more of the sacrificial base, which restricts their application if only a catalytic amount thereof is demanded.

Compared with aliphatic or alicyclic carbonyl compounds,^4^ the asymmetric organocatalyzed hydroxymethylation of heterocyclic substrates is generally an underdeveloped area, as illustrated in Scheme 1. This is evident, especially for isoindolinones (entry a), which, as difficult substrates, gained considerably less attention than their oxindole counterparts (entries b–f).^5,6^ As a consequence, to our knowledge, there is only one prior procedure for the asymmetric hydroxymethylation of isoindolinones.^5^ This reaction was pioneered by Massa and co-workers in 2018 and involved the Takemoto catalyst and paraformaldehyde. Requiring a prolonged reaction time (7 days), the substrate scope of the above methodology was limited to two isoindolinones only, and the resulting adducts were delivered with low to moderate enantioenrichment (<78.5:21.5 e.r.).

**Scheme 1:**
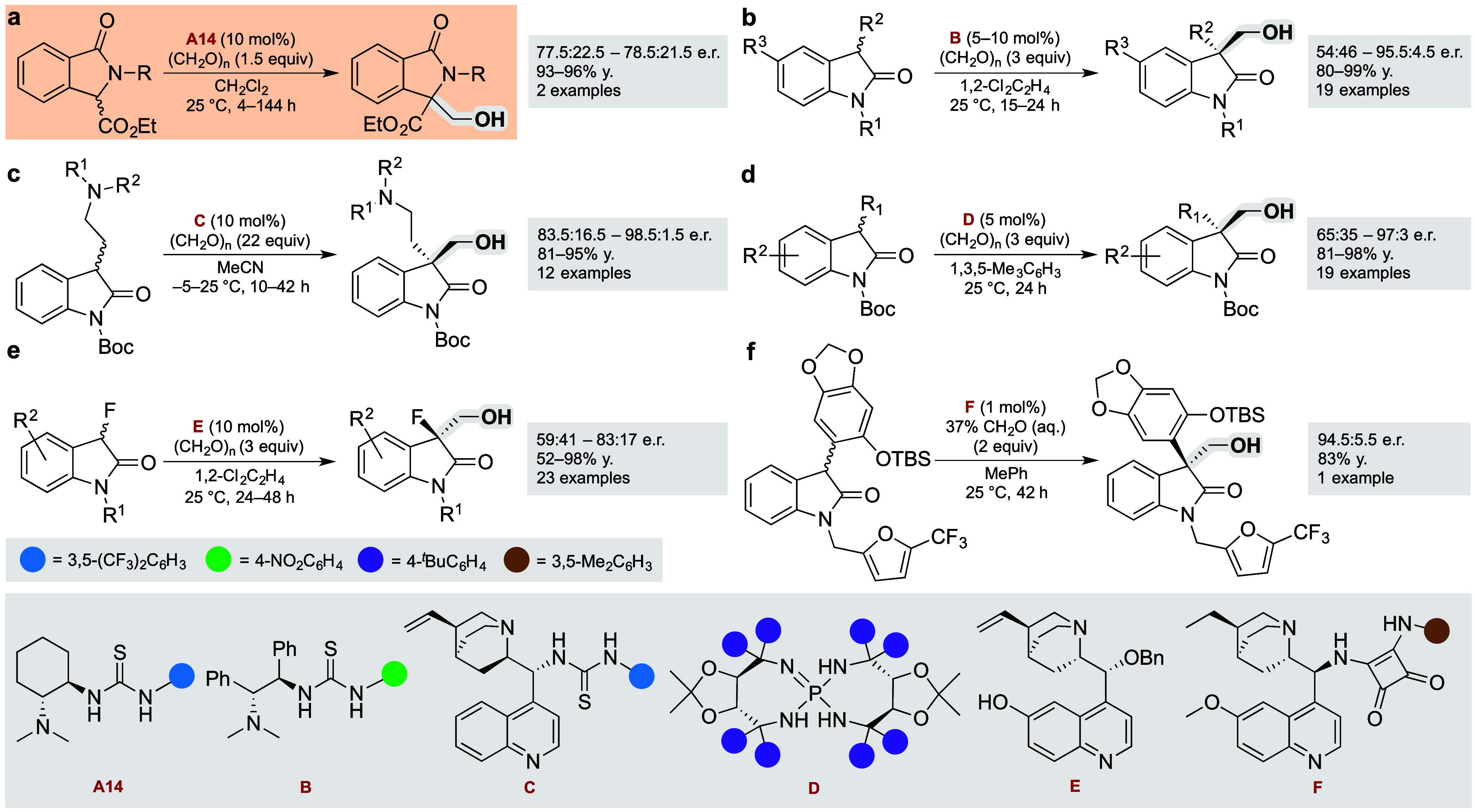
Overview of the Asymmetric Organocatalyzed Hydroxymethylations of Isoindolinones and Oxindoles Massa and co-workers, 2019.^5^ Yuan and co-workers, 2010.^6g^ Bisai and co-workers, 2015.^6f^ Wang and co-workers, 2016.^6e^ Ren, Li, and co-workers, 2018.^6d^ Herrera, Bernardi, and co-workers, 2022.^6a^

Considering the attractiveness of the isoindolinone scaffold for medicinal chemistry^7^ and driven by our long-term interest in asymmetric aldol-type processes,^8^ we provide herein a feasible solution to this challenging task.

Our attempts to apply the reaction conditions described by Massa et al.^5^ on 3-cyanoisoindolinones were unsuccessful and led to only traces of the hydroxymethylated products (68:32 e.r., <10% yield) after 24 h. Thus, we soon realized that a modified reaction setup would be needed to increase the reaction rate and broaden the scope meaningfully.

Inspired by the work of Bischoff and co-workers,^3e^ we were pleased by preliminary findings that the hydroxymethylation of **1a** can be speeded up substantially using suitable formaldehyde surrogates derived from nitrogen heterocycles, indicating that the slow chain unzipping of paraformaldehyde may constitute here a possible rate-limiting step. Surrogate **3a**, discovered in the early stages of our study, offered reasonable reactivity and easy removal from the crude reaction mixture by filtration through a silica plug. Thus, it was employed in catalyst screening henceforth.

An inceptive search of chiral organocatalysts **A1**–**25** (Figure S1) gave us strong evidence that Takemoto-type molecules (**A14**) were superior to other investigated skeletons in regard to the asymmetric induction (87:13 e.r., 30% yield). Follow-up structural optimizations of **A14**, as showcased in Figures S2–S5, furnished the piperidine-based catalyst **A50** as the best candidate for the subsequent experiments (90:10 e.r., 29% yield).

Alongside catalysts, we commenced with screening of formaldehyde surrogates (**3**–**5**) under the model reaction conditions involving **1a** and **A14** (20 mol %), as shown in Figure S6. *O*-Alkylated surrogates (**3m**, **4d**, **4e**) turned out to be completely unreactive, demonstrating that the free hydroxy group is among the surrogate’s essential attributes regarding the release of formaldehyde under base catalysis of **A14**. Surrogates **3c** and **3d** were also ineffective hydroxymethylation reagents. This is in accordance with the earlier report^3e^ proposing that cleavage of the surrogate in basic conditions is driven by the increased thermodynamic stability of the anion of the surrogate’s leaving group in comparison with the alkoxide resulting from deprotonation thereof (*int*-**1***vsint*-**2**; Scheme 3). From this viewpoint, it is worth noting that surrogate **3o** gave merely traces of product **2a** (87:13 e.r., 5% yield), even though the properties of **3o** met the above hypothesis. The leaving group of **3o** (*i.e.*, saccharine) is a mild acid, having p*K*_a_ of around 1.6.^9^ Hence, it protonates fully the basic site of catalyst **A14**, making it unavailable for activation of pronucleophile **1a**, thereby ceasing the hydroxymethylation (entry 17, Table S2). On the other hand, surrogates with leaving groups possessing intrinsic basicity (*e.g.*, **3f**, **3g**, **3j**, or **3u**) often provided **2a** in good yield, albeit with somewhat reduced enantioselectivity. This was likely caused by their tendency toward an autocatalytic release of formaldehyde reflected in a considerable rate of the competing racemic background pathway (Table S1).

By the above, it is evident that a delicate balance between the acid–base properties of the surrogate and its leaving group ability was needed to create a controllable and convenient formaldehyde donor working under reaction conditions with a catalytic amount of base. These requirements were best fulfilled in the last group of surrogates (*i.e.*, **3b** and **3i**), which were bench-stable solids with defined chemical structures (not mixtures of regioisomers like **3k**, **3s**, *etc.*), evincing the background reaction to a negligible extent only (<5% yield). The conjugated acids of their leaving groups have p*K*_a_ within the range of 5–6,^10^ which are values low enough to ensure a strong driving force for the formaldehyde release (a, Scheme 3) while still high enough to maintain a sufficient fraction of base catalyst **A** in its nonionized form (b, Scheme 3), which is necessary for the hydroxymethyl transfer to **1** to run at a reasonable rate (c, Scheme 3). Accordingly, **3i** was chosen as an ideal surrogate for the onward experiments.^11^

With the proper catalyst **A50** and surrogate **3i** in hand, we investigated the rest of the reaction parameters (Tables S2–S4). In line with that, five sets of optimal conditions dictated by the reactivity of substrates were established, all using *tert*-butyl methyl ether as a solvent, **3i** (3 equiv), and **A50** (10–20 mol %) at room or slightly elevated temperature (Scheme 2).

**Scheme 2:**
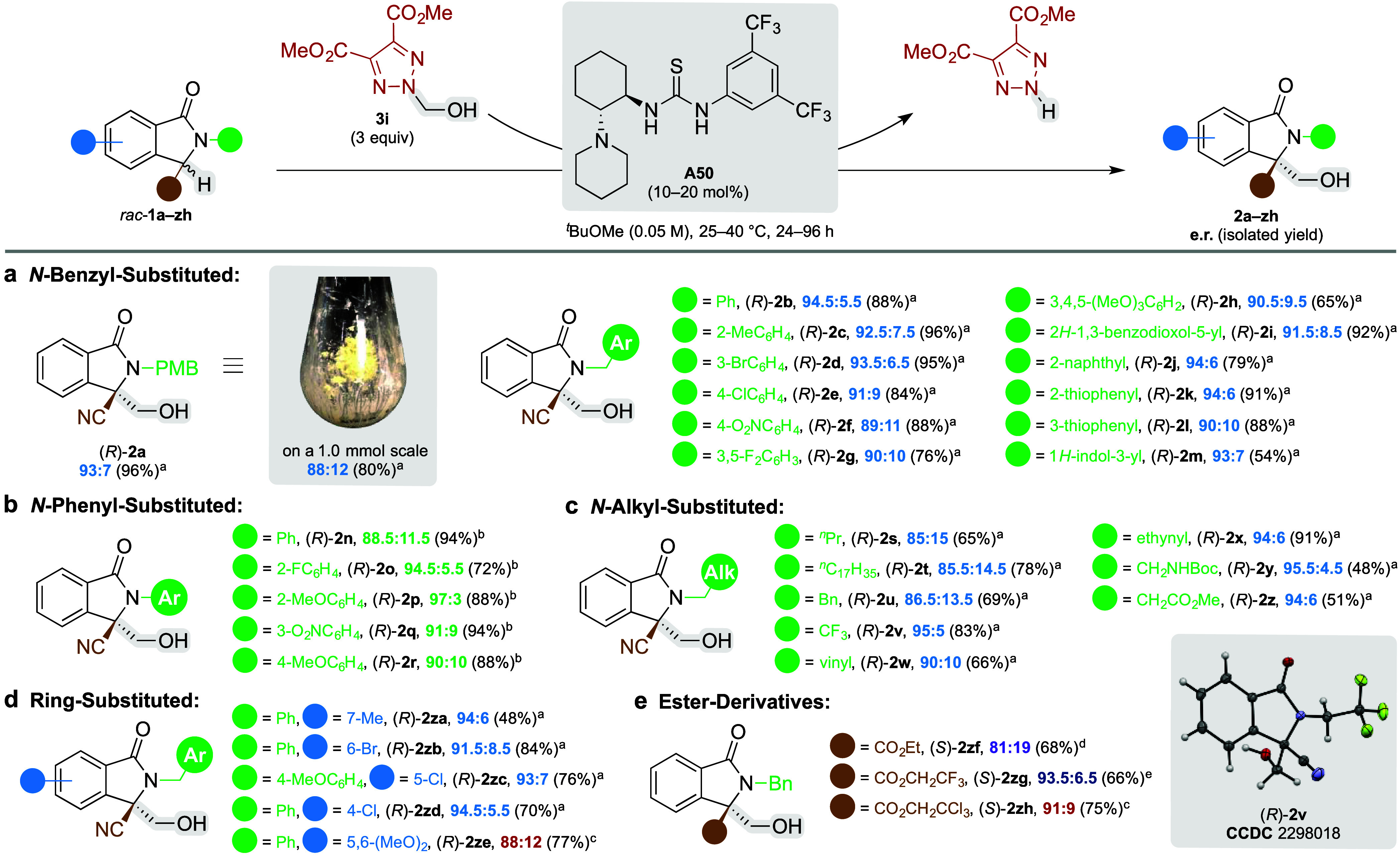
Substrate Scope of the Asymmetric Hydroxymethylation See the Supporting Information for full details. The reactions were standardly performed on a 0.05 mmol scale; e.r. values were determined by chiral HPLC analyses of the isolated products; the yields refer to the isolated products. Reaction conditions: (a) **A50** (10 mol %), 40 °C, 24 h; (b) **A50** (20 mol %), 25 °C, 48 h; (c) **A50** (20 mol %), 40 °C, 48 h; (d) **A50** (20 mol %), 40 °C, 96 h; (e) **A50** (20 mol %), 40 °C, 24 h.

Having the optimized conditions developed, we focused on the possible substrate scope. Considering isoindolinones, the presence of an electron-withdrawing group at position 3 was indispensable to render a stereocenter labile enough to be easily enolizable under the mildly basic environment (*int*-**3**; Scheme 3). Isoindolinones lacking the blocked nitrogen atom behaved unsatisfactorily, furnishing various portions of the respective *C*- and *N*-hydroxymethylated adducts (**XVIa** and **XVIb**), the former with 76.5:23.5 e.r. (Figure S20). Additionally, these mixtures were highly challenging to separate from reagent **3i** and its byproduct chromatographically.

Within the above structural constraints, the process proved to be quite general in scope, delivering products with good outcomes in terms of yields (48–96%) and enantioselectivities (81:19–97:3 e.r.). As depicted in Scheme 2, a broad range of *N*-substituents were well-tolerated. These included benzyl (**2a**–**i**), 2-naphthylmethyl (**2j**), thiophenylmethyl (**2k**, **2l**), phenyl (**2n**–**r**), alkyl (**2s**, **2t**), phenethyl (**2u**), polyfluoroalkyl (**2v**), allyl (**2w**), and propargyl (**2x**) groups. Moreover, substrates bearing unprotected 3-indolylmethyl (**2m**), *N*-Boc-masked amine (**2y**), or methyl ester (**2z**) were suitable as well. The same procedure worked for ring-substituted isoindolinones (**2za**–**zd**) too. Contrarily, more electron-rich **1ze** produced adduct **2ze** using a doubled loading of **A50** (20 mol %) and 48 h, which was also required for the corresponding ester derivatives (**2zf**–**zh**). Remarkably, although substrate **1zf** needed an even longer reaction time (96 h), product **2zf** was still gathered in nearly half of the previously reported period.^5^ We hypothesized that the moderate e.r. of **2zf** might be improved by the increased steric hindrance of its ester function, enhancing discrimination between prochiral faces of the intermediary enolate. Moreover, the bulky group containing electronegative atoms, such as halogens, could also stabilize the enolate ion through an inductive effect. Accordingly, 2,2,2-trihaloethyl ester derivatives (**2zg**, **2zh**) were prepared with the aforementioned reactivity and selectivity drawbacks widely removed.

The absolute configuration of (*R*)-**2v** determined from the anomalous scattering with a Flack parameter very close to zero allowed us to postulate a working hypothesis of the stereochemical model (*int*-**4**; Scheme 3). The remaining hydroxymethylated products were all assigned by analogy thereto (Scheme 2). In connection with our preliminary mechanistic considerations (Figures S7–S16) and the previous work regarding electrophilic trapping of leaving groups from the analogous reagents,^3e,12^ we assume that formaldehyde extrusion rather than direct nucleophilic attack on the deprotonated surrogate is instrumental under the given conditions (a–c, Scheme 3).

**Scheme 3:**
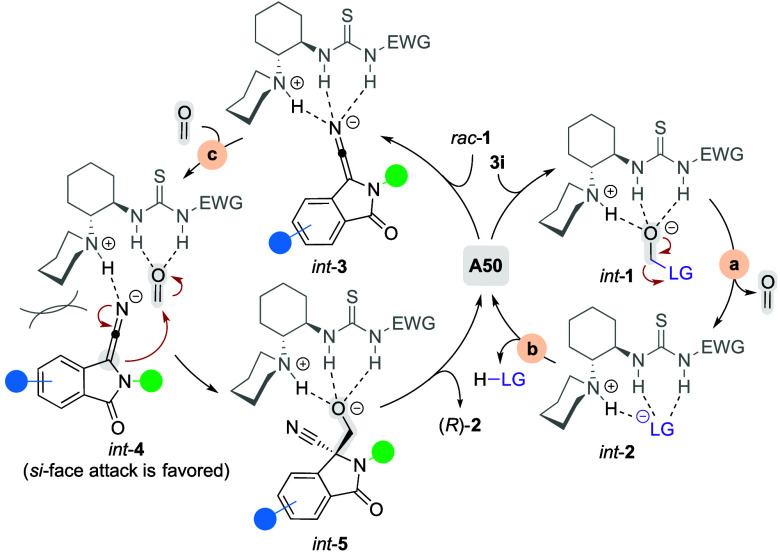
Proposed Catalytic Cycle

Taking into account a negligible rate of the background reaction in the absence of base and principal reversibility of hemiaminal formation and the subsequent aldol-type process in the basic environment,^13^**A50** seems to operate in both the surrogate and isoindolinone cycle with formaldehyde being the transferred group (Scheme 3).

In the next task, as outlined in Scheme 4, we surveyed the possible downstream transformations of the enantioenriched products. Noteworthily, these reactions served us as a proof of concept. Thus, no attempts were made to optimize the yields further. Compound (*R*)-**2a** underwent 100% enantiospecific *N*-deprotection with ceric ammonium nitrate (CAN) smoothly, which was in contrast with (*R*)-**2r**, where the same conditions resulted in a full recovery of the starting material. Likewise, Radziszewski amidation of (*R*)-**2a** gave amide (*S*)-**7** with complete stereoretention. The free hydroxy groups of adducts (*R*)-**2a**, (*R*)-**2b**, and (*R*)-**2m** were also acetylated, methylated, and silylated, respectively, without any detectable deterioration of their initial enantiopurities. On the other hand, the attempt to oxidize (*R*)-**2a** with Dess–Martin periodinane (DMP) was unsuccessful, ending up in deformylation back to *rac*-**1a**. Racemization of the enriched adducts caused by the propensity toward retro-aldol cleavage (Figures S13–S16) was also noticed through their exposure to stronger inorganic bases, such as NaOH or K_3_PO_4_, especially during the prolonged periods of heating, *e.g.*, in the Suzuki–Miyaura cross-coupling of (*R*)-**2zb** with 4-methoxyphenylboronic acid. Despite our extensive experimentation on possible leaving groups, we were unable to find any convenient conditions for nucleophilic substitution at the α-carbon of the hydroxymethyl unit, which can be rationalized by its hindered, neopentyl-like character.

**Scheme 4:**
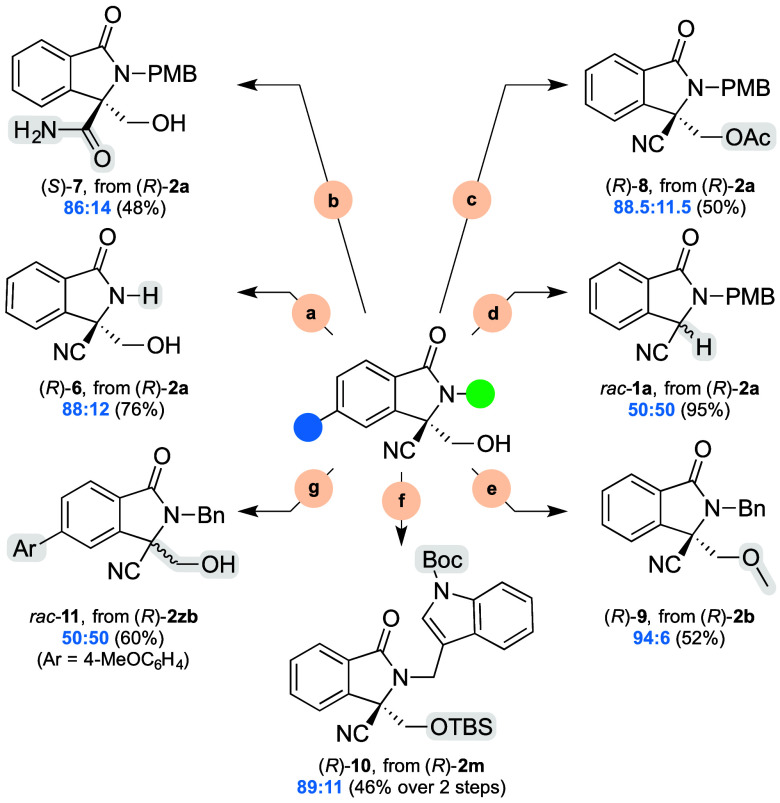
Downstream Transformations of the Adducts See the Supporting Information for full details. Reaction conditions: (a) CAN, MeCN–H_2_O, 0–25 °C, 3.5 h; (b) Na_2_CO_3_, EtOH–30% H_2_O_2_, 0–25 °C, 12 h; (c) AcCl, DMAP, CH_2_Cl_2_, 0–25 °C, 24 h; (d) DMP, CH_2_Cl_2_, 25 °C, 2 h; (e) CH_2_N_2_ in CH_2_Cl_2_, 48% HBF_4_, CH_2_Cl_2_, 0 °C, 4 h; (f) TBSCl, imidazole, CH_2_Cl_2_, 40 °C, 12 h, then (Boc)_2_O, DMAP, CH_2_Cl_2_, 25 °C, 1 h; (g) 4-MeOC_6_H_4_B(OH)_2_, Pd(OAc)_2_, PPh_3_, K_3_PO_4_, 1,4-dioxane–H_2_O, 80 °C, 4 h.

In conclusion, we have disclosed a novel process for the asymmetric transfer hydroxymethylation between formaldehyde surrogates and activated isoindolinones. The enantioenriched hydroxymethylated adducts were delivered in good to excellent yields (48–96%) and enantiopurities (81:19–97:3 e.r.). The substrate scope tested on 34 entries showed the considerable generality of the developed asymmetric transformation. A scale-up experiment and multiple enantioselective downstream transformations were also carried out, suggesting the prospective synthetic utility of the products.

## Data Availability

The data underlying this study are available in the published article and its Supporting Information.

## Supporting Information Available

Experimental procedures, additional optimization data, characterization for all compounds, and chiral HPLC data (PDF)
