# Supplementary material for: Asymmetric Organocatalyzed Transfer Hydroxymethylation of Isoindolinones Using Formaldehyde Surrogates
Source: Org Lett. 2024 Mar 19;26(12):2505–10. doi: 10.1021/acs.orglett.4c00818 (PMC10985653; doi:10.1021/acs.orglett.4c00818)
Supplement: Supplementary file 1 — ol4c00818_si_001.pdf [file ol4c00818_si_001.pdf]

SUPPORTING INFORMATION

Asymmetric Organocatalyzed Transfer Hydroxymethylation of Isoindolinones Using Formaldehyde Surrogates

David Svestka[1], Pavel Bobal[1]*, Mario Waser[2], Jan Otevrel[1]*

*corresponding authors:
otevrelj@pharm.muni.cz, bobalp@pharm.muni.cz

[1]*Department of Chemical Drugs, Faculty of Pharmacy, Masaryk University, Palackeho 1, 612 00 Brno, CZ*

[2]*Institute of Organic Chemistry, Johannes Kepler University, Altenbergerstrasse 69, 4040 Linz, AT*

Table of Contents

1. General Information **3**
1.1. General Methods 3
2. Optimization of the Reaction Conditions **4**
2.1 Catalyst Screening 4
2.2 Formaldehyde Surrogate Screening 8
2.3 Investigations on the Racemic Background Reaction 9
2.4 Additives Screening 10
2.5 Solvent Optimization 11
2.6 Optimization of the Remaining Reaction Conditions 12
2.7 Preliminary Estimation of Surrogate **3i** – Formaldehyde Equilibria 13
2.8 Formaldehyde Trapping 16
2.9 Stability of **3i** Under Basic Conditions 17
2.10 Determination of the Reaction Reversibility by Crossover Experiments 19
3. Miscellaneous Syntheses **24**
3.1 Syntheses of Catalysts 24
3.2 Syntheses of Formaldehyde Surrogates 34
3.3 Syntheses of Isoindolinones 39
3.4 Syntheses of Racemic Products 52
3.5 Syntheses of Enantioenriched Products 52
4. Examples of Unsuitable Substrates **69**
4.1 Substrates Exhibiting No Reactivity 69
4.2 Substrates Exhibiting Insufficient Enantioselectivity 69
5. A Scale-up Experiment for Adduct (*R*)-2a **72**
6. Downstream Transformations of Enantioenriched Adducts **73**
6.1 *N*-Deprotection of (*R*)-**2a** 73
6.2 Radziszewski Amidation of (*R*)-**2a** 74
6.3 *O*-Acetylation of (*R*)-**2a** 75
6.4 Oxidation of (*R*)-**2a** with DMP 76
6.5 *O*-Methylation of (*R*)-**2b** 77
6.6 *O*-TBS and *N*-Boc Diprotection of (*R*)-**2m** 78
6.7 Suzuki Cross-Coupling of (*R*)-**2zb** 79
7. X-Ray Single-Crystal Analysis **80**
8. Copies of the Product NMR Spectra **82**
9. Copies of HPLC Chromatograms **207**
10. Copies of the Product MS Spectra **251**
11. References **278**

1. General Information

1.1. General Methods

^{1}H, ^{13}C, ^{19}F, and ^{31}P NMR spectra were recorded on a Bruker Avance III 300 MHz spectrometer (Bruker), a Bruker Avance DRX 500 MHz spectrometer (Bruker), or a JEOL ECZR-400 MHz spectrometer (Jeol). NMR experiments were standardly performed at 25 °C, chemical shifts are reported in δ parts per million (ppm) and *J* values in Hz, the signal of TMS or the residual solvent signals of $CDCl_3$, C_6D_6, DMSO-d_6, MeOH-d_4, or THF-d_8 were used as a reference. Mass spectra were obtained using an LTQ Orbitrap XL high-resolution mass spectrometer (ThermoFisher Scientific), a Bruker Impact II Q-TOF high-resolution mass spectrometer (Bruker Daltonics), and an Agilent 6224 Accurate-Mass TOF LC-MS (Agilent Technologies) mass spectrometer. HPLC was performed using a Dionex UltiMate 3000 LC System and a Spectra-System (ThermoFisher Scientific). The specific rotation was determined by an automatic polarimeter AA-10 (Optical Activity). Melting points were measured by a Böetius apparatus (Franz Küstner Nachf.) and are uncorrected. As described in our previous report,[1] small-scale catalytic experiments were performed in screw-cap vials equipped with magnetic stirring bars with the reaction temperature maintained by a thermostatic circulating water bath Cool Tech 320 Chiller (Thermo Scientific).

Catalysts **A1** (CAS 848821-76-1), **A2** (CAS 346440-54-8), **A8** (CAS 950194-37-3), **A13** (CAS 934762-68-2), **A14** (CAS 620960-26-1), **A17** (CAS 2227157-06-2), **A19** (CAS 118-10-5), **A20** (CAS 130-95-0), and **A34** (CAS 1211565-08-0); surrogates **3l** (CAS 118-29-6) and **3r** (CAS 28539-02-8) were supplied from Merck. Catalysts **A3** (CAS 1110711-01-7) and **A49** (CAS 620960-27-2) were supplied from Strem Chemicals. Catalyst **A4** (CAS 1197922-04-5) was supplied from TCI Europe N.V. Catalyst **A5** (CAS 287384-12-7) was supplied from FUJIFILM Wako Pure Chemical Corporation. Catalysts **A9** (CAS 791616-63-2), **A10** (CAS 1242066-20-1), **A32** (CAS 1263205-96-4), **A35** (CAS 834917-24-7), **A36** (CAS 1048692-60-9), **A37** (CAS 1263205-97-5), **A48** (CAS 1314743-49-1), **A50** (CAS 1289514-24-4), **A56** (CAS 1233369-39-5), **A57** (CAS 1429516-79-9), and **A60** (CAS 1069114-13-1) were supplied from Ambeed. Catalysts **A6**,[2] **A7**,[3] **A11–12**,[4] **A15–16**,[4] **A18**,[5] **A21–25**,[6] **A46**,[7] **A47**,[8] **A51**,[9] **A53**,[10] **A58**,[8] **A59**,[11] and surrogate **4c**[12] were prepared according to the literature.

All other chemicals were purchased from commercial vendors (Acros Organics, Ambeed, Fluorochem, Merck) and used without further purification unless otherwise stated.

All experiments were performed standardly under open-vessel conditions. Moisture and air-sensitive reactions were done in oven-dried glassware (140 °C) under an Ar atmosphere in anhydrous solvents. Anhydrous solvents and reagents were absolutized as usual and distilled prior to use. A diazomethane solution was prepared using the MNNG-Diazomethane Generation Apparatus (Z101001).

2. Optimization of the Reaction Conditions

2.1 Catalyst Screening

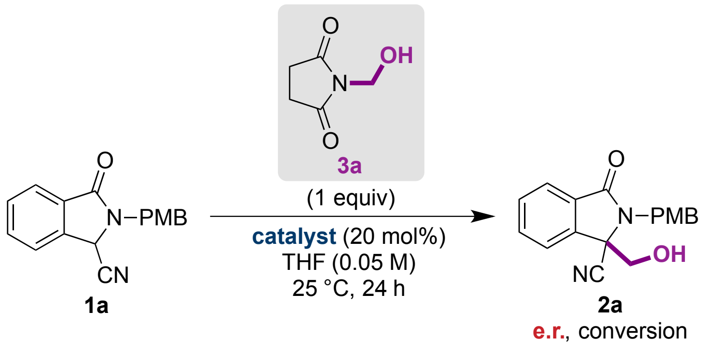

Screening of catalysts was carried out using the following procedure. Substrate **1a** (0.025 mmol, 6.9 mg), formaldehyde surrogate **3a** (0.025 mmol, 3.4 mg), and the corresponding catalyst **A** (0.005 mmol, see **Figs. S1–5**) were dissolved or suspended in THF (500 µL). The resulting reaction mixture was stirred at room temperature (rt) for 24 h. Then, the reaction was quenched by the addition of a saturated aqueous solution of NH_4Cl and repetitively extracted with EtOAc. The combined organic extracts were dried over anhydrous Na_2SO_4, filtered through a pad of SiO_2, evaporated *in vacuo*, and subjected to HPLC analysis (see **Figs. S1–5** for details regarding conversion and e.r.).

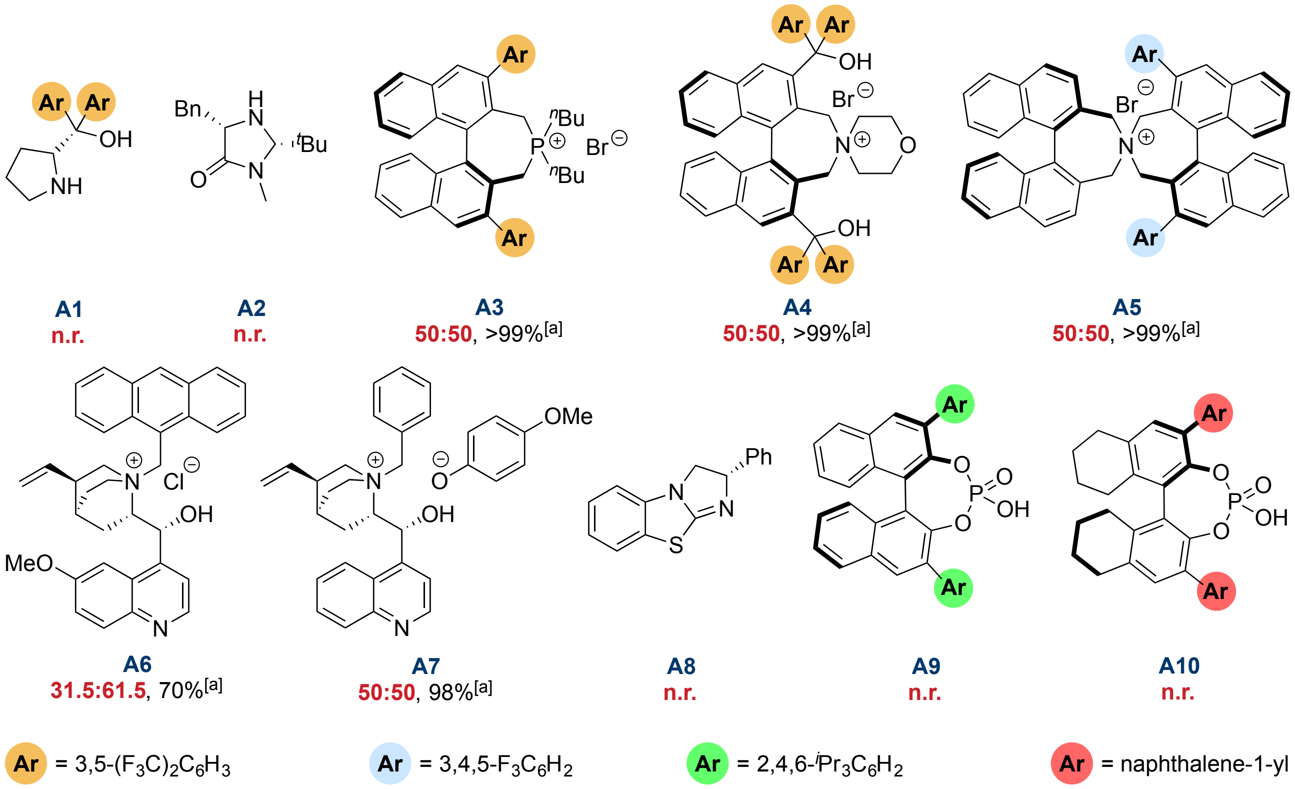

Figure S1: Searching for a suitable catalyst backbone. Notes: [a] K_2CO_3 (1 equiv) was added to the reaction mixture as an external base; e.r. values were analyzed by HPLC with CSP; e.r. values reflect the elution order of enantiomers, the first number corresponds to (*R*)-enantiomer and the second to (*S*)-enantiomer; conversion of **1a** to **2a** was determined by RP-HPLC analyses of the crude reaction mixtures with external standard calibration, relative response factor (RRF) = 1.03; n.r. stands for no reaction.

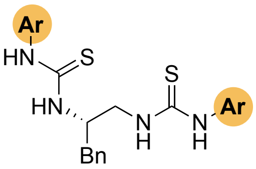

A11
45:55, 13%[b]

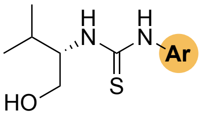

A12
45.5:55.5, 5%[b]

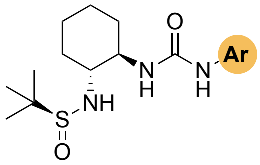

A13
48:52, 20%[b]

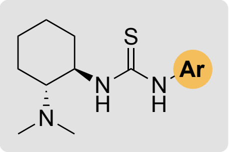

A14
83:17, 30%

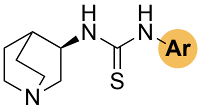

A15
52.5:47.5, 33%

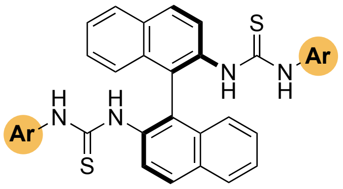

A16
n.r.[b]

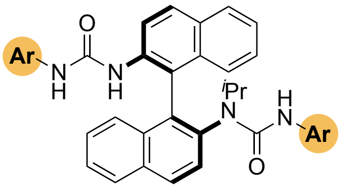

A17
50:50, 42%[b]

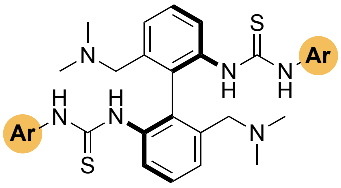

A18
45.5:55.5, 14%

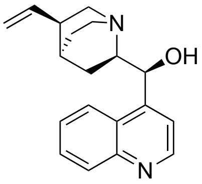

A19
53:47, 22%

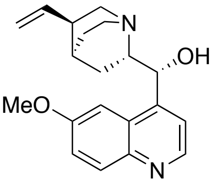

A20
30:70, 29%

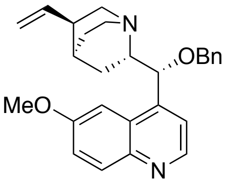

A21
48:52, 4%

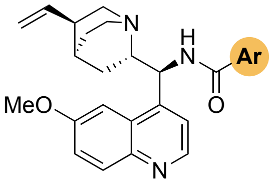

A22
35:65, 6%

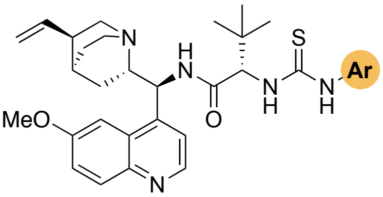

A23
46:54, 13%

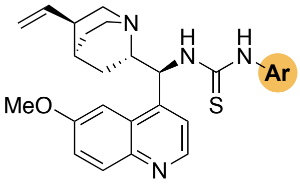

A24
25:75, 27%

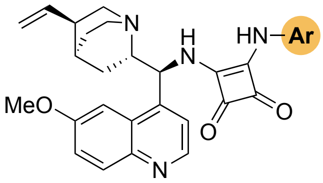

A25
48:52, 29%

Ar = 3,5-$(F_3C)_2C_6H_3$

Figure S1, cont'd: Searching for a suitable catalyst backbone. Notes: [b] DIPEA (20 mol%) was added to the reaction mixture as an external base; e.r. values were analyzed by HPLC with CSP; e.r. values reflect the elution order of enantiomers, the first number corresponds to (*R*)-enantiomer and the second to (*S*)-enantiomer; conversion of **1a** to **2a** was determined by RP-HPLC analyses of the crude reaction mixtures with external standard calibration, relative response factor (RRF) = 1.03; n.r. stands for no reaction.

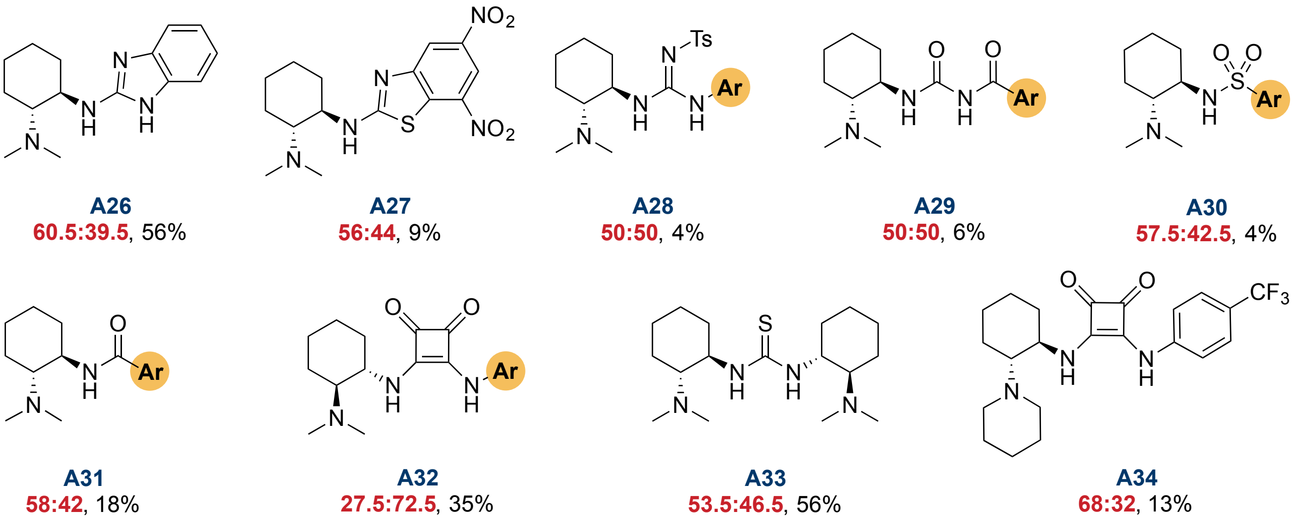

Ar = 3,5-$(F_3C)_2C_6H_3$

Figure S2: Variating of the catalyst H-boding site. Notes: e.r. values were analyzed by HPLC with CSP; e.r. values reflect the elution order of enantiomers, the first number corresponds to (*R*)-enantiomer and the second to (*S*)-enantiomer; conversion of **1a** to **2a** was determined by RP-HPLC analyses of the crude reaction mixtures with external standard calibration, RRF = 1.03.

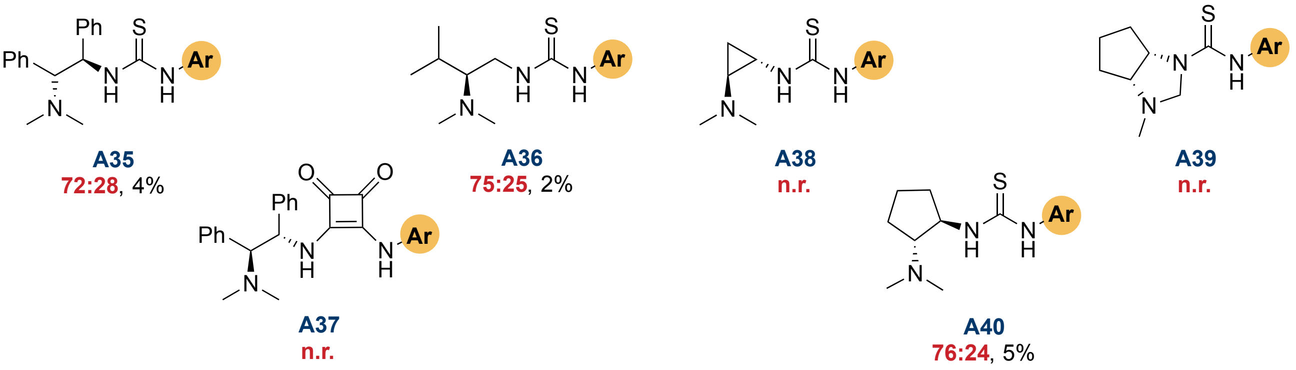

Ar = 3,5-$(F_3C)_2C_6H_3$

Figure S3: Variating of the catalyst 1,2-diamine core. Notes: e.r. values were analyzed by HPLC with CSP; e.r. values reflect the elution order of enantiomers, the first number corresponds to (*R*)-enantiomer and the second to (*S*)-enantiomer; conversion of **1a** to **2a** was determined by RP-HPLC analyses of the crude reaction mixtures with external standard calibration, RRF = 1.03; n.r. stands for no reaction.

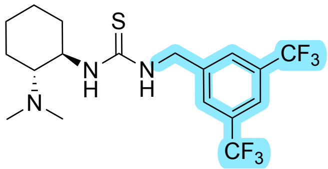

A41
79:21, 48%

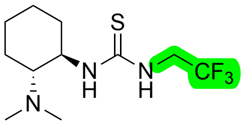

A42
78.5:21.5, 22%

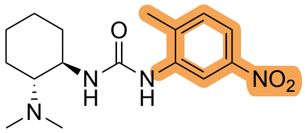

A43
78.5:21.5, 47%

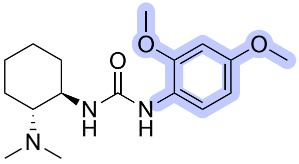

A44
61.5:38.5, 31%

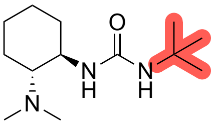

A45
75:25, 36%

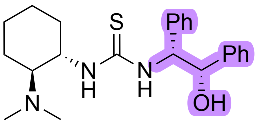

A46
44.5:55.5, 56%

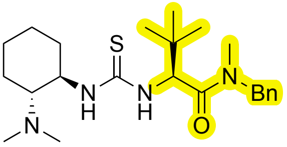

A47
46:54, 22%

Figure S4: Variating of the (thio)urea *N*-substituent. Notes: e.r. values were analyzed by HPLC with CSP; e.r. values reflect the elution order of enantiomers, the first number corresponds to (*R*)-enantiomer and the second to (*S*)-enantiomer; conversion of **1a** to **2a** was determined by RP-HPLC analyses of the crude reaction mixtures with external standard calibration, RRF = 1.03.

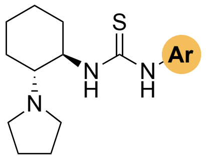

A48
88:12, 54%

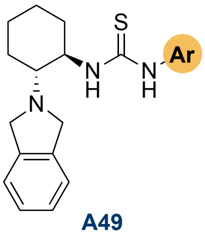

A49
n.r.

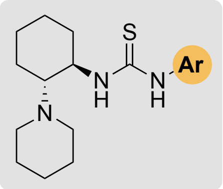

A50
90:10, 29%

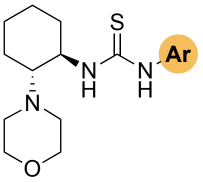

A51
74:26, 2%

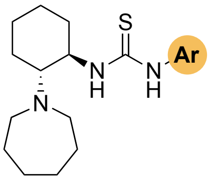

A52
85:15, 31%

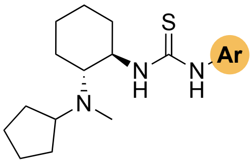

A53
87.5:12.5, 42%

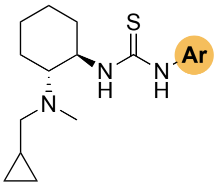

A54
76:24, 29%

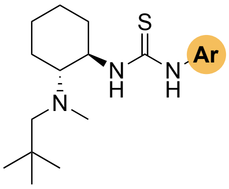

A55
63:37, 6%

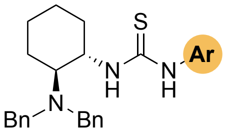

A56
n.r.

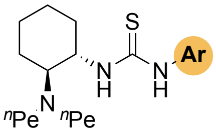

A57
19:81, 17%

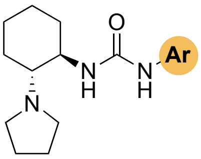

A58
80:20, 68%

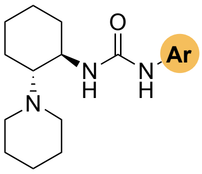

A 59
80:20, 27%

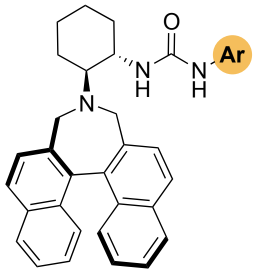

A60
68:32, 3%

Ar = 3,5-$(F_3C)_2C_6H_3$

Figure S5: Optimization of the catalyst basic moiety. Notes: e.r. values were analyzed by HPLC with CSP; e.r. values reflect the elution order of enantiomers, the first number corresponds to (*R*)-enantiomer and the second to (*S*)-enantiomer; conversion of **1a** to **2a** was determined by RP-HPLC analyses of the crude reaction mixtures with external standard calibration, RRF = 1.03; n.r. stands for no reaction.

2.2 Formaldehyde Surrogate Screening

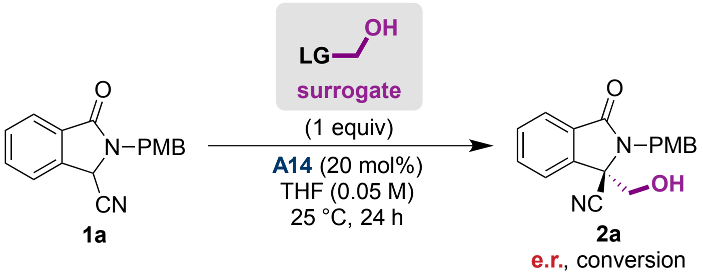

Screening of formaldehyde surrogates was carried out using the following procedure. Substrate **1a** (0.025 mmol, 6.9 mg), the corresponding formaldehyde surrogate (0.025 mmol, see **Fig. S6**), and catalyst **A14** (0.005 mmol, 2.1 mg) were dissolved or suspended in THF (500 µL). The resulting reaction mixture was stirred at rt for 24 h. Then, the reaction was quenched by the addition of a saturated aqueous solution of NH_4Cl and repetitively extracted with EtOAc. The combined organic extracts were dried over anhydrous Na_2SO_4, filtered through a pad of SiO_2, evaporated *in vacuo*, and subjected to HPLC analysis (see **Fig. S6** for details regarding conversion and e.r.).

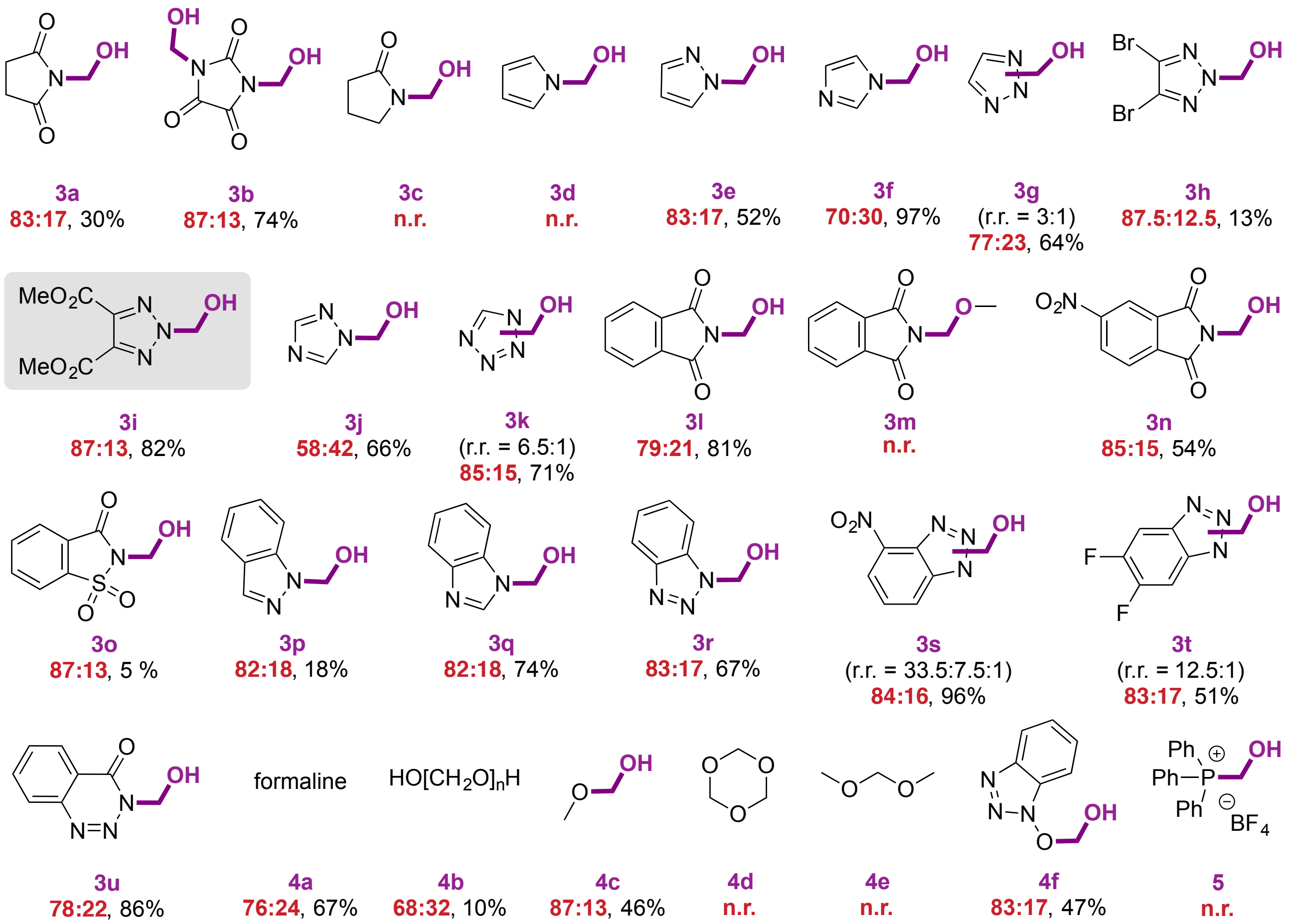

Figure S6: Optimization of the formaldehyde surrogate. Notes: e.r. values were analyzed by HPLC with CSP; e.r. values reflect the elution order of enantiomers, the first number corresponds to (*R*)-enantiomer and the second to (*S*)-enantiomer; conversion of **1a** to **2a** was determined by RP-HPLC analyses of the crude reaction mixtures with external standard calibration, RRF = 1.03; r.r. stands for ratio of regioisomers as determined by ^{1}H NMR.

2.3 Investigations on the Racemic Background Reaction

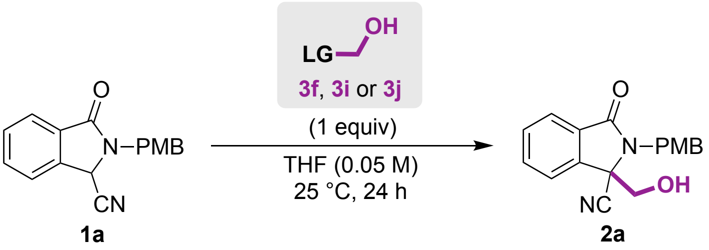

Substrate **1a** (0.025 mmol, 6.9 mg) and formaldehyde surrogate **3f**, **3i** or **3j** (0.025 mmol, see **Tab. S1**) were dissolved in THF (500 μL). The resulting reaction mixture was allowed to stir at rt for 24 h. Then, the reaction was quenched by the addition of a saturated aqueous solution of NH_4Cl and repetitively extracted with EtOAc. The combined organic extracts were dried over anhydrous Na_2SO_4, filtered through a pad of SiO_2, evaporated *in vacuo*, and subjected to HPLC analysis (see **Tab. S1** for details regarding conversion).

Table S1: The extent of the racemic background reaction. Notes: conversion of **1a** to **2a** was determined by RP-HPLC analyses of the crude reaction mixtures with external standard calibration, RRF = 1.03.

entry	surrogate	conv. / %
**1**	**3f**	90
**2**	**3i**	3
**3**	**3j**	55

2.4 Additives Screening

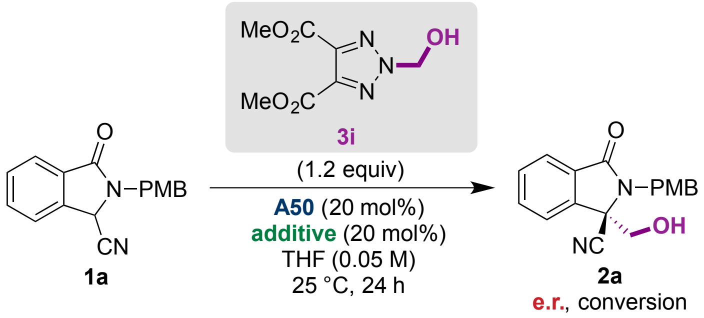

Screening of additives was carried out using the following procedure. Substrate **1a** (0.025 mmol, 6.9 mg), formaldehyde surrogate **3i** (0.030 mmol, 6.5 mg), catalyst **A50** (0.005 mmol, 2.3 mg), and the corresponding additive (0.005 mmol, see **Tab. S2**) were dissolved or suspended in THF (500 µL). The resulting reaction mixture was stirred at rt for 24 h. Then, the reaction was quenched by the addition of a saturated aqueous solution of NH_4Cl and repetitively extracted with EtOAc. The combined organic extracts were dried over anhydrous Na_2SO_4, filtered through a pad of SiO_2, evaporated *in vacuo*, and subjected to HPLC analysis (see **Tab. S2** for details regarding conversion and e.r.).

Table S2: Screening of potential additives. Notes: [a] 1.0 equiv was used; e.r. values were analyzed by HPLC with CSP; e.r. values reflect the elution order of enantiomers, the first number corresponds to (*R*) and the second to (*S*)-enantiomer; conversion of **1a** to **2a** was determined by RP-HPLC analyses of the crude reaction mixtures with external standard calibration, RRF = 1.03; n.d. stands for not determined.

entry	additive	conv. / %	e.r.
**4**	pyrocatechol	60	94 : 6
**5**	2,2-dimethylpropane-1,3-diol	52	94 : 6
**6**	hexafluoroisopropanol	55	95 : 5
**7**	LiBr	48	92 : 8
**8**	CsF	74	93 : 7
**9**	$MgSO_4$	50	92.5 : 7.5
**10**	zinc(II) trifluoromethanesulfonate	0	n.d.
**11**	*N*,*O*-bis(trimethylsilyl)acetamide	54	93 : 7
**12**	2,4-dimethoxybenzoic acid	50	94 : 6
**13**	methoxyacetic acid	46	94 : 6
**14**	trifluoroacetic acid	0	n.d.
**15**	Schreiner thiourea	55	94 : 6
**16**	TBAB[a]	71	52 : 48
**17**	saccharine[a]	0	n.d.

2.5 Solvent Optimization

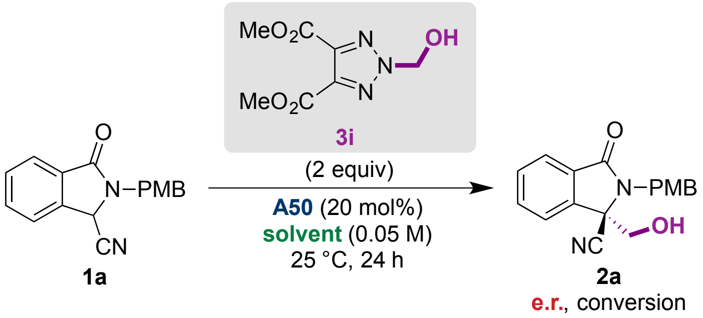

Screening of solvents was carried out using the following procedure. Substrate **1a** (0.025 mmol, 6.9 mg), formaldehyde surrogate **3i** (0.050 mmol, 10.8 mg), and catalyst **A50** (0.005 mmol, 2.3 mg) were dissolved or suspended in the corresponding solvent (500 µL, see **Tab. S3**). The resulting reaction mixture was stirred at rt for 24 h. Then, the reaction was quenched by the addition of a saturated aqueous solution of NH_4Cl and repetitively extracted with EtOAc. The combined organic extracts were dried over anhydrous Na_2SO_4, filtered through a pad of SiO_2, evaporated *in vacuo*, and subjected to HPLC analysis (see **Tab. S3** for details regarding conversion and e.r.).

Table S3: Solvent optimization. Notes: e.r. values were analyzed by HPLC with CSP; e.r. values reflect the elution order of enantiomers, the first number corresponds to (*R*)-enantiomer and the second to (*S*)-enantiomer; conversion of **1a** to **2a** was determined by RP-HPLC analyses of the crude reaction mixtures with external standard calibration, RRF = 1.03.

entry	solvent	conv. / %	e.r.
**18**	toluene	74	85 : 15
**19**	dichloromethane	75	86 : 14
**20**	acetonitrile	87	85 : 15
**21**	tetrahydrofuran	83	90 : 10
**22**	diethyl ether	85	92 : 8
**23**	*tert*-butyl methyl ether	89	94 : 6
**24**	1,2-dimethoxyethane	85	90 : 10
**25**	diisopropyl ether	72	90 : 10
**26**	di-*n*-butyl ether	35	83 : 17
**27**	cyclopentyl methyl ether	30	89 : 11

2.6 Optimization of the Remaining Reaction Conditions

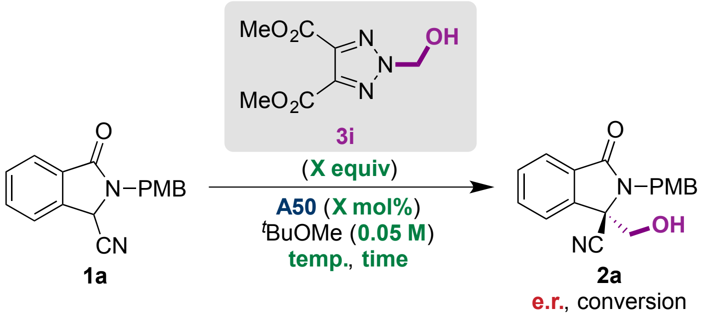

Optimization of remaining reaction conditions was carried out using the following procedure. Substrate **1a** (0.025 mmol, 6.9 mg), formaldehyde surrogate **3i** (see **Tab. S4**), and catalyst **A50** (see **Tab. S4**) were dissolved in *tert*-butyl methyl ether (500 µL). The resulting reaction mixture was stirred at 10, 25, or 40 °C for 24 or 48 h (see **Tab. S4**). Then, the reaction was quenched by the addition of a saturated aqueous solution of NH_4Cl and repetitively extracted with EtOAc. The combined organic extracts were dried over anhydrous Na_2SO_4, filtered through a pad of SiO_2, evaporated *in vacuo*, and subjected to HPLC analysis (see **Tab. S4** for details regarding conversion and e.r.).

Table S4: Optimization of remaining reaction conditions. Notes: [a] a thermostatic circulating water bath was used to maintain the reaction temperature; e.r. values were analyzed by HPLC with CSP; e.r. values reflect the elution order of enantiomers, the first number corresponds to (*R*) and the second to (*S*)-enantiomer; conversion of **1a** to **2a** was determined by RP-HPLC analyses of the crude reaction mixtures with external standard calibration, RRF = 1.03.

entry	3i / mmol	A50 / mol%	temp. / °C[a]	time / h	conv. / %	e.r.
**28**	0.050	20	10	24	32	94 : 6
**29**	0.075	20	25	24	98	94 : 6
**30**	0.075	10	25	48	88	95 : 5
**31**	0.075	20	40	24	>99	94 : 6
**32**	0.075	10	40	24	>99	94 : 6
**33**	0.075	5	40	24	98	94 : 6

2.7 Preliminary Estimation of Surrogate 3i – Formaldehyde Equilibria

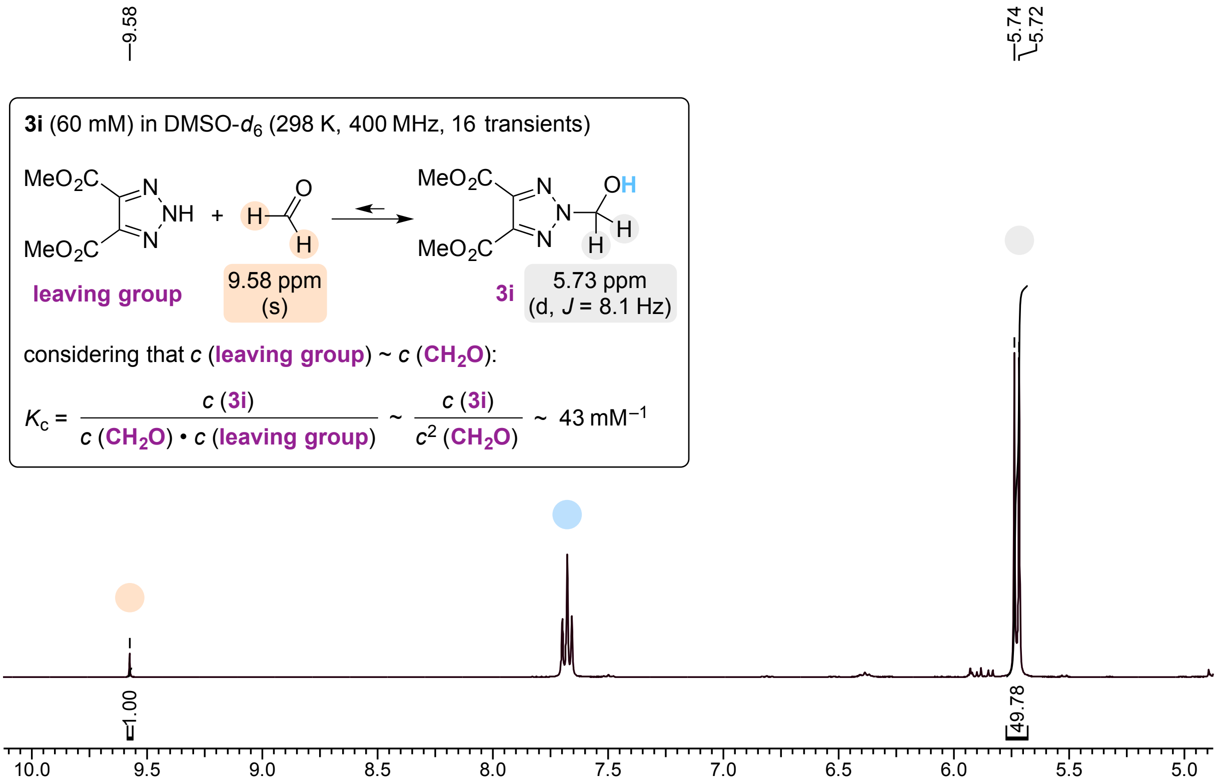

Figure S7. An inset of the ^{1}H NMR spectrum of **3i** in a range of 10.00–5.00 ppm showing equilibrium between **3i** and formaldehyde for a solution of **3i** (6.5 mg, 30 µmol) in DMSO-d_6 (0.50 mL, 60 mM) at 25 °C. The solution was allowed to equilibrate in the NMR probe for 10 min before the corresponding ^{1}H NMR spectrum was recorded (400 MHz, 25 °C, 16 transients). Equilibrium was attained rapidly, and virtually the same **3i/CH$_2$O** ratio was found even after several days.

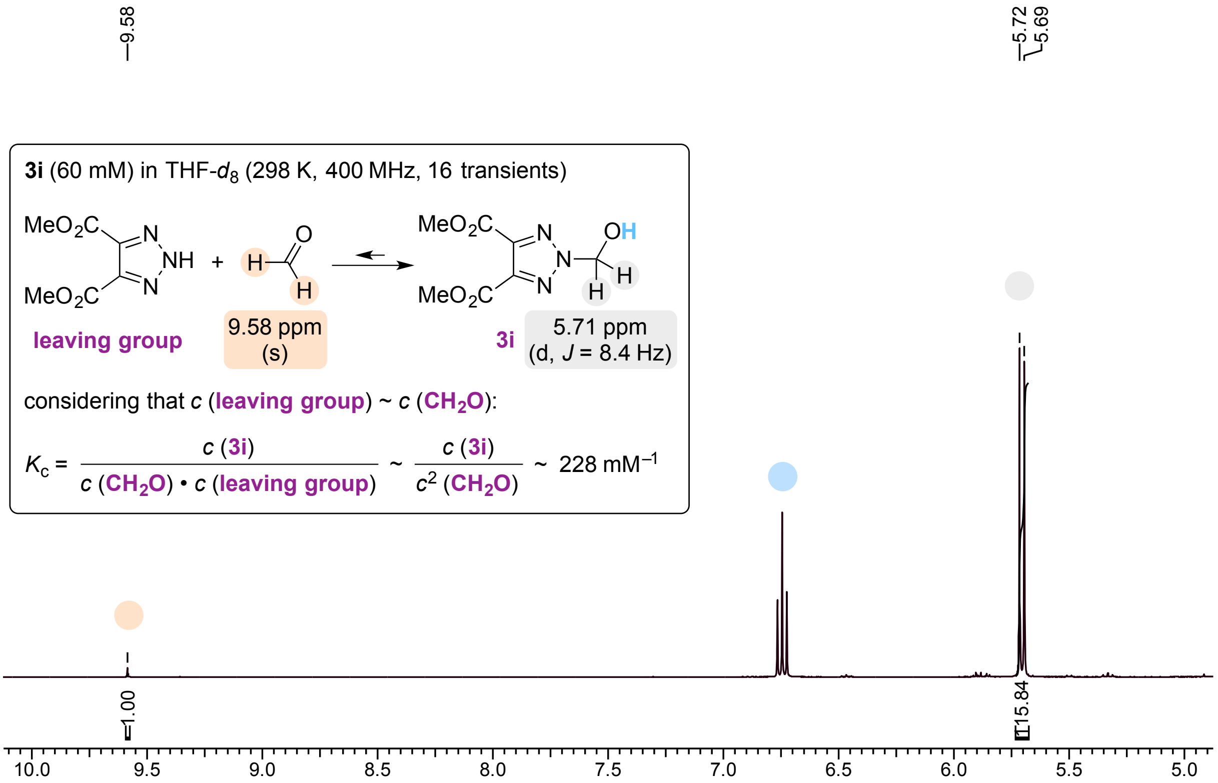

Figure S8. An inset of the ^{1}H NMR spectrum of **3i** in a range of 10.00–5.00 ppm showing equilibrium between **3i** and formaldehyde for a solution of **3i** (6.5 mg, 30 µmol) in THF-d_8 (0.50 mL, 60 mM) at 25 °C. The solution was allowed to equilibrate in the NMR probe for 10 min before the corresponding ^{1}H NMR spectrum was recorded (400 MHz, 25 °C, 16 transients). Equilibrium was attained rapidly, and virtually the same **3i**/**CH_2O** ratio was found even after several days.

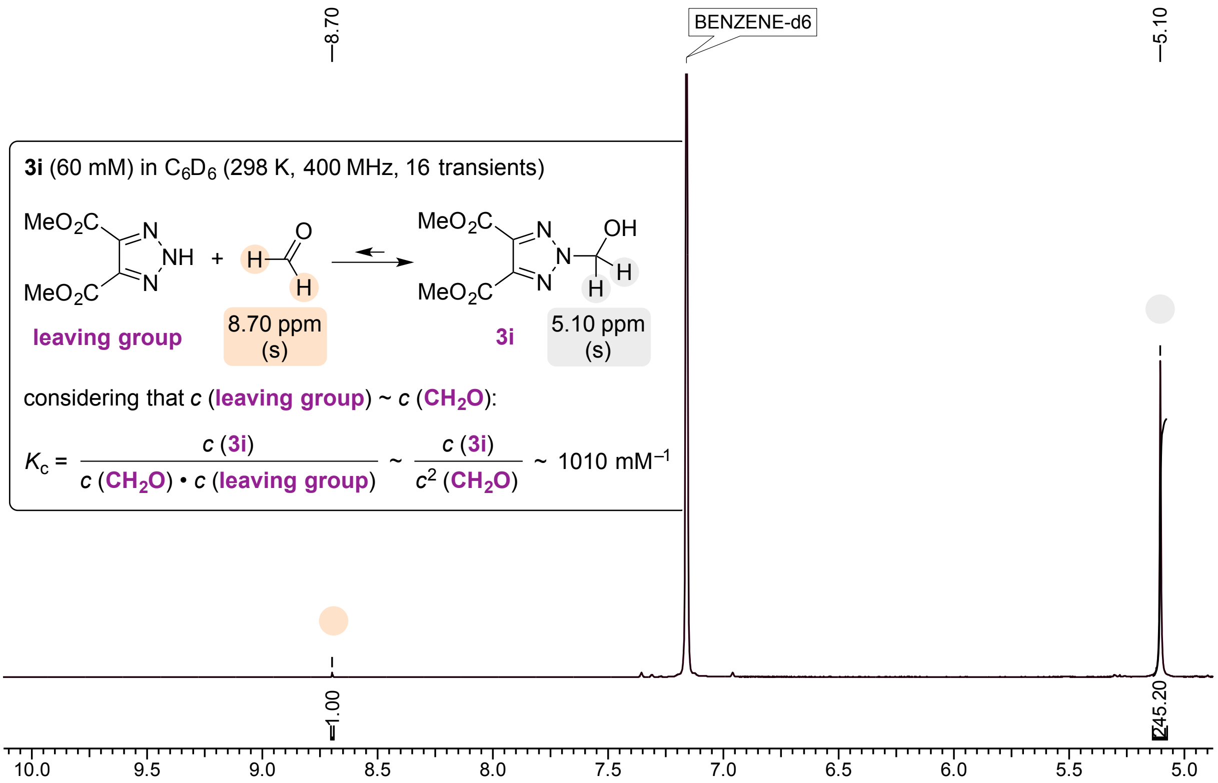

Figure S9. An inset of the ^{1}H NMR spectrum of **3i** in a range of 10.00–5.00 ppm showing equilibrium between **3i** and formaldehyde for a solution of **3i** (6.5 mg, 30 µmol) in C_6D_6 (0.50 mL, 60 mM) at 25 °C. The solution was allowed to equilibrate in the NMR probe for 10 min before the corresponding ^{1}H NMR spectrum was recorded (400 MHz, 25 °C, 16 transients). Equilibrium was attained rapidly, and virtually the same **3i/CH_2O** ratio was found even after several days.

2.8 Formaldehyde Trapping

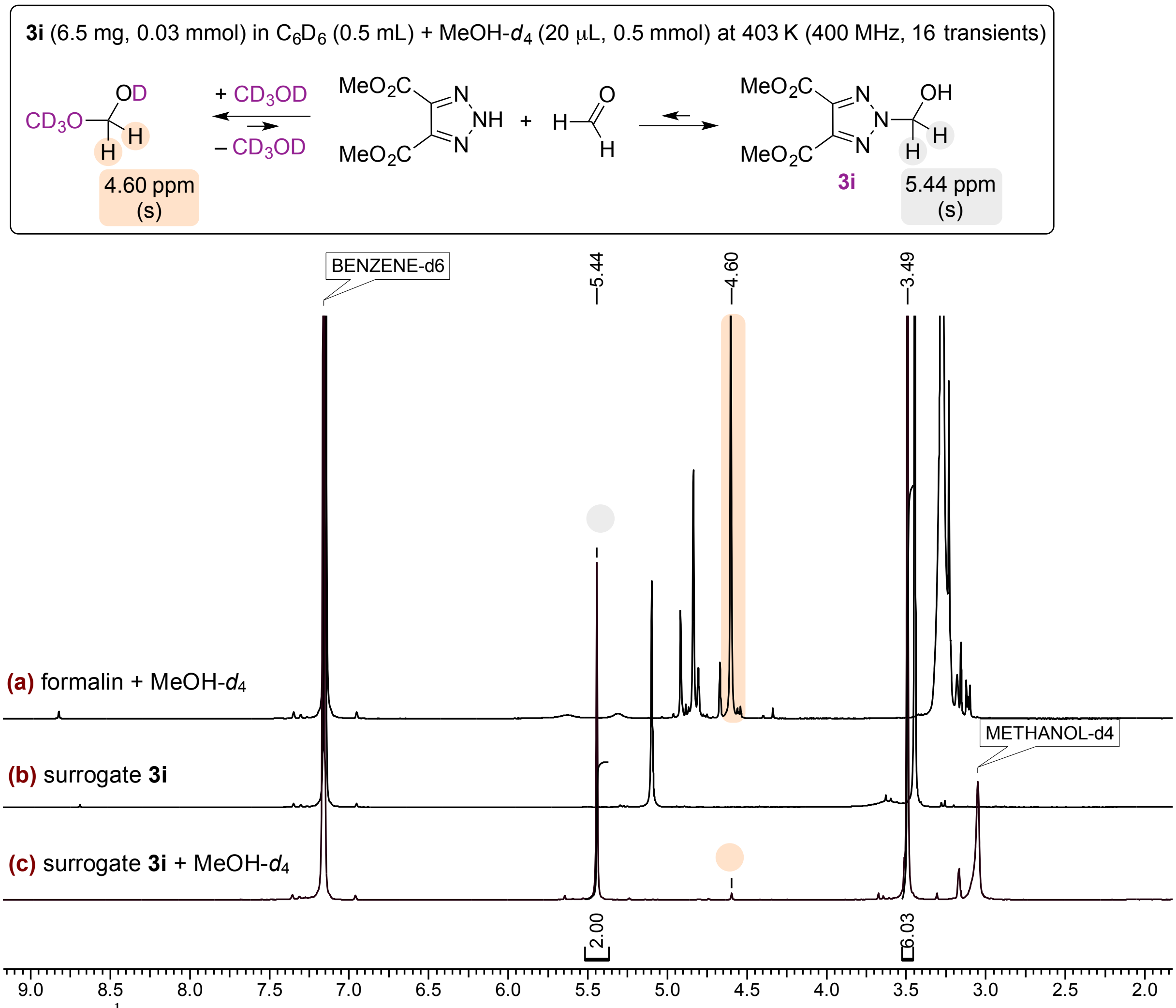

Figure S10. ^{1}H NMR experiments showing trapping of formaldehyde as methoxymethanol-d_4 based on a literature procedure[13] (insets of NMR spectra are in a range of 9.00–2.00 ppm). **(a)** A standard for the respective ^{1}H NMR peak of methoxymethanol-d_4 (4.60 ppm, s) prepared by the addition of an excess of MeOH-d_4 (20 µL, 500 µmol) to a 37% aqueous solution of formaldehyde (2.2 µL, 30 µmol) in C_6D_6 (0.50 mL, 60 mM) using a microsyringe. A septum-cap NMR tube containing the sample was shaken thoroughly afterward by a vortex mixer (10 s). Then, the mixture was allowed to equilibrate in the NMR probe for 10 min before the corresponding ^{1}H NMR spectrum was recorded (400 MHz, 40 °C, 16 transients). **(b)** A solution of **3i** (6.5 mg, 30 µmol) in C_6D_6 (0.50 mL, 60 mM) for comparison. **(c)** A solution of **3i** (6.5 mg, 30 µmol) in C_6D_6 (0.50 mL, 60 mM) treated with an excess of MeOH-d_4 (20 µL, 500 µmol) using a microsyringe. A septum-cap NMR tube containing the sample was shaken thoroughly afterward by a vortex mixer (10 s). Then, the mixture was allowed to equilibrate in the NMR probe for 10 min before the corresponding ^{1}H NMR spectrum was recorded (400 MHz, 40 °C, 16 transients). Equilibrium was attained rapidly, and virtually the same ratio was found even after several days.

2.9 Stability of 3i Under Basic Conditions

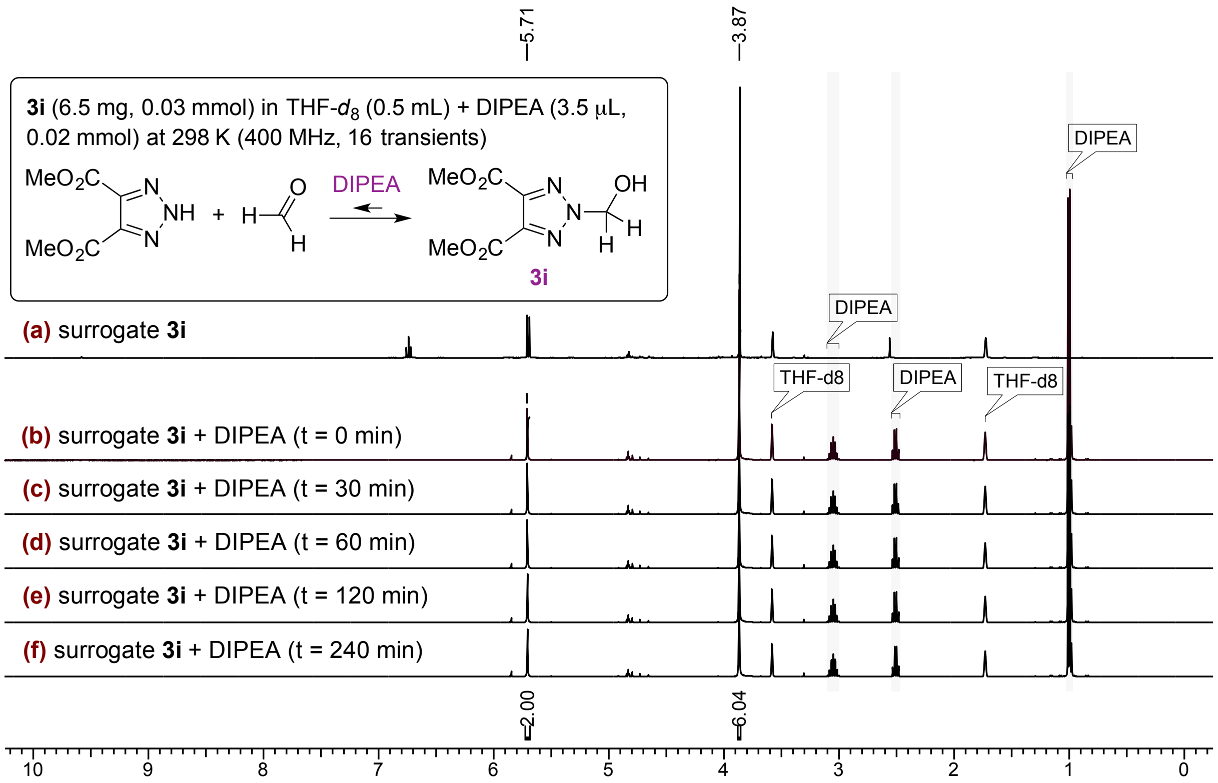

Figure S11. A time-course monitoring ^{1}H NMR experiment showing the stability of surrogate **3i** under basic conditions. **(a)** A solution of **3i** (6.5 mg, 30 μmol) in THF-d_8 (0.50 mL, 60 mM) for comparison. **(b)** A solution of **3i** (6.5 mg, 30 μmol) in THF-d_8 (0.50 mL, 60 mM) treated with *N*,*N*-diisopropylethylamine (DIPEA, 3.5 μL, 20 μmol) using a microsyringe. A septum-cap NMR tube containing the sample was shaken thoroughly afterward by a vortex mixer (10 s). Then, the mixture was allowed to equilibrate in the NMR probe for 10 min before the corresponding ^{1}H NMR spectrum was recorded (400 MHz, 25 °C, 16 transients). The above mixture was monitored over the next **(c)** 30, **(d)** 60, **(e)** 120, and **(f)** 240 min by ^{1}H NMR (400 MHz, 25 °C, 16 transients each), providing virtually identical ^{1}H NMR spectra as **(b)** with no apparent degradation of **3i**.

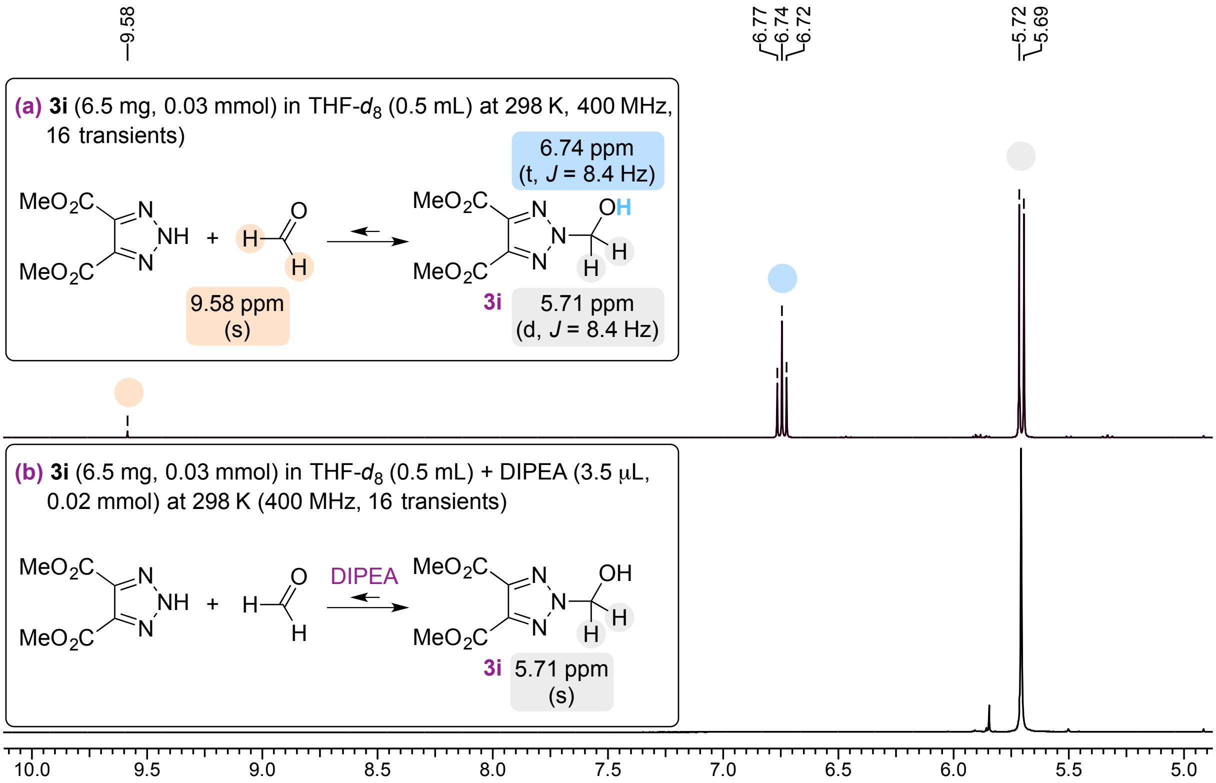

Figure S12. A detail of spectra **(a)** and **(b)** from **Fig. S11** showing a disappearance of the formaldehyde peak (9.58 ppm, s) and a loss of signal multiplicity due to the base-catalyzed chemical exchange (insets of NMR spectra are in a range of 10.00–5.00 ppm).

2.10 Determination of the Reaction Reversibility by Crossover Experiments

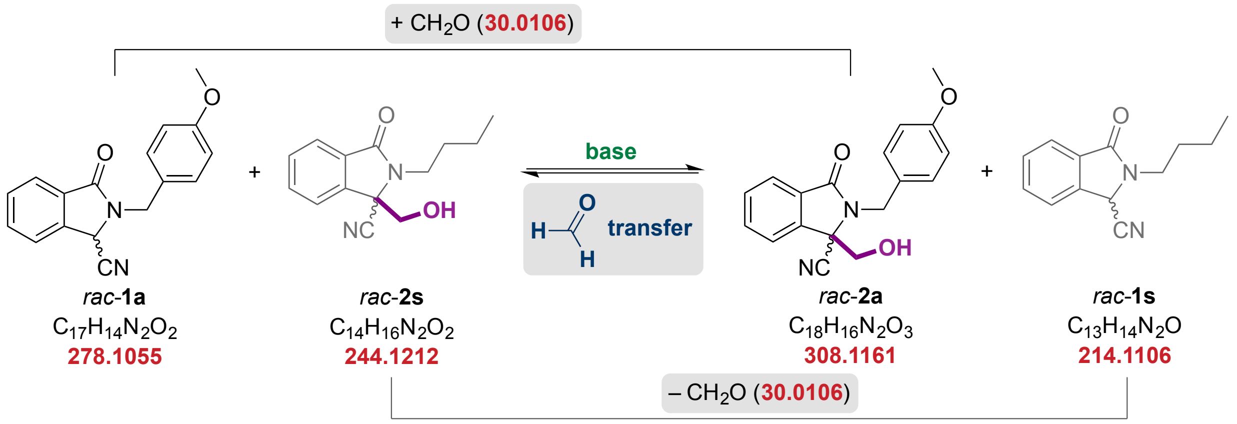

2.10.1 Crossover Experiment with K_3PO_4 (Under Conditions for the Suzuki Coupling in **Chapter 6.7**)

In a Schlenk tube, K_3PO_4 (21.2 mg, 0.1 mmol) was added to a solution of *rac*-**1a** (13.9 mg, 0.05 mmol) and *rac*-**2s** (12.3 mg, 0.05 mmol) in a mixture of 1,4-dioxane and water (4:1, 1 mL). The tube was sealed and the reaction mass was stirred and heated at 80 °C for 4 h (oil bath). Then, the mixture was diluted with water and repetitively extracted with CH_2Cl_2. The combined organic layers were washed with brine, dried over anhydrous Na_2SO_4, and evaporated *in vacuo*. The crude residue was directly analyzed by LC-HRMS (APCI+), as shown in **Figs. S13–14**.

2.10.2 Crossover Experiment with NaOH

In a 4-mL screw-cap vial, 10% aqueous NaOH (0.1 mL) was added to a solution of *rac*-**1a** (13.9 mg, 0.05 mmol) and *rac*-**2s** (12.3 mg, 0.05 mmol) in THF (1 mL). The resulting mixture was left to stir at rt for 24 h. Then, the reaction medium was acidified with 1 M HCl to pH = 3. The water phase was repetitively extracted with CH_2Cl_2. The combined organic layers were washed with brine, dried over anhydrous Na_2SO_4, and evaporated *in vacuo*. The crude residue was directly analyzed by LC-HRMS (APCI+), as shown in **Figs. S15–16**.

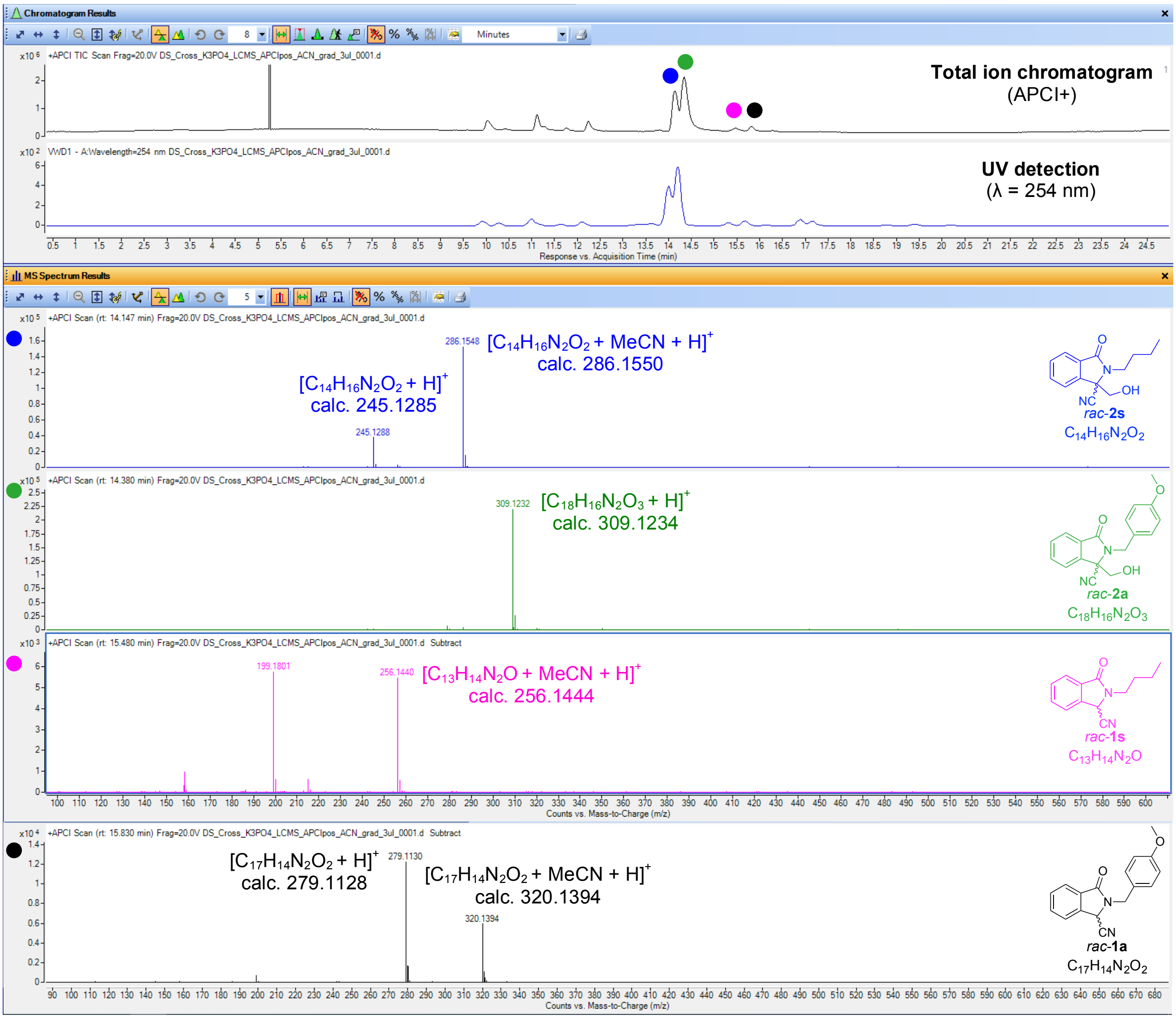

Figure S13. Total ion chromatogram (APCI+) with extracted mass spectra of the searched compounds for the crude residue of the crossover experiment with K_3PO_4 (see **2.10.1**). HPLC (a Phenomenex Gemini NX-C18 column (150 × 4.6 mm, 3 µm), mobile phase gradient: 0 min: TFA (0.1%)–MeCN = 90:10; 0–15 min: TFA (0.1%)–MeCN = 90:10 → 10:90; 15–18 min: TFA (0.1%)–MeCN = 10:90 → 90:10; 18–25 min: TFA (0.1%)–MeCN = 90:10, flow rate = 0.5 mL/min, λ = 254 nm, injection 3 µL).

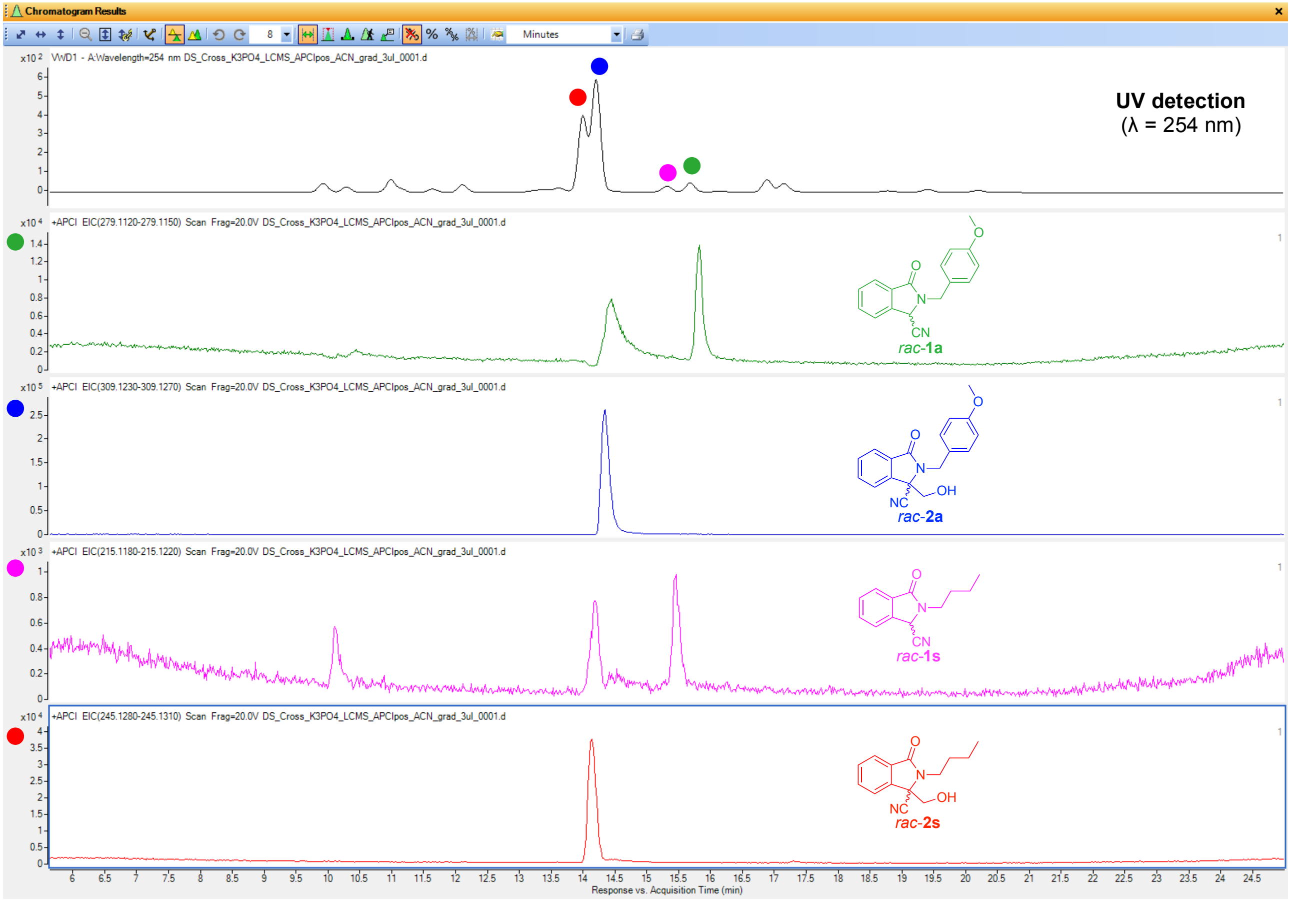

Figure S14. Extracted ion chromatograms of the searched compounds for the crude residue of the crossover experiment with K_3PO_4 (see **2.10.1**). HPLC (a Phenomenex Gemini NX-C18 column (150 × 4.6 mm, 3 μm), mobile phase gradient: 0 min: TFA (0.1%)–MeCN = 90:10; 0–15 min: TFA (0.1%)–MeCN = 90:10 → 10:90; 15–18 min: TFA (0.1%)–MeCN = 10:90 → 90:10; 18–25 min: TFA (0.1%)–MeCN = 90:10, flow rate = 0.5 mL/min, λ = 254 nm, injection 3 μL).

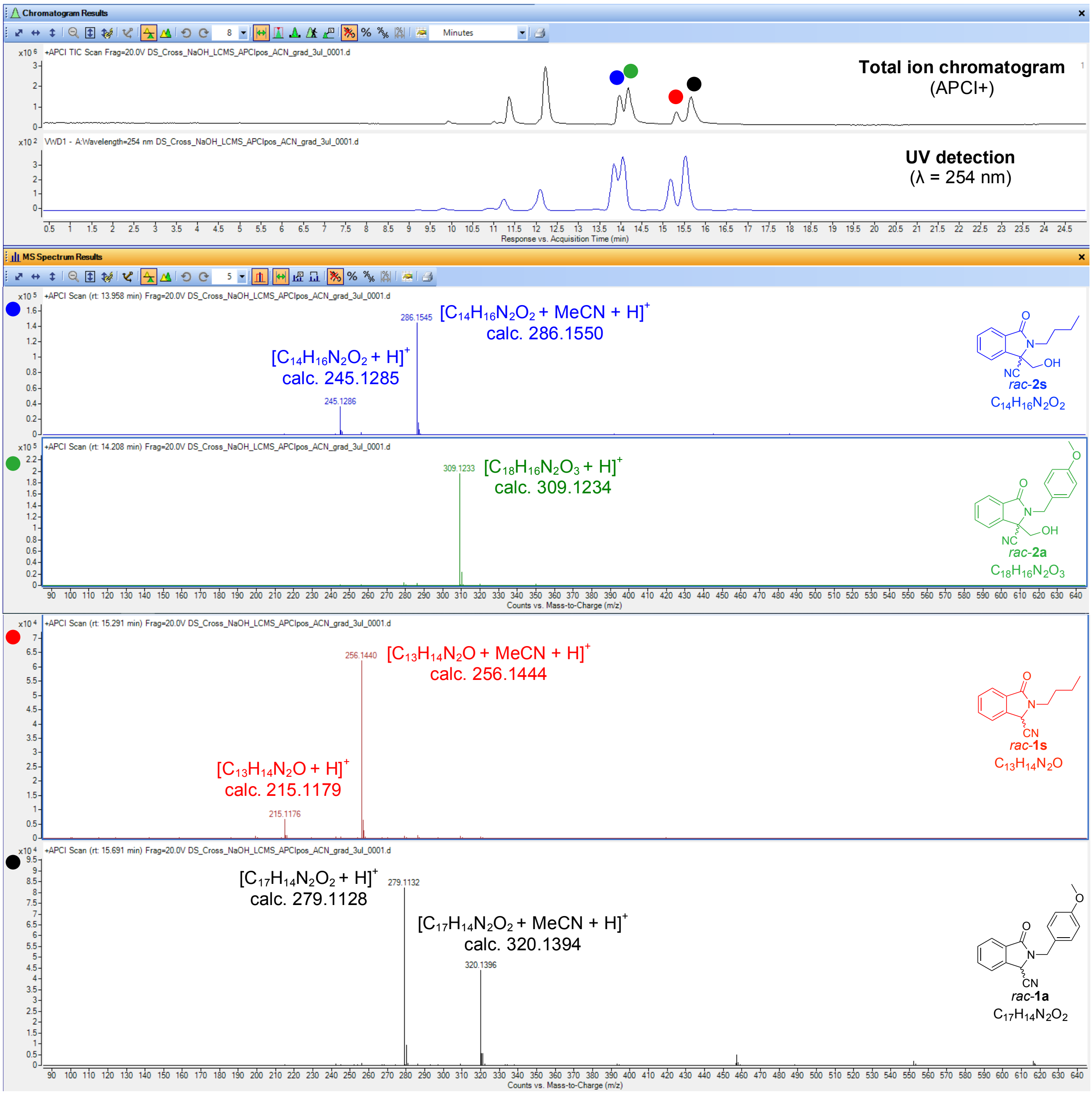

Figure S15. Total ion chromatogram (APCI+) with extracted mass spectra of the searched compounds for the crude residue of the crossover experiment with NaOH (see **2.10.2**). HPLC (a Phenomenex Gemini NX-C18 column (150 × 4.6 mm, 3 µm), mobile phase gradient: 0 min: TFA (0.1%)–MeCN = 90:10; 0–15 min: TFA (0.1%)–MeCN = 90:10 → 10:90; 15–18 min: TFA (0.1%)–MeCN = 10:90 → 90:10; 18–25 min: TFA (0.1%)–MeCN = 90:10, flow rate = 0.5 mL/min, λ = 254 nm, injection 3 µL).

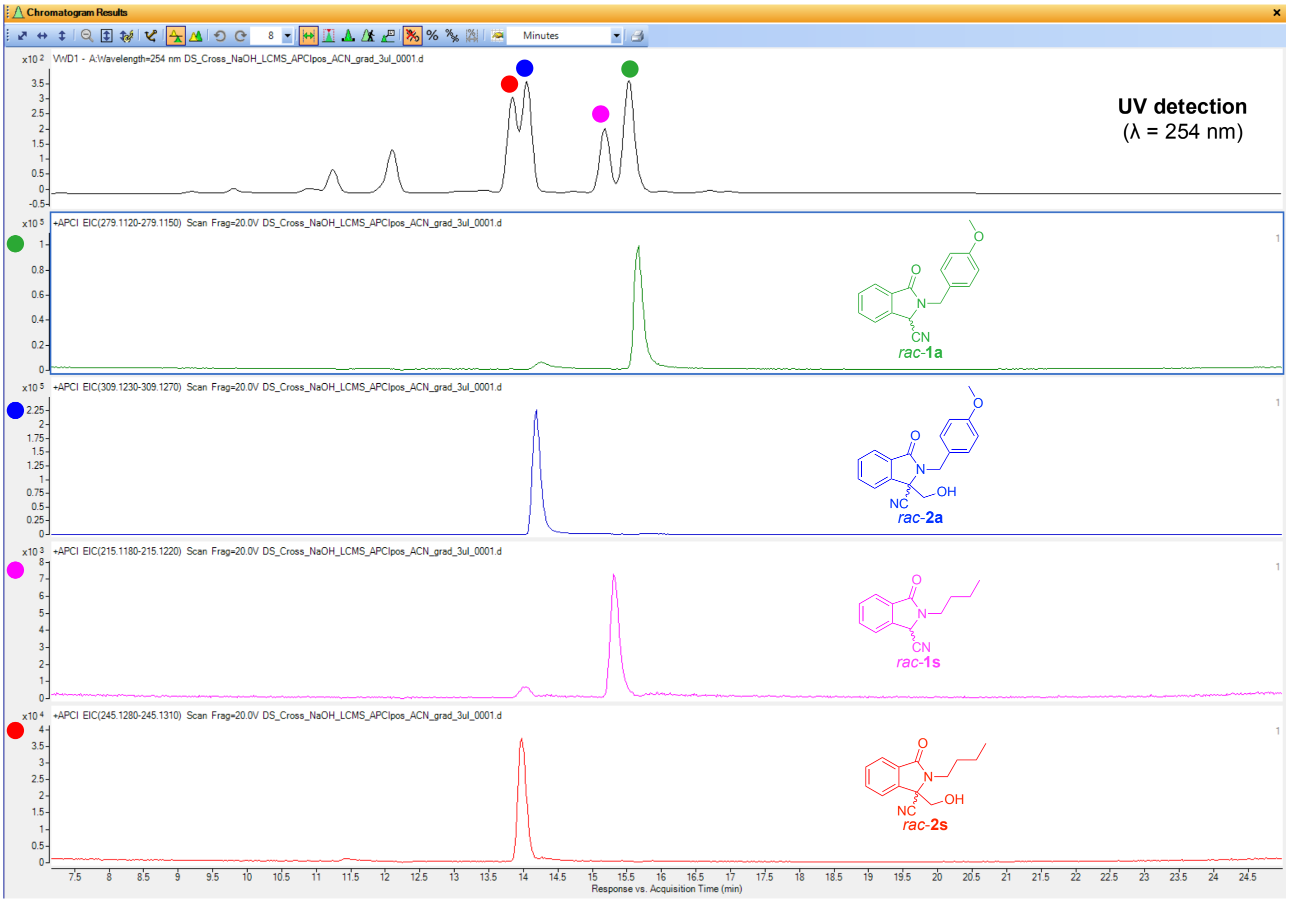

Figure S16. Extracted ion chromatograms of the searched compounds for the crude residue of the crossover experiment with NaOH (see **2.10.2**). HPLC (a Phenomenex Gemini NX-C18 column (150 × 4.6 mm, 3 µm), mobile phase gradient: 0 min: TFA (0.1%)–MeCN = 90:10; 0–15 min: TFA (0.1%)–MeCN = 90:10 → 10:90; 15–18 min: TFA (0.1%)–MeCN = 10:90 → 90:10; 18–25 min: TFA (0.1%)–MeCN = 90:10, flow rate = 0.5 mL/min, λ = 254 nm, injection 3 µL).

3. Miscellaneous Syntheses

3.1 Syntheses of Catalysts

Analytical data for the prepared compounds are listed below:

Compound **A26** **(1*R*,2*R*)-*N*[1]-(1*H*-benzo[*d*]imidazol-2-yl)-*N*[2],*N*[2]-dimethylcyclohexane-1,2-diamine**:[14]

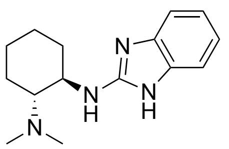

A26

Compound **A26** was prepared according to the literature procedure.[14] Yellowish powder. $[\alpha]^{25}_D$ −41 (*c* 1.0, MeOH) (lit.[14] $[\alpha]^{20}_D$ −55.0 (*c* 1.0, MeOH)). ^{1}H NMR (300 MHz, $CDCl_3$) δ/ppm: 8.39 (br s, 1H), 7.29–7.26 (m, 2H), 7.04–7.00 (m, 2H), 5.52 (br s, 1H), 3.50–3.42 (m, 1H), 2.68–2.64 (m, 1H), 2.31–2.25 (m, 1H), 2.18 (s, 6H), 1.84–1.80 (m, 2H), 1.64–1.61 (m, 1H), 1.28–1.09 (m, 4H). ^{13}C NMR (75 MHz, $CDCl_3$) δ/ppm: 155.8, 138.2, 120.2, 112.3, 67.2, 54.0, 39.9, 33.2, 25.2, 24.3, 21.2. HRMS (ESI-Orbitrap) *m/z*: $[M + H]^+$ calcd for $C_{15}H_{23}N_4$ 259.1917; found 259.1917.

Compound **A27** **(1*R*,2*R*)-*N*[1]-(5,7-dinitrobenzo[*d*]thiazol-2-yl)-*N*[2],*N*[2]-dimethylcyclohexane-1,2-diamine**:

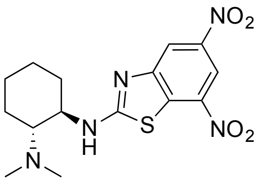

A27

A solution of 3,5-dinitrophenyl isothiocyanate (290 mg, 1.3 mmol) in dry CH_2Cl_2 (2 ml) was added dropwise to the ice cold mixture of (1*R*,2*R*)-*N*[1],*N*[1]-dimethylcyclohexane-1,2-diaminium trifluoroacetate (480 mg, 1.3 mmol) and Et_3N (720 µL, 5.2 mmol) in dry CH_2Cl_2 (8 mL). The reaction mass was stirred at rt overnight under Ar. After that, it was diluted with CH_2Cl_2 and washed with a saturated aqueous solution of $NaHCO_3$, brine, dried over anhydrous Na_2SO_4, filtered, and evaporated. The residue was subjected to column chromatography (SiO_2, Et_2O–MeOH = 95:5). Yellow powder, yield 105 mg (22%). M.p. 162–165 °C. $[\alpha]^{25}_D$ +8 (*c* 0.25, MeOH). ^{1}H NMR (400 MHz, $CDCl_3$) δ/ppm: 8.80 (d, *J* = 2.0 Hz, 1H), 8.51 (d, *J* = 2.0 Hz, 1H), 3.58–3.52 (m, 1H), 2.82–2.78 (m, 1H), 2.50–2.44 (m, 1H), 2.29 (s, 6H), 1.97–1.91 (m, 2H), 1.84–1.80 (m, 1H), 1.50–1.42 (m, 1H), 1.32–1.20 (m, 4H). ^{13}C NMR (100 MHz, $CDCl_3$) δ/ppm: 170.7, 155.8, 146.2, 141.1, 133.7, 117.4, 111.6, 66.7, 56.1, 39.9, 31.9, 25.2, 24.4, 21.1. HRMS (APCI) *m/z*: $[M + H]^+$ calcd for $C_{15}H_{20}N_5O_4S$ 366.1231, found 366.1233.

Compound **A28** ***N*-(((3,5-bis(trifluoromethyl)phenyl)amino)(((1*R*,2*R*)-2-(dimethylamino)cyclohexyl)-amino)methylene)-4-methylbenzenesulfonamide**:[15]

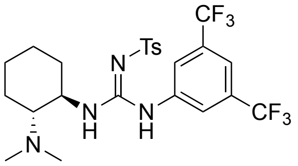

A28

Compound **A28** was prepared according to the literature procedure.[15] Colorless glassy solid. M.p. 52–55 °C. $[\alpha]^{25}_D$ −9 (*c* 1.0, MeOH). ^{1}H NMR (400 MHz, $CDCl_3$) δ/ppm: 12.72 (br s, 1H), 7.83–7.80 (m, 2H), 7.51 (br s, 1H), 7.49 (br s, 2H), 7.30–7.28 (m, 2H), 3.41–3.36 (m, 1H), 2.42 (s, 6H), 2.39 (s, 3H), 2.26–2.21 (m, 1H), 1.97–1.94 (m, 1H), 1.86–1.79 (m, 2H), 1.41 (br s, 1H), 1.31–1.16 (m,

4H). ^{13}C NMR (100 MHz, $CDCl_3$) δ/ppm: 142.3, 140.5, 129.4, 125.9, 123.1 (q, $^{1}J_{CF}$ = 272.9 Hz), 68.9, 40.9, 33.4, 24.5, 24.3, 21.4. ^{19}F NMR (376 MHz, $CDCl_3$) δ/ppm: −62.96 (s, 6F). HRMS (ESI-Orbitrap) *m/z*: $[M + H]^+$ calcd for $C_{24}H_{29}F_6N_4O_2S$ 551.1910, found 551.1916.

Compound **A29** ***N*-(((1*R*,2*R*)-2-(dimethylamino)cyclohexyl)carbamoyl)-3,5-bis(trifluoromethyl)-benzamide**: Oxalyl chloride (115 μL, 1.33 mmol) was added to a solution of 3,5-bis(trifluoromethyl)benzamide (200 mg, 0.78 mmol) in dry 1,2-dichloroethane (5 mL). The mixture was refluxed overnight under Ar (oil bath). Then, the volatiles were removed *in vacuo*, and the resulting semisolid residue was dissolved in dry THF (2 mL). Dry Et_3N (650 μL, 4.7 mmol) and (1*R*,2*R*)-N^1,N^1-dimethylcyclohexane-1,2-diaminium trifluoroacetate (290 mg, 0.78 mmol) were added thereto, and the solution was stirred at ambient temperature overnight under Ar. Thereafter, the mixture was diluted with CH_2Cl_2, washed with a saturated aqueous solution of $NaHCO_3$, brine, dried over anhydrous Na_2SO_4, filtered, and evaporated *in vacuo*. The crude product was subjected to column chromatography (SiO_2, CH_2Cl_2–MeOH = 99:1 → 93:7). White solid, yield 131 mg (39% over two steps). M.p. 133–135 °C. $[\alpha]^{25}_{D}$ −18 (*c* 0.5, MeOH). ^{1}H NMR (300 MHz, $CDCl_3$) δ/ppm: 11.00 (br s, 1H), 8.98 (d, *J* = 6.3 Hz, 1H), 8.60 (s, 2H), 8.08 (s, 1H), 3.68–3.60 (m, 1H), 2.42–2.32 (m, 1H), 2.25 (s, 6H), 1.90–1.82 (m, 2H), 1.68–1.61 (m, 1H), 1.26–1.19 (m, 5H). ^{13}C NMR (75 MHz, $CDCl_3$) δ/ppm: 165.8, 154.2, 135.1, 132.1 (q $^{2}J_{CF}$ = 34.3 Hz), 128.7, 125.9, 123.0 (q $^{1}J_{CF}$ = 272.9 Hz), 66.7, 51.2, 40.1, 33.0, 25.2, 24.5, 21.9. ^{19}F NMR (282 MHz, $CDCl_3$) δ/ppm: −62.66 (s, 6F). HRMS (APCI) *m/z*: $[M + H]^+$ calcd for $C_{18}H_{22}F_6N_3O_2$ 426.1611, found 426.1614.

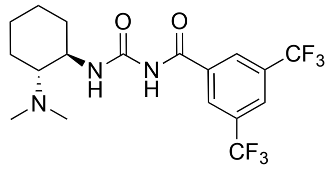

A29

Compound **A30** ***N*-((1*R*,2*R*)-2-(dimethylamino)cyclohexyl)-3,5-bis(trifluoromethyl)benzene-sulfonamide**:[16] The mixture of (1*R*,2*R*)-N^1,N^1-dimethylcyclohexane-1,2-diaminium trifluoroacetate (260 mg, 0.7 mmol), 3,5-bis(trifluoromethyl)benzenesulfonyl chloride (242 mg, 0.77 mmol) and Et_3N (585 μL, 4.2 mmol) in dry CH_2Cl_2 (5 mL) was stirred at rt overnight under Ar. The solution was diluted with CH_2Cl_2, washed with a saturated aqueous solution of $NaHCO_3$, brine, dried over anhydrous Na_2SO_4, filtered, and evaporated. The residue was subjected to column chromatography (SiO_2, CH_2Cl_2–MeOH = 99:1 → 93:7). White solid, yield 240 mg (82%). M.p. 57–58 °C, (lit.[16] m.p. 80–81 °C). $[\alpha]^{25}_{D}$ −64 (*c* 1.25, $CHCl_3$), (lit.[16] $[\alpha]^{22}_{D}$ −65.9 (*c* 1.28, $CHCl_3$)). ^{1}H NMR (300 MHz, $CDCl_3$) δ/ppm: 8.35 (s, 2H), 8.06 (s, 1H), 2.81–2.72 (m, 1H), 2.30–2.16 (m, 2H), 2.01 (s, 6H), 1.82–1.77 (m, 2H), 1.69–1.66 (m, 1H), 1.24–1.02 (m, 4H). ^{13}C NMR (75 MHz, $CDCl_3$) δ/ppm: 143.6, 132.7 (q $^{2}J_{CF}$ = 34.5 Hz), 127.5, 125.8, 122.5 (q $^{1}J_{CF}$ = 273.3 Hz), 66.3, 54.4, 39.6, 32.6, 24.9, 24.1, 21.1. ^{19}F NMR (282 MHz, $CDCl_3$) δ/ppm: −62.94 (s, 6F). HRMS (ESI-Orbitrap) *m/z*: $[M + H]^+$ calcd for $C_{16}H_{21}F_6N_2O_2S$ 419.1222, found 419.1223.

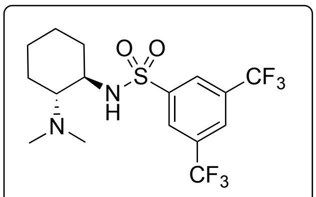

A30

Compound **A31** ***N*-((1*R*,2*R*)-2-(dimethylamino)cyclohexyl)-3,5-bis(trifluoromethyl)benzamide**: A

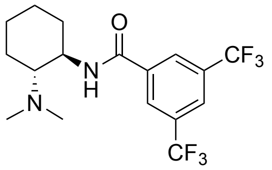

A31

solution of 3,5-bis(trifluoromethyl)benzoic acid (100 mg, 0.39 mmol), (1*R*,2*R*)-N^1,N^1-dimethylcyclohexane-1,2-diamine (55 mg, 0.39 mmol), EDCl (90 mg, 0.47 mmol), and HOBt•H_2O (63 mg) in anhydrous THF (1 mL) was stirred at rt overnight. The mixture was diluted with water and repetitively extracted with EtOAc. The combined organic layers were washed with a saturated aqueous solution of $NaHCO_3$, brine, dried over anhydrous Na_2SO_4, filtered, and evaporated. The residue was subjected to column chromatography (SiO_2, CH_2Cl_2–MeOH = 97:3). White solid, yield 97 mg (65%). M.p. 133–135 °C. $[\alpha]^{25}_D$ −16 (*c* 0.5, MeOH). ^{1}H NMR (400 MHz, $CDCl_3$) δ/ppm: 8.25 (s, 2H), 7.98 (s, 1H), 7.25 (br s, 1H), 3.72–3.64 (m, 1H), 2.66–2.63 (m, 1H), 2.57–2.52 (m, 1H), 2.29 (s, 6H), 1.95–1.89 (m, 2H), 1.78–1.75 (m, 1H), 1.43–1.16 (m, 4H). ^{13}C NMR (100 MHz, $CDCl_3$) δ/ppm: 165.0, 137.2, 132.4 (q, $^2J_{CF}$ = 33.7 Hz), 127.5, 124.7, 123.0 (q, $^1J_{CF}$ = 272.9 Hz), 66.3, 52.0, 39.8, 32.3, 25.2, 24.6, 21.2. ^{19}F NMR (376 MHz, $CDCl_3$) δ/ppm: −62.77 (s, 6F). HRMS (ESI-Orbitrap) *m/z*: $[M + H]^+$ calcd for $C_{17}H_{21}F_6N_2O$ 383.1553, found 383.1554.

Compound **A33** **1,3-bis((1*R*,2*R*)-2-(dimethylamino)cyclohexyl)thiourea**: Compound **A33** was prepared

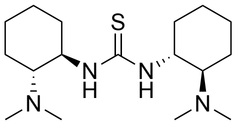

A33

according to the literature procedure.[9] Yellowish glassy solid. ^{1}H NMR (300 MHz, $CDCl_3$) δ/ppm: 7.09 (br s, 2H), 3.67–3.60 (m, 2H), 2.48–2.33 (m, 4H), 2.24 (s, 12H), 1.88–1.78 (m, 4H), 1.71–1.66 (m, 2H), 1.31–1.07 (m, 8H). ^{13}C NMR (75 MHz, $CDCl_3$) δ/ppm: 183.1, 66.9, 56.1, 40.1, 33.1, 25.1, 24.7, 21.9. HRMS (ESI-Orbitrap) *m/z*: $[M + H]^+$ calcd for $C_{17}H_{35}N_4S$ 327.2577, found 327.2589.

Synthesis of **A38 1-(3,5-bis(trifluoromethyl)phenyl)-3-((1*S*,2*S*)-2-(dimethylamino)cyclopropyl)thiourea**:

Step 1: Compound **I di-*tert*-butyl (1S,2S)-cyclopropane-1,2-diyldicarbamate**:[17] (1*S*,2*S*)-Cyclopropane-

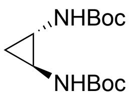

I

1,2-dicarboxylic acid[18] (1.0 g, 7.5 mmol) with a catalytic amount of DMF (few drops) were suspended in dry PhH (20 mL), $SOCl_2$ (70 mmol, 5.0 mL) was added thereto and the resulting mixture was refluxed overnight under Ar (oil bath). The volatiles were removed under reduced pressure, and the crude dicarboxylic acid dichloride was immediately dissolved in dry MePh (10 mL). The solution thereof was added dropwise at 70 °C to a vigorously stirred suspension of finely powdered NaN_3 (77 mmol, 5.0 g) in dry MePh (10 mL). After the complete addition, the reaction mixture was left to stir at 80 °C for 4 h (oil bath). Then dry *t*-BuOH (5 mL) was added at once, and the resulting reaction mass was allowed to stir at 80 °C overnight (oil bath). Next, a white precipitate was filtered off, and washed with several portions of fresh MePh and the filtrate was concentrated *in vacuo*. Traces of *t*-BuOH were removed azeotropically with *n*-heptane. The crude yellow product was purified by a silica plug (SiO_2, *n*-hexane–EtOAc = 3:2) to afford **I**, which was used directly in the following step.

Yellowish powder, yield 680 mg (33%). M.p. 130–131 °C (lit.[17] m. p. 131–132 °C). $[\alpha]^{32}_{D}$ +51 (*c* 1.0, $CHCl_3$).

Step 2: Compound **II (1*S*,2*S*)-2-((*tert*-butoxycarbonyl)amino)cyclopropanaminium hydrochloride**:[19] Di-*tert*-butyl (1*S*,2*S*)-cyclopropane-1,2-diyldicarbamate (500 mg, 1.8 mmol) was dissolved in dry MeOH (10 mL), acetyl chloride (155 µL, 2.16 mmol) was added dropwise at 0 °C. The mixture was left to stir at rt overnight under Ar. The volatiles were evaporated *in vacuo*. The residue was triturated with Et_2O, filtered, washed with fresh Et_2O, and then dried. White powder, yield 244 mg (65%). ^{1}H NMR (400 MHz, DMSO-d_6) δ/ppm: 8.54 (br s, 3H), 7.18 (br s, 1H), 2.90 (br s, 1H), 2.48 (br s, 1H), 1.38 (s, 9H), 1.11–1.06 (m, 1H), 0.92–0.87 (m, 1H).

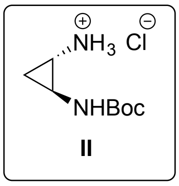

Step 3: Compound **III *tert*-butyl ((1*S*,2*S*)-2-(dimethylamino)cyclopropyl)carbamate**: Compound **II** (150 mg, 0.69 mmol) was dissolved in MeOH (2 mL). Aqueous formaldehyde (37%, 260 µL, 3.45 mmol) and subsequently $NaBH_3CN$ (130 mg, 2.1 mmol) were added thereto. The mixture was stirred at rt overnight. Then, it was diluted with water and repetitively extracted with CH_2Cl_2, the combined organic phases were washed with brine, dried over anhydrous Na_2SO_4, filtered, and evaporated. The product was used directly in the next step without further purification. Off-white solid, yield 130 mg (94%). ^{1}H NMR (400 MHz, $CDCl_3$) δ/ppm: 4.62 (br s, 1H), 2.51–2.47 (m, 1H), 2.37 (s, 6H), 1.64-1.61 (m, 1H), 1.44 (s, 9H), 0.85–0.81 (m, 1H), 0.67–0.62 (m, 1H).

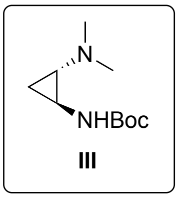

Step 4: Compound **IV (1*S*,2*S*)-*N*[1],*N*[1]-dimethylcyclopropane-1,2-diaminium chloride**: Compound **III** (100 mg, 0.5 mmol) was dissolved in anhydrous MeOH (1 mL). A saturated solution of HCl in MeOH (1 mL) was added, and the mixture was stirred at rt for 2 h. Then, the volatiles were evaporated, and the residue was triturated with Et_2O, filtered, washed with fresh Et_2O, and dried. White powder, yield 86 mg (quantitative).

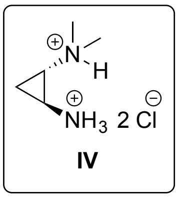

Step 5: Compound **A38 1-(3,5-bis(trifluoromethyl)phenyl)-3-((1*S*,2*S*)-2-(dimethylamino)-cyclopropyl)thiourea**: Compound **IV** (85 mg, 0.49 mmol) was suspended in dry THF (2 mL). Et_3N (290 µL, 2 mmol), and subsequently 3,5-bis(trifluoromethyl)phenyl isothiocyanate (114 µL, 0.6 mmol) were added thereto. The reaction mass was stirred at rt for 2 h under Ar. The solvent was evaporated *in vacuo*, and the crude product was purified by column chromatography (SiO_2, CH_2Cl_2–MeOH = 95:5). Yellowish solid, yield 90 mg (48%). M.p. 133–135 °C. $[\alpha]^{25}_{D}$ +14 (*c* 0.5, THF). ^{1}H NMR (400 MHz, $CDCl_3$) δ/ppm: 8.04 (br s, 3H), 7.72 (s, 1H), 7.10 (br s, 1H), 2.70 (br s, 1H), 2.40 (s, 6H), 2.01 (br s, 1H), 1.16–1.12 (m, 1H), 0.98 (br s, 1H). ^{13}C NMR (100 MHz, $CDCl_3$) δ/ppm: 181.6, 139.6. 132.1 (q, $^2J_{CF}$ = 33.7 Hz), 124.7, 122.9 (q, $^1J_{CF}$ = 272.6 Hz), 119.5, 47.5, 44.6. ^{19}F NMR (376 MHz, $CDCl_3$) δ/ppm: −62.86 (s, 6F). HRMS (APCI) *m/z*: $[M + H]^+$ calcd for $C_{14}H_{16}F_6N_3S$ 372.0964, found 372.0965.

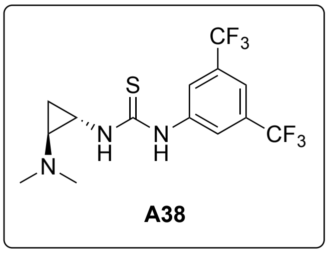

Synthesis of compound **A39 (3a*R*,6a*S*)-*N*-(3,5-bis(trifluoromethyl)phenyl)-3-methylhexahydro-cyclopenta[*d*]imidazole-1(2*H*)-carbothioamide**:

Step 1: Compound **V (3a*S*,6a*R*)-1-methyloctahydrocyclopenta[*d*]imidazole**: *tert*-Butyl (1*S*,2*R*)-2-aminocyclopentylcarbamate (80 mg, 0.4 mmol) was dissolved in MeOH (5 mL). Aqueous formaldehyde (37%, 150 µL, 2.0 mmol) and subsequently $NaBH_3CN$ (50 mg, 0.8 mmol) were added thereto. The mixture was stirred at rt overnight. Then, it was diluted with water and repetitively extracted with CH_2Cl_2. The combined organic phases were washed with brine, dried over anhydrous Na_2SO_4, filtered, and evaporated. The product was used directly in the next step without further purification. It was dissolved in anhydrous CH_2Cl_2 (1 mL) and trifluoroacetic acid (612 µL, 8 mmol) was added. The mixture was stirred at rt overnight. Then, it was diluted with CH_2Cl_2 and neutralized with a saturated aqueous solution of $NaHCO_3$. The aqueous layer was repetitively extracted with CH_2Cl_2. The combined organic extracts were washed with a saturated aqueous solution of $NaHCO_3$, brine, dried over anhydrous Na_2SO_4, filtered, and evaporated. A crude product obtained as a brown oil, yield 11 mg (22%), was carried forward without purification.

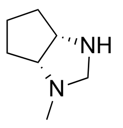

V

Step 2: Compound **A39 (3a*R*,6a*S*)-*N*-(3,5-bis(trifluoromethyl)phenyl)-3-methylhexahydro-cyclopenta[*d*]imidazole-1(2*H*)-carbothioamide**: Compound **V** 11 mg (87 µmol) was dissolved in anhydrous CH_2Cl_2 (3 mL), and 3,5-bis(trifluoromethyl)phenyl isothiocyanate (16 µL, 90 µmol) was added thereto. The reaction mass was stirred at rt overnight under Ar. The solvent was evaporated *in vacuo* and the crude product was purified by column chromatography (SiO_2, CH_2Cl_2–MeOH = 97:3). Off-white solid, yield 20 mg (13% over 3 steps). M.p. 164–166 °C. $[\alpha]^{25}_D$ 0 (*c* 0.5, $CHCl_3$). ^1H NMR (300 MHz, $CDCl_3$) δ/ppm: 7.97 (s, 2H), 7.68 (s, 1H), 7.06 (br s, 1H), 4.86 (d, *J* = 7.5 Hz, 1H), 4.36 (br s, 1H), 4.24 (d, *J* = 7.5 Hz, 1H), 3.39–3.37 (m, 1H), 2.40 (s, 3H), 2.10–1.96 (m, 3H), 1.79–1.64 (m, 3H). ^{13}C NMR (75 MHz, $CDCl_3$) δ/ppm: 176.2, 140.4, 131.8 (q, $^2J_{CF}$ = 33.7 Hz), 124.9, 123.0 (q, $^1J_{CF}$ = 272.9 Hz), 118.8, 71.8, 38.7, 31.7, 29.5, 24.1. ^{19}F NMR (282 MHz, $CDCl_3$) δ/ppm: −62.91 (s, 6F). HRMS (ESI-Orbitrap) *m/z*: $[M + H]^+$ calcd for $C_{16}H_{18}F_6N_3S$ 398.1120, found 398.1130.

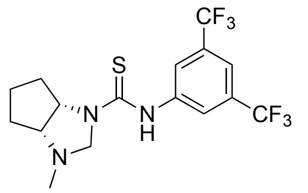

A39

Synthesis of compound **A40 1-(3,5-bis(trifluoromethyl)phenyl)-3-((1*R*,2*R*)-2-(dimethylamino)-cyclopentyl)thiourea**:

Step 1: Compound **VI di-*tert*-butyl (1*R*,2*R*)-cyclopentane-1,2-diyldicarbamate**: (1*R*,2*R*)-(–)-cyclopentane-1,2-diamine[20] (75 mg, 0.75 mmol) was dissolved in anhydrous THF (1 mL), a solution of $(Boc)_2O$ (330 mg, 1.5 mmol) in THF (1 mL) was added dropwise. The mixture was left to stir at rt overnight under Ar. After that, it was diluted with water and repetitively extracted with CH_2Cl_2. The organic phase was washed with 0.5 M HCl, a saturated aqueous solution of $NaHCO_3$, brine, dried over anhydrous Na_2SO_4, filtered, and evaporated. Off-

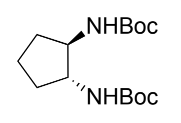

VI

white solid, yield 165 mg (73%). $[\alpha]^{25}_{D}$ +5 (*c* 1.0, $CHCl_3$). ^{1}H NMR (400 MHz, $CDCl_3$) δ/ppm: 4.88 (br s, 2H), 3.63 (br s, 2H), 2.15–2.07 (m, 2H), 1.71–1.65 (m, 2H), 1.43 (s, 18 H), 1.41–1.35 (m, 2H). ^{13}C NMR (100 MHz, $CDCl_3$) δ/ppm: 156.3, 79.3, 57.6, 29.9, 28.4, 19.5.

Step 2: Compound **A40 1-(3,5-bis(trifluoromethyl)phenyl)-3-((1*R*,2*R*)-2-(dimethylamino)-cyclopentyl)thiourea**: Prepared from compound **VI** (150 mg, 0.5 mmol) following steps 2–4 in the procedure described for **A38**. The crude product was purified by column chromatography (SiO_2, CH_2Cl_2–MeOH = 97:3). White solid, yield 60 mg (31% over 4 steps). M.p. 102–103 °C. $[\alpha]^{25}_{D}$ −36 (*c* 0.5, $CHCl_3$). ^{1}H NMR (400 MHz, $CDCl_3$) δ/ppm: 12.89 (br s, 1H), 8.08 (s, 2H), 7.60 (s, 1H), 6.64 (br s, 1H), 3.94 (br s, 1H), 3.23–3.21 (m, 1H), 2.39 (s, 6H), 2.22–2.15 (m, 1H), 1.82–1.76 (m, 3H), 1.66–1.56 (m, 2H). ^{13}C NMR (100 MHz, $CDCl_3$) δ/ppm: 182.0, 142.1, 131.6 (q, $^2J_{CF}$ = 33.4 Hz), 123.2 (q, $^1J_{CF}$ = 272.6 Hz), 122.9, 117.6, 72.5, 59.1, 40.5, 32.2, 23.7, 21.0. ^{19}F NMR (376 MHz, $CDCl_3$) δ/ppm: −62.91 (s, 6F). HRMS (ESI-Orbitrap) *m/z*: $[M + H]^+$ calcd for $C_{16}H_{19}F_6N_3S$ 400.1277, found 400.1275.

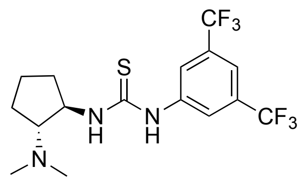

A40

Compound **A41 1-(3,5-bis(trifluoromethyl)benzyl)-3-((1*R*,2*R*)-2-(dimethylamino)cyclohexyl)thiourea**: A solution of 1,3-bis(trifluoromethyl)-5-(isothiocyanatomethyl)benzene (400 mg, 1.4 mmol) in dry CH_2Cl_2 (4 ml) was added dropwise to (1*R*,2*R*)-N^1,N^1-dimethylcyclohexane-1,2-diamine (200 mg, 1.4 mmol) in dry CH_2Cl_2 (4 mL). The reaction mass was stirred at rt overnight under Ar. After that, the solvent was evaporated, and the residue was subjected to column chromatography (SiO_2, CH_2Cl_2–MeOH = 97:3 → 95:5). Colorless glassy solid, yield 520 mg (87%). $[\alpha]^{25}_{D}$ +54 (*c* 1.0, $CHCl_3$). (lit.[21] $[\alpha]^{25}_{D}$ −46.4 (*c* 1.0, $CHCl_3$) for the opposite enantiomer). ^{1}H NMR (400 MHz, $CDCl_3$) δ/ppm: 7.79 (s, 3H), 6.42 (br s, 1H), 4.94 (dd, *J* = 15.4, 4.5 Hz, 1H), 4.77 (dd, *J* = 15.4, 4.5 Hz, 1H), 3.65 (br s, 1H), 2.56 (br s, 1H), 2.39–2.34 (m, 2H), 2.11 (s, 6H), 1.87–1.82 (m, 2H), 1.73–1.70 (m, 1H), 1.28–1.11 (m, 5H). ^{13}C NMR (100 MHz, $CDCl_3$) δ/ppm: 184.2, 141.3, 131.9 (q, $^2J_{CF}$ = 33.7 Hz), 127.7, 123.2 (q, $^1J_{CF}$ = 272.6 Hz), 121.3, 67.6, 56.3, 47.9, 40.1, 33.2, 24.7, 24.5, 22.1. ^{19}F NMR (376 MHz, $CDCl_3$) δ/ppm: −62.71 (s, 6F). HRMS (APCI) *m/z*: $[M + H]^+$ calcd for $C_{18}H_{24}F_6N_3S$ 428.1590, found 428.1594.

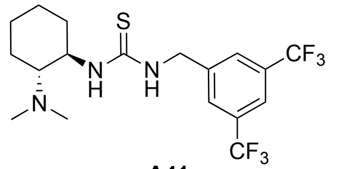

A41

Compound **A42 1-((1*R*,2*R*)-2-(dimethylamino)cyclohexyl)-3-(2,2,2-trifluoroethyl)thiourea**: Prepared according to the literature.[22] Thiophosgene (150 µL, 2.0 mmol) was added dropwise to an ice cooled solution of 2,2,2-trifluoroethylamine (150 µL, 1.9 mmol) and Et_3N (530 µL, 3.8 mmol) in dry CH_2Cl_2 (5 ml). The mixture was stirred at rt for 16 h under Ar. Thereafter, a solution of (1*R*,2*R*)-N^1,N^1-dimethylcyclohexane-1,2-diamine (270 mg, 1.9 mmol) in dry CH_2Cl_2 (2 mL) was added dropwise to the reaction mass. It was left to stir at rt for additional 4 h under Ar. The reaction was quenched with water and diluted with CH_2Cl_2.

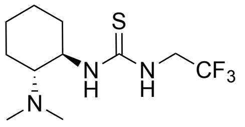

A42

Aqueous layer was repetitively extracted with CH_2Cl_2, combined organic phases were washed with saturated aqueous $NaHCO_3$ and brine, dried over anhydrous Na_2SO_4, filtered, and the solvent was evaporated. The residue was subjected to column chromatography (SiO_2, CH_2Cl_2–MeOH = 97:3 → 95:5 → 90:10). Yellowish solid, yield: 70 mg (13% over two steps). M.p. 86–89 °C. $[\alpha]^{25}_D$ +15 (*c* 0.5, $CHCl_3$). (lit.[22] $[\alpha]^{25}_D$ −64.8 (*c* 0.5, $CHCl_3$) for the opposite enantiomer). ^{1}H NMR (400 MHz, $CDCl_3$) δ/ppm: 4.38–4.25 (m, 2H), 3.61 (br s, 1H), 2.45 (br s, 1H), 2.34 (s, 6H), 2.24 (br s, 1H), 1.94–1.90 (m, 1H), 1.85–1.82 (m, 1H), 1.76–1.73 (m, 1H), 1.30–1.18 (m, 4H). ^{13}C NMR (100 MHz, $CDCl_3$) δ/ppm: 185.0, 124.4 (q, $^1J_{CF}$ = 278.4 Hz), 67.7, 56.4, 46.6 (q, $^2J_{CF}$ = 31.8 Hz), 40.3, 33.2, 24.5, 24.4, 22.5. ^{19}F NMR (376 MHz, $CDCl_3$) δ/ppm: −71.42 (t, J_{HF} = 8.7 Hz, 3F). HRMS (ESI-Orbitrap) *m/z*: $[M + H]^+$ calcd for $C_{11}H_{21}F_3N_3S$ 284.1403, found 284.1406.

Compound **A43 1-((1*R*,2*R*)-2-(dimethylamino)cyclohexyl)-3-(2-methyl-5-nitrophenyl)urea**: A solution of 2-methyl-5-nitrophenyl isocyanate (98 mg, 0.6 mmol) in dry CH_2Cl_2 (1 ml) was added dropwise to the ice cold mixture of (1*R*,2*R*)-*N*[1],*N*[1]-dimethylcyclohexane-1,2-diaminium trifluoroacetate (185 mg, 0.5 mmol) and Et_3N (280 µL, 2 mmol) in dry CH_2Cl_2 (3 mL). The reaction mass was stirred at rt overnight under Ar. After that, it was diluted with CH_2Cl_2 and washed with a saturated aqueous solution of $NaHCO_3$, brine, dried over anhydrous Na_2SO_4, filtered, and evaporated. The residue was subjected to column chromatography (SiO_2, CH_2Cl_2–MeOH = 97:3). Yellowish solid, yield 71 mg (44%). M.p. 77–80 °C. $[\alpha]^{25}_D$ −10 (*c* 0.5, MeOH). ^{1}H NMR (300 MHz, $CDCl_3$) δ/ppm: 8.64 (d, *J* = 2.4 Hz, 1H), 7.83 (dd, *J* = 8.3, 2.4 Hz, 1H), 7.28 (d, *J* = 8.3 Hz, 1H), 7.08 (br s, 1H), 5.93 (d, *J* = 3.9 Hz, 1H), 3.53–3.43 (m, 1H), 2.50–2.45 (m, 1H), 2.34 (s, 3H), 2.32–2.27 (m, 1H), 2.25 (s, 6H), 1.89–1.82 (m, 2H), 1.72–1.67 (m, 1H), 1.35–1.11 (m, 4H). ^{13}C NMR (75 MHz, $CDCl_3$) δ/ppm: 155.9, 146.9, 138.3, 135.8, 130.8, 118.1, 116.9, 66.7, 52.0, 39.9, 33.5, 25.2, 24.6, 21.3, 18.2. HRMS (ESI-Orbitrap) *m/z*: $[M + H]^+$ calcd for $C_{16}H_{25}N_4O_3$ 321.1921, found 321.1926.

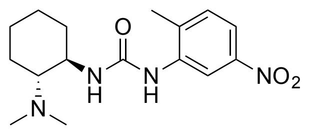

A43

Compound **A44 1-(2,4-dimethoxyphenyl)-3-((1*R*,2*R*)-2-(dimethylamino)cyclohexyl)urea**: Prepared by the addition of 2,4-dimethoxyphenyl isocyanate (99 mg, 0.6 mmol) in dry CH_2Cl_2 (1 mL) to a mixture of (1*R*,2*R*)-*N*[1],*N*[1]-dimethylcyclohexane-1,2-diaminium trifluoroacetate (185 mg, 0.5 mmol) and Et_3N (280 µL, 2 mmol) in dry CH_2Cl_2 (3 mL), according to the procedure reported for **A43**. Yellowish solid, yield 120 mg (75%). M.p. 131–134 °C. $[\alpha]^{25}_D$ −24 (*c* 1.0, $CHCl_3$). ^{1}H NMR (300 MHz, $CDCl_3$) δ/ppm: 7.85–7.82 (m, 1H), 6.73 (br s, 1H), 6.46–6.43 (m, 2H), 5.70 (d, *J* = 3.5 Hz, 1H), 3.79 (s, 3H), 3.78 (s, 3H), 3.48–3.39 (m, 1H), 2.53–2.47 (m, 1H), 2.33–2.29 (m, 1H), 2.25 (s, 6H), 1.87–1.81 (m, 2H), 1.69–1.65 (m, 1H), 1.35–1.07 (m, 4H). ^{13}C NMR (75 MHz, $CDCl_3$) δ/ppm: 156.4, 155.6, 149.7, 122.2, 120.8, 103.8, 98.7, 66.5, 55.6, 55.5, 51.8, 39.9, 33.6, 25.3, 24.6, 21.3. HRMS (ESI-Orbitrap) *m/z*: $[M + H]^+$ calcd for $C_{17}H_{28}N_3O_3$ 322.2125, found 322.2127.

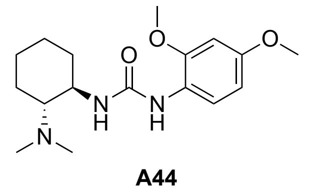

A44

Compound **A45 1-(*tert*-butyl)-3-((1*R*,2*R*)-2-(dimethylamino)cyclohexyl)urea**: Prepared by the addition of *tert*-butyl isocyanate (63 μL, 0.6 mmol) in dry CH_2Cl_2 (1 mL) to a mixture of (1*R*,2*R*)-N^1,N^1-dimethylcyclohexane-1,2-diaminium trifluoroacetate (185 mg, 0.5 mmol) and Et_3N (280 μL, 2 mmol) in dry CH_2Cl_2 (3 mL), according to the procedure reported for **A43**. Off-white solid, yield 76 mg (63%). M.p. 122–125 °C. $[\alpha]^{25}_D$ −53 (*c* 1.0, $CHCl_3$). ^1H NMR (300 MHz, $CDCl_3$) δ/ppm: 5.10 (br s, 1H), 4.49 (br s, 1H), 3.32–3.23 (m, 1H), 2.49–2.43 (m, 1H), 2.29–2.20 (m, 1H), 2.24 (s, 6H), 1.84–1.79 (m, 2H), 1.67–1.62 (m, 1H), 1.32 (s, 9H), 1.27–1.01 (m, 4H). ^{13}C NMR (75 MHz, $CDCl_3$) δ/ppm: 158.5, 66.7, 51.8, 50.1, 39.8, 33.9, 29.5, 25.3, 24.6, 21.3. HRMS (ESI-Orbitrap) *m/z*: $[M + H]^+$ calcd for $C_{13}H_{27}N_3O$ 242.2227, found 242.2235.

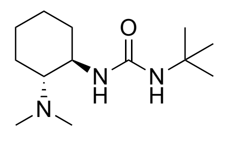

A45

Compound **A52 1-((1*R*,2*R*)-2-(azepan-1-yl)cyclohexyl)-3-(3,5-bis(trifluoromethyl)phenyl)thiourea**: Prepared according to the literature.[23] White solid. ^1H NMR (400 MHz, $CDCl_3$) δ/ppm: 7.89 (s, 2H), 7.66 (s, 1H), 3.87 (s, 1H), 2.78–2.59 (6H, overlapped), 1.91 (d, J = 11.7 Hz, 1H), 1.85 (dd, J = 12.2, 1.6 Hz, 1H), 1.74 (d, J = 13.3 Hz, 1H), 1.47–1.11 (m, 13H). ^{13}C NMR (100 MHz, $CDCl_3$) δ/ppm: 180.7, 139.7, 132.6 (q, $^2J_{CF}$ = 33.7 Hz), 123.5, 122.9 (q, $^1J_{CF}$ = 273.6 Hz), 118.4, 69.1, 55.8, 50.6, 32.1, 28.1, 26.7, 25.2, 24.3, 23.9. ^{19}F NMR (376 MHz, $CDCl_3$) δ/ppm: −62.91 (s, 6F).

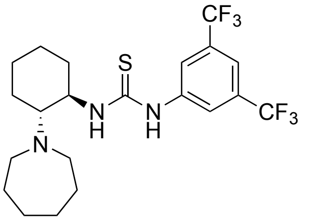

A52

Synthesis of compound **A54 1-(3,5-bis(trifluoromethyl)phenyl)-3-((1*R*,2*R*)-2-((cyclopropylmethyl)-(methyl)amino)cyclohexyl)thiourea**:

Step 1: Compound **VII (*S*,1*R*,2*R*)-N^1-(cyclopropylmethyl)-N^1-methylcyclohexane-1,2-diaminium trifluoroacetate**: Cyclopropanecarboxaldehyde (374 μL, 5 mmol) was added to a solution of *tert*-butyl (1*R*,2*R*)-2-aminocyclohexylcarbamate (214 mg, 1 mmol) in MeOH (5 mL) and the mixture was left to stir at rt for 1 h. After that, it was cooled in an ice bath, and $NaBH_4$ (187 mg, 5 mmol) was added portionwise. The reaction mass was stirred for 1 h at 0 °C, then an additional hour at rt. Aqueous formaldehyde (37%, 140 μL, 5 mmol) was added, followed by $NaBH_3CN$ (372 mg, 6 mmol) and the resulting mixture was left to stir overnight. Then, it was diluted with water and repetitively extracted with CH_2Cl_2. The combined organic layers were washed with brine, dried over anhydrous Na_2SO_4, filtered, and evaporated. The crude product was dissolved in CH_2Cl_2 (5 mL), trifluoroacetic acid (1.5 mL, 20 mmol) was added, and the mixture was stirred at rt overnight under Ar. Volatiles were evaporated *in vacuo*, and the residue was kneaded with Et_2O. The precipitated solid was filtered off, and the filter cake was further washed with several portions of Et_2O to afford **VII** as a white powder, yield 310 mg (76%), which was used directly in the next step.

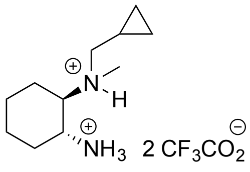

VII

Step 2: Compound **A54 1-(3,5-bis(trifluoromethyl)phenyl)-3-((1*R*,2*R*)-2-((cyclopropylmethyl)(methyl)amino)cyclohexyl)thiourea**: The mixture of compound **VII** (150 mg, 0.37 mmol), Et_3N (206 μL, 1.48 mmol), and 3,5-bis(trifluoromethyl)phenyl isothiocyanate (73 μL, 0.41 mmol) in anhydrous CH_2Cl_2 (3 mL) was stirred at rt overnight under Ar. After that, it was diluted with CH_2Cl_2 and washed with a saturated aqueous solution of $NaHCO_3$, brine, dried over anhydrous Na_2SO_4, filtered, and evaporated. The residue was subjected to column chromatography (SiO_2, CH_2Cl_2–MeOH = 97:3). Colorless glassy solid, yield 84 mg (52%). $[\alpha]^{25}_D$ −26 (*c* 0.5, MeOH). ^1H NMR (400 MHz, $CDCl_3$) δ/ppm: 7.97 (s, 2H), 7.61 (s, 1H), 3.85 (br s, 1H), 2.75 (br s, 1H), 2.58–2.51 (m, 3H), 2.43 (s, 3H), 1.93–1.76 (m, 3H), 1.35–1.16 (m, 4H), 0.83 (br s, 1H), 0.56–0.52 (m, 2H), 0.13–0.12 (m, 2H). ^{13}C NMR (100 MHz, $CDCl_3$) δ/ppm: 132.2 (q, $^2J_{CF}$ = 31.8 Hz), 123.1 (q, $^1J_{CF}$ = 272.9 Hz), 123.0, 117.9, 24.7, 24.4, 8.9, 4.4, 3.9. ^{19}F NMR (376 MHz, $CDCl_3$) δ/ppm: −62.88 (s, 6F). HRMS (ESI-Orbitrap) *m/z*: $[M + H]^+$ calcd for $C_{20}H_{26}F_6N_3S$ 454.1746, found 454.1762.

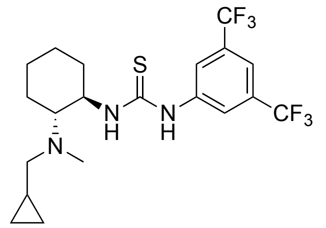

A54

Synthesis of compound **A55 1-(3,5-bis(trifluoromethyl)phenyl)-3-((1*R*,2*R*)-2-(methyl(neopentyl)amino)-cyclohexyl)thiourea**:

Step 1: Compound **VIII (*S*,1*R*,2*R*)-*N*[1]-methyl-*N*[1]-neopentylcyclohexane-1,2-diaminium trifluoroacetate**: Prepared according to the procedure reported for **A54**. Pivaldehyde (543 μL, 5 mmol) was added to a solution of *tert*-butyl (1*R*,2*R*)-2-aminocyclohexylcarbamate (214 mg, 1 mmol) in MeOH (5 mL) and the mixture was left to stir at rt for 1 h. After that, it was cooled in an ice bath, and $NaBH_4$ (187 mg, 5 mmol) was added portionwise. The reaction mass was stirred for 1 h at 0 °C, then an additional hour at rt. Aqueous formaldehyde (37%, 140 μL, 5 mmol) was added, followed by $NaBH_3CN$ (372 mg, 6 mmol) and the resulting mixture was left to stir overnight. Then, it was diluted with water and repetitively extracted with CH_2Cl_2. The combined organic layers were washed with brine, dried over anhydrous Na_2SO_4, filtered, and evaporated. The crude product was dissolved in CH_2Cl_2 (5 mL), trifluoroacetic acid (1.5 mL, 20 mmol) was added, and the mixture was stirred at rt overnight under Ar. Volatiles were evaporated *in vacuo*, and the residue was kneaded with Et_2O. The precipitated solid was filtered off, and the filter cake was further washed with several portions of Et_2O to afford **VIII** as a white powder, yield 254 mg (60%), which was used directly in the next step.

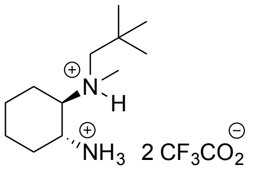

VIII

Step 2: Compound **A55 1-(3,5-bis(trifluoromethyl)phenyl)-3-((1*R*,2*R*)-2-(methyl(neopentyl)amino)-cyclohexyl)thiourea**:

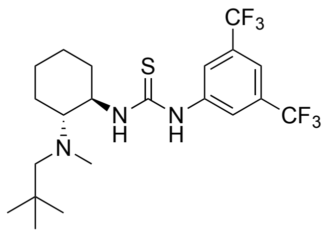

A55

Prepared by the addition of 3,5-bis(trifluoromethyl)phenyl isothiocyanate (71 μL, 0.39 mmol) in dry CH_2Cl_2 (1 mL) to a mixture of compound **VIII** (150 mg, 0.35 mmol) and Et_3N (195 μL, 1.4 mmol) in dry CH_2Cl_2 (3 mL), according to the procedure reported for **A43**. The crude product was purified by column chromatography (SiO_2, CH_2Cl_2–MeOH = 97:3). White solid, yield 120 mg (73%). M.p. 126–127 °C. $[\alpha]^{25}_{D}$ +32 (*c* 1.0, $CHCl_3$). ^{1}H NMR (400 MHz, $CDCl_3$) δ/ppm: 8.65 (br s, 1H), 7.77 (s, 2H), 7.70 (s, 1H), 6.81 (br s, 1H), 3.79–3.73 (m, 1H), 2.84–2.81 (m, 1H), 2.29–2.24 (m, 1H), 2.15, 2.04 (q, AB, J_{AB} = 14.0 Hz, 2H), 2.13 (s, 3H, partially overlapped with the previous signal), 1.85–1.71 (m, 3H), 1.40–1.06 (m, 4H), 0.56 (s, 9H). ^{13}C NMR (100 MHz, $CDCl_3$) δ/ppm: 181.5, 139.0, 133.2 (q, $^2J_{CF}$ = 34.0 Hz), 124.6, 122.7 (q, $^1J_{CF}$ = 273.3 Hz), 119.6, 68.7, 67.2, 56.9, 37.8, 32.8, 32.3, 27.7, 25.4, 24.5, 22.9. ^{19}F NMR (376 MHz, $CDCl_3$) δ/ppm: −62.98 (s, 6F). HRMS (ESI-Orbitrap) *m/z*: $[M + H]^+$ calcd for $C_{21}H_{30}F_6N_3S$ 470.2059, found 470.2099.

3.2 Syntheses of Formaldehyde Surrogates

Analytical data for the prepared compounds are listed below:

Compound **3a** **1-(hydroxymethyl)pyrrolidine-2,5-dione**:[24] Succinimide (2 g, 20 mmol) was refluxed for 90 min (oil bath) in 37% aqueous formaldehyde (2 mL), then cooled down, and the whole reaction mixture was evaporated to viscous oil (80 °C, 20 mbar). The flask was put in an ice bath afterward and left there until the reaction mixture solidified. Toluene (30 mL) and a spoon of Na_2SO_4 were added thereto, and the resulting mixture was heated at 85–90 °C (oil bath) to dissolve as much *N*-hydroxymethylsuccinimide as possible, then filtered or decanted while still hot. The residual solid was boiled again in 10 mL of additional toluene (repeated 2–3 times). The combined toluene filtrates were evaporated to provide a white fluffy solid (1.1 g), which was dissolved in a small amount of EtOAc at 50–60 °C (oil bath) and precipitated with a slight excess of *n*-heptane. The obtained white solid (yield 0.8 g, 31%) was preserved in a freezer as it decomposed gradually back to the starting material. ^{1}H NMR (300 MHz, $CDCl_3$) δ/ppm: 5.07 (d, *J* = 8.1 Hz, 2H), 3.36 (t, *J* = 8.1 Hz, 1H), 2.76 (s, 4H). ^{13}C NMR (75 MHz, $CDCl_3$) δ/ppm: 176.9, 62.4, 28.2.

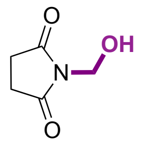
3a

Compound **3b** **1,3-bis(hydroxymethyl)imidazolidine-2,4,5-trione**:[25] white solid. ^{1}H NMR (400 MHz, DMSO-d_6) δ/ppm: 6.69 (br s, 2H), 4.90 (s, 4H). ^{13}C NMR (100 MHz, DMSO-d_6) δ/ppm: 156.9, 153.3, 61.5.

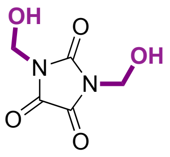
3b

Compound **3c** **1-(hydroxymethyl)pyrrolidin-2-one**:[26] white solid. ^{1}H NMR (400 MHz, DMSO-d_6) δ/ppm: 5.76 (br s, 1H), 4.57 (d, *J* = 4.8 Hz, 2H), 3.41–3.37 (m, 2H), 2.24–2.20 (m, 2H), 1.94–1.86 (m, 2H). ^{13}C NMR (100 MHz, DMSO-d_6) δ/ppm: 174.0, 64.8, 44.9, 30.9, 17.4.

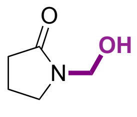
3c

Compound **3d** **(1*H*-pyrrol-1-yl)methanol**:[27] colorless oil. ^{1}H NMR (400 MHz, DMSO-d_6) δ/ppm: 6.82 (t, *J* = 2.1 Hz, 2H), 6.37 (t, *J* = 7.4 Hz, 1H), 6.00 (t, *J* = 2.1 Hz, 2H), 5.16 (d, *J* = 7.4 Hz, 2H). ^{13}C NMR (100 MHz, DMSO-d_6) δ/ppm: 120.2, 108.0, 71.3.

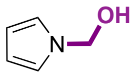
3d

Compound **3e (1*H*-pyrazol-1-yl)methanol**:[28] white solid. ^{1}H NMR (400 MHz, DMSO-d_6) δ/ppm: 7.78 (dd, *J* = 2.3, 0.5 Hz, 1H), 7.47 (app d, *J* = 1.3 Hz, 1H), 6.71 (t, *J* = 7.6 Hz, 1H), 6.27 (app t, *J* = 2.0 Hz, 1H), 5.36 (d, *J* = 7.6 Hz, 2H). ^{13}C NMR (100 MHz, DMSO-d_6) δ/ppm: 138.8, 129.3, 105.6, 73.2.

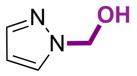

3e

Compound **3f (1*H*-imidazol-1-yl)methanol**:[29] white solid. ^{1}H NMR (400 MHz, DMSO-d_6) δ/ppm: 7.69 (s, 1H), 7.20 (t, *J* = 1.1 Hz, 1H), 6.90 (s, 1H), 6.70 (br s, 1H), 5.28 (s, 2H). ^{13}C NMR (100 MHz, DMSO-d_6) δ/ppm: 128.5, 118.1, 69.0.

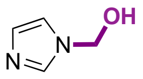

3f

Compounds **3g (2*H*-1,2,3-triazolyl)methanol** – a mixture of regioisomers (r.r. = 3:1):[30] colorless oil. ^{1}H NMR (400 MHz, DMSO-d_6) δ/ppm: 7.83 (s, 2H), 7.19 (t, *J* = 7.9 Hz, 1H), 5.62 (d, *J* = 7.9 Hz, 2H). ^{13}C NMR (100 MHz, DMSO-d_6) δ/ppm: 134.9, 75.7 (spectra of the major ^{2}*N*-substituted regioisomer).

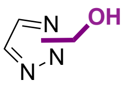

3g

Compound **3h (4,5-dibromo-2*H*-1,2,3-triazol-2-yl)methanol**:[31] white solid. ^{1}H NMR (400 MHz, $CDCl_3$) δ/ppm: 5.74 (d, *J* = 8.1 Hz, 2H), 3.89 (t, *J* = 8.1 Hz, 1H). ^{13}C NMR (100 MHz, $CDCl_3$) δ/ppm: 126.3, 77.5.

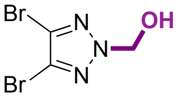

3h

Compound **3i dimethyl 2-(hydroxymethyl)-2*H*-1,2,3-triazole-4,5-dicarboxylate**:[31] white solid, which was stored in a screw-cap amber vial at rt over one year without any apparent decomposition. ^{1}H NMR (400 MHz, DMSO-d_6) δ/ppm: 7.68 (t, *J* = 8.1 Hz, 1H), 5.73 (d, *J* = 8.1 Hz, 2H), 3.89 (s, 6H). ^{13}C NMR (100 MHz, DMSO-d_6) δ/ppm: 160.0, 139.2, 77.9, 52.7.

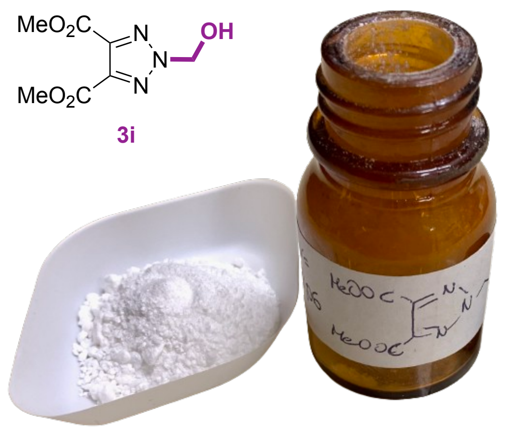

Compound **3j (1*H*-1,2,4-triazol-1-yl)methanol**:[32] white solid. ^{1}H NMR (400 MHz, DMSO-d_6) δ/ppm: 8.59 (s, 1H), 7.99 (s, 1H), 6.99 (br s, 1H), 5.46 (s, 2H). ^{13}C NMR (100 MHz, DMSO-d_6) δ/ppm: 151.6, 71.1.

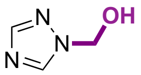

3j

Compounds **3k (1*H*-tetrazolyl)methanol** – a mixture of regioisomers (r.r. = 6.5:1):[33] white solid. ^{1}H NMR (400 MHz, DMSO-d_6) δ/ppm: 9.47 (s, 1H), 7.44 (t, J = 7.4 Hz, 1H), 5.77 (d, J = 6.4 Hz, 2H). ^{13}C NMR (100 MHz, DMSO-d_6) δ/ppm: 143.6, 70.5 (spectra of the major regioisomer).

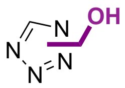

3k

Compound **3m 2-(((trimethylsilyl)oxy)methyl)isoindoline-1,3-dione**:[34] white solid. ^{1}H NMR (400 MHz, $CDCl_3$) δ/ppm: 7.94–7.90 (m, 2H), 7.80–7.76 (m, 2H), 5.10 (s, 2H), 3.43 (s, 3H). ^{13}C NMR (100 MHz, $CDCl_3$) δ/ppm: 168.0, 134.4, 131.8, 123.7, 68.7, 57.4.

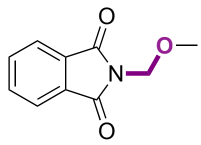

3m

Compound **3n 2-(hydroxymethyl)-5-nitroisoindoline-1,3-dione**:[35] yellowish solid. ^{1}H NMR (400 MHz, DMSO-d_6) δ/ppm: 8.65 (dd, J = 8.2, 2.1 Hz, 1H), 8.54 (d, J = 2.1 Hz, 1H), 8.17 (d, J = 2.1 Hz, 1H), 6.54 (br s, 1H), 5.00 (s, 2H). ^{13}C NMR (100 MHz, DMSO-d_6) δ/ppm: 165.9, 165.6, 151.6, 136.2, 132.9, 129.9, 124.9, 118.3, 60.7.

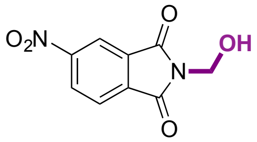

3n

Compound **3o 2-(hydroxymethyl)benzo[*d*]isothiazol-3(2*H*)-one 1,1-dioxide**:[36] white solid. ^{1}H NMR (300 MHz, $CDCl_3$) δ/ppm: 8.12–8.08 (m, 1H), 7.97–7.87 (m, 3H), 5.40 (s, 2H), 3.42 (br s, 1H). ^{13}C NMR (75 MHz, $CDCl_3$) δ/ppm: 159.1, 137.9, 135.4, 134.6, 127.0, 125.6, 121.2, 63.3.

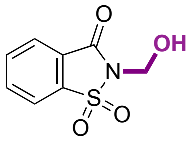

3o

Compound **3p (1*H*-indazol-1-yl)methanol**:[37] white solid. ^{1}H NMR (400 MHz, DMSO-d_6) δ/ppm: 8.09 (s, 1H), 7.78–7.70 (m, 2H), 7.43–7.39 (m, 1H), 7.19–7.15 (m, 1H), 6.67 (t, J = 6.2 Hz, 1H), 5.72 (d, J = 6.2 Hz, 2H). ^{13}C NMR (100 MHz, DMSO-d_6) δ/ppm: 139.0, 133.3, 126.11, 124.2, 120.8, 110.1, 70.7.

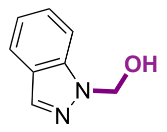

3p

Compound **3q (1*H*-benzo[*d*]imidazol-1-yl)methanol**:[38] white solid. ^{1}H NMR (400 MHz, DMSO-d_6) δ/ppm: 8.27 (s, 1H), 7.67–7.65 (m, 2H), 7.29–7.20 (m, 2H), 6.71 (t, J = 7.4 Hz, 1H), 5.60 (d, J = 7.4 Hz, 2H). ^{13}C NMR (100 MHz, DMSO-d_6) δ/ppm: 143.7, 133.3, 122.4, 121.8, 119.4, 110.9, 67.3.

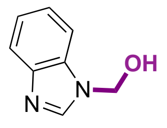

3q

Compounds **3s (4-nitro-1*H*-benzo[*d*][1,2,3]triazolyl)methanol** – a mixture of regioisomers (r.r. = 33.5:7.5:1): 4-Nitrobenzotriazole (1.0 g, 6.1 mmol) was suspended in 37% aqueous formaldehyde (20 mL). The mixture was refluxed overnight (oil bath). After cooling to room temperature, the solid precipitate was filtered off, washed with water, and dried *in vacuo*. Yellowish solid, yield 970 mg (82%). ^{1}H NMR (400 MHz, DMSO-d_6) δ/ppm: 8.43 (dd, J = 8.3, 0.7 Hz, 1H), 8.35 (dd, J = 7.7, 0.7 Hz, 1H), 7.83 (dd, J = 8.3, 7.7 Hz, 1H), 7.47 (t, J = 7.9 Hz, 1H), 6.14 (d, J = 7.9 Hz, 2H). ^{13}C NMR (100 MHz, DMSO-d_6) δ/ppm: 137.9, 137.8, 134.7, 127.2, 121.7, 119.0, 71.1 (spectra of the major regioisomer). HRMS (APCI) m/z: $[M - CH_2O + H]^+$ calcd for $C_6H_5N_4O_2$ 165.0407, found 165.0405.

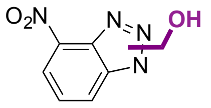

3s

Compounds **3t (5,6-difluoro-1*H*-benzo[*d*][1,2,3]triazolyl)methanol** – a mixture of regioisomers (r.r. = 12.5:1): 5,6-Difluorobenzotriazole (0.5 g, 3.2 mmol) was suspended in 37% aqueous formaldehyde (10 mL). The mixture was refluxed overnight (oil bath). After cooling to room temperature, the solid precipitate was filtered off, washed with water, and dried *in vacuo*. Yellow solid, yield 424 mg (71%). ^{1}H NMR (400 MHz, DMSO-d_6) δ/ppm: 8.25–8.20 (m, 1H), 8.10–8.06 (m, 1H), 7.28 (t, J = 7.5 Hz, 1H), 6.00 (d, J = 7.5 Hz, 2H). ^{13}C NMR (100 MHz, DMSO-d_6) δ/ppm: 147.0 (dd, J_{CF} = 238.9, 15.4 Hz), 146.7 (dd, J_{CF} = 237.5, 15.0 Hz), 138.9 (d, J_{CF} = 10.6 Hz), 128.8 (d, J_{CF} = 10.6 Hz), 106.8 (d, J_{CF} = 19.3 Hz), 99.3 (d, J_{CF} = 23.1 Hz), 67.7. ^{19}F NMR (376 MHz, DMSO-d_6) δ/ppm: −134.03 to −134.13 (m, 1F), −139.34 to −139.44 (m, 1F) (spectra of the major ^1N-substituted regioisomer). HRMS (APCI) m/z: $[M + H]^+$ calcd for $C_7H_6F_2N_3O$ 186.0473, found 186.0474.

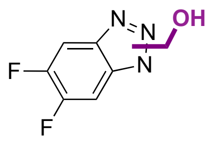

3t

Compound **3u** **3-(hydroxymethyl)benzo[*d*][1,2,3]triazin-4(3*H*)-one**:[39] white solid. ^{1}H NMR (400 MHz, DMSO-d_6) δ/ppm: 8.29–8.28 (m, 1H), 8.22–8.20 (m, 1H), 8.12–8.08 (m, 1H), 7.96–7.92 (m, 1H), 7.05 (t, J = 7.5 Hz, 1H), 5.59 (d, J = 7.5 Hz, 2H). ^{13}C NMR (100 MHz, DMSO-d_6) δ/ppm: 154.7, 143.8, 135.7, 133.1, 128.1, 124.7, 119.6, 71.8.

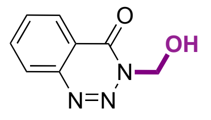

3u

Compound **4f** **((1*H*-benzo[*d*][1,2,3]triazol-1-yl)oxy)methanol**:[40] white solid. ^{1}H NMR (400 MHz, DMSO-d_6) δ/ppm: 7.95–7.93 (m, 1H), 7.91–7.88 (m, 1H), 7.74–7.70 (m, 1H), 7.46 (ddd, J = 8.4, 7.0, 0.8 Hz, 1H), 7.25 (t, J = 7.6 Hz, 1H), 5.76 (d, J = 7.3 Hz, 2H). ^{13}C NMR (100 MHz, DMSO-d_6) δ/ppm: 133.4, 130.5, 129.9, 124.5, 114.7, 112.2, 70.5.

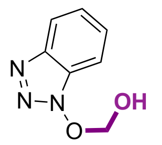

4f

Compound **5** **(hydroxymethyl)triphenylphosphonium tetrafluoroborate**:[41] white solid. ^{1}H NMR (400 MHz, DMSO-d_6) δ/ppm: 7.93–7.89 (m, 3H), 7.81–7.76 (m, 12H), 7.00 (br s, 1H), 5.62 (s, 2H). ^{13}C NMR (100 MHz, DMSO-d_6) δ/ppm: 135.0, 134.0 (d, J_{CP} = 9.6 Hz), 130.2 (d, J_{CP} = 12.5 Hz), 117.4 (d, J_{CP} = 82.8 Hz), 56.2 (d, J_{CP} = 60.7 Hz). ^{19}F NMR (376 MHz, DMSO-d_6) δ/ppm: −148.12 (s, 0.81F, corresponding to $^{10}BF_4^-$), −148.18 (br s, 3.19F, corresponding to $^{11}BF_4^-$). ^{31}P{^{1}H} NMR (162 MHz, DMSO-d_6) δ/ppm: 18.2 (s).

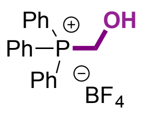

5

3.3 Syntheses of Isoindolinones

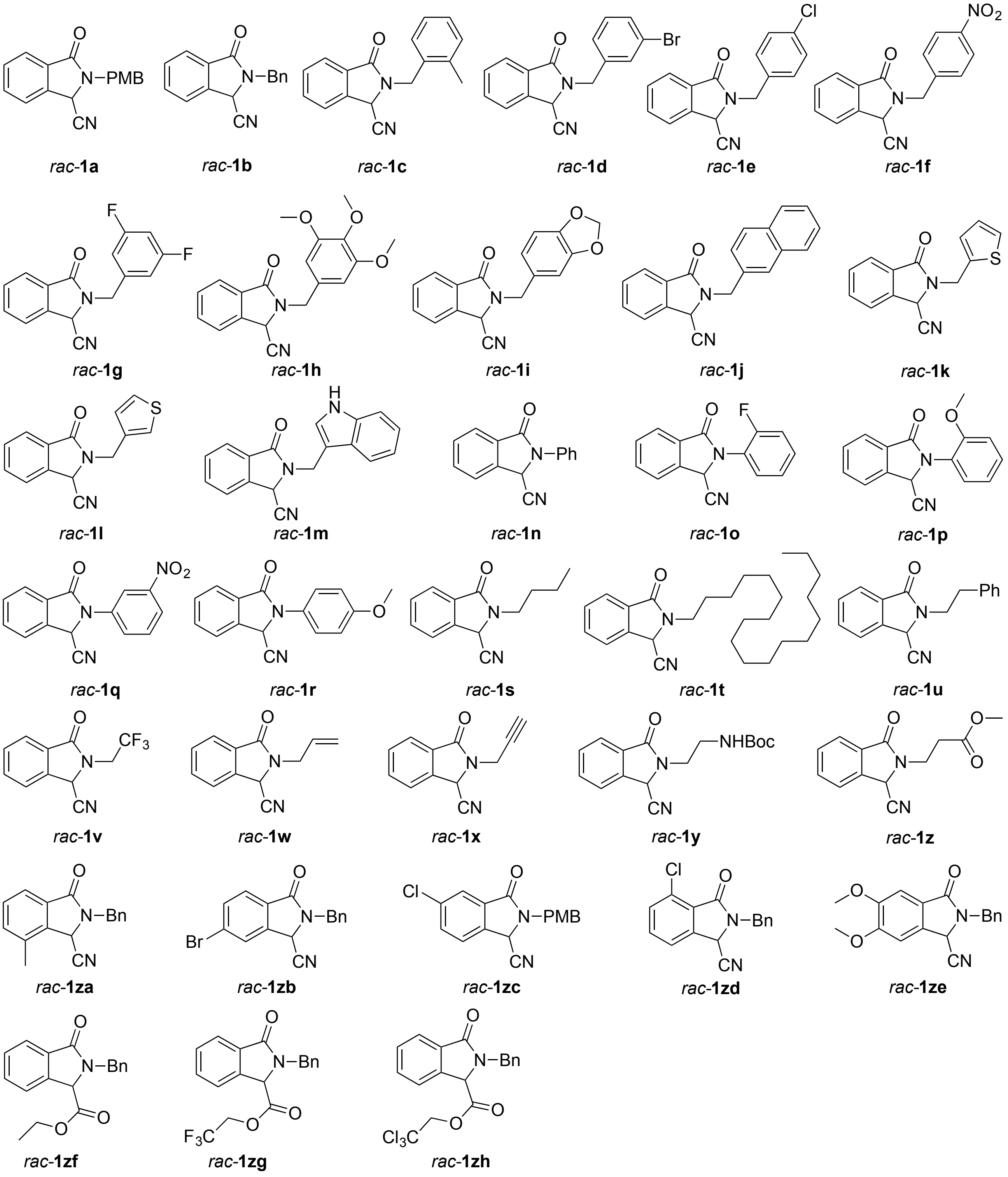

Figure S17: Numbering of the starting material.

Analytical data for the prepared compounds are listed below:

Compound **IX methyl 2-formyl-3-methylbenzoate**:[42] Prepared according to the literature procedure[42] from

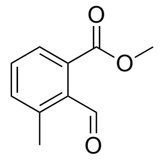
IX

2-methylbenzaldehyde (0.5 mL, 4.3 mmol), potassium 2-methoxy-2-oxoacetate (1.22 g, 8.6 mmol), $Pd(OAc)_2$ (97 mg, 0.43 mmol), $K_2S_2O_8$ (3.50 g, 13 mmol), $AgNO_3$ (1.42 g, 5.1 mmol), 4-fluoro-3-(trifluoromethyl)aniline (280 μL, 2.15 mmol), hexafluoroisopropanol (12 mL), and 1,2-dichloroethane (3 mL). The crude product was purified by column chromatography (SiO_2, *n*-hexane–EtOAc 20:1). Waxy brown solid, yield 200 mg (26%). ^{1}H NMR (400 MHz, $CDCl_3$) δ/ppm: 10.48 (s, 1H), 7.76 (dd, *J* = 6.8, 2.0 Hz, 1H), 7.48–7.42 (m, 2H), 3.94 (s, 3H), 2.51 (s, 3H). ^{13}C NMR (100 MHz, $CDCl_3$) δ/ppm: 194.1, 167.5, 138.2, 137.0, 135.2, 131.6, 131.0, 127.6, 52.8, 19.9.

Synthesis of compound **XII methyl 4-bromo-2-formylbenzoate**:[43]

Step 1: Compound **X 3,5-dibromoisobenzofuran-1(3*H*)-one**: A mixture of 5-bromoisobenzofuran-1(3*H*)-

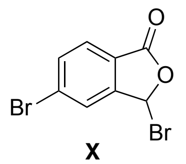
X

one (1.0 g, 4.7 mmol), NBS (0.92 g, 5.2 mmol), and AIBN (39 mg, 0.24 mmol) were refluxed in CCl_4 (15 mL) overnight under Ar (oil bath). The volatiles were evaporated *in vacuo*, the residue was subjected to column chromatography (SiO_2, *n*-hexane–EtOAc 5:1), providing yellowish solid, yield 1.22 g (89%), which was carried forward in the next step.

Step 2: Compound **XI 5-bromo-3-hydroxyisobenzofuran-1(3*H*)-one**: A product from the previous step (**X**)

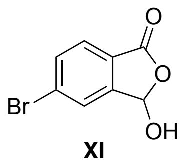
XI

was suspended in water (10 mL) and refluxed for 2 h. After cooling in an ice bath, the precipitate was filtered off, washed with water, and dried in *vacuo*. The resulting product obtained as a white solid, yield 770 mg (80%), was used directly in the next step.

Step 3: Compound **XII methyl 4-bromo-2-formylbenzoate**:[43] A product **XI** from the previous step

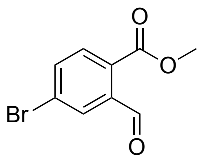
XII

(770 mg, 3.4 mmol), K_2CO_3 (255 mg, 1.8 mmol), and iodomethane (400 μL, 6.4 mmol) suspended in dry DMF (5 mL) were heated at 60 °C for 1 h under Ar (oil bath). Then, the reaction mixture was diluted with water, and the aqueous layer was repetitively extracted with EtOAc. The combined organic phases were washed with cold water, a saturated aqueous solution of $NaHCO_3$, brine, dried over anhydrous Na_2SO_4, filtered, and evaporated *in vacuo*. White solid, yield 770 mg (67% over 3 steps). ^{1}H NMR (400 MHz, $CDCl_3$) δ/ppm: 10.61 (s, 1H), 8.06 (d, *J* = 2.1 Hz, 1H), 7.88 (d, *J* = 8.3 Hz, 1H), 7.77 (dd, *J* = 8.3, 2.1 Hz, 1H), 3.98 (s, 3H). ^{13}C NMR (100 MHz, $CDCl_3$) δ/ppm: 190.7, 165.9, 138.5, 135.8, 132.1, 131.4, 130.4, 127.7, 52.9.

Compound **XIII 7-chloroisobenzofuran-1(3*H*)-one**:[44] Prepared according to the literature procedure[44] from

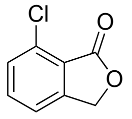

XIII

2-chlorobenzoic acid (783 mg, 5 mmol), $Pd(OAc)_2$ (112 mg, 0.5 mmol), K_2HPO_4 (2.61 g, 15 mmol), and CH_2Br_2 (10 mL). The crude product was purified by column chromatography (SiO_2, *n*-hexane–EtOAc 5:1 → 2:1). Yellow oil, yield 325 mg (39%). ^{1}H NMR (400 MHz, $CDCl_3$) δ/ppm: 7.62 (dd, *J* = 7.9, 7.6 Hz, 1H), 7.49 (dd, *J* = 7.9, 0.6 Hz, 1H), 7.40 (dd, *J* = 7.6, 0.8 Hz, 1H), 5.29 (s, 1H). ^{13}C NMR (100 MHz, $CDCl_3$) δ/ppm: 168.0, 148.8, 135.0, 133.5, 130.5, 122.5, 120.5, 68.3.

Compound **XIV methyl 2-chloro-6-formylbenzoate**:[45] Prepared according to the procedure described for

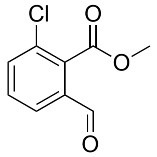

XIV

compound **XII**. **Step 1**: 7-Chloroisobenzofuran-1(3*H*)-one (300 mg, 1.78 mmol), NBS (316 mg, 1.78 mmol), and AIBN (15 mg, 0.09 mmol) were refluxed in CCl_4 (5 mL) overnight under Ar (oil bath). The volatiles were evaporated *in vacuo*, and the residue was subjected to column chromatography (SiO_2, *n*-hexane–EtOAc, 5:1). Yield 360 mg (82%). **Step 2:** A product from the previous step was suspended in water (3 mL) and refluxed for 2 h (oil bath). After cooling in an ice bath, the precipitate was filtered off, washed with water, and dried *in vacuo*. The resulting product 202 mg (75%) was used directly in the next step. **Step 3**: A product from the previous step (200 mg, 1.08 mmol), K_2CO_3 (82 mg, 0.60 mmol), and iodomethane (128 µL, 2.05 mmol) suspended in dry DMF (2 mL) were heated at 60 °C for 1 h under Ar (oil bath). Then, the reaction mixture was diluted with water, and the aqueous layer was repetitively extracted with EtOAc. The combined organic phases were washed with cold water, a saturated aqueous solution of $NaHCO_3$, brine, dried over anhydrous Na_2SO_4, filtered, and evaporated *in vacuo*. Yellowish oil, yield 190 mg (89%, 54% over 3 steps). ^{1}H NMR (400 MHz, $CDCl_3$) δ/ppm: 9.96 (s, 1H), 7.80 (dd, *J* = 7.6, 1.2 Hz, 1H), 7.68 (dd, *J* = 8.1, 1.2 Hz, 1H), 7.57 (t, *J* = 7.8 Hz, 1H), 4.02 (s, 3H).

Compound **XV methyl 2-formyl-4,5-dimethoxybenzoate**:[46] Prepared according to the procedure described

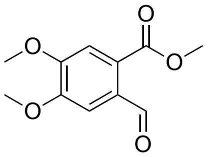

XV

for compound **XII**. **Step 1**: 5,6-Dimethoxyisobenzofuran-1(3*H*)-one (500 mg, 2.57 mmol), NBS (460 mg, 2.58 mmol), and AIBN (21 mg, 0.13 mmol) were refluxed in CCl_4 (10 mL) overnight under Ar (oil bath). The volatiles were evaporated *in vacuo*, and the residue was subjected to column chromatography (SiO_2, *n*-hexane–EtOAc, 5:1). Yield 410 mg (58%). **Step 2:** A product from the previous step was suspended in water (5 mL) and refluxed for 2 h (oil bath). After cooling in an ice bath, the precipitate was filtered off, washed with water, and dried *in vacuo*. The resulting product 228 mg (72%) was used directly in the next step. **Step 3**: A product from the previous step (220 mg, 1.05 mmol), K_2CO_3 (80 mg, 0.58 mmol), and iodomethane (124 µL, 2.00 mmol) suspended in dry DMF (2 mL) were heated at 60 °C for 1 h under Ar (oil bath). Then, the reaction mixture was diluted with water, and the aqueous layer was repetitively extracted with EtOAc. The combined organic phases were washed with cold water, a saturated aqueous solution of $NaHCO_3$, brine, dried over anhydrous Na_2SO_4, filtered, and evaporated *in vacuo*. White solid, yield 200 mg

(85%, 35% over 3 steps). ^{1}H NMR (400 MHz, $CDCl_3$) δ/ppm: 10.64 (s, 1H), 7.50 (s, 1H), 7.46 (s, 1H), 4.00 (s, 3H), 3.98 (s, 3H), 3.96 (s, 3H). ^{13}C NMR (100 MHz, $CDCl_3$) δ/ppm: 191.1, 166.2, 152.4, 151.9, 131.2, 126.0, 112.6, 109.6, 56.3, 56.2, 52.6.

Syntheses of isoindolinones were based on the literature procedure:[47]

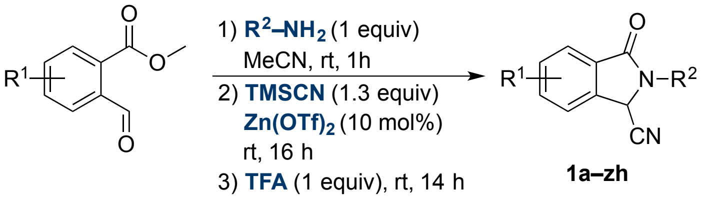

Please check the details below regarding the scale of each entry. The corresponding methyl 2-formylbenzoate (1 equiv) and amine (1 equiv) in dry MeCN (0.3 M) were stirred 1 h at rt under Ar. Then $Zn(OTf)_2$ (10 mol%) and TMS-CN (1.3 equiv) were added. The resulting mixture was left to stir at rt for 16 h under Ar. Thereafter, trifluoroacetic acid (1 equiv) was added, and the mixture was stirred under the same conditions for an additional 14 h. Then, the reaction was carefully diluted with a saturated aqueous solution of $NaHCO_3$ and extracted with EtOAc (4×). The combined organic extracts were washed with a saturated aqueous solution of $NaHCO_3$, brine, dried over anhydrous Na_2SO_4, filtered, and evaporated *in vacuo*. The crude product was purified by trituration with a mixture of EtOAc–*n*-heptane or chromatographed on SiO_2 (EtOAc–*n*-hexane) as specified hereunder for each compound.

Compound **1a** ***rac*-2-(4-methoxybenzyl)-3-oxoisoindoline-1-carbonitrile**:[48] Prepared from methyl 2-formylbenzoate (1.0 g, 0.85 mL, 6.1 mmol) and 4-methoxybenzylamine (0.79 mL, 6.1 mmol) following the above general procedure. The crude product was triturated with EtOAc–*n*-heptane (1:10) to provide a precipitate, which was filtered off, washed, and dried *in vacuo*. White powder, yield 1.15 g (68%). M.p. 153–154 °C (lit.[48] m.p. 152–154 °C). ^{1}H NMR (300 MHz, $CDCl_3$) δ/ppm: 7.95–7.92 (m, 1H), 7.69–7.57 (m, 3H), 7.32–7.29 (m, 2H), 6.92–6.87 (m, 2H), 5.45 (d, J = 14.9 Hz, 1H), 5.07 (s, 1H), 4.25 (d, J = 14.9 Hz, 1H), 3.81 (s, 3H). ^{13}C NMR (75 MHz, $CDCl_3$) δ/ppm: 166.8, 159.7, 136.8, 132.9, 131.3, 130.4, 130.0, 127.2, 124.6, 123.1, 114.6, 114.5, 55.3, 48.7, 44.4.

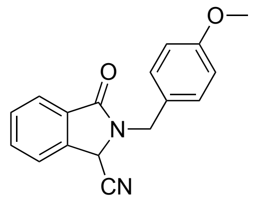

1a

Compound **1b** ***rac*-2-benzyl-3-oxoisoindoline-1-carbonitrile**:[47] White powder. ^{1}H NMR (400 MHz, DMSO-d_6) δ/ppm: 7.86–7.84 (m, 1H), 7.81–7.75 (m, 2H), 7.70–7.66 (m, 1H), 7.40–7.29 (m, 5H), 5.91 (s, 1H), 5.01 (d, J = 15.6 Hz, 1H), 4.60 (d, J = 15.6 Hz, 1H).

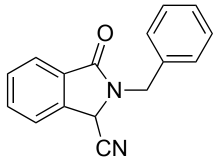

1b

Compound **1c** ***rac*-2-(2-methylbenzyl)-3-oxoisoindoline-1-carbonitrile**:[48] White powder. ^{1}H NMR (400 MHz, $CDCl_3$) δ/ppm: 7.95–7.93 (m, 1H), 7.69–7.58 (m, 3H), 7.28–7.24 (m, 1H), 7.17–7.14 (m, 3H), 6.49 (d, J = 15.0 Hz, 1H), 5.09 (s, 1H), 4.25 (d, J = 15.0 Hz, 1H), 2.35 (s, 3H).

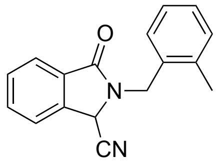

1c

Compound **1d** ***rac*-2-(3-bromobenzyl)-3-oxoisoindoline-1-carbonitrile**:[49] Colorless powder. ^{1}H NMR (400 MHz, $CDCl_3$) δ/ppm: 7.96–7.95 (m, 1H), 7.72–7.61 (m, 3H), 7.51–7.47 (m, 2H), 7.32–7.30 (m, 1H), 7.28–7.24 (m, 1H), 5.45 (d, J = 15.2 Hz, 1H), 5.13 (s, 1H), 4.30 (d, J = 15.2 Hz, 1H).

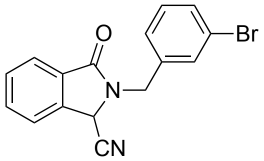

1d

Compound **1e** ***rac*-2-(4-chlorobenzyl)-3-oxoisoindoline-1-carbonitrile**:[48] White powder. ^{1}H NMR (400 MHz, $CDCl_3$) δ/ppm: 7.95–7.93 (m, 1H), 7.71–7.60 (m, 3H), 7.36–7.34 m, 2H), 7.32–7.30 (m, 2H), 5.44 (d, J = 15.2 Hz, 1H), 5.10 (s, 1H), 4.31 (d, J = 15.2 Hz, 1H).

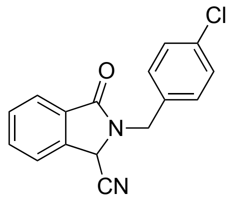

1e

Compound **1f** ***rac*-2-(4-nitrobenzyl)-3-oxoisoindoline-1-carbonitrile**: Prepared from methyl 2-formylbenzoate (328 mg, 278 μL, 2 mmol) and 4-nitrobenzylamine (304 mg, 2 mmol) following the above general procedure. The crude product was triturated with EtOAc–*n*-heptane (1:10) to provide a precipitate, which was filtered off, washed, and dried *in vacuo*. Yellowish powder, yield 436 mg (74%). M.p. 168–169 °C. ^{1}H NMR (400 MHz, DMSO-d_6) δ/ppm: 8.24–8.22 (m, 2H), 7.87–7.77 (m, 3H), 7.71–7.68 (m, 1H), 7.63–7.61 (m, 2H), 6.05 (s, 1H), 5.07,

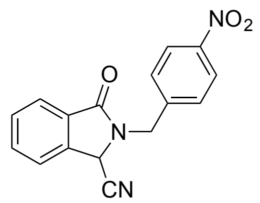

1f

4.87 (q, AB, J_{AB} = 16.4 Hz, 2H). ^{13}C NMR (100 MHz, DMSO-d_6) δ/ppm: 167.0, 147.0, 144.3, 138.0, 133.4, 130.4, 130.2, 129.0, 123.91, 123.86, 123.8, 115.8, 50.1, 44.6. HRMS (APCI) *m/z*: $[M + H]^+$ calcd for $C_{16}H_{12}N_3O_3$ 294.0873, found 294.0874.

Compound **1g** ***rac*-2-(3,5-difluorobenzyl)-3-oxoisoindoline-1-carbonitrile**: Prepared from methyl 2-formylbenzoate (328 mg, 278 μL, 2 mmol) and 3,5-difluorobenzylamine (237 μL, 2 mmol) following the above general procedure. The crude product was triturated with EtOAc–*n*-heptane (1:10) to provide a precipitate, which was filtered off, washed, and dried *in vacuo*. White powder, yield 237 mg (42%). M.p. 192–194 °C. ^{1}H NMR (400 MHz, DMSO-d_6) δ/ppm: 7.86–7.77 (m, 3H), 7.71–7.67 (m, 1H), 7.20–7.14 (m, 1H), 7.10–7.08 (m, 2H), 6.03 (s, 1H), 4.95, 4.75 (q, AB, J_{AB} = 16.0 Hz, 2H). ^{13}C NMR (100 MHz, DMSO-d_6) δ/ppm: 166.8, 162.5 (dd, J_{CF} = 246.6, 12.5 Hz), 140.8 (app t, J_{CF} = 9.2 Hz), 137.9, 133.2, 130.2, 130.1, 123.7, 115.6, 111.0 (dd, J_{CF} = 19.3, 6.7 Hz), 103.0 (app t, J_{CF} = 26.0 Hz), 49.9, 44.3. ^{19}F NMR (376 MHz, DMSO-d_6) δ/ppm: −109.45 to −109.48 (m, 2F). HRMS (APCI) *m/z*: $[M + H]^+$ calcd for $C_{16}H_{11}F_2N_2O$ 285.0834, found 285.0836.

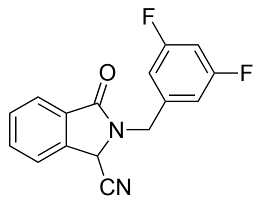

1g

Compound **1h** ***rac*-3-oxo-2-(3,4,5-trimethoxybenzyl)isoindoline-1-carbonitrile**:[49] White powder. ^{1}H NMR (400 MHz, $CDCl_3$) δ/ppm: 7.96–7.93 (m, 1H), 7.71–7.67 (m, 1H), 7.66–7.61 (m, 2H), 6.59 (s, 2H), 5.46 (d, *J* = 14.7 Hz, 1H), 5.11 (s, 1H), 4.20 (d, *J* = 14.7 Hz, 1H), 3.86 (s, 6H), 3.84 (s, 3H).

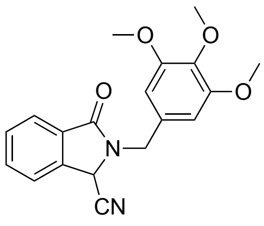

1h

Compound **1i** ***rac*-2-(benzo[*d*][1,3]dioxol-5-ylmethyl)-3-oxoisoindoline-1-carbonitrile**: Prepared from methyl 2-formylbenzoate (328 mg, 278 μL, 2 mmol) and 3,4-(methylenedioxy)benzylamine (248 μL, 2 mmol) following the above general procedure. The crude product was triturated with EtOAc–*n*-heptane (1:10) to provide a precipitate, which was filtered off, washed, and dried *in vacuo*. White powder, yield 472 mg (89%). M.p. 101–102 °C. ^{1}H NMR (400 MHz, DMSO-d_6) δ/ppm: 7.84–7.82 (m, 1H), 7.79–7.74 (m, 2H), 7.69–7.65 (m, 1H), 6.91–6.83 (m, 3H), 6.00 (m, 2H), 5.87 (s, 1H), 4.91 (d, *J* = 15.2 Hz, 1H), 4.51 (d, *J* = 15.2 Hz, 1H). ^{13}C NMR (100 MHz, DMSO-d_6) δ/ppm: 166.5, 147.5, 146.8, 137.8, 133.0, 130.4, 130.2, 129.6, 123.7, 123.6, 121.6, 115.6, 108.4, 108.3, 101.0, 49.3, 44.6. HRMS (ESI-Q-TOF) *m/z*: $[M + H]^+$ calcd for $C_{17}H_{13}N_2O_3$ 293.0921, found 293.0893.

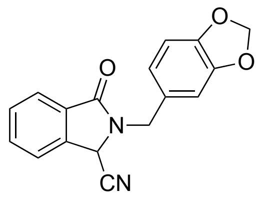

1i

Compound **1j** ***rac*-2-(naphthalen-2-ylmethyl)-3-oxoisoindoline-1-carbonitrile**: Prepared from methyl 2-formylbenzoate (328 mg, 278 µL, 2 mmol) and 1-(2-naphthyl)methanamine (314 mg, 2 mmol) following the above general procedure. The crude product was triturated with EtOAc–*n*-heptane (1:10) to provide a precipitate, which was filtered off, washed, and dried *in vacuo*. White powder, yield 400 mg (47%). M.p. 100–101 °C. ^{1}H NMR (400 MHz, $CDCl_3$) δ/ppm: 7.98–7.96 (m, 1H), 7.86–7.83 (m, 4H), 7.68–7.61 (m, 2H), 7.56–7.54 (m, 1H), 7.53–7.49 (m, 2H), 7.45–7.42 (m, 1H), 5.69 (d, *J* = 15.0 Hz, 1H), 5.08 (s, 1H), 4.45 (d, *J* = 15.0 Hz, 1H). ^{13}C NMR (100 MHz, $CDCl_3$) δ/ppm: 166.9, 136.8, 133.3, 133.0, 131.1. 130.4, 129.3, 127.84, 127.79, 127.7, 126.6, 126.5, 125.9, 124.7, 123.1, 114.5, 48.8, 45.1. HRMS (APCI) *m/z*: $[M + H]^+$ calcd for $C_{20}H_{15}N_2O$ 299.1179, found 299.1182.

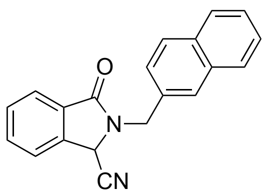

1j

Compound **1k** ***rac*-3-oxo-2-(thiophen-2-ylmethyl)isoindoline-1-carbonitrile**:[49] Colorless powder. ^{1}H NMR (400 MHz, $CDCl_3$) δ/ppm: 7.94–7.92 (m, 1H), 7.70–7.61 (m, 3H), 7.31 (dd, *J* = 5.1, 0.8 Hz, 1H), 7.16 (d, *J* = 3.5 Hz, 1H), 7.01 (dd, *J* = 5.1, 3.5 Hz, 1H), 5.59 (d, *J* = 15.6 Hz, 1H), 5.22 (s, 1H), 4.60 (d, *J* = 15.6 Hz, 1H).

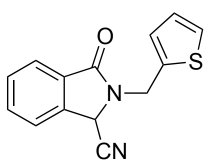

1k

Compound **1l** ***rac*-3-oxo-2-(thiophen-3-ylmethyl)isoindoline-1-carbonitrile**:[49] Light-yellow powder. ^{1}H NMR (400 MHz, $CDCl_3$) δ/ppm: 7.94–7.92 (m, 1H), 7.70–7.60 (m, 3H), 7.35–7.33 (m, 2H), 7.07–7.05 (m, 1H), 5.41 (d, *J* = 15.1 Hz, 1H), 5.13 (s, 1H), 4.43 (d, *J* = 15.1 Hz, 1H).

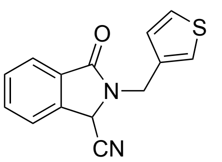

1l

Compound **1m** ***rac*-2-((1*H*-indol-3-yl)methyl)-3-oxoisoindoline-1-carbonitrile**: Prepared from methyl 2-formylbenzoate (512 mg, 435 µL, 3.12 mmol) and (1*H*-indol-3-yl)methanamine (457 mg, 3.12 mmol) following the above general procedure. The crude product was purified by column chromatography (SiO_2, *n*-hexane–EtOAc = 3:1 → 2:1). Off-white powder, yield 660 mg (74%). M.p. 143–145 °C. ^{1}H NMR (400 MHz, $CDCl_3$) δ/ppm: 8.32 (br s, 1H), 7.95–7.92 (m, 1H), 7.69–7.67 (m, 1H), 7.64–7.57 (m, 2H), 7.52–7.50 (m, 1H), 7.42–7.39 (m, 2H), 7.23 (ddd, *J* = 8.1, 7.1, 1.1 Hz, 1H), 7.12 (ddd, *J* = 8.0, 7.1, 1.0 Hz, 1H), 5.70 (d, *J* = 15.1 Hz, 1H), 5.03 (s, 1H), 4.54 (d, *J* = 15.1 Hz, 1H). ^{13}C NMR (100 MHz, $CDCl_3$) δ/ppm: 166.8, 136.8, 136.4, 132.7, 131.4, 130.3, 126.3, 124.7, 124.5, 123.0, 122.9, 120.5, 118.8, 114.9, 111.4, 110.0, 48.7, 36.1. HRMS (APCI) *m/z*: $[M - H]^-$ calcd for $C_{18}H_{12}N_3O$ 286.0986, found 286.0984.

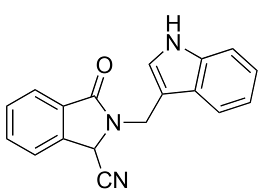

1m

Compound **1n** ***rac*-3-oxo-2-phenylisoindoline-1-carbonitrile**:[47] White powder. ^{1}H NMR (400 MHz, $CDCl_3$) δ/ppm: 8.01–7.99 (m, 1H), 7.79–7.66 (m, 5H), 7.54–7.49 (m, 2H), 7.37–7.32 (m, 1H), 5.91 (s, 1H).

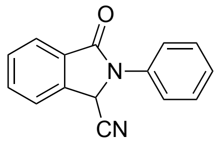

1n

Compound **1o** ***rac*-2-(2-fluorophenyl)-3-oxoisoindoline-1-carbonitrile**: Prepared from methyl 2-formylbenzoate (328 mg, 278 μL, 2 mmol) and 2-fluoroaniline (193 μL, 2 mmol) following the above general procedure. The crude product was triturated with EtOAc–*n*-heptane (1:10) to provide a precipitate, which was filtered off, washed, and dried *in vacuo*. White powder, yield 288 mg (57%). M.p. 129–131 °C. ^{1}H NMR (400 MHz, $CDCl_3$) δ/ppm: 8.02–8.00 (m, 1H), 7.79–7.63 (m, 4H), 7.46–7.41 (m, 1H), 7.34–7.25 (m, 2H), 5.95 (s, 1H). ^{13}C NMR (100 MHz, $CDCl_3$) δ/ppm: 166.3, 157.7 (d, J_{CF} = 250.5 Hz), 137.1, 133.6, 130.6, 130.4, 130.2 (d, J_{CF} = 7.7 Hz), 129.5, 125.2 (d, J_{CF} = 3.9 Hz), 125.1, 123.2, 122.8 (d, J_{CF} = 11.6 Hz), 116.9 (d, J_{CF} = 20.2 Hz), 114.6, 51.7 (d, J_{CF} = 4.8 Hz). ^{19}F NMR (376 MHz, $CDCl_3$) δ/ppm: −120.38 (s, 1F). HRMS (APCI) *m/z*: $[M + H]^+$ calcd for $C_{15}H_{10}FN_2O$ 253.0772, found 253.0770.

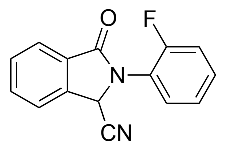

1o

Compound **1p** ***rac*-2-(2-methoxyphenyl)-3-oxoisoindoline-1-carbonitrile**:[47] Prepared from methyl 2-formylbenzoate (328 mg, 278 μL, 2 mmol) and 2-methoxyaniline (226 μL, 2 mmol) following the above general procedure. The crude product was triturated with EtOAc–*n*-heptane (1:10) to provide a precipitate, which was filtered off, washed, and dried *in vacuo*. White powder, yield 380 mg (72%). M.p. 140–141 °C. (lit.[47] m.p. 136–137 °C) ^{1}H NMR (400 MHz, DMSO-d_6) δ/ppm: 7.91–7.82 (m, 3H), 7.75–7.71 (m, 1H), 7.50–7.46 (m, 2H), 7.24 (dd, J = 8.7, 1.2 Hz, 1H), 7.11 (td, J = 7.6, 1.2 Hz, 1H), 6.49 (s, 1H), 3.80 (s, 3H). ^{13}C NMR (100 MHz, DMSO-d_6) δ/ppm: 165.9, 155.4, 137.9, 133.6, 130.5, 130.4, 130.3, 129.8, 124.1, 124.0, 123.7, 116.0, 112.7, 55.9, 51.8. HRMS (APCI) *m/z*: $[M - H]^-$ calcd for $C_{16}H_{11}N_2O_2$ 263.0823, found 263.0821.

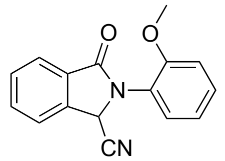

1p

Compound **1q** ***rac*-2-(3-nitrophenyl)-3-oxoisoindoline-1-carbonitrile**:[48] Yellowish powder. ^{1}H NMR (400 MHz, DMSO-d_6) δ/ppm: 8.82 (s, 1H), 8.20–8.16 (m, 1H), 7.98–7.84 (m, 4H), 7.77–7.74 (m, 1H), 7.00 (s, 1H).

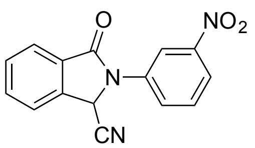

1q

Compound **1r** ***rac*-2-(4-methoxyphenyl)-3-oxoisoindoline-1-carbonitrile**:[47] Colorless powder. ^{1}H NMR (400 MHz, $CDCl_3$) δ/ppm: 7.98–7.96 (m, 1H), 7.76–7.65 (m, 3H), 7.55–7.51 (m, 2H), 7.04–7.00 (m, 2H), 5.81 (s, 1H), 3.86 (s, 3H).

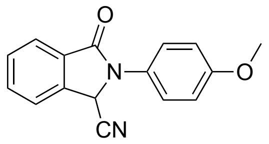

1r

Compound **1s** ***rac*-2-butyl-3-oxoisoindoline-1-carbonitrile**:[48] Light-yellow oil. ^{1}H NMR (400 MHz, $CDCl_3$) δ/ppm: 7.91–7.88 (m, 1H), 7.71–7.59 (m, 3H), 5.36 (s, 1H), 4.00 (ddd, J = 14.3, 8.5, 7.3 Hz, 1H), 3.47 (ddd, J = 14.3, 8.5, 5.6 Hz, 1H), 1.80–1.67 (m, 2H), 1.46–1.37 (m, 2H), 0.99 (t, J = 7.4 Hz, 3H).

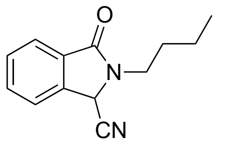

1s

Compound **1t** ***rac*-2-octadecyl-3-oxoisoindoline-1-carbonitrile**:[49] Colorless powder. ^{1}H NMR (300 MHz, $CDCl_3$) δ/ppm: 7.91–7.89 (m, 1H), 7.69–7.59 (m, 3H), 5.36 (s, 1H), 3.98 (ddd, J = 14.3, 7.2, 5.5 Hz, 1H), 3.46 (ddd, J = 14.2, 8.5, 5.7, 1H), 1.80–1.65 (m, 2H), 1.28 (br s, 30H), 0.89 (t, J = 6.7 Hz, 3H).

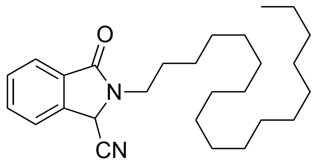

1t

Compound **1u** ***rac*-3-oxo-2-phenethylisoindoline-1-carbonitrile**:[48] White powder. ^{1}H NMR (400 MHz, DMSO-d_6) δ/ppm: 7.86–7.84 (m, 1H), 7.78–7.74 (m, 2H), 7.67–7.63 (m, 1H), 7.32–7.28 (m, 4H), 7.23–7.19 (m, 1H), 6.06 (s, 1H), 4.10–4.03 (m, 1H), 3.61 (ddd, J = 14.2, 8.6, 6.0 Hz, 1H), 3.08–2.93 (m, 2H).

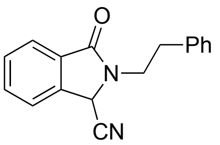

1u

Compound **1v** ***rac*-3-oxo-2-(2,2,2-trifluoroethyl)isoindoline-1-carbonitrile**:[49] Light-yellow powder. ^{1}H NMR (400 MHz, $CDCl_3$) δ/ppm: 7.98–7.96 (m, 1H), 7.79–7.75 (m, 1H), 7.72–7.66 (m, 2H), 5.59 (s, 1H), 4.86 (dq, J = 15.7, 9.4 Hz, 1H), 3.84 (dq, J = 15.7, 8.1 Hz, 1H).

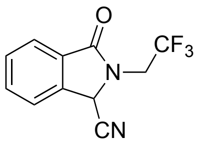

1v

Compound **1w** ***rac*-2-allyl-3-oxoisoindoline-1-carbonitrile**:[47] White powder. ^{1}H NMR (400 MHz, $CDCl_3$) δ/ppm: 7.92–7.90 (m, 1H), 7.72–7.61 (m, 3H), 5.90–5.83 (m, 1H), 5.43–5.35 (m, 3H), 4.80 (ddt, J = 15.5, 4.7, 1.6 Hz, 1H), 3.91 (dd, J = 15.5, 8.0 Hz, 1H).

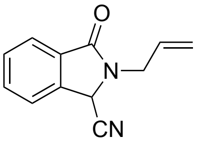

1w

Compound **1x** ***rac*-3-oxo-2-(prop-2-yn-1-yl)isoindoline-1-carbonitrile**: Prepared from methyl 2-formylbenzoate (328 mg, 278 μL, 2 mmol) and propargylamine (128 μL, 2 mmol) the above general procedure. The crude product was purified by column chromatography (SiO_2, *n*-hexane–EtOAc = 5:1→3:1→1:1). White solid, yield 337 mg (86%). M.p. 110–112 °C. ^{1}H NMR (400 MHz, DMSO-d_6) δ/ppm: 7.88–7.86 (m, 1H), 7.83–7.78 (m, 2H), 7.70–7.66 (m, 1H), 6.09 (s, 1H), 4.56 (dd, J = 18.1, 2.5 Hz, H), 4.36 (dd, J = 18.1, 2.5 Hz, 1H), 3.46 (t, J = 2.5 Hz, 1H). ^{13}C NMR (100 MHz, DMSO-d_6) δ/ppm: 166.2, 138.0, 133.5, 130.4, 130.0, 124.0, 123.8, 115.8, 77.7, 76.1, 49.5, 31.0. HRMS (APCI) m/z: $[M + H]^+$ calcd for $C_{12}H_9N_2O$ 197.0709, found 197.0709.

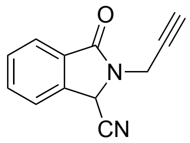

1x

Compound **1y** ***rac*-*tert*-butyl (2-(1-cyano-3-oxoisoindolin-2-yl)ethyl)carbamate**: Prepared from methyl 2-formylbenzoate (328 mg, 278 μL, 2 mmol) and *tert*-butyl (2-aminoethyl)carbamate (320 μL, 2 mmol) following the above general procedure. The crude product was purified by column chromatography (SiO_2, *n*-hexane–EtOAc = 3:1). White powder, yield 250 mg (42%). M.p. 129–130 °C. ^{1}H NMR (400 MHz, $CDCl_3$) δ/ppm: 7.86 (d, J = 7.6 Hz, 1H), 7.68–7.65 (m, 2H), 7.60–7.57 (m, 1H), 5.85 (s, 1H), 4.88 (br s, 1H), 4.28–4.21 (m, 1H), 3.75–3.67 (m, 1H), 3.45–3.41 (m, 1H), 3.37–3.23 (m, 1H), 1.17 (s, 9H). ^{13}C NMR (100 MHz, $CDCl_3$) δ/ppm: 167.9, 156.4, 137.3, 132.9, 130.9, 130.1, 124.3, 123.1, 114.9, 79.7, 49.5, 41.4, 38.2, 27.9. HRMS (APCI) m/z: $[M - H]^-$ calcd for $C_{16}H_{18}N_3O_3$ 300.1354, found 300.1352.

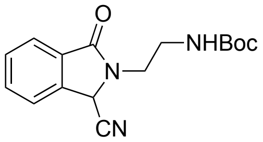

1y

Compound **1z** ***rac*-methyl 3-(1-cyano-3-oxoisoindolin-2-yl)propanoate**: Prepared from methyl 2-formylbenzoate (328 mg, 278 μL, 2 mmol) and methyl 3-aminopropanoate (206 mg, 2 mmol) following the above general procedure. The crude product was purified by column chromatography (SiO_2, *n*-hexane–EtOAc = 3:1). Yellow oil, which partially solidified upon standing, yield 224 mg (46%). ^{1}H NMR (400 MHz, $CDCl_3$) δ/ppm: 7.90–7.87 (m, 1H), 7.71–7.59 (m, 3H), 5.69 (s, 1H), 4.23–

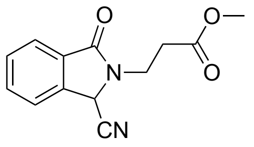

1z

4.16 (m, 1H), 3.81–3.74 (m, 1H), 3.71 (s, 3H), 2.96–2.88 (m, 1H), 2.79–2.72 (m, 1H). ^{13}C NMR (100 MHz, $CDCl_3$) δ/ppm: 172.4, 167.4, 137.3, 133.0, 131.1, 130.3, 124.3, 123.1, 115.0, 52.1, 51.1, 37.5, 32.8. HRMS (APCI) *m/z*: $[M - H]^-$ calcd for $C_{13}H_{11}N_2O_3$ 243.0775, found 243.0772.

Compound **1za** ***rac*-2-benzyl-7-methyl-3-oxoisoindoline-1-carbonitrile**: Prepared from methyl 2-formyl-3-methylbenzoate (160 mg, 0.9 mmol) and benzylamine (100 μL, 0.9 mmol) following the above general procedure. The crude product was purified by column chromatography (SiO_2, *n*-hexane–EtOAc = 20:1 → 9:1 → 5:1 → 2:1). White powder, yield 85 mg (36%). M.p. 88–89 °C. ^{1}H NMR (400 MHz, $CDCl_3$) δ/ppm: 7.79–7.78 (m, 1H), 7.55–7.51 (m, 1H), 7.45–7.43 (m, 1H), 7.41–7.35 (m, 5H), 5.56 (d, J = 15.0 Hz, 1H), 4.97 (s, 1H), 4.30 (d, J = 15.0 Hz, 1H), 2.46 (s, 3H). ^{13}C NMR (100 MHz, $CDCl_3$) δ/ppm: 167.3, 135.2, 134.8, 134.1, 133.5, 131.1, 130.6, 129.2, 128.6, 128.4, 122.2, 113.6, 48.1, 44.8, 17.5. HRMS (APCI) *m/z*: $[M + H]^+$ calcd for $C_{17}H_{15}N_2O$ 263.1179, found 263.1177.

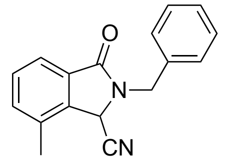

1za

Compound **1zb** ***rac*-2-benzyl-6-bromo-3-oxoisoindoline-1-carbonitrile**: Prepared from methyl 4-bromo-2-formylbenzoate (486 mg, 2 mmol) and benzylamine (220 μL, 2 mmol) following the above general procedure. The crude product was triturated with EtOAc–*n*-heptane (1:10) to provide a precipitate, which was filtered off, washed, and dried *in vacuo*. White powder, yield 570 mg (88%). M.p. 153–154 °C. ^{1}H NMR (400 MHz, $CDCl_3$) δ/ppm: 7.81–7.75 (m, 3H), 7.41–7.33 (m, 5H), 5.48 (d, J = 15.1 Hz, 1H), 5.06 (s, 1H), 4.29 (d, J = 15.1 Hz, 1H). ^{13}C NMR (100 MHz, $CDCl_3$) δ/ppm: 165.9, 138.3, 134.8, 134.0, 130.1, 129.2, 128.6, 127.6, 126.6, 126.0, 113.9, 48.3, 45.1. HRMS (APCI) *m/z*: $[M - H]^-$ calcd for $C_{16}H_{10}BrN_2O$ 324.9982, found 324.9981.

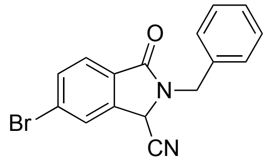

1zb

Compound **1zc** ***rac*-5-chloro-2-(4-methoxybenzyl)-3-oxoisoindoline-1-carbonitrile**:[49] Colorless powder. ^{1}H NMR (400 MHz, $CDCl_3$) δ/ppm: 7.90 (d, J = 1.9 Hz, 1H), 7.63 (dd, J = 8.2, 1.9 Hz, 1H), 7.52 (d, J = 8.2 Hz, 1H), 7.31–7.28 (m, 2H), 6.92–6.88 (m, 2H), 5.43 (d, J = 14.9 Hz, 1H), 5.04 (s, 1H), 4.24 (d, J = 14.9 Hz, 1H), 3.81 (s, 3H).

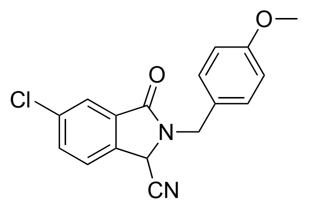

1zc

Compound **1zd** ***rac*-2-benzyl-4-chloro-3-oxoisoindoline-1-carbonitrile**: Prepared from methyl 2-chloro-6-formylbenzoate (150 mg, 0.76 mmol) and benzylamine (83 μL, 0.76 mmol) following the above general procedure. The crude product was purified by column chromatography (SiO_2, *n*-hexane–EtOAc = 3:1). Glassy solid, yield 133 mg (62%). ^1H NMR (400 MHz, $CDCl_3$) δ/ppm: 7.61–7.55 (m, 2H), 7.51–7.48 (m, 1H), 7.39–7.35 (m, 5H), 5.51 (d, *J* = 15.0 Hz, 1H), 5.06 (s, 1H), 4.28 (d, *J* = 15.0 Hz, 1H). ^{13}C NMR (100 MHz, $CDCl_3$) δ/ppm: 164.5, 138.9, 134.9, 133.6, 132.6, 132.2, 129.2, 128.7, 128.5, 127.2, 121.7, 114.1, 48.0, 45.0. HRMS (APCI) *m/z*: $[M + H]^+$ calcd for $C_{16}H_{12}ClN_2O$ 283.0633, found 283.0635.

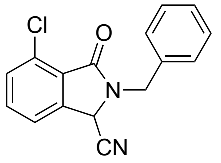

1zd

Compound **1ze** ***rac*-2-benzyl-5,6-dimethoxy-3-oxoisoindoline-1-carbonitrile**: Prepared from methyl 2-formyl-4,5-dimethoxybenzoate (224 mg, 1 mmol) and benzylamine (110 μL, 1 mmol) following the above general procedure. The crude product was purified by column chromatography (SiO_2, *n*-hexane–EtOAc = 3:1). White powder, yield 165 mg (54%). M.p. 99–101 °C. ^1H NMR (400 MHz, $CDCl_3$) δ/ppm: 7.39–7.33 (m, 6H), 7.01 (s, 1H), 5.46 (d, *J* = 15.1 Hz, 1H), 5.00 (s, 1H), 4.29 (d, *J* = 15.1 Hz, 1H), 3.97 (s, 3H), 3.96 (s, 3H). ^{13}C NMR (100 MHz, $CDCl_3$) δ/ppm: 167.3, 153.7, 151.4, 135.5, 130.2, 129.1, 128.5, 128.3, 123.6, 114.8, 105.8, 104.9, 56.5, 56.4, 48.5, 45.1. HRMS (APCI) *m/z*: $[M - H]^-$ calcd for $C_{18}H_{15}N_2O_3$ 307.1088, found 307.1087.

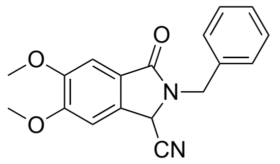

1ze

Compound **1zf** ***rac*-ethyl 2-benzyl-3-oxoisoindoline-1-carboxylate**:[50] Yellowish viscous oil. ^1H NMR (400 MHz, $CDCl_3$) δ/ppm: 7.92–7.89 (m, 1H), 7.56–7.50 (m, 3H), 7.36–7.26 (m, 5H), 5.50 (d, *J* = 14.9 Hz, 1H), 4.91 (s, 1H), 4.29 (d, *J* = 14.9 Hz, 1H), 4.29–4.16 (m, 2H), 1.29 (t, *J* = 7.1 Hz. 3H).

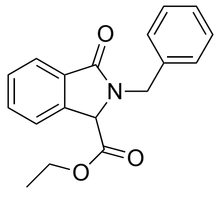

1zf

Compound **1zg** ***rac*-2,2,2-trifluoroethyl 2-benzyl-3-oxoisoindoline-1-carboxylate**: A mixture of 2-benzyl-3-oxoisoindoline-1-carboxylic acid[50a] (1.0 g, 3.7 mmol), 2,2,2-trifluoroethanol (10 mL), and concentrated sulfuric acid (0.1 mL) was refluxed for 5 days (oil bath). After that, the volatiles were evaporated, and the residue was dissolved in CH_2Cl_2. The organic phase was washed with a saturated aqueous solution of $NaHCO_3$ and brine, dried over anhydrous Na_2SO_4, filtered, and evaporated *in vacuo*. The crude product was purified by column chromatography (SiO_2, *n*-hexane–EtOAc 3:1). White solid, yield 580 mg (45%). M.p. 95–96 °C. ^1H NMR (400 MHz, $CDCl_3$) δ/ppm: 7.93–7.91 (m, 1H), 7.58–7.52 (m, 3H), 7.36–7.24 (m, 5H), 5.52 (d, *J* = 15.0 Hz, 1H), 5.00 (s, 1H), 4.66–4.57 (m, 1H), 4.53–4.43

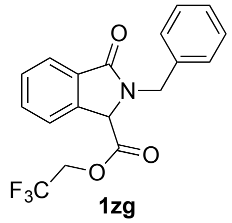

1zg

(m, 1H), 4.25 (d, J = 15.0 Hz, 1H). ^{13}C NMR (100 MHz, $CDCl_3$) δ/ppm: 168.2, 166.8, 138.2, 135.9, 132.2, 131.6, 129.7, 129.0, 128.5, 128.1, 124.4, 122.8, 122.5 (q, $^1J_{CF}$ = 277.4 Hz), 61.1 (q, $^2J_{CF}$ = 37.1 Hz), 60.6, 45.1. ^{19}F NMR (376 MHz, $CDCl_3$) δ/ppm: −73.44 (t, J_{HF} = 7.9 Hz, 3F). HRMS (APCI) *m/z*: $[M + H]^+$ calcd for $C_{18}H_{15}F_3NO_3$ 350.0999, found 350.0998.

Compound **1zh** ***rac*-2,2,2-trichloroethyl 2-benzyl-3-oxoisoindoline-1-carboxylate**: Prepared according to the procedure described for compound **1zg** from 2-benzyl-3-oxoisoindoline-1-carboxylic acid (1.0 g, 3.7 mmol), 2,2,2-trichloroethanol (10 mL), and concentrated sulfuric acid (0.1 mL). The crude product was purified by column chromatography (SiO_2, *n*-hexane–EtOAc 3:1). White solid, yield 855 mg (58%). M.p. 128–130 °C. ^{1}H NMR (400 MHz, $CDCl_3$) δ/ppm: 7.93–7.91 (m, 1H), 7.66–7.64 (m, 1H), 7.58–7.53 (m, 2H), 7.35–7.27 (m, 5H), 5.54 (d, J = 15.0 Hz, 1H), 5.05 (s, 1H), 4.90 (d, J = 11.8 Hz, 1H), 4.73 (d, J = 11.8 Hz, 1H), 4.33 (d, J = 15.0 Hz, 1H). ^{13}C NMR (100 MHz, $CDCl_3$) δ/ppm: 168.3, 166.7, 138.4, 136.1, 132.1, 131.7, 129.7, 129.0, 128.6, 128.0, 124.3, 123.3, 94.2, 74.6, 60.8, 45.1. HRMS (APCI) *m/z*: $[M - H]^-$ calcd for $C_{18}H_{13}Cl_3NO_3$ 395.9966, found 395.9961.

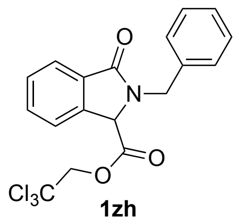

1zh

3.4 Syntheses of Racemic Products

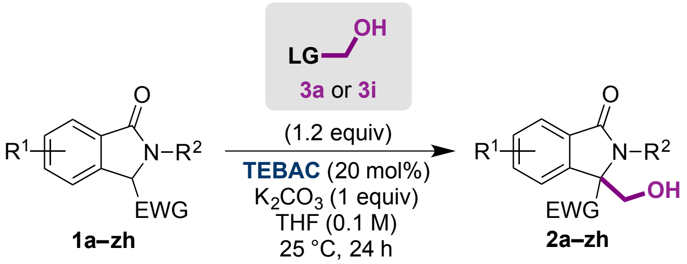

A mixture of isoindolinone derivative **1** (0.4 mmol), anhydrous K_2CO_3 (55 mg, 0.4 mmol), benzyltriethylammonium chloride (TEBAC, 18 mg, 0.08 mmol), and formaldehyde surrogate **3a** (62 mg, 0.48 mmol) or **3i** (103 mg, 0.48 mmol) in dry THF (4 mL) was stirred at rt for 24 h under Ar. Then water (10 mL) and EtOAc (10 mL) were added, and the aqueous phase was repetitively extracted with EtOAc. The combined organic phase was washed with brine, dried over anhydrous Na_2SO_4, filtered, and evaporated *in vacuo*. The residue was subjected to column chromatography (SiO_2, *n*-hexane–EtOAc = 2:1 → 1:1, or CH_2Cl_2–MeOH = 99:1) as specified below for each entry.

3.5 Syntheses of Enantioenriched Products

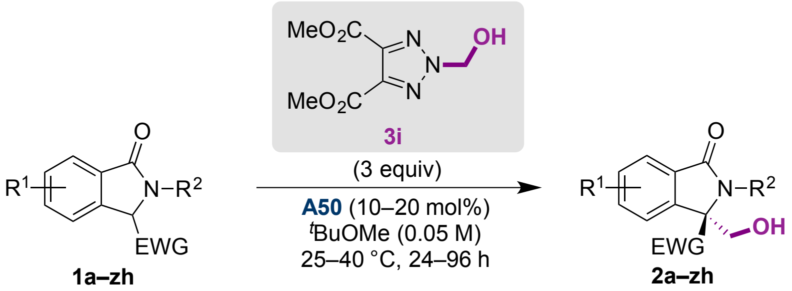

The corresponding isoindolinone derivative **1** (0.05 mmol), formaldehyde surrogate **3i** (32.3 mg, 0.15 mmol), and the catalyst **A50** (2.3 or 4.5 mg, 0.005 or 0.01 mmol, as specified below for each entry) were dissolved in dry *tert*-butyl methyl ether (1.0 mL) in a 4-mL screw-cap vial. The reaction mass was stirred under the conditions specified below for each entry (time, temperature). The temperature was maintained using a thermostatic circulating water bath (**Fig. S19**). Then, the solvent was evaporated *in vacuo*. The crude residue was purified by column chromatography (SiO_2, CH_2Cl_2–MeOH = 99:1).

Stereochemical assignment: The absolute configuration of (*R*)-**2v** was elucidated by a single-crystal X-ray diffraction measurement. The absolute configurations of the remaining adducts were assigned by analogy thereto.

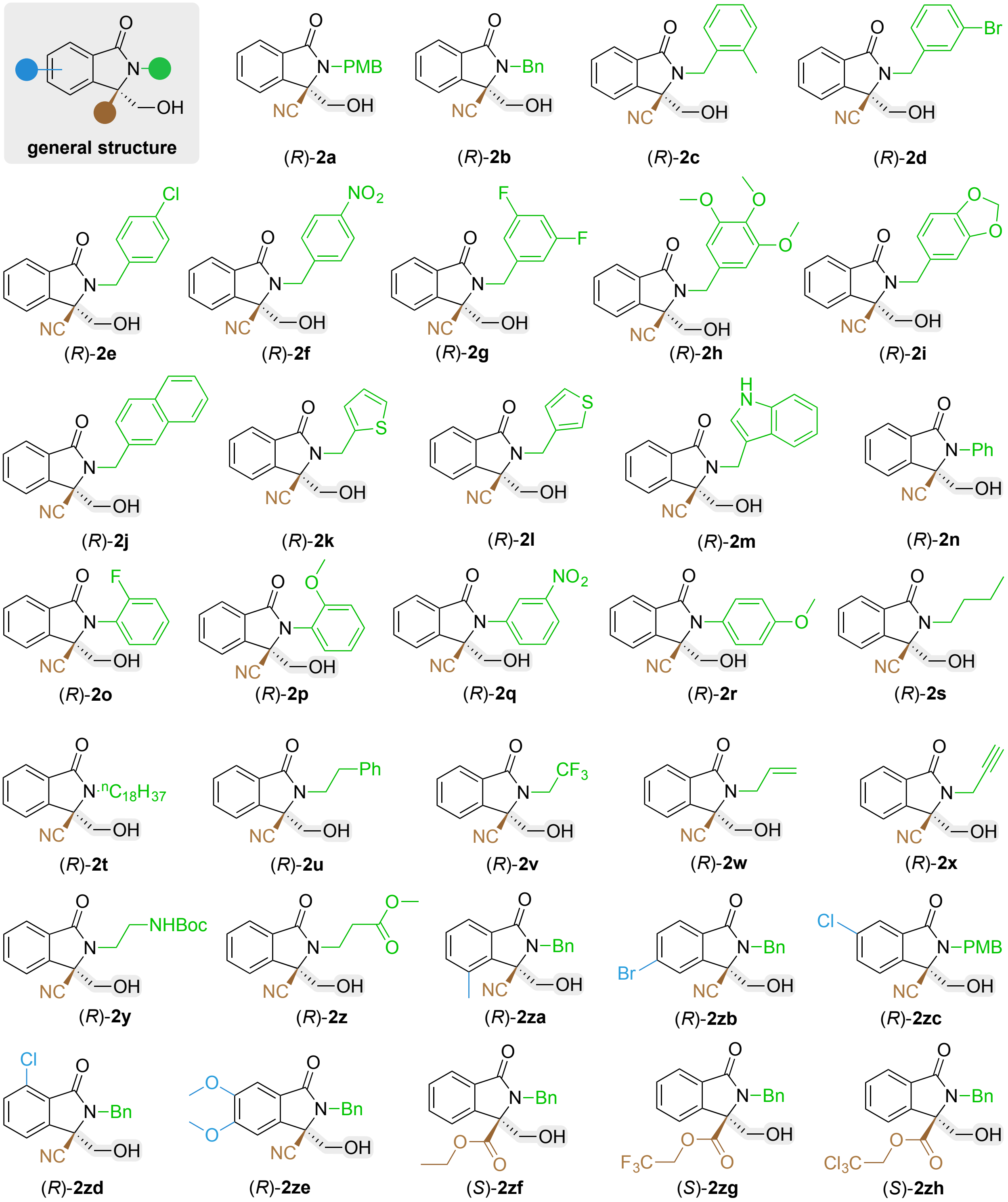

Figure S18: Numbering of the products.

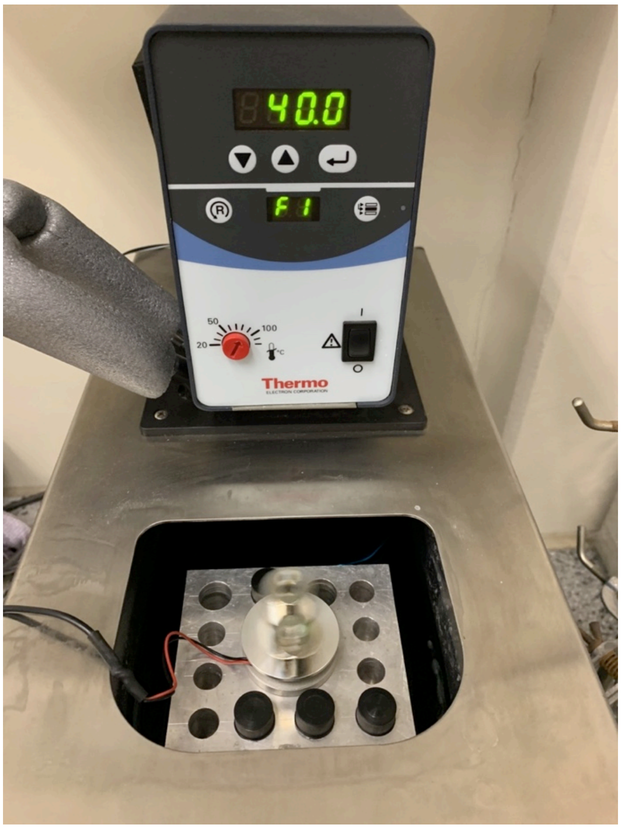

Figure S19: A typical reaction setup for small-scale catalytic experiments. As described in our previous report,[1] the custom-made stirred aluminum block was immersed in a thermostatic circulating water bath Cool Tech 320 Chiller (Thermo Scientific).

Analytical data for the prepared compounds are listed below:

Compound **2a** **(*R*)-1-(hydroxymethyl)-2-(4-methoxybenzyl)-3-oxoisoindoline-1-carbonitrile**: Prepared following the general procedure using 10 mol% (2.3 mg) of **A50** (24 h, 40 °C). The crude product was purified by column chromatography (SiO_2, CH_2Cl_2–MeOH = 99:1) and obtained as a white solid (14.8 mg, 96%). E.r. = 93:7. M.p. 130–132 °C. $[\alpha]_D^{25}$ −45 (*c* 1.0, $CHCl_3$). ^1H NMR (300 MHz, $CDCl_3$) δ/ppm: 7.97–7.93 (m, 1H), 7.73–7.61 (m, 3H), 7.44–7.39 (m, 2H), 6.93–6.88 (m, 2H), 5.36 (d, *J* = 15.4 Hz, 1H), 4.41 (d, *J* = 15.4 Hz, 1H), 3.92 (dd, *J* = 11.9, 8.2 Hz, 1H), 3.81 (s, 3H), 3.77 (dd, *J* = 11.9, 6.5 Hz, 1H), 1.43 (dd, *J* = 8.2, 6.5 Hz, 1H). ^{13}C NMR (125 MHz, $CDCl_3$) δ/ppm: 168.0, 159.6, 139.8, 133.2, 130.9, 130.6, 129.4, 128.7, 124.5, 122.5, 115.6, 114.6, 65.3, 63.8, 55.3, 44.1. HRMS (ESI-Q-TOF) *m/z*: $[M + Na]^+$ calcd for $C_{18}H_{16}N_2O_3Na$ 331.1053, found 331.1020. HPLC (a Hypersil silica column (3 µm, 100 × 4.6 mm) used as a precolumn, which was connected *via* the standard blue PEEK capillary tubing (L 300 mm, ID 0.01", OD 1/16") to a Daicel® Chiralpak IB (5 µm, 250 × 4.6 mm), *n*-heptane–*i*-PrOH = 70:30, flow rate = 0.5 mL/min, λ = 230 nm, 25 °C) t_R = 16.05 min (major), 17.51 min (minor).

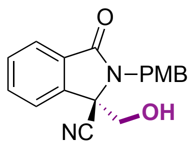

2a

Compound **2b** **(*R*)-1-(hydroxymethyl)-2-benzyl-3-oxoisoindoline-1-carbonitrile**: Prepared following the general procedure using 10 mol% (2.3 mg) of **A50** (24 h, 40 °C). The crude product was purified by column chromatography (SiO_2, CH_2Cl_2–MeOH = 99:1) and obtained as a colorless waxy solid (12.3 mg, 88%). E.r. = 94.5:5.5. $[\alpha]_D^{25}$ −66 (*c* 0.5, $CHCl_3$). ^{1}H NMR (400 MHz, $CDCl_3$) δ/ppm: 7.93–7.91 (m, 1H), 7.70–7.67 (m, 2H), 7.65–7.59 (m, 1H), 7.46–7.44 (m, 2H), 7.40–7.32 (m, 3H), 5.30 (d, *J* = 15.6 Hz, 1H), 4.56 (d, *J* = 15.6 Hz, 1H), 3.93 (d, *J* = 11.8 Hz, 1H), 3.77 (d, *J* = 11.8 Hz, 1H), 2.00 (br s, 1H). ^{13}C NMR (100 MHz, $CDCl_3$) δ/ppm: 168.1, 139.9, 136.6, 133.2, 130.7, 130.6, 129.2, 128.4, 128.0, 124.5, 122.6, 115.5, 65.3, 63.7, 44.7. HRMS (APCI) *m/z*: $[M + H]^+$ calcd for $C_{17}H_{15}N_2O_2$ 279.1128, found 279.1130. HPLC (a Hypersil silica column (3 µm, 100 × 4.6 mm) used as a precolumn, which was connected *via* the standard blue PEEK capillary tubing (L 300 mm, ID 0.01", OD 1/16") to a Daicel® Chiralpak IA (5 µm, 250 × 4.6 mm), *n*-heptane–*i*-PrOH = 90:10, flow rate = 0.5 mL/min, λ = 230 nm, 25 °C) t_R = 37.13 min (major), 39.82 min (minor).

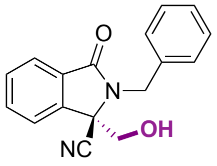

2b

Compound **2c** **(*R*)-1-(hydroxymethyl)-2-(2-methylbenzyl)-3-oxoisoindoline-1-carbonitrile**: Prepared following the general procedure using 10 mol% (2.3 mg) of **A50** (24 h, 40 °C). The crude product was purified by column chromatography (SiO_2, CH_2Cl_2–MeOH = 99:1) and obtained as a viscous oil (14.0 mg, 96%). E.r. = 92.5:7.5. $[\alpha]_D^{25}$ −60 (*c* 1.0, $CHCl_3$). ^{1}H NMR (400 MHz, $CDCl_3$) δ/ppm: 7.94–7.92 (m, 1H), 7.69–7.68 (m, 2H), 7.64–7.60 (m, 1H), 7.28–7.24 (m, 3H), 7.15–7.14 (m, 1H), 5.30 (d, *J* = 15.6 Hz, 1H), 4.47 (d, *J* = 15.6 Hz, 1H), 3.94–3.91 (m, 1H), 3.77 (d, *J* = 11.8 Hz, 1H), 2.35 (s, 3H), 1.84 (br s, 1H). ^{13}C NMR (100 MHz, $CDCl_3$) δ/ppm: 168.1, 140.0, 139.1, 136.6, 133.2, 130.8, 130.6, 129.2, 129.1, 128.6, 124.9, 124.5, 122.6, 115.6, 65.3, 63.8, 44.6, 21.4. HRMS (APCI) *m/z*: $[M + H]^+$ calcd for $C_{18}H_{17}N_2O_2$ 293.1285, found 293.1288. HPLC (a Hypersil silica column (3 µm, 100 × 4.6 mm) used as a precolumn, which was connected *via* the standard blue PEEK capillary tubing (L 300 mm, ID 0.01", OD 1/16") to a Daicel® Chiralpak IA (5 µm, 250 × 4.6 mm), *n*-heptane–*i*-PrOH = 90:10, flow rate = 0.5 mL/min, λ = 230 nm, 25 °C) t_R = 30.79 min (major), 33.53 min (minor).

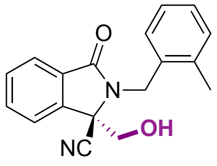

2c

Compound **2d** **(*R*)-2-(3-bromobenzyl)-1-(hydroxymethyl)-3-oxoisoindoline-1-carbonitrile**: Prepared following the general procedure using 10 mol% (2.3 mg) of **A50** (24 h, 40 °C). The crude product was purified by column chromatography (SiO_2, CH_2Cl_2–MeOH = 99:1) and obtained as a glassy solid (17.0 mg, 95%). E.r. = 93.5:6.5. $[\alpha]_D^{25}$ −28 (*c* 1.0, $CHCl_3$). ^{1}H NMR (400 MHz, $CDCl_3$) δ/ppm: 7.93–7.91 (m, 1H), 7.71–7.70 (m, 2H), 7.67–7.62 (m, 1H), 7.59 (br s, 1H), 7.47–7.45 (m, 1H), 7.38–7.36 (m, 1H), 7.25–7.21 (m, 1H), 5.15 (d, *J* = 15.8 Hz, 1H), 4.63 (d, *J* = 15.8 Hz, 1H), 3.97 (d, *J* = 11.2 Hz, 1H), 3.83 (d, *J* = 11.2 Hz, 1H), 2.27 (br s, 1H). ^{13}C NMR (100 MHz, $CDCl_3$) δ/ppm: 168.1, 139.8, 138.8, 133.4, 131.4, 131.0, 130.8, 130.5, 130.4, 126.6, 124.7, 123.0, 122.8,

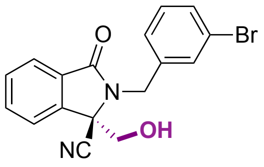

2d

115.5, 65.7, 63.5, 44.3. HRMS (APCI) *m/z*: $[M + H]^+$ calcd for $C_{17}H_{14}BrN_2O_2$ 357.0233, found 357.0236. HPLC (a Hypersil silica column (3 µm, 100 × 4.6 mm) used as a precolumn, which was connected *via* the standard blue PEEK capillary tubing (L 300 mm, ID 0.01”, OD 1/16”) to a Daicel® Chiralpak IA (5 µm, 250 × 4.6 mm), *n*-heptane–*i*-PrOH = 90:10, flow rate = 0.5 mL/min, λ = 230 nm, 25 °C) t_R = 35.78 min (major), 39.23 min (minor).

Compound **2e** **(*R*)-2-(4-chlorobenzyl)-1-(hydroxymethyl)-3-oxoisoindoline-1-carbonitrile**: Prepared following the general procedure using 10 mol% (2.3 mg) of **A50** (24 h, 40 °C). The crude product was purified by column chromatography (SiO_2, CH_2Cl_2–MeOH = 99:1) and obtained as a white solid (13.1 mg, 84%). E.r. = 91:9. M.p. 153–156 °C. $[\alpha]_D^{25}$ −33 (*c* 0.5, $CHCl_3$). ^{1}H NMR (400 MHz, $CDCl_3$) δ/ppm: 7.93–7.91 (m, 1H), 7.71–7.69 (m, 2H), 7.65–7.61 (m, 1H), 7.40–7.38 (m, 2H), 7.34–7.32 (m, 2H), 5.16 (d, *J* = 15.7 Hz, 1H), 4.62 (d, *J* = 15.7 Hz, 1H), 3.96 (dd, *J* = 11.5, 4.8 Hz, 1H), 3.81 (dd, *J* = 11.5, 3.3 Hz, 1H), 2.14 (br s, 1H). ^{13}C NMR (100 MHz, $CDCl_3$) δ/ppm: 168.1, 139.8, 135.1, 134.2, 133.4, 130.8, 130.5, 129.4, 129.2, 124.6, 122.7, 115.5, 65.6, 63.5, 44.2. HRMS (APCI) *m/z*: $[M + H]^+$ calcd for $C_{17}H_{14}ClN_2O_2$ 313.0738, found 313.0738. HPLC (a Hypersil silica column (3 µm, 100 × 4.6 mm) used as a precolumn, which was connected *via* the standard blue PEEK capillary tubing (L 300 mm, ID 0.01”, OD 1/16”) to a Daicel® Chiralpak IA (5 µm, 250 × 4.6 mm), *n*-heptane–*i*-PrOH = 80:20, flow rate = 0.5 mL/min, λ = 230 nm, 25 °C) t_R = 18.78 min (major), 21.24 min (minor).

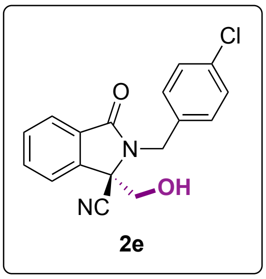

Compound **2f** **(*R*)-1-(hydroxymethyl)-2-(4-nitrobenzyl)-3-oxoisoindoline-1-carbonitrile**: Prepared following the general procedure using 10 mol% (2.3 mg) of **A50** (24 h, 40 °C). The crude product was purified by column chromatography (SiO_2, CH_2Cl_2–MeOH = 99:1) and obtained as a yellowish waxy solid (14.2 mg, 88%). E.r. = 89:11. $[\alpha]_D^{25}$ −19 (*c* 1.0, $CHCl_3$). ^{1}H NMR (400 MHz, DMSO-d_6) δ/ppm: 8.21–8.19 (m, 2H), 7.94–7.92 (m, 1H), 7.86–7.84 (m, 1H), 7.82–7.78 (m, 1H), 7.72–7.68 (m, 1H), 7.66–7.64 (m, 2H), 5.88 (t, *J* = 5.6 Hz, 1H), 5.07. 4.92 (q, AB, J_{AB} = 16.7 Hz, 2H), 4.09 (d, *J* = 5.6 Hz, 1H). ^{13}C NMR (100 MHz, DMSO-d_6) δ/ppm: 167.2, 146.7, 144.8, 140.6, 133.3, 130.5, 130.0, 128.7, 123.5, 123.4, 123.3, 116.4, 63.5, 62.5, 43.3. HRMS (APCI) *m/z*: $[M + H]^+$ calcd for $C_{17}H_{14}N_3O_4$ 324.0979, found 324.0980. HPLC (a Hypersil silica column (3 µm, 100 × 4.6 mm) used as a precolumn, which was connected *via* the standard blue PEEK capillary tubing (L 300 mm, ID 0.01”, OD 1/16”) to a Daicel® Chiralpak IA (5 µm, 250 × 4.6 mm), *n*-heptane–*i*-PrOH = 80:20, flow rate = 0.5 mL/min, λ = 230 nm, 25 °C) t_R = 30.59 min (major), 33.95 min (minor).

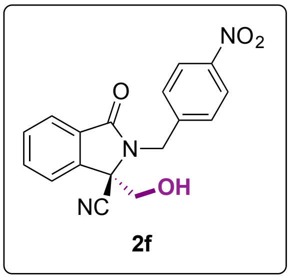

Compound **2g** **(*R*)-2-(3,5-difluorobenzyl)-1-(hydroxymethyl)-3-oxoisoindoline-1-carbonitrile:** Prepared following the general procedure using 10 mol% (2.3 mg) of **A50** (24 h, 40 °C). The crude product was purified by column chromatography (SiO_2, CH_2Cl_2–MeOH = 99:1) and obtained as a colorless waxy solid (12.0 mg, 76%). E.r. = 90:10. $[\alpha]_D^{25}$ −26 (*c* 0.5, $CHCl_3$). ^{1}H NMR (400 MHz, DMSO-d_6) δ/ppm: 7.93–7.91 (m, 1H), 7.84 (dt, *J* = 7.5, 0.9 Hz, 1H), 7.79 (dt, *J* = 7.5, 1.2 Hz, 1H), 7.69 (dt, *J* = 7.5, 1.0 Hz, 1H), 7.18–7.09 (m, 3H), 5.88 (t, *J* = 5.8 Hz, 1H), 4.98 (d, *J* = 16.4 Hz, 1H), 4.75 (d, *J* = 16.4 Hz, 1H), 4.11 (d, *J* = 5.8 Hz, 2H). ^{13}C NMR (100 MHz, DMSO-d_6) δ/ppm: 167.3, 162.4 (dd, J_{CF} = 246.1, 13.0 Hz), 141.6 (app t, J_{CF} = 8.7 Hz), 140.7, 133.3, 130.5, 130.2, 123.5 (d, J_{CF} = 24.1 Hz), 116.5, 110.9 (dd, J_{CF} = 18.3, 6.7 Hz), 102.9 (app t, J_{CF} = 25.5 Hz), 63.2, 62.5, 43.0. ^{19}F NMR (376 MHz, DMSO-d_6) δ/ppm: −109.83 (t, *J* = 7.2 Hz, 2F). HRMS (APCI) *m/z*: $[M + H]^+$ calcd for $C_{17}H_{13}F_2N_2O_2$ 315.0940, found 315.0939. HPLC (a Hypersil silica column (3 µm, 100 × 4.6 mm) used as a precolumn, which was connected *via* the standard blue PEEK capillary tubing (L 300 mm, ID 0.01", OD 1/16") to a Daicel® Chiralpak IA (5 µm, 250 × 4.6 mm), *n*-heptane–*i*-PrOH = 90:10, flow rate = 0.5 mL/min, λ = 230 nm, 25 °C) t_R = 31.85 min (major), 36.96 min (minor).

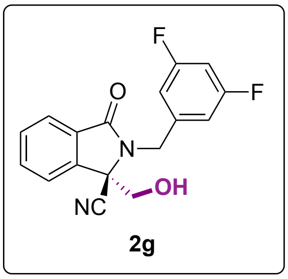

Compound **2h** **(*R*)-1-(hydroxymethyl)-3-oxo-2-(3,4,5-trimethoxybenzyl)isoindoline-1-carbonitrile**: Prepared following the general procedure using 10 mol% (2.3 mg) of **A50** (24 h, 40 °C). The crude product was purified by column chromatography (SiO_2, CH_2Cl_2–MeOH = 99:1) and obtained as a colorless waxy solid (12.0 mg, 65%). E.r. = 90.5:9.5. $[\alpha]_D^{25}$ −42 (*c* 1.0, $CHCl_3$). ^{1}H NMR (400 MHz, DMSO-d_6) δ/ppm: 7.91–7.90 (m, 1H), 7.84–7.82 (m, 1H), 7.80–7.76 (m, 1H), 7.70–7.66 (m, 1H), 6.73 (s, 2H), 5.87 (t, *J* = 5.7 Hz, 1H), 4.87 (d, *J* = 15.7 Hz, 1H), 4.63 (d, *J* = 15.4 Hz, 1H), 4.12 (dd, *J* = 11.3, 6.0 Hz, 1H), 4.00 (dd, *J* = 11.3, 5.5 Hz, 1H), 3.73 (s, 6H), 3.63 (s, 3H). ^{13}C NMR (100 MHz, DMSO-d_6) δ/ppm: 167.2, 152.7, 141.0, 136.8, 133.2, 132.3, 130.4, 130.3, 123.5, 123.4, 116.7, 105.5, 63.2, 62.5, 60.0, 55.8, 43.9. HRMS (ESI-Q-TOF) *m/z*: $[M + Na]^+$ calcd for $C_{20}H_{20}N_2O_5Na$ 391.1264, found 391.1225. HPLC (a Hypersil silica column (3 µm, 100 × 4.6 mm) used as a precolumn, which was connected *via* the standard blue PEEK capillary tubing (L 300 mm, ID 0.01", OD 1/16") to a Daicel® Chiralpak IA (5 µm, 250 × 4.6 mm), *n*-heptane–*i*-PrOH = 80:20, flow rate = 0.5 mL/min, λ = 230 nm, 25 °C) t_R = 32.30 min (minor), 59.65 min (major).

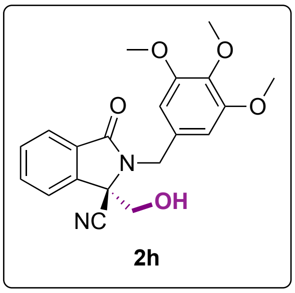

Compound **2i** **(*R*)-(2-(benzo[*d*][1,3]dioxol-5-ylmethyl)-1-(hydroxymethyl)-3-oxoisoindoline-1-carbonitrile)**: Prepared following the general procedure using 10 mol% (2.3 mg) of **A50** (24 h, 40 °C). The crude product was purified by column chromatography (SiO_2, CH_2Cl_2–MeOH = 99:1) and obtained as a colorless waxy solid (14.9 mg, 92%). E.r. = 91.5:8.5. $[\alpha]_D^{25}$ −21 (*c* 1.0, $CHCl_3$). ^1H NMR (400 MHz, DMSO-d_6) δ/ppm: 8.00–7.98 (m, 1H), 7.88–7.83 (m, 2H), 7.75–7.71 (m, 1H), 7.55–7.51 (m, 1H), 7.45–7.44 (m, 2H), 7.36–7.34 (m, 1H), 5.89 (t, *J* = 5.7 Hz, 1H), 5.36 (t, *J* = 5.7 Hz, 1H), 4.58 (d, *J* = 5.7 Hz, 2H), 4.17 (dd, *J* = 11.2, 5.7 Hz, 1H), 3.77 (dd, *J* = 11.2, 5.7 Hz, 1H). ^{13}C NMR (100 MHz, DMSO-d_6) δ/ppm: 166.7, 144.4, 140.7, 134.6, 133.5, 130.6, 130.4, 129.3, 127.0, 126.8, 126.5, 123.8, 123.6, 117.0, 64.2, 62.9, 62.4. HRMS (APCI) *m/z*: $[M + H]^+$ calcd for $C_{18}H_{15}N_2O_4$ 323.1026, found 323.1028. HPLC (a Hypersil silica column (3 µm, 100 × 4.6 mm) used as a precolumn, which was connected *via* the standard blue PEEK capillary tubing (L 300 mm, ID 0.01”, OD 1/16”) to a Daicel® Chiralpak IB (5 µm, 250 × 4.6 mm), *n*-heptane–*i*-PrOH = 80:20, flow rate = 0.5 mL/min, λ = 230 nm, 25 °C) t_R = 24.60 min (major), 42.99 min (minor).

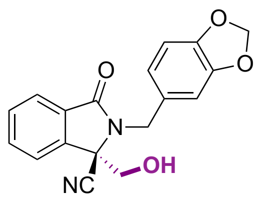

2i

Compound **2j** **(*R*)-1-(hydroxymethyl)-2-((naphthalen-2-yl)methyl)-3-oxoisoindoline-1-carbonitrile**: Prepared following the general procedure using 10 mol% (2.3 mg) of **A50** (24 h, 40 °C). The crude product was purified by column chromatography (SiO_2, CH_2Cl_2–MeOH = 99:1) and obtained as a white solid (13.0 mg, 79%). E.r. = 94:6. M.p. 155–156 °C. $[\alpha]_D^{25}$ −50 (*c* 1.0, $CHCl_3$). ^1H NMR (400 MHz, $CDCl_3$) δ/ppm: 8.00–7.97 (m, 1H), 7.91 (br s, 1H), 7.88–7.83 (m, 3H), 7.73–7.63 (m, 3H), 7.57 (dd, *J* = 8.4, 1.8 Hz, 1H), 7.53–7.49 (m, 2H), 5.57 (d, *J* = 15.6 Hz, 1H), 4.64 (d, *J* = 15.6 Hz, 1H), 3.90 (d, *J* = 11.8 Hz, 1H), 3.72 (d, *J* = 11.8 Hz, 1H). ^{13}C NMR (100 MHz, $CDCl_3$) δ/ppm: 168.1, 140.0, 134.2, 133.30, 133.27, 133.0, 130.8, 130.7, 129.4, 127.9, 127.0, 126.8, 126.5, 125.4, 124.6, 122.6, 115.6, 65.4, 63.9, 45.0. HRMS (ESI-Q-TOF) *m/z*: $[M + H]^+$ calcd for $C_{21}H_{17}N_2O_2$ 329.1285, found 329.1256. HPLC (a Hypersil silica column (3 µm, 100 × 4.6 mm) used as a precolumn, which was connected *via* the standard blue PEEK capillary tubing (L 300 mm, ID 0.01”, OD 1/16”) to a Daicel® Chiralpak IA (5 µm, 250 × 4.6 mm), *n*-heptane–*i*-PrOH = 80:20, flow rate = 0.5 mL/min, λ = 230 nm, 25 °C) t_R = 22.35 min (major), 25.20 min (minor).

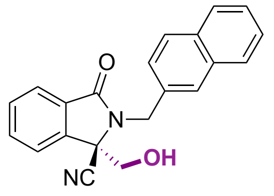

2j

Compound **2k** **(*R*)-(1-(hydroxymethyl)-3-oxo-2-(thiophen-2-ylmethyl)isoindoline-1-carbonitrile** Prepared following the general procedure using 10 mol% (2.3 mg) of **A50** (24 h, 40 °C). The crude product was purified by column chromatography (SiO_2, CH_2Cl_2–MeOH = 99:1) and obtained as a colorless waxy solid (12.9 mg, 91%). E.r. = 94:6. $[\alpha]_D^{25}$ −20 (*c* 1.0, $CHCl_3$). ^1H NMR (400 MHz, $CDCl_3$) δ/ppm: 7.91–7.89 (m, 1H), 7.70–7.67 (m, 2H), 7.64–7.58 (m, 1H), 7.30 (dd, *J* = 5.1, 1.1 Hz, 1H), 7.19 (d, *J* = 3.5 Hz, 1H), 6.99 (dd, *J* = 5.1, 3.5 Hz, 1H), 5.33 (d, *J* = 16.0 Hz, 1H), 4.86 (d, *J* = 16.0 Hz, 1H), 4.06–4.01 (m,

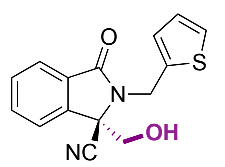

2k

1H), 3.98–3.94 (m, 1H), 2.30 (br s, 1H). ^{13}C NMR (100 MHz, $CDCl_3$) δ/ppm: 167.7, 139.9, 138.8, 133.3, 130.6, 130.5, 127.6, 127.2, 126.6, 124.6, 122.6, 115.4, 65.6, 63.4, 39.5. HRMS (APCI) *m/z*: $[M + H]^+$ calcd for $C_{15}H_{13}N_2O_2S$ 285.0692, found 285.0695. HPLC (a Hypersil silica column (3 µm, 100 × 4.6 mm) used as a precolumn, which was connected *via* the standard blue PEEK capillary tubing (L 300 mm, ID 0.01", OD 1/16") to a Daicel® Chiralcel OJ-3 column (3 µm, 150 × 4.6 mm), *n*-heptane–*i*-PrOH = 80:20, flow rate = 0.5 mL/min, λ = 230 nm, 25 °C) t_R = 24.73 min (major), 33.63 min (minor).

Compound **2l** **(*R*)-(1-(hydroxymethyl)-3-oxo-2-(thiophen-3-ylmethyl)isoindoline-1-carbonitrile)**:

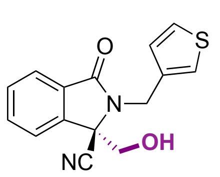

2l

Prepared following the general procedure using 10 mol% (2.3 mg) of **A50** (24 h, 40 °C). The crude product was purified by column chromatography (SiO_2, CH_2Cl_2–MeOH = 99:1) and obtained as a white solid (12.5 mg, 88%). E.r. = 90:10. M.p. 156–157 °C. $[\alpha]_D^{25}$ −32 (*c* 0.5, $CHCl_3$). ^{1}H NMR (400 MHz, $CDCl_3$) δ/ppm: 7.92–7.90 (m, 1H), 7.72–7.67 (m, 2H), 7.64–7.60 (m, 1H), 7.38–7.35 (m, 2H), 7.19 (dd, *J* = 4.9, 1.4 Hz, 1H), 5.20 (d, *J* = 15.7 Hz, 1H), 4.65 (d, *J* = 15.7 Hz, 1H), 4.00–3.96 (m, 1H), 3.89–3.86 (m, 1H), 1.98 (br s, 1H). ^{13}C NMR (100 MHz, $CDCl_3$) δ/ppm: 167.8, 139.8, 137.3, 133.2, 130.8, 130.6, 127.6, 127.5, 124.5, 123.8, 122.5, 115.5, 65.5, 63.5, 39.7. HRMS (APCI) *m/z*: $[M + H]^+$ calcd for $C_{15}H_{13}N_2O_2S$ 285.0692, found 285.0693. HPLC (a Hypersil silica column (3 µm, 100 × 4.6 mm) used as a precolumn, which was connected *via* the standard blue PEEK capillary tubing (L 300 mm, ID 0.01", OD 1/16") to a Daicel® Chiralpak IA (5 µm, 250 × 4.6 mm), *n*-heptane–*i*-PrOH = 95:5, flow rate = 0.5 mL/min, λ = 230 nm, 25 °C) t_R = 78.92 min (major), 83.78 min (minor).

Compound **2m** **(*R*)-2-((1*H*-indol-3-yl)methyl)-1-(hydroxymethyl)-3-oxoisoindoline-1-carbonitrile**:

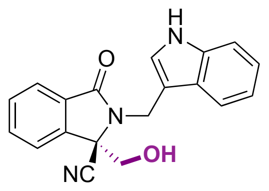

2m

Prepared following the general procedure using 10 mol% (2.3 mg) of **A50** (24 h, 40 °C). The crude product was purified by column chromatography (SiO_2, *n*-hexane–EtOAc = 2:1→1:1) and obtained as a brown solid (8.5 mg, 54%). E.r. = 93:7. M.p. 53–55 °C. $[\alpha]_D^{25}$ −33 (*c* 1.0, THF). ^{1}H NMR (400 MHz, $CDCl_3$) δ/ppm: 8.36 (br s, 1H), 7.97–7.94 (m, 1H), 7.83–7.81 (m, 1H), 7.69–7.59 (m, 3H), 7.42–7.38 (m, 2H), 7.26–7.22 (m, 1H), 7.18–7.14 (m, 1H), 5.57 (dd, *J* = 15.6, 0.9 Hz, 1H), 4.77 (d, *J* = 15.7 Hz, 1H), 4.00 (d, *J* = 3.1 Hz, 1H), 3.88 (d, *J* = 11.8 Hz, 1H), 3.74 (d, *J* = 11.8 Hz, 1H). ^{13}C NMR (100 MHz, $CDCl_3$) δ/ppm: 168.1, 140.1, 136.4, 133.1, 131.1, 130.5, 125.8, 124.5, 124.2, 123.2, 122.3, 120.9, 118.7, 115.5, 111.7, 110.9, 65.4, 63.6, 36.1. HRMS (APCI) *m/z*: $[M + H]^+$ calcd for $C_{19}H_{16}N_3O_2$ 318.1237, found 318.1240. HPLC (a Hypersil silica column (3 µm, 100 × 4.6 mm) used as a precolumn, which was connected *via* the standard blue PEEK capillary tubing (L 300 mm, ID 0.01", OD 1/16") to a Daicel® Chiralpak IA (5 µm, 250 × 4.6 mm), *n*-heptane–*i*-PrOH = 90:10, flow rate = 0.5 mL/min, 25 °C) t_R = 77.40 min (major), 86.10 min (minor).

Compound **2n** (***R***)**-(1-(hydroxymethyl)-3-oxo-2-phenylisoindoline-1-carbonitrile**: Prepared following the general procedure using 20 mol% (4.5 mg) of **A50** (48 h, 25 °C). The crude product was purified by column chromatography (SiO_2, CH_2Cl_2–MeOH = 99:1) and obtained as a white solid (12.4 mg, 94%). E.r. = 88.5:11.5. M.p. 158–160 °C. $[\alpha]_D^{25}$ −22 (*c* 1.0, $CHCl_3$). ^{1}H NMR (400 MHz, $CDCl_3$) δ/ppm: 7.89–7.87 (m, 1H), 7.77–7.71 (m, 2H), 7.64–7.60 (m, 1H), 7.52–7.44 (m, 5H), 4.16 (dd, *J* = 11.5, 6.0, 1H), 3.85 (dd, *J* = 11.5, 7.2, 1H), 2.60 (t, *J* = 6.7 Hz, 1H). ^{13}C NMR (100 MHz, $CDCl_3$) δ/ppm: 167.5, 139.8, 134.3, 133.4, 131.0, 130.8, 129.9, 129.1, 128.3, 124.8, 122.8, 116.4, 64.4, 64.3. HRMS (APCI) *m/z*: $[M + H]^+$ calcd for $C_{16}H_{13}N_2O_2$ 265.0972, found 265.0973. HPLC (a Hypersil silica column (3 µm, 100 × 4.6 mm) used as a precolumn, which was connected *via* the standard blue PEEK capillary tubing (L 300 mm, ID 0.01", OD 1/16") to a Daicel® Chiralpak IA (5 µm, 250 × 4.6 mm), *n*-heptane–*i*-PrOH = 80:20, flow rate = 0.5 mL/min, λ = 230 nm, 25 °C) t_R = 19.53 min (major), 24.53 min (minor).

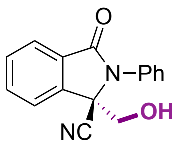

2n

Compound **2o** (***R***)**-2-(2-fluorophenyl)-1-(hydroxymethyl)-3-oxoisoindoline-1-carbonitrile**: Prepared following the general procedure using 20 mol% (4.5 mg) of **A50** (48 h, 25 °C). The crude product was purified by column chromatography (SiO_2, CH_2Cl_2–MeOH = 99:1) and obtained as a white solid (10.1 mg, 72%). E.r. = 94.5:5.5. M.p. 221–222 °C. $[\alpha]_D^{25}$ −43 (*c* 0.5, THF). ^{1}H NMR (400 MHz, DMSO-d_6) δ/ppm: 8.02–8.00 (m, 1H), 7.91–7.85 (m, 2H), 7.77–7.73 (m 1H), 7.63–7.58 (m, 2H), 7.50–7.39 (m, 2H), 6.00 (t, *J* = 5.6 Hz, 1H), 4.16 (dd, *J* = 11.2, 5.6 Hz, 1H), 3.87 (dd, *J* = 11.2, 5.6 Hz, 1H). ^{13}C NMR (100 MHz, DMSO-d_6) δ/ppm: 166.4, 158.8 (d, J_{CF} = 250.5 Hz), 140.9, 133.7, 131.6 (d, J_{CF} = 7.7 Hz), 131.5, 130.7, 129.4, 125.2, 123.8 (d, J_{CF} = 23.1 Hz), 121.9 (d, J_{CF} = 12.5 Hz), 116.6 (d, J_{CF} = 19.3 Hz), 116.2, 64.2, 63.7. ^{19}F NMR (376 MHz, DMSO-d_6) δ/ppm: −118.57 to −118.63 (m, 1F). HRMS (APCI) *m/z*: $[M + H]^+$ calcd for $C_{16}H_{12}FN_2O_2$ 283.0877, found 283.0876. HPLC (a Hypersil silica column (3 µm, 100 × 4.6 mm) used as a precolumn, which was connected *via* the standard blue PEEK capillary tubing (L 300 mm, ID 0.01", OD 1/16") to a Daicel® Chiralpak IA (5 µm, 250 × 4.6 mm), *n*-heptane–*i*-PrOH = 80:20, flow rate = 0.5 mL/min, λ = 230 nm, 25 °C) t_R = 19.95 min (major), 22.44 min (minor).

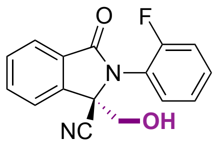

2o

Compound **2p** (***R***)**-1-(hydroxymethyl)-2-(2-methoxyphenyl)-3-oxoisoindoline-1-carbonitrile**: Prepared following the general procedure using 20 mol% (4.5 mg) of **A50** (48 h, 25 °C). The crude product was purified by column chromatography (SiO_2, CH_2Cl_2–MeOH = 99:1) and obtained as a white solid (13.0 mg, 88%). E.r. = 97:3. M.p. 204–207 °C. $[\alpha]_D^{25}$ −58 (*c* 0.5, THF). ^{1}H NMR (400 MHz, DMSO-d_6) δ/ppm: 7.96–7.95 (m, 1H), 7.86–7.81 (m, 2H), 7.74–7.70 (m, 1H), 7.54–7.49 (m, 1H), 7.41 (dd, *J* = 7.7, 1.5 Hz, 1H), 7.25–7.23 (m, 1H), 7.12–7.09 (m, 1H), 5.91 (t, *J* = 5.5 Hz, 1H), 4.08 (dd, *J* = 11.2, 5.5 Hz, 1H), 3.76 (dd, *J* = 11.2, 5.5 Hz, 1H), 3.74 (s, 3H). ^{13}C NMR (100 MHz, DMSO-d_6) δ/ppm: 166.5, 156.6, 141.2, 133.2, 131.0, 130.9, 130.4, 130.0, 123.7, 122.8, 120.7, 116.4, 112.7, 64.0, 63.8, 55.7.

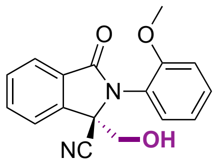

2p

HRMS (APCI) *m/z*: $[M + H]^+$ calcd for $C_{17}H_{15}N_2O_3$ 295.1077, found 295.1079. HPLC (a Hypersil silica column (3 µm, 100 × 4.6 mm) used as a precolumn, which was connected *via* the standard blue PEEK capillary tubing (L 300 mm, ID 0.01", OD 1/16") to a Daicel® Chiralpak IA (5 µm, 250 × 4.6 mm), *n*-heptane–*i*-PrOH = 80:20, flow rate = 0.5 mL/min, λ = 230 nm, 25 °C) t_R = 23.57 min (major), 25.44 min (minor).

Compound **2q** **(*R*)-1-(hydroxymethyl)-2-(3-nitrophenyl)-3-oxoisoindoline-1-carbonitrile**: Prepared following the general procedure using 20 mol% (4.5 mg) of **A50** (48 h, 25 °C). The crude product was purified by column chromatography (SiO_2, CH_2Cl_2–MeOH = 99:1) and obtained as a white solid (14.5 mg, 94%). E.r. = 91:9. M.p. 158–161 °C. $[\alpha]_D^{25}$ −46 (*c* 1.0, THF). ^{1}H NMR (400 MHz, DMSO-d_6) δ/ppm: 8.46–8.45 (m, 1H), 8.35 (ddd, J = 8.3, 2.2, 0.9, 1H), 8.04–8.00 (m, 2H), 7.93–7.86 (m, 3H), 7.78–7.74 (m, 1H), 5.93 (t, *J* = 5.6 Hz, 1H), 4.19 (dd, *J* = 11.4, 5.6 Hz, 1H), 3.91 (dd, *J* = 11.4, 5.6 Hz, 1H). ^{13}C NMR (100 MHz, DMSO-d_6) δ/ppm: 166.8, 148.4, 140.3, 136.1, 134.7, 133.9, 131.0, 130.8, 130.0, 124.0, 123.5, 123.3, 123.1, 116.8, 64.2, 63.0. HRMS (APCI) *m/z*: $[M + H]^+$ calcd for $C_{16}H_{12}N_3O_4$ 310.0822, found 310.0823. HPLC (a Hypersil silica column (3 µm, 100 × 4.6 mm) used as a precolumn, which was connected *via* the standard blue PEEK capillary tubing (L 300 mm, ID 0.01", OD 1/16") to a Daicel® Chiralpak IB (5 µm, 250 × 4.6 mm), *n*-heptane–*i*-PrOH = 90:10, flow rate = 0.5 mL/min, λ = 230 nm, 25 °C) t_R = 66.60 min (minor), 68.98 min (major).

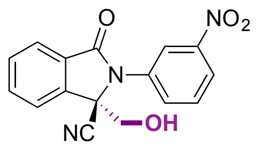

2q

Compound **2r** **(*R*)-1-(hydroxymethyl)-2-(4-methoxyphenyl)-3-oxoisoindoline-1-carbonitrile**: Prepared following the general procedure using 20 mol% (4.5 mg) of **A50** (48 h, 25 °C). The crude product was purified by column chromatography (SiO_2, CH_2Cl_2–MeOH = 99:1) and obtained as a white solid (13.0 mg, 88%). E.r. = 90:10. M.p. 154–155 °C. $[\alpha]_D^{25}$ −41 (*c* 1.0, THF). ^{1}H NMR (400 MHz, DMSO-d_6) δ/ppm: 7.98–7.96 (m, 1H), 7.87–7.81 (m, 2H), 7.74–7.70 (m, 1H), 7.40–7.37 (m, 2H), 7.13–7.09 (m, 2H), 5.87 (t, *J* = 5.7 Hz, 1H), 4.15 (dd, *J* = 11.2, 5.7 Hz, 1H), 3.83 (s, 3H), 3.75 (dd, *J* = 11.2, 5.7 Hz, 1H) ^{13}C NMR (100 MHz, DMSO-d_6) δ/ppm: 166.8, 159.4, 140.5, 133.3, 130.6, 130.5, 130.4, 126.9, 123.7, 123.5, 117.0, 114.8, 64.4, 62.9, 55.4. HRMS (APCI) *m/z*: $[M + H]^+$ calcd for $C_{17}H_{15}N_2O_3$ 295.1077, found 295.1080. HPLC (a Hypersil silica column (3 µm, 100 × 4.6 mm) used as a precolumn, which was connected *via* the standard blue PEEK capillary tubing (L 300 mm, ID 0.01", OD 1/16") to a Daicel® Chiralpak IA (5 µm, 250 × 4.6 mm), *n*-heptane–*i*-PrOH = 80:20, flow rate = 0.5 mL/min, λ = 230 nm, 25 °C) t_R = 26.33 min (major), 45.30 min (minor).

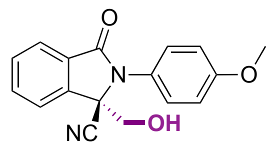

2r

Compound **2s** (***R***)**-(2-butyl-1-(hydroxymethyl)-3-oxoisoindoline-1-carbonitrile)**: Prepared following the general procedure using 10 mol% (2.3 mg) of **A50** (24 h, 40 °C). The crude product was purified by column chromatography (SiO_2, CH_2Cl_2–MeOH = 99:1) and obtained as a viscous oil (7.9 mg, 65%). E.r. = 85:15. $[\alpha]_D^{25}$ −19 (*c* 0.5, $CHCl_3$). ^{1}H NMR (400 MHz, $CDCl_3$) δ/ppm: 7.80–7.78 (m, 1H), 7.71–7.65 (m, 2H), 7.59–7.55 (m, 1H), 4.23–4.18 (m, 1H), 4.13–4.08 (m, 1H), 3.72 (ddd, *J* = 14.3, 10.0, 6.2 Hz, 1H), 3.56 (ddd, *J* = 14.3, 10.0, 5.5 Hz, 1H), 3.01–2.95 (m, 1H), 1.89–1.70 (m, 2H), 1.43 (app sext, *J* = 7.5 Hz, 2H), 0.98 (t, *J* = 7.5 Hz, 3H). ^{13}C NMR (100 MHz, $CDCl_3$) δ/ppm: 168.0, 139.8, 132.9, 131.3, 130.6, 124.2, 122.5, 116.3, 65.5, 62.9, 41.5, 30.3, 20.3, 13.7. HRMS (APCI) *m/z*: $[M + H]^+$ calcd for $C_{14}H_{17}N_2O_2$ 245.1285, found 245.1283. HPLC (a Hypersil silica column (3 µm, 100 × 4.6 mm) used as a precolumn, which was connected *via* the standard blue PEEK capillary tubing (L 300 mm, ID 0.01”, OD 1/16”) to a Daicel® Chiralpak IA (5 µm, 250 × 4.6 mm), *n*-heptane–*i*-PrOH = 95:5, flow rate = 0.5 mL/min, λ = 230 nm, 25 °C) t_R = 42.76 min (major), 51.99 min (minor).

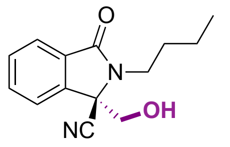

2s

Compound **2t** (***R***)**-1-(hydroxymethyl)-2-octadecyl-3-oxoisoindoline-1-carbonitrile**: Prepared following the general procedure using 10 mol% (2.3 mg) of **A50** (24 h, 40 °C). The crude product was purified by column chromatography (SiO_2, CH_2Cl_2–MeOH = 99:1) and obtained as a white solid (17.1 mg, 78%). E.r. = 85.5:14.5. M.p. 94–96 °C. $[\alpha]_D^{25}$ −12 (*c* 0.5, $CHCl_3$). ^{1}H NMR (400 MHz, $CDCl_3$) δ/ppm: 7.84–7.75 (m, 1H), 7.71–7.64 (m, 2H), 7.61–7.53 (m, 1H), 4.22–4.19 (m, 1H), 4.12–4.08 (m, 1H), 3.76–3.65 (m, 1H), 3.59–3.52 (m, 1H), 1.91–1.71 (m, 2H), 1.26 (br s, 30H), 0.89 (t, *J* = 6.8 Hz, 3H). ^{13}C NMR (100 MHz, $CDCl_3$) δ/ppm: 168.0, 139.7, 132.9, 131.3, 130.5, 124.2, 122.5, 116.3, 65.5, 62.9, 41.8, 31.9, 29.69, 29.65, 29.59, 29.56, 29.4, 29.2, 28.4, 27.1, 22.7, 14.1 HRMS (APCI) *m/z*: $[M + H]^+$ calcd for $C_{28}H_{45}N_2O_2$ 441.3476, found 441.3474. HPLC (a Hypersil silica column (3 µm, 100 × 4.6 mm) used as a precolumn, which was connected *via* the standard blue PEEK capillary tubing (L 300 mm, ID 0.01”, OD 1/16”) to a Daicel® Chiralpak IA (5 µm, 250 × 4.6 mm), *n*-heptane–*i*-PrOH = 90:10, flow rate = 0.5 mL/min, λ = 230 nm, 25 °C) t_R = 16.49 min (major), 18.98 min (minor).

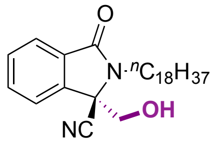

2t

Compound **2u** (***R***)**-(1-(hydroxymethyl)-3-oxo-2-phenethylisoindoline-1-carbonitrile)**: Prepared following the general procedure using 20 mol% (4.5 mg) of **A50** (24 h, 25 °C). The crude product was purified by column chromatography (SiO_2, CH_2Cl_2–MeOH = 99:1) and obtained as a white solid (10.1 mg, 69%). E.r. = 86.5:13.5. M.p. 169–171 °C. $[\alpha]_D^{25}$ −31 (*c* 0.5, $CHCl_3$). ^{1}H NMR (400 MHz, $CDCl_3$) δ/ppm: 7.79–7.77 (m, 1H), 7.64–7.63 (m, 2H), 7.55–7.51 (m, 1H), 7.31–7.21 (m, 5H), 4.00–3.92 (m, 2H), 3.85–3.81 (m, 2H), 3.23–3.08 (m, 2H), 3.03–3.01 (m, 1H). ^{13}C NMR (100 MHz, $CDCl_3$) δ/ppm: 168.4, 139.7, 138.4, 133.0, 131.1, 130.6, 128.9, 128.7, 126.8, 124.1, 122.5, 116.0, 65.6, 63.2, 44.0, 34.2. HRMS (APCI) *m/z*: $[M + H]^+$ calcd for $C_{18}H_{17}N_2O_2$ 293.1285, found 293.1282. HPLC (a Hypersil silica column (3 µm, 100 × 4.6

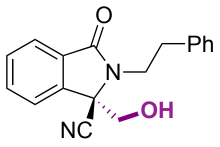

2u

mm) used as a precolumn, which was connected *via* the standard blue PEEK capillary tubing (L 300 mm, ID 0.01”, OD 1/16”) to a Daicel® Chiralpak IA (5 µm, 250 × 4.6 mm), *n*-heptane–*i*-PrOH = 80:20, flow rate = 0.5 mL/min, λ = 230 nm, 25 °C) t_R = 17.76 min (major), 22.30 min (minor).

Compound **2v** **(*R*)-(1-(hydroxymethyl)-3-oxo-2-(2,2,2-trifluoroethyl)isoindoline-1-carbonitrile)**: Prepared following the general procedure using 10 mol% (2.3 mg) of **A50** (24 h, 40 °C). The crude product was purified by column chromatography (SiO_2, CH_2Cl_2–MeOH = 99:1) and obtained as a white solid (11.2 mg, 83%). E.r. = 95:5. M.p. 128–129 °C. $[\alpha]_D^{25}$ −21 (*c* 1.0, $CHCl_3$). ^{1}H NMR (400 MHz, $CDCl_3$) δ/ppm: 7.93–7.91 (m, 1H), 7.79–7.74 (m, 2H), 7.69–7.63 (m, 1H), 4.50–4.39 (m, 1H), 4.30–4.16 (m, 3H), 2.59 (br s, 1H). ^{13}C NMR (100 MHz, $CDCl_3$) δ/ppm: 168.4, 139.9, 134.1, 131.1, 129.3, 125.0, 123.6 (q, $^1J_{CF}$ = 280.0 Hz), 122.9, 115.2, 65.8, 63.2, 43.0 (q, $^2J_{CF}$ = 36.3 Hz). ^{19}F NMR (376 MHz, $CDCl_3$) δ/ppm: −68.44 (t, J = 8.7 Hz, 3F). HRMS (APCI) *m/z*: $[M + H]^+$ calcd for $C_{12}H_{10}F_3N_2O_2$ 271.0689, found 271.0689. HPLC (a Hypersil silica column (3 µm, 100 × 4.6 mm) used as a precolumn, which was connected *via* the standard blue PEEK capillary tubing (L 300 mm, ID 0.01”, OD 1/16”) to a Daicel® Chiralpak IB (5 µm, 250 × 4.6 mm), *n*-heptane–*i*-PrOH = 95:5, flow rate = 0.5 mL/min, λ = 230 nm, 25 °C) t_R = 47.23 min (major), 50.89 min (minor).

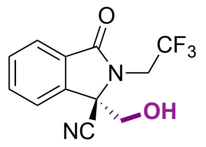

2v

Compound **2w** **(*R*)-(2-allyl-1-(hydroxymethyl)-3-oxoisoindoline-1-carbonitrile)**: Prepared following the general procedure using 10 mol% (2.3 mg) of **A50** (24 h, 40 °C). The crude product was purified by column chromatography (SiO_2, CH_2Cl_2–MeOH = 99:1) and obtained as a white solid (7.5 mg, 66%). E.r. = 90:10. M.p. 114–116 °C. $[\alpha]_D^{25}$ −22 (*c* 0.5, $CHCl_3$). ^{1}H NMR (400 MHz, $CDCl_3$) δ/ppm: 7.85–7.83 (m, 1H), 7.72–7.66 (m, 2H), 7.61–7.57 (m, 1H), 6.08–5.98 (m, 1H), 5.44–5.40 (m, 1H), 5.33–5.30 (m, 1H), 4.50–4.43 (m, 1H), 4.25–4.15 (m, 2H), 4.10–4.06 (m, 1H), 2.72 (br s, 1H). ^{13}C NMR (100 MHz, $CDCl_3$) δ/ppm: 167.6, 140.0, 133.1, 132.7, 130.9, 130.6, 124.4, 122.6, 119.0, 115.9, 65.6, 63.3, 43.9. HRMS (APCI) *m/z*: $[M + H]^+$ calcd for $C_{13}H_{13}N_2O_2$ 229.0972, found 229.0971. HPLC (a Hypersil silica column (3 µm, 100 × 4.6 mm) used as a precolumn, which was connected *via* the standard blue PEEK capillary tubing (L 300 mm, ID 0.01”, OD 1/16”) to a Daicel® Chiralpak IA (5 µm, 250 × 4.6 mm), *n*-heptane–*i*-PrOH = 95:5, flow rate = 0.5 mL/min, λ = 230 nm, 25 °C) t_R = 55.35 min (major), 59.85 min (minor).

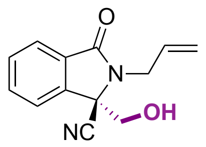

2w

Compound **2x** **(*R*)-1-(hydroxymethyl)-3-oxo-2-(prop-2-yn-1-yl)isoindoline-1-carbonitrile**: Prepared following the general procedure using 10 mol% (2.3 mg) of **A50** (24 h, 40 °C). The crude product was purified by column chromatography (SiO_2, CH_2Cl_2–MeOH = 99:1) and obtained as a colorless viscous oil (10.3 mg, 91%). E.r. = 94:6. $[\alpha]_D^{25}$ +7 (*c* 0.5, $CHCl_3$). ^{1}H NMR (400 MHz, DMSO-d_6) δ/ppm: 7.93–7.91 (m, 1H), 7.82–7.77 (m, 2H), 7.70–7.66 (m, 1H), 5.89 (t, *J* = 5.8 Hz, 1H), 4.58–4.53 (m, 1H), 4.40–4.35 (m, 1H), 4.20–4.16 (m, 1H), 4.07–4.03 (m, 1H), 3.26 (t, *J* = 2.5 Hz, 1H). ^{13}C NMR (100 MHz, DMSO-d_6) δ/ppm: 166.2, 140.7, 133.3, 130.4, 129.8, 123.4, 123.3, 116.2, 78.1, 74.7, 63.5, 61.9, 29.5. HRMS (APCI) *m/z*: $[M + H]^+$ calcd for $C_{13}H_{11}N_2O_2$ 227.0815, found 227.0815. HPLC (a Hypersil silica column (3 µm, 100 × 4.6 mm) used as a precolumn, which was connected *via* the standard blue PEEK capillary tubing (L 300 mm, ID 0.01”, OD 1/16”) to a Daicel® Chiralcel OJ-3 column (3 µm, 150 × 4.6 mm), *n*-heptane–*i*-PrOH = 90:10, flow rate = 0.5 mL/min, λ = 230 nm, 25 °C) t_R = 48.88 min (major), 53.44 min (minor).

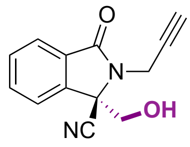

2x

Compound **2y** ***tert*-butyl (*R*)-(2-(1-cyano-1-(hydroxymethyl)-3-oxoisoindolin-2-yl)ethyl)carbamate**: Prepared following the general procedure using 10 mol% (2.3 mg) of **A50** (24 h, 40 °C). The crude product was purified by preparative TLC (SiO_2, CH_2Cl_2–MeOH = 99:1) and obtained as white solid (8 mg, 48%). E.r. = 95.5:4.5. M.p. 48–49 °C. $[\alpha]_D^{25}$ −64 (*c* 0.5, $CHCl_3$). ^{1}H NMR (400 MHz, $CDCl_3$) δ/ppm: 7.84–7.82 (m, 1H), 7.72–7.68 (m, 1H), 7.66–7.63 (m, 1H), 7.61–7.57 (m, 1H), 5.27 (dd, *J* = 8.3, 4.3 Hz, 1H), 5.10 (br s, 1H), 4.35–4.32 (m, 1H), 4.18–4.07 (m, 2H), 3.72–3.67 (m, 1H), 3.55–3.50 (m, 1H), 3.33–3.30 (m, 1H), 1.35 (s, 9H). ^{13}C NMR (100 MHz, $CDCl_3$) δ/ppm: 169.2, 157.5, 139.9, 133.2, 130.9, 130.4, 124.1, 122.3, 115.8, 80.7, 65.4, 64.8, 42.0, 40.4, 28.2. HRMS (APCI) *m/z*: $[M + H - Boc + H]^+$ calcd for $C_{12}H_{14}N_3O_2$ 232.1081, found 232.1079. HPLC (a Hypersil silica column (3 µm, 100 × 4.6 mm) used as a precolumn, which was connected *via* the standard blue PEEK capillary tubing (L 300 mm, ID 0.01”, OD 1/16”) to a Phenomenex® Lux Cellulose-1 (3 µm, 250 × 4.6 mm), *n*-heptane–*i*-PrOH = 90:10, flow rate = 0.5 mL/min, λ = 230 nm, 25 °C) t_R = 30.03 min (major), 32.91 min (minor).

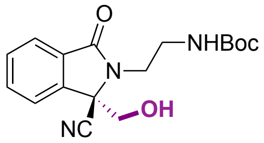

2y

Compound **2z** **methyl (*R*)-3-(1-cyano-1-(hydroxymethyl)-3-oxoisoindolin-2-yl)propanoate**: Prepared following the general procedure using 10 mol% (2.3 mg) of **A50** (24 h, 40 °C). The crude product was purified by column chromatography (SiO_2, CH_2Cl_2–MeOH = 99:1) and obtained as a white solid (7 mg, 51%). E.r. = 94:6. M.p. 125–128 °C. $[\alpha]_D^{25}$ −14 (*c* 0.5, $CHCl_3$). ^{1}H NMR (400 MHz, $CDCl_3$) δ/ppm: 7.84–7.82 (m, 1H), 7.71–7.64 (m, 2H), 7.61–7.57 (m, 1H), 4.32–4.20 (m, 2H), 4.10–4.06 (m, 1H), 4.03–3.96 (m, 1H), 3.93–3.85 (m, 1H), 3.72 (s, 3H), 3.37–3.29 (m, 1H), 2.84–2.77 (m, 1H). ^{13}C NMR (100 MHz, $CDCl_3$) δ/ppm: 173.6, 169.1, 139.7, 133.2, 131.0, 130.6, 124.2, 122.3, 115.8, 65.7, 63.8, 52.4, 38.1, 32.3. HRMS (APCI) *m/z*: $[M + H]^+$ calcd for $C_{14}H_{15}N_2O_4$ 275.1026, found 275.1027. HPLC (a Hypersil silica column (3 µm, 100 × 4.6 mm) used as a precolumn, which was connected *via* the standard

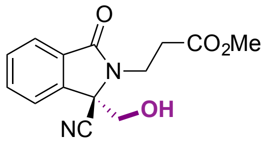

2z

blue PEEK capillary tubing (L 300 mm, ID 0.01", OD 1/16") to a Daicel® Chiralpak IA (5 µm, 250 × 4.6 mm), *n*-heptane–*i*-PrOH = 80:20, flow rate = 0.5 mL/min, λ = 230 nm) t_R= 22.00 min (major), 24.12 min (minor).

Compound **2za** **(*R*)-2-benzyl-1-(hydroxymethyl)-7-methyl-3-oxoisoindoline-1-carbonitrile**: Prepared following the general procedure using 10 mol% (2.3 mg) of **A50** (24 h, 40 °C). The crude product was purified by column chromatography (SiO_2, CH_2Cl_2–MeOH = 99:1) and obtained as a white solid (7 mg, 48%). E.r. = 94:6. M.p. 37–39 °C. $[\alpha]_D^{25}$ −58 (*c* 0.5, $CHCl_3$). ^{1}H NMR (400 MHz, DMSO-d_6) δ/ppm: 7.67–7.65 (m, 1H), 7.60–7.55 (m, 2H), 7.39–7.37 (m, 2H), 7.34–7.31 (m, 2H), 7.28–7.24 (m, 1H), 5.70 (app t, *J* = 6.2 Hz, 1H), 5.04 (d, *J* = 16.1 Hz, 1H), 4.65 (d, *J* = 16.1 Hz, 1H), 4.25 (dd, *J* = 12.0, 6.0 Hz, 1H), 4.11 (dd, *J* = 12.0, 6.4 Hz, 1H), 2.53 (s, 3H). ^{13}C NMR (100 MHz, DMSO-d_6) δ/ppm: 167.2, 137.2, 136.6, 134.9, 133.4, 131.3, 130.3, 128.2, 127.7, 127.2, 121.0, 115.2, 62.4, 61.0, 43.3, 17.2. HRMS (APCI) *m/z*: $[M + H]^+$ calcd for $C_{18}H_{17}N_2O_2$ 293.1285, found 293.1285. HPLC (a Hypersil silica column (3 µm, 100 × 4.6 mm) used as a precolumn, which was connected *via* the standard blue PEEK capillary tubing (L 300 mm, ID 0.01", OD 1/16") to a Daicel® Chiralpak IA (5 µm, 250 × 4.6 mm), *n*-heptane–*i*-PrOH = 80:20, flow rate = 0.5 mL/min, λ = 230 nm, 25 °C) t_R= 18.57 min (major), 20.89 min (minor).

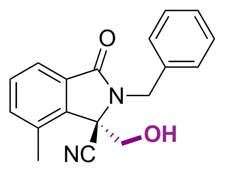

2za

Compound **2zb** **(*R*)-2-benzyl-6-bromo-1-(hydroxymethyl)-3-oxoisoindoline-1-carbonitrile**: Prepared following the general procedure using 10 mol% (2.3 mg) of **A50** (24 h, 40 °C). The crude product was purified by column chromatography (SiO_2, CH_2Cl_2–MeOH = 99:1) and obtained as a white solid (15.0 mg, 84%). E.r. = 91.5:8.5. M.p. 155–157 °C. $[\alpha]_D^{25}$ −56 (*c* 1.0, $CHCl_3$). ^{1}H NMR (400 MHz, $CDCl_3$) δ/ppm: 7.87–7.86 (m, 1H), 7.79–7.74 (m, 2H), 7.44–7.42 (m, 2H), 7.40–7.32 (m, 3H), 5.28 (d, *J* = 15.6 Hz, 1H), 4.52 (d, *J* = 15.6 Hz, 1H), 3.92 (dd, *J* = 11.8, 7.8 Hz, 1H), 3.70 (dd, *J* = 11.8, 6.2 Hz, 1H), 2.08 (t, *J* = 7.0 Hz, 1H). ^{13}C NMR (100 MHz, $CDCl_3$) δ/ppm: 167.2, 141.7, 136.3, 134.1, 129.6, 129.2, 128.5, 128.0, 127.9, 126.3, 125.8, 115.0, 65.1, 63.3, 44.8. HRMS (APCI) *m/z*: $[M + H]^+$ calcd for $C_{17}H_{14}BrN_2O_2$ 357.0233, found 357.0234. HPLC (a Hypersil silica column (3 µm, 100 × 4.6 mm) used as a precolumn, which was connected *via* the standard blue PEEK capillary tubing (L 300 mm, ID 0.01", OD 1/16") to a Daicel® Chiralpak IA (5 µm, 250 × 4.6 mm), *n*-heptane–*i*-PrOH = 80:20, flow rate = 0.5 mL/min, λ = 230 nm, 25 °C) t_R = 16.96 min (major), 18.81 min (minor).

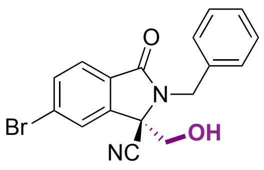

2zb

Compound **2zc** **(*R*)-5-chloro-1-(hydroxymethyl)-2-(4-methoxybenzyl)-3-oxoisoindoline-1-carbonitrile**:

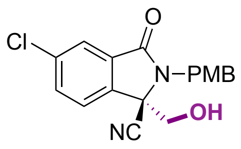

2zc

Prepared following the general procedure using 10 mol% (2.3 mg) of **A50** (24 h, 40 °C). The crude product was purified by column chromatography (SiO_2, CH_2Cl_2–MeOH = 99:1) and obtained as a white solid (13.0 mg, 76%). E.r. = 93:7. M.p. 158–159 °C. $[\alpha]_D^{25}$ −49 (*c* 1.0, $CHCl_3$). ^1H NMR (400 MHz, $CDCl_3$) δ/ppm: 7.88 (dd, *J* = 1.8, 0.6 Hz, 1H), 7.67–7.64 (m, 1H), 7.63–7.61 (m, 1H), 7.40–7.36 (m, 2H), 6.92–6.88 (m, 2H), 5.27 (d, *J* = 15.5 Hz, 1H), 4.44 (d, *J* = 15.5 Hz, 1H), 3.92 (dd, *J* = 11.8, 8.1 Hz, 1H), 3.81 (s, 3H), 3.72 (dd, *J* = 11.8, 6.2 Hz, 1H), 1.80 (t, *J* = 7.0 Hz, 1H). ^{13}C NMR (100 MHz, $CDCl_3$) δ/ppm: 166.7, 159.7, 138.2, 137.2, 133.3, 132.6, 129.5, 128.2, 124.7, 123.9, 115.1, 114.6, 65.1, 63.5, 55.3, 44.3. HRMS (APCI) *m/z*: $[M + H]^+$ calcd for $C_{18}H_{16}ClN_2O_3$ 343.0844, found 343.0846. HPLC (a Hypersil silica column (3 µm, 100 × 4.6 mm) used as a precolumn, which was connected *via* the standard blue PEEK capillary tubing (L 300 mm, ID 0.01”, OD 1/16”) to a Daicel® Chiralpak IB (5 µm, 250 × 4.6 mm), *n*-heptane–*i*-PrOH = 80:20, flow rate = 0.5 mL/min, λ = 230 nm, 25 °C) t_R = 21.89 min (major), 24.12 min (minor).

Compound **2zd** **(*R*)-2-benzyl-4-chloro-1-(hydroxymethyl)-3-oxoisoindoline-1-carbonitrile**: Prepared

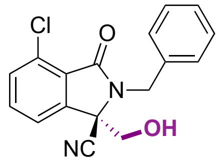

2zd

following the general procedure using 10 mol% (2.3 mg) of **A50** (24 h, 40 °C). The crude product was purified by column chromatography (SiO_2, CH_2Cl_2–MeOH = 99:1) and obtained as a glassy solid (11.0 mg, 70%). E.r. = 94.5:5.5. $[\alpha]_D^{25}$ −89 (*c* 1.0, $CHCl_3$). ^1H NMR (400 MHz, $CDCl_3$) δ/ppm: 7.64–7.56 (m, 3H), 7.50–7.48 (m, 2H), 7.42–7.35 (m, 3H), 5.39 (d, *J* = 15.6, Hz, 1H), 4.45 (d, *J* = 15.6, Hz, 1H), 3.91 (dd, *J* = 11.8, 7.8 Hz, 1H), 3.74 (dd, *J* = 11.8, 5.6 Hz, 1H), 1.60–1.58 (m, 1H). ^{13}C NMR (100 MHz, $CDCl_3$) δ/ppm: 165.7, 142.1, 136.6, 133.8, 132.5, 132.3, 129.3, 128.6, 128.1, 126.9, 121.1, 115.1, 65.2, 62.9, 44.7. HRMS (APCI) *m/z*: $[M + H]^+$ calcd for $C_{17}H_{14}ClN_2O_2$ 313.0738, found 313.0740. HPLC (a Hypersil silica column (3 µm, 100 × 4.6 mm) used as a precolumn, which was connected *via* the standard blue PEEK capillary tubing (L 300 mm, ID 0.01”, OD 1/16”) to a Daicel® Chiralpak IA (5 µm, 250 × 4.6 mm), *n*-heptane–*i*-PrOH = 80:20, flow rate = 0.5 mL/min, λ = 230 nm, 25 °C) t_R = 19.15 min (minor), 21.20 min (major).

Compound **2ze** **(*R*)-2-benzyl-1-(hydroxymethyl)-5,6-dimethoxy-3-oxoisoindoline-1-carbonitrile**:

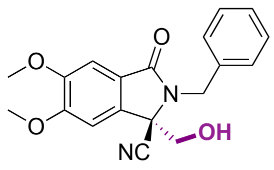

2ze

Prepared following the general procedure using 20 mol% (4.5 mg) of **A50** (48 h, 40 °C). The crude product was purified by column chromatography (SiO_2, CH_2Cl_2–MeOH = 99:1) and obtained as a white solid (13.0 mg, 77%). E.r. = 88:12. M.p. >260 °C. $[\alpha]_D^{25}$ −54 (*c* 0.5, $CHCl_3$). ^1H NMR (400 MHz, DMSO-d_6) δ/ppm: 7.46 (s, 1H), 7.37–7.24 (m, 6H), 5.81 (t, *J* = 5.8 Hz, 1H), 4.92 (d, *J* = 16.1 Hz, 1H), 4.68 (d, *J* = 16.1 Hz, 1H), 4.05–4.01 (m, 1H), 3.98–3.94 (m, 1H), 3.89 (s, 3H), 3.87 (s, 3H). ^{13}C NMR (100 MHz, DMSO-d_6) δ/ppm: 167.4, 153.2, 150.9, 137.1, 134.2, 128.3, 127.6, 127.3, 122.5, 116.7,

105.7, 105.1, 63.6, 62.0, 56.2, 56.0, 43.7. HRMS (APCI) *m/z*: $[M + H]^+$ calcd for $C_{19}H_{19}N_2O_4$ 339.1339, found 339.1342. HPLC (a Hypersil silica column (3 µm, 100 × 4.6 mm) used as a precolumn, which was connected *via* the standard blue PEEK capillary tubing (L 300 mm, ID 0.01”, OD 1/16”) to a Daicel® Chiralpak IA (5 µm, 250 × 4.6 mm), *n*-heptane–*i*-PrOH = 90:10, flow rate = 0.5 mL/min, λ = 230 nm, 25 °C) retention times: t_R = 71.72 min (major), 77.81 min (minor).

Compound **2zf** **ethyl (*S*)-1-(hydroxymethyl)-2-benzyl-3-oxoisoindoline-1-carboxylate**:[51] Prepared following the general procedure using 20 mol% (4.5 mg) of **A50** (96 h, 40 °C). The crude product was purified by column chromatography (SiO_2, CH_2Cl_2–MeOH = 99:1) and obtained as a waxy solid (11.0 mg, 68%). E.r. = 81:19. $[\alpha]_D^{25}$ +40 (*c* 0.5, $CHCl_3$), (lit.[48] $[\alpha]_D^{20}$ +4.97 (*c* 0.91, $CHCl_3$) for e.r. = 78.5:21.5). ^{1}H NMR (400 MHz, DMSO-d_6) δ/ppm: 7.75–7.73 (m, 1H), 7.67–7.54 (m, 3H), 7.36–7.33 (m, 2H), 7.30–7.26 (m, 2H), 7.23–7.20 (m, 1H), 5.13 (t, *J* = 5.1 Hz, 1H), 4.85 (d, *J* = 15.7, 1H), 4.58 (d, *J* = 15.7, 1H), 4.24 (dd, *J* = 11.4, 5.7 1H), 4.02 (dd, *J* = 11.4, 5.7 1H), 3.86–3.69 (m, 2H), 0.91 (t, *J* = 7.1 Hz, 3H). ^{13}C NMR (100 MHz, DMSO-d_6) δ/ppm: 168.8, 168.5, 142.8, 137.8, 132.2, 131.6, 129.2, 128.0, 127.8, 126.7, 122.9, 122.5, 72.4, 61.9, 61.5, 44.5, 13.4. HRMS (APCI) *m/z*: $[M + H]^+$ calcd for $C_{19}H_{20}NO_4$ 326.1387, found 326.1390. HPLC (a Hypersil silica column (3 µm, 100 × 4.6 mm) used as a precolumn, which was connected *via* the standard blue PEEK capillary tubing (L 300 mm, ID 0.01”, OD 1/16”) to a Daicel® Chiralpak IA (5 µm, 250 × 4.6 mm), *n*-heptane–*i*-PrOH = 80:20, flow rate = 0.5 mL/min, λ = 230 nm, 25 °C) t_R = 22.24 min (minor), 23.94 min (major).

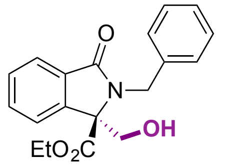

2zf

Compound **2zg** **2,2,2-trifluoroethyl (*S*)-2-benzyl-1-(hydroxymethyl)-3-oxoisoindoline-1-carboxylate**: Prepared following the general procedure using 20 mol% (4.5 mg) of **A50** (24 h, 40 °C). The crude product was purified by column chromatography (SiO_2, CH_2Cl_2–MeOH = 99:1) and obtained as a waxy solid (12.5 mg, 66%). E.r. = 93.5:6.5. $[\alpha]_D^{25}$ +20 (*c* 1.0, $CHCl_3$). ^{1}H NMR (400 MHz, $CDCl_3$) δ/ppm: 7.96–7.93 (m, 1H), 7.63–7.53 (m, 3H), 7.44–7.42 (m, 2H), 7.37–7.29 (m, 3H), 5.29 (d, *J* = 15.9 Hz, 1H), 4.54 (d, *J* = 15.9 Hz, 1H), 4.42–4.24 (m, 2H), 4.16 (dd, *J* = 12.2, 5.8 Hz, 1H), 4.02 (dd, *J* = 12.2, 7.1 Hz, 1H), 1.54 (app t, *J* = 7.0 Hz, 1H). ^{13}C NMR (100 MHz, $CDCl_3$) δ/ppm: 169.2, 168.1, 140.6, 137.5, 132.5, 131.6, 130.0, 129.0, 128.0, 124.4, 122.3 (q, $^1J_{CF}$ = 277.4 Hz), 122.0, 72.7, 64.5, 61.0 (q, $^2J_{CF}$ = 37.3 Hz), 44.9. ^{19}F NMR (376 MHz, $CDCl_3$) δ/ppm: −73.50 (t, *J* = 8.7 Hz, 3F). HRMS (APCI) *m/z*: $[M + H]^+$ calcd for $C_{19}H_{17}F_3NO_4$ 380.1104, found 380.1102. HPLC (a Hypersil silica column (3 µm, 100 × 4.6 mm) used as a precolumn, which was connected *via* the standard blue PEEK capillary tubing (L 300 mm, ID 0.01”, OD 1/16”) to a Daicel® Chiralpak IA (5 µm, 250 × 4.6 mm), *n*-heptane–*i*-PrOH = 80:20, flow rate = 0.5 mL/min, λ = 230 nm, 25 °C) t_R = 18.67 min (minor), 20.56 min (major).

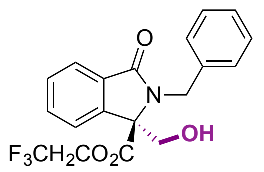

2zg

Compound **2zh** **2,2,2-trichloroethyl (*S*)-2-benzyl-1-(hydroxymethyl)-3-oxoisoindoline-1-carboxylate**:

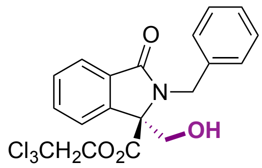

2zh

Prepared following the general procedure using 20 mol% (4.5 mg) of **A50** (48 h, 40 °C). The crude product was purified by column chromatography (SiO_2, CH_2Cl_2–MeOH = 99:1) and obtained as a yellow waxy solid (16 mg, 75%). E.r. = 91:9. $[\alpha]_D^{25}$ +33 (*c* 1.0, $CHCl_3$). ^{1}H NMR (400 MHz, $CDCl_3$) δ/ppm: 7.96–7.94 (m, 1H), 7.64–7.55 (m, 3H), 7.46–7.44 (m, 2H), 7.38–7.30 (m, 3H), 5.36 (d, J = 15.7 Hz, 1H), 4.68, 4.57 (q, AB, J_{AB} = 11.8 Hz, 2H), 4.58 (d, J = 15.7 Hz, 1H, partially overlapped with the previous signal), 4.19 (dd, J = 12.3, 4.6 Hz, 1H), 4.08 (dd, J = 12.3, 6.5 Hz, 1H), 1.49 (app t, J = 6.5 Hz, 1H). ^{13}C NMR (100 MHz, $CDCl_3$) δ/ppm: 169.3, 167.8, 140.6, 137.7, 132.4, 131.7, 130.0, 129.1, 128.0, 124.3, 122.4, 94.0, 74.4, 73.0, 64.5, 45.1. HRMS (APCI) *m/z*: $[M + H]^+$ calcd for $C_{19}H_{17}Cl_3NO_4$ 428.0218, found 428.0222. HPLC (a Hypersil silica column (3 µm, 100 × 4.6 mm) used as a precolumn, which was connected *via* the standard blue PEEK capillary tubing (L 300 mm, ID 0.01”, OD 1/16”) to a Phenomenex® Lux Amylose-1 (3 µm, 250 × 4.6 mm), *n*-heptane–*i*-PrOH = 80:20, flow rate = 0.5 mL/min, λ = 230 nm, 25 °C) t_R = 22.59 min (minor), 25.24 min (major).

4. Examples of Unsuitable Substrates

4.1 Substrates Exhibiting No Reactivity

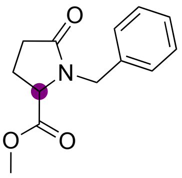

NR1

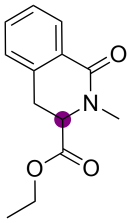

NR2

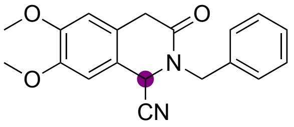

NR3

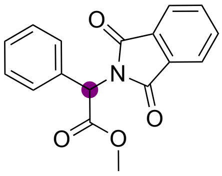

NR4

4.2 Substrates Exhibiting Insufficient Enantioselectivity

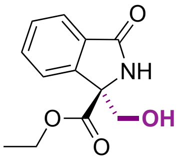

(*S*)-**XVIa**[a]
conv. >99%[b]
e.r. 76.5:23.5

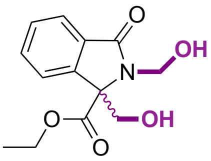

XVIb[c]
rac-**XVIa** + *rac*-**XVIb**
conv. >99%
XVIa (40%),[d] **XVIb** (39%)[d]

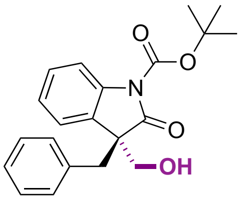

(*S*)-**XVII**[e]
conv. >99% (60%)[d]
e.r. 66:34

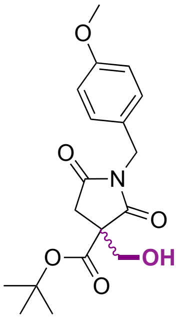

XVIII
conv. 96% (80%)[d]
e.r. 50:50

Figure S20: Examples of products with low enantioselectivity obtained under typical reaction conditions. Notes: [a] a sole product was obtained under conditions described for enantioenriched derivatives – please, see **chapter 3.5** for a procedure, the above product was highly challenging to separate from **3i** and its byproduct; [b] not isolated from the reaction mixture; [c] a mixture was produced under conditions described for racemic derivatives – please, see **chapter 3.4** for a procedure; [d] isolated yield; [e] the absolute configuration was determined by the relative elution order of resolved peaks with the literature data;[52] e.r. values were analyzed by HPLC with CSP; conversions were determined by RP-HPLC analyses of the crude reaction mixtures with external standard calibration.

Compound **XVIa ethyl (*S*)-3-oxoisoindoline-1-carboxylate**:[51] Prepared following the general procedure

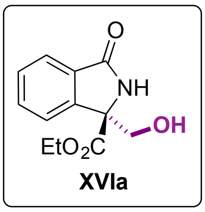

using 20 mol% (4.5 mg) of **A50** (24 h, 40 °C). The crude mixture was filtered through a silica plug (SiO_2, EtOAc), evaporated, and subjected to HPLC analysis directly, without isolation of the product (conv. >99%). E.r. = 76.5:23.5. ^{1}H NMR (400 MHz, $CDCl_3$) δ/ppm: 8.01 (br s, 1H), 7.84–7.82 (m, 1H) 7.69–7.68 (m, 1H), 7.62–7.58 (m, 1H), 7.54–7.50 (m, 1H), 4.50 (d, J = 11.2 Hz, 1H), 4.30–4.21 (m, 2H), 3.71 (d, J = 11.2 Hz, 1H), 1.27 (t, J = 7.1 Hz, 1H). ^{13}C NMR (100 MHz, $CDCl_3$) δ/ppm: 170.8, 169.6, 141.9, 132.6, 131.3, 129.7, 124.2, 123.1, 70.5, 68.5, 62.6, 14.0. HRMS (APCI) *m/z*: $[M + H]^+$ calcd for $C_{12}H_{14}NO_4$ 236.0917, found 236.0919. HPLC (a Hypersil silica column (3 µm, 100 × 4.6 mm) used as a precolumn, which was connected *via* the standard blue PEEK capillary tubing (L 300 mm, ID 0.01", OD 1/16") to a Daicel® Chiralpak IA (5 µm, 250 × 4.6 mm), *n*-heptane–*i*-PrOH = 80:20, flow rate = 0.5 mL/min, λ = 230 nm, 25 °C) t_R = 18.05 min (minor), 22.12 min (major).

Compound **XVIb ethyl *rac*-1,2-bis(hydroxymethyl)-3-oxoisoindoline-1-carboxylate**: Prepared from ethyl

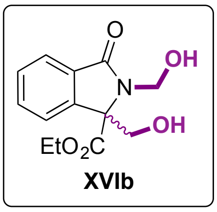

3-oxoisoindoline-1-carboxylate (50 mg, 0.24 mmol), surrogate **3i** (105 mg, 0.48 mmol), K_2CO_3 (33 mg, 0.24 mmol), and TEBAC (11 mg, 0.048 mol) following the conditions described for racemic derivatives in chapter **3.4**. The crude mixture, comprising compounds **XVIa** and **XVIb** in a 1:1 ratio, was purified by column chromatography (SiO_2, *n*-hexane–EtOAc 2:1 → 1:1 → 1:2). **XVIa** was isolated as a glassy solid (23 mg, 40%) with spectral data identical to those reported above. Compound **XVIb** was obtained as a glassy solid (25 mg, 39%) with spectral data as follows: ^{1}H NMR (400 MHz, $CDCl_3$) δ/ppm: 7.80–7.78 (m, 1H), 7.66–7.59 (m, 2H), 7.53–7.49 (m, 1H), 5.78 (dd, J = 11.6, 4.7 Hz, 1H), 5.26 (dd, J = 9.8, 5.0 Hz, 1H), 4.81 (dd, J = 11.6, 9.8 Hz, 1H), 4.55 (dd, J = 12.0, 8.3 Hz, 1H), 4.38–4.18 (m, 3H), 3.84 (dd, J = 12.0, 6.4 Hz, 1H), 1.29 (t, J = 7.2 Hz, 3H). ^{13}C NMR (100 MHz, $CDCl_3$) δ/ppm: 169.4, 168.5, 141.0, 133.0, 130.7, 129.7, 124.2, 122.6, 73.7, 67.2, 64.8, 62.6, 14.0. HRMS (APCI) *m/z*: $[M + Cl]^-$ calcd for $C_{13}H_{15}ClNO_5$ 300.0644, found 300.0649. HPLC (a Hypersil silica column (3 µm, 100 × 4.6 mm) used as a precolumn, which was connected *via* the standard blue PEEK capillary tubing (L 300 mm, ID 0.01", OD 1/16") to a Daicel® Chiralpak IA (5 µm, 250 × 4.6 mm), *n*-heptane–*i*-PrOH = 80:20, flow rate = 0.5 mL/min, λ = 230 nm, 25 °C) t_R = 16.71 min, 19.59 min.

Compound **XVII *tert*-butyl (*S*)-3-benzyl-3-(hydroxymethyl)-2-oxoindoline-1-carboxylate**:[52] Prepared

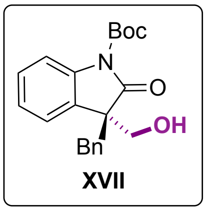

following the general procedure using 10 mol% (2.3 mg) of **A50** (24 h, 40 °C). The crude product was purified by column chromatography (SiO_2, CH_2Cl_2–MeOH = 99:1) and obtained as a waxy solid (10.6 mg, 60%). E.r. = 66:34. $[\alpha]_D^{25}$ +10 (*c* 1.0, $CHCl_3$). ^{1}H NMR (400 MHz, $CDCl_3$) δ/ppm: 7.68–7.66 (m, 1H), 7.29–7.25 (m, 1H), 7.18–7.07 (m, 5H), 6.91–6.89 (m, 2H), 4.04 (dd, J = 11.1, 9.5 Hz, 1H), 3.89 (dd, J = 11.1, 3.4 Hz, 1H), 3.23, 3.16 (q, AB. J_{AB} = 15.2 Hz, 2H), 2.34 (dd, J = 9.5, 3.4 Hz, 1H), 1.59 (s, 9H). ^{13}C NMR (100 MHz,

$CDCl_3$) δ/ppm: 178.0, 148.7, 140.1, 134.7, 130.0, 128.7, 127.9, 127.8, 126.8, 124.2, 123.5, 115.0, 84.3, 66.3, 55.9, 40.0, 28.0. HRMS (APCI) *m/z*: $[M + H - Boc + H]^+$ calcd for $C_{16}H_{16}NO_2$ 254.1176, found 254.1175. HPLC (a Hypersil silica column (3 µm, 100 × 4.6 mm) used as a precolumn, which was connected *via* the standard blue PEEK capillary tubing (L 300 mm, ID 0.01", OD 1/16") to a Daicel® Chiralpak IA (5 µm, 250 × 4.6 mm), *n*-heptane–*i*-PrOH = 90:10, flow rate = 0.5 mL/min, λ = 230 nm, 25 °C) t_R = 17.43 min (minor), 19.43 min (major).

Compound **XVIII** ***tert*-butyl *rac*-3-(hydroxymethyl)-1-(4-methoxybenzyl)-2,5-dioxopyrrolidine-3-carboxylate**: Prepared following the general procedure using 10 mol% (2.3 mg) of **A50** (24 h, 40 °C). The crude product was purified by column chromatography (SiO_2, CH_2Cl_2–MeOH = 99:1) and obtained as a white solid (14 mg, 80%). E.r. = 50:50. M.p. 85–87 °C. ^1H NMR (400 MHz, $CDCl_3$) δ/ppm: 7.31–7.28 (m, 2H), 6.84–6.80 (m, 2H), 4.62 (s, 2H), 4.06–3.97 (m, 2H), 3.77 (s, 3H), 2.96, 2.93 (q, AB, J_{AB} = 18.1 Hz, 2H), 2.31 (br s, 1H), 1.33 (s, 9H). ^{13}C NMR (100 MHz, $CDCl_3$) δ/ppm: 175.0, 174.6, 167.7, 159.3, 129.9, 127.6, 113.9, 83.8, 63.0 57.3, 55.2, 42.2, 36.3, 27.6. HRMS (APCI) *m/z*: $[M + NH_4]^+$ calcd for $C_{18}H_{27}N_2O_6$ 367.1864, found 367.1863. HPLC (a Hypersil silica column (3 µm, 100 × 4.6 mm) used as a precolumn, which was connected *via* the standard blue PEEK capillary tubing (L 300 mm, ID 0.01", OD 1/16") to a Daicel® Chiralpak IA (5 µm, 250 × 4.6 mm), *n*-heptane–*i*-PrOH = 95:5, flow rate = 0.5 mL/min, λ = 230 nm, 25 °C) t_R = 55.11 min, 60.98 min.

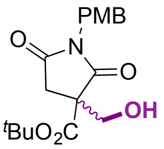

XVIII

5. A Scale-up Experiment for Adduct (*R*)-2a

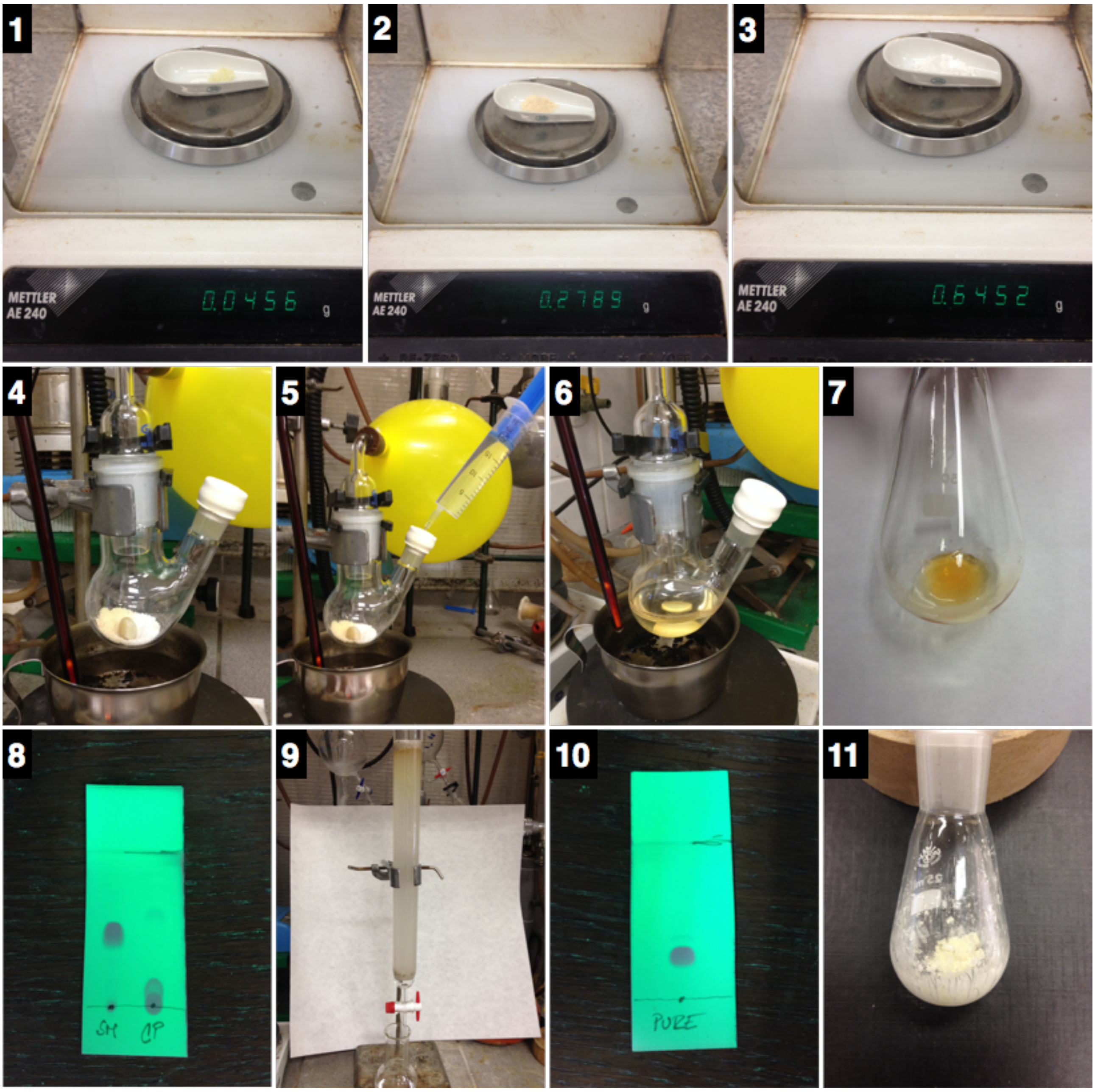

Figure S21: Performing the **A50**-catalyzed reaction between **1a** and **3i** on a 1.0 mmol scale. **(1)** weighing of the catalyst **A50** (45.6 mg, 0.1 mmol), **(2)** substrate **1a** (278.9 mg, 1.0 mmol), and **(3–4)** formaldehyde surrogate **3i** (645.2 mg, 3.0 mmol); **(5)** the addition of *tert*-butyl methyl ether (20 mL) to the reaction mixture; **(6)** the appearance of the reaction mass after 24 h at 40 °C (oil bath); **(7)** a crude product after evaporation; **(8)** with the corresponding TLC (SiO_2, CH_2Cl_2–MeOH = 99:1); **(9)** purification of the crude product by column chromatography (SiO_2, CH_2Cl_2–MeOH = 99:1); **(10)** TLC (SiO_2, CH_2Cl_2–MeOH = 99:1) of the pure product; **(11)** a pure product (*R*)-**2a** (245 mg, 80%) after evaporation; e.r. = 88:12 – for details see **chapter 9** (a copy of chromatogram).

6. Downstream Transformations of Enantioenriched Adducts

6.1 *N*-Deprotection of (*R*)-2a

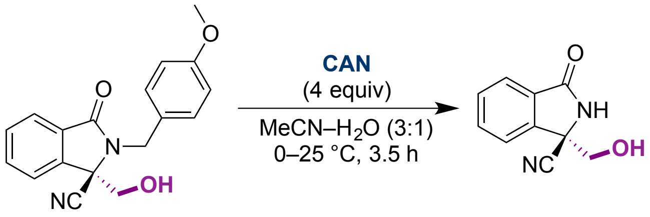

(*R*)-**2a**
e.r. 88:12

(*R*)-**6**
e.r. 88:12 (76%)

A solution of **2a** (50 mg, 0.16 mmol) in acetonitrile (3 mL) and water (1 mL) was cooled in an ice bath. Ammonium cerium(IV) nitrate (355 mg, 0.64 mmol) was added portionwise. The mixture was stirred at 0 °C for 30 min and then at ambient temperature for an additional 3 h. The reaction was quenched with a saturated aqueous solution of $NaHCO_3$, and the water layer was repetitively extracted with CH_2Cl_2. The combined organic phases were washed with brine, dried over anhydrous Na_2SO_4, filtered, and evaporated *in vacuo*. The crude residue was dissolved in CH_2Cl_2 (5 mL), and the formation of a precipitate occurred after a few minutes. Hexane (20 mL) was added to the resulting suspension, which was sonicated, filtered, and washed with several portions of hexane–EtOAc (20:1). The obtained powder was dried *in vacuo*.

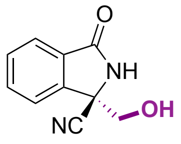

6

Compound **6 (*R*)-1-(hydroxymethyl)-3-oxoisoindoline-1-carbonitrile**: White powder (23 mg, 76%). E.r. = 88:12. M.p. 148–150 °C. $[\alpha]_D^{25}$ −50 (*c* 1.0, THF). ^{1}H NMR (400 MHz, MeOH-d_4) δ/ppm: 7.84–7.81 (m, 2H), 7.78–7.74 (m, 1H), 7.69–7.65 (m, 1H), 3.91 (s, 2H). ^{13}C NMR (100 MHz, MeOH-d_4) δ/ppm: 171.6, 143.7, 134.6, 132.4, 131.8, 125.2, 124.7, 118.7, 67.8, 61.1. HRMS (APCI) *m/z*: $[M + H]^+$ calcd for $C_{10}H_9N_2O_2$ 189.0659, found 189.0662. HPLC (a Hypersil silica column (3 μm, 100 × 4.6 mm) used as a precolumn, which was connected *via* the standard blue PEEK capillary tubing (L 300 mm, ID 0.01", OD 1/16") to a Daicel® Chiralpak OJ-3 (3 μm, 150 × 4.6 mm), *n*-heptane–*i*-PrOH = 95:5, flow rate = 0.5 mL/min, λ = 230 nm, 25 °C) t_R = 60.14 min (minor), 64.03 min (major).

6.2 Radziszewski Amidation of (*R*)-2a

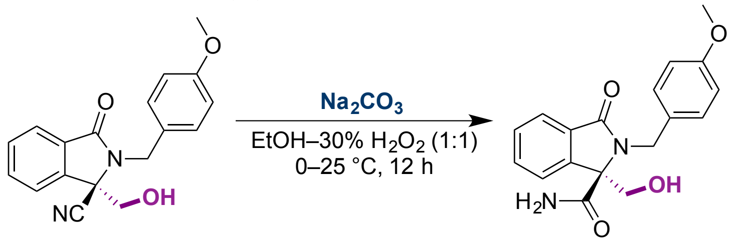

(*R*)-**2a** e.r. 88:12 (*S*)-**7** e.r. 86:14 (48%)

A modified literature procedure was used.[49] To an ice-cold solution of (*R*)-**2a** (50 mg, 0.16 mmol) in ethanol (3.2 mL), a 30% aqueous solution of H_2O_2 (3.2 mL) was added dropwise, followed by the addition of Na_2CO_3 (860 mg, 8 mmol). The given order of the reagents was essential to follow to prevent excessive racemization of the product. The resulting suspension was stirred at 0 °C for 30 min; then, it was allowed to warm to ambient temperature and left to stir overnight. Thereafter, water and CH_2Cl_2 were added, and the layers were separated. The aqueous phase was repetitively extracted with CH_2Cl_2. The combined organic phases were washed with brine, dried over anhydrous Na_2SO_4, filtered, and evaporated *in vacuo*. The product was further purified by preparative TLC (SiO_2, CH_2Cl_2–MeOH = 97:3).

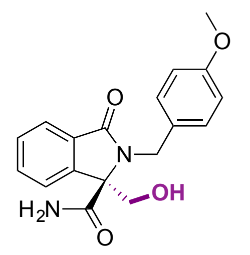

7

Compound **7** **(*S*)-1-(hydroxymethyl)-2-(4-methoxybenzyl)-3-oxoisoindoline-1-carboxamide**: White solid (25 mg, 48%). E.r. = 86:14. M.p. 74–75 °C. $[\alpha]_D^{25}$ +39 (*c* 1.0, THF). ^{1}H NMR (400 MHz, MeOH-d_4) δ/ppm: 7.85–7.83 (m, 1H), 7.65–7.63 (m, 2H), 7.59–7.55 (m, 1H), 7.38–7.34 (m, 2H), 6.87–6.84 (m, 2H), 4.84 (d, J = 15.4 Hz, 1H), 4.64 (d, J = 15.4 Hz, 1H), 4.16 (d, J = 11.7 Hz, 1H), 3.96 (d, J = 11.7 Hz, 1H), 3.76 (s, 3H). ^{13}C NMR (100 MHz, MeOH-d_4) δ/ppm: 173.3, 172.2, 160.8, 145.9, 133.7, 132.9, 131.0, 130.9, 130.5, 124.6, 123.8, 115.0, 74.1, 64.0, 55.8, 45.7. HRMS (APCI) *m/z*: $[M + H]^+$ calcd for $C_{18}H_{19}N_2O_4$ 327.1339, found 327.1341. HPLC (a Hypersil silica column (3 µm, 100 × 4.6 mm) used as a precolumn, which was connected *via* the standard blue PEEK capillary tubing (L 300 mm, ID 0.01", OD 1/16") to a Daicel® Chiralpak IA (5 µm, 250 × 4.6 mm), *n*-heptane–*i*-PrOH = 80:20, flow rate = 0.5 mL/min, λ = 230 nm, 25 °C) t_R = 50.18 min (minor), 67.00 min (major).

6.3 *O*-Acetylation of (*R*)-2a

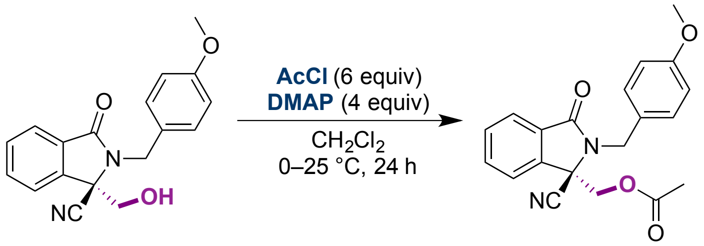

(*R*)-**2a** e.r. 88:12 (*R*)-**8** e.r. 88.5:11.5 (50%)

A solution of (*R*)-**2a** (30 mg, 0.1 mmol) in CH_2Cl_2 (1 mL) was cooled in an ice bath, DMAP (24 mg, 0.2 mmol) and acetyl chloride (22 µL, 0.3 mmol) were added. The mixture was stirred at ambient temperature under Ar. After 6 h, the reaction mass was cooled, and additional portions of DMAP (24 mg, 0.2 mmol) and acetyl chloride (22 µL, 0.3 mmol) were added thereto. The resulting mixture was left to stir at rt for 18 h. Then it was diluted with CH_2Cl_2, and the organic phase was washed sequentially with 1 M HCl, a saturated aqueous solution of $NaHCO_3$, and brine, dried over anhydrous Na_2SO_4, filtered, and evaporated *in vacuo*. The crude product was subjected to column chromatography (SiO_2, *n*-hexane–EtOAc 3:1).

Compound **8 (*R*)-2-(4-methoxybenzyl)-1-cyano-3-oxoisoindolin-1-yl)methyl acetate**: White waxy solid

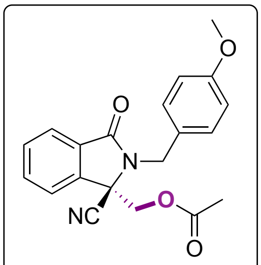

8

(17 mg, 50%). E.r. = 88.5:11.5. $[\alpha]_D^{25}$ −41 (*c* 1.0, $CHCl_3$). ^1H NMR (400 MHz, $CDCl_3$) δ/ppm: 7.97–7.94 (m, 1H), 7.72–7.63 (m, 3H), 7.36–7.32 (m, 2H), 6.89–6.85 (m, 2H), 5.12 (d, *J* = 15.6 Hz, 1H), 4.63 (d, *J* = 15.6 Hz, 1H), 4.42 (d, *J* = 11.5 Hz, 1H), 4.15 (d, *J* = 11.5 Hz, 1H), 3.80 (s, 3H), 1.93 (s, 3H). ^{13}C NMR (100 MHz, $CDCl_3$) δ/ppm: 169.2, 167.6, 159.4, 139.6, 133.2, 130.9, 130.6, 129.4, 128.2, 124.6, 122.7, 114.9, 114.1, 64.8, 61.1, 55.3, 44.4, 20.3. HRMS (APCI) *m/z*: $[M + H]^+$ calcd for $C_{20}H_{19}N_2O_4$ 351.1339, found 351.1339. HPLC (a Hypersil silica column (3 µm, 100 × 4.6 mm) used as a precolumn, which was connected *via* the standard blue PEEK capillary tubing (L 300 mm, ID 0.01", OD 1/16") to a Daicel® Chiralpak IA (5 µm, 250 × 4.6 mm), *n*-heptane–*i*-PrOH = 80:20, flow rate = 0.5 mL/min, λ = 230 nm, 25 °C) t_R = 28.68 min (major), 33.78 min (minor).

6.4 Oxidation of (*R*)-2a with DMP

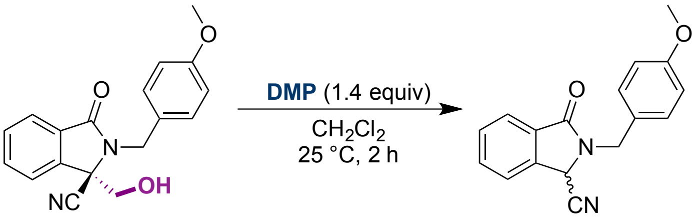

(*R*)-**2a**
e.r. 88:12

rac-**1a**
e.r. 50:50 (95%)

Dess-Martin periodinate (96 mg, 0.23 mmol) was added to a solution of (*R*)-**2a** (50 mg, 0.16 mmol) in CH_2Cl_2 (2 mL). The resulting mixture was stirred at room temperature for 2 h. After that, it was diluted with CH_2Cl_2. The organic phase was washed sequentially with a saturated aqueous solution of $Na_2S_2O_3$, a saturated aqueous solution of $NaHCO_3$, and brine, dried over anhydrous Na_2SO_4, filtered, and evaporated *in vacuo*.

Compound **1a** ***rac*-2-(4-methoxybenzyl)-3-oxoisoindoline-1-carbonitrile**:[48] White powder (43 mg, 95%). E.r. = 50:50. ^{1}H and ^{13}C NMR spectra correspond to *rac*-**1a** synthesized according to the general procedure. HPLC (a Hypersil silica column (3 µm, 100 × 4.6 mm) used as a precolumn, which was connected *via* the standard blue PEEK capillary tubing (L 300 mm, ID 0.01", OD 1/16") to a Phenomenex® Lux Amylose-1 (3 µm, 250 × 4.6 mm), *n*-heptane–*i*-PrOH = 80:20, flow rate = 0.5 mL/min, λ = 230 nm, 25 °C) t_R = 28.13 min, 35.87 min.

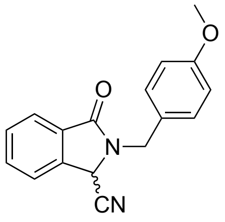

1a

6.5 *O*-Methylation of (*R*)-2b

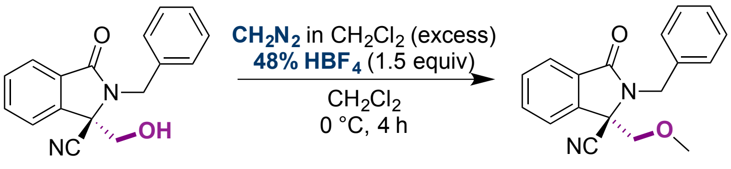

A diazomethane solution in CH_2Cl_2 (3 mL) was prepared from Diazald® (370 mg, 1.71 mmol), carbitol (1 mL), and 37% aqueous KOH (1.5 mL) according to the Aldrich® Technical Bulletin AL-180.[53] The resulting ice-cold solution of diazomethane in CH_2Cl_2 was transferred portionwise to the cooled and vigorously stirred emulsion of (*R*)-**2b** (20 mg, 0.072 mmol) and 48% aqueous HBF_4 (20 µL, 1.5 equiv) in CH_2Cl_2 by a Pasteur pipette with the fire-polished tip. Three or four additional batches of the above diazomethane solution were usually required for better conversion. Methylation protocols describing the use of $BF_3{\cdot}Et_2O$ or SiO_2 instead of 48% aqueous HBF_4 provided inferior yields of (*R*)-**9** in our hands. The crude product was purified by column chromatography (SiO_2, *n*-hexane–EtOAc = 2:1).

Compound **9** **(*R*)-2-benzyl-1-(methoxymethyl)-3-oxoisoindoline-1-carbonitrile:** White powder (11 mg, 52%). E.r. = 94:6. M.p. 142–145 °C. $[\alpha]_D^{25}$ −38 (*c* 0.5, $CHCl_3$). ^1H NMR (400 MHz, $CDCl_3$) δ/ppm: 7.96–7.94 (m, 1H), 7.72–7.62 (m, 3H), 7.42–7.40 (m, 2H), 7.37–7.30 (m, 3H), 5.25 (d, J = 15.7 Hz, 1H), 4.62 (d, J = 15.7 Hz, 1H), 3.57, 3.47 (q, AB, J_{AB} = 9.5 Hz, 2H). 3.10 (s, 3H). ^{13}C NMR (100 MHz, $CDCl_3$) δ/ppm: 167.8, 140.3, 136.9, 133.0, 130.6, 130.5, 128.6, 128.0, 127.8, 124.4, 123.1, 115.9, 74.9, 62.2, 59.3, 45.1. HRMS (APCI) *m/z*: $[M + H]^+$ calcd for $C_{18}H_{17}N_2O_2$ 293.1285, found 293.1286. HPLC (a Hypersil silica column (3 µm, 100 × 4.6 mm) used as a precolumn, which was connected *via* the standard blue PEEK capillary tubing (L 300 mm, ID 0.01", OD 1/16") to a Daicel® Chiralpak IA (5 µm, 250 × 4.6 mm), *n*-heptane–*i*-PrOH = 80:20, flow rate = 0.5 mL/min, λ = 230 nm, 25 °C) t_R = 18.20 min (major), 22.18 min (minor).

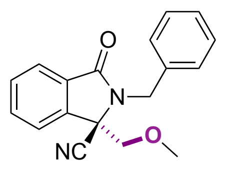

9

6.6 *O*-TBS and *N*-Boc Diprotection of (*R*)-2m

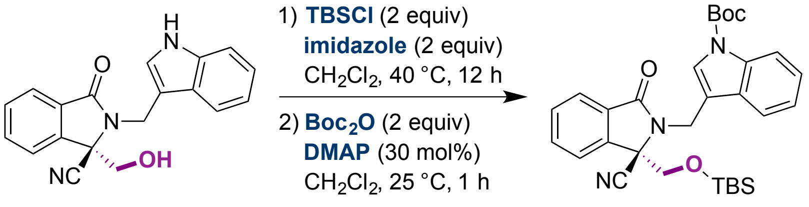

A modified literature procedure was used.[54] The mixture of (*R*)-**2m** (27 mg, 0.085 mmol), TBSCl (26 mg, 0.17 mmol), and imidazole (12 mg, 0.17 mmol) in dry CH_2Cl_2 was heated at 40 °C for 12 h under Ar (oil bath). The reaction mass was diluted with CH_2Cl_2, washed successively with a 10% aqueous solution of citric acid, a saturated aqueous solution of $NaHCO_3$, and brine, dried over anhydrous Na_2SO_4, filtered, and evaporated *in vacuo*. The residue was dissolved in CH_2Cl_2, di-*tert*-butyl dicarbonate (37 mg, 0.17 mmol), and DMAP (3 mg, 0.025 mmol) were added thereto. The solution was stirred at ambient temperature for 1 h. After that, the volatiles were evaporated *in vacuo*. The crude product was subjected to column chromatography (SiO_2, *n*-hexane–EtOAc 19:1).

Compound **10** ***tert*-butyl (*R*)-3-((1-(((*tert*-butyldimethylsilyl)oxy)methyl)-1-isocyano-3-oxoisoindolin-2-yl)methyl)-1*H*-indole-1-carboxylate**: Colorless glassy solid (21 mg, 46%). E.r. = 89:11. $[\alpha]_D^{25}$ −27 (*c* 1.0, CH_2Cl_2). ^{1}H NMR (400 MHz, $CDCl_3$) δ/ppm: 8.14 (d, *J* = 7.4 Hz, 1H), 7.95–7.93 (m, 1H), 7.75–7.68 (m, 3H), 7.67–7.58 (m, 2H), 7.35–7.31 (m, 1H), 7.27–7.23 (m, 1H), 5.27 (d, *J* = 15.7 Hz, 1H), 4.86 (d, *J* = 15.7 Hz, 1H), 3.96 (d, *J* = 9.8 Hz, 1H), 3.54 (d, *J* = 9.8 Hz, 1H), 1.68 (s, 9H), 0.69 (s, 9H), −0.15 (s, 3H), −0.17 (s, 3H). ^{13}C NMR (100 MHz, $CDCl_3$) δ/ppm: 167.7, 149.4, 141.1, 135.6, 132.8, 130.6, 130.3, 129.2, 125.3, 124.9, 124.3, 123.1, 123.0, 119.6, 116.0, 115.9, 115.3, 84.0, 66.1, 63.3, 35.9, 28.2, 25.4, 18.0, –5.9, –6.0. HRMS (APCI) *m/z*: $[M + H]^+$ calcd for $C_{30}H_{38}N_3O_4Si$ 532.2626, found 532.2621. HPLC (a Hypersil silica column (3 µm, 100 × 4.6 mm) used as a precolumn, which was connected *via* the standard blue PEEK capillary tubing (L 300 mm, ID 0.01”, OD 1/16”) to a Daicel® Chiralpak IA (5 µm, 250 × 4.6 mm), *n*-heptane–*i*-PrOH = 90:10, flow rate = 0.5 mL/min, λ = 230 nm, 25 °C) t_R = 12.76 min (major), 14.22 min (minor).

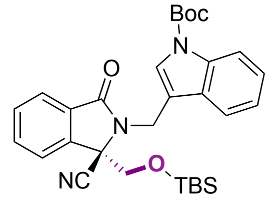

10

6.7 Suzuki Cross-Coupling of (*R*)-2zb

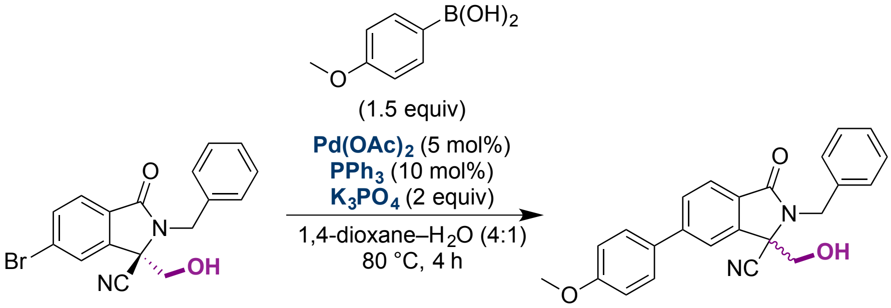

(*R*)-**2zb** e.r. 91.5:8.5 | *rac*-**11** e.r. 50:50 (60%)

A modified literature procedure was used.[55] Compound (*R*)-**2zb** (50 mg, 0.104 mmol), K_3PO_4 (44 mg, 0.21 mmol), triphenylphosphine (3 mg, 0.011 mmol), and 4-methoxyphenyl boronic acid (24 mg, 0.16 mmol) were loaded into the Schlenk tube and dissolved in 1,4-dioxane/water 4:1 (3 mL). The solution was purged with Ar for 15 min, then palladium(II) acetate (1.2 mg, 0.005 mmol) was added, and the mixture was purged with Ar for a further 15 min. The reaction mass was heated at 80 °C for 4 h (oil bath). It was quenched with water afterward and repetitively extracted with EtOAc. The combined organic phases were washed with brine, dried over anhydrous Na_2SO_4, filtered, and evaporated *in vacuo*. The product was purified by column chromatography (SiO_2, *n*-hexane–EtOAc = 2:1).

Compound **11** ***rac*-2-benzyl-1-(hydroxymethyl)-6-(4-methoxyphenyl)-3-oxoisoindoline-1-carbonitrile**:

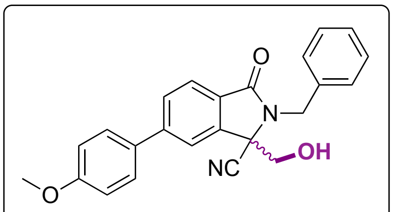

11

White solid (24 mg, 60%). E.r. 50:50. M.p. 45–47 °C. ^{1}H NMR (400 MHz, $CDCl_3$) δ/ppm: 7.96 (d, *J* = 7.9 Hz, 1H), 7.81–7.78 (m, 2H), 7.60–7.56 (m, 2H), 7.49–7.47 (m, 2H), 7.41–7.34 (m, 3H), 7.04–7.00 (m, 2H), 5.37 (d, *J* = 15.7 Hz, 1H), 4.53 (d, *J* = 15.7 Hz, 1H), 3.98–3.94 (m, 1H), 3.88 (s, 3H), 3.80–3.77 (m, 1H), 1.72 (br s, 1H). ^{13}C NMR (100 MHz, $CDCl_3$) δ/ppm: 168.0, 160.2, 146.4, 140.7, 136.9, 131.8, 129.24, 129.19, 128.8, 128.6, 128.4, 128.0, 124.9, 120.5, 115.7, 114.6, 65.5, 63.8, 55.4, 44.8. HRMS (APCI) *m/z*: $[M + H]^+$ calcd for $C_{24}H_{21}N_2O_3$ 385.1547, found 385.1545. HPLC (a Hypersil silica column (3 µm, 100 × 4.6 mm) used as a precolumn, which was connected *via* the standard blue PEEK capillary tubing (L 300 mm, ID 0.01", OD 1/16") to a Daicel® Chiralpak IA (5 µm, 250 × 4.6 mm), *n*-heptane–*i*-PrOH = 80:20, flow rate = 0.5 mL/min, λ = 230 nm, 25 °C) t_R = 26.04 min, 27.81 min.

7. X-Ray Single-Crystal Analysis

Compound (*R*)-2v:

Data were collected on a Rigaku Oxford Diffraction Synergy X-ray diffractometer at 120(2) K using Hybrid Pixel Array Detector HyPix-6000HE and monochromated Cu-Kα radiation (λ = 1.54 Å) from MicroMax-007HF DW 1.2 kW rotating anode (Rigaku Corp.). CrysAlisPro 1.171.41.115a (Rigaku Oxford Diffraction, 2021) software package was used for data collection and data reduction. The structures were solved by SHELXT,[56] and refined using SHELXL programs.[57]

Compound (*R*)-**2v** crystallized from $CHCl_3$ in an orthorhombic space group $P2_12_12_1$ and one molecule in the asymmetric unit. The absolute configuration (*R*) on C1 carbon was confirmed with a Flack parameter of 0.046(21) from 1022 selected quotients (Parson's method).[58] **CCDC** 2298018 contains the supplementary crystallographic data for compound (*R*)-**2v**.[59]

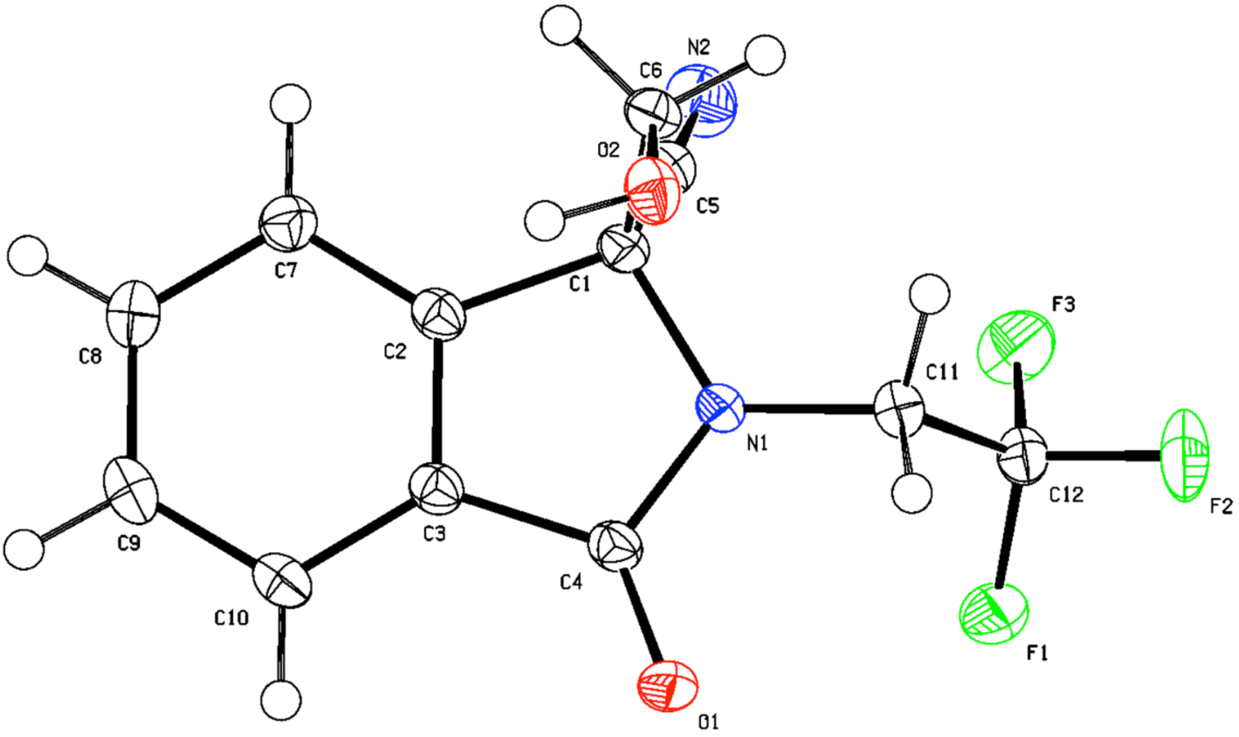

Figure S22: Displacement ellipsoid representation of (*R*)-**2v** (**CCDC** 2298018) at 50% probability.

Table S5: Crystal Data and Structure Refinement for Compound (*R*)-**2v** (**CCDC** 2298018).

Identification code	0607
Empirical formula	C12 H9 F3 N2 O2
Formula weight	270.21
Temperature	120(2) K
Wavelength	1.54184 A
Crystal system, space group	Orthorhombic, P2(1)2(1)2(1)
Unit cell dimensions	a = 7.70269(10) A alpha = 90 deg.<br>b = 7.74352(11) A beta = 90 deg.<br>c = 19.8083(2) A gamma = 90 deg.
Volume	1181.49(3) A^3
Z, Calculated density	4, 1.519 Mg/m^3
Absorption coefficient	1.196 mm^-1
F(000)	552
Crystal size	0.300 x 0.120 x 0.060 mm
Theta range for data collection	4.464 to 77.229 deg.
Limiting indices	-9<=h<=9, -9<=k<=8, -24<=l<=25
Reflections collected / unique	13364 / 2482 [R(int) = 0.0139]
Completeness to theta = 67.684	99.6 %
Absorption correction	Semi-empirical from equivalents
Max. and min. transmission	1.00000 and 0.78927
Refinement method	Full-matrix least-squares on F^2
Data / restraints / parameters	2482 / 0 / 175
Goodness-of-fit on F^2	1.053
Final R indices [I>2sigma(I)]	R1 = 0.0230, wR2 = 0.0589
R indices (all data)	R1 = 0.0230, wR2 = 0.0589
Absolute structure parameter	0.05(2)
Extinction coefficient	0.0065(9)
Largest diff. peak and hole	0.208 and -0.192 e.A^-3

8. Copies of the Product NMR Spectra

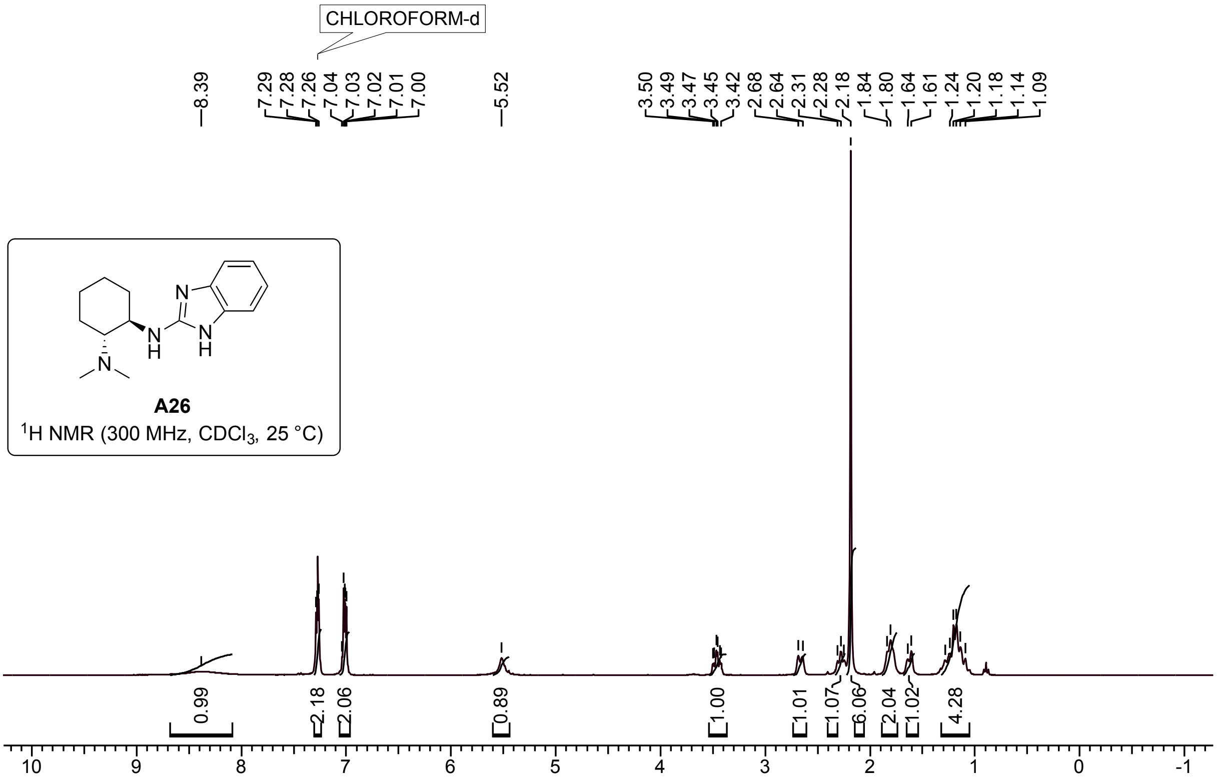

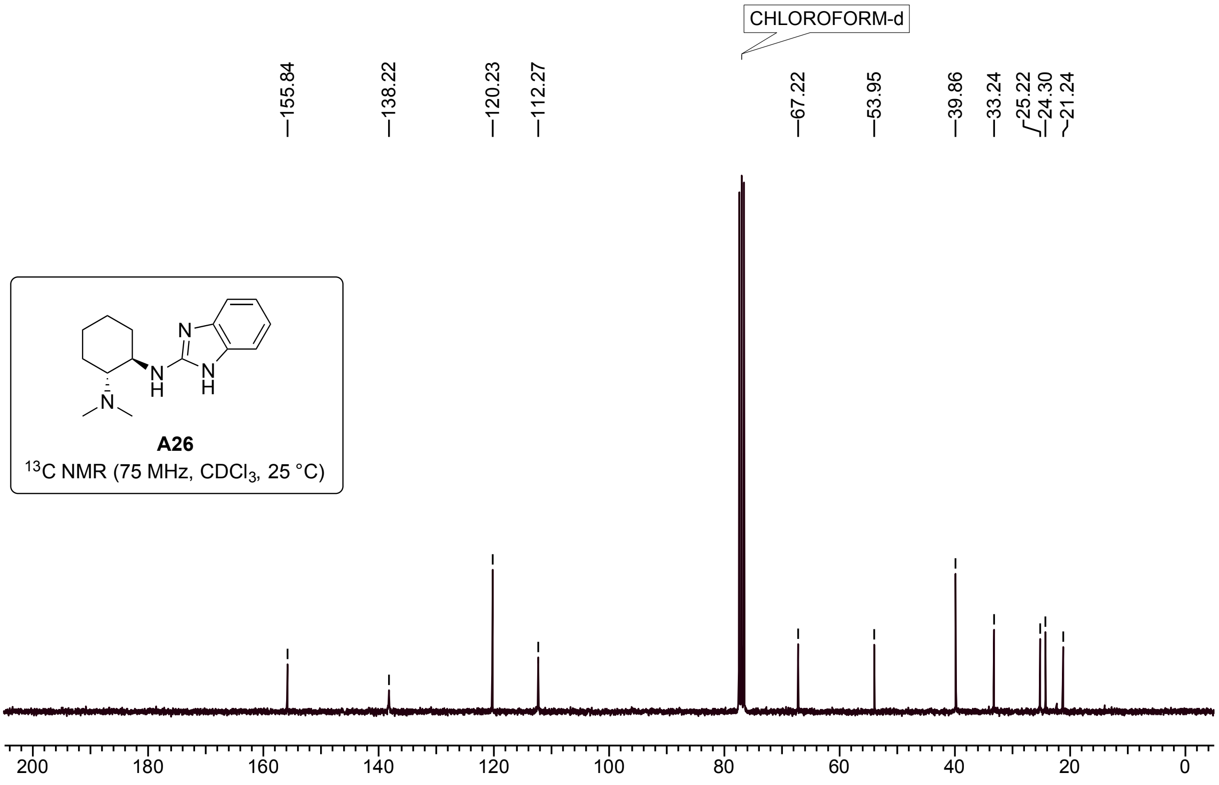

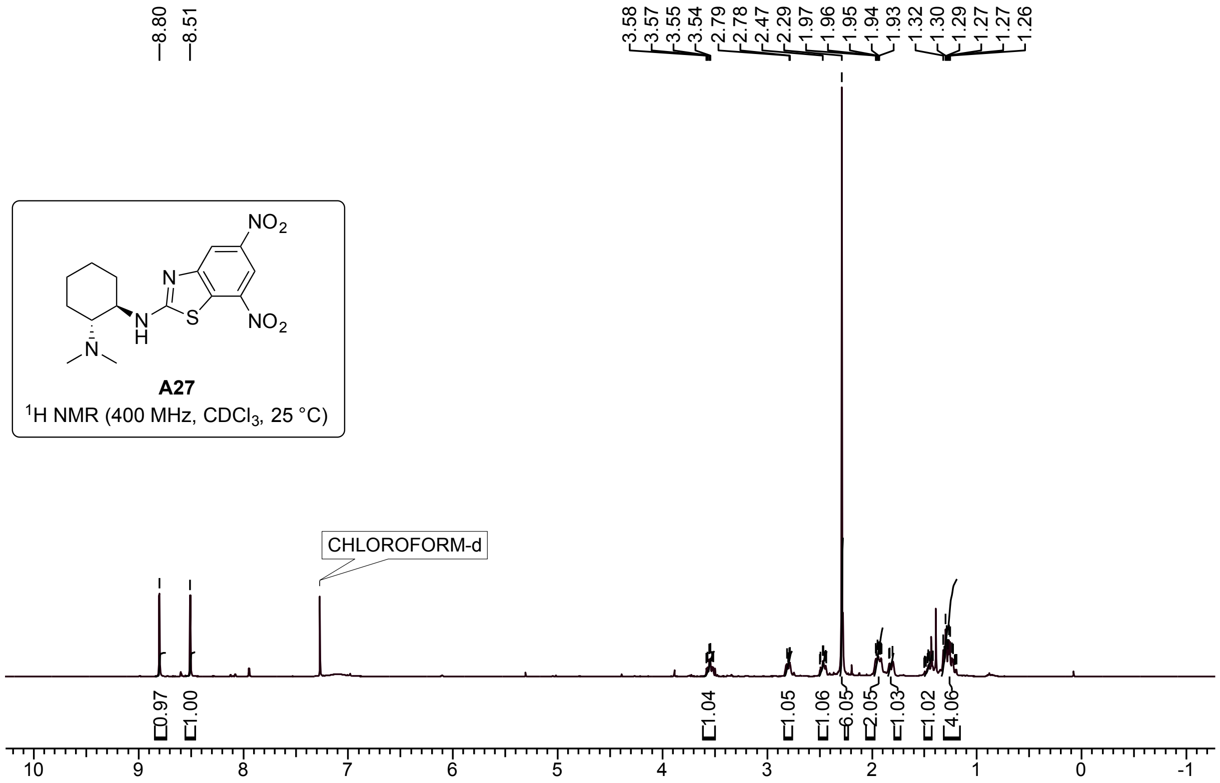

8.80
8.51
3.58
3.57
3.55
3.54
2.79
2.78
2.47
2.29
1.97
1.96
1.95
1.94
1.93
1.32
1.30
1.29
1.27
1.27
1.26
NO2
N
N
H
S
NO2
N
A27
1H NMR (400 MHz, CDCl3, 25 °C)
CHLOROFORM-d
0.97
1.00
1.04
1.05
1.06
6.05
2.05
1.03
1.02
4.06
10
9
8
7
6
5
4
3
2
1
0
-1

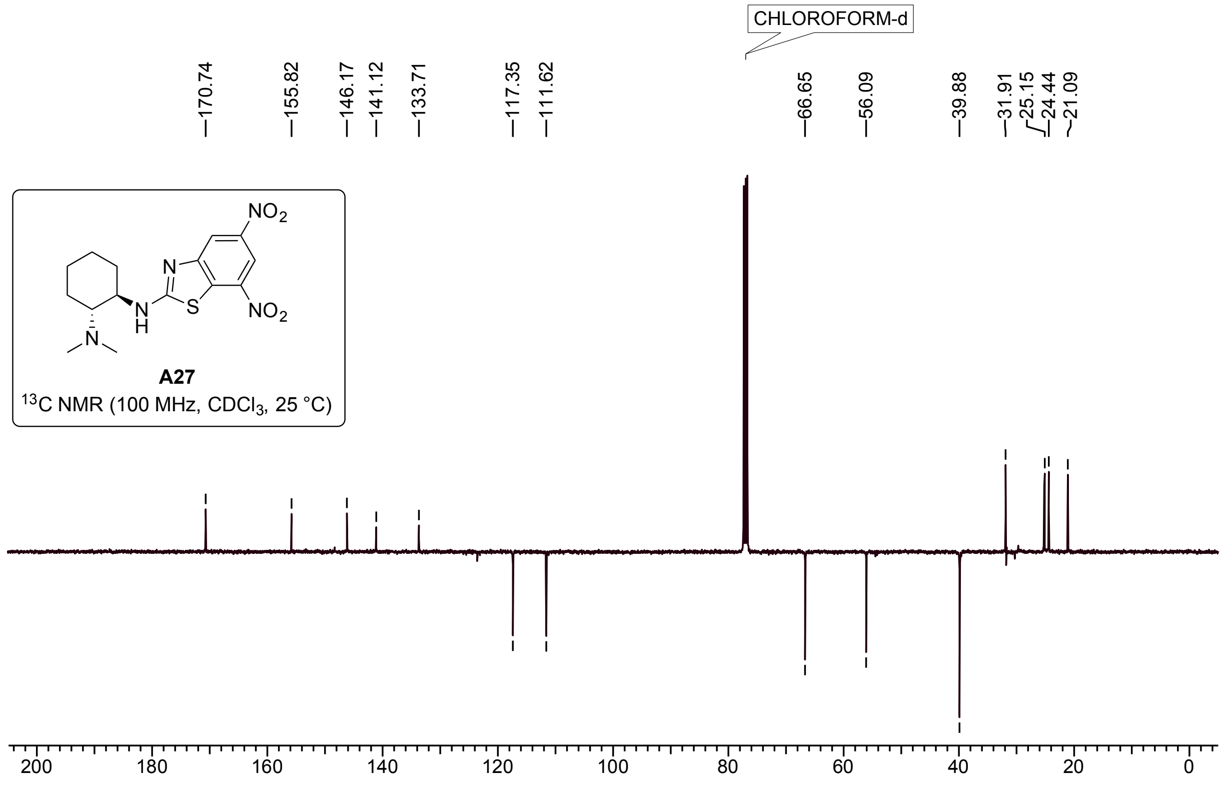

CHLOROFORM-d
170.74
155.82
146.17
141.12
133.71
117.35
111.62
66.65
56.09
39.88
31.91
25.15
24.44
21.09
NO2
N
N
H
S
NO2
N
A27
13C NMR (100 MHz, CDCl3, 25 °C)
200
180
160
140
120
100
80
60
40
20
0

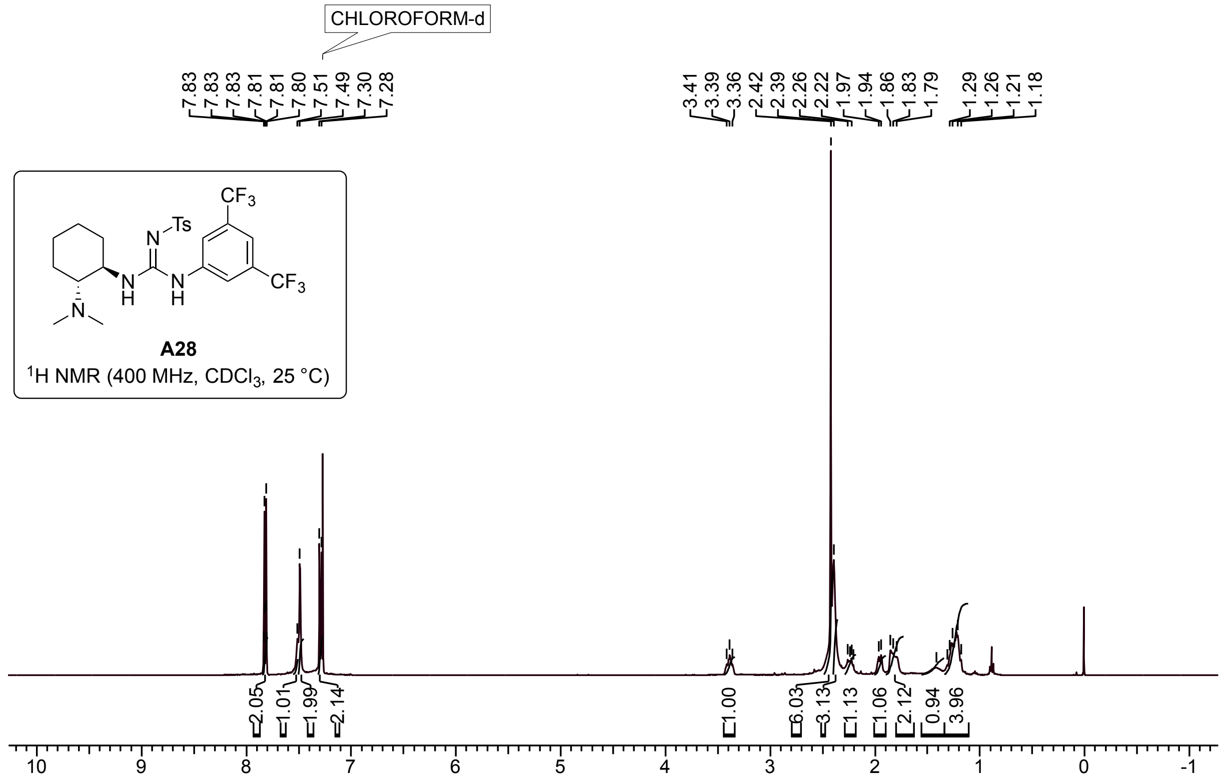
CHLOROFORM-d
7.83 7.83 7.83 7.81 7.81 7.80 7.51 7.49 7.30 7.28
3.41 3.39 3.36 2.42 2.39 2.26 2.22 1.97 1.94 1.86 1.83 1.79 1.29 1.26 1.21 1.18
A28
1H NMR (400 MHz, CDCl3, 25 °C)
2.05 1.01 1.99 2.14 1.00 6.03 3.13 1.13 1.06 2.12 0.94 3.96
10 9 8 7 6 5 4 3 2 1 0 -1

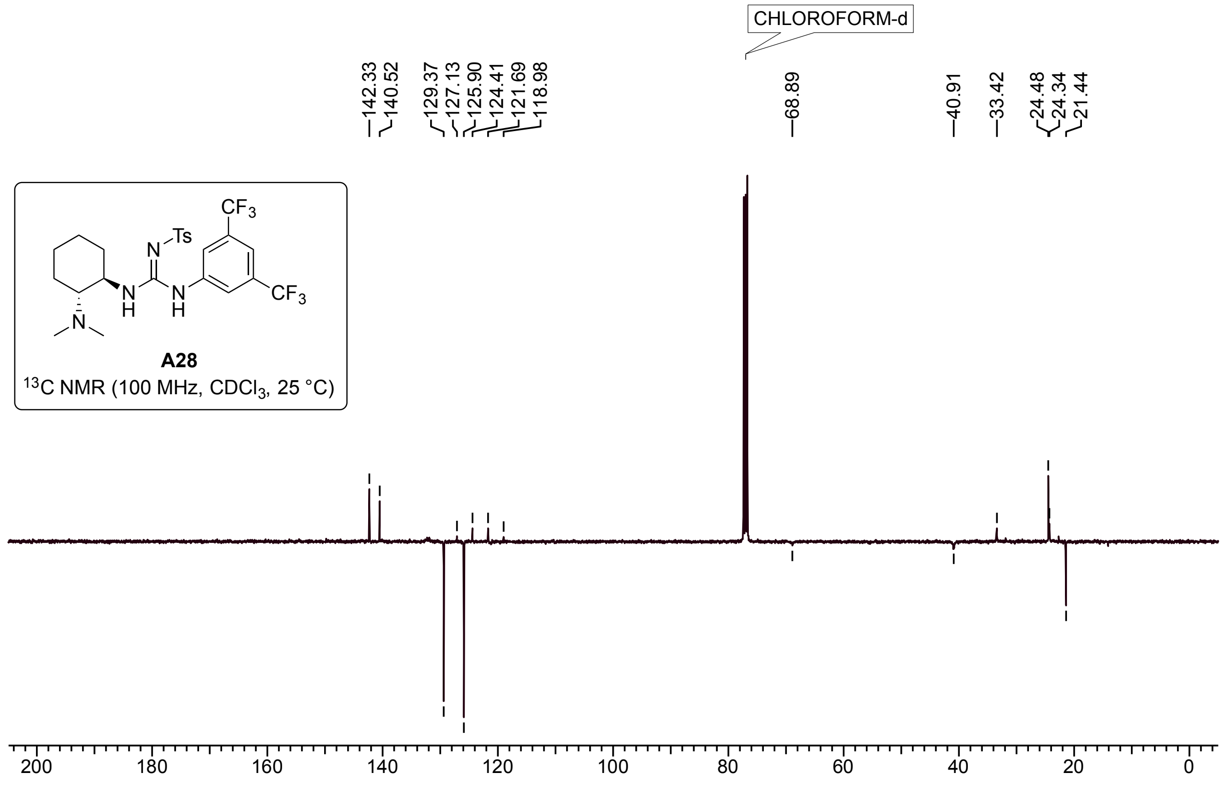
CHLOROFORM-d
142.33 140.52 129.37 127.13 125.90 124.41 121.69 118.98 68.89 40.91 33.42 24.48 24.34 21.44
A28
13C NMR (100 MHz, CDCl3, 25 °C)
200 180 160 140 120 100 80 60 40 20 0

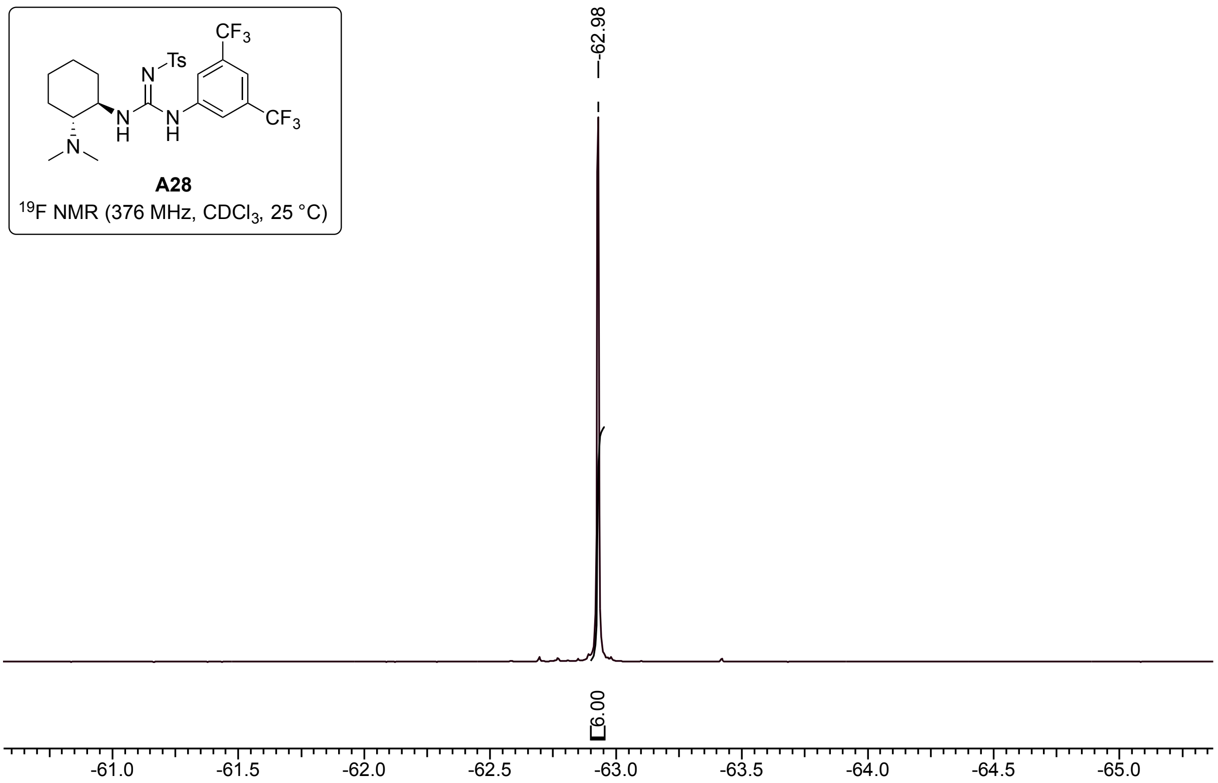
A28
19F NMR (376 MHz, CDCl3, 25 °C)
-62.98
6.00
-61.0 -61.5 -62.0 -62.5 -63.0 -63.5 -64.0 -64.5 -65.0

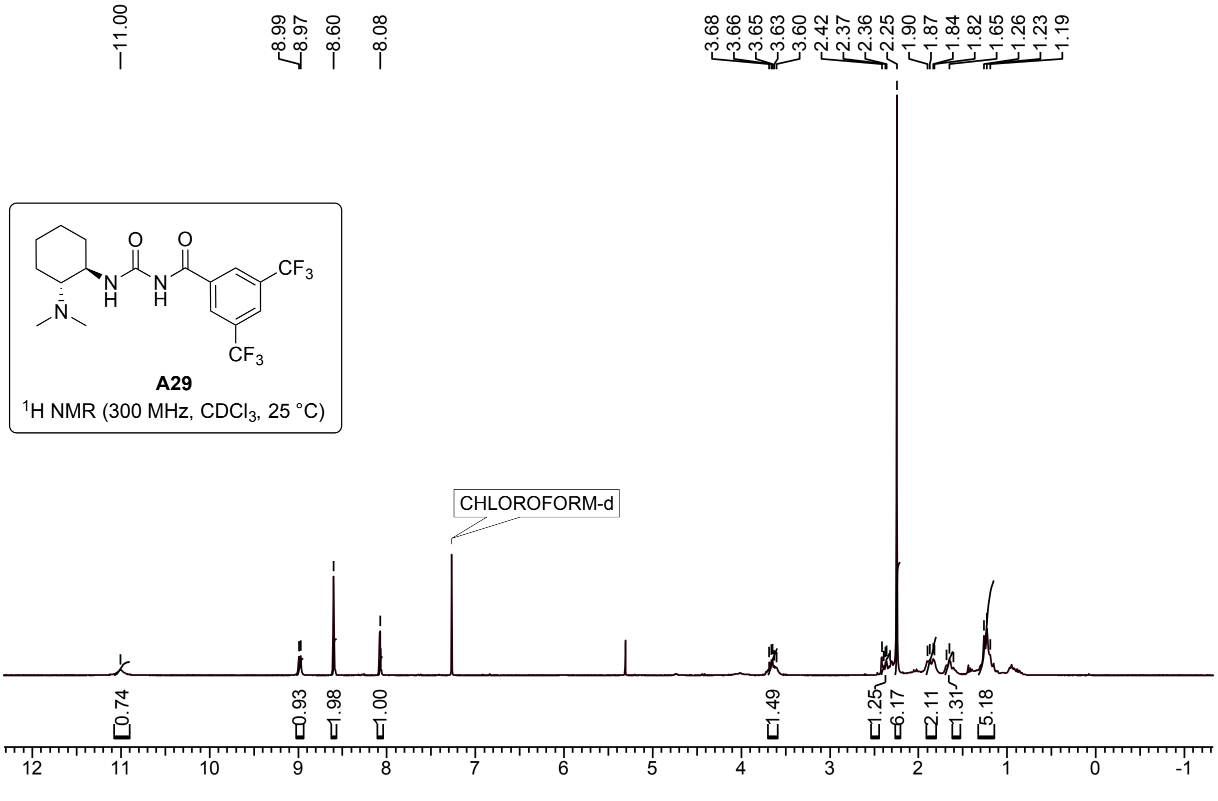
A29
1H NMR (300 MHz, CDCl3, 25 °C)
11.00
8.99 8.97 8.60 8.08
3.68 3.66 3.65 3.63 3.60 2.42 2.37 2.36 2.25 1.90 1.87 1.84 1.82 1.65 1.26 1.23 1.19
CHLOROFORM-d
0.74 0.93 1.98 1.00 1.49 1.25 6.17 2.11 1.31 5.18
12 11 10 9 8 7 6 5 4 3 2 1 0 -1

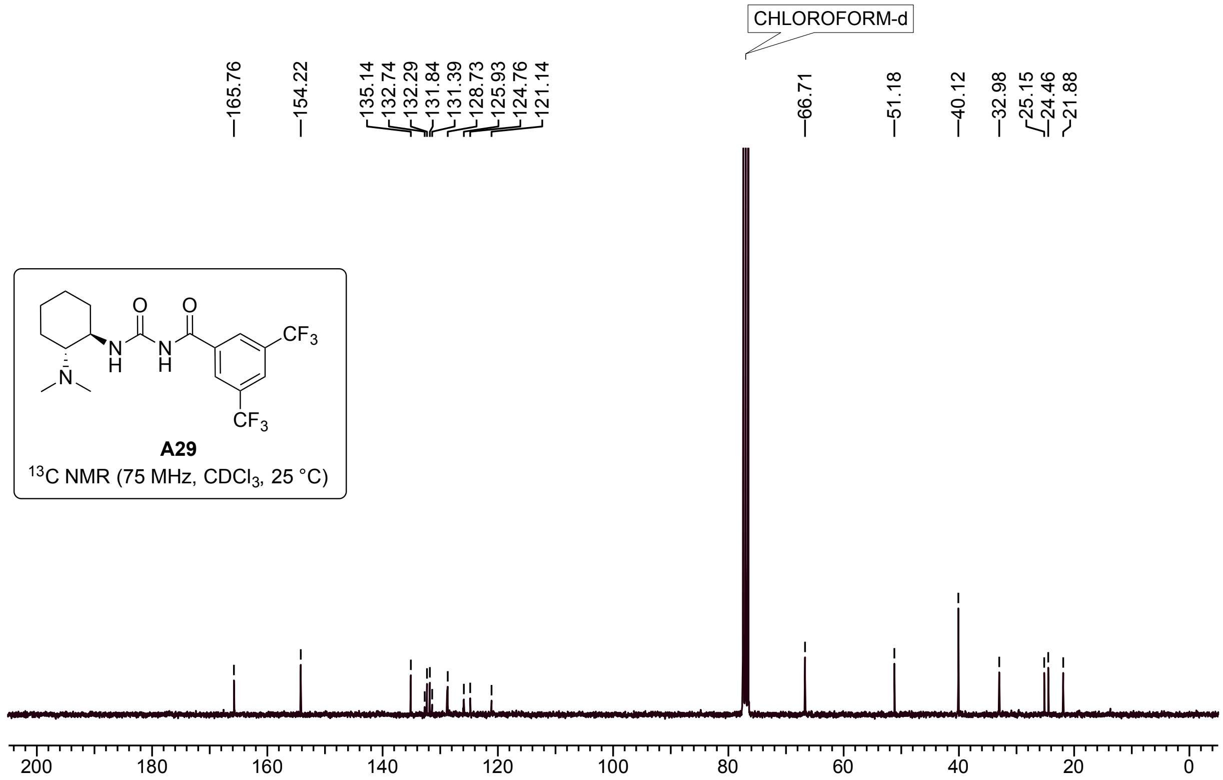
CHLOROFORM-d
165.76
154.22
135.14
132.74
132.29
131.84
131.39
128.73
125.93
124.76
121.14
66.71
51.18
40.12
32.98
25.15
24.46
21.88
CF3
CF3
A29
13C NMR (75 MHz, CDCl3, 25 °C)
200
180
160
140
120
100
80
60
40
20
0

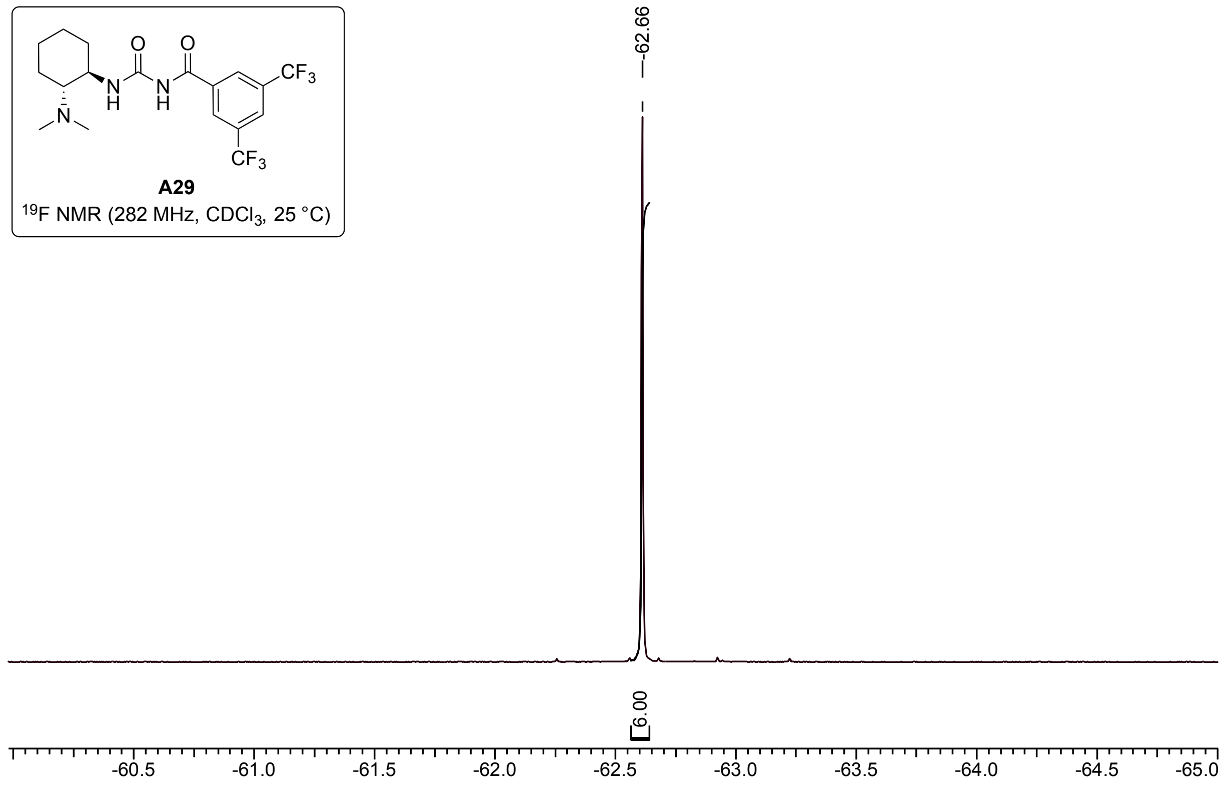
-62.66
CF3
CF3
A29
19F NMR (282 MHz, CDCl3, 25 °C)
6.00
-60.5
-61.0
-61.5
-62.0
-62.5
-63.0
-63.5
-64.0
-64.5
-65.0

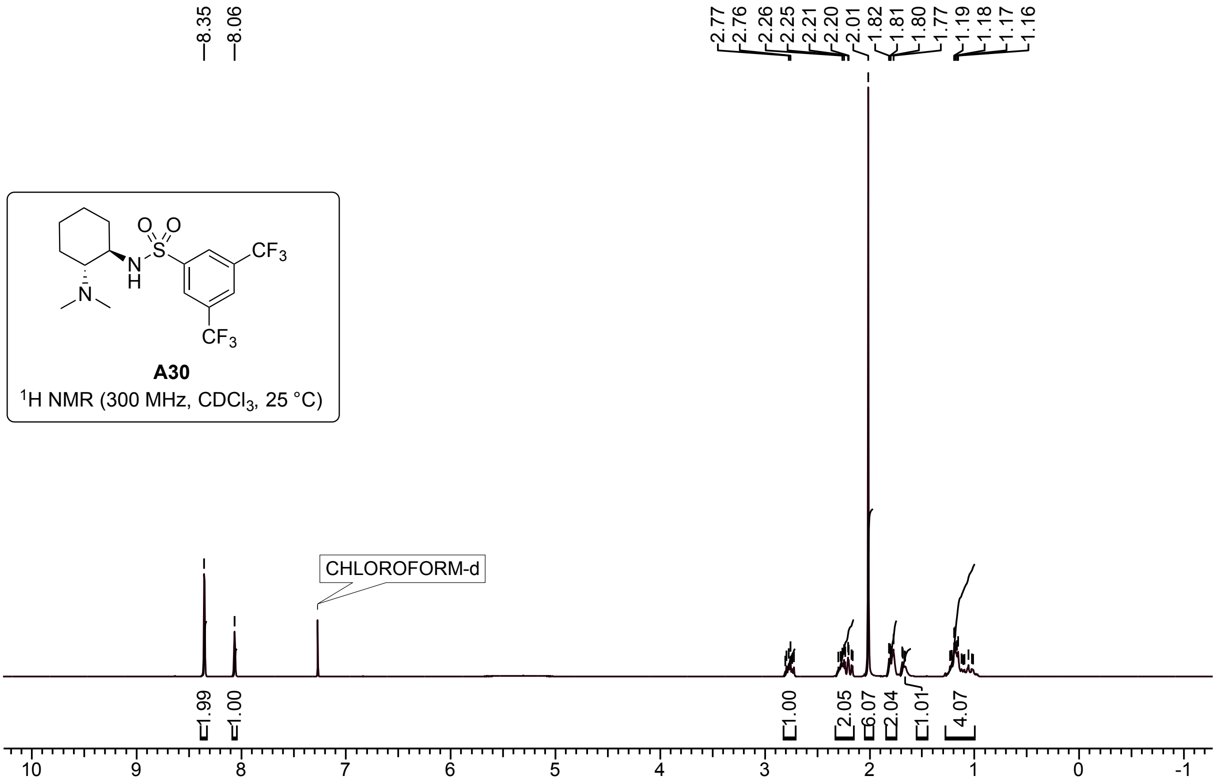

8.35
8.06
2.77
2.76
2.26
2.25
2.21
2.20
2.01
1.82
1.81
1.80
1.77
1.19
1.18
1.17
1.16
CHLOROFORM-d
1.99
1.00
1.00
2.05
6.07
2.04
1.01
4.07
10
9
8
7
6
5
4
3
2
1
0
-1
CF3
N
H
S
O
O
N
A30
1H NMR (300 MHz, CDCl3, 25 °C)

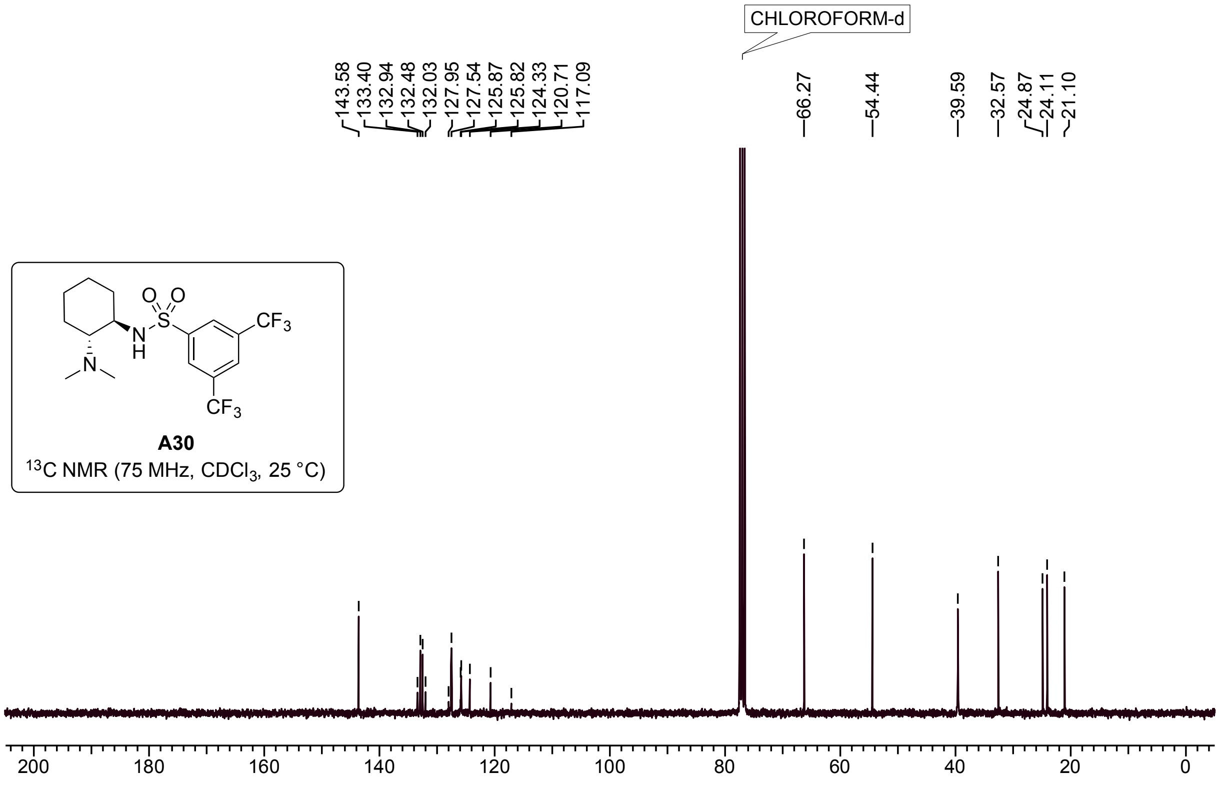

CHLOROFORM-d
143.58
133.40
132.94
132.48
132.03
127.95
127.54
125.87
125.82
124.33
120.71
117.09
66.27
54.44
39.59
32.57
24.87
24.11
21.10
200
180
160
140
120
100
80
60
40
20
0
CF3
N
H
S
O
O
N
A30
13C NMR (75 MHz, CDCl3, 25 °C)

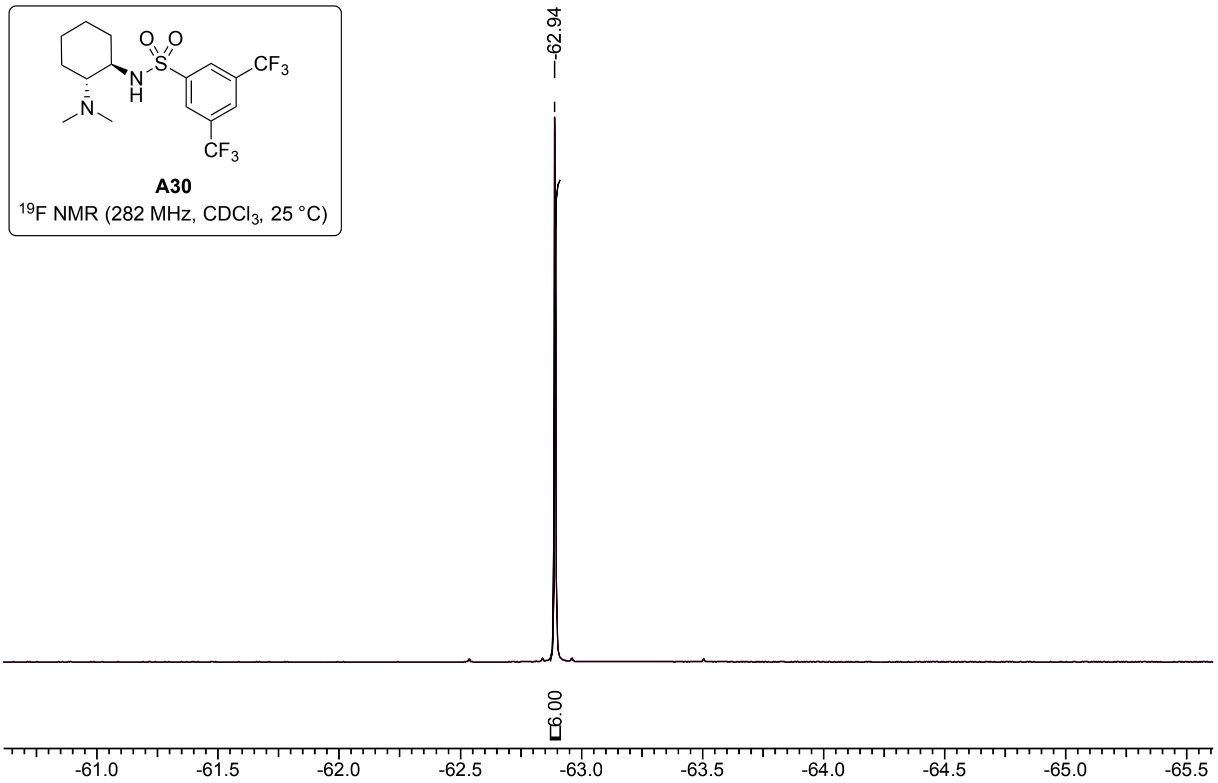

A30
19F NMR (282 MHz, CDCl3, 25 °C)
-62.94
6.00
-61.0 -61.5 -62.0 -62.5 -63.0 -63.5 -64.0 -64.5 -65.0 -65.5

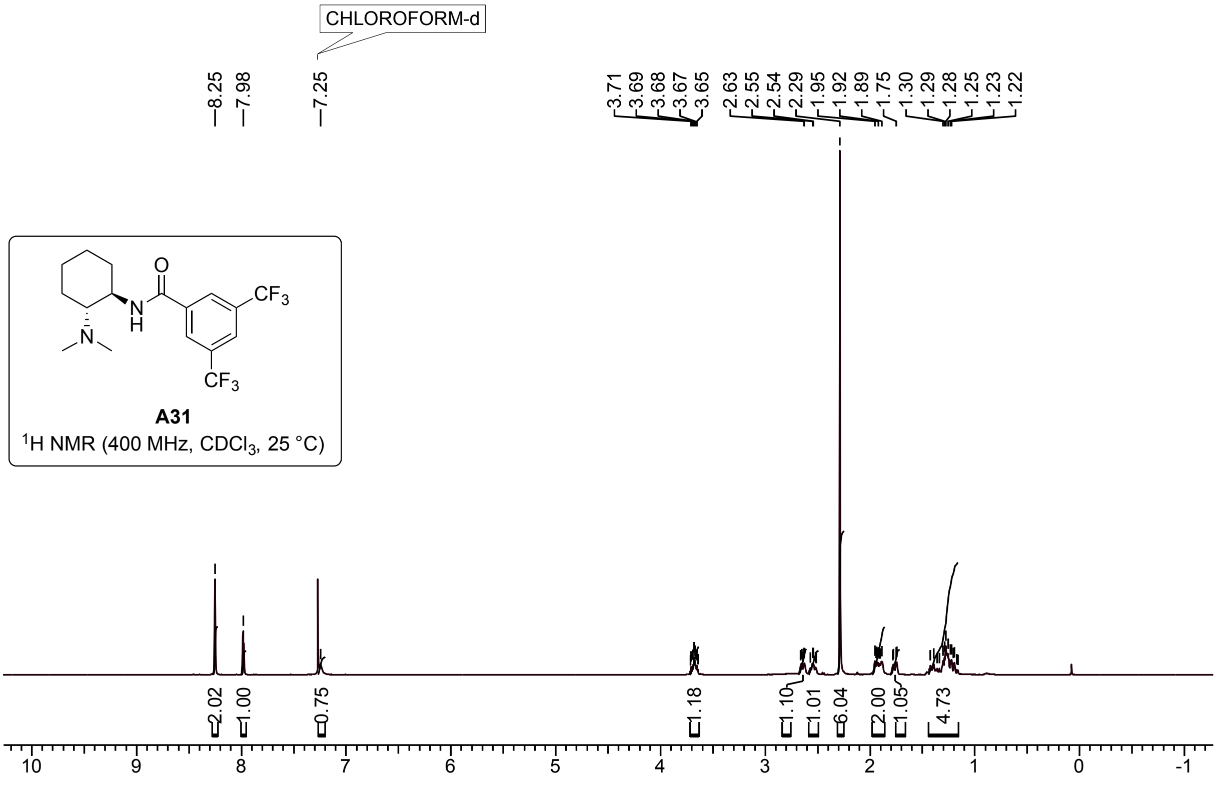

A31
1H NMR (400 MHz, CDCl3, 25 °C)
CHLOROFORM-d
8.25 7.98 7.25
3.71 3.69 3.68 3.67 3.65 2.63 2.55 2.54 2.29 1.95 1.92 1.89 1.75 1.30 1.29 1.28 1.25 1.23 1.22
2.02 1.00 0.75 1.18 1.10 1.01 6.04 2.00 1.05 4.73
10 9 8 7 6 5 4 3 2 1 0 -1

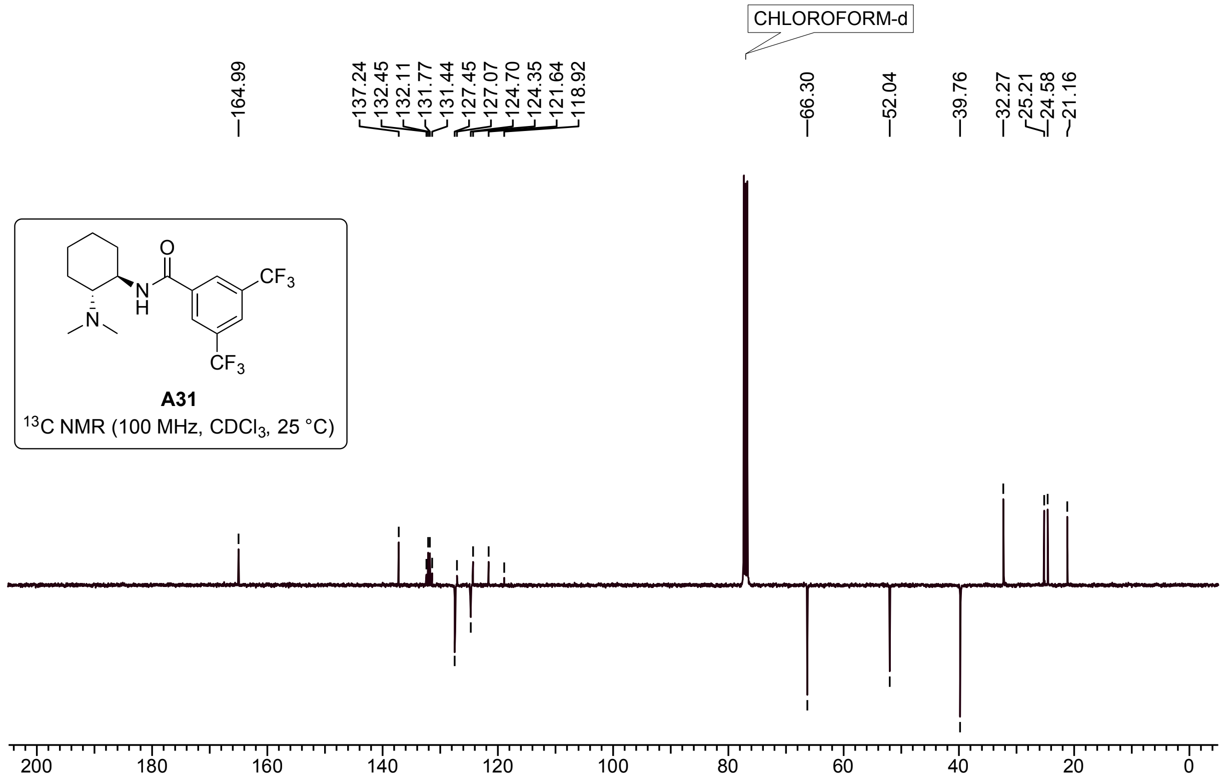
CHLOROFORM-d
164.99
137.24
132.45
132.11
131.77
131.44
127.45
127.07
124.70
124.35
121.64
118.92
66.30
52.04
39.76
32.27
25.21
24.58
21.16
CF3
O
N
H
N
CF3
A31
13C NMR (100 MHz, CDCl3, 25 °C)
200
180
160
140
120
100
80
60
40
20
0

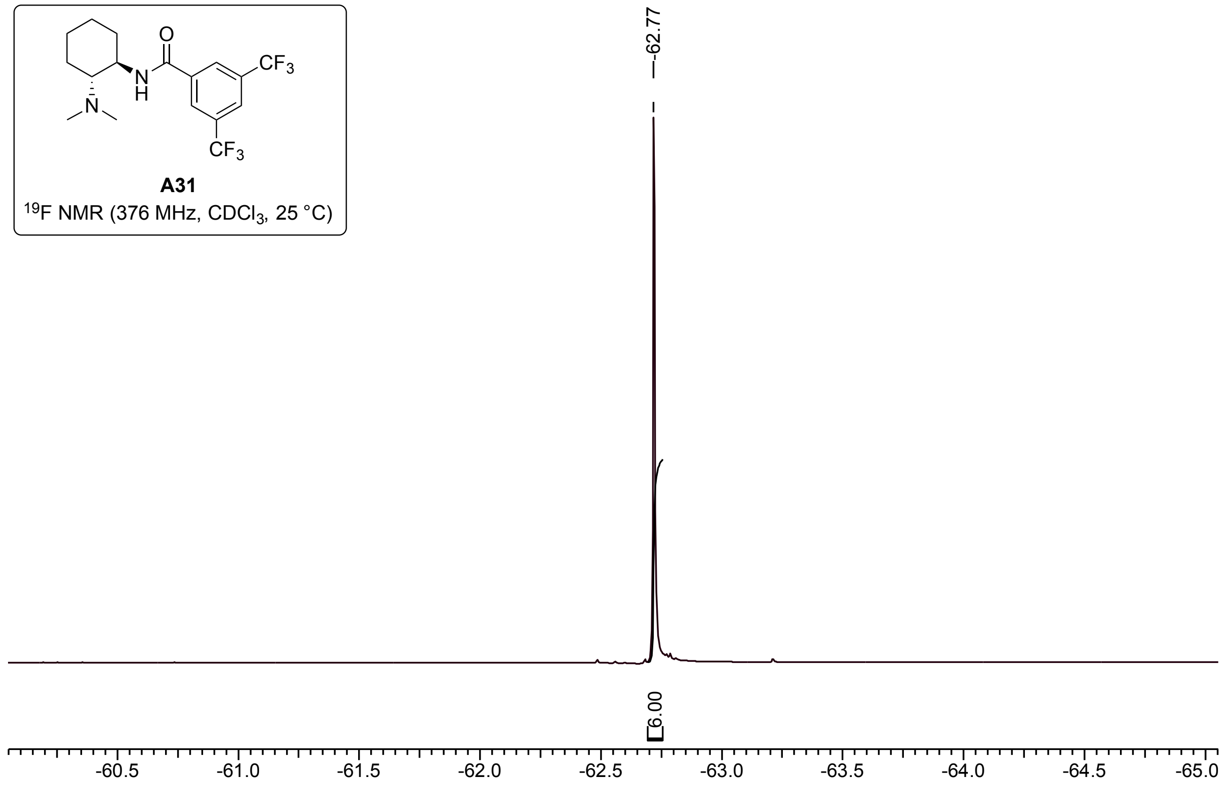
-62.77
CF3
O
N
H
N
CF3
A31
19F NMR (376 MHz, CDCl3, 25 °C)
6.00
-60.5
-61.0
-61.5
-62.0
-62.5
-63.0
-63.5
-64.0
-64.5
-65.0

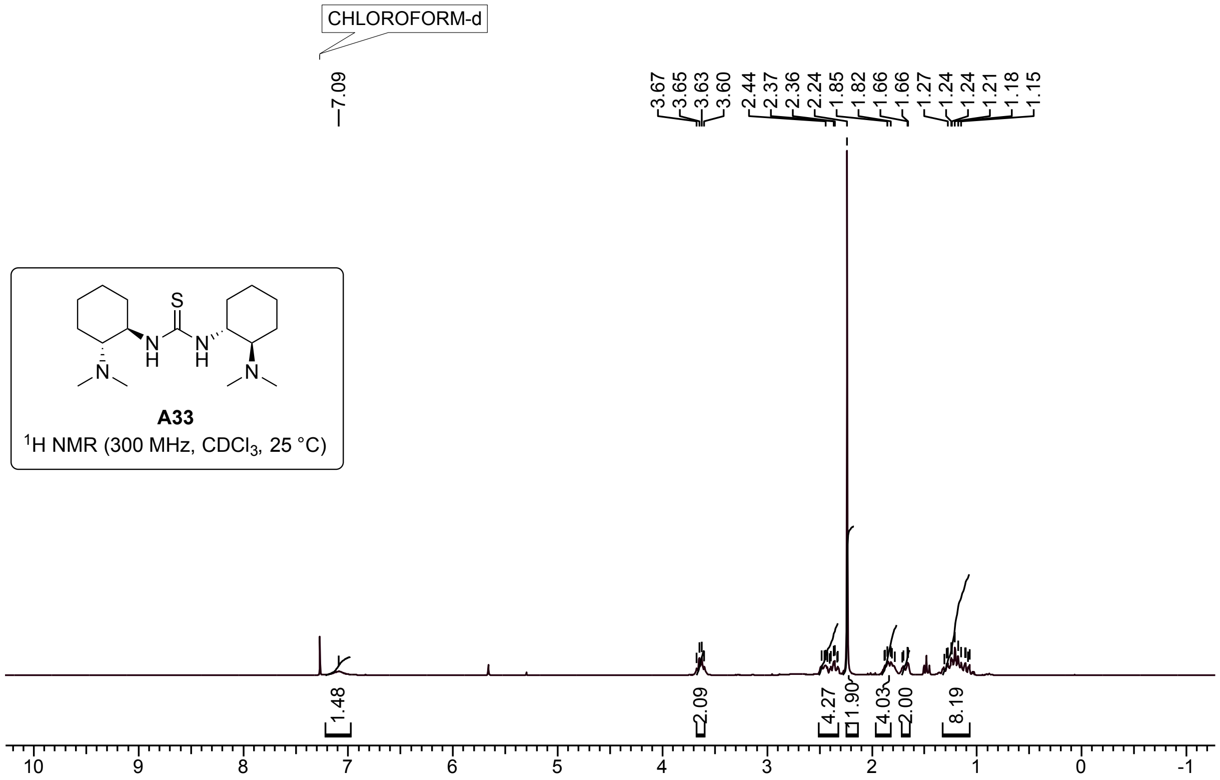
CHLOROFORM-d
7.09
3.67 3.65 3.63 3.60 2.44 2.37 2.36 2.24 1.85 1.82 1.66 1.66 1.27 1.24 1.24 1.21 1.18 1.15
1.48
2.09
4.27
1.90
4.03
2.00
8.19
10 9 8 7 6 5 4 3 2 1 0 -1
S
N
H
N
H
N
N
A33
1H NMR (300 MHz, CDCl3, 25 °C)

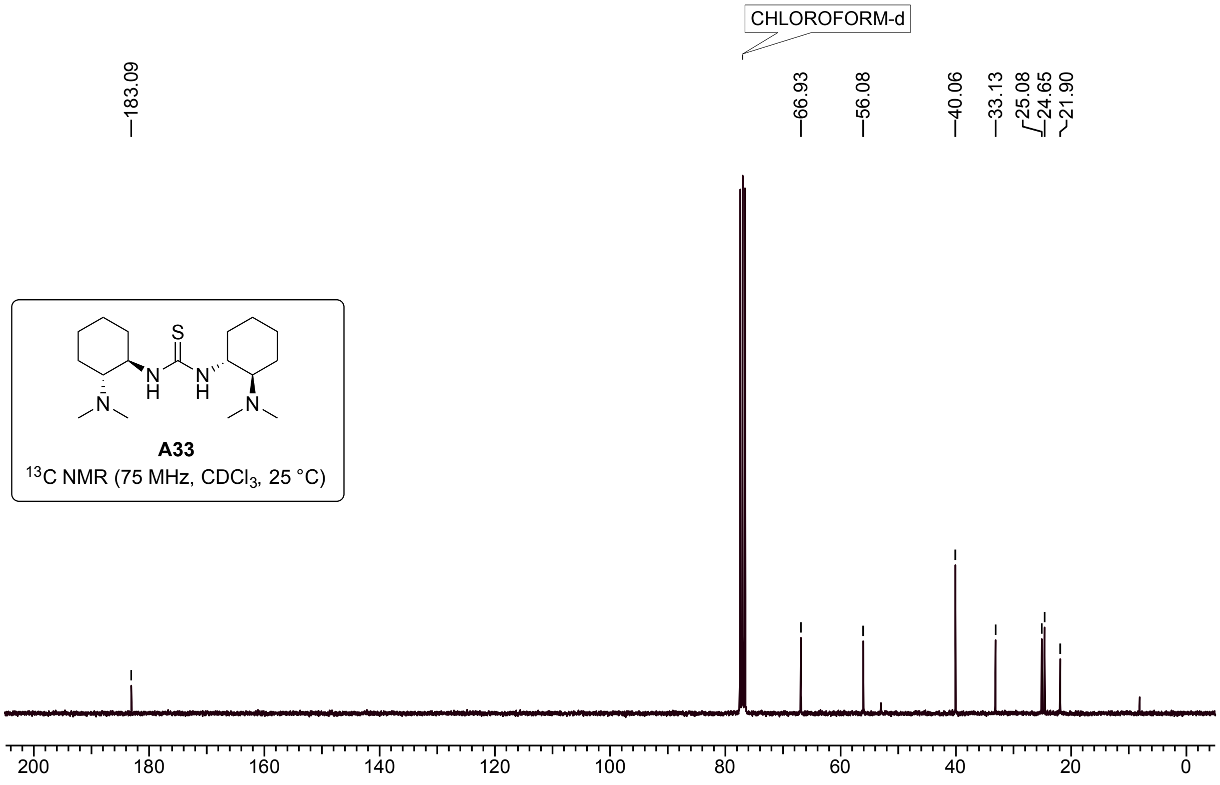
CHLOROFORM-d
183.09
66.93
56.08
40.06
33.13
25.08
24.65
21.90
200 180 160 140 120 100 80 60 40 20 0
S
N
H
N
H
N
N
A33
13C NMR (75 MHz, CDCl3, 25 °C)

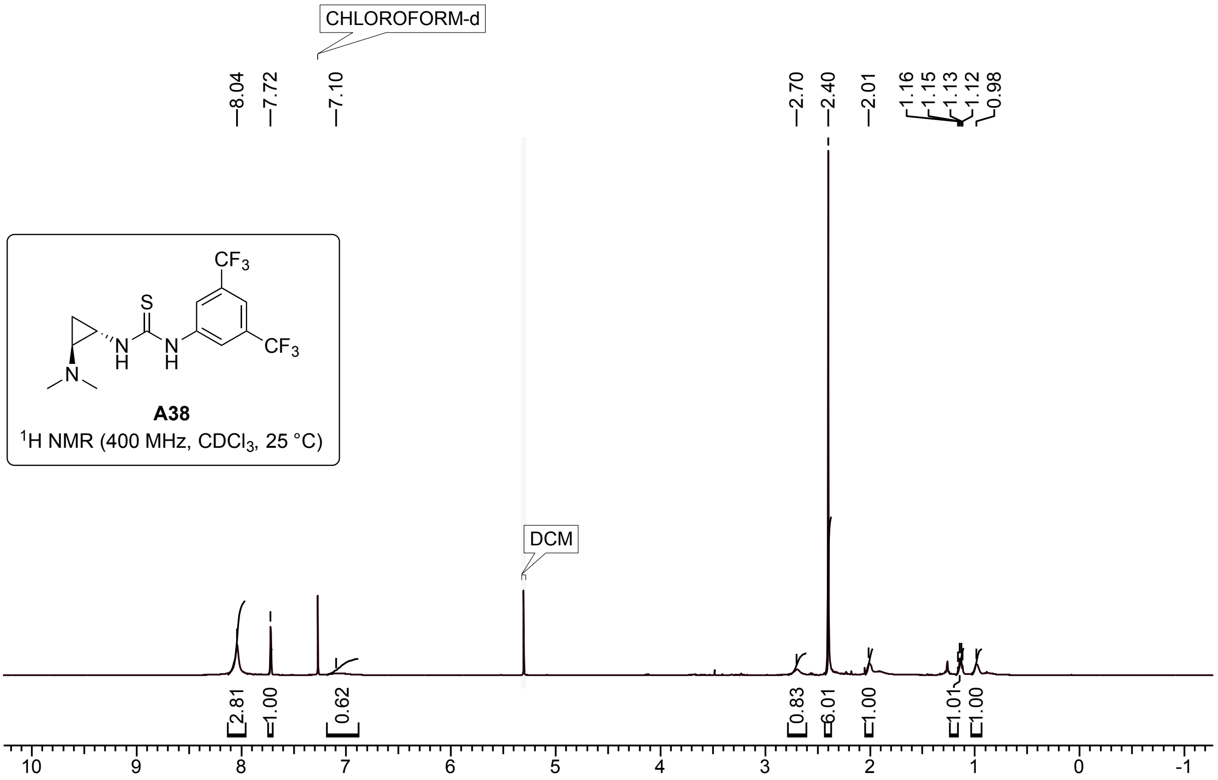
CHLOROFORM-d
8.04
7.72
7.10
2.70
2.40
2.01
1.16
1.15
1.13
1.12
0.98
CF3
S
N
H
N
H
CF3
N
A38
1H NMR (400 MHz, CDCl3, 25 °C)
DCM
2.81
1.00
0.62
0.83
6.01
1.00
1.01
1.00
10
9
8
7
6
5
4
3
2
1
0
-1

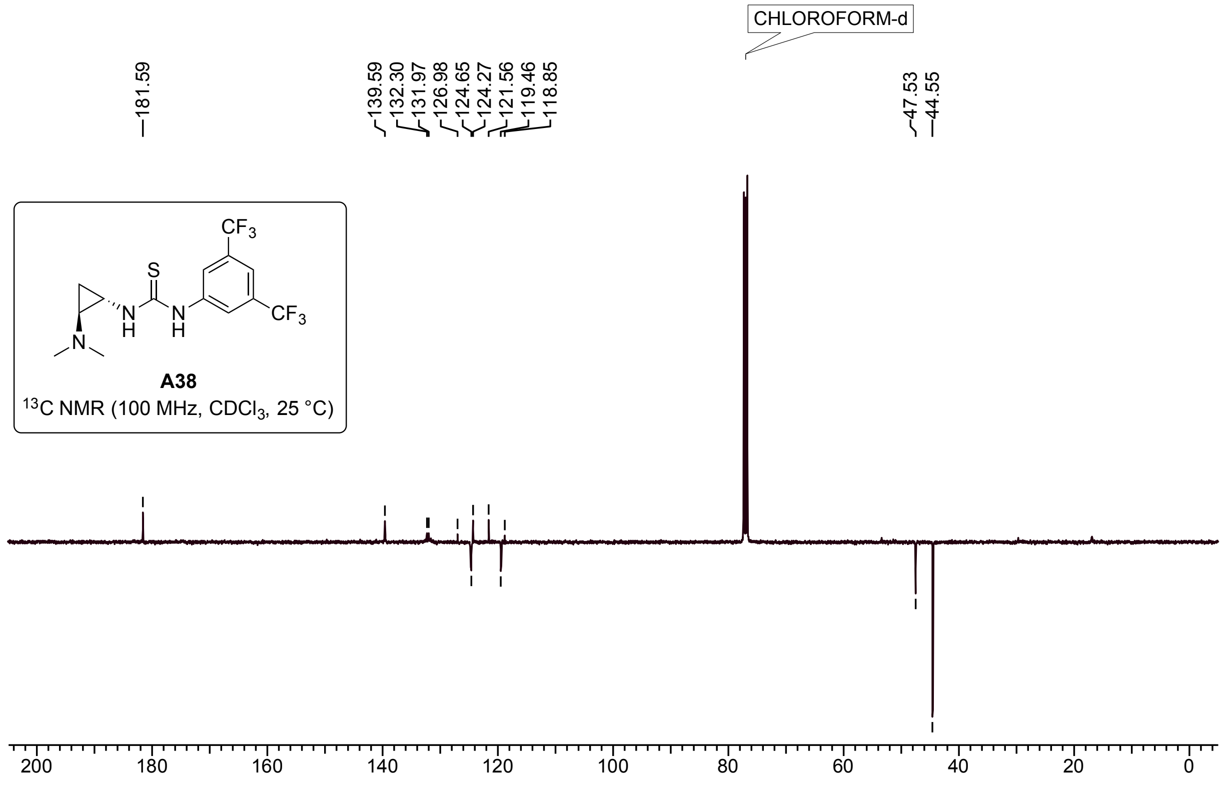
CHLOROFORM-d
181.59
139.59
132.30
131.97
126.98
124.65
124.27
121.56
119.46
118.85
47.53
44.55
CF3
S
N
H
N
H
CF3
N
A38
13C NMR (100 MHz, CDCl3, 25 °C)
200
180
160
140
120
100
80
60
40
20
0

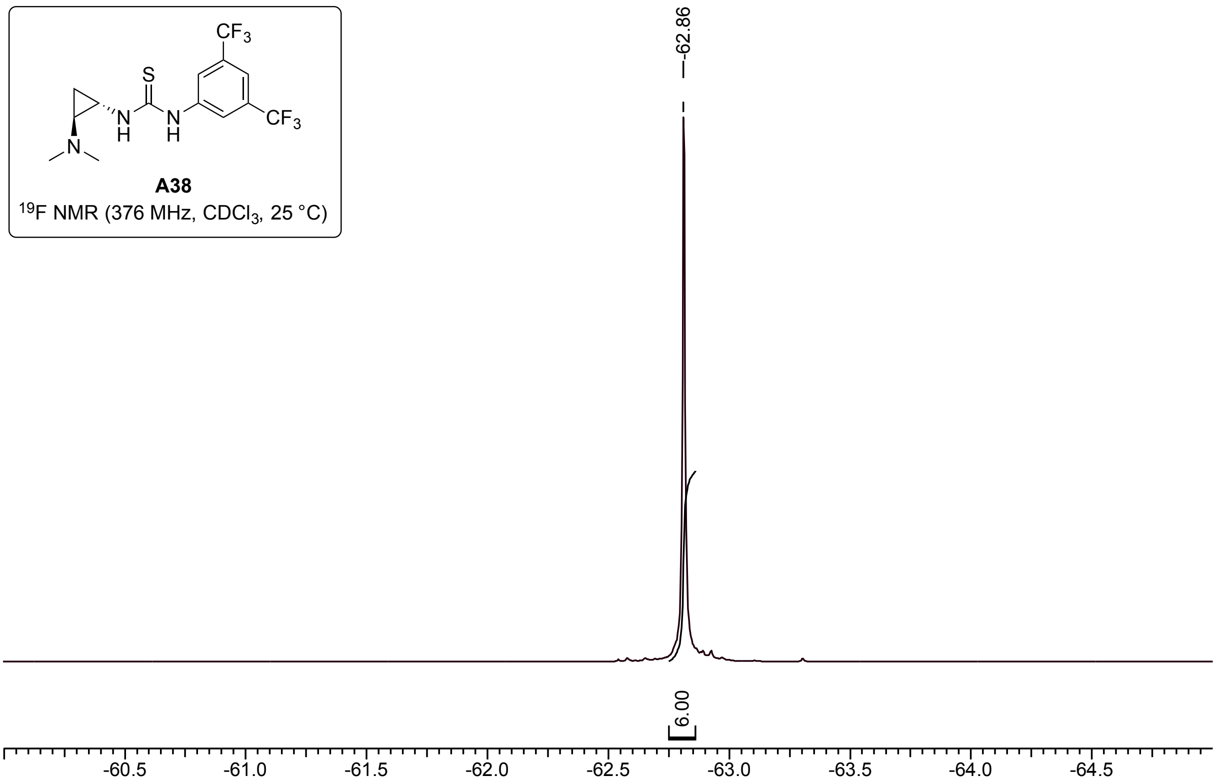
CF3
S
N
H
N
H
CF3
N
A38
19F NMR (376 MHz, CDCl3, 25 °C)
—62.86
6.00
-60.5
-61.0
-61.5
-62.0
-62.5
-63.0
-63.5
-64.0
-64.5

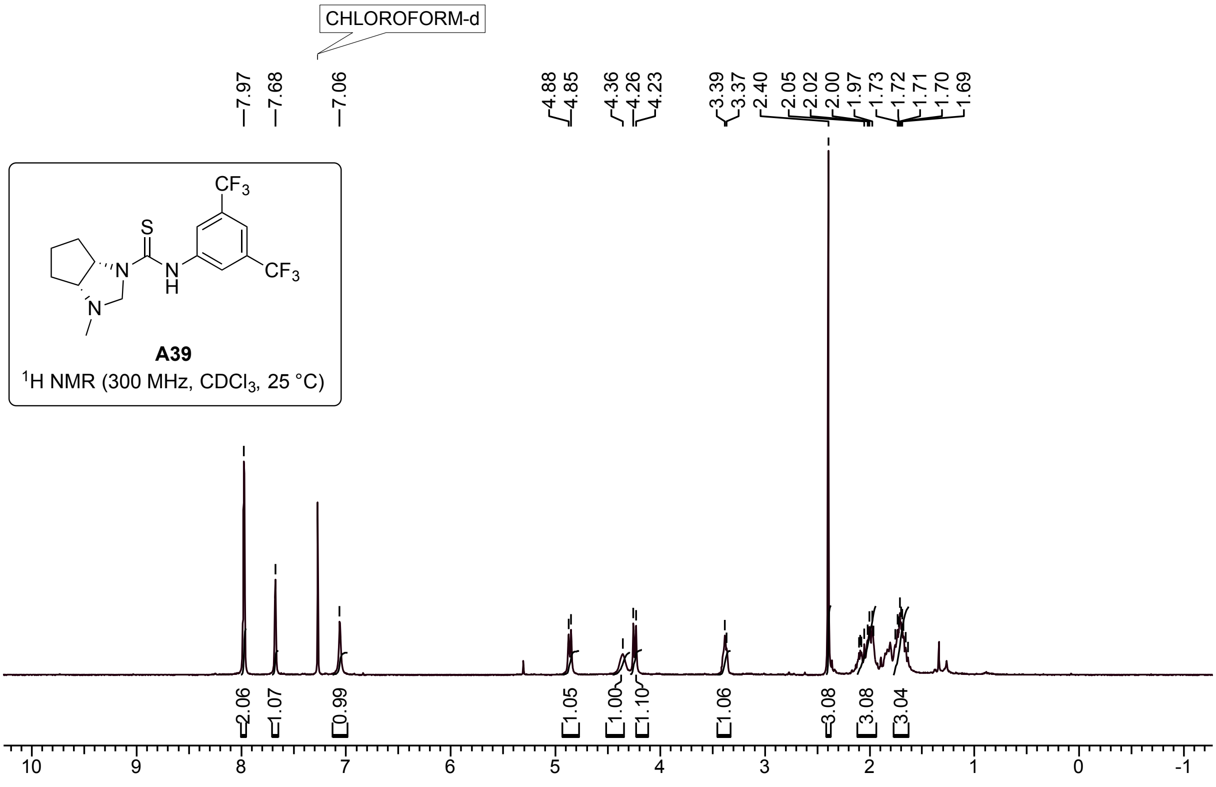
CHLOROFORM-d
7.97
7.68
7.06
4.88
4.85
4.36
4.26
4.23
3.39
3.37
2.40
2.05
2.02
2.00
1.97
1.73
1.72
1.71
1.70
1.69
CF3
S
N
N
H
CF3
N
A39
1H NMR (300 MHz, CDCl3, 25 °C)
2.06
1.07
0.99
1.05
1.00
1.10
1.06
3.08
3.08
3.04
10
9
8
7
6
5
4
3
2
1
0
-1

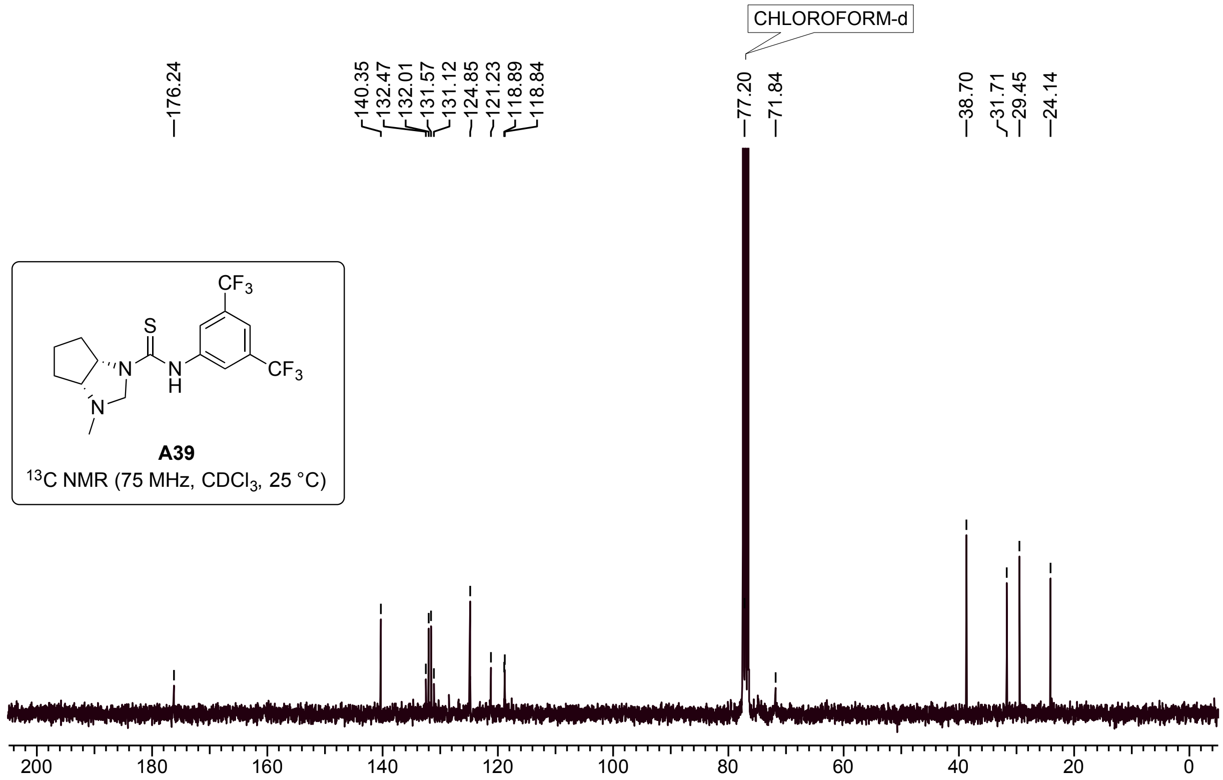
CHLOROFORM-d
176.24
140.35
132.47
132.01
131.57
131.12
124.85
121.23
118.89
118.84
77.20
71.84
38.70
31.71
29.45
24.14
CF3
S
N
H
N
CF3
A39
13C NMR (75 MHz, CDCl3, 25 °C)
200
180
160
140
120
100
80
60
40
20
0

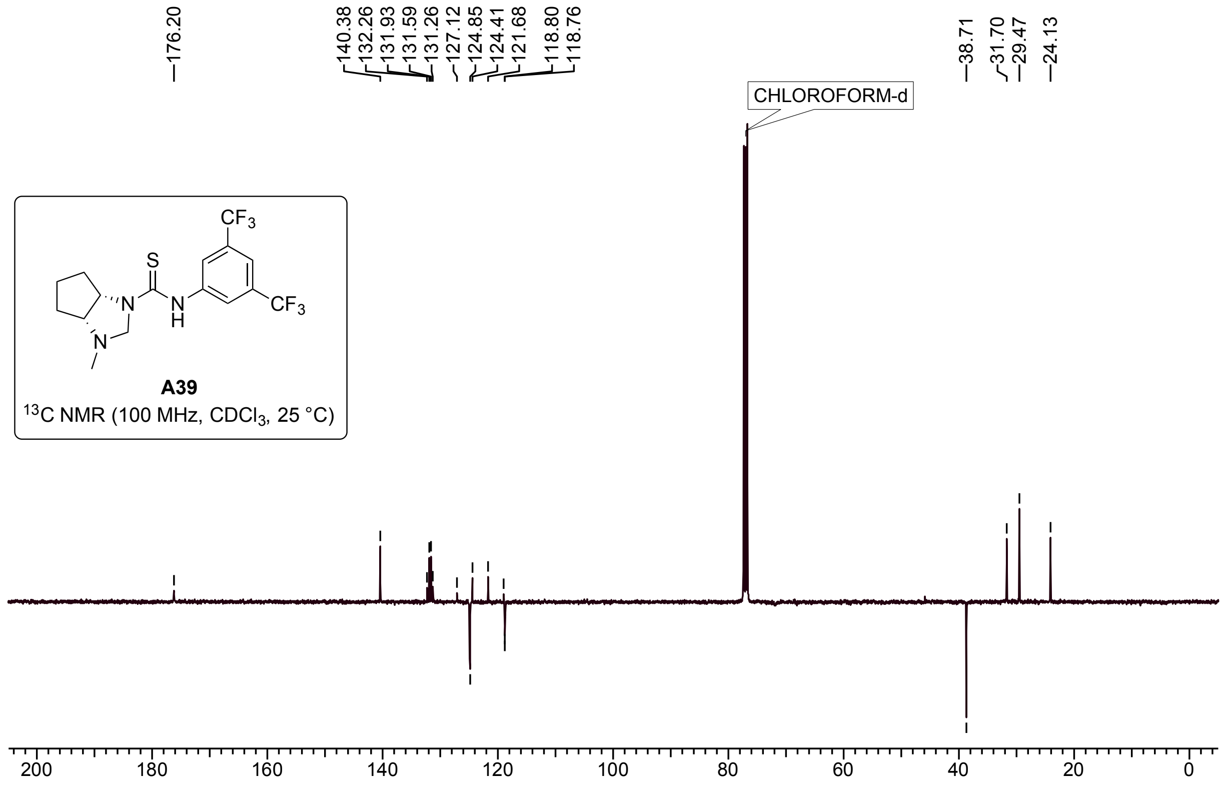
176.20
140.38
132.26
131.93
131.59
131.26
127.12
124.85
124.41
121.68
118.80
118.76
38.71
31.70
29.47
24.13
CHLOROFORM-d
CF3
S
N
H
N
CF3
A39
13C NMR (100 MHz, CDCl3, 25 °C)
200
180
160
140
120
100
80
60
40
20
0

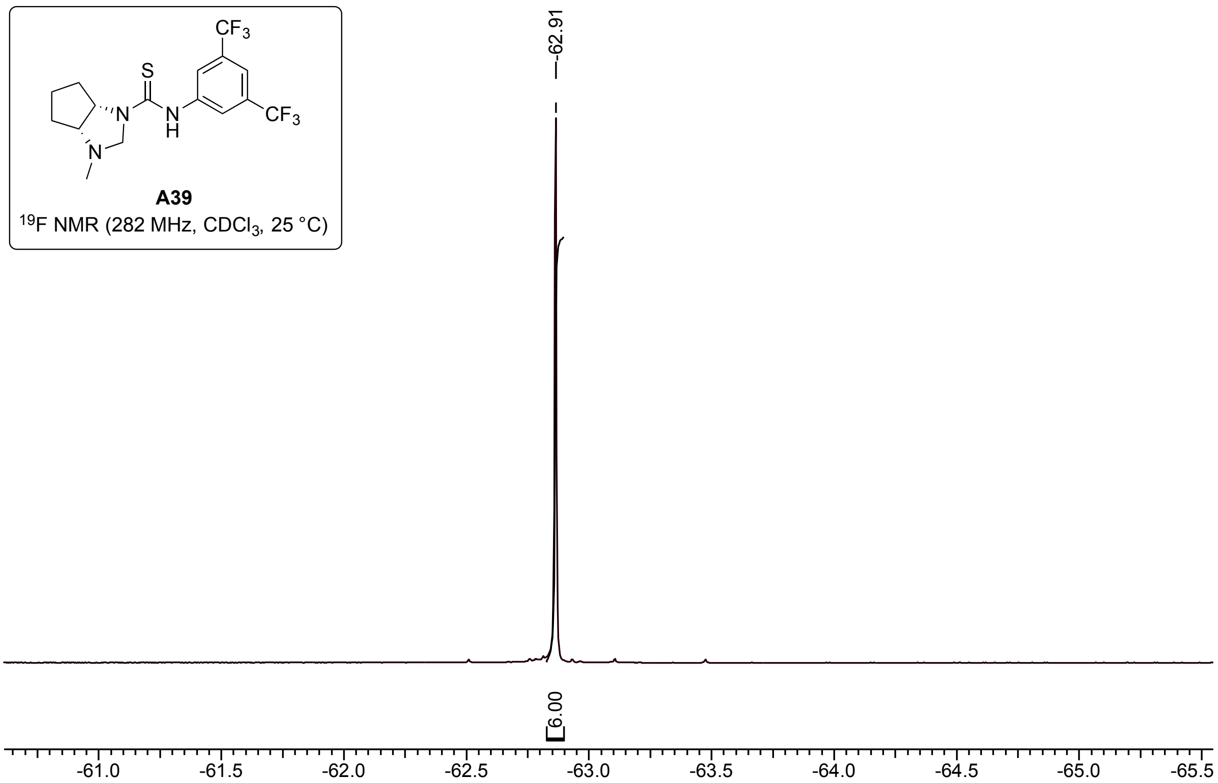

CF3
S
N
N
H
CF3
N
A39
19F NMR (282 MHz, CDCl3, 25 °C)
-62.91
6.00
-61.0
-61.5
-62.0
-62.5
-63.0
-63.5
-64.0
-64.5
-65.0
-65.5

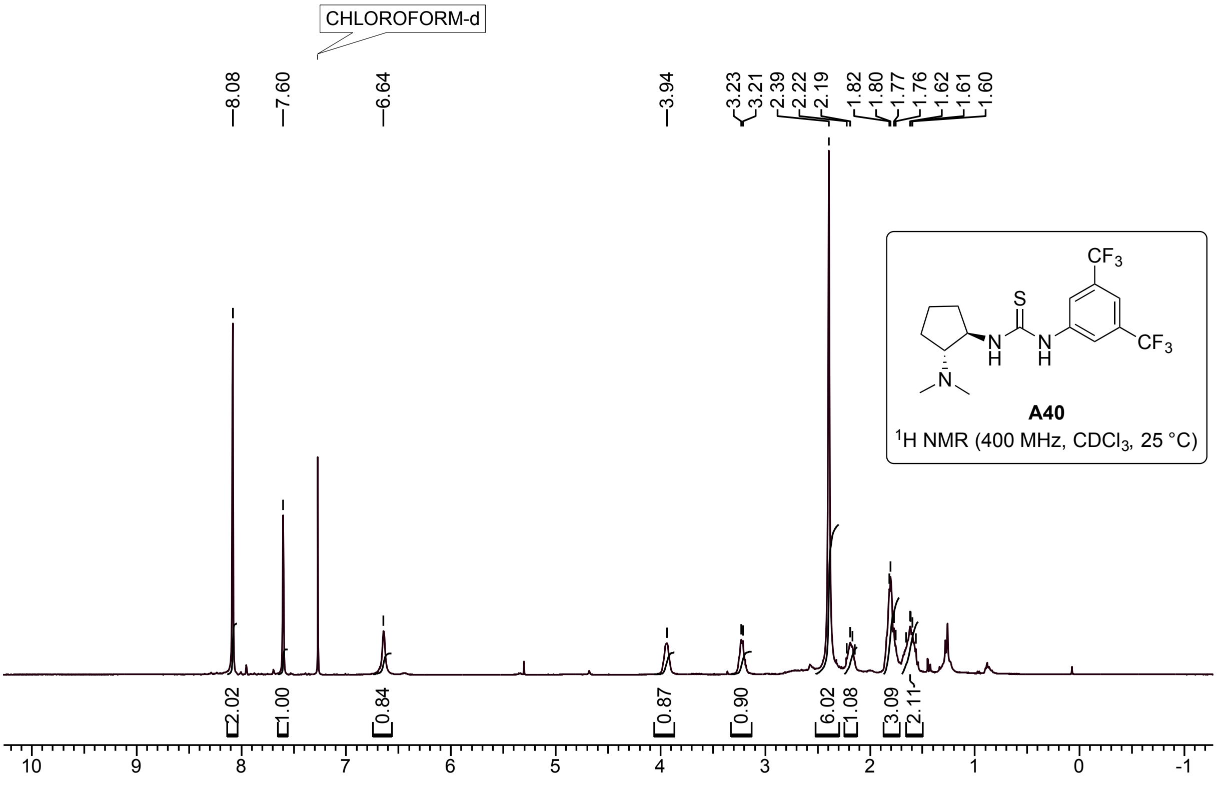

CHLOROFORM-d
8.08
7.60
6.64
3.94
3.23
3.21
2.39
2.22
2.19
1.82
1.80
1.77
1.76
1.62
1.61
1.60
CF3
S
N
H
N
H
CF3
N
A40
1H NMR (400 MHz, CDCl3, 25 °C)
2.02
1.00
0.84
0.87
0.90
6.02
1.08
3.09
2.11
10
9
8
7
6
5
4
3
2
1
0
-1

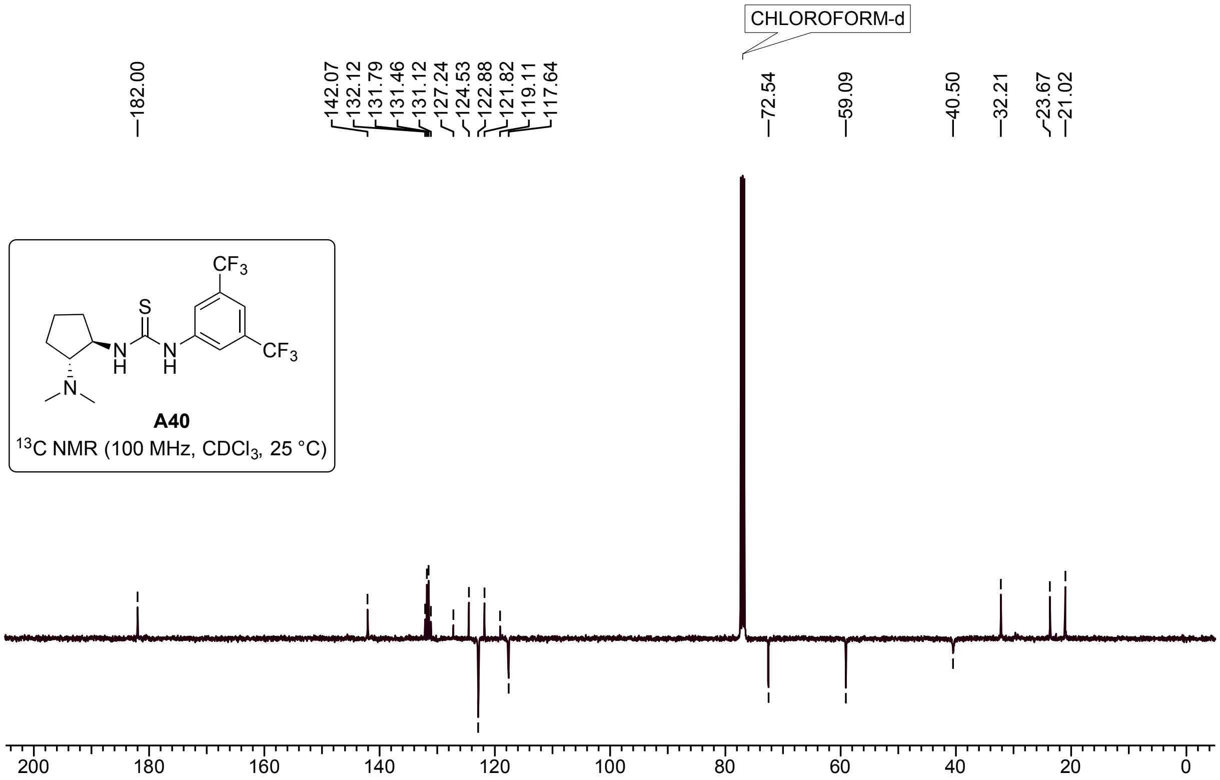
CHLOROFORM-d
182.00
142.07
132.12
131.79
131.46
131.12
127.24
124.53
122.88
121.82
119.11
117.64
72.54
59.09
40.50
32.21
23.67
21.02
CF3
S
N
H
N
H
CF3
N
A40
13C NMR (100 MHz, CDCl3, 25 °C)
200
180
160
140
120
100
80
60
40
20
0

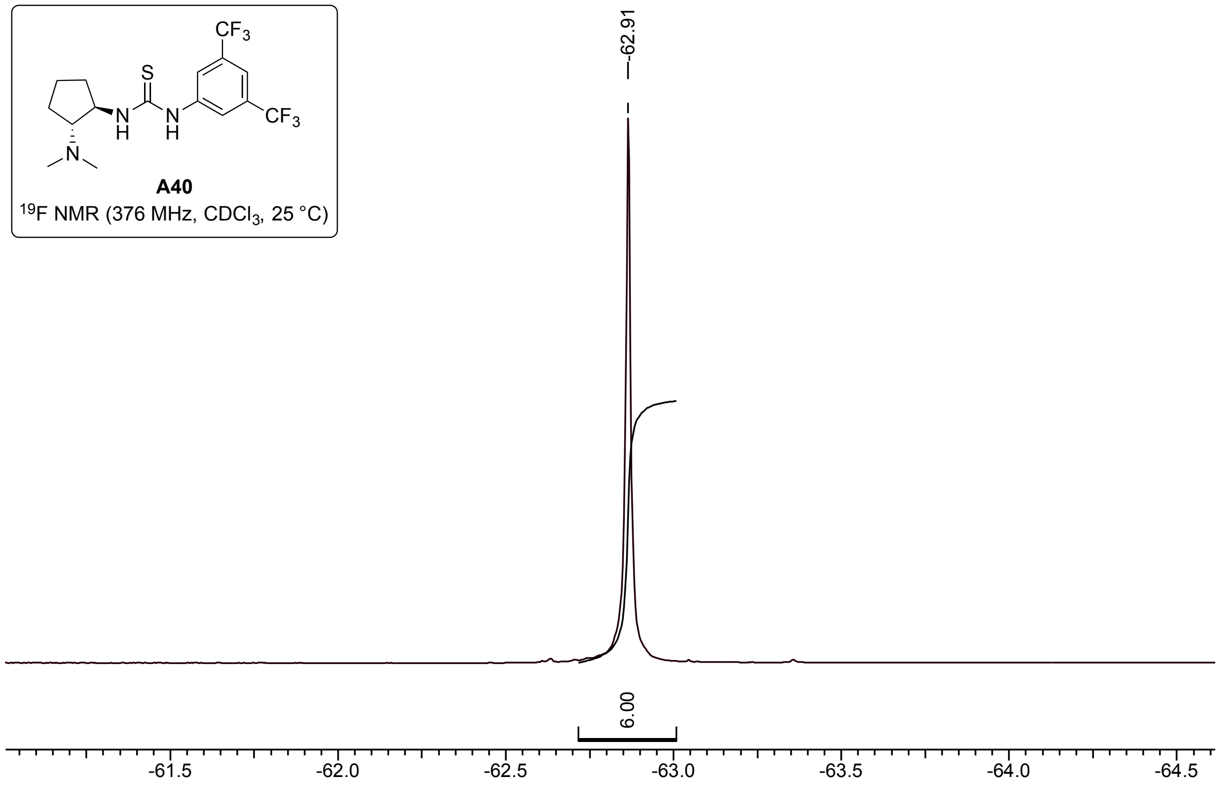
-62.91
CF3
S
N
H
N
H
CF3
N
A40
19F NMR (376 MHz, CDCl3, 25 °C)
6.00
-61.5
-62.0
-62.5
-63.0
-63.5
-64.0
-64.5

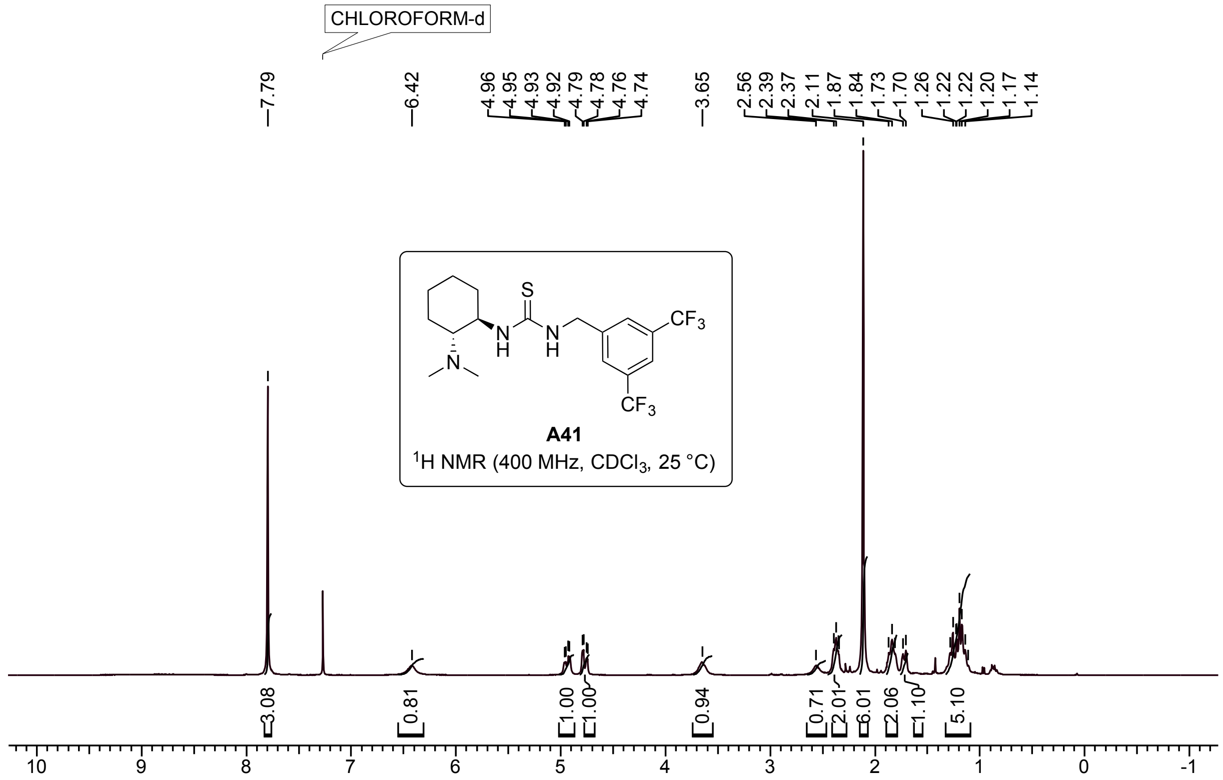
CHLOROFORM-d
7.79
6.42
4.96
4.95
4.93
4.92
4.79
4.78
4.76
4.74
3.65
2.56
2.39
2.37
2.11
1.87
1.84
1.73
1.70
1.26
1.22
1.22
1.20
1.17
1.14
S
N
H
N
H
N
CF3
CF3
A41
1H NMR (400 MHz, CDCl3, 25 °C)
3.08
0.81
1.00
1.00
0.94
0.71
2.01
6.01
2.06
1.10
5.10
10
9
8
7
6
5
4
3
2
1
0
-1

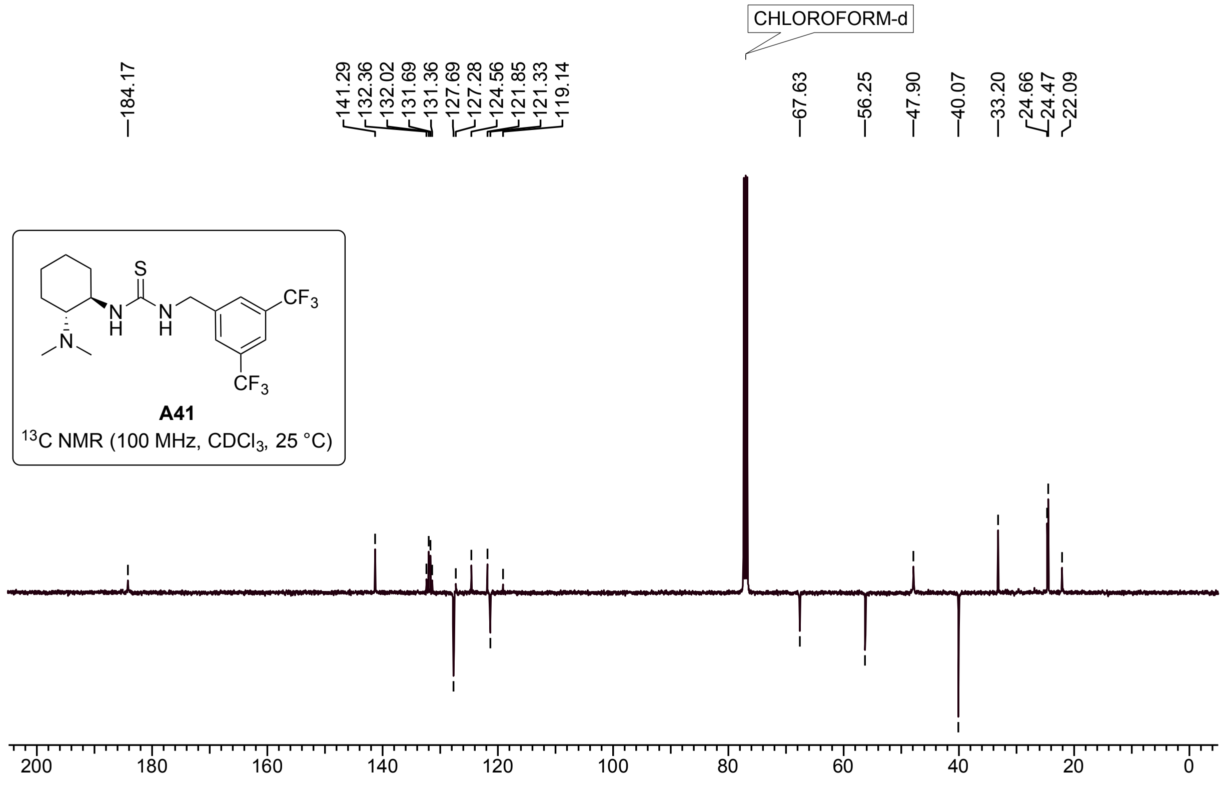
CHLOROFORM-d
184.17
141.29
132.36
132.02
131.69
131.36
127.69
127.28
124.56
121.85
121.33
119.14
67.63
56.25
47.90
40.07
33.20
24.66
24.47
22.09
S
N
H
N
H
N
CF3
CF3
A41
13C NMR (100 MHz, CDCl3, 25 °C)
200
180
160
140
120
100
80
60
40
20
0

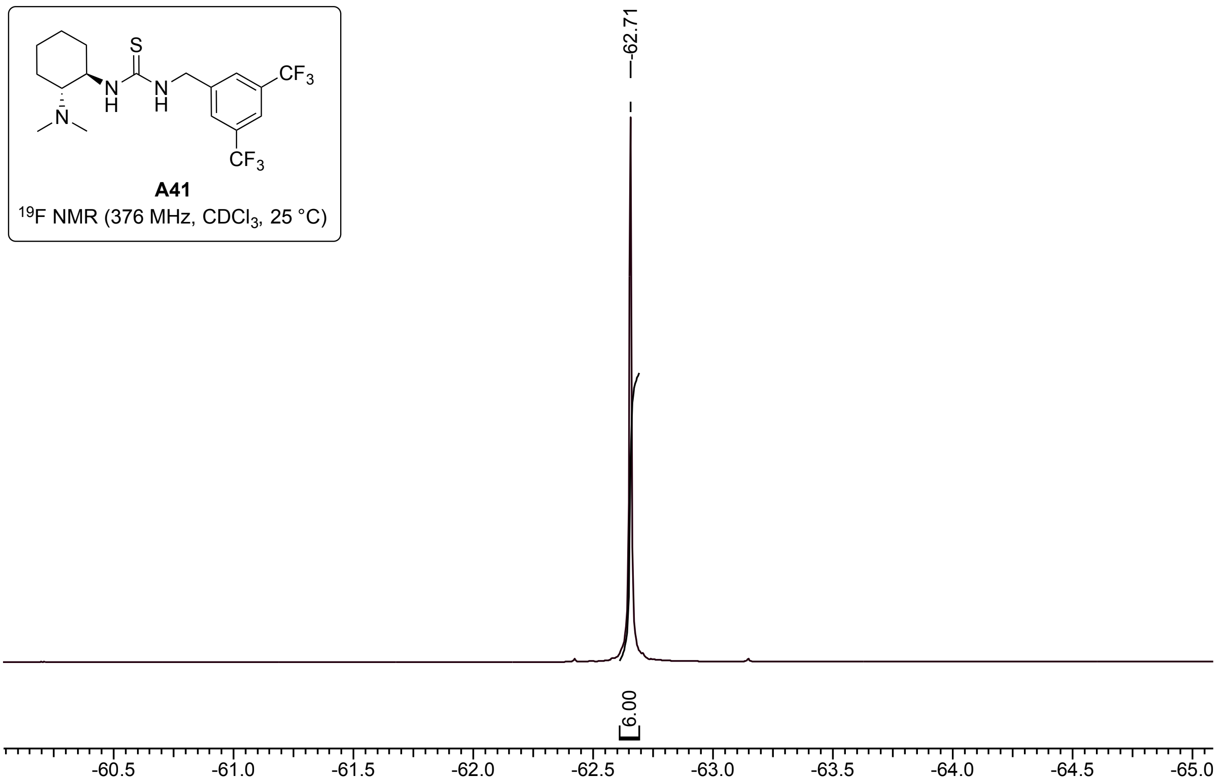
A41
^{19}F NMR (376 MHz, CDCl$_3$, 25 °C)
-62.71
6.00

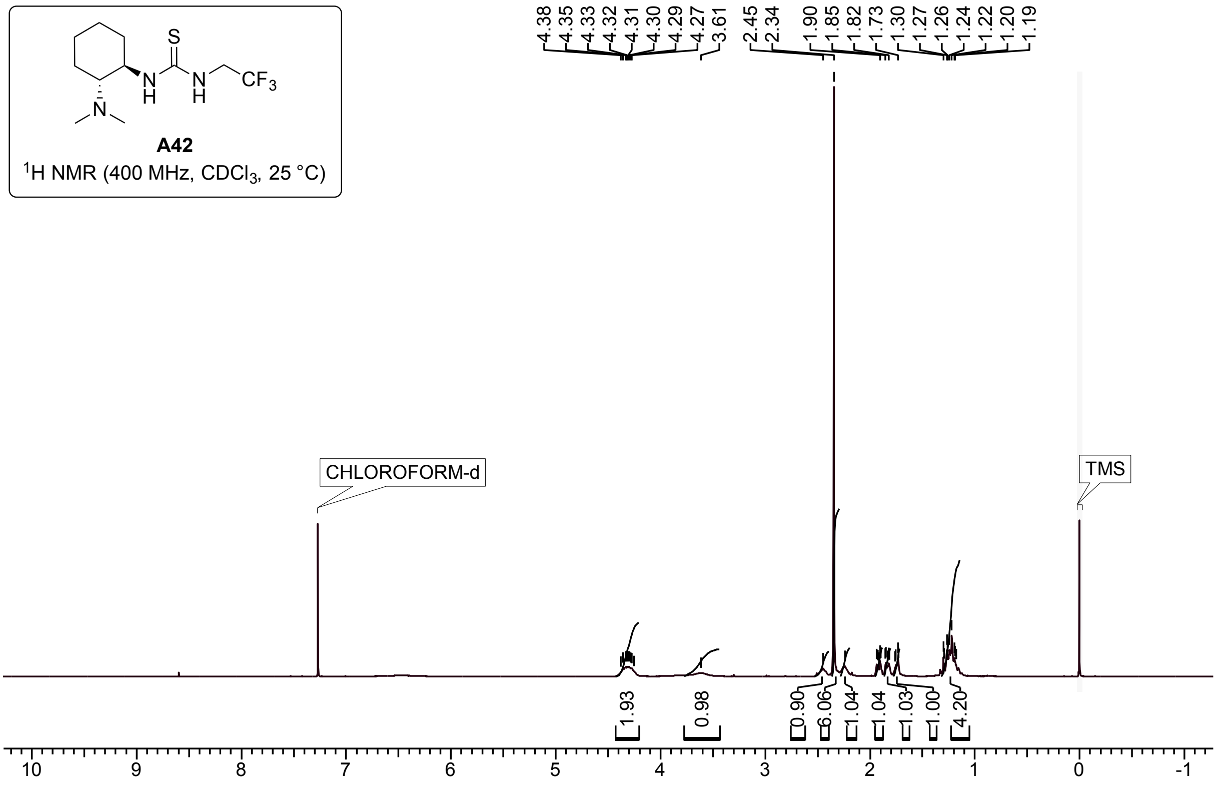
A42
^{1}H NMR (400 MHz, CDCl$_3$, 25 °C)
CHLOROFORM-d
TMS

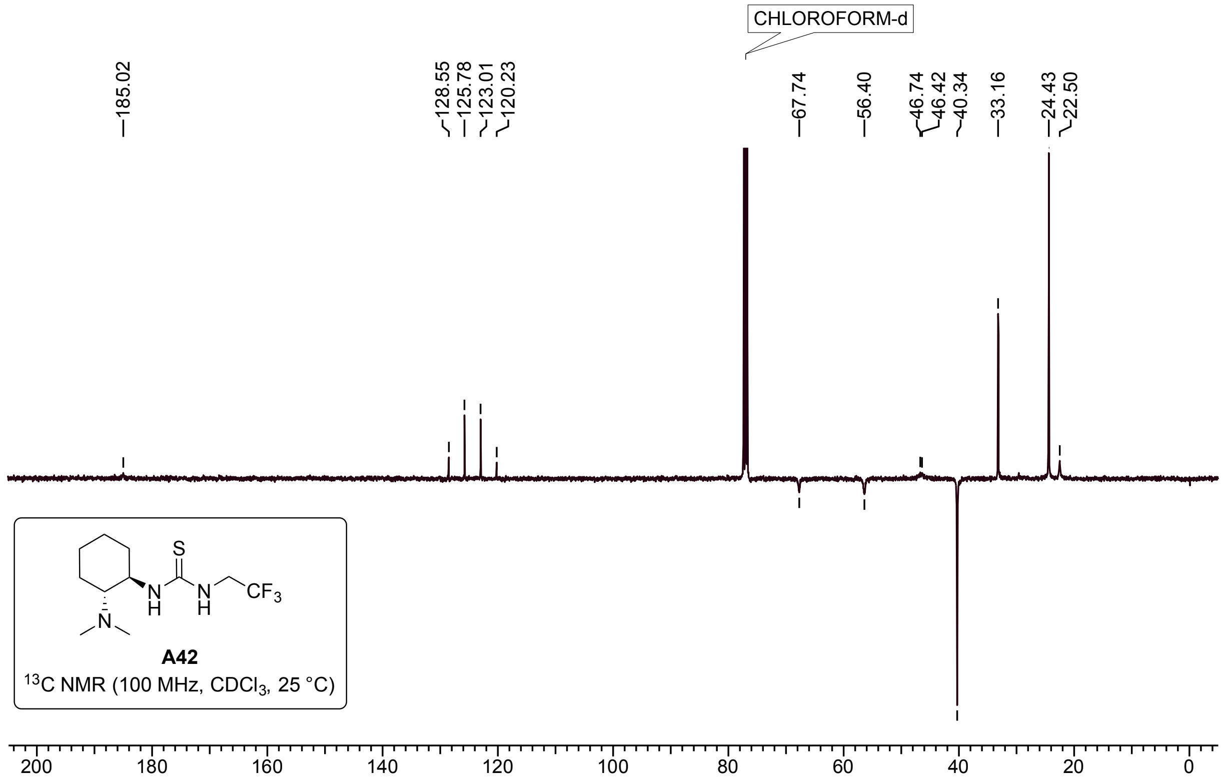
CHLOROFORM-d
185.02
128.55
125.78
123.01
120.23
67.74
56.40
46.74
46.42
40.34
33.16
24.43
22.50
S
N
H
N
H
CF3
N
A42
13C NMR (100 MHz, CDCl3, 25 °C)
200
180
160
140
120
100
80
60
40
20
0

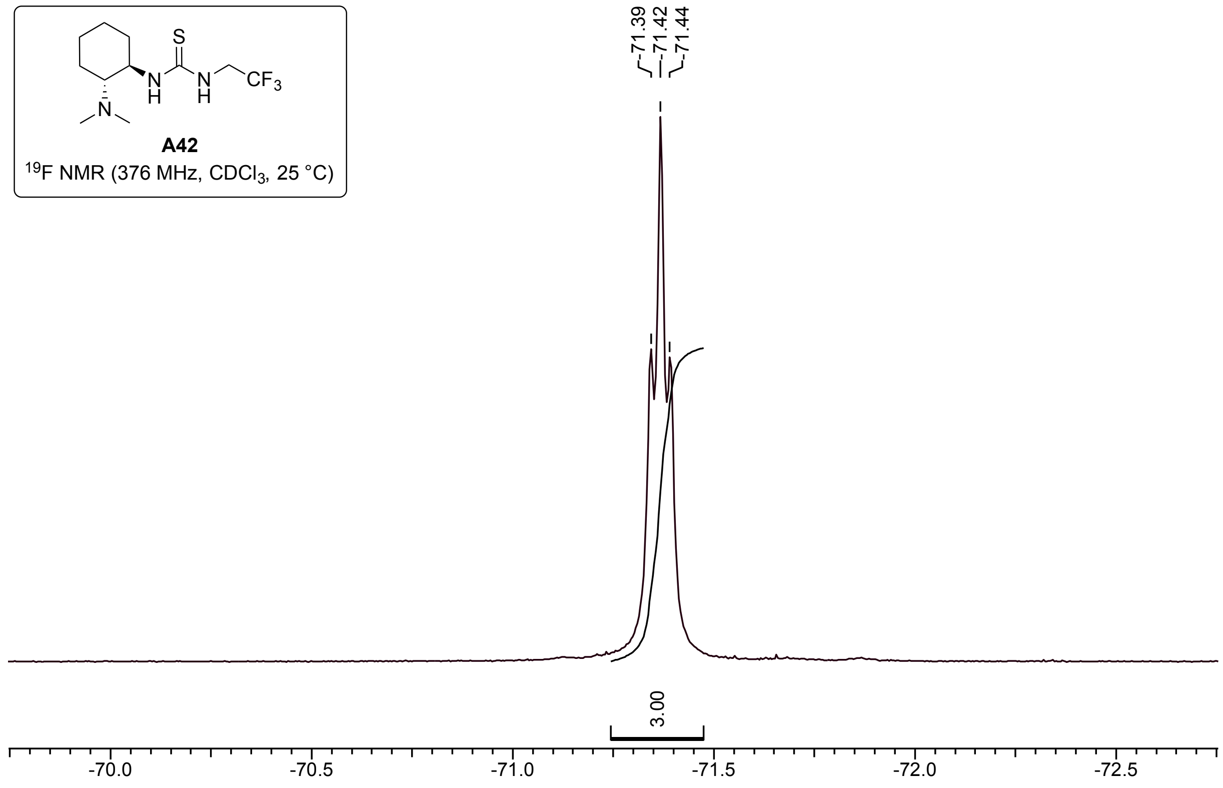
S
N
H
N
H
CF3
N
A42
19F NMR (376 MHz, CDCl3, 25 °C)
-71.39
-71.42
-71.44
3.00
-70.0
-70.5
-71.0
-71.5
-72.0
-72.5

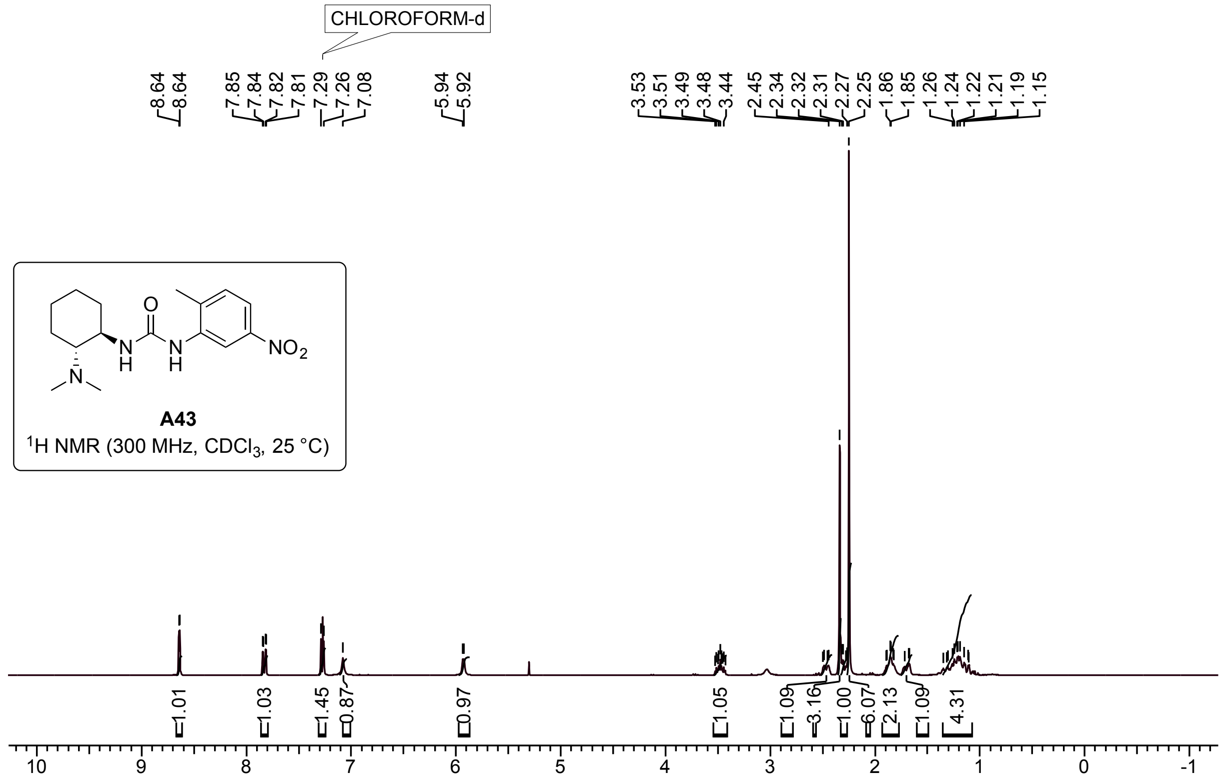
CHLOROFORM-d
8.64 8.64 7.85 7.84 7.82 7.81 7.29 7.26 7.08 5.94 5.92 3.53 3.51 3.49 3.48 3.44 2.45 2.34 2.32 2.31 2.27 2.25 1.86 1.85 1.26 1.24 1.22 1.21 1.19 1.15
NO2
A43
1H NMR (300 MHz, CDCl3, 25 °C)
1.01 1.03 1.45 0.87 0.97 1.05 1.09 3.16 1.00 6.07 2.13 1.09 4.31
10 9 8 7 6 5 4 3 2 1 0 -1

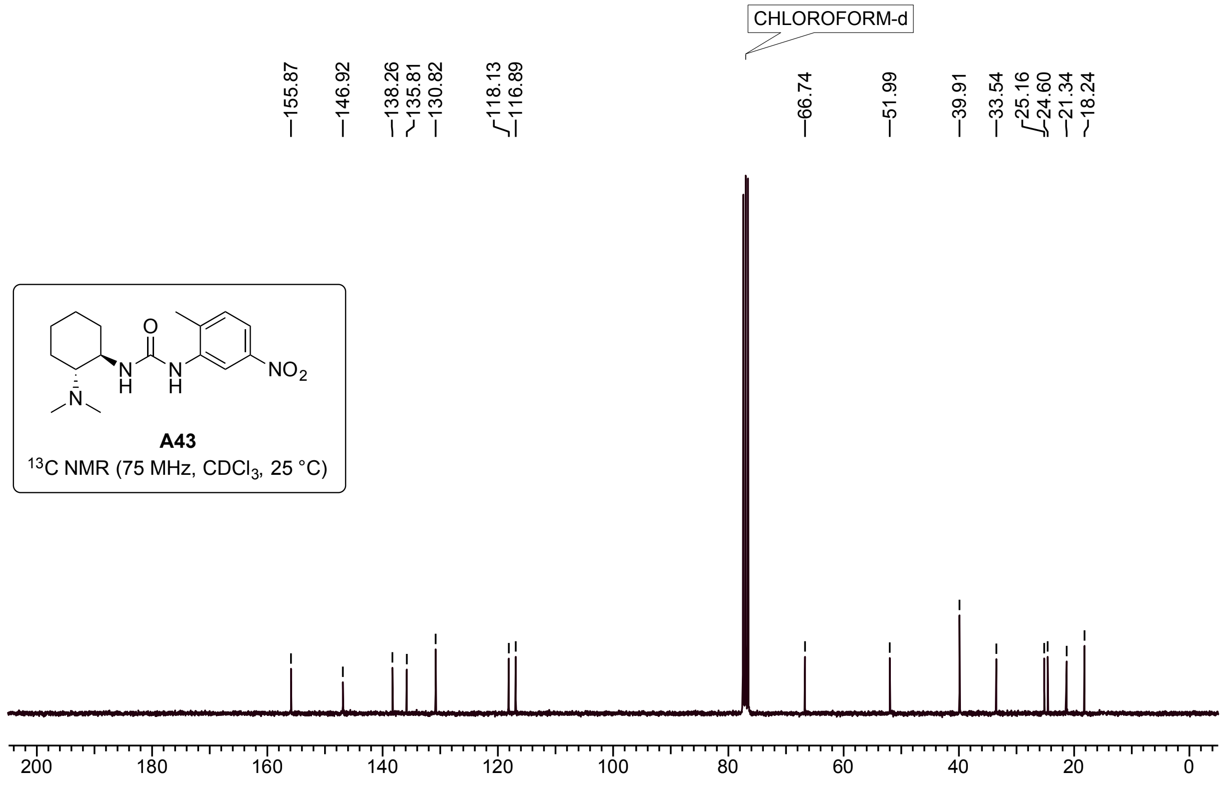
CHLOROFORM-d
155.87 146.92 138.26 135.81 130.82 118.13 116.89 66.74 51.99 39.91 33.54 25.16 24.60 21.34 18.24
NO2
A43
13C NMR (75 MHz, CDCl3, 25 °C)
200 180 160 140 120 100 80 60 40 20 0

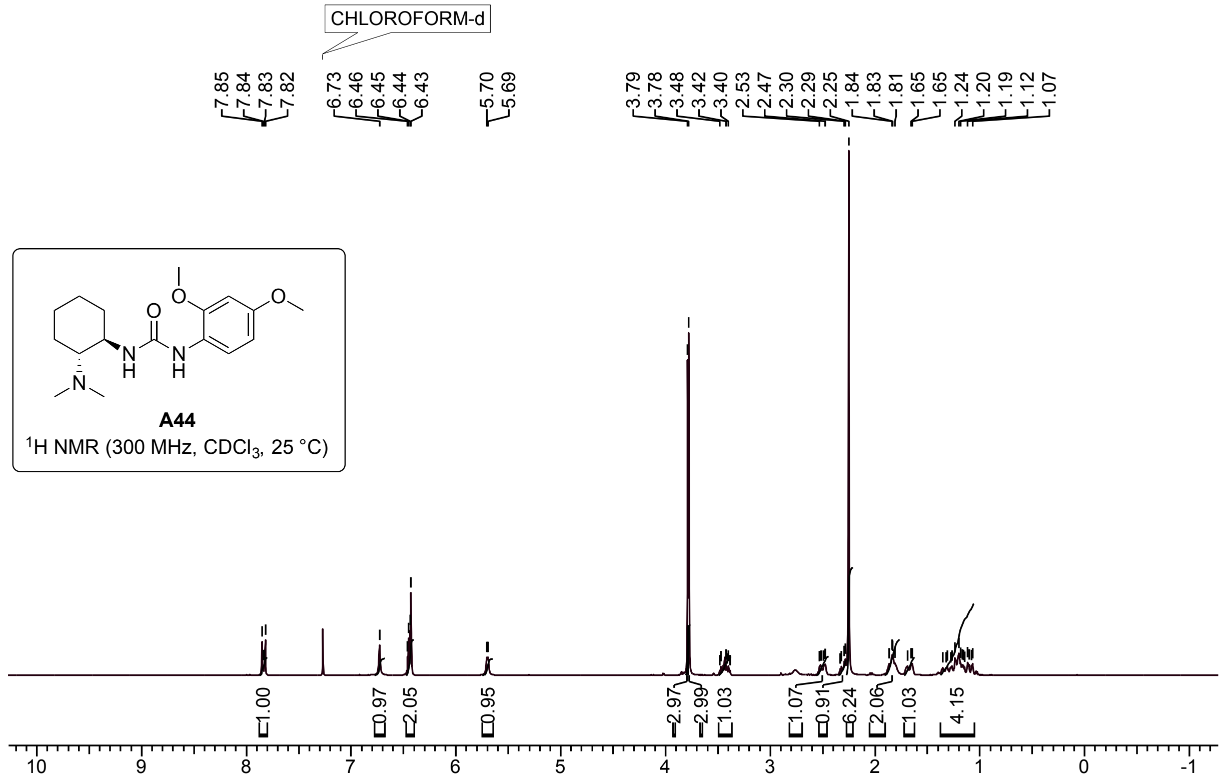
CHLOROFORM-d
7.85
7.84
7.83
7.82
6.73
6.46
6.45
6.44
6.43
5.70
5.69
3.79
3.78
3.48
3.42
3.40
2.53
2.47
2.30
2.29
2.25
1.84
1.83
1.81
1.65
1.65
1.24
1.20
1.19
1.12
1.07
A44
^{1}H NMR (300 MHz, $CDCl_3$, 25 °C)
1.00
0.97
2.05
0.95
2.97
2.99
1.03
1.07
0.91
6.24
2.06
1.03
4.15
10
9
8
7
6
5
4
3
2
1
0
-1

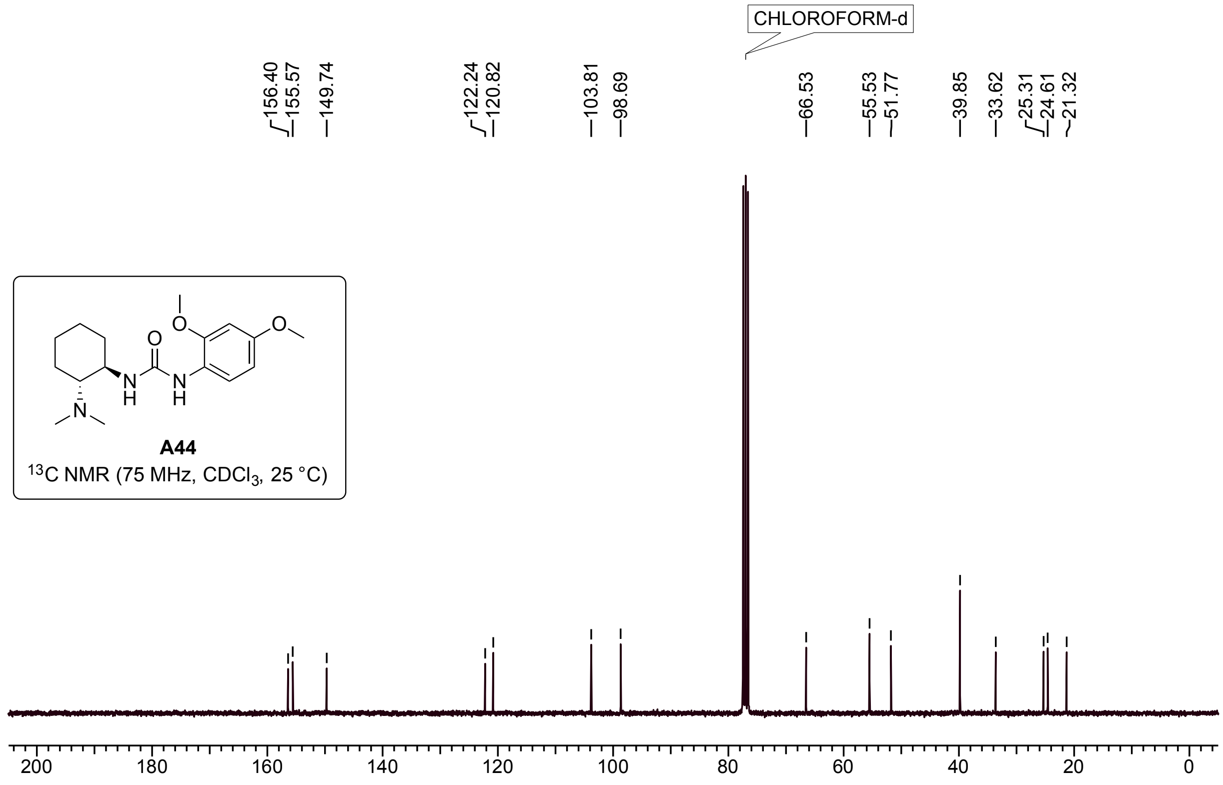
CHLOROFORM-d
156.40
155.57
149.74
122.24
120.82
103.81
98.69
66.53
55.53
51.77
39.85
33.62
25.31
24.61
21.32
A44
^{13}C NMR (75 MHz, $CDCl_3$, 25 °C)
200
180
160
140
120
100
80
60
40
20
0

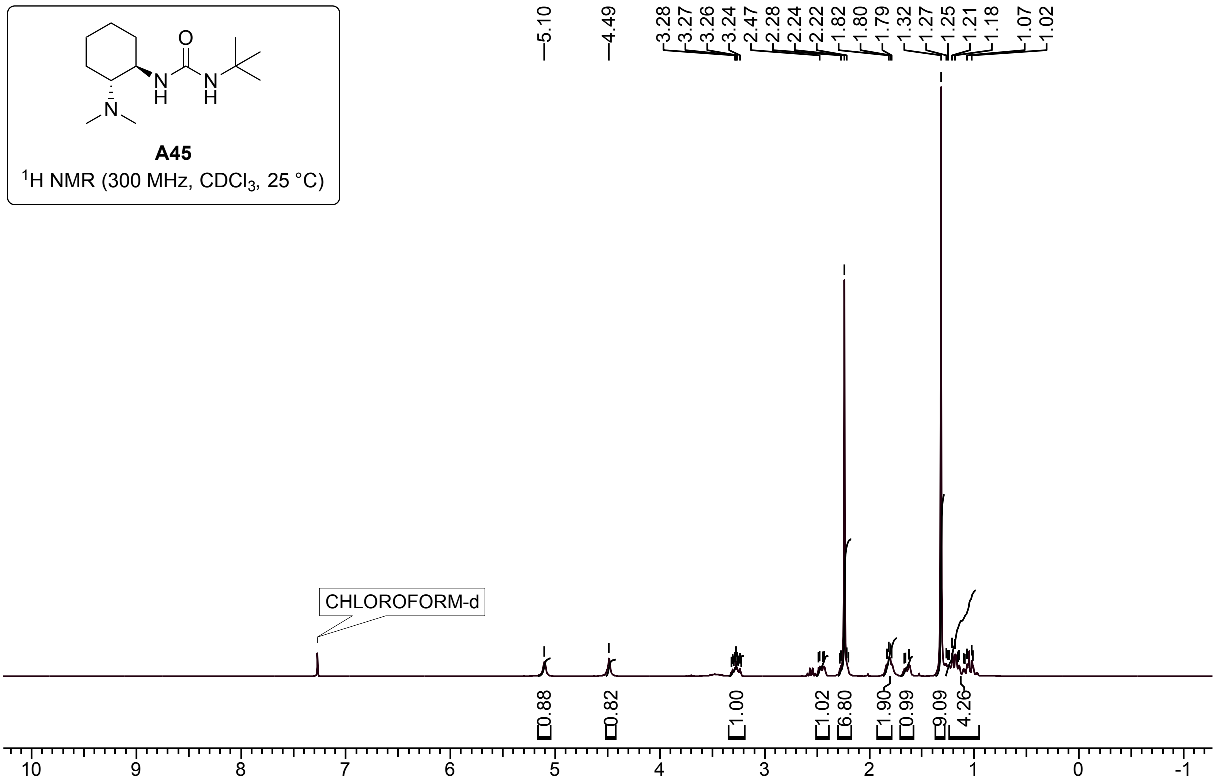
A45
1H NMR (300 MHz, CDCl3, 25 °C)
5.10
4.49
3.28
3.27
3.26
3.24
2.47
2.28
2.24
2.22
1.82
1.80
1.79
1.32
1.27
1.25
1.21
1.18
1.07
1.02
CHLOROFORM-d
0.88
0.82
1.00
1.02
6.80
1.90
0.99
9.09
4.26
10
9
8
7
6
5
4
3
2
1
0
-1

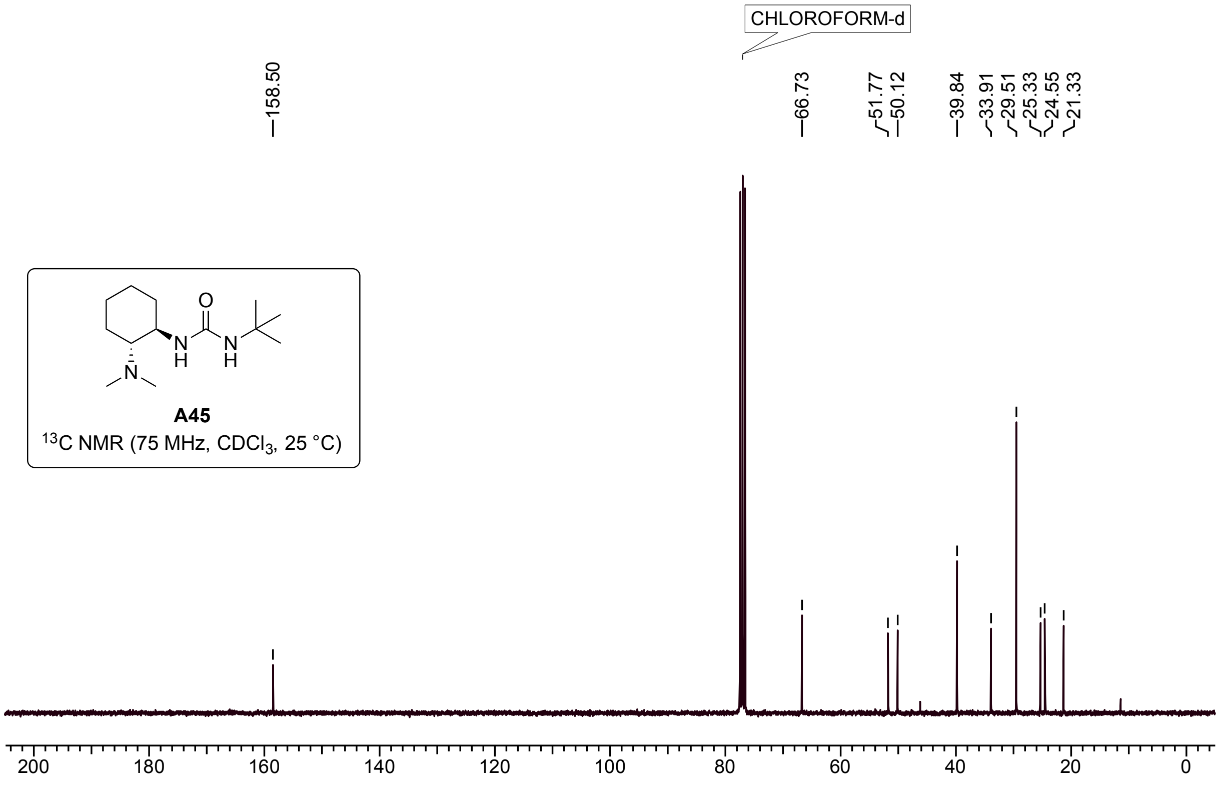
CHLOROFORM-d
158.50
66.73
51.77
50.12
39.84
33.91
29.51
25.33
24.55
21.33
A45
13C NMR (75 MHz, CDCl3, 25 °C)
200
180
160
140
120
100
80
60
40
20
0

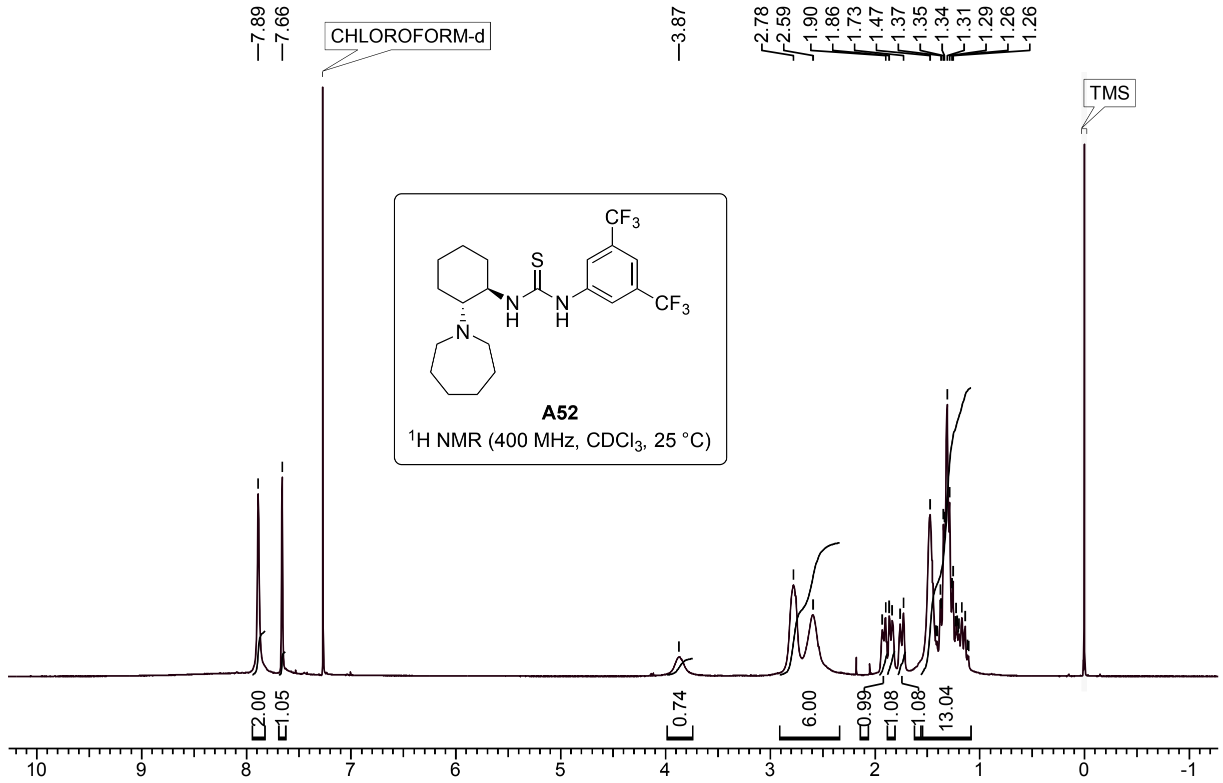
7.89
7.66
CHLOROFORM-d
3.87
2.78
2.59
1.90
1.86
1.73
1.47
1.37
1.35
1.34
1.31
1.29
1.26
1.26
TMS
CF3
S
N
H
CF3
A52
^{1}H NMR (400 MHz, $CDCl_3$, 25 °C)
2.00
1.05
0.74
6.00
0.99
1.08
1.08
13.04
10
9
8
7
6
5
4
3
2
1
0
-1

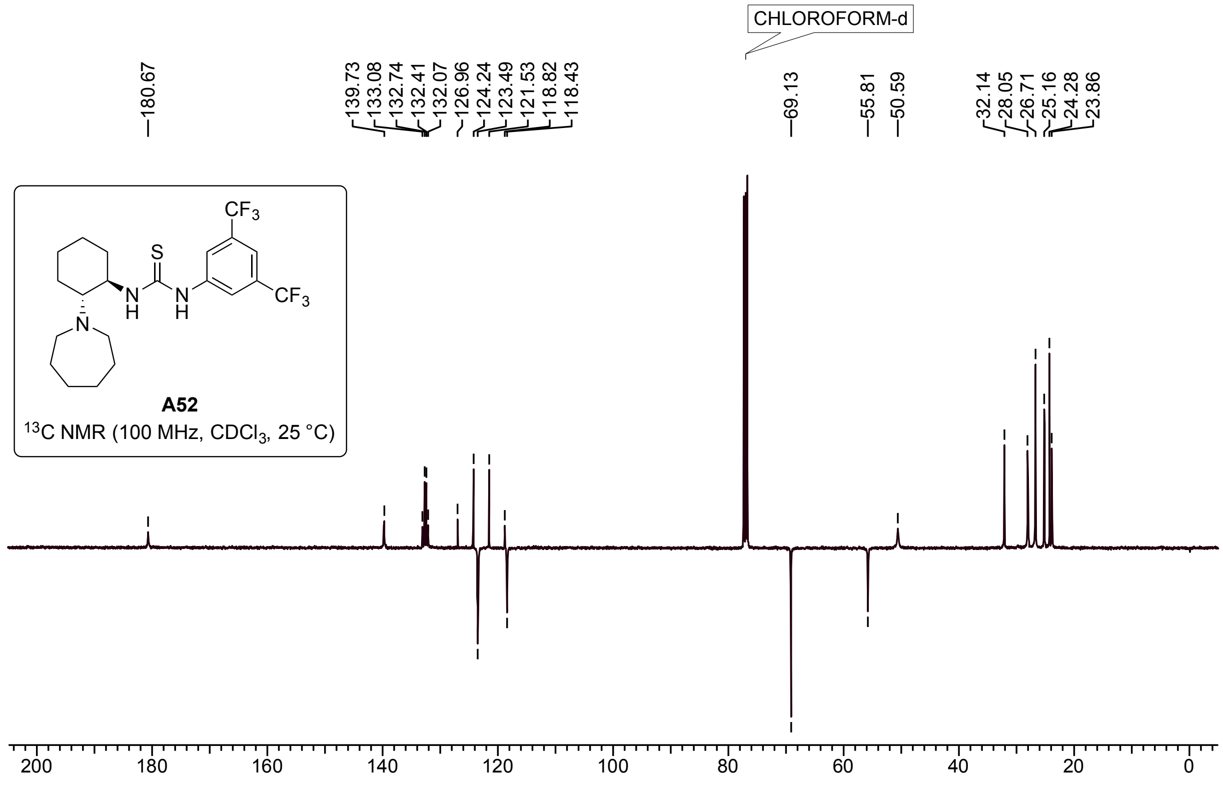
CHLOROFORM-d
180.67
139.73
133.08
132.74
132.41
132.07
126.96
124.24
123.49
121.53
118.82
118.43
69.13
55.81
50.59
32.14
28.05
26.71
25.16
24.28
23.86
CF3
S
N
H
CF3
A52
^{13}C NMR (100 MHz, $CDCl_3$, 25 °C)
200
180
160
140
120
100
80
60
40
20
0

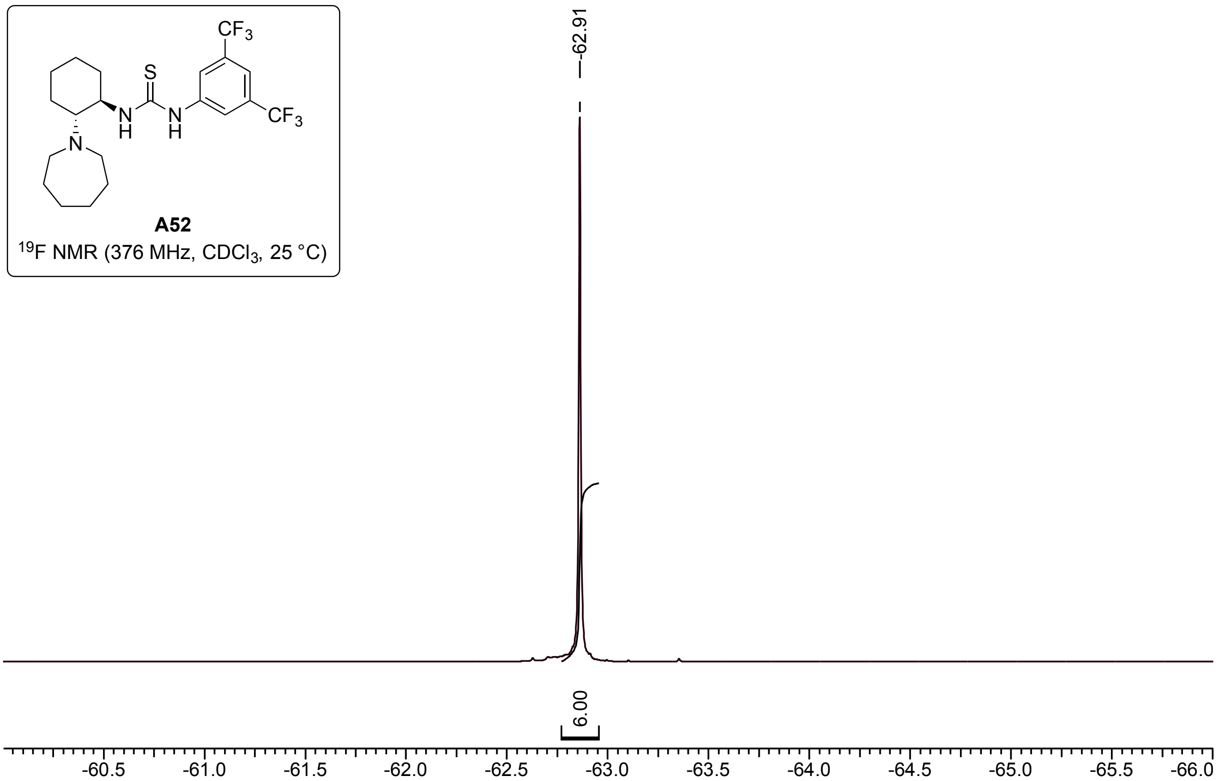
-62.91
CF3
S
N
H
N
H
CF3
N
A52
19F NMR (376 MHz, CDCl3, 25 °C)
6.00
-60.5
-61.0
-61.5
-62.0
-62.5
-63.0
-63.5
-64.0
-64.5
-65.0
-65.5
-66.0

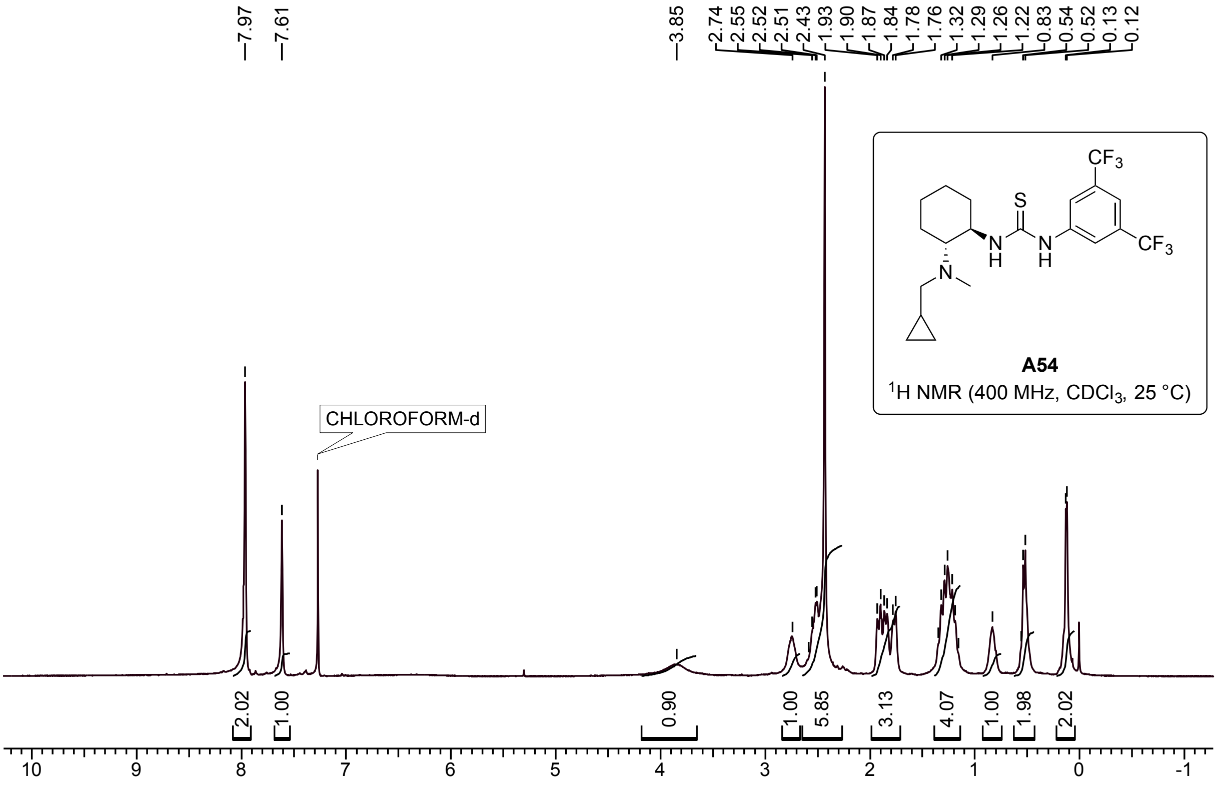
7.97
7.61
3.85
2.74
2.55
2.52
2.51
2.43
1.93
1.90
1.87
1.84
1.78
1.76
1.32
1.29
1.26
1.22
0.83
0.54
0.52
0.13
0.12
CF3
S
N
H
N
H
CF3
N
A54
1H NMR (400 MHz, CDCl3, 25 °C)
CHLOROFORM-d
2.02
1.00
0.90
1.00
5.85
3.13
4.07
1.00
1.98
2.02
10
9
8
7
6
5
4
3
2
1
0
-1

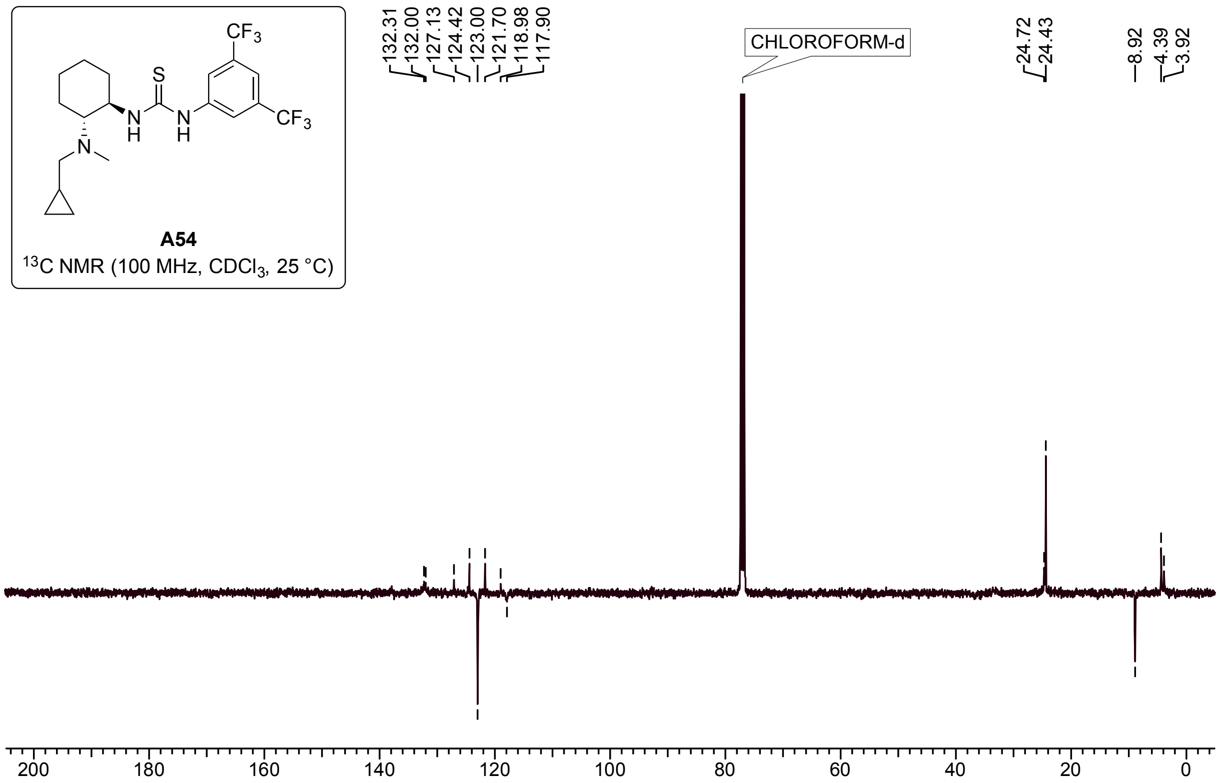
CF3
S
N
H
N
H
CF3
N
A54
13C NMR (100 MHz, CDCl3, 25 °C)
132.31
132.00
127.13
124.42
123.00
121.70
118.98
117.90
CHLOROFORM-d
24.72
24.43
8.92
4.39
3.92
200
180
160
140
120
100
80
60
40
20
0

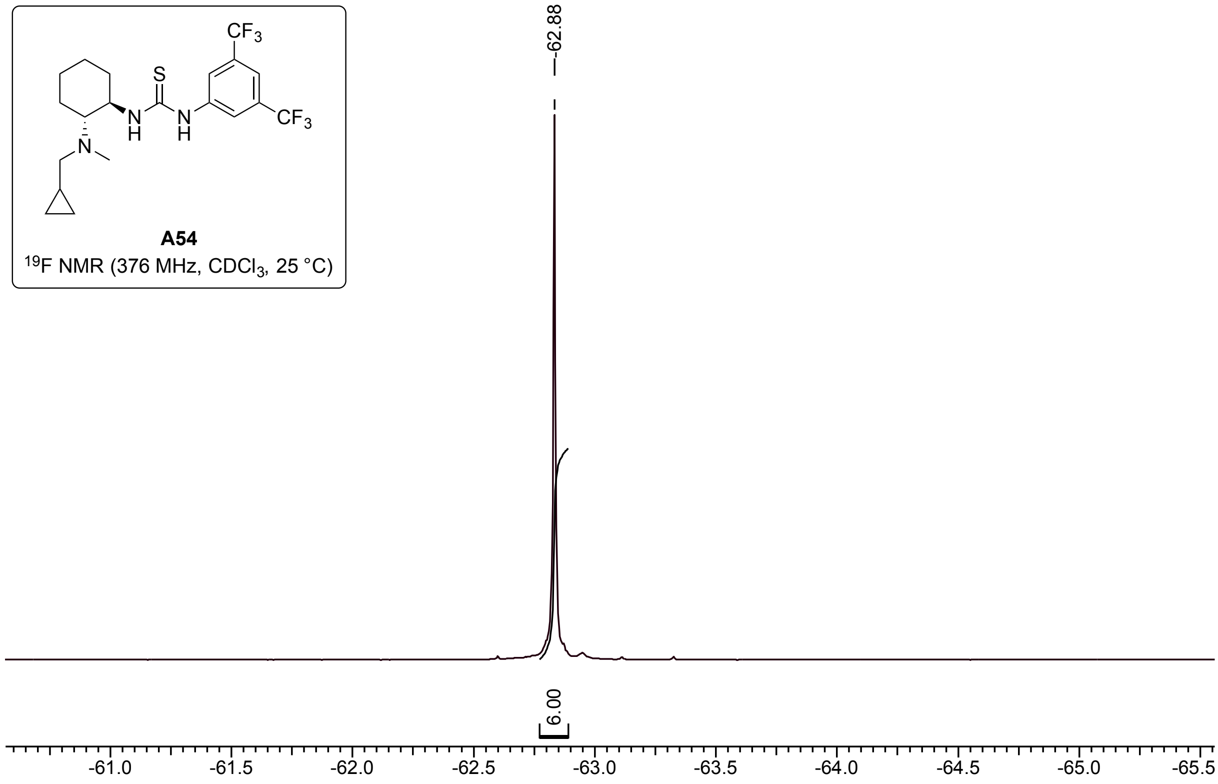
CF3
S
N
H
N
H
CF3
N
A54
19F NMR (376 MHz, CDCl3, 25 °C)
-62.88
6.00
-61.0
-61.5
-62.0
-62.5
-63.0
-63.5
-64.0
-64.5
-65.0
-65.5

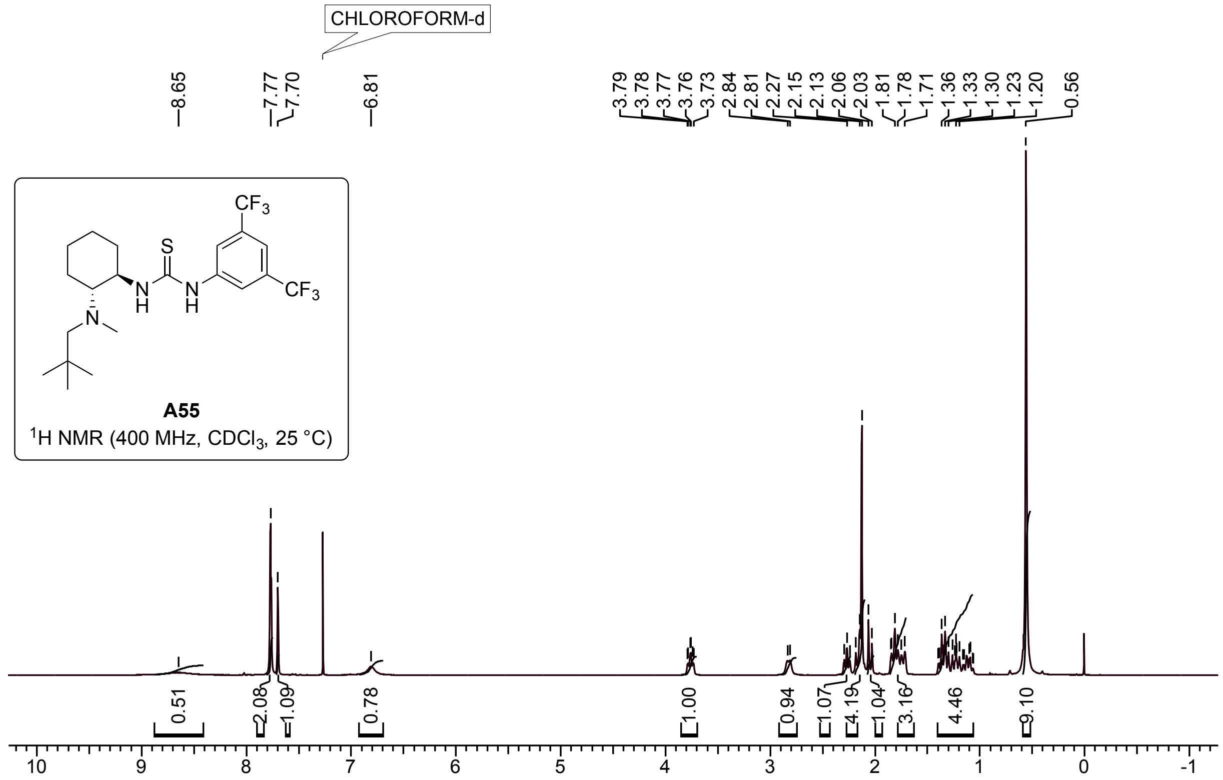
CHLOROFORM-d
8.65
7.77
7.70
6.81
3.79
3.78
3.77
3.76
3.73
2.84
2.81
2.27
2.15
2.13
2.06
2.03
1.81
1.78
1.71
1.36
1.33
1.30
1.23
1.20
0.56
A55
^{1}H NMR (400 MHz, $CDCl_3$, 25 °C)
0.51
2.08
1.09
0.78
1.00
0.94
1.07
4.19
1.04
3.16
4.46
9.10

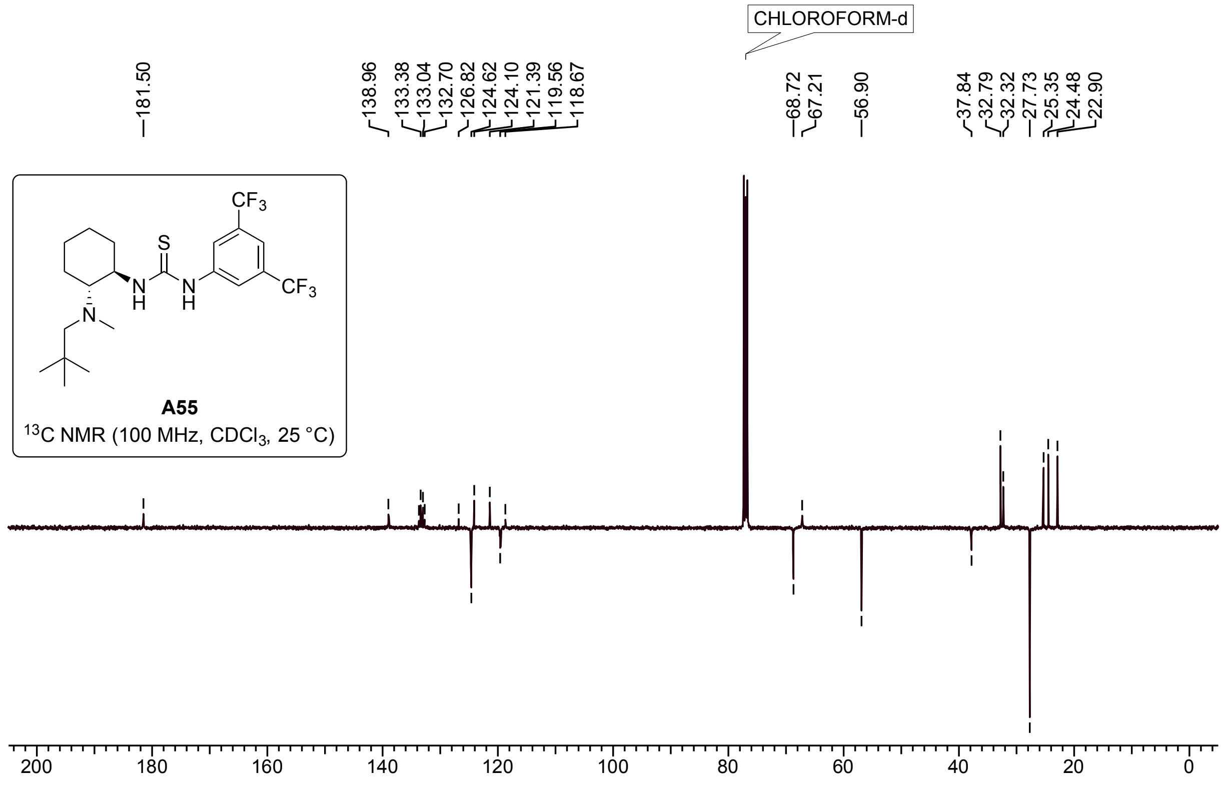
CHLOROFORM-d
181.50
138.96
133.38
133.04
132.70
126.82
124.62
124.10
121.39
119.56
118.67
68.72
67.21
56.90
37.84
32.79
32.32
27.73
25.35
24.48
22.90
A55
^{13}C NMR (100 MHz, $CDCl_3$, 25 °C)

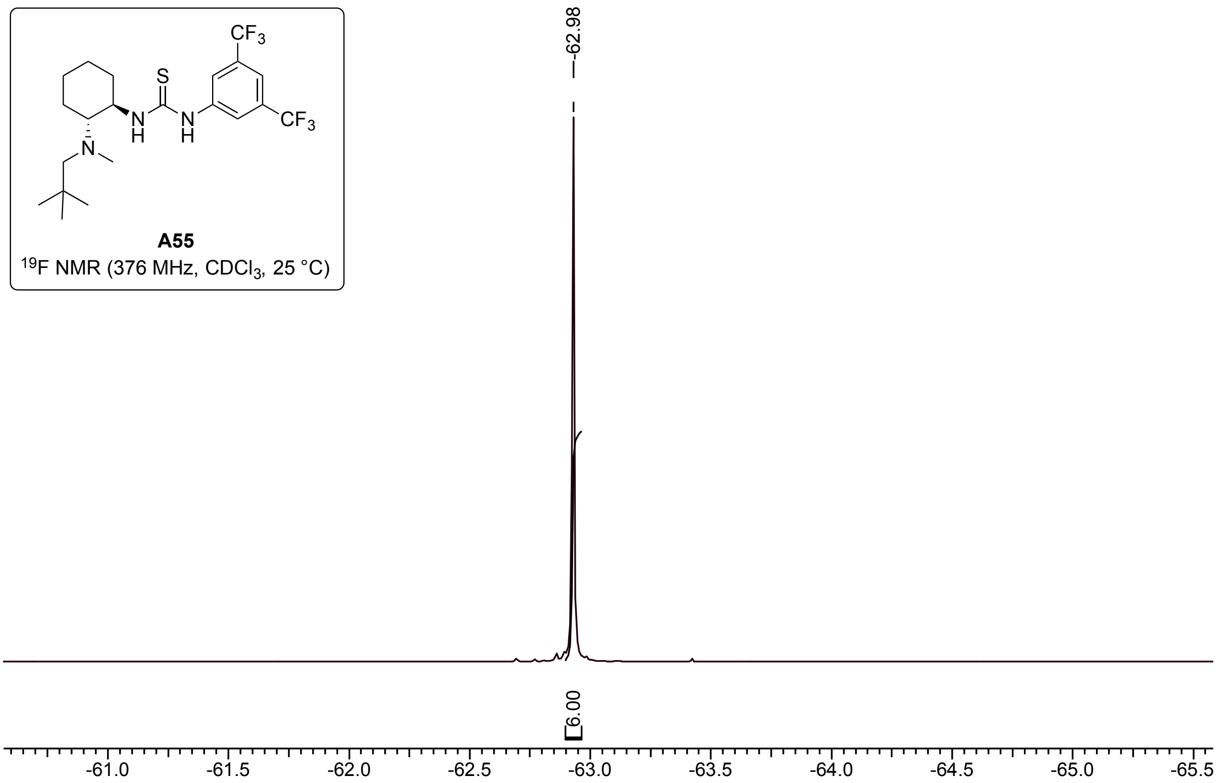
CF3
S
N H
N H
CF3
N
A55
19F NMR (376 MHz, CDCl3, 25 °C)
-62.98
6.00
-61.0 -61.5 -62.0 -62.5 -63.0 -63.5 -64.0 -64.5 -65.0 -65.5

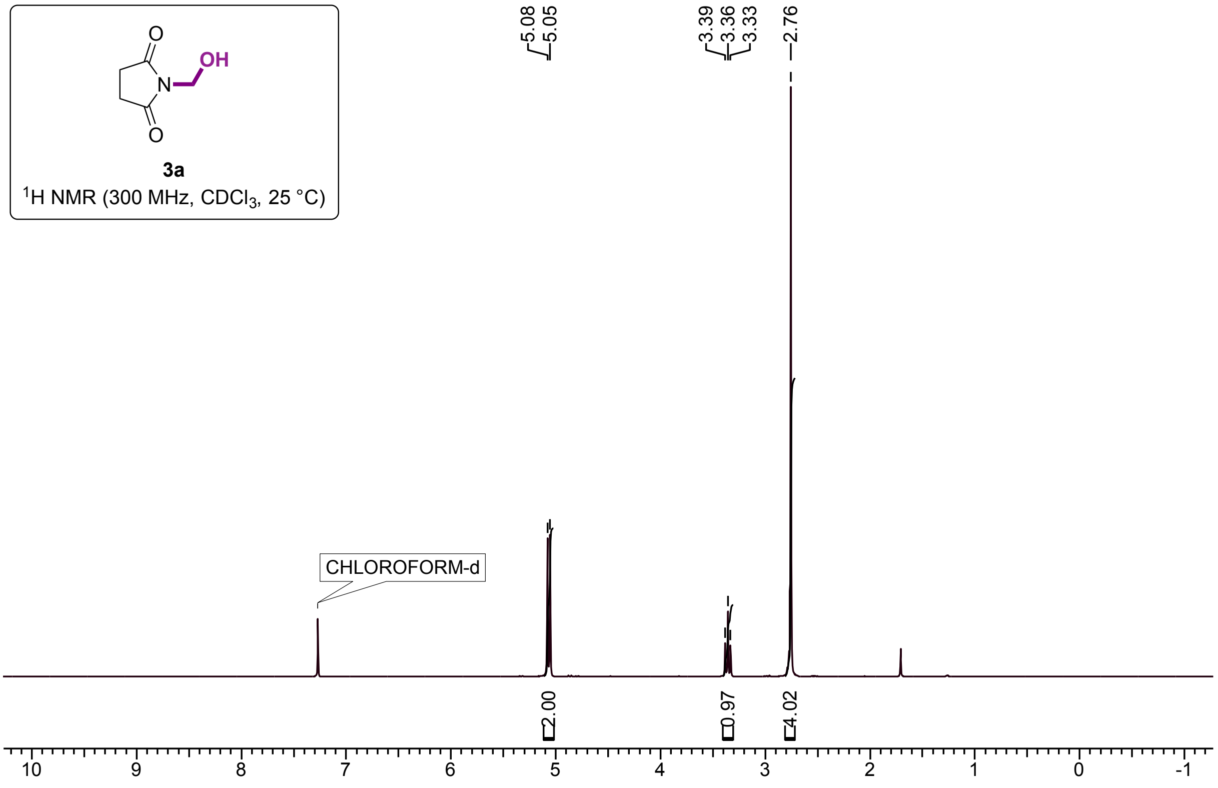
O
N
OH
O
3a
1H NMR (300 MHz, CDCl3, 25 °C)
5.08
5.05
3.39
3.36
3.33
2.76
CHLOROFORM-d
2.00
0.97
4.02
10 9 8 7 6 5 4 3 2 1 0 -1

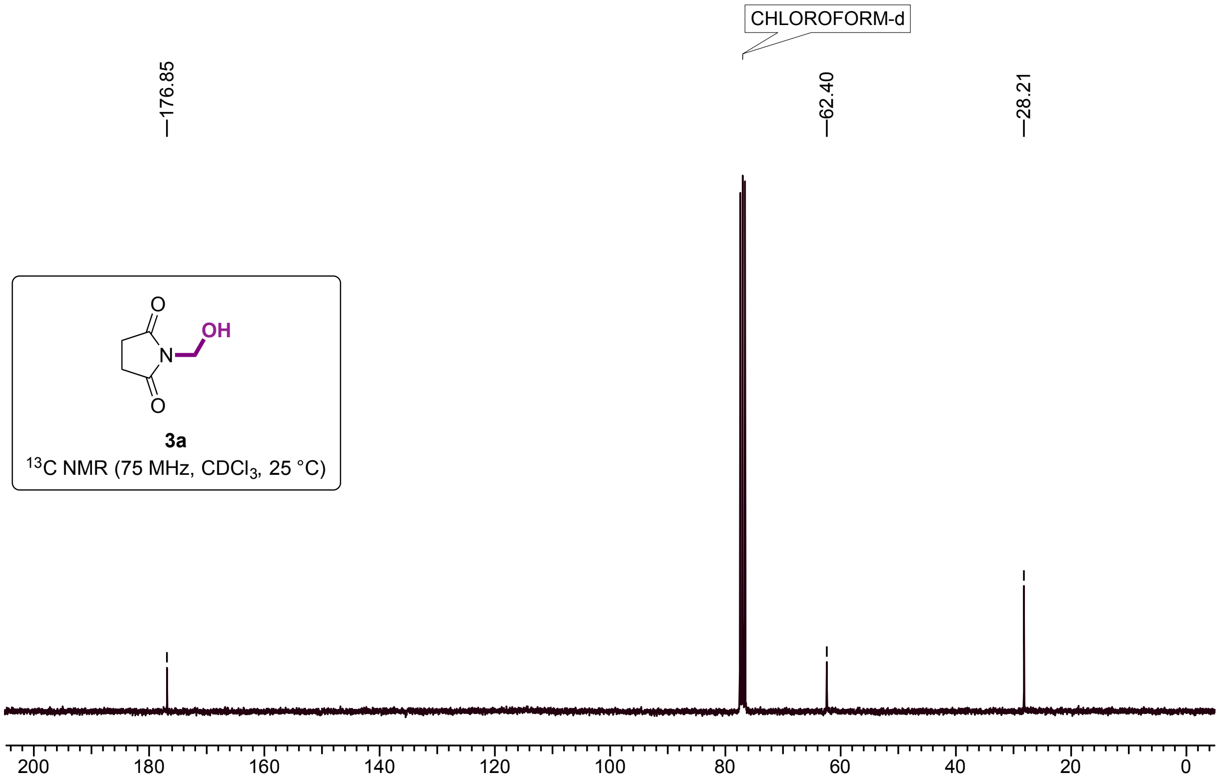
CHLOROFORM-d
176.85
62.40
28.21
OH
N
O
O
3a
^{13}C NMR (75 MHz, $CDCl_3$, 25 °C)
200
180
160
140
120
100
80
60
40
20
0

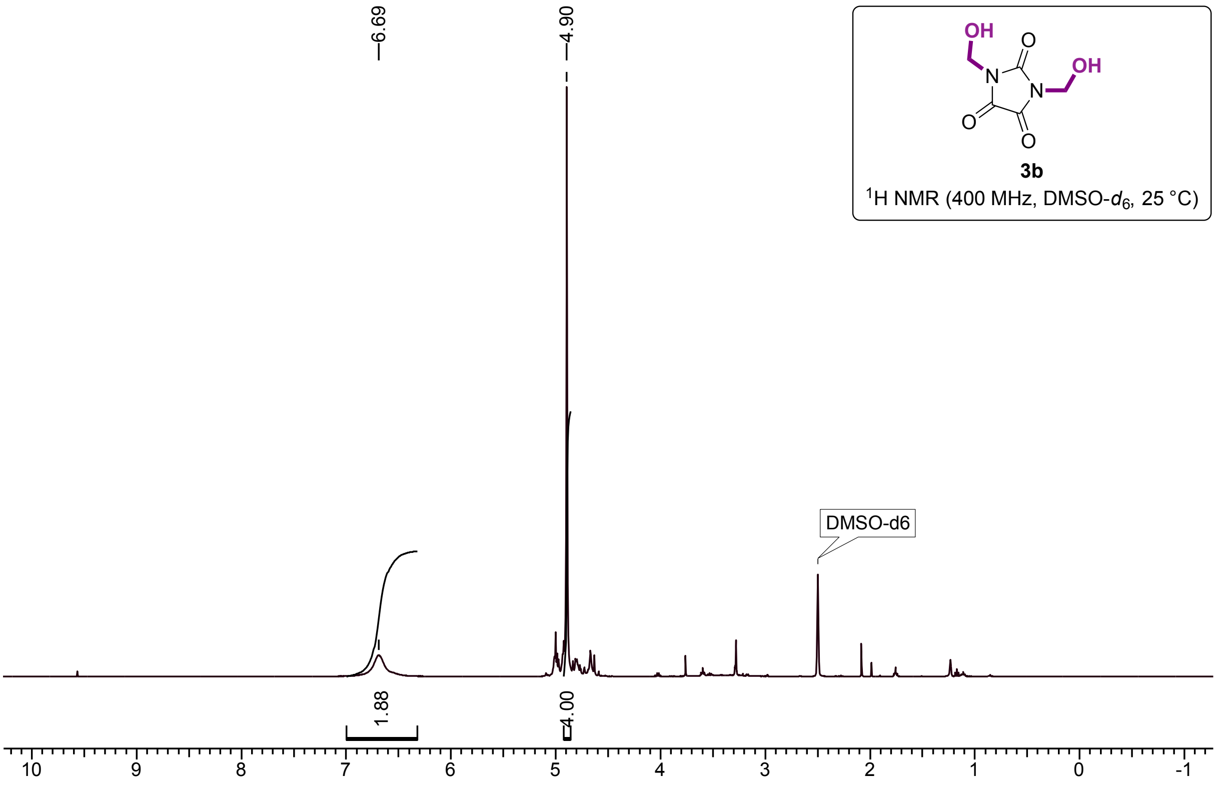
6.69
4.90
OH
O
N
N
OH
O
O
3b
^{1}H NMR (400 MHz, DMSO-d_6, 25 °C)
DMSO-d6
1.88
4.00
10
9
8
7
6
5
4
3
2
1
0
-1

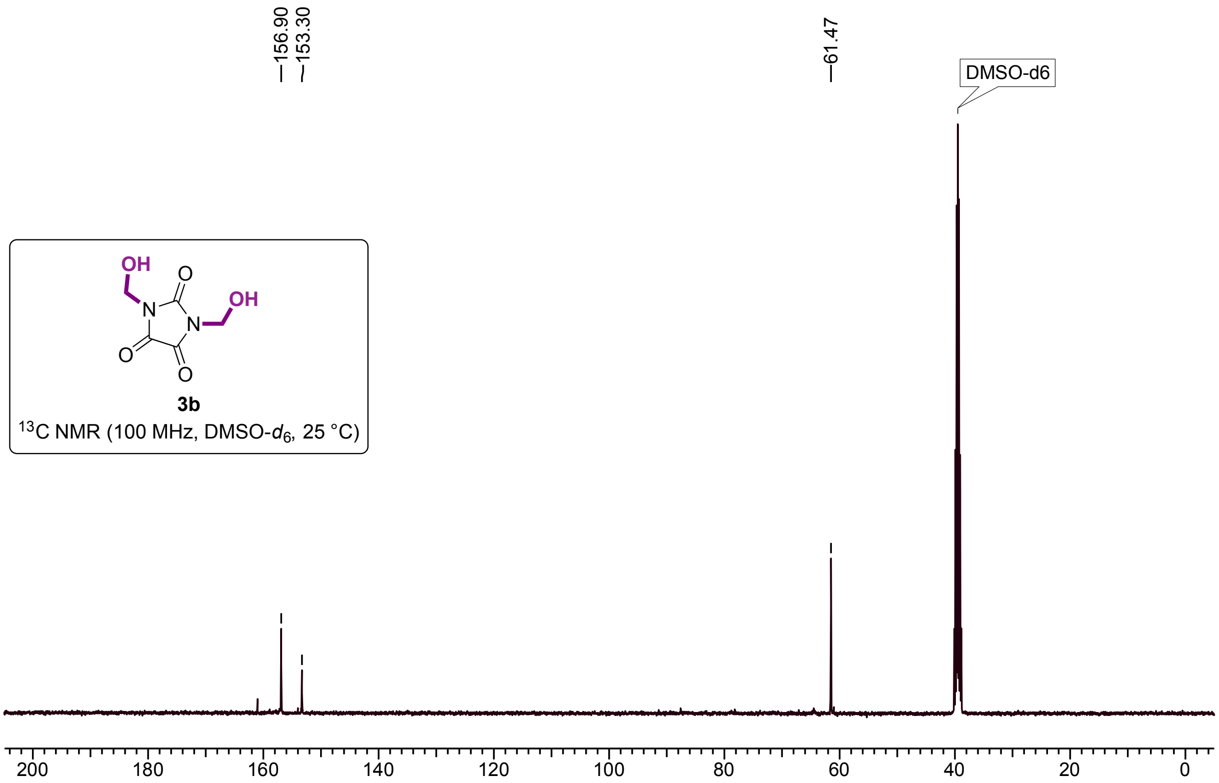
156.90
153.30
61.47
DMSO-d6
OH
O
N
OH
N
O
O
3b
13C NMR (100 MHz, DMSO-d6, 25 °C)
200 180 160 140 120 100 80 60 40 20 0

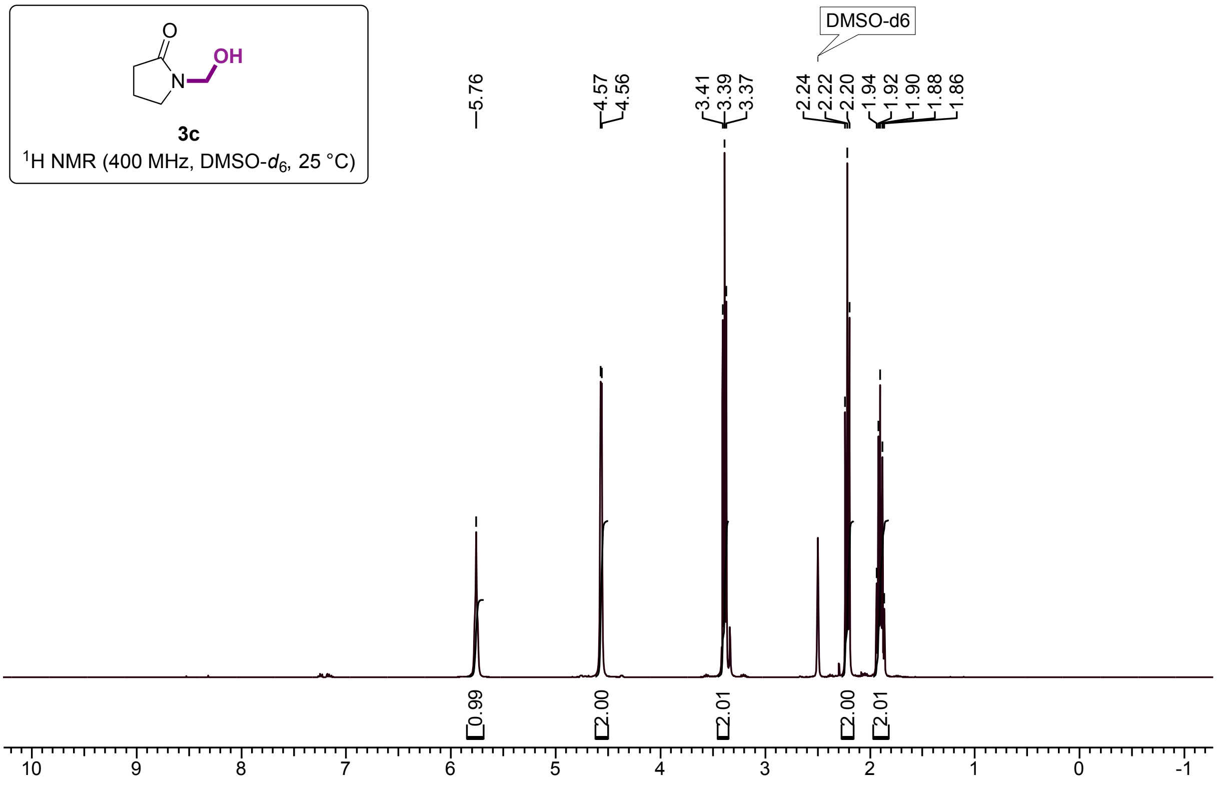
O
OH
N
3c
1H NMR (400 MHz, DMSO-d6, 25 °C)
DMSO-d6
5.76
4.57
4.56
3.41
3.39
3.37
2.24
2.22
2.20
1.94
1.92
1.90
1.88
1.86
0.99
2.00
2.01
2.00
2.01
10 9 8 7 6 5 4 3 2 1 0 -1

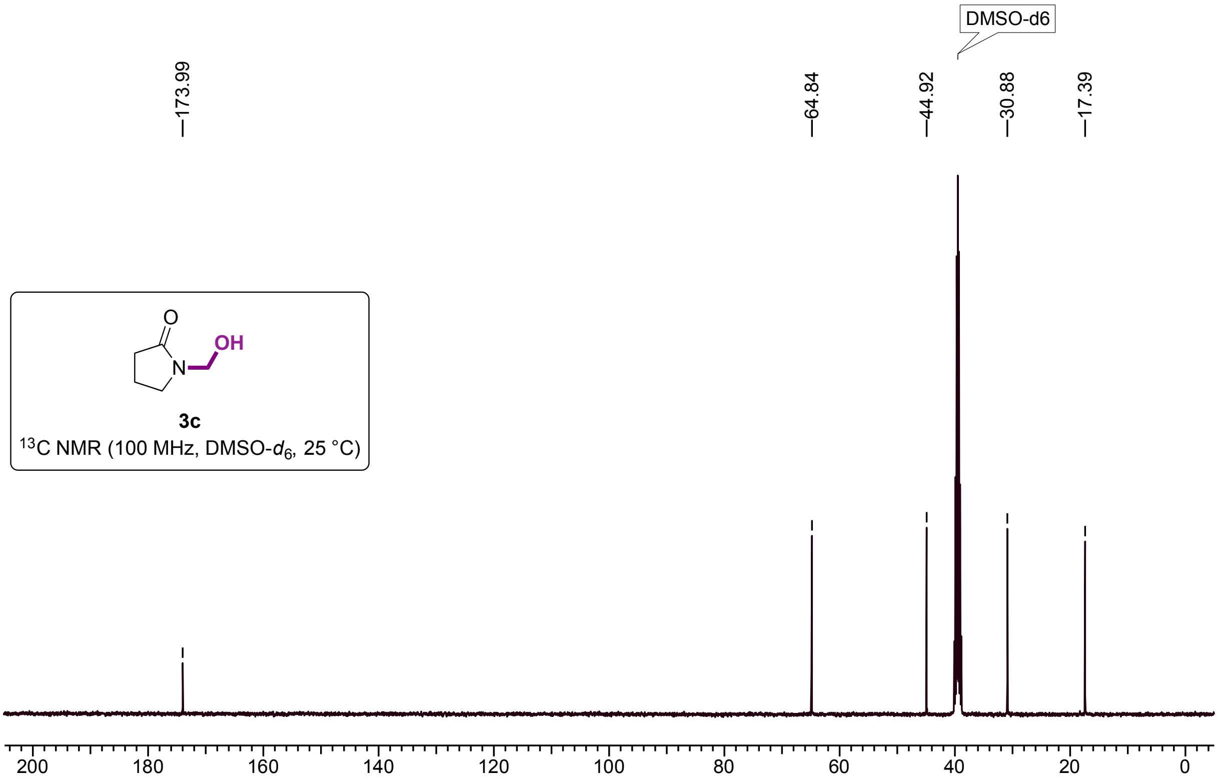
173.99
64.84
44.92
30.88
17.39
DMSO-d6
3c
^{13}C NMR (100 MHz, DMSO-d_6, 25 °C)
200 180 160 140 120 100 80 60 40 20 0

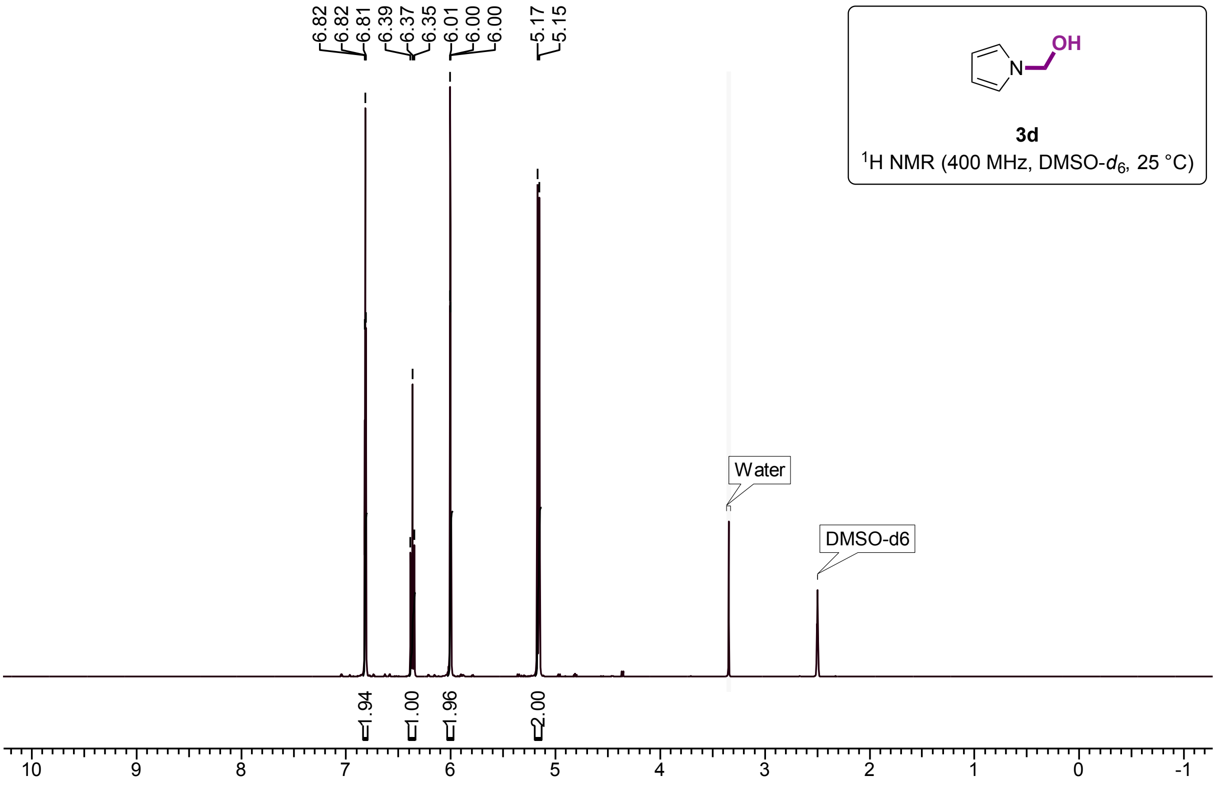
6.82
6.82
6.81
6.39
6.37
6.35
6.01
6.00
6.00
5.17
5.15
3d
^{1}H NMR (400 MHz, DMSO-d_6, 25 °C)
Water
DMSO-d6
1.94
1.00
1.96
2.00
10 9 8 7 6 5 4 3 2 1 0 -1

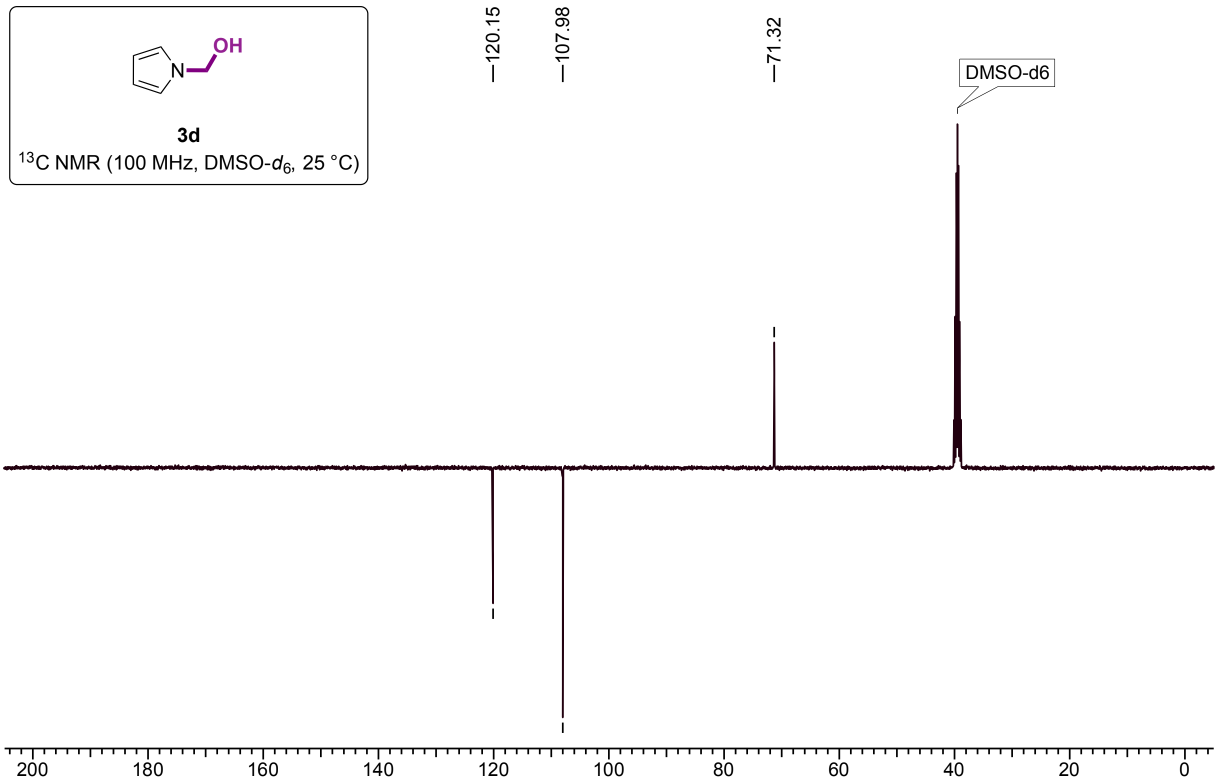
OH
N
3d
13C NMR (100 MHz, DMSO-d6, 25 °C)
120.15
107.98
71.32
DMSO-d6
200
180
160
140
120
100
80
60
40
20
0

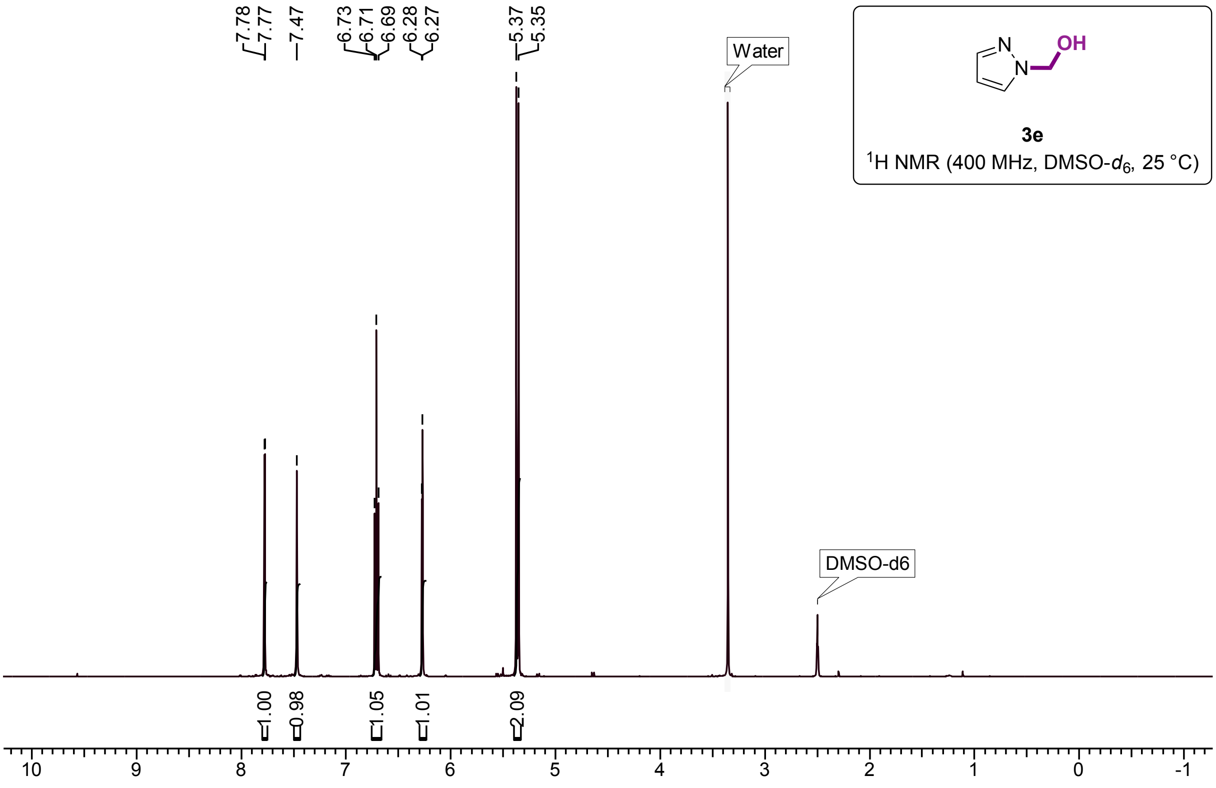
7.78
7.77
7.47
6.73
6.71
6.69
6.28
6.27
5.37
5.35
Water
N
N
OH
3e
1H NMR (400 MHz, DMSO-d6, 25 °C)
DMSO-d6
1.00
0.98
1.05
1.01
2.09
10
9
8
7
6
5
4
3
2
1
0
-1

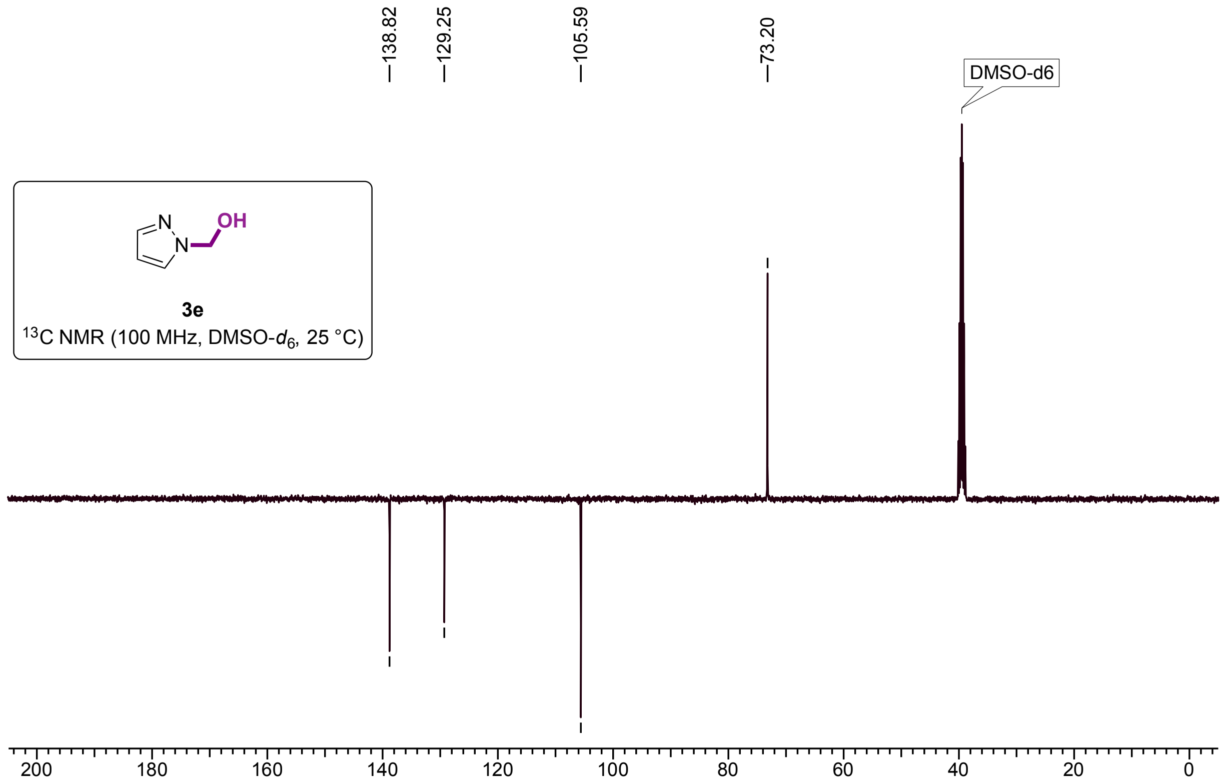
138.82
129.25
105.59
73.20
DMSO-d6
3e
^{13}C NMR (100 MHz, DMSO-d_6, 25 °C)
200 180 160 140 120 100 80 60 40 20 0

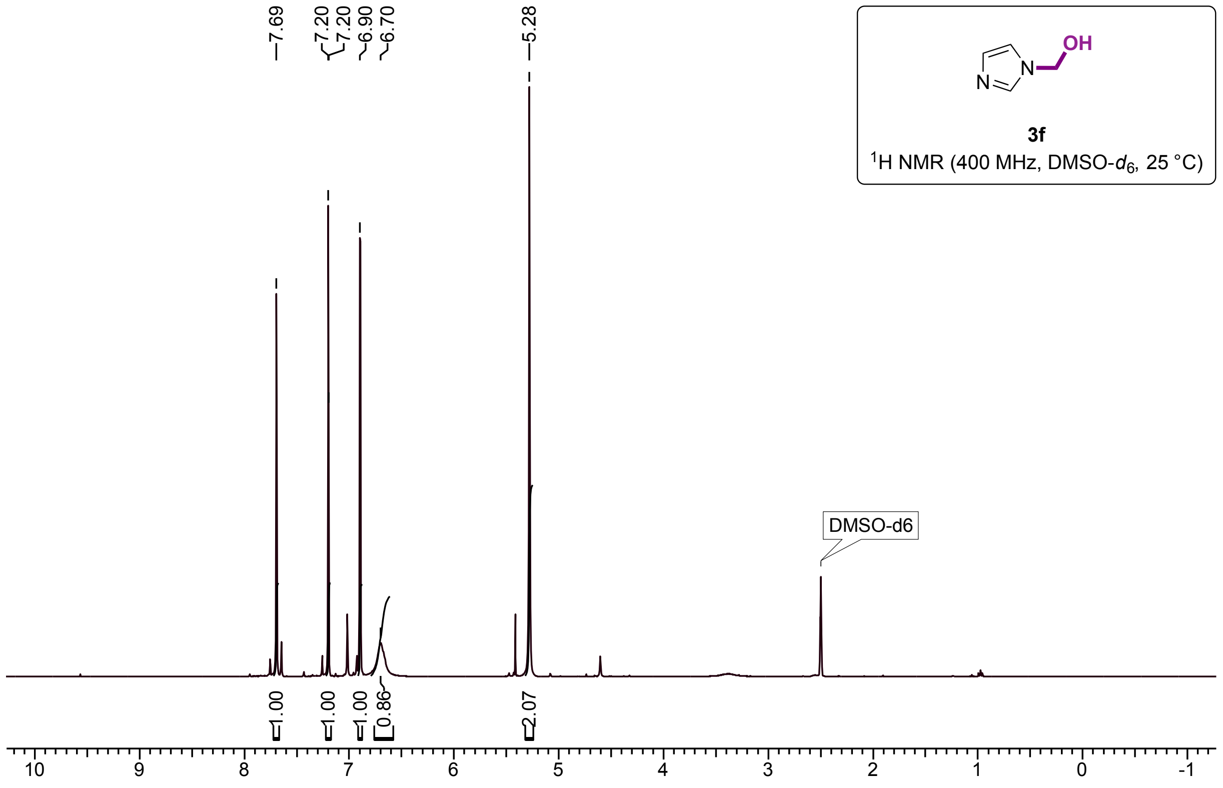
7.69
7.20
7.20
6.90
6.70
5.28
3f
^{1}H NMR (400 MHz, DMSO-d_6, 25 °C)
DMSO-d6
1.00
1.00
1.00
0.86
2.07
10 9 8 7 6 5 4 3 2 1 0 -1

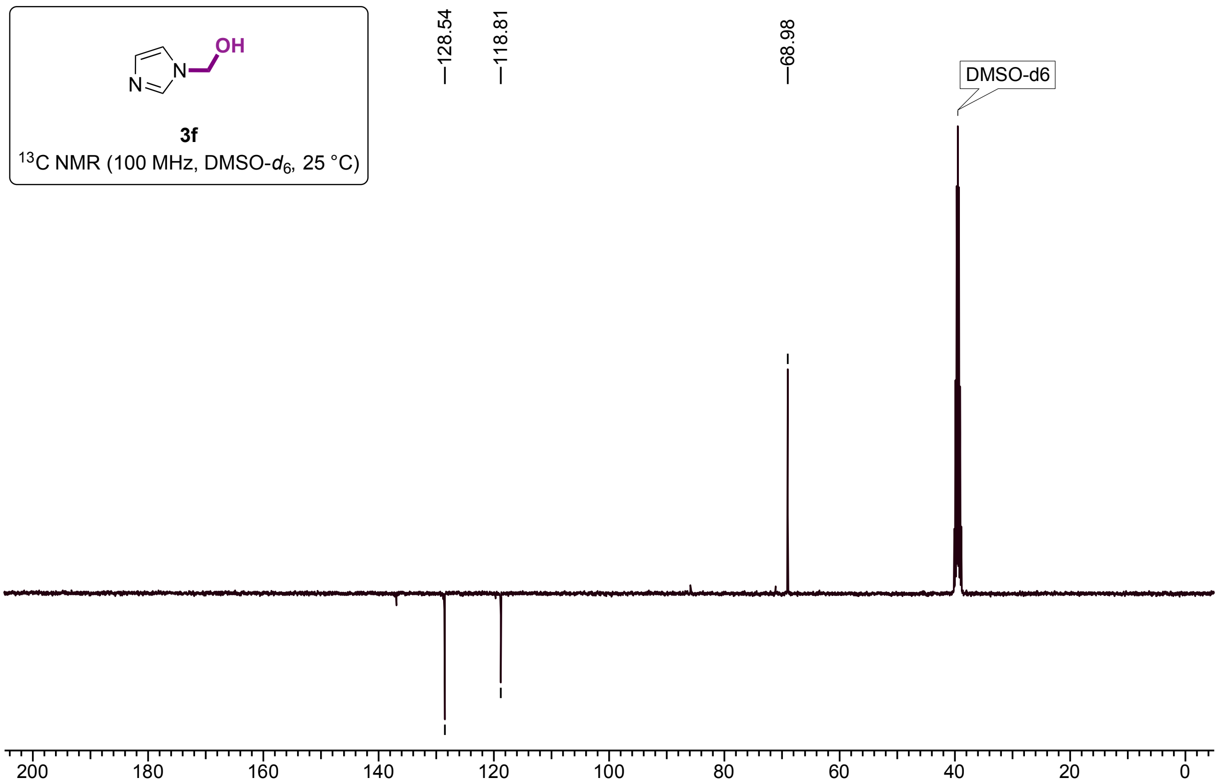
OH
N
N
3f
^{13}C NMR (100 MHz, DMSO-d_6, 25 °C)
128.54
118.81
68.98
DMSO-d6
200 180 160 140 120 100 80 60 40 20 0

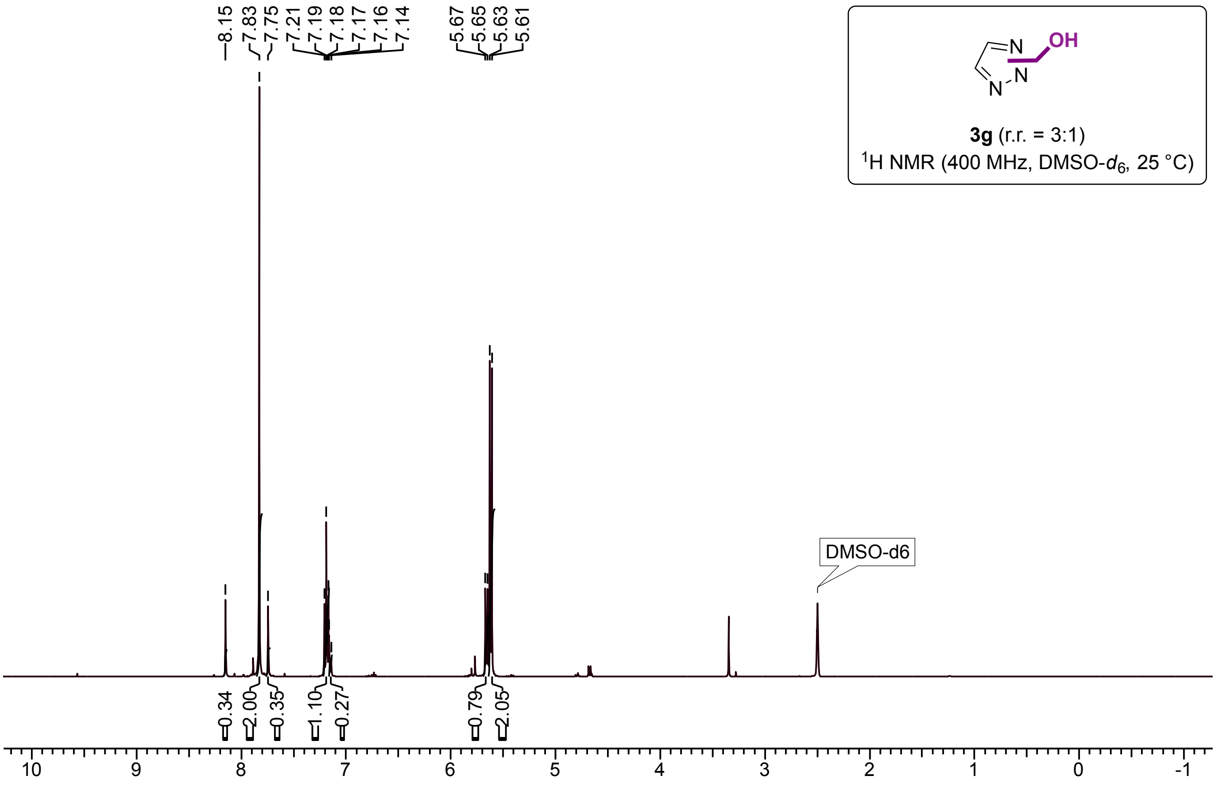
8.15 7.83 7.75 7.21 7.19 7.18 7.17 7.16 7.14
5.67 5.65 5.63 5.61
N
OH
N
N
3g (r.r. = 3:1)
^{1}H NMR (400 MHz, DMSO-d_6, 25 °C)
DMSO-d6
0.34 2.00 0.35 1.10 0.27 0.79 2.05
10 9 8 7 6 5 4 3 2 1 0 -1

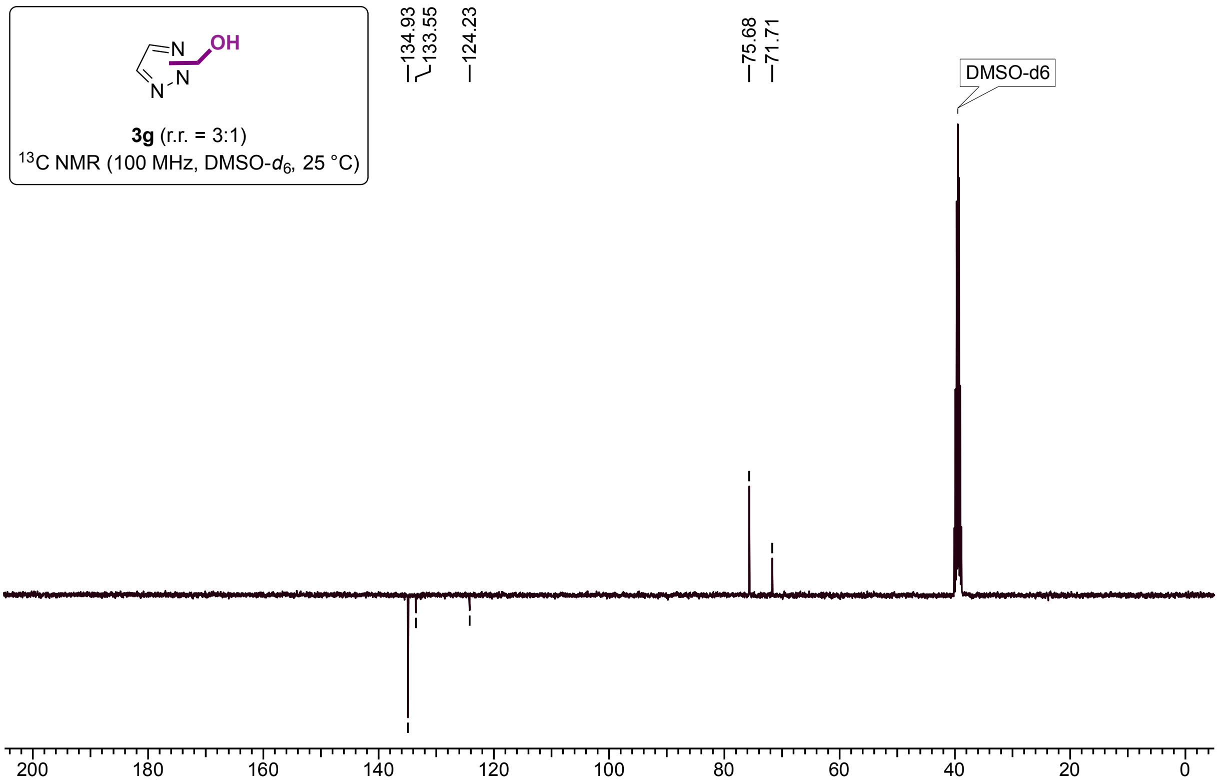

3g (r.r. = 3:1)
^{13}C NMR (100 MHz, DMSO-d_6, 25 °C)

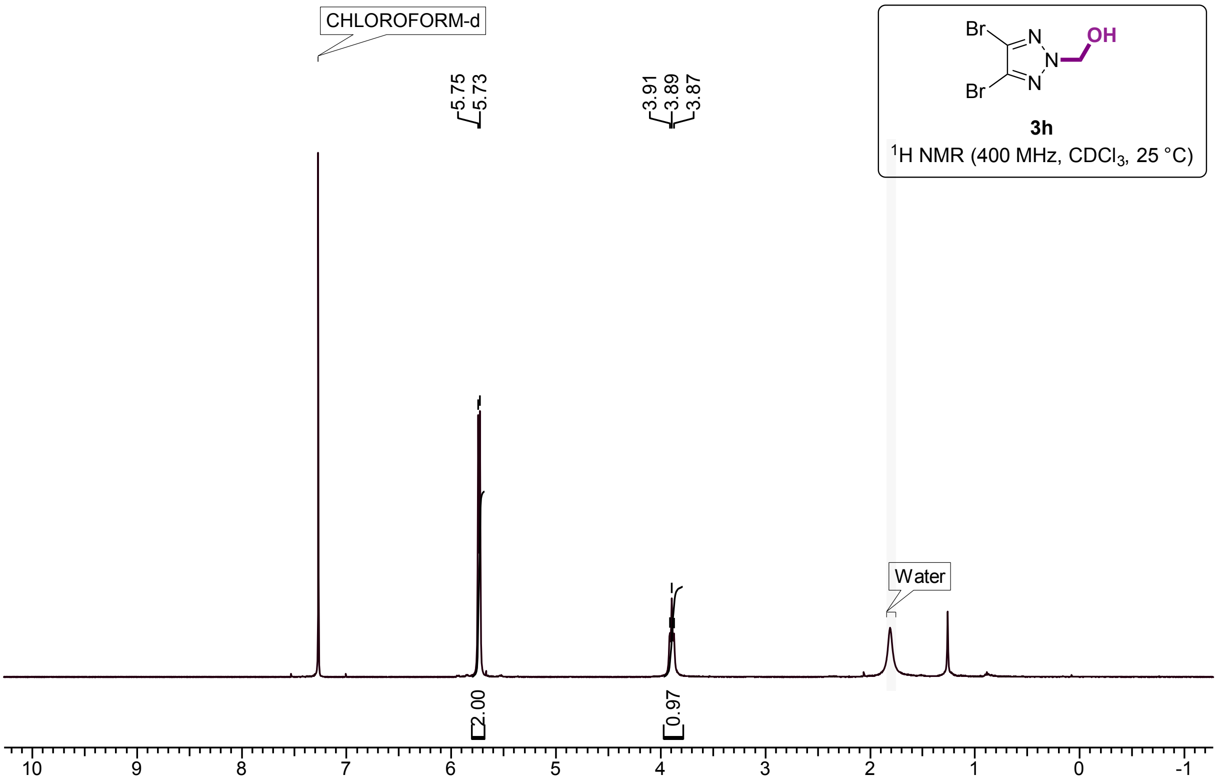

3h
^{1}H NMR (400 MHz, $CDCl_3$, 25 °C)

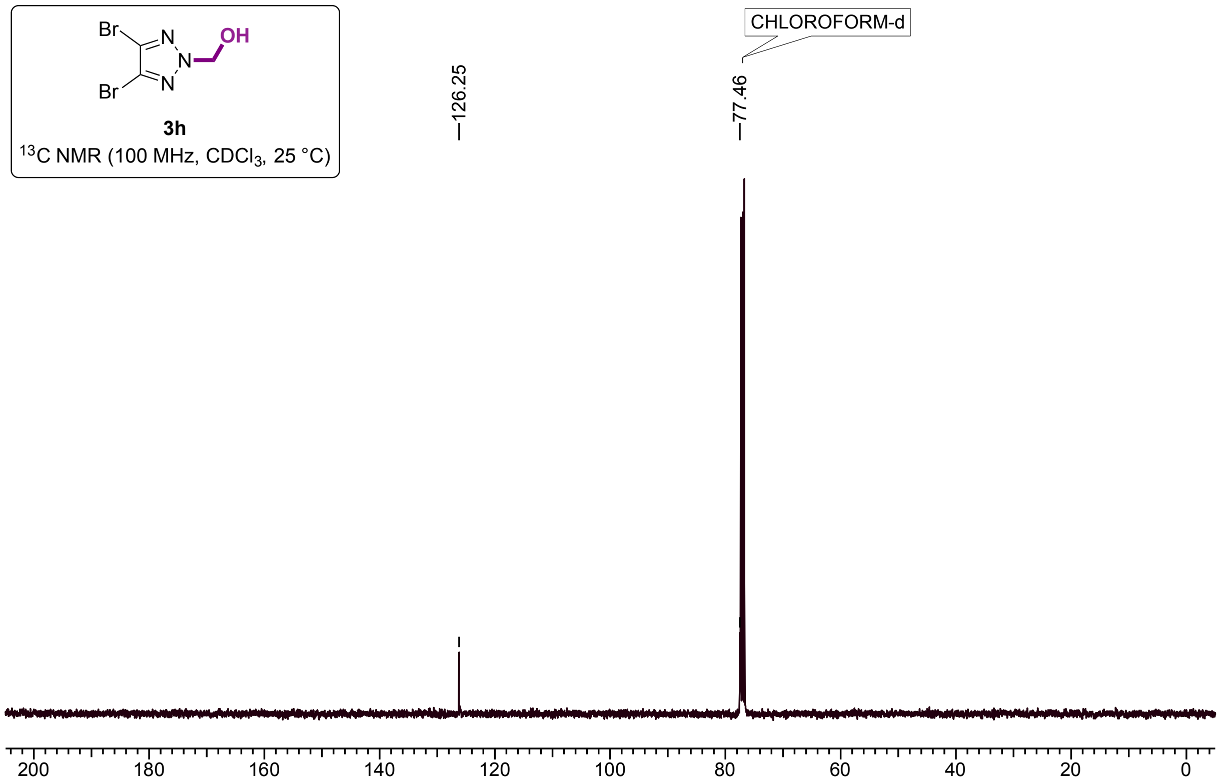
Br
N
N
N
OH
Br
3h
13C NMR (100 MHz, CDCl3, 25 °C)
126.25
77.46
CHLOROFORM-d
200
180
160
140
120
100
80
60
40
20
0

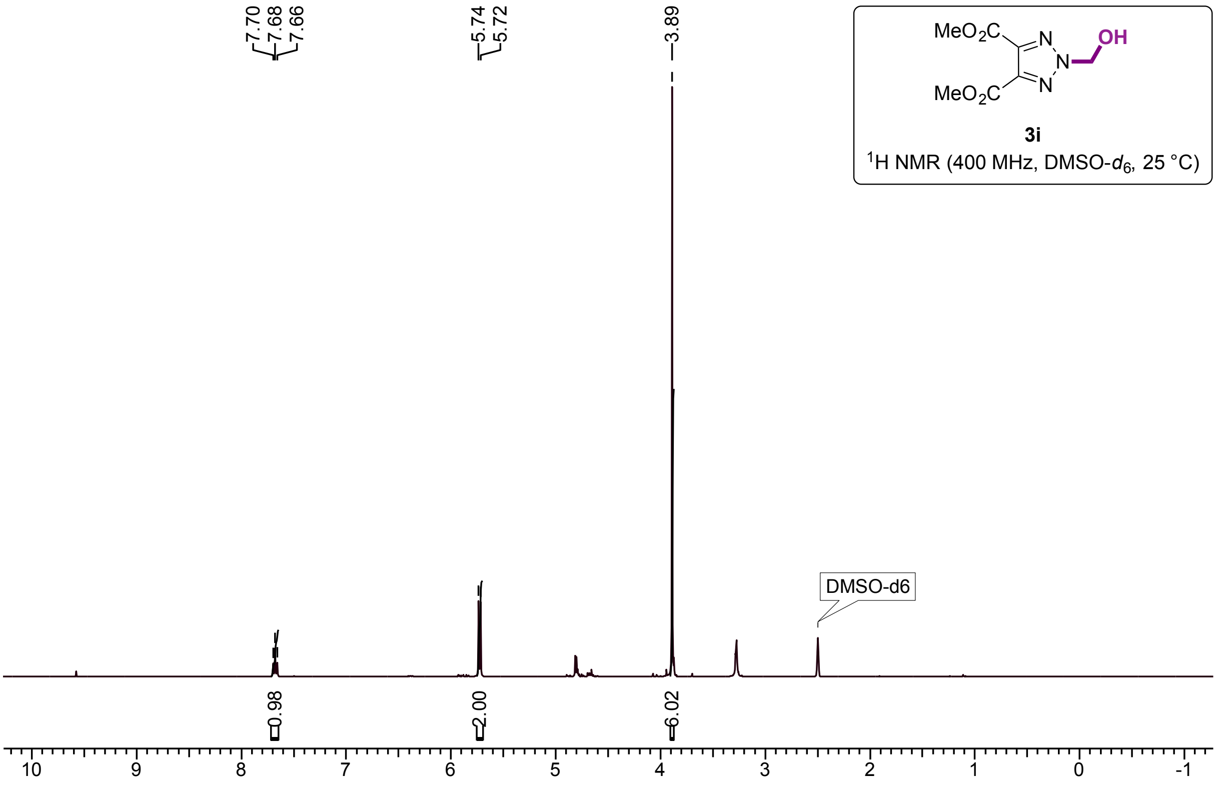
7.70
7.68
7.66
5.74
5.72
3.89
MeO2C
N
N
OH
MeO2C
N
3i
1H NMR (400 MHz, DMSO-d6, 25 °C)
DMSO-d6
0.98
2.00
6.02
10
9
8
7
6
5
4
3
2
1
0
-1

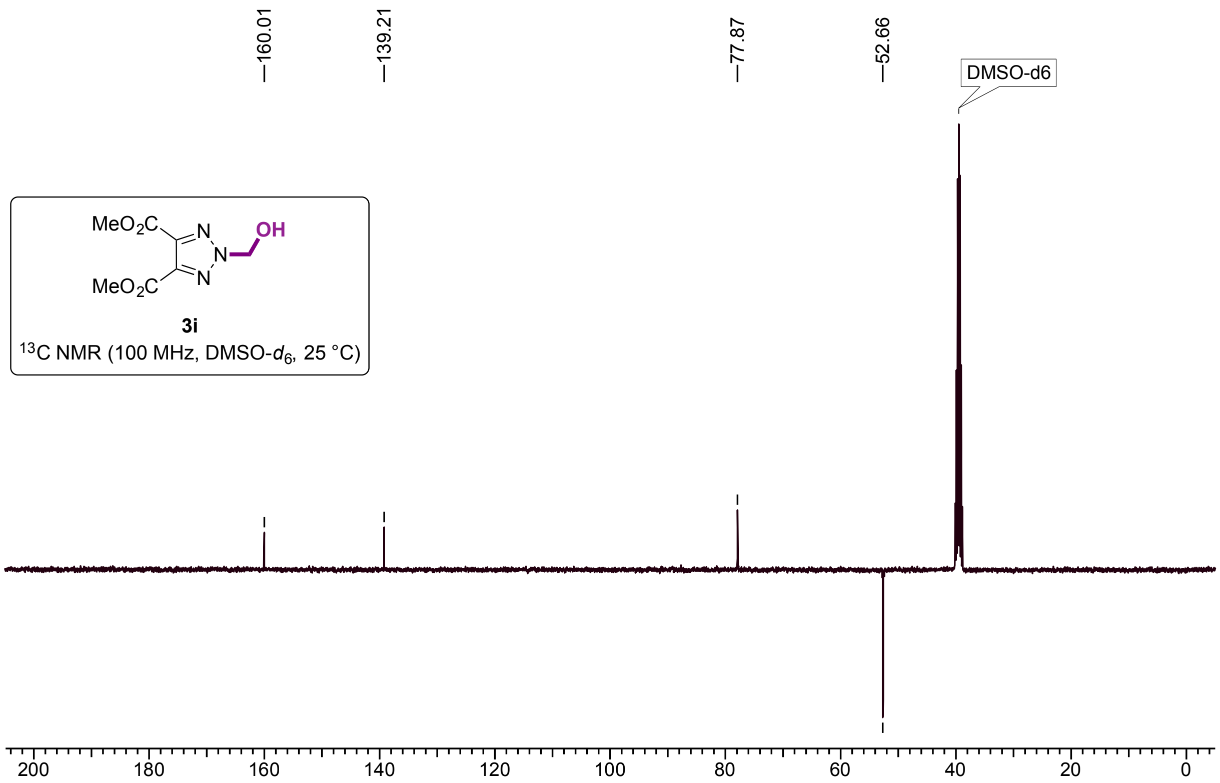
160.01
139.21
77.87
52.66
DMSO-d6
MeO2C
N
N
N
OH
MeO2C
3i
13C NMR (100 MHz, DMSO-d6, 25 °C)
200
180
160
140
120
100
80
60
40
20
0

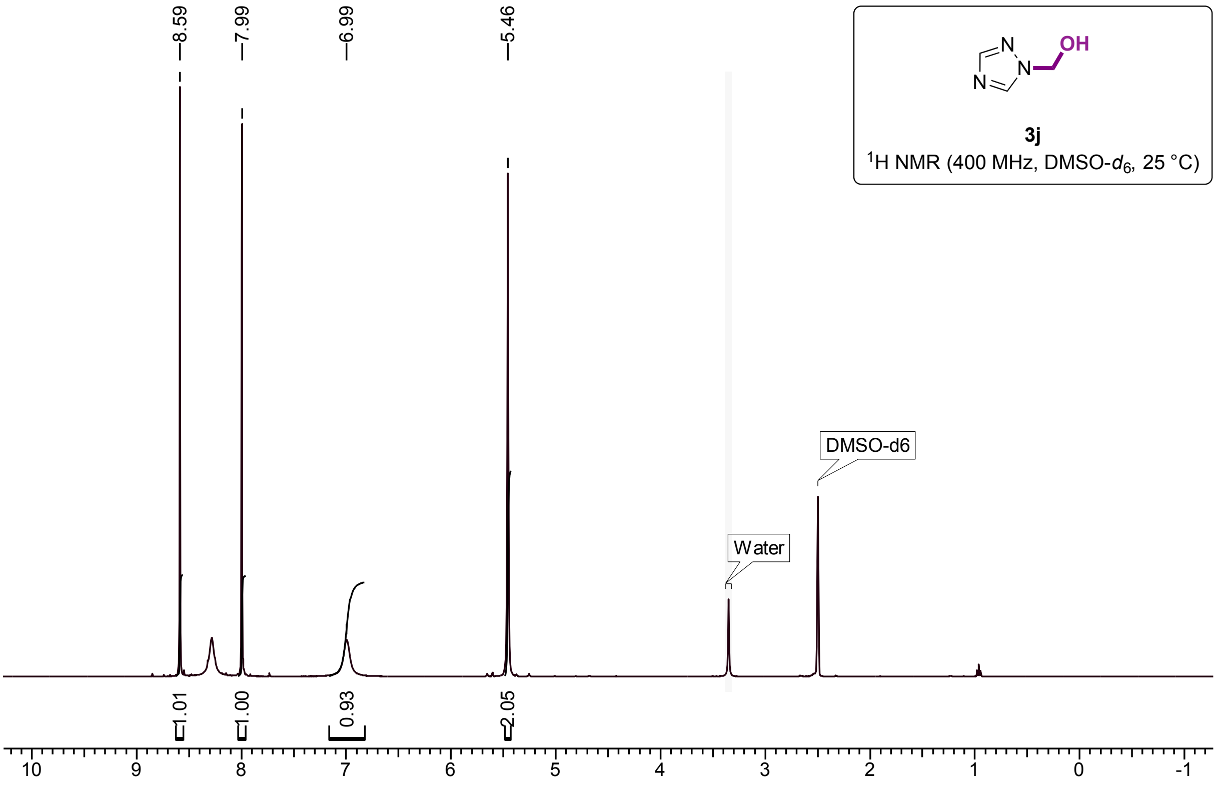
8.59
7.99
6.99
5.46
N
N
N
OH
3j
1H NMR (400 MHz, DMSO-d6, 25 °C)
DMSO-d6
Water
1.01
1.00
0.93
2.05
10
9
8
7
6
5
4
3
2
1
0
-1

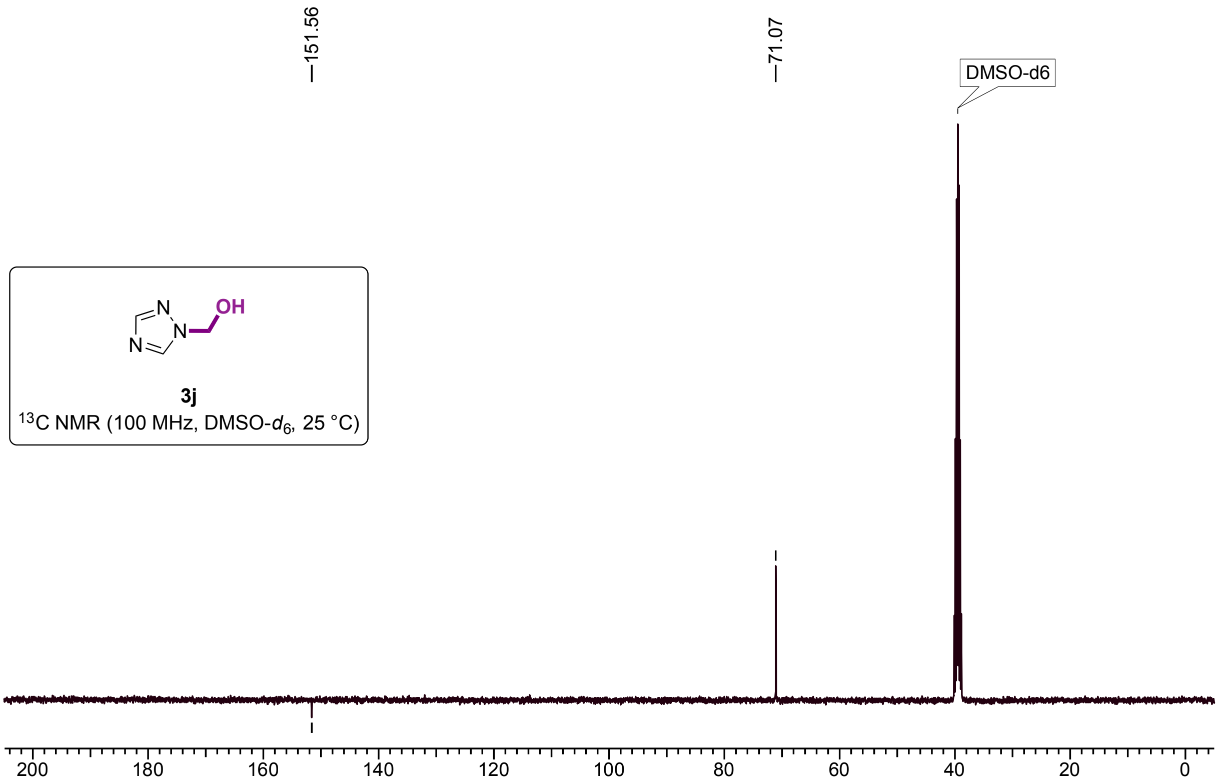

151.56
71.07
DMSO-d6
N
N
N
OH
3j
^{13}C NMR (100 MHz, DMSO-d_6, 25 °C)
200
180
160
140
120
100
80
60
40
20
0

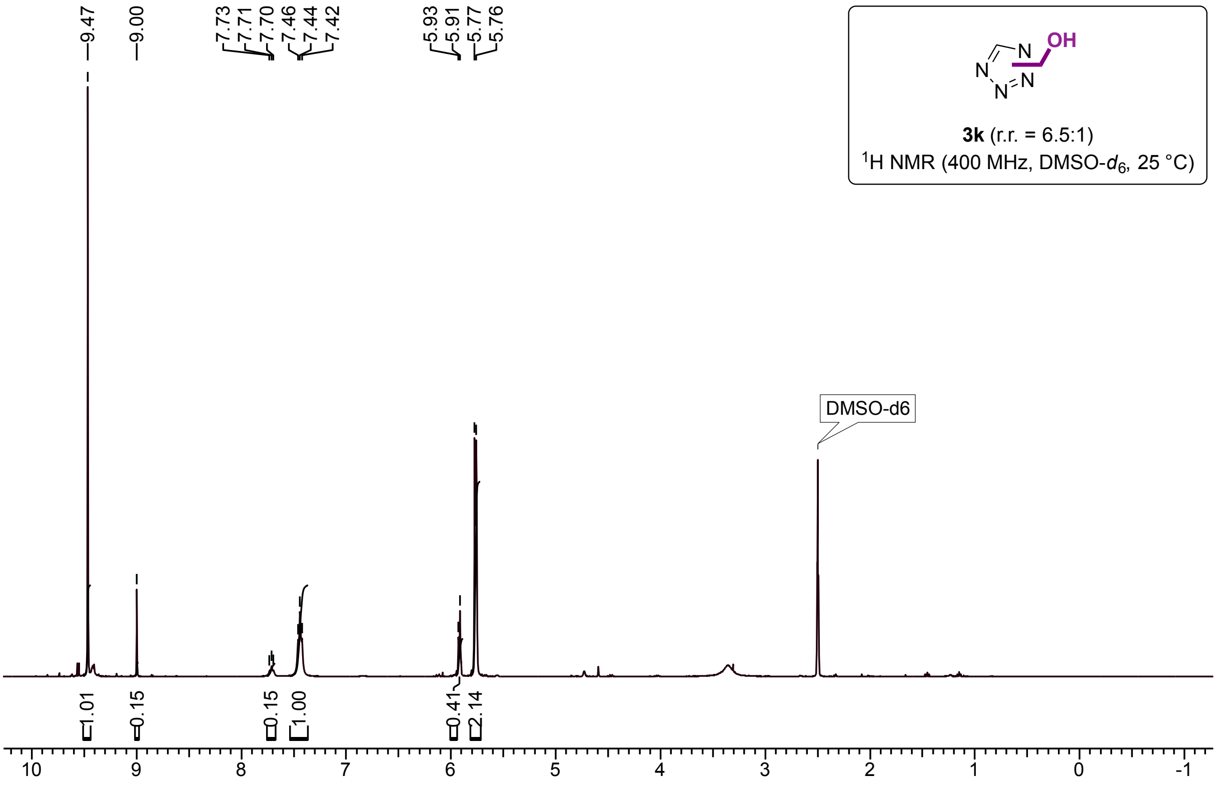

9.47
9.00
7.73
7.71
7.70
7.46
7.44
7.42
5.93
5.91
5.77
5.76
N
N
N
N
OH
3k (r.r. = 6.5:1)
^{1}H NMR (400 MHz, DMSO-d_6, 25 °C)
DMSO-d6
1.01
0.15
0.15
1.00
0.41
2.14
10
9
8
7
6
5
4
3
2
1
0
-1

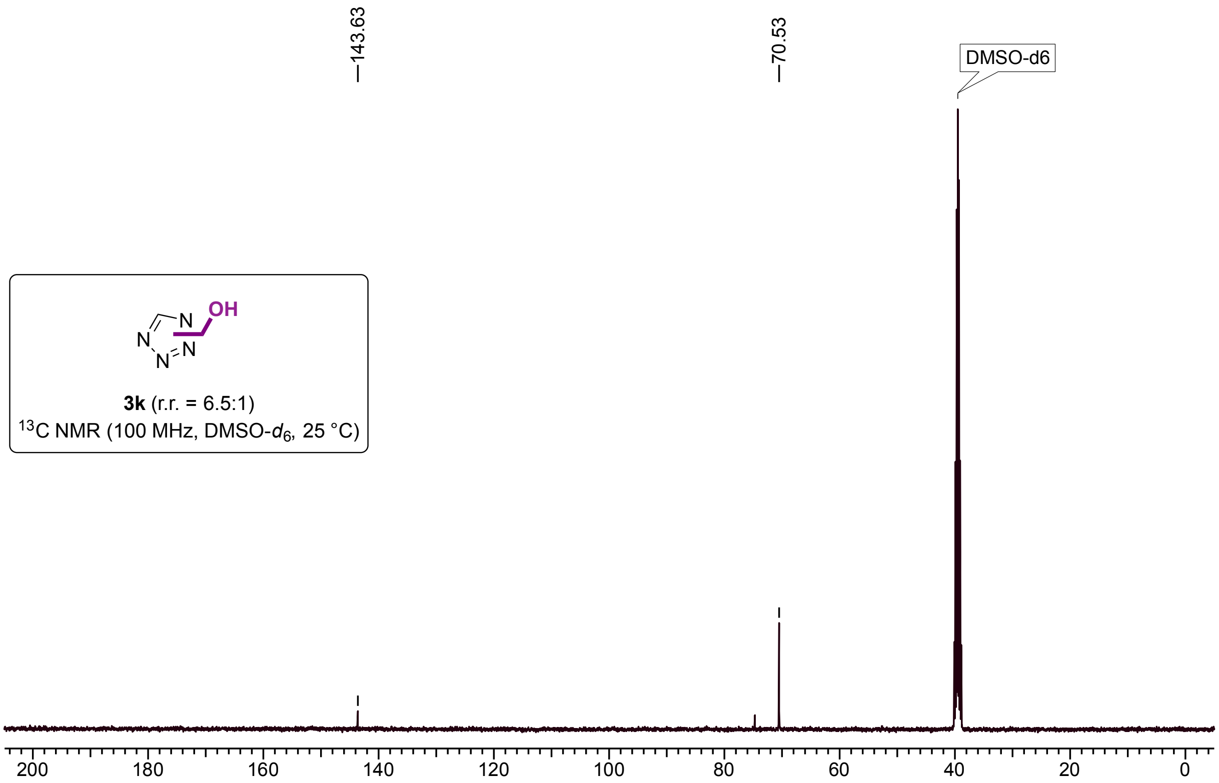

143.63
70.53
DMSO-d6
OH
N
N
N
N
3k (r.r. = 6.5:1)
13C NMR (100 MHz, DMSO-d6, 25 °C)
200
180
160
140
120
100
80
60
40
20
0

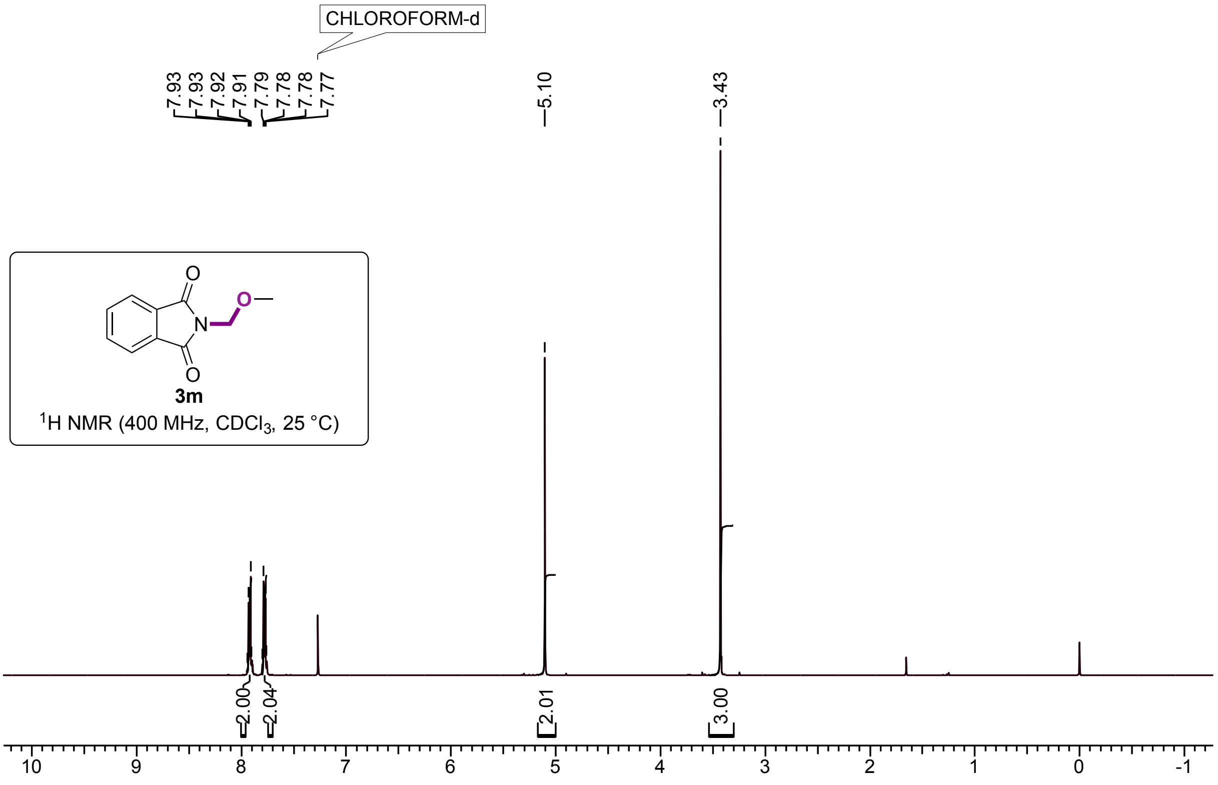

CHLOROFORM-d
7.93
7.93
7.92
7.91
7.79
7.78
7.78
7.77
5.10
3.43
O
N
O
O
3m
1H NMR (400 MHz, CDCl3, 25 °C)
2.00
2.04
2.01
3.00
10
9
8
7
6
5
4
3
2
1
0
-1

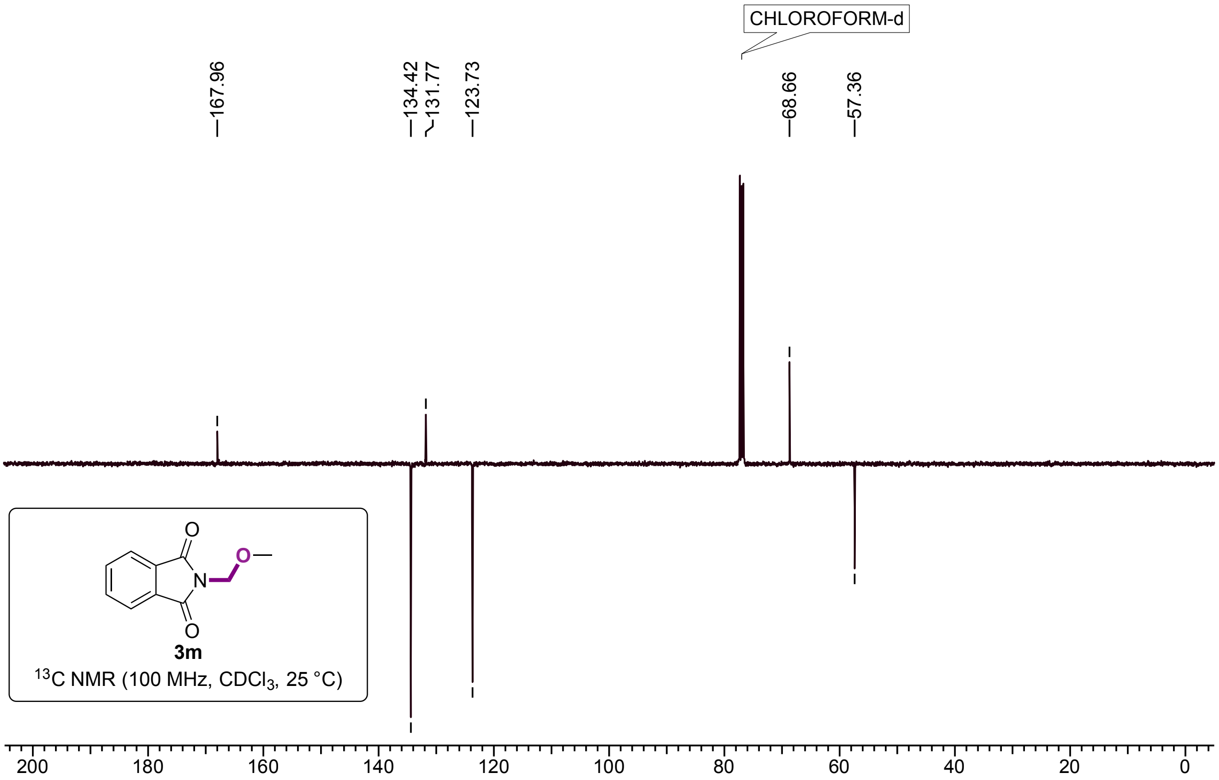
CHLOROFORM-d
167.96
134.42
131.77
123.73
68.66
57.36
3m
^{13}C NMR (100 MHz, CDCl$_3$, 25 °C)
200
180
160
140
120
100
80
60
40
20
0

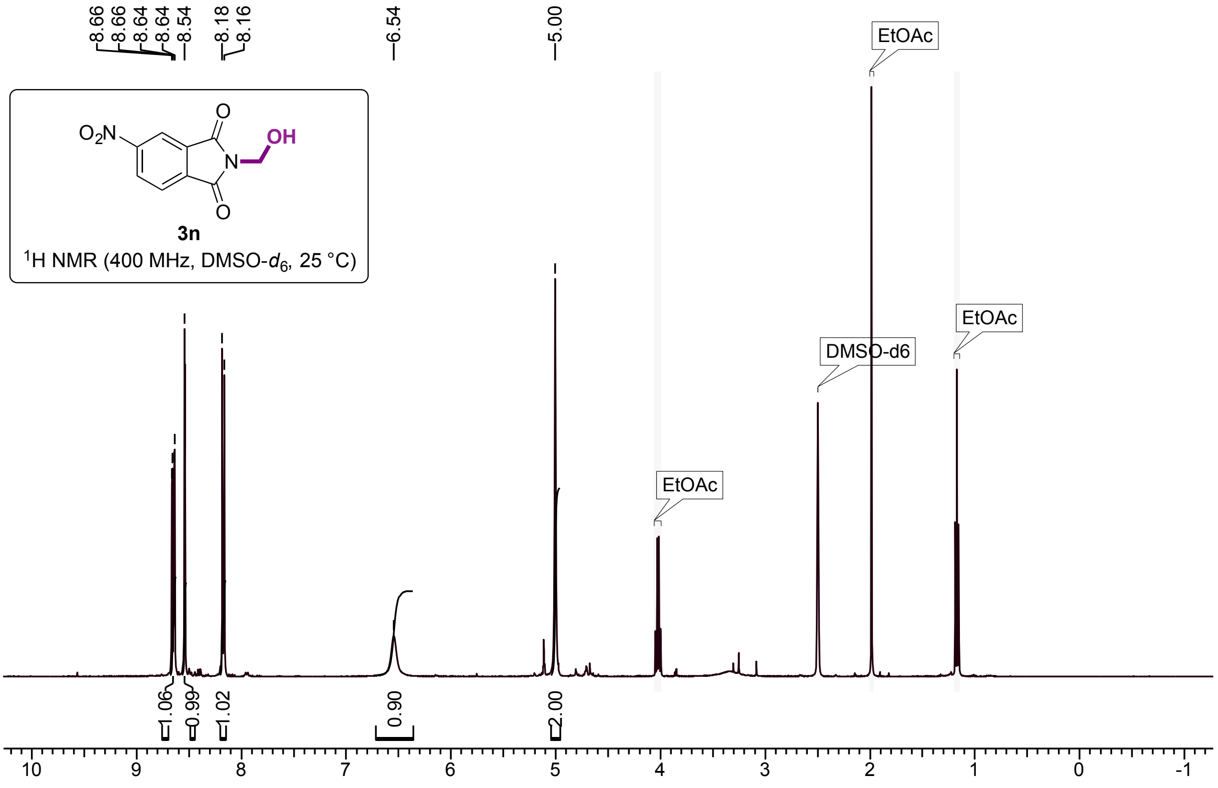
8.66
8.66
8.64
8.64
8.54
8.18
8.16
6.54
5.00
EtOAc
O_2N
OH
3n
^{1}H NMR (400 MHz, DMSO-d_6, 25 °C)
EtOAc
DMSO-d6
EtOAc
1.06
0.99
1.02
0.90
2.00
10
9
8
7
6
5
4
3
2
1
0
-1

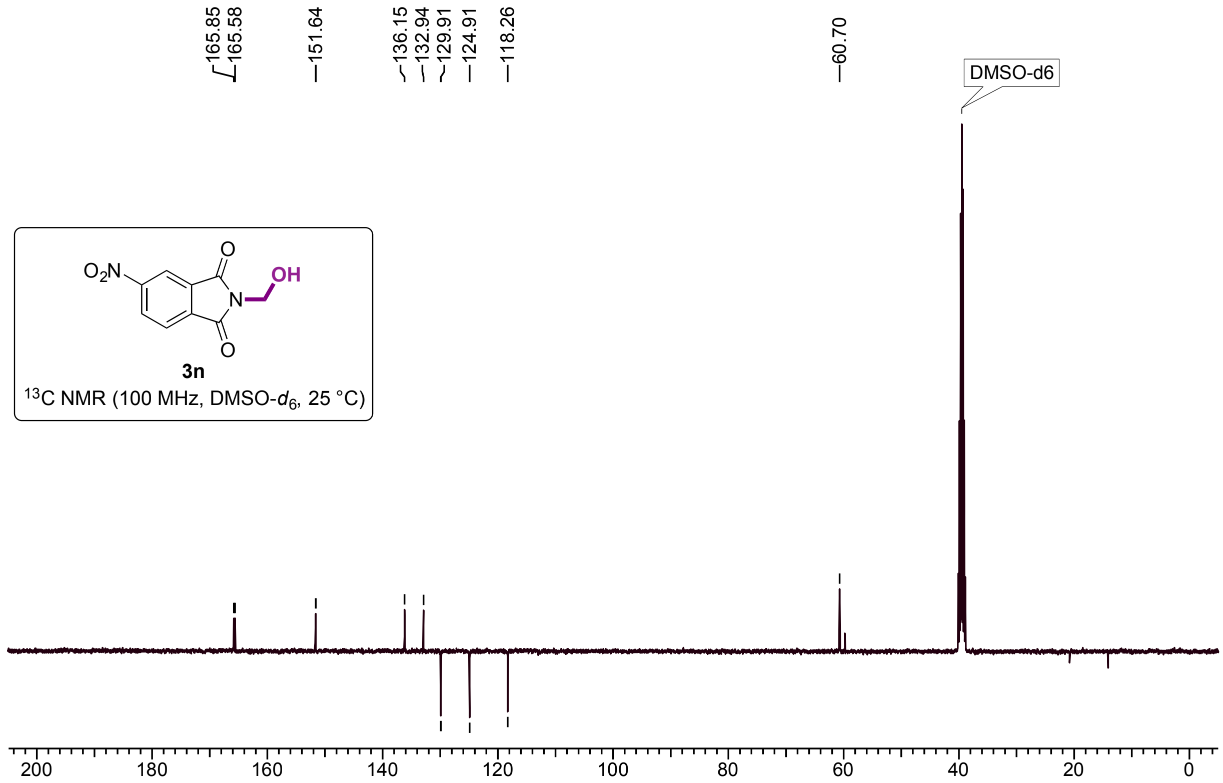
165.85
165.58
151.64
136.15
132.94
129.91
124.91
118.26
60.70
DMSO-d6
O_2N
O
N
OH
O
3n
^{13}C NMR (100 MHz, DMSO-d_6, 25 °C)
200
180
160
140
120
100
80
60
40
20
0

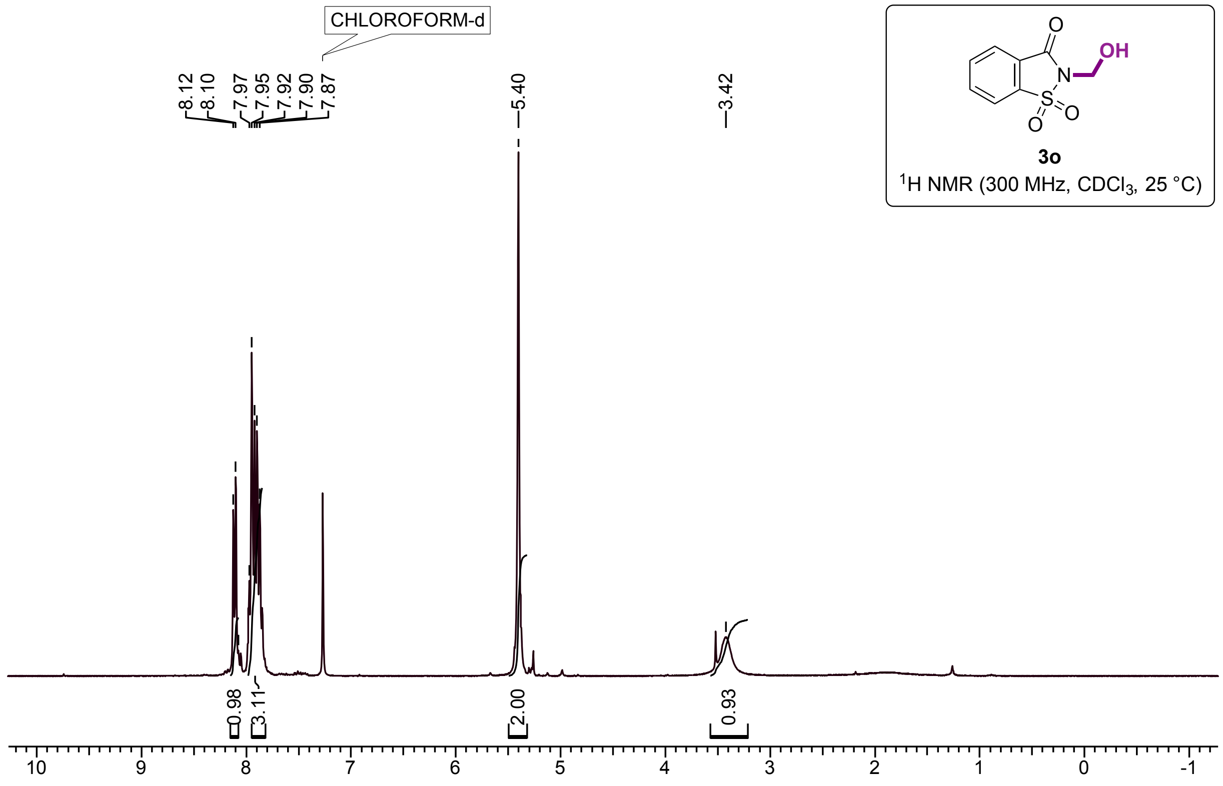
CHLOROFORM-d
8.12
8.10
7.97
7.95
7.92
7.90
7.87
5.40
3.42
O
N
OH
S
O
O
3o
^1H NMR (300 MHz, $CDCl_3$, 25 °C)
0.98
3.11
2.00
0.93
10
9
8
7
6
5
4
3
2
1
0
-1

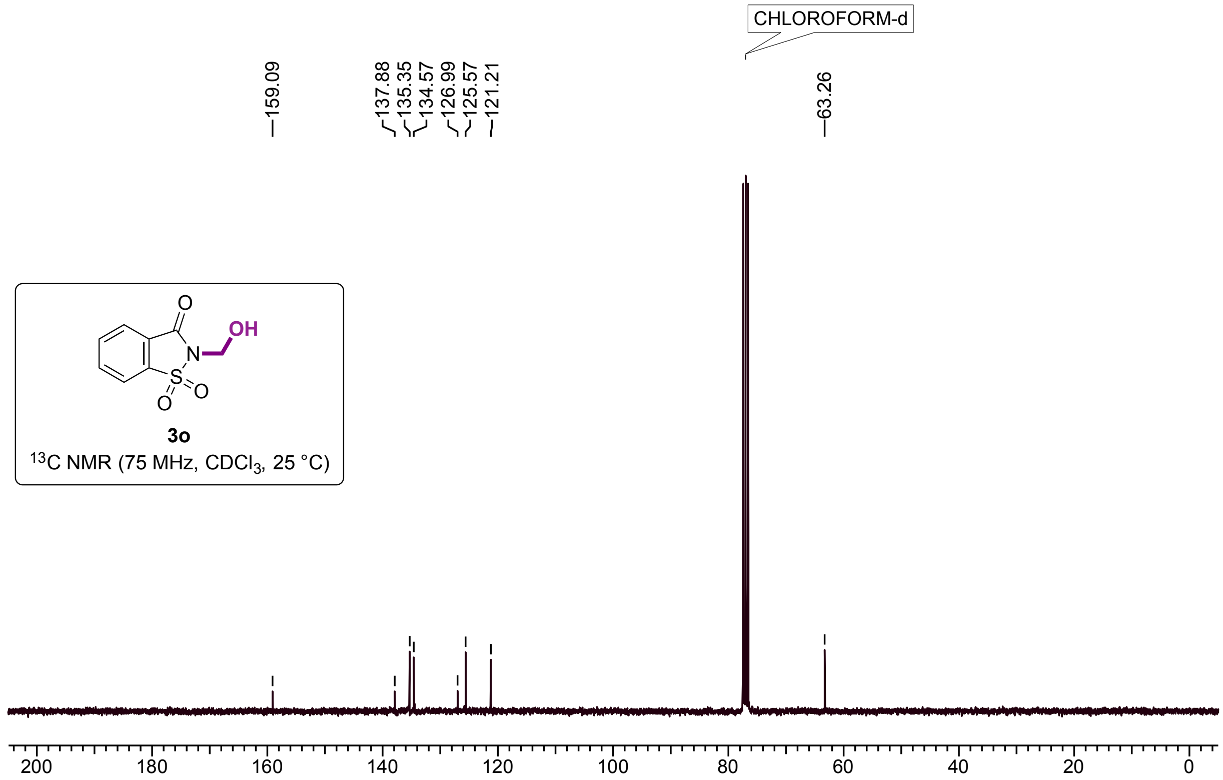
CHLOROFORM-d
159.09
137.88
135.35
134.57
126.99
125.57
121.21
63.26
3o
^{13}C NMR (75 MHz, $CDCl_3$, 25 °C)
200 180 160 140 120 100 80 60 40 20 0

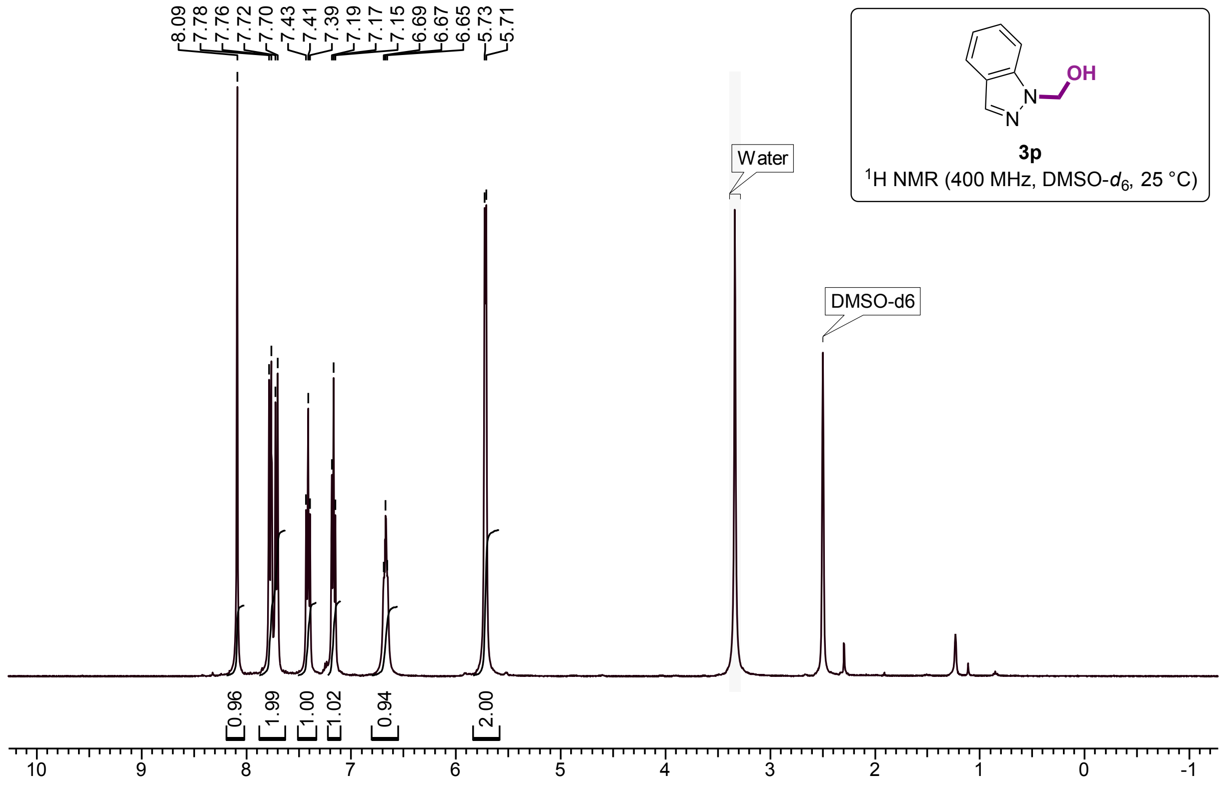
8.09
7.78
7.76
7.72
7.70
7.43
7.41
7.39
7.19
7.17
7.15
6.69
6.67
6.65
5.73
5.71
Water
DMSO-d6
3p
^{1}H NMR (400 MHz, DMSO-d_6, 25 °C)
0.96
1.99
1.00
1.02
0.94
2.00
10 9 8 7 6 5 4 3 2 1 0 -1

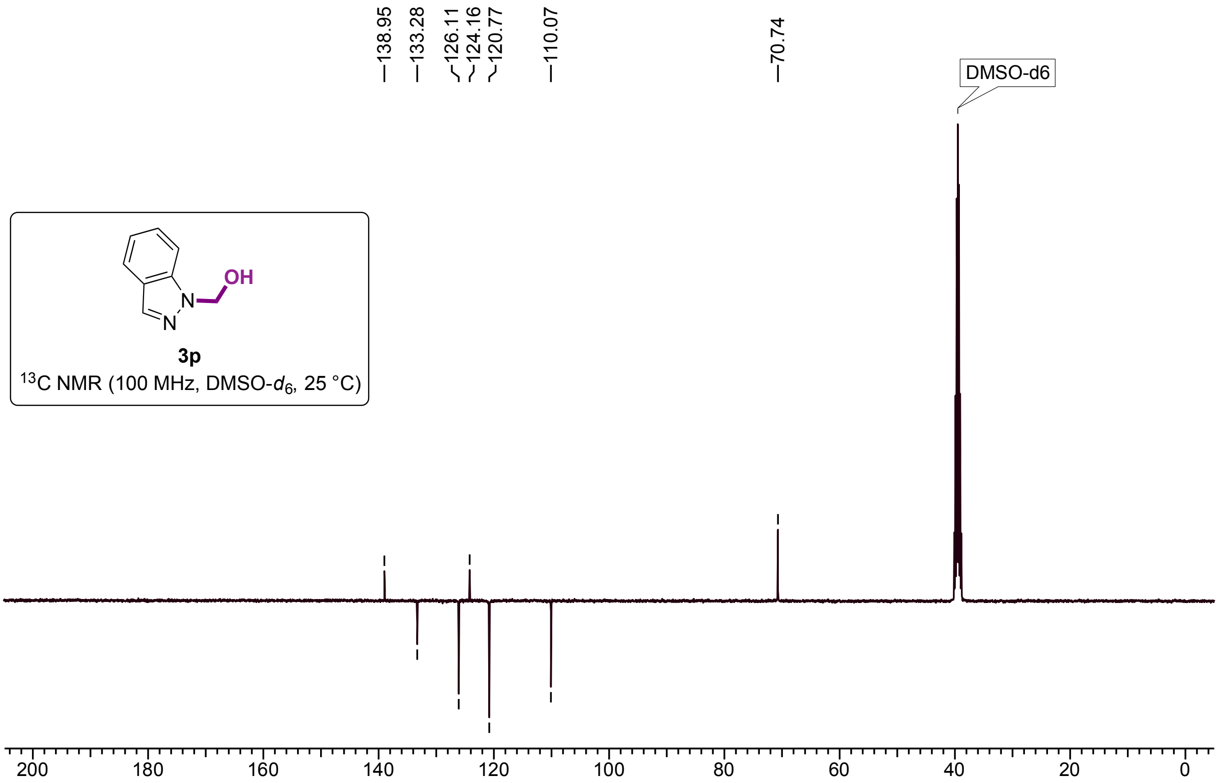
138.95
133.28
126.11
124.16
120.77
110.07
70.74
DMSO-d6
OH
N
N
3p
13C NMR (100 MHz, DMSO-d6, 25 °C)
200
180
160
140
120
100
80
60
40
20
0

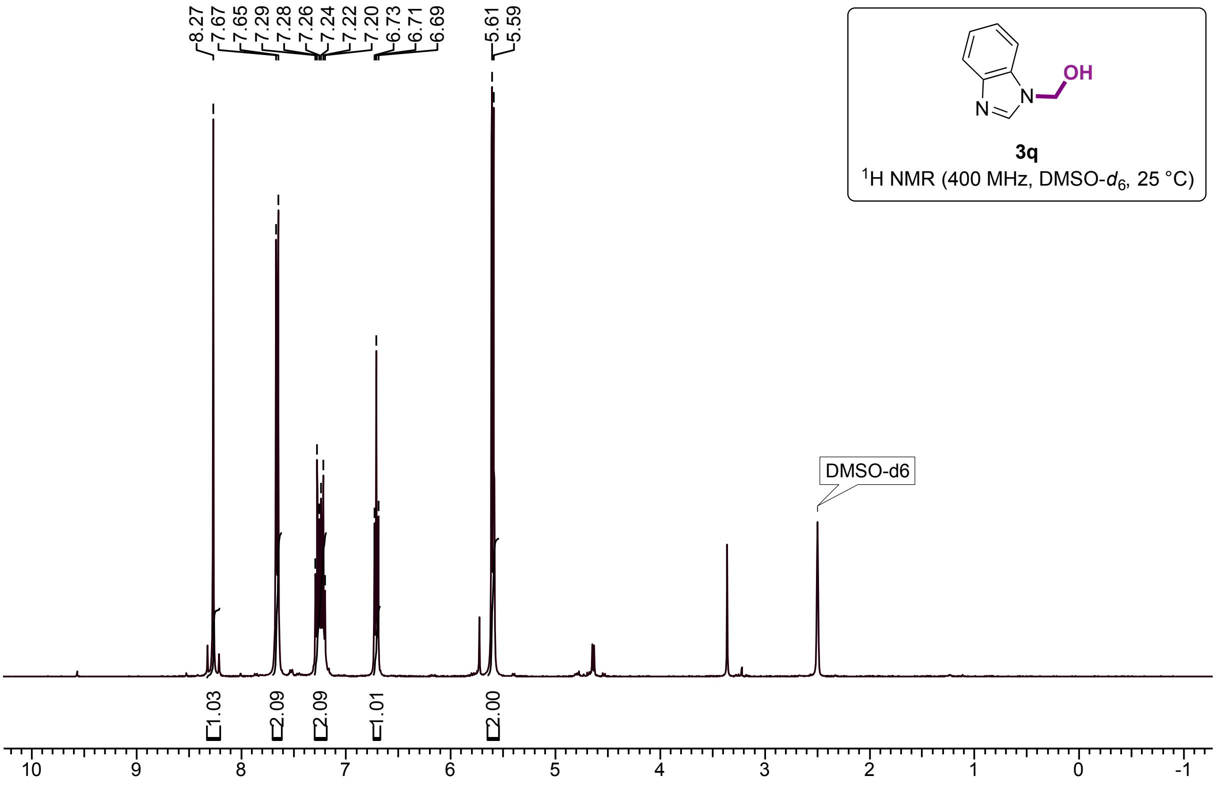
8.27
7.67
7.65
7.29
7.28
7.26
7.24
7.22
7.20
6.73
6.71
6.69
5.61
5.59
OH
N
N
3q
1H NMR (400 MHz, DMSO-d6, 25 °C)
DMSO-d6
1.03
2.09
2.09
1.01
2.00
10
9
8
7
6
5
4
3
2
1
0
-1

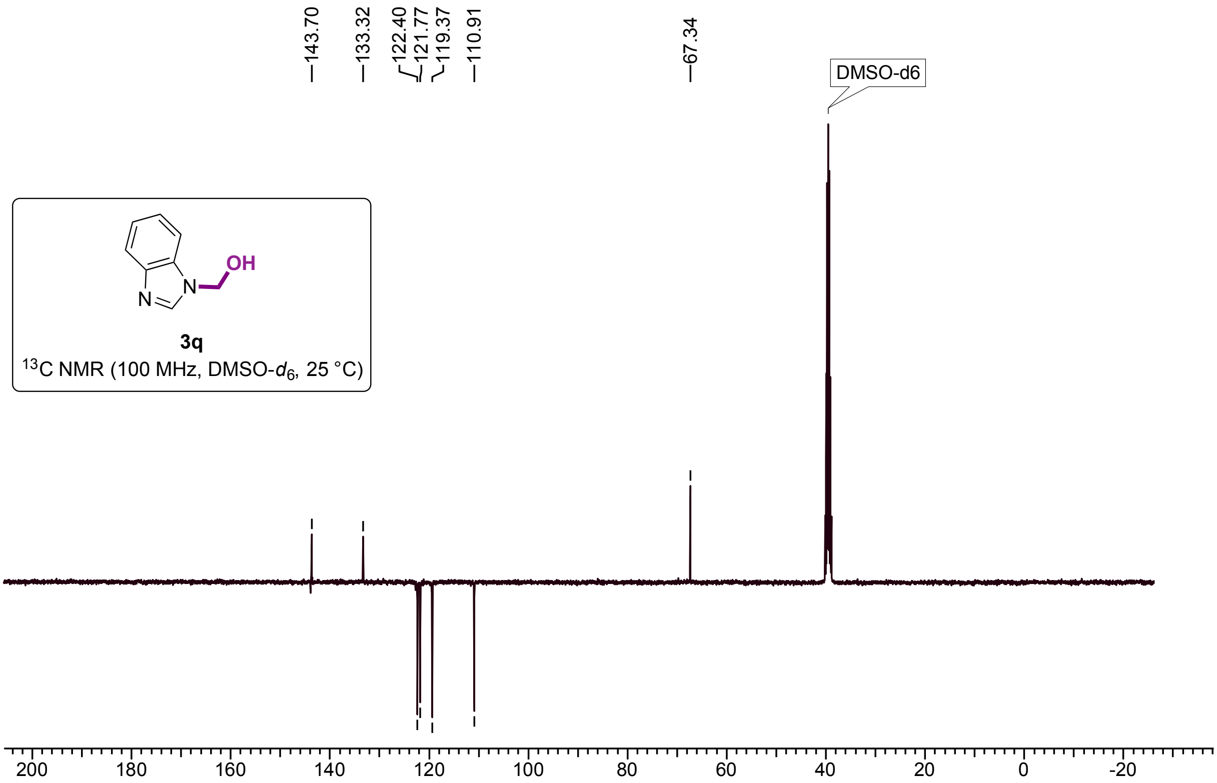
143.70
133.32
122.40
121.77
119.37
110.91
67.34
DMSO-d6
OH
N
N
3q
13C NMR (100 MHz, DMSO-d6, 25 °C)
200
180
160
140
120
100
80
60
40
20
0
-20

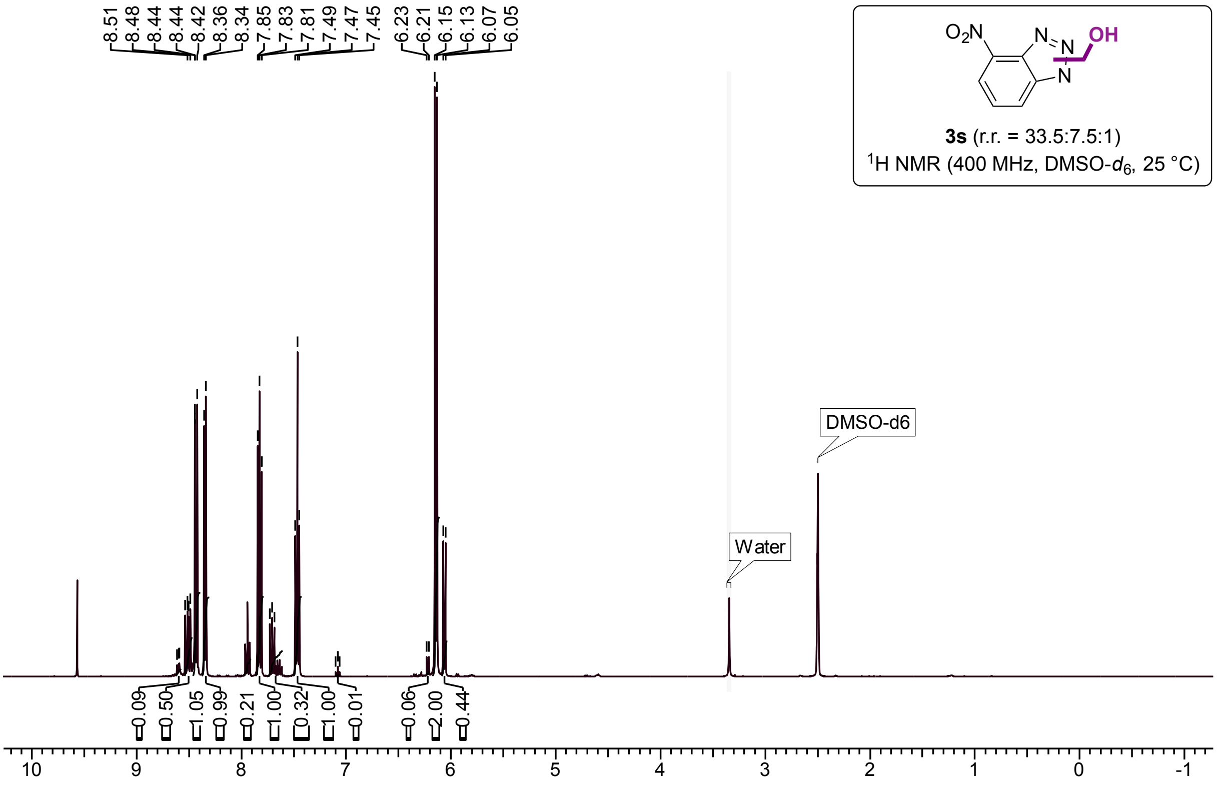
8.51
8.48
8.44
8.44
8.42
8.36
8.34
7.85
7.83
7.81
7.49
7.47
7.45
6.23
6.21
6.15
6.13
6.07
6.05
O2N
N
N
OH
N
3s (r.r. = 33.5:7.5:1)
1H NMR (400 MHz, DMSO-d6, 25 °C)
DMSO-d6
Water
0.09
0.50
1.05
0.99
0.21
1.00
0.32
1.00
0.01
0.06
2.00
0.44
10
9
8
7
6
5
4
3
2
1
0
-1

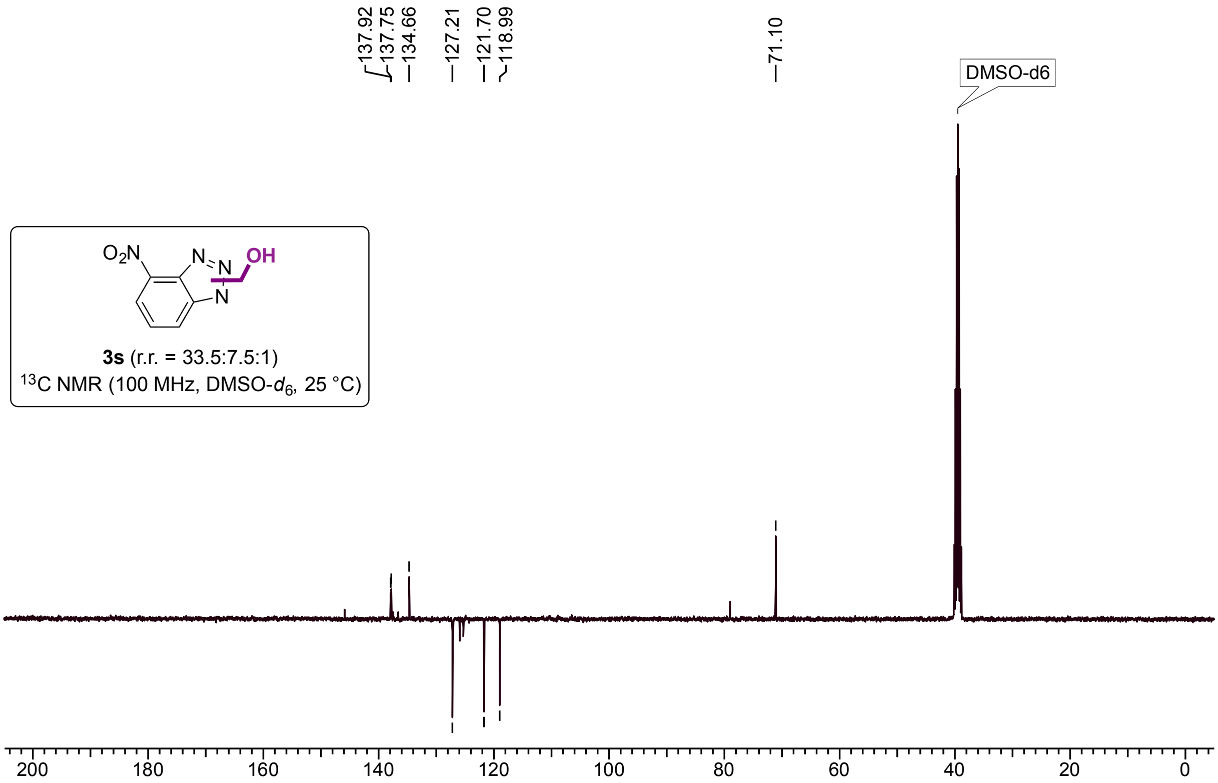

3s (r.r. = 33.5:7.5:1)
^{13}C NMR (100 MHz, DMSO-d_6, 25 °C)

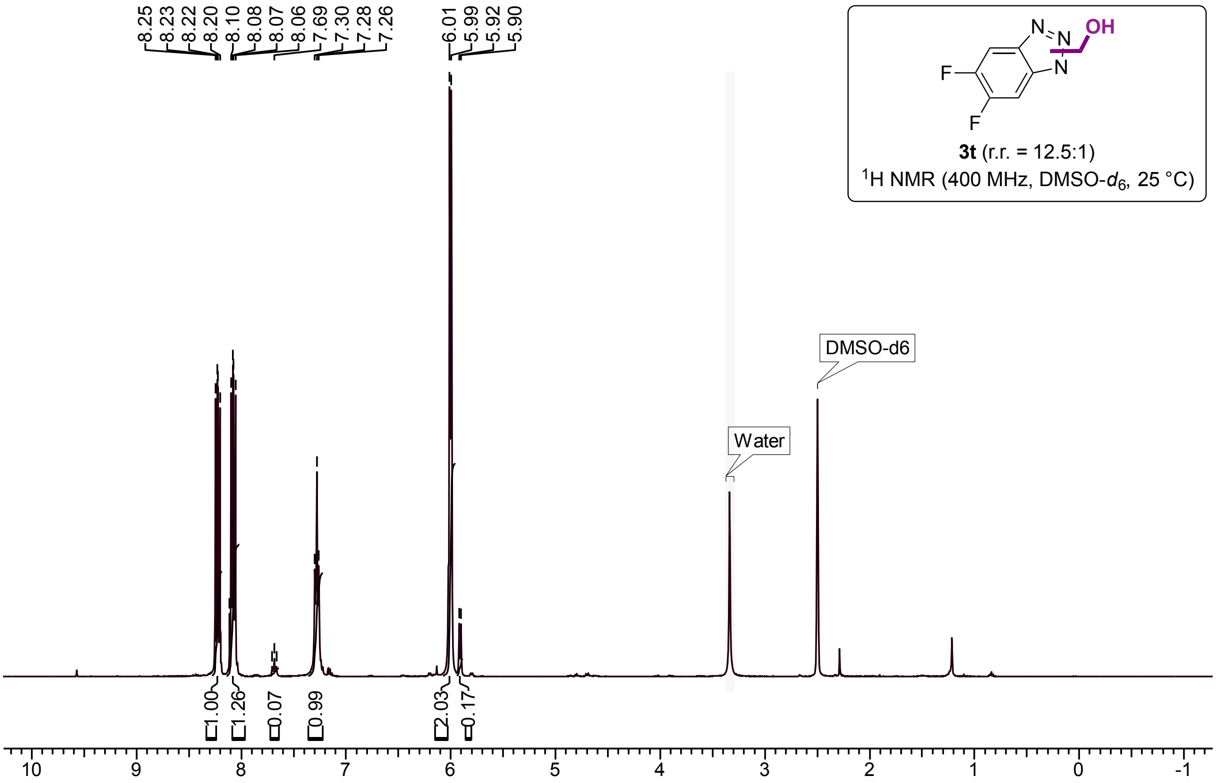

3t (r.r. = 12.5:1)
^{1}H NMR (400 MHz, DMSO-d_6, 25 °C)

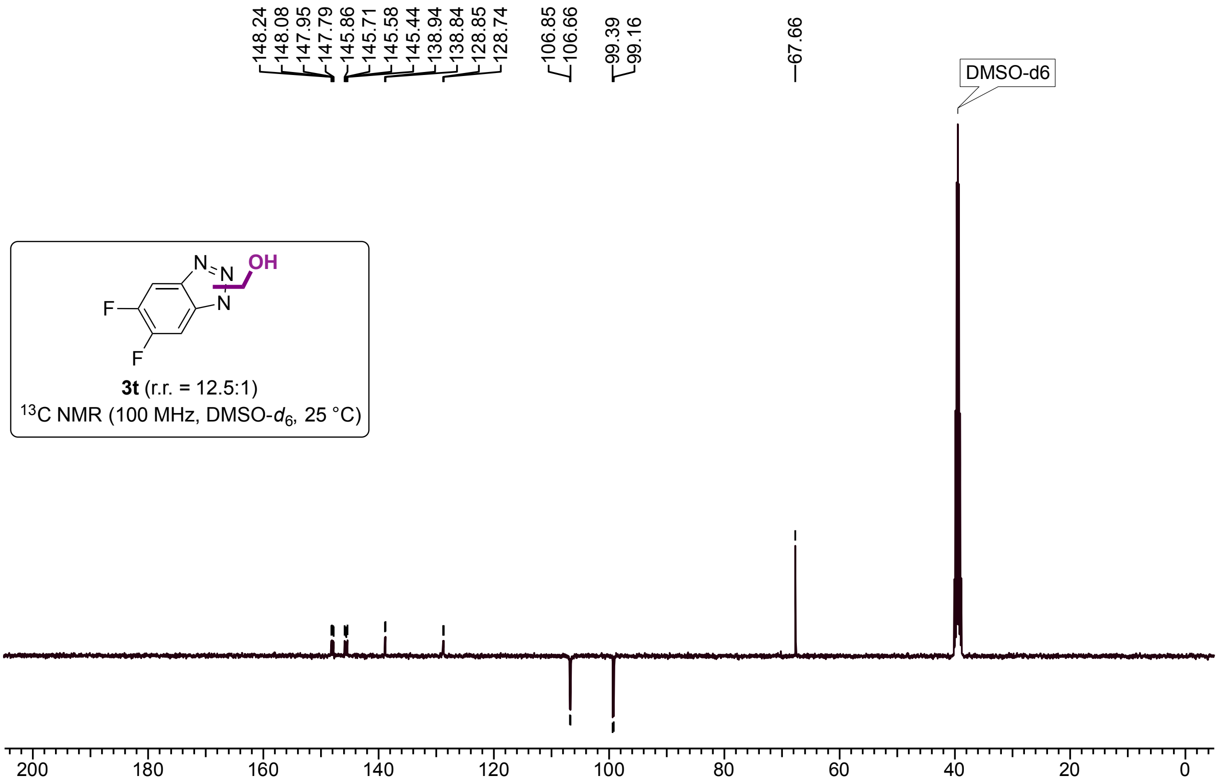
148.24
148.08
147.95
147.79
145.86
145.71
145.58
145.44
138.94
138.84
128.85
128.74
106.85
106.66
99.39
99.16
67.66
DMSO-d6
3t (r.r. = 12.5:1)
13C NMR (100 MHz, DMSO-d6, 25 °C)
200
180
160
140
120
100
80
60
40
20
0

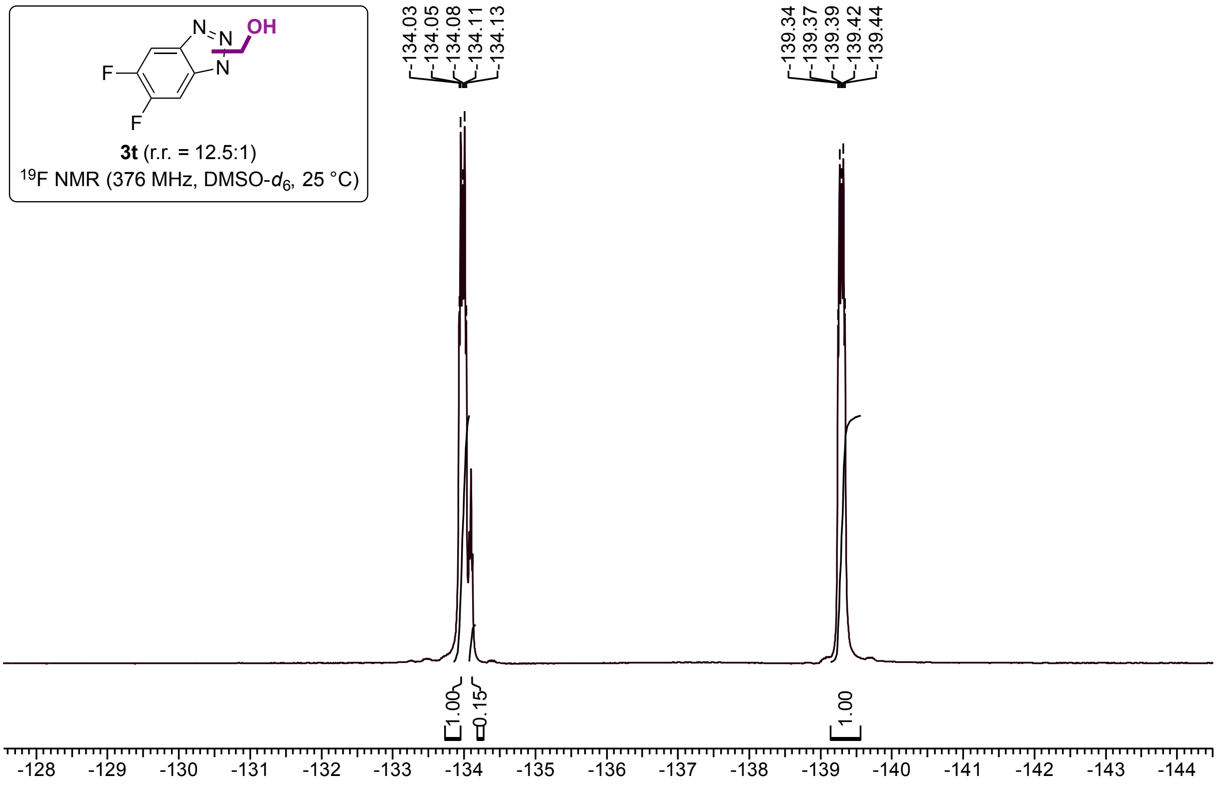
-134.03
-134.05
-134.08
-134.11
-134.13
-139.34
-139.37
-139.39
-139.42
-139.44
3t (r.r. = 12.5:1)
19F NMR (376 MHz, DMSO-d6, 25 °C)
1.00
0.15
1.00
-128
-129
-130
-131
-132
-133
-134
-135
-136
-137
-138
-139
-140
-141
-142
-143
-144

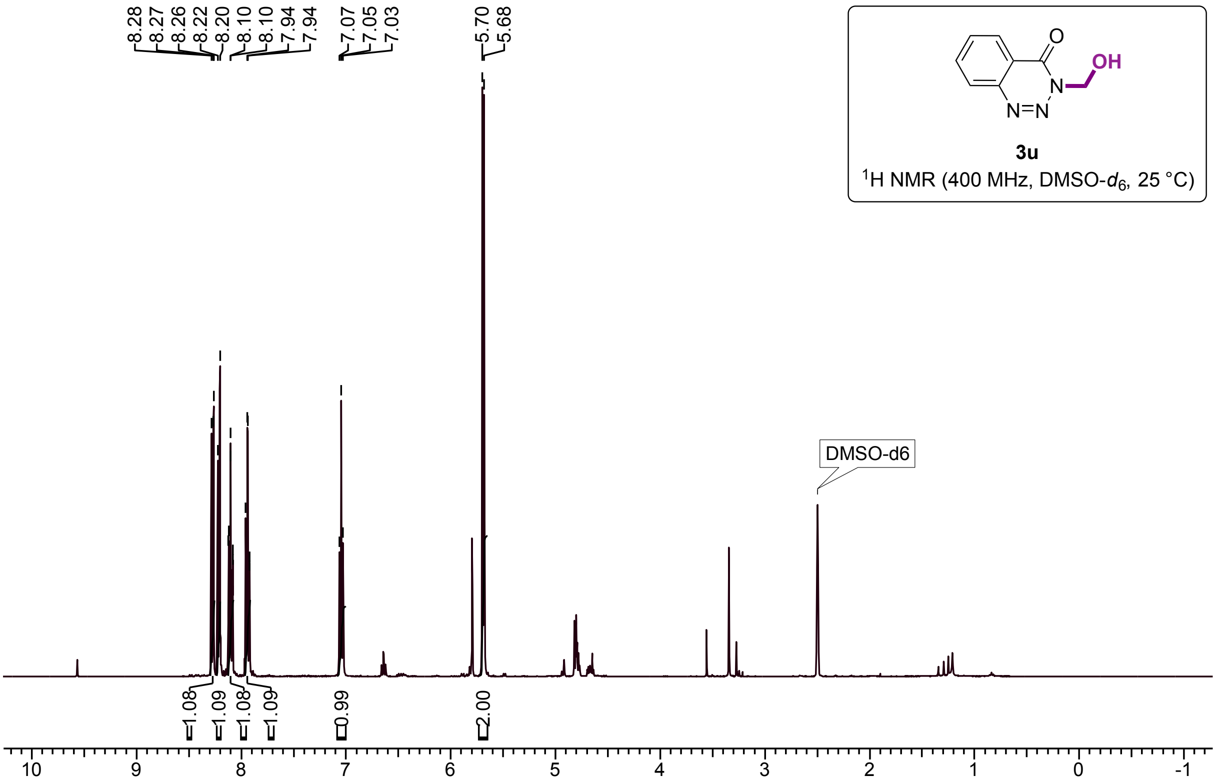
8.28
8.27
8.26
8.22
8.20
8.10
8.10
7.94
7.94
7.07
7.05
7.03
5.70
5.68
DMSO-d6
1.08
1.09
1.08
1.09
0.99
2.00
10 9 8 7 6 5 4 3 2 1 0 -1
OH
3u
1H NMR (400 MHz, DMSO-d6, 25 °C)

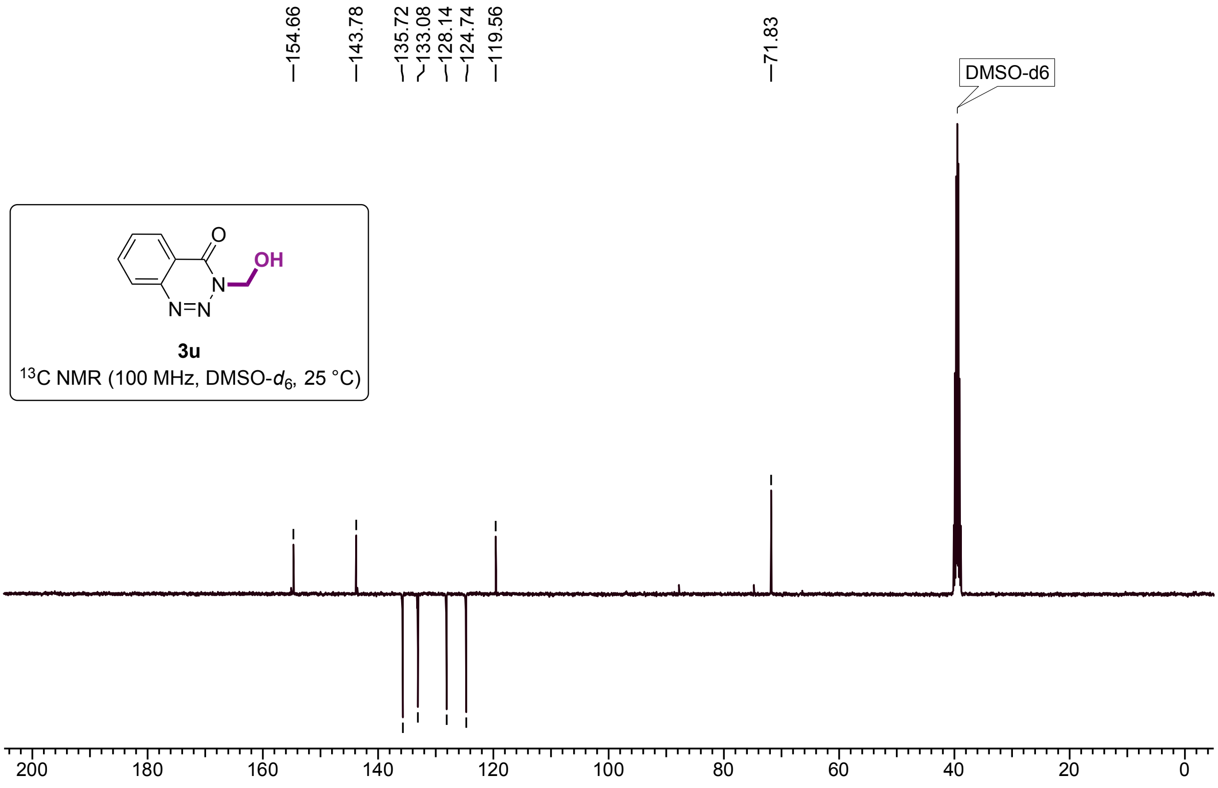
154.66
143.78
135.72
133.08
128.14
124.74
119.56
71.83
DMSO-d6
OH
3u
13C NMR (100 MHz, DMSO-d6, 25 °C)
200 180 160 140 120 100 80 60 40 20 0

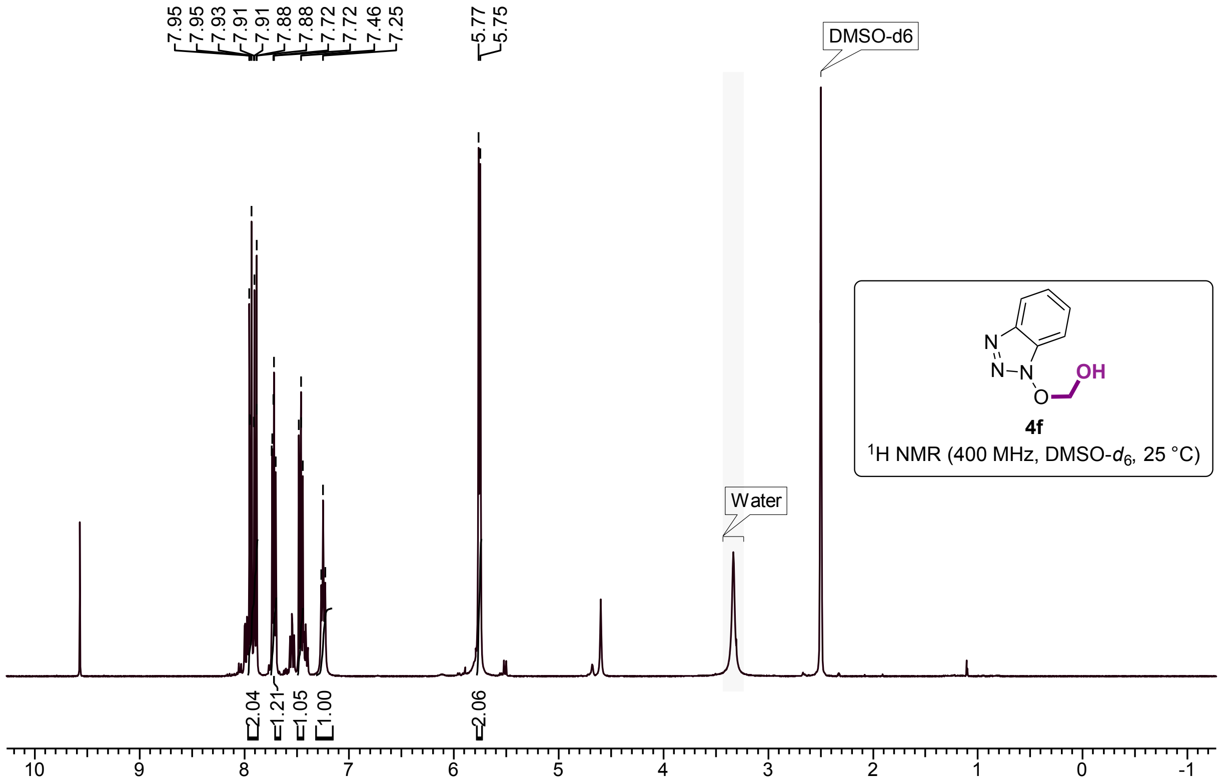
7.95
7.95
7.93
7.91
7.91
7.88
7.88
7.72
7.72
7.46
7.25
5.77
5.75
DMSO-d6
Water
OH
4f
1H NMR (400 MHz, DMSO-d6, 25 °C)
2.04
1.21
1.05
1.00
2.06
10
9
8
7
6
5
4
3
2
1
0
-1

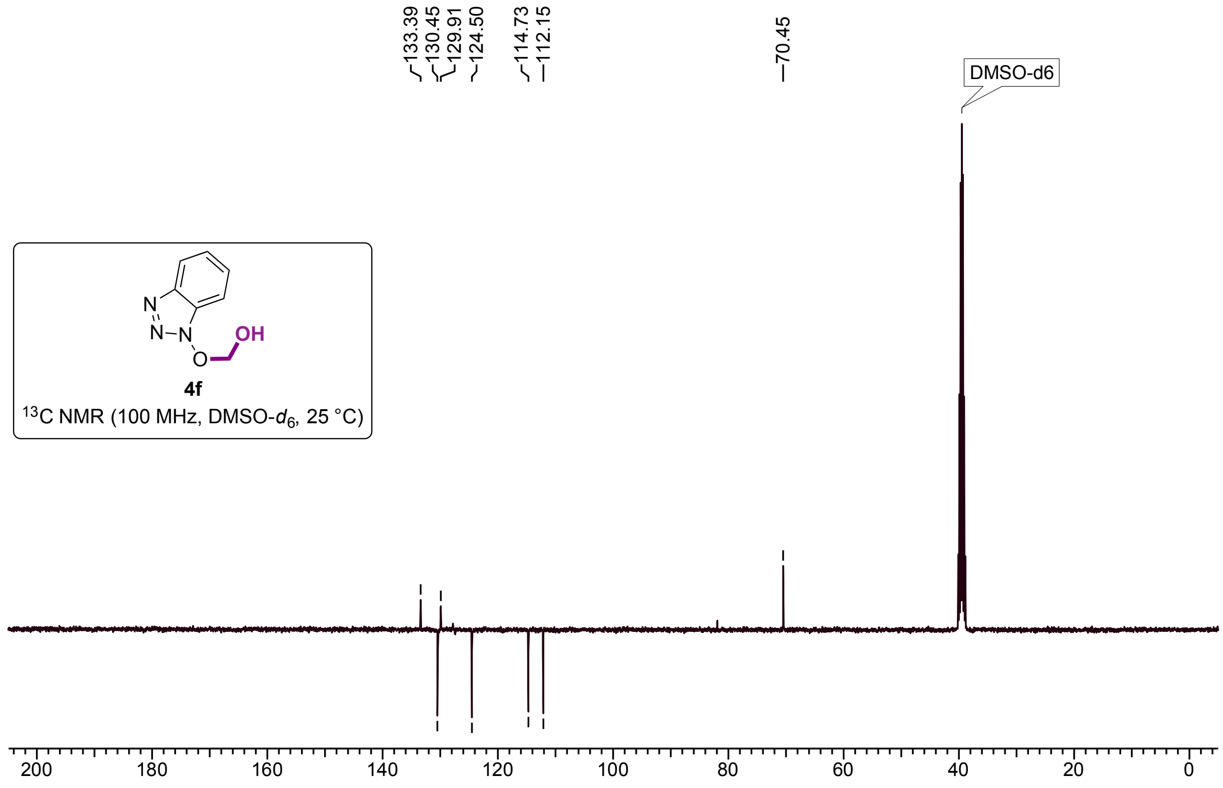
133.39
130.45
129.91
124.50
114.73
112.15
70.45
DMSO-d6
OH
4f
13C NMR (100 MHz, DMSO-d6, 25 °C)
200
180
160
140
120
100
80
60
40
20
0

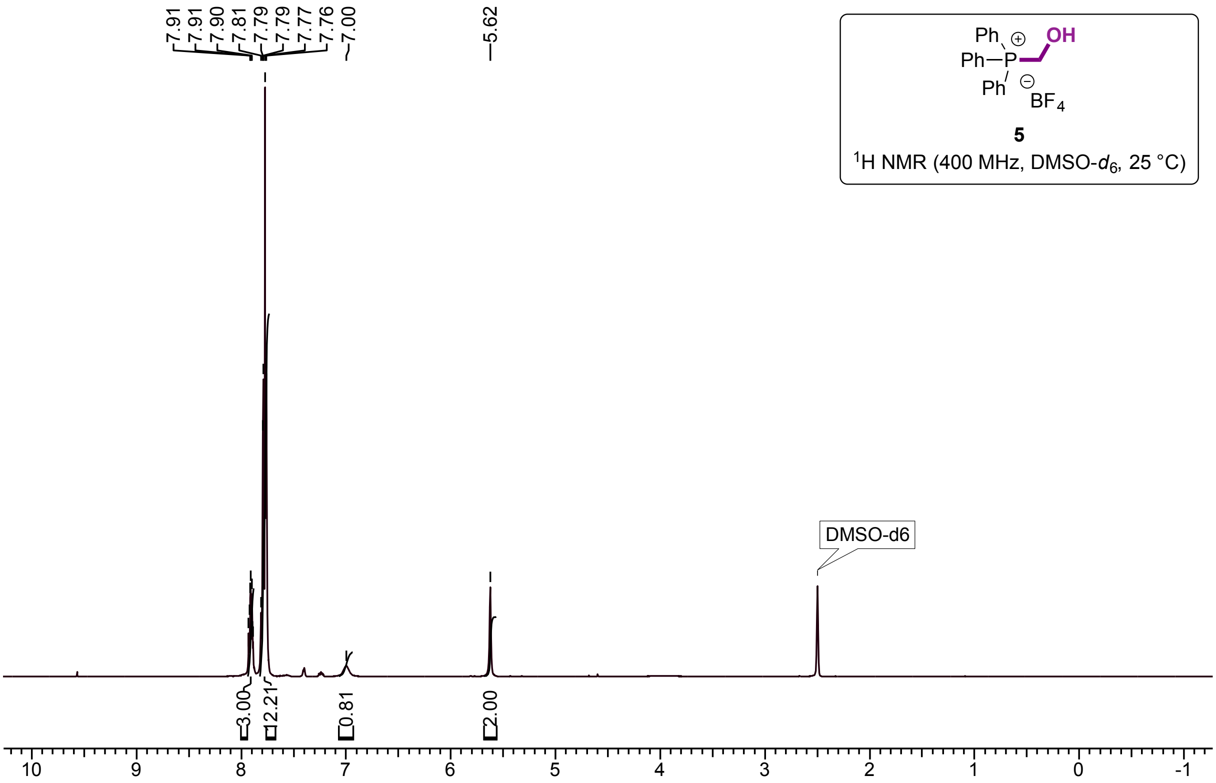
7.91
7.91
7.90
7.81
7.79
7.79
7.77
7.76
7.00
5.62
Ph
Ph—P
Ph
OH
BF4
5
1H NMR (400 MHz, DMSO-d6, 25 °C)
DMSO-d6
3.00
12.21
0.81
2.00
10
9
8
7
6
5
4
3
2
1
0
-1

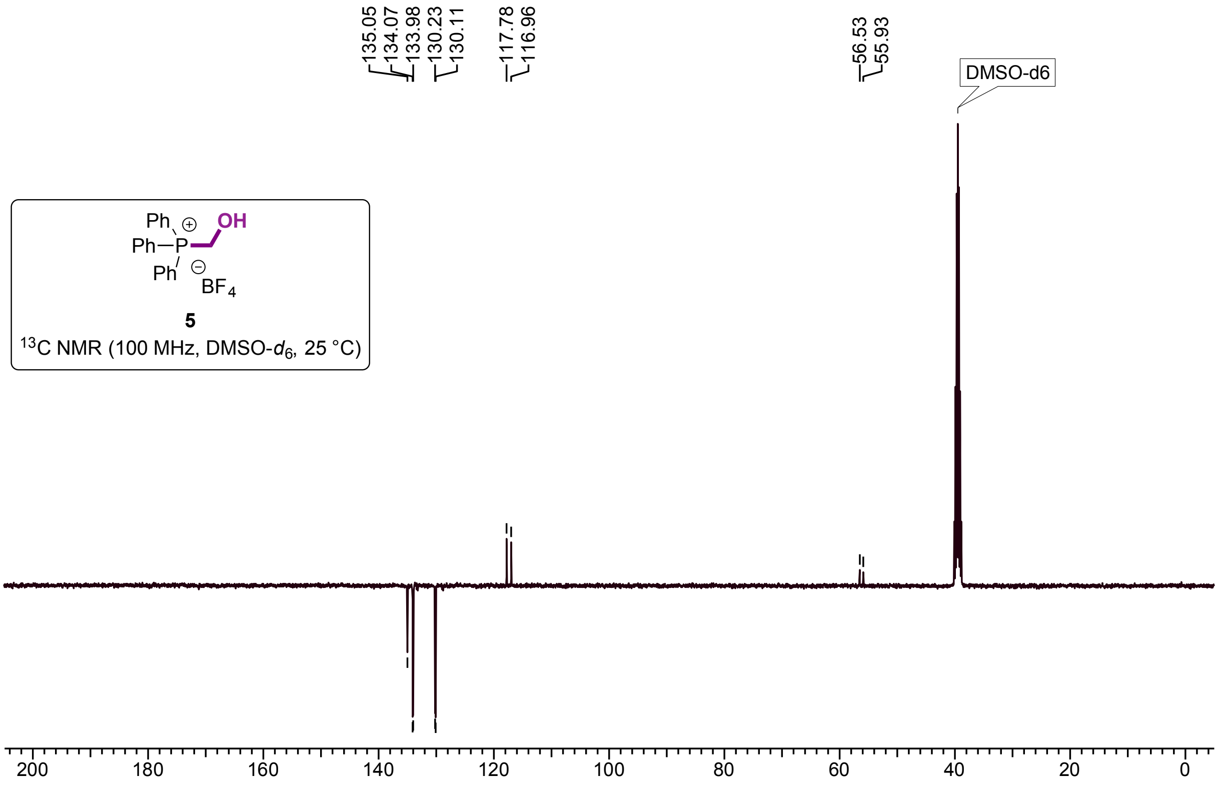
135.05
134.07
133.98
130.23
130.11
117.78
116.96
56.53
55.93
DMSO-d6
Ph
Ph—P
Ph
OH
BF4
5
13C NMR (100 MHz, DMSO-d6, 25 °C)
200
180
160
140
120
100
80
60
40
20
0

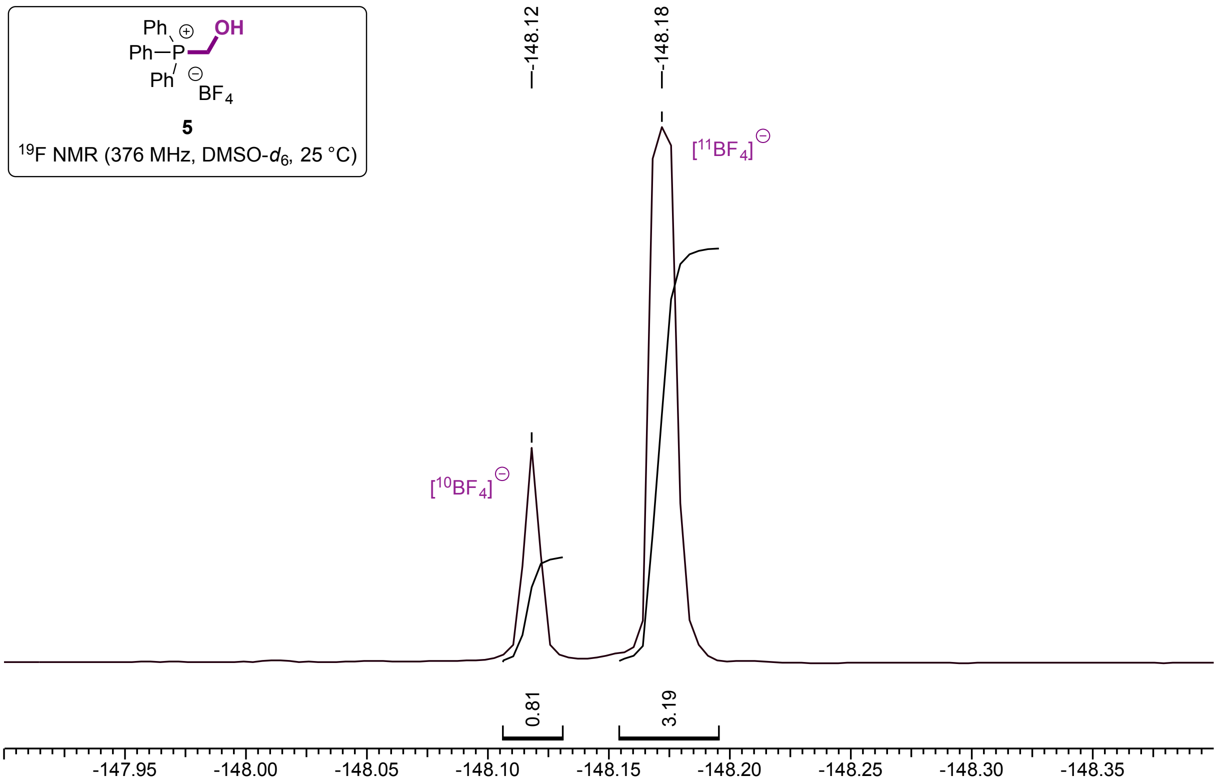
Ph
Ph—P
Ph
OH
BF 4
5
19F NMR (376 MHz, DMSO-d6, 25 °C)
-148.12
-148.18
[11BF4]
[10BF4]
0.81
3.19
-147.95
-148.00
-148.05
-148.10
-148.15
-148.20
-148.25
-148.30
-148.35

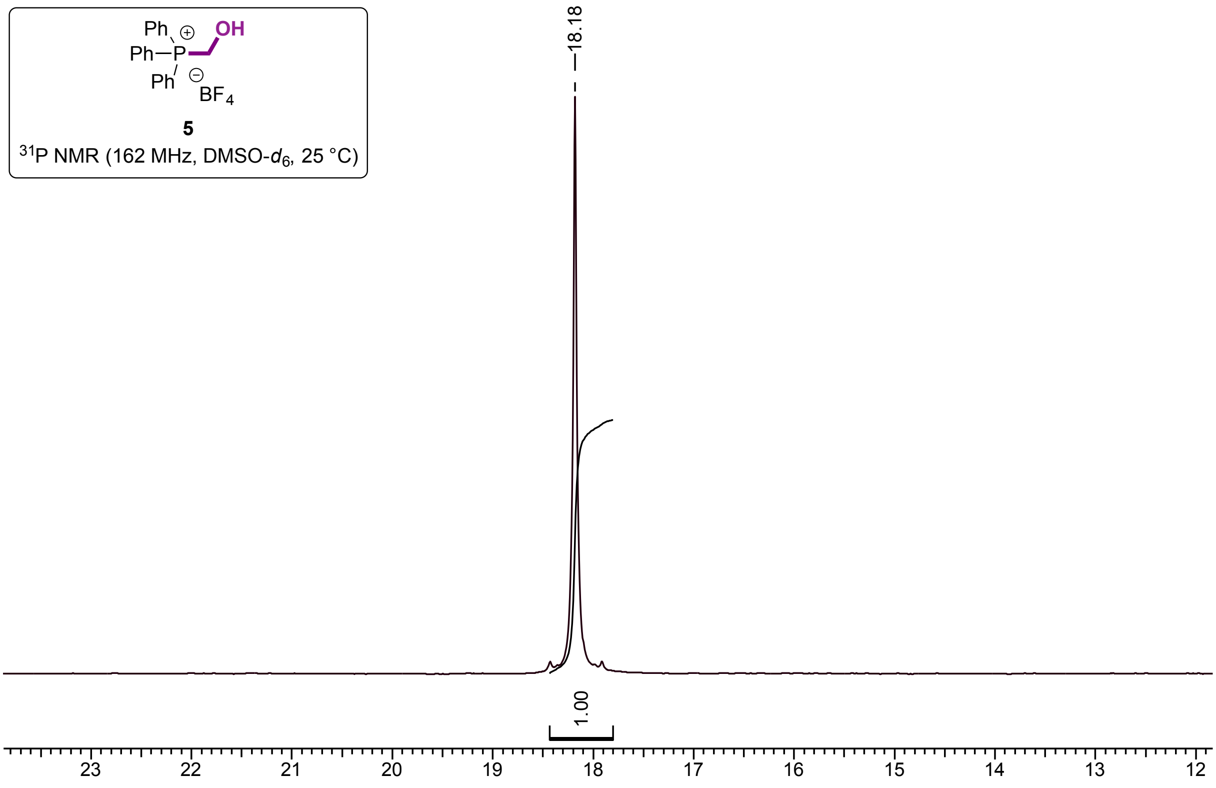
Ph
Ph—P
Ph
OH
BF 4
5
31P NMR (162 MHz, DMSO-d6, 25 °C)
18.18
1.00
23
22
21
20
19
18
17
16
15
14
13
12

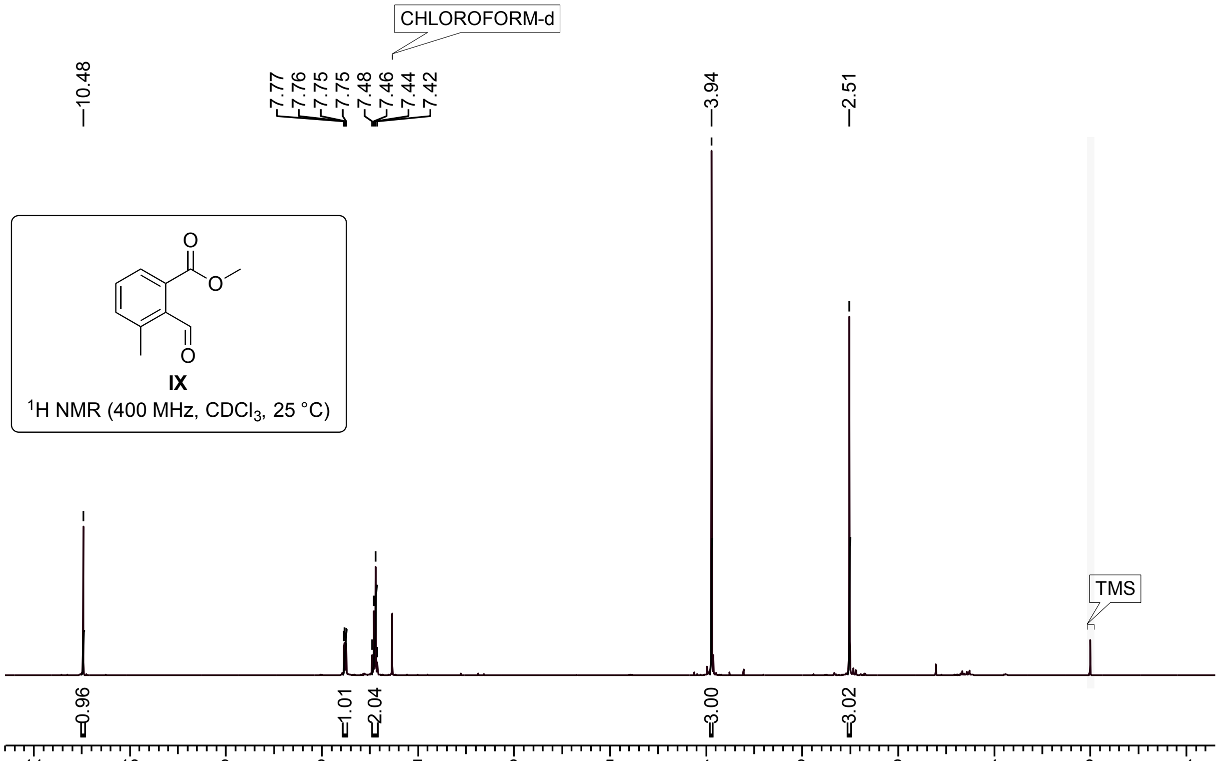

CHLOROFORM-d
10.48
7.77
7.76
7.75
7.75
7.48
7.46
7.44
7.42
3.94
2.51
O
O
O
IX
1H NMR (400 MHz, CDCl3, 25 °C)
TMS
0.96
1.01
2.04
3.00
3.02
11
10
9
8
7
6
5
4
3
2
1
0
-1

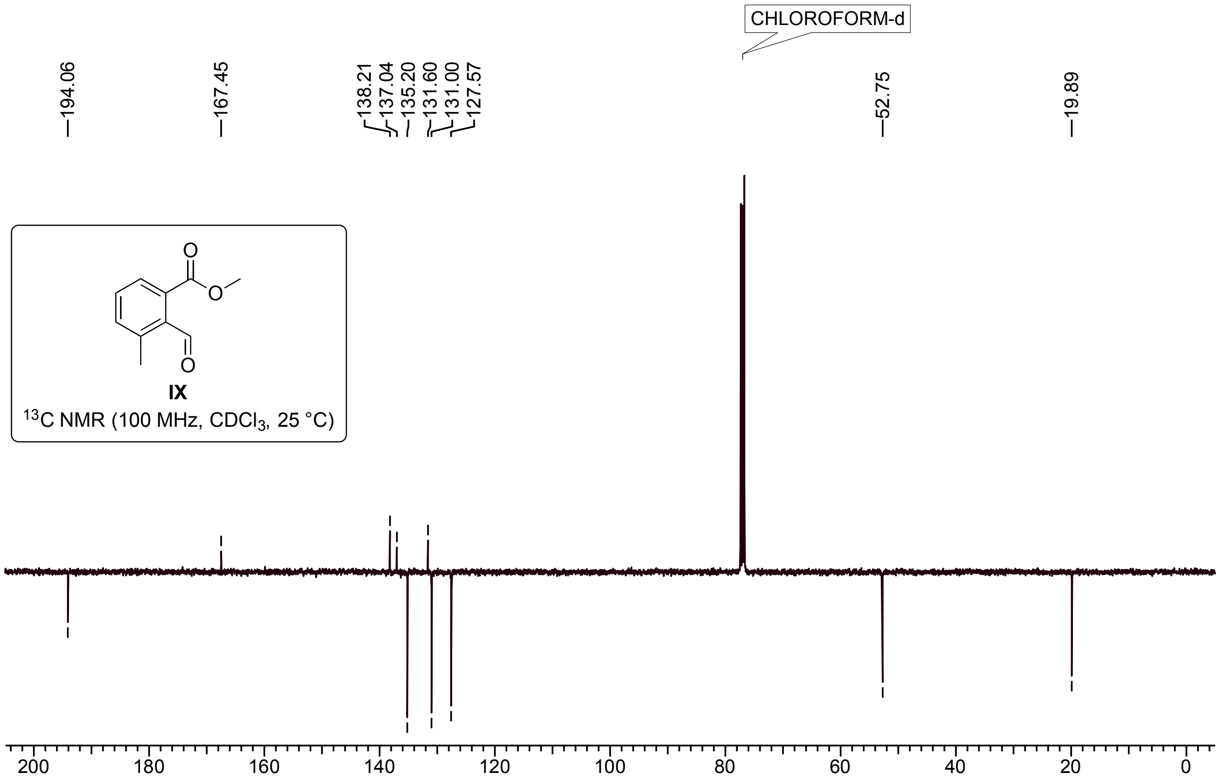

CHLOROFORM-d
194.06
167.45
138.21
137.04
135.20
131.60
131.00
127.57
52.75
19.89
O
O
O
IX
13C NMR (100 MHz, CDCl3, 25 °C)
200
180
160
140
120
100
80
60
40
20
0

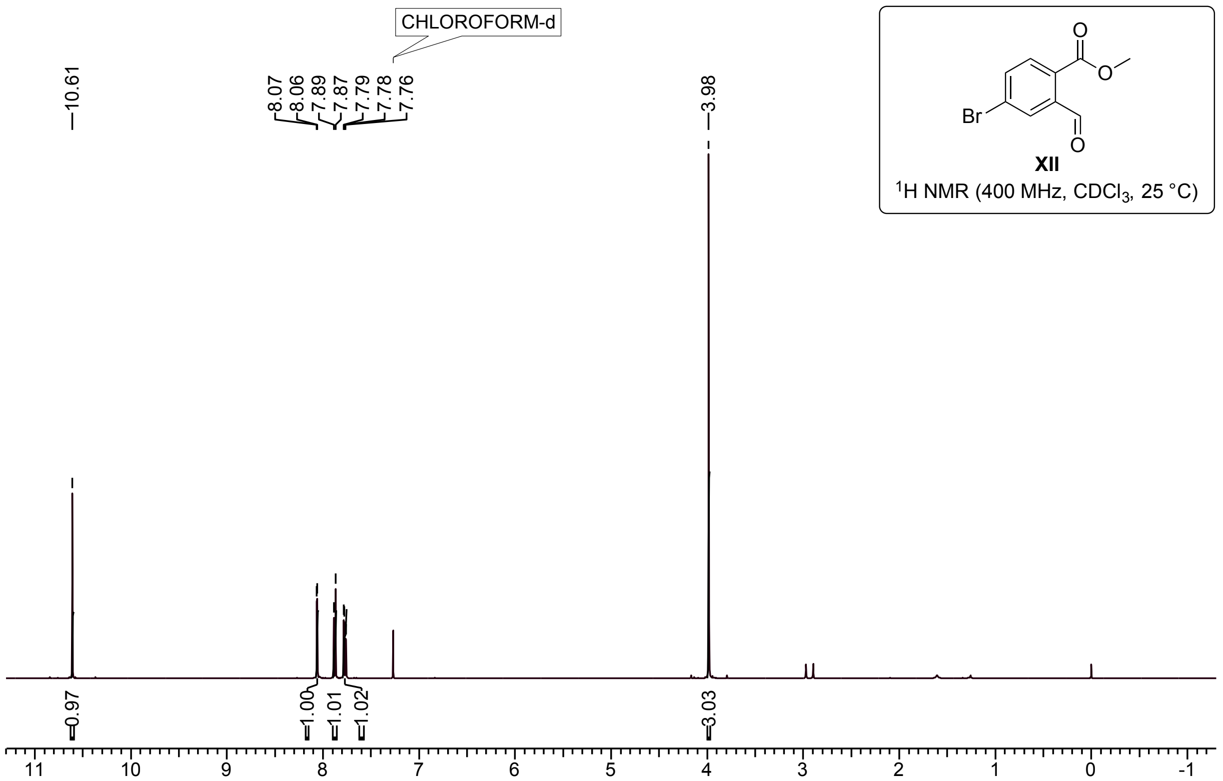

CHLOROFORM-d
10.61
8.07
8.06
7.89
7.87
7.79
7.78
7.76
3.98
Br
O
O
O
XII
1H NMR (400 MHz, CDCl3, 25 °C)
0.97
1.00
1.01
1.02
3.03
11
10
9
8
7
6
5
4
3
2
1
0
-1

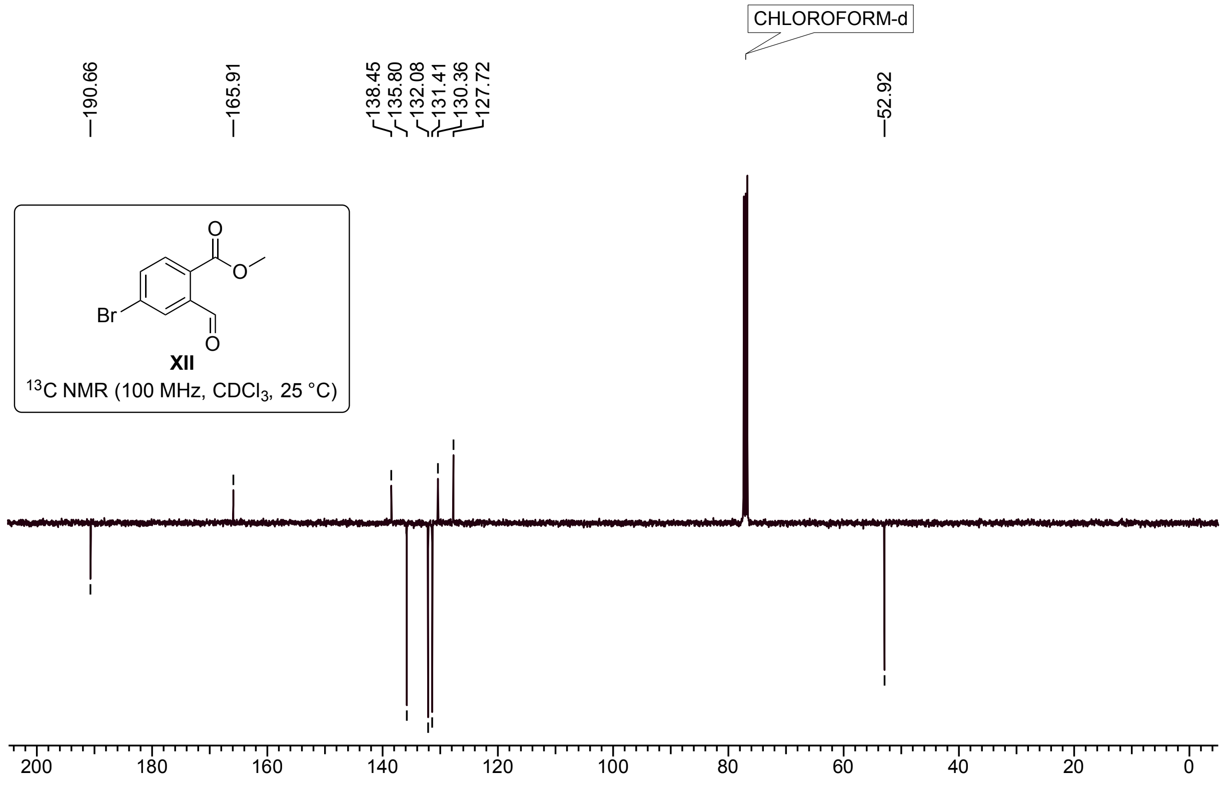

CHLOROFORM-d
190.66
165.91
138.45
135.80
132.08
131.41
130.36
127.72
52.92
Br
O
O
O
XII
13C NMR (100 MHz, CDCl3, 25 °C)
200
180
160
140
120
100
80
60
40
20
0

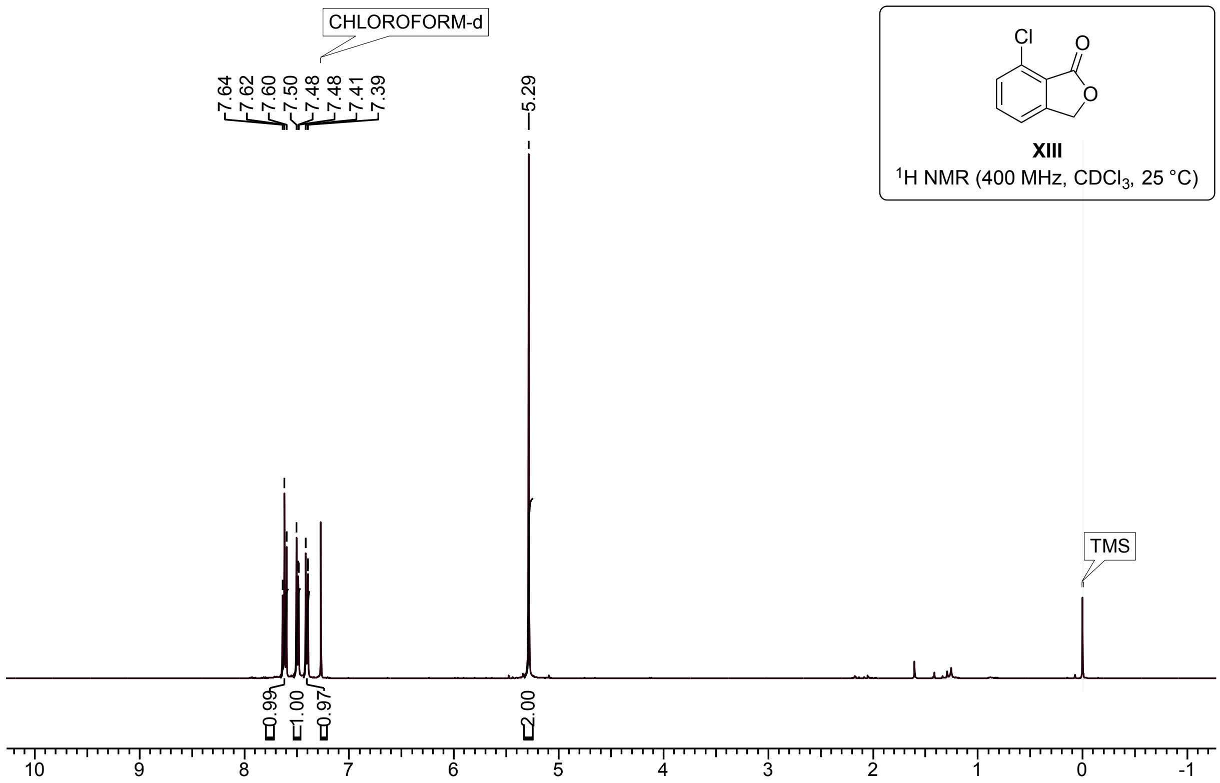
CHLOROFORM-d
7.64
7.62
7.60
7.50
7.48
7.48
7.41
7.39
5.29
Cl
O
O
XIII
1H NMR (400 MHz, CDCl3, 25 °C)
TMS
0.99
1.00
0.97
2.00
10
9
8
7
6
5
4
3
2
1
0
-1

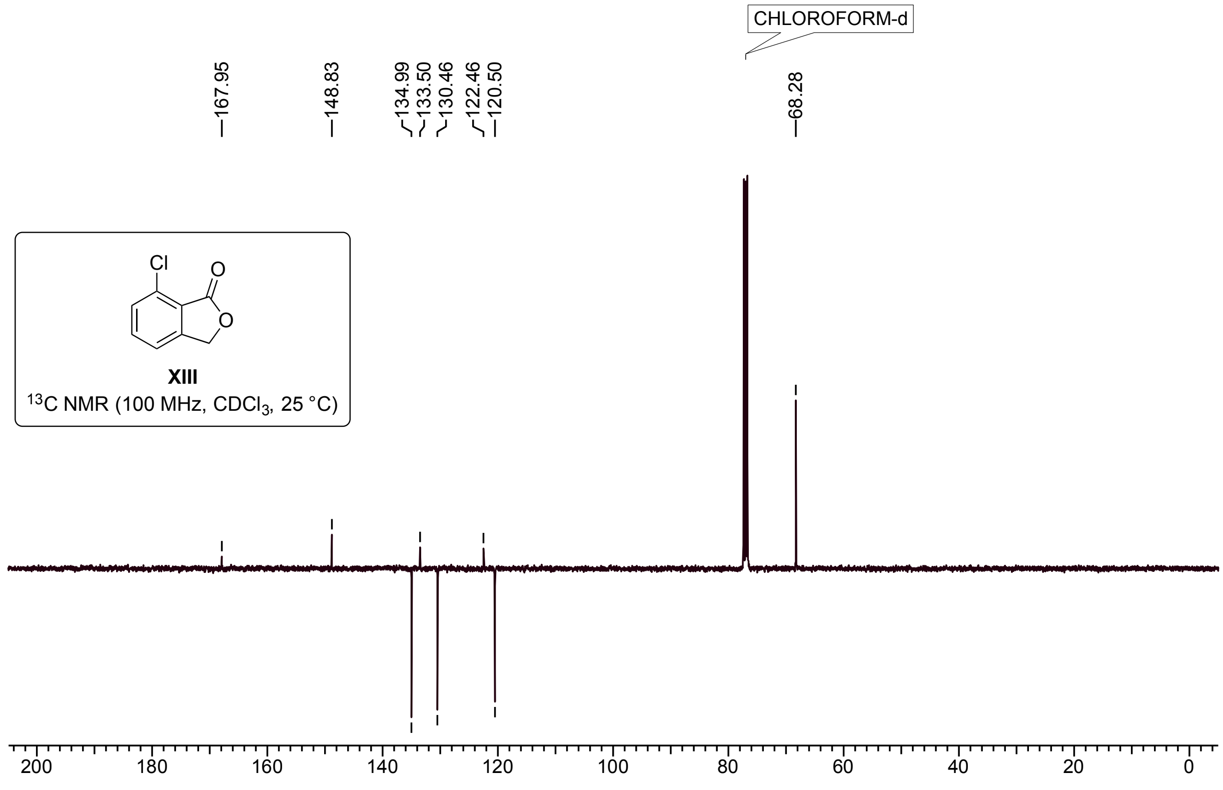
CHLOROFORM-d
167.95
148.83
134.99
133.50
130.46
122.46
120.50
68.28
Cl
O
O
XIII
13C NMR (100 MHz, CDCl3, 25 °C)
200
180
160
140
120
100
80
60
40
20
0

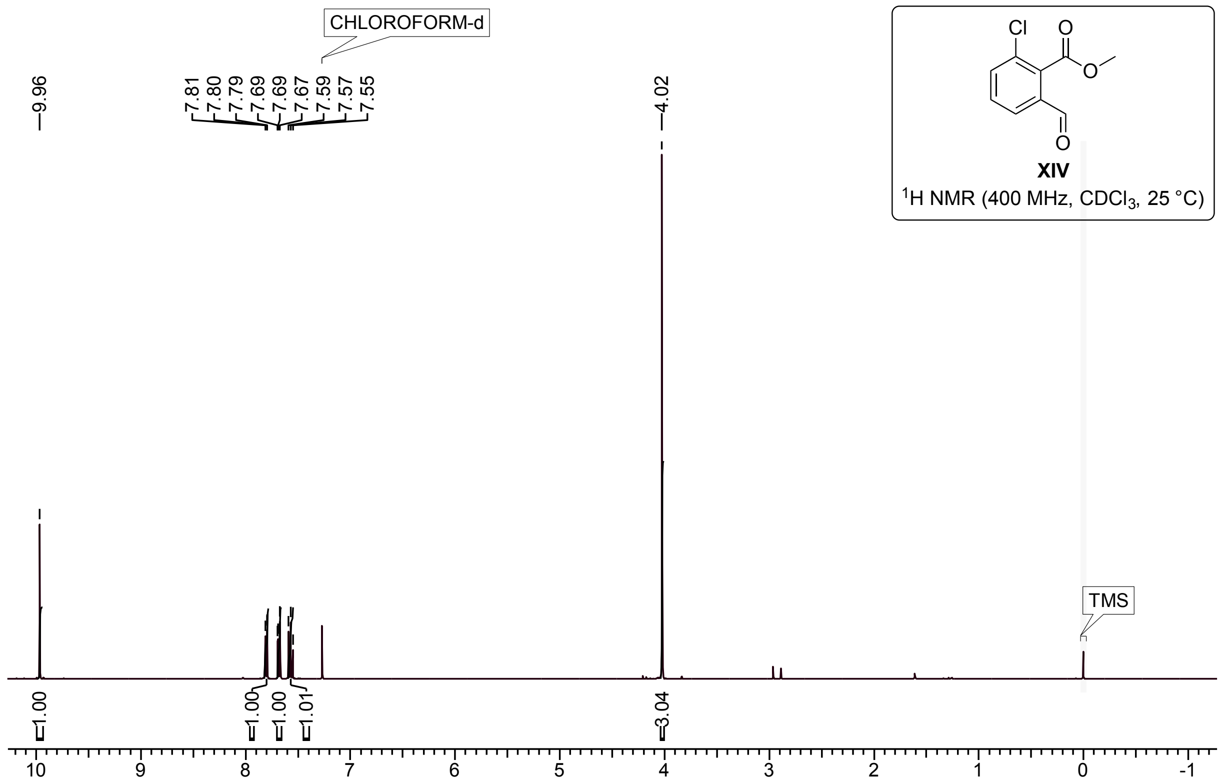

XIV

^1H NMR (400 MHz, $CDCl_3$, 25 °C)

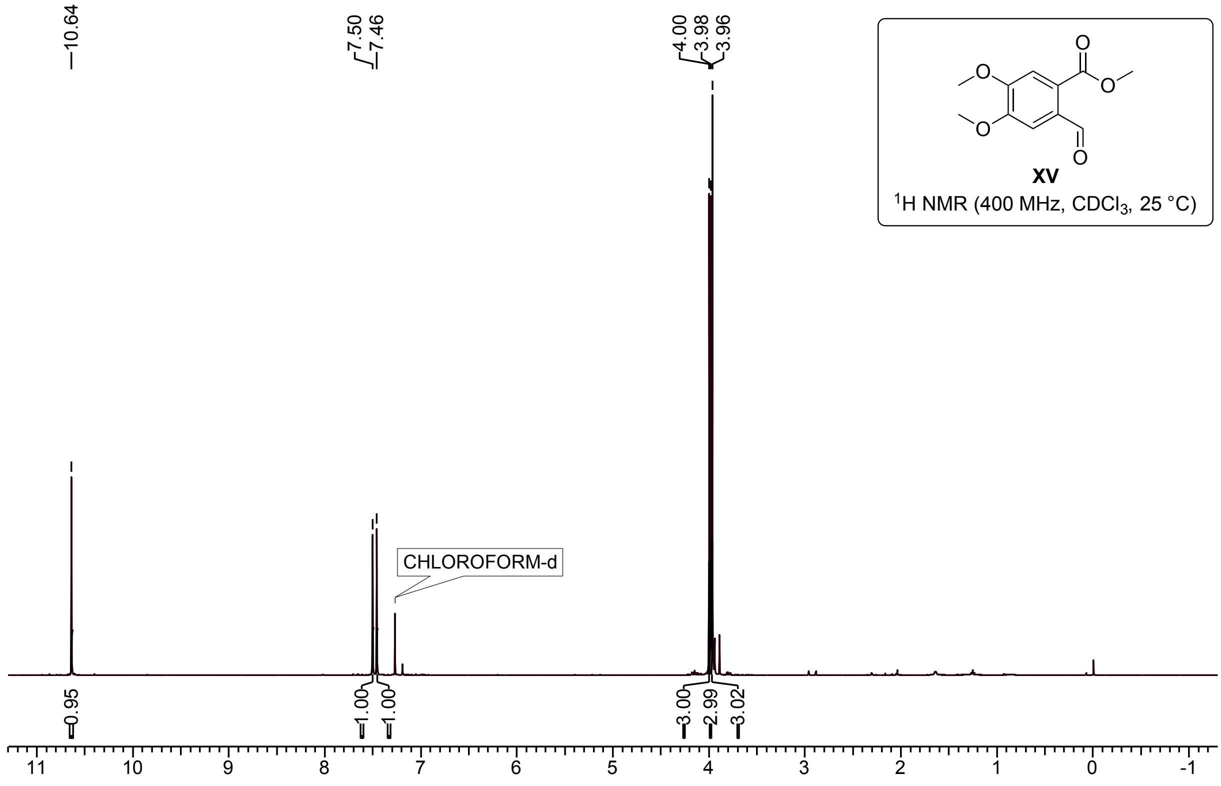

XV

^1H NMR (400 MHz, $CDCl_3$, 25 °C)

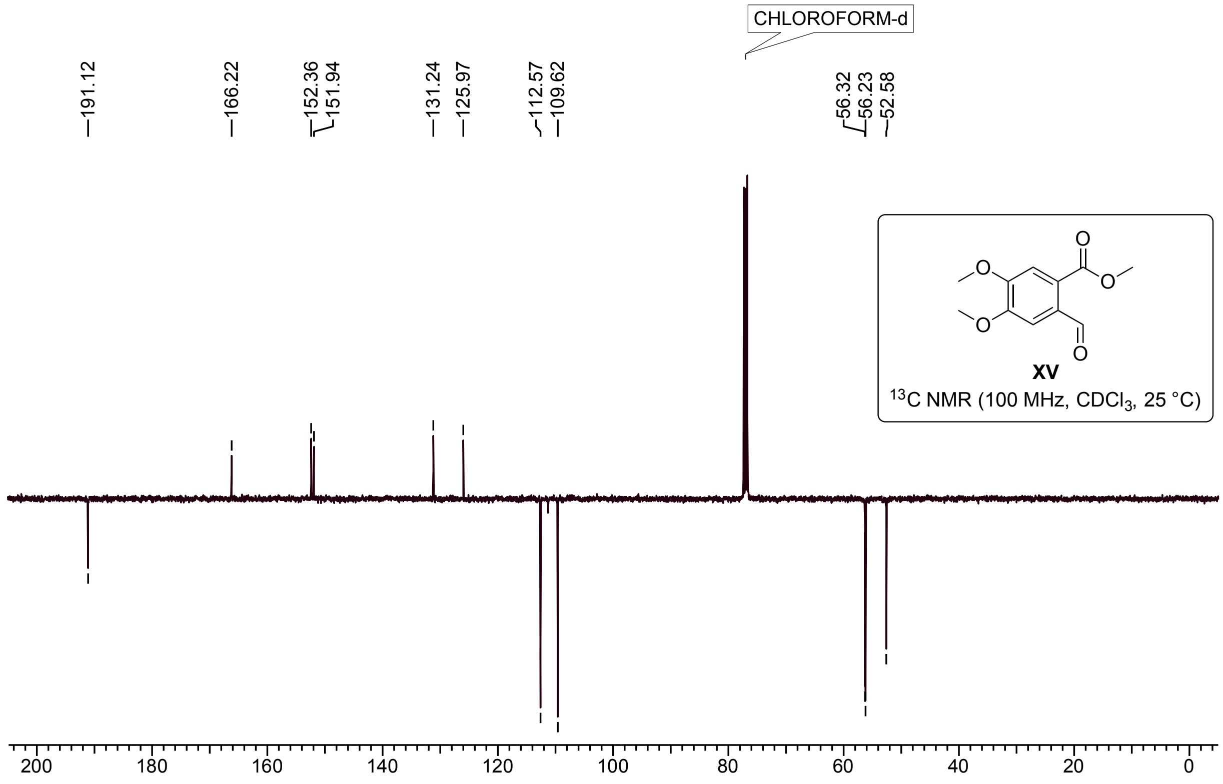
CHLOROFORM-d
191.12
166.22
152.36
151.94
131.24
125.97
112.57
109.62
56.32
56.23
52.58
XV
^{13}C NMR (100 MHz, CDCl$_3$, 25 °C)
200
180
160
140
120
100
80
60
40
20
0

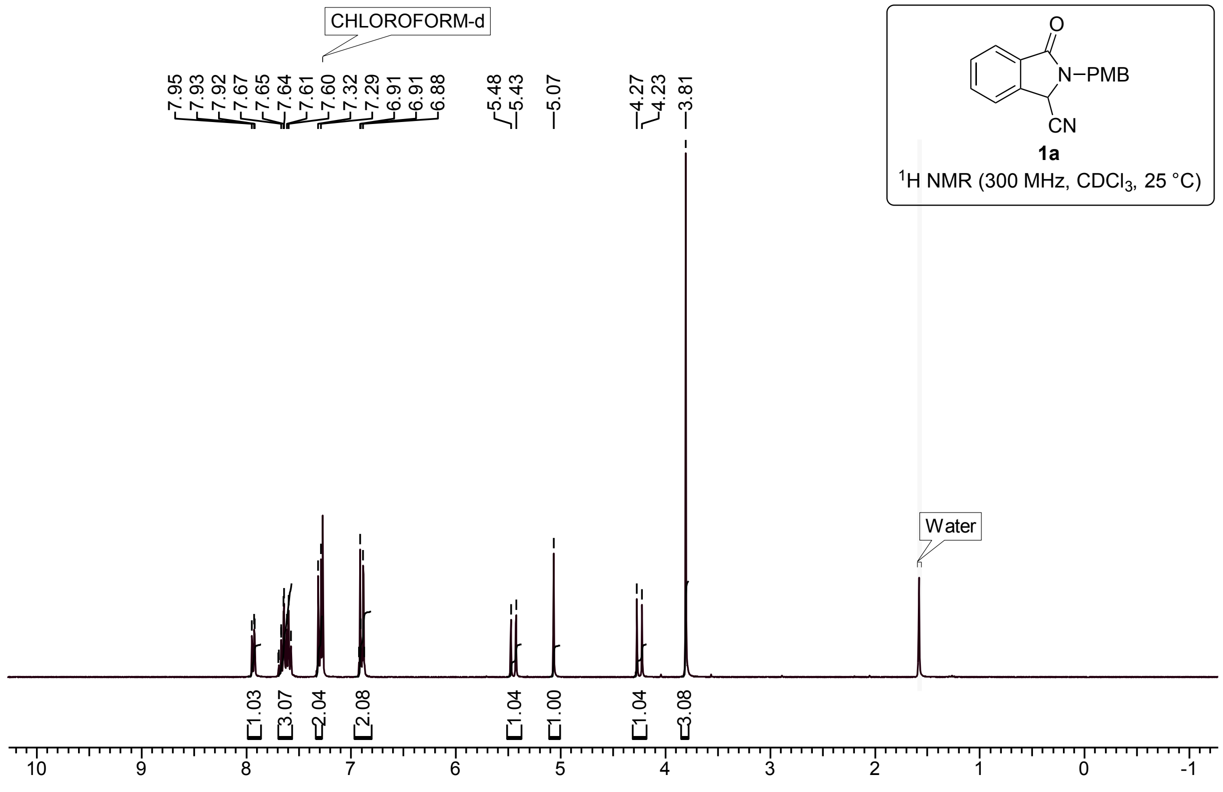
CHLOROFORM-d
7.95
7.93
7.92
7.67
7.65
7.64
7.61
7.60
7.32
7.29
6.91
6.88
5.48
5.43
5.07
4.27
4.23
3.81
N−PMB
CN
1a
^{1}H NMR (300 MHz, CDCl$_3$, 25 °C)
Water
1.03
3.07
2.04
2.08
1.04
1.00
1.04
3.08
10
9
8
7
6
5
4
3
2
1
0
-1

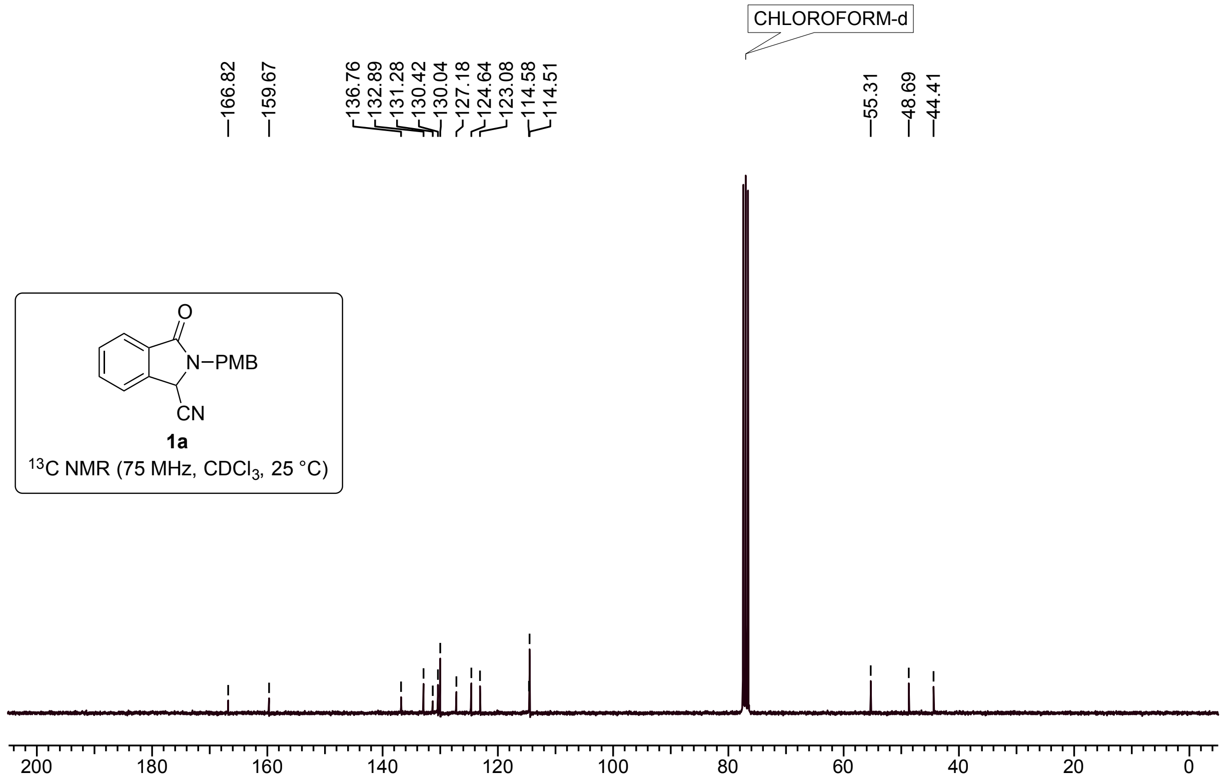
CHLOROFORM-d
166.82
159.67
136.76
132.89
131.28
130.42
130.04
127.18
124.64
123.08
114.58
114.51
55.31
48.69
44.41
N−PMB
O
CN
1a
^{13}C NMR (75 MHz, $CDCl_3$, 25 °C)
200
180
160
140
120
100
80
60
40
20
0

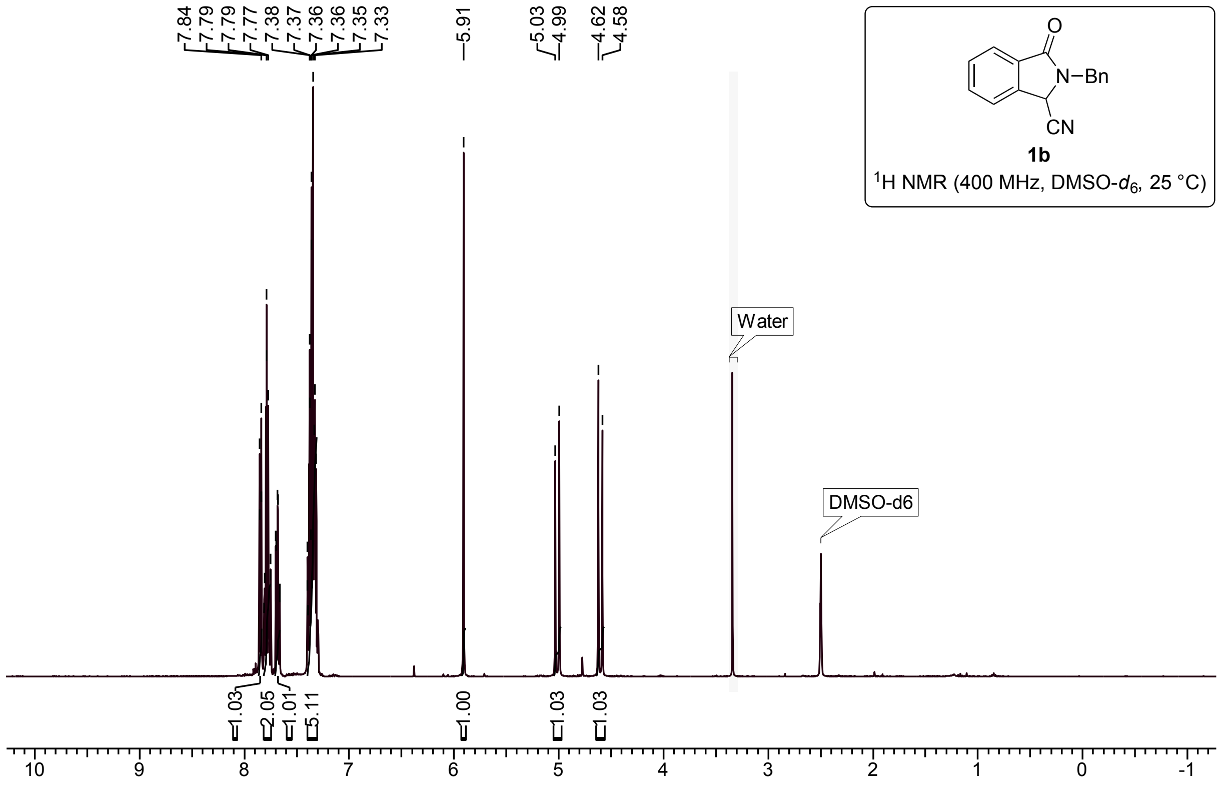
7.84
7.79
7.79
7.77
7.38
7.37
7.36
7.36
7.35
7.33
5.91
5.03
4.99
4.62
4.58
O
N−Bn
CN
1b
^{1}H NMR (400 MHz, DMSO-d_6, 25 °C)
Water
DMSO-d6
1.03
2.05
1.01
5.11
1.00
1.03
1.03
10
9
8
7
6
5
4
3
2
1
0
-1

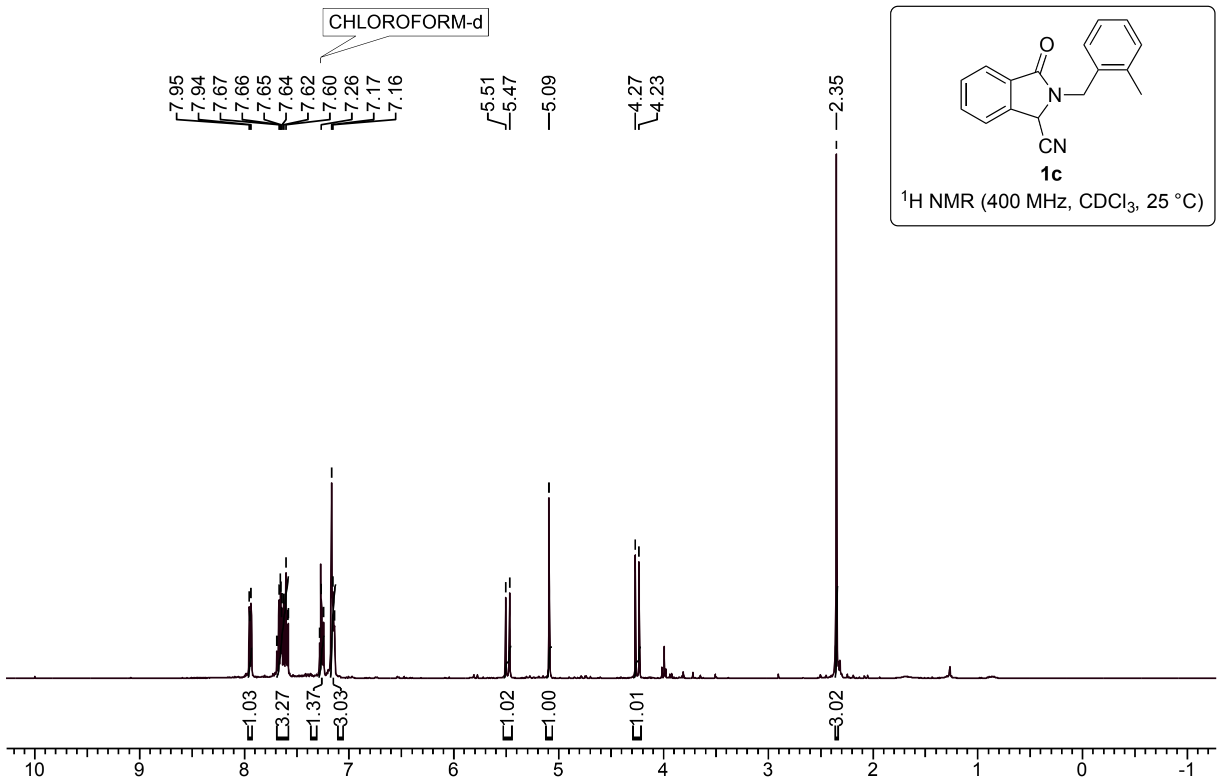
CHLOROFORM-d
7.95
7.94
7.67
7.66
7.65
7.64
7.62
7.60
7.26
7.17
7.16
5.51
5.47
5.09
4.27
4.23
2.35
O
N
CN
1c
1H NMR (400 MHz, CDCl3, 25 °C)
1.03
3.27
1.37
3.03
1.02
1.00
1.01
3.02
10
9
8
7
6
5
4
3
2
1
0
-1

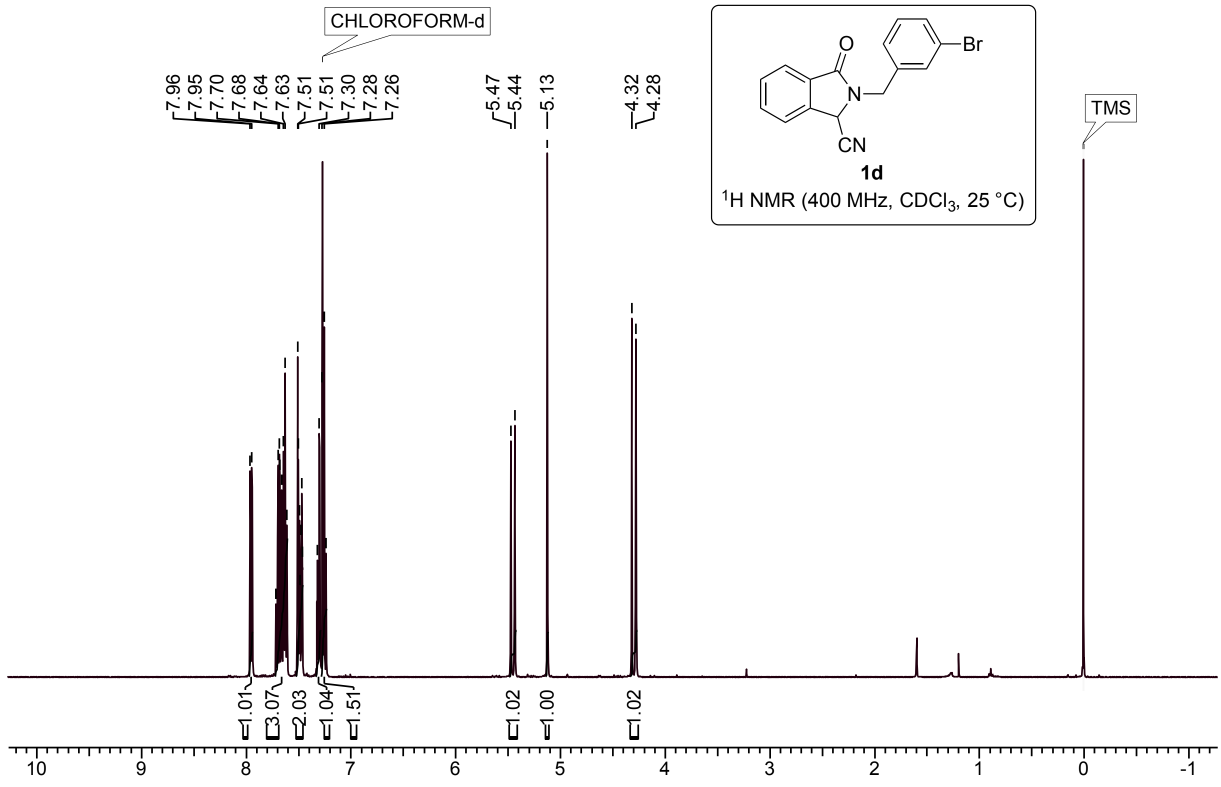
CHLOROFORM-d
7.96
7.95
7.70
7.68
7.64
7.63
7.51
7.51
7.30
7.28
7.26
5.47
5.44
5.13
4.32
4.28
O
N
Br
CN
1d
1H NMR (400 MHz, CDCl3, 25 °C)
TMS
1.01
3.07
2.03
1.04
1.51
1.02
1.00
1.02
10
9
8
7
6
5
4
3
2
1
0
-1

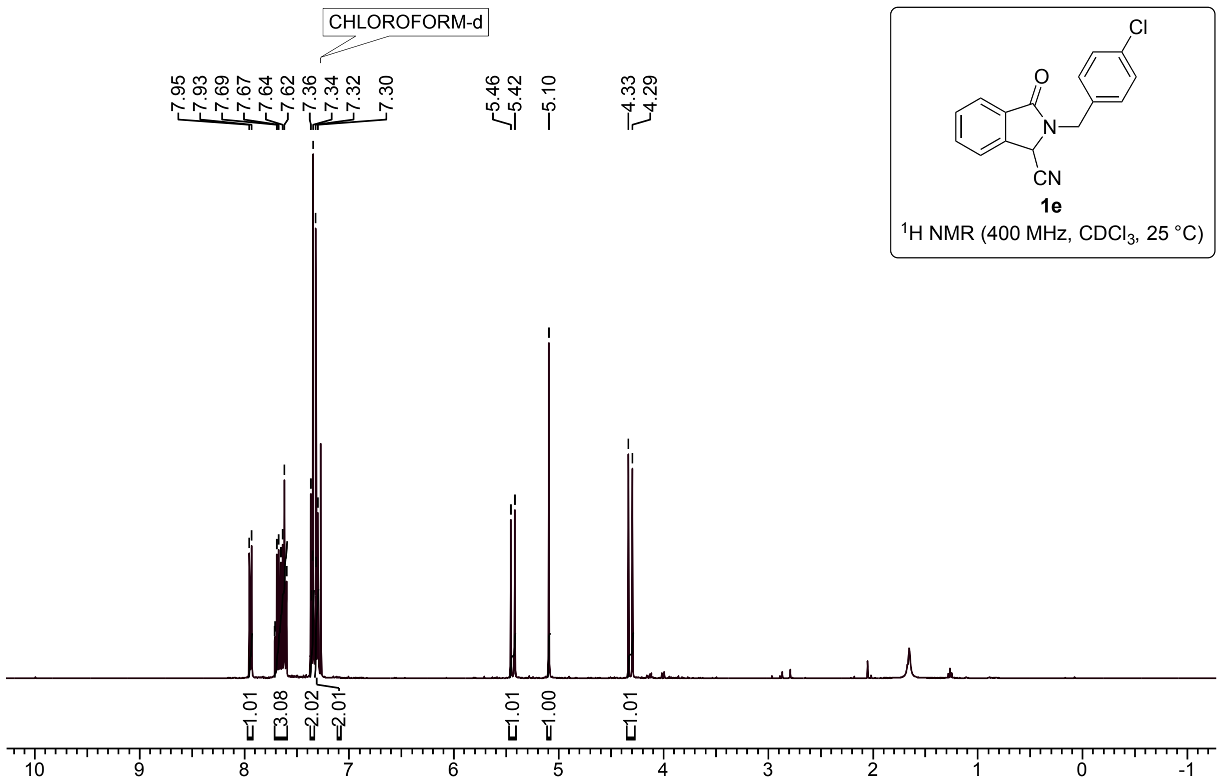
CHLOROFORM-d
7.95
7.93
7.69
7.67
7.64
7.62
7.36
7.34
7.32
7.30
5.46
5.42
5.10
4.33
4.29
Cl
O
N
CN
1e
1H NMR (400 MHz, CDCl3, 25 °C)
1.01
3.08
2.02
2.01
1.01
1.00
1.01
10
9
8
7
6
5
4
3
2
1
0
-1

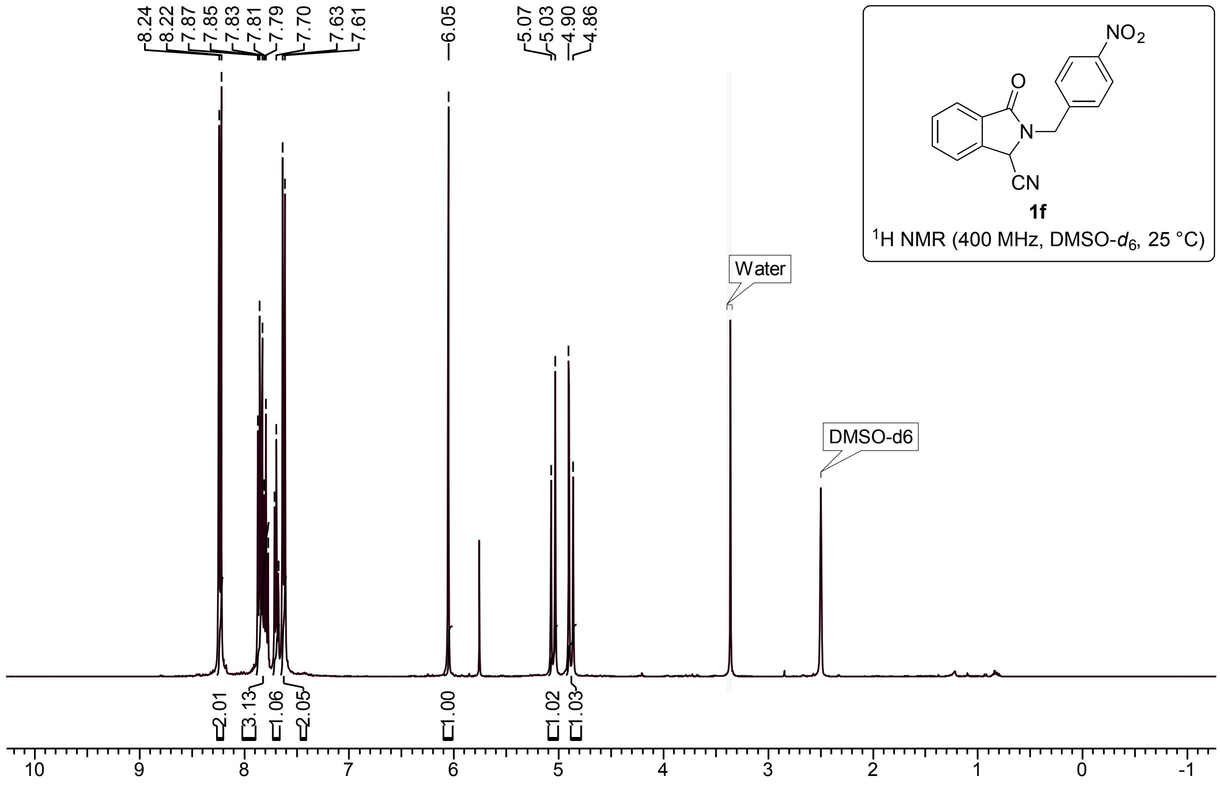
8.24
8.22
7.87
7.85
7.83
7.81
7.79
7.70
7.63
7.61
6.05
5.07
5.03
4.90
4.86
NO2
O
N
CN
1f
1H NMR (400 MHz, DMSO-d6, 25 °C)
Water
DMSO-d6
2.01
3.13
1.06
2.05
1.00
1.02
1.03
10
9
8
7
6
5
4
3
2
1
0
-1

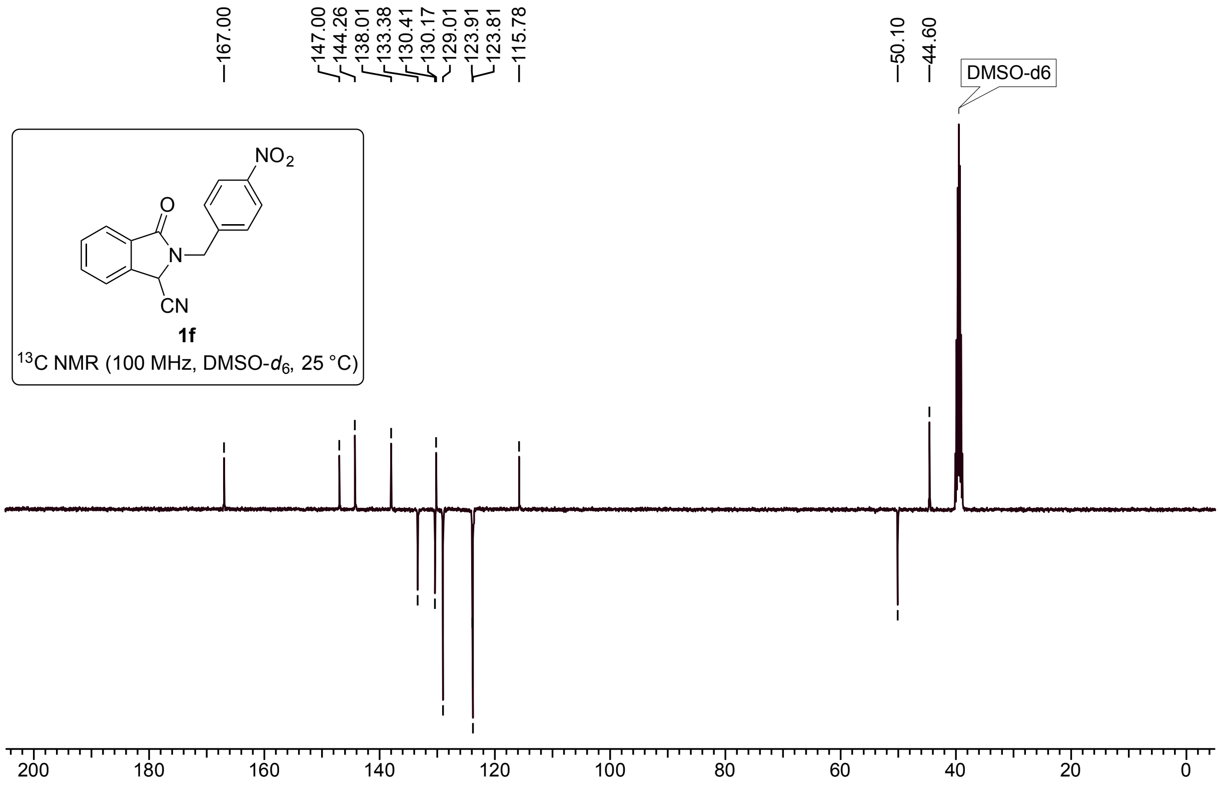
167.00
147.00
144.26
138.01
133.38
130.41
130.17
129.01
123.91
123.81
115.78
50.10
44.60
DMSO-d6
NO2
O
N
CN
1f
13C NMR (100 MHz, DMSO-d6, 25 °C)
200
180
160
140
120
100
80
60
40
20
0

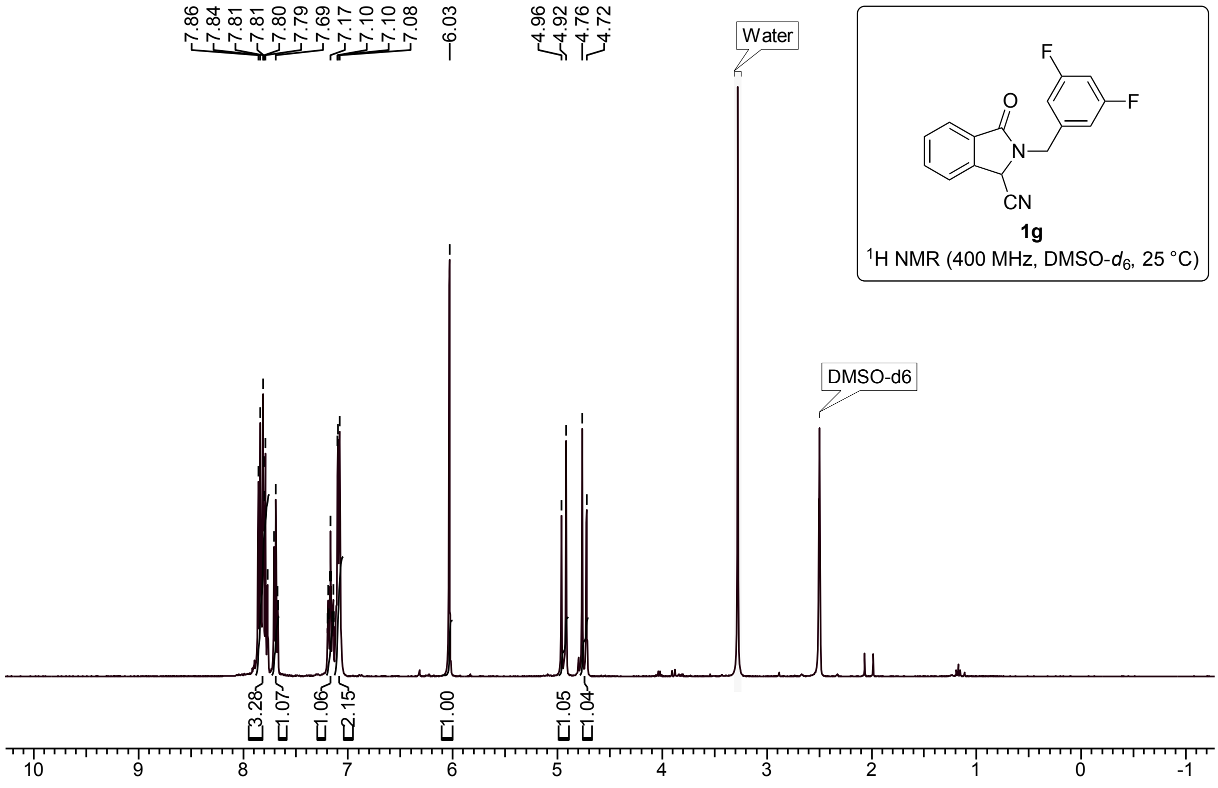
7.86
7.84
7.81
7.81
7.80
7.79
7.69
7.17
7.10
7.10
7.08
6.03
4.96
4.92
4.76
4.72
Water
F
F
O
N
CN
1g
1H NMR (400 MHz, DMSO-d6, 25 °C)
DMSO-d6
3.28
1.07
1.06
2.15
1.00
1.05
1.04
10
9
8
7
6
5
4
3
2
1
0
-1

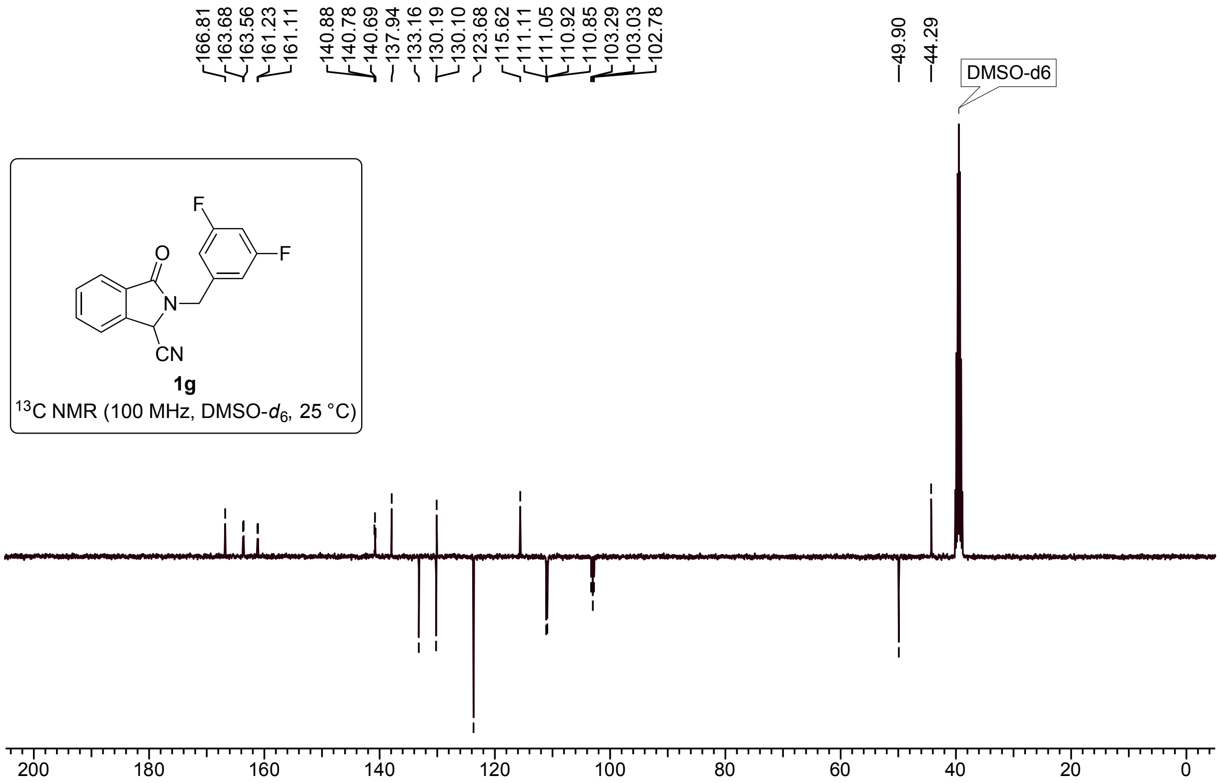

1g
^{13}C NMR (100 MHz, DMSO-d_6, 25 °C)
DMSO-d6
166.81
163.68
163.56
161.23
161.11
140.88
140.78
140.69
137.94
133.16
130.19
130.10
123.68
115.62
111.11
111.05
110.92
110.85
103.29
103.03
102.78
49.90
44.29

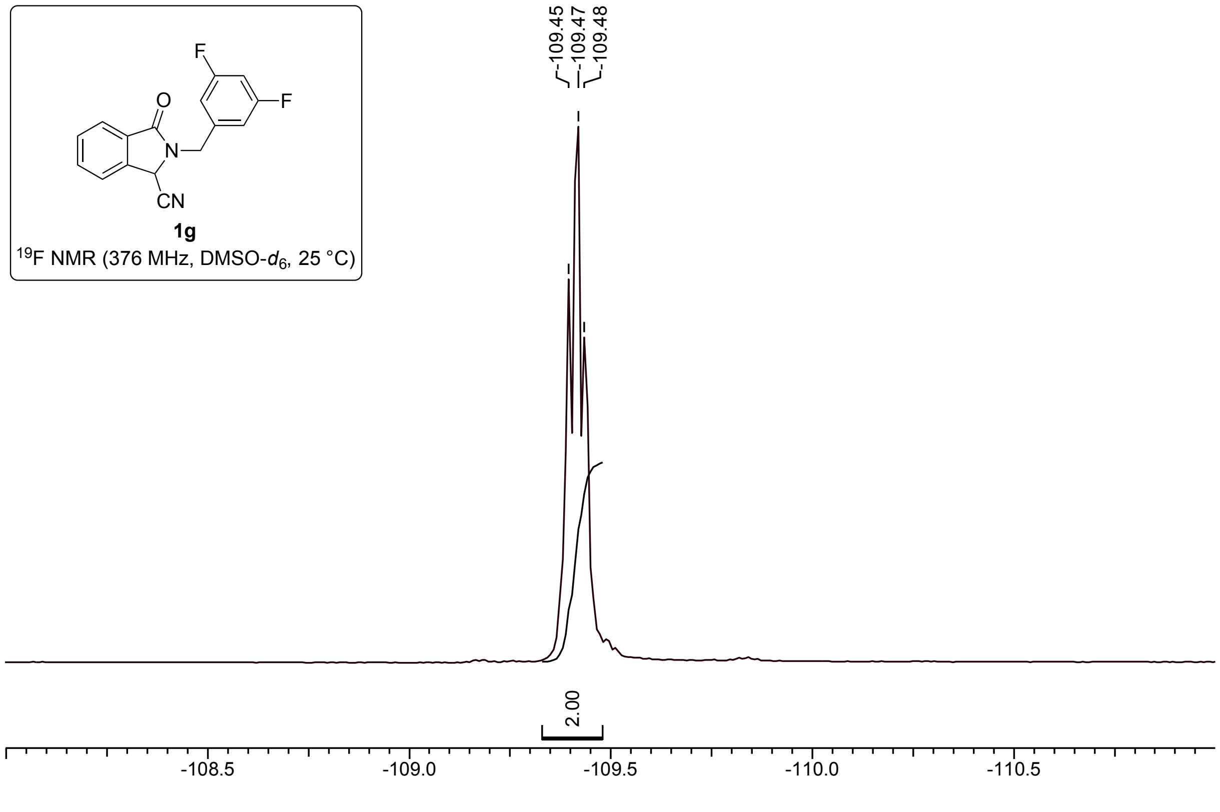

1g
^{19}F NMR (376 MHz, DMSO-d_6, 25 °C)
-109.45
-109.47
-109.48
2.00

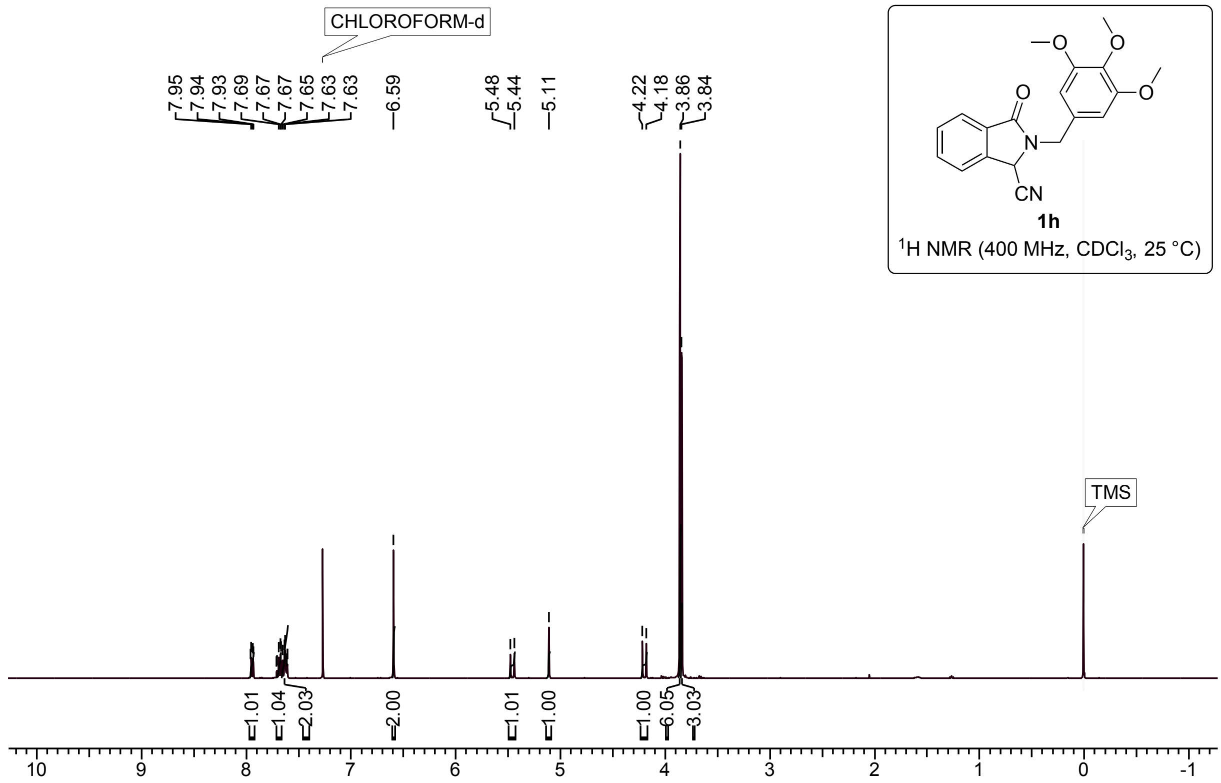

1h

^{1}H NMR (400 MHz, $CDCl_3$, 25 °C)

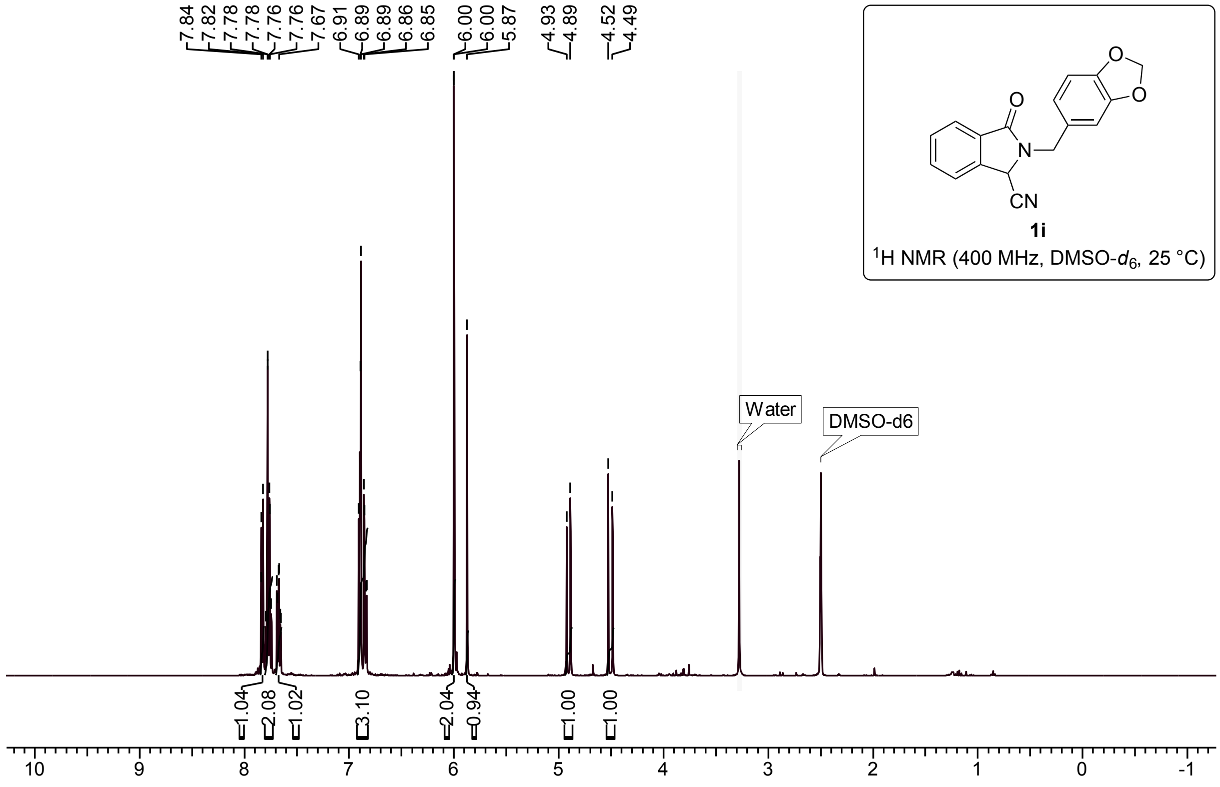

1i

^{1}H NMR (400 MHz, DMSO-d_6, 25 °C)

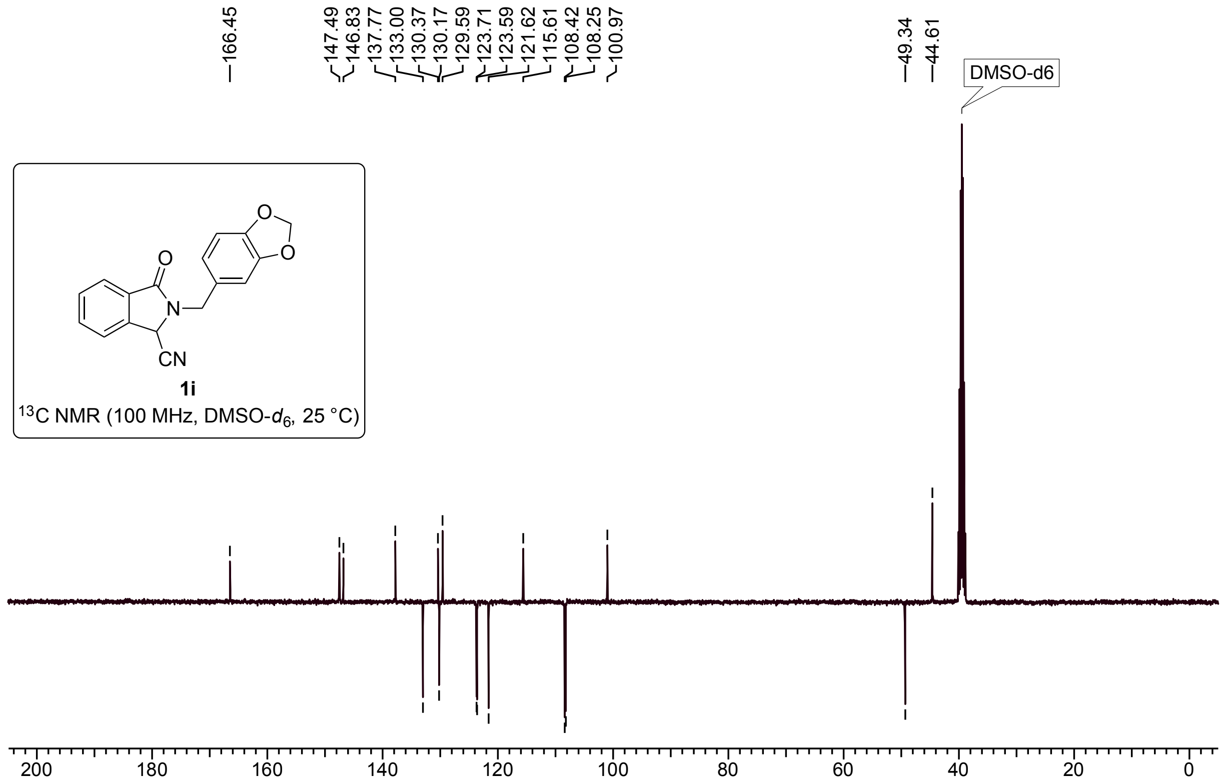
166.45
147.49
146.83
137.77
133.00
130.37
130.17
129.59
123.71
123.59
121.62
115.61
108.42
108.25
100.97
49.34
44.61
DMSO-d6
O
O
O
N
CN
1i
13C NMR (100 MHz, DMSO-d6, 25 °C)
200 180 160 140 120 100 80 60 40 20 0

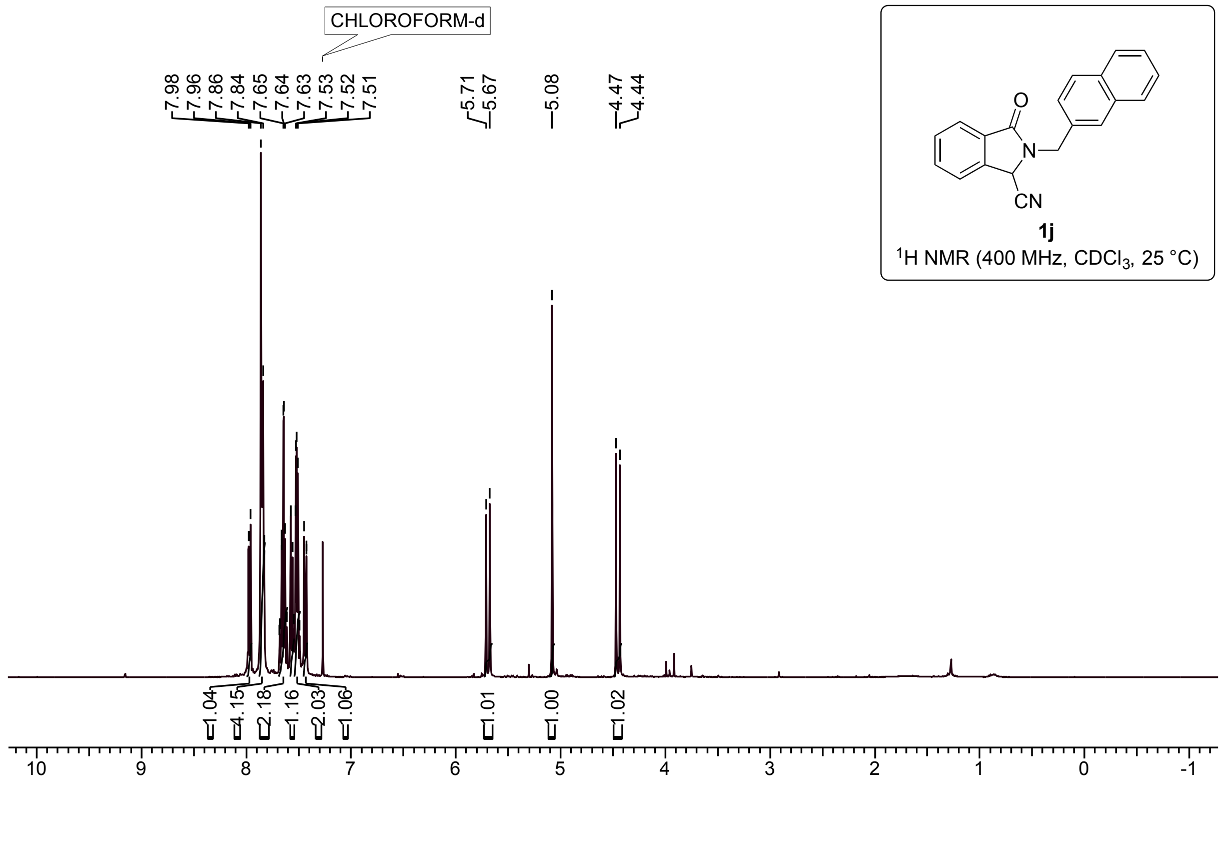
CHLOROFORM-d
7.98
7.96
7.86
7.84
7.65
7.64
7.63
7.53
7.52
7.51
5.71
5.67
5.08
4.47
4.44
O
N
CN
1j
1H NMR (400 MHz, CDCl3, 25 °C)
1.04
4.15
2.18
1.16
2.03
1.06
1.01
1.00
1.02
10 9 8 7 6 5 4 3 2 1 0 -1

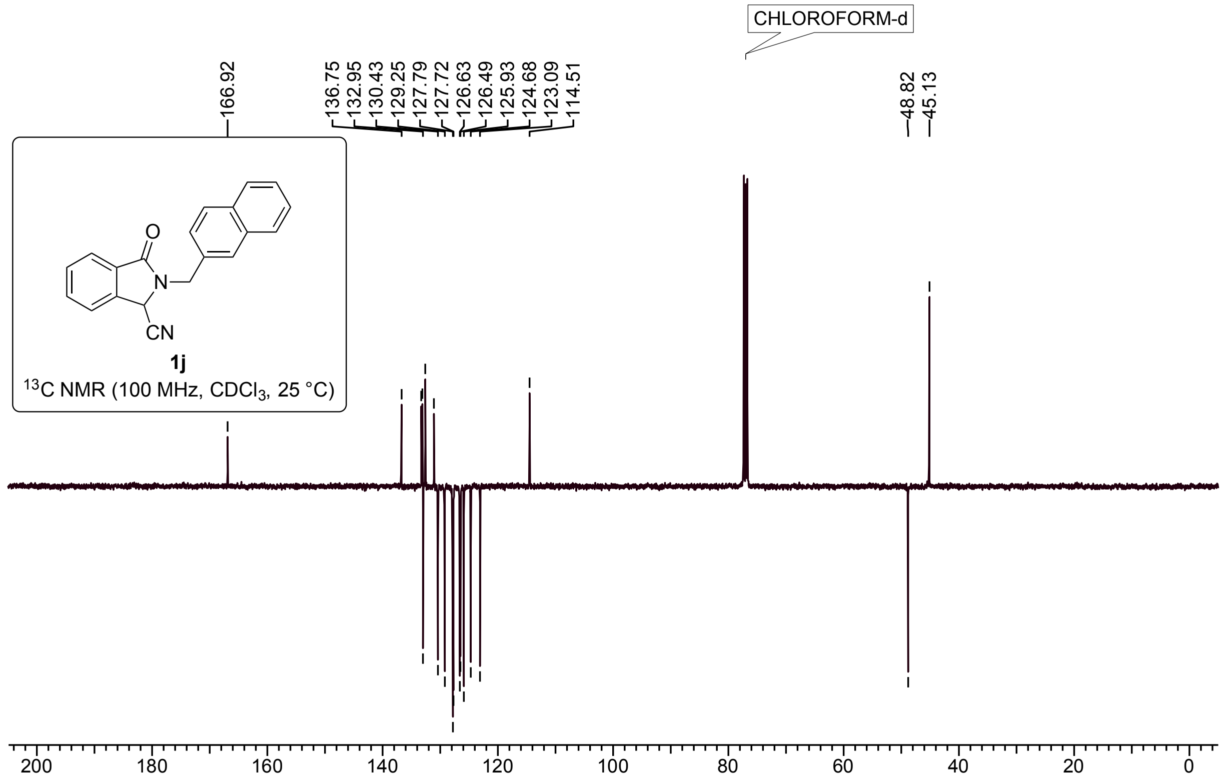

CHLOROFORM-d
166.92
136.75
132.95
130.43
129.25
127.79
127.72
126.63
126.49
125.93
124.68
123.09
114.51
48.82
45.13
O
N
CN
1j
^{13}C NMR (100 MHz, CDCl$_3$, 25 °C)
200
180
160
140
120
100
80
60
40
20
0

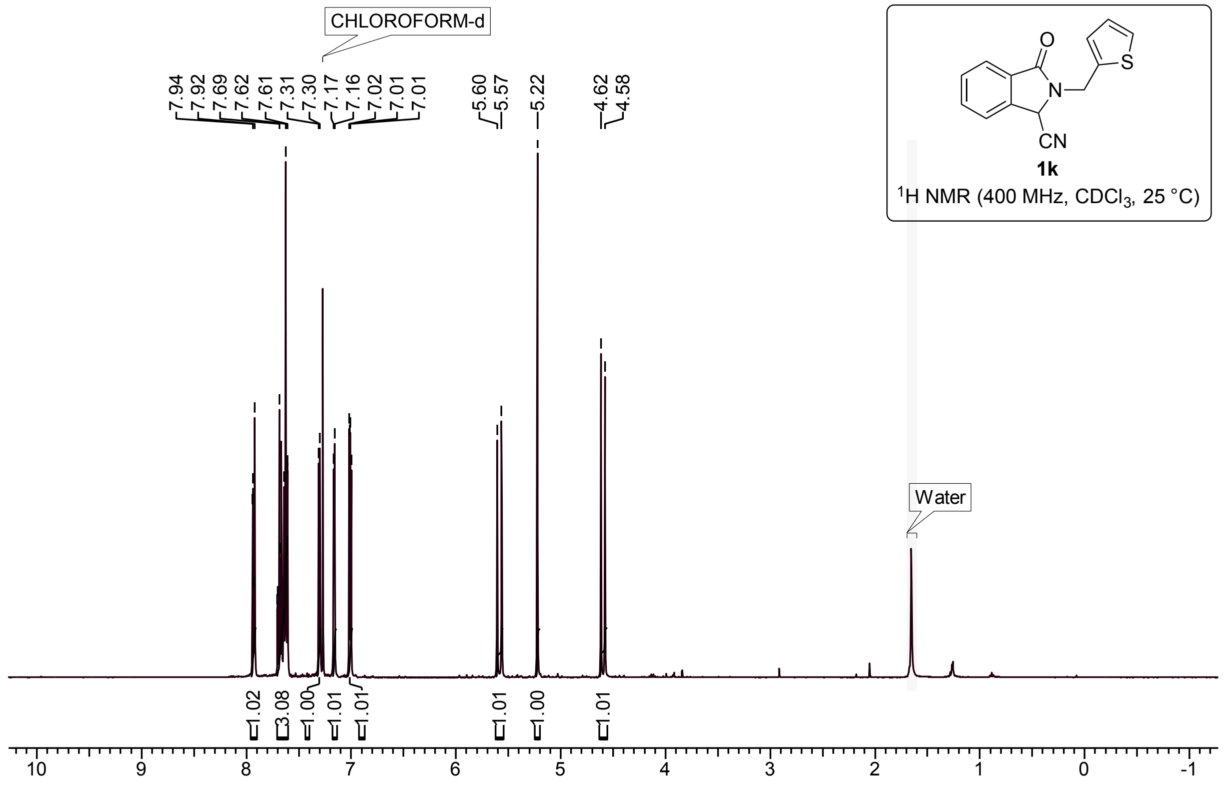

CHLOROFORM-d
7.94
7.92
7.69
7.62
7.61
7.31
7.30
7.17
7.16
7.02
7.01
7.01
5.60
5.57
5.22
4.62
4.58
O
N
S
CN
1k
^{1}H NMR (400 MHz, CDCl$_3$, 25 °C)
Water
1.02
3.08
1.00
1.01
1.01
1.01
1.00
1.01
10
9
8
7
6
5
4
3
2
1
0
-1

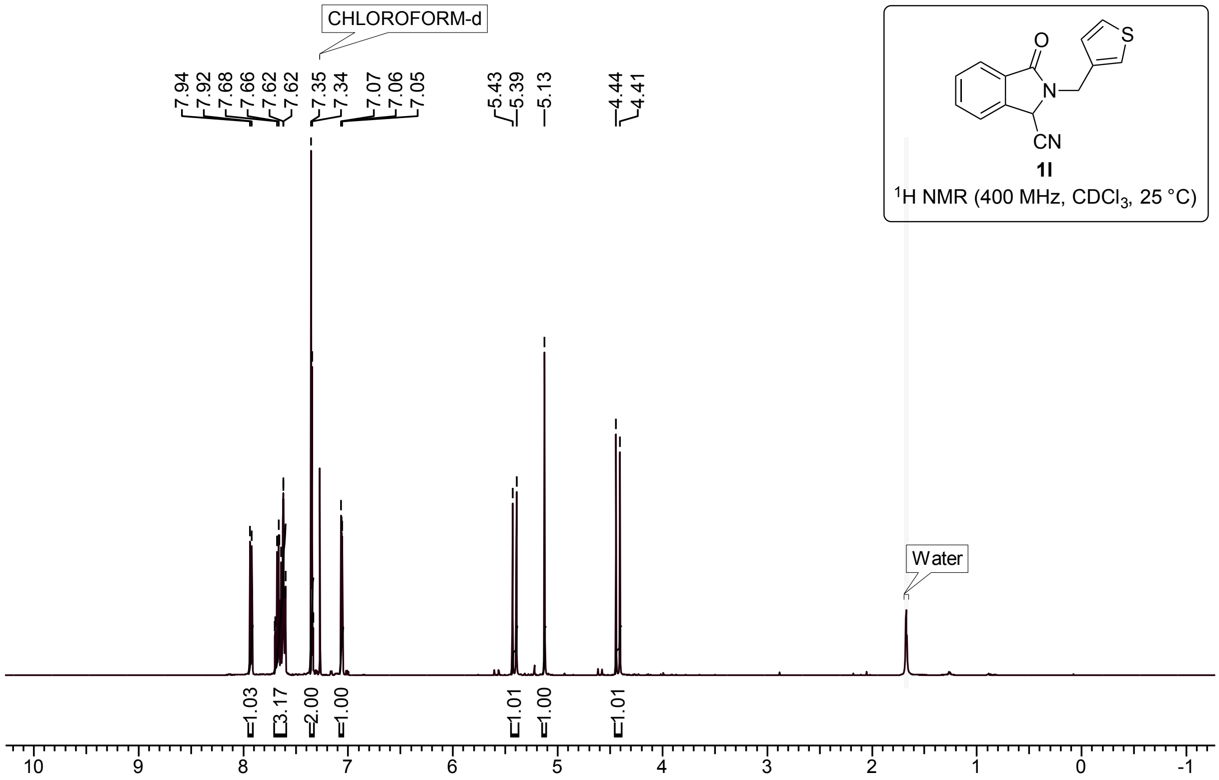
CHLOROFORM-d
7.94 7.92 7.68 7.66 7.62 7.62 7.35 7.34 7.07 7.06 7.05
5.43 5.39 5.13
4.44 4.41
O
S
N
CN
1l
¹H NMR (400 MHz, CDCl₃, 25 °C)
Water
1.03 3.17 2.00 1.00 1.01 1.00 1.01
10 9 8 7 6 5 4 3 2 1 0 -1

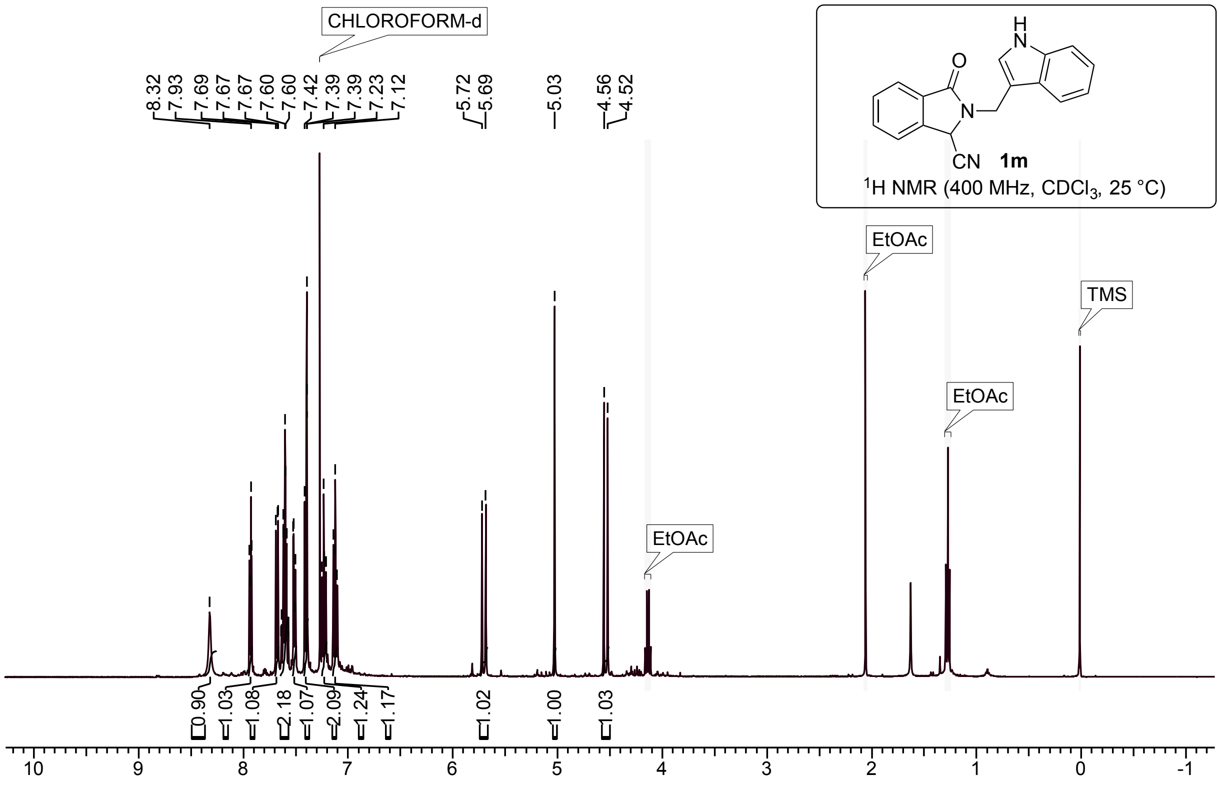
CHLOROFORM-d
8.32 7.93 7.69 7.67 7.60 7.60 7.42 7.39 7.39 7.23 7.12
5.72 5.69 5.03
4.56 4.52
H
N
O
N
CN 1m
¹H NMR (400 MHz, CDCl₃, 25 °C)
EtOAc
TMS
EtOAc
EtOAc
0.90 1.03 1.08 2.18 1.07 2.09 1.24 1.17 1.02 1.00 1.03
10 9 8 7 6 5 4 3 2 1 0 -1

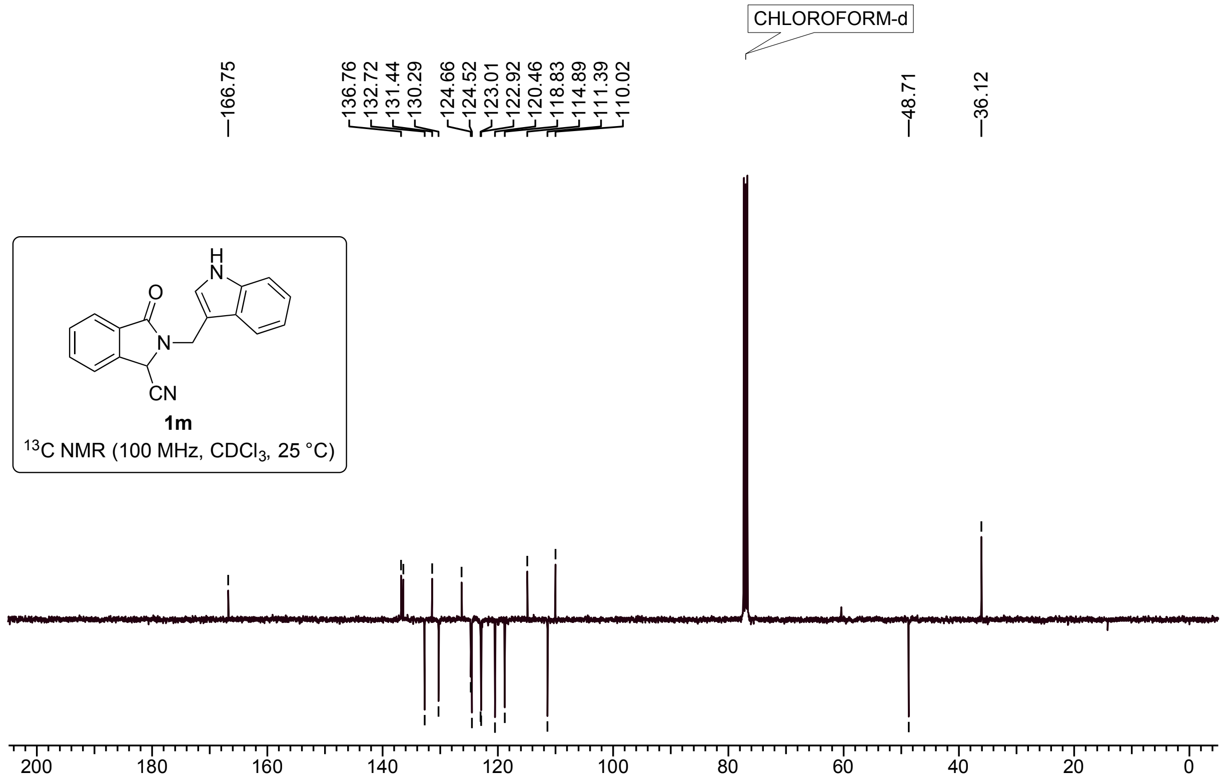
CHLOROFORM-d
166.75
136.76
132.72
131.44
130.29
124.66
124.52
123.01
122.92
120.46
118.83
114.89
111.39
110.02
48.71
36.12
1m
^{13}C NMR (100 MHz, CDCl$_3$, 25 °C)
200 180 160 140 120 100 80 60 40 20 0

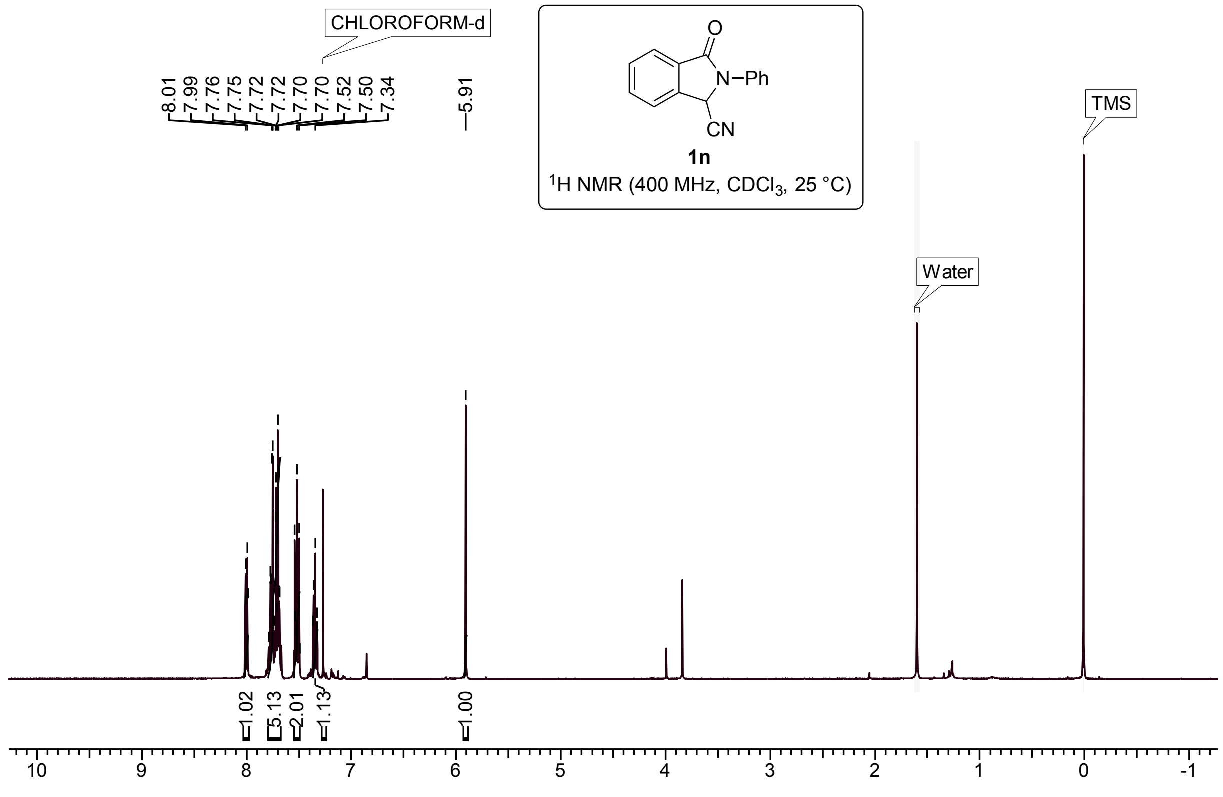
CHLOROFORM-d
8.01
7.99
7.76
7.75
7.72
7.72
7.70
7.70
7.52
7.50
7.34
5.91
1n
^{1}H NMR (400 MHz, CDCl$_3$, 25 °C)
TMS
Water
1.02
5.13
2.01
1.13
1.00
10 9 8 7 6 5 4 3 2 1 0 -1

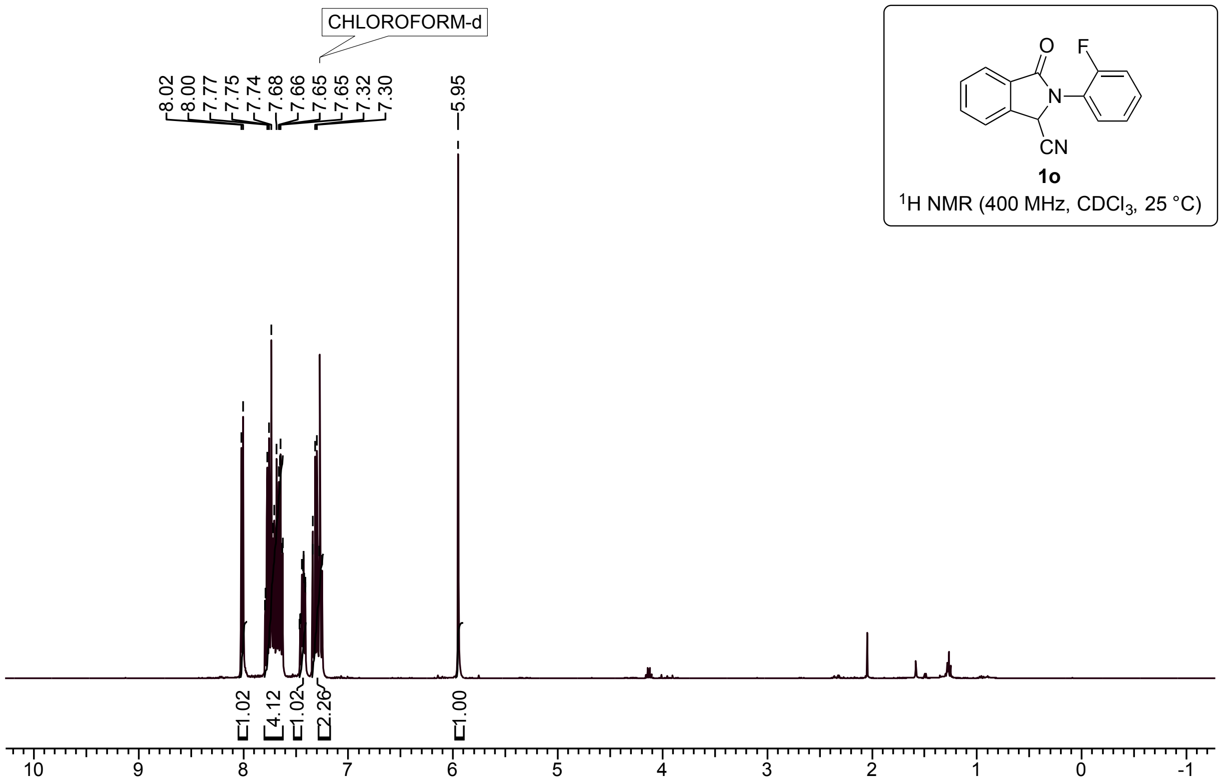
CHLOROFORM-d
8.02
8.00
7.77
7.75
7.74
7.68
7.66
7.65
7.65
7.32
7.30
5.95
1o
^{1}H NMR (400 MHz, CDCl$_3$, 25 °C)
1.02
4.12
1.02
2.26
1.00
10
9
8
7
6
5
4
3
2
1
0
-1

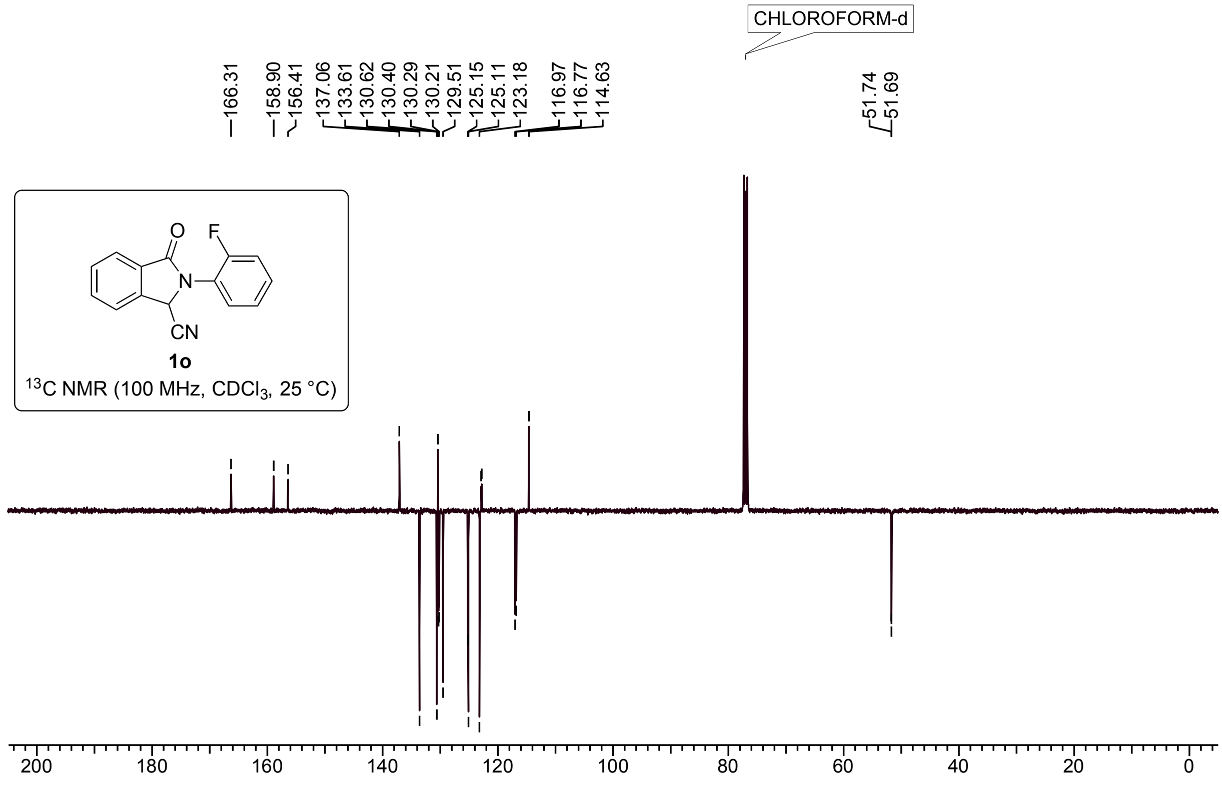
CHLOROFORM-d
166.31
158.90
156.41
137.06
133.61
130.62
130.40
130.29
130.21
129.51
125.15
125.11
123.18
116.97
116.77
114.63
51.74
51.69
1o
^{13}C NMR (100 MHz, CDCl$_3$, 25 °C)
200
180
160
140
120
100
80
60
40
20
0

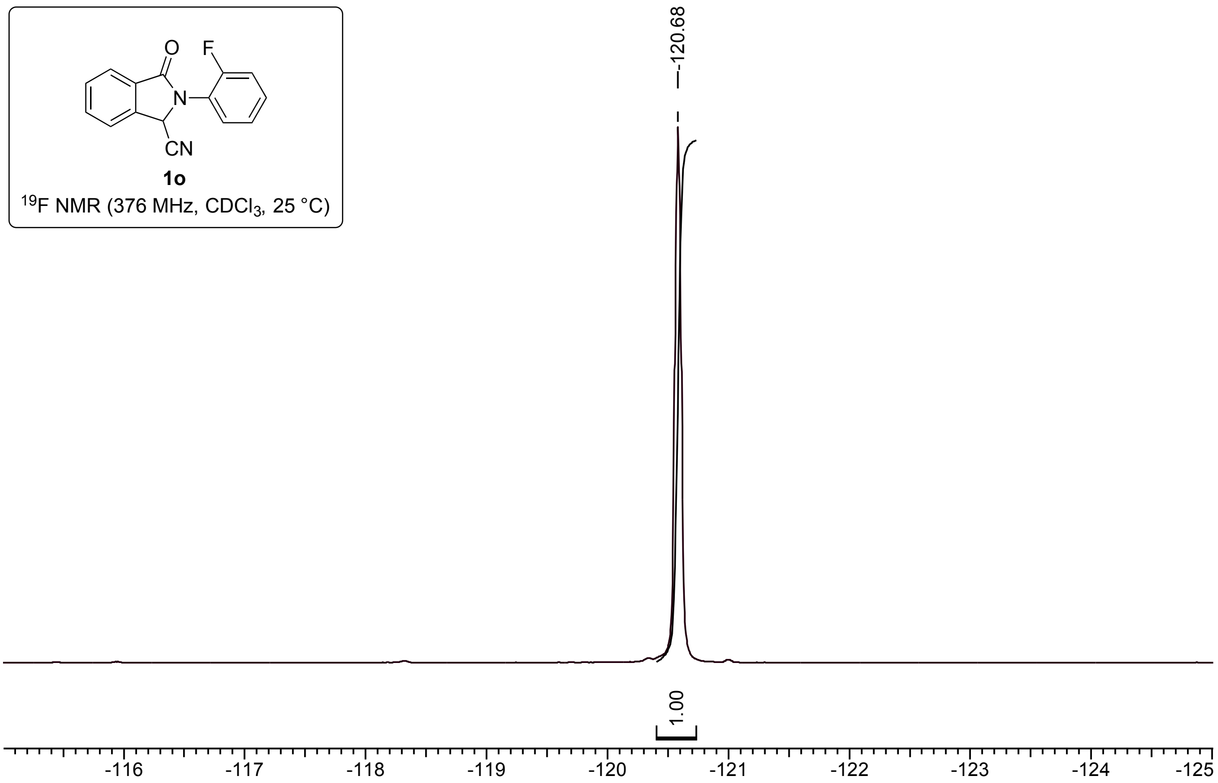

O
F
N
CN
1o
^{19}F NMR (376 MHz, CDCl$_3$, 25 °C)
-120.68
1.00
-116
-117
-118
-119
-120
-121
-122
-123
-124
-125

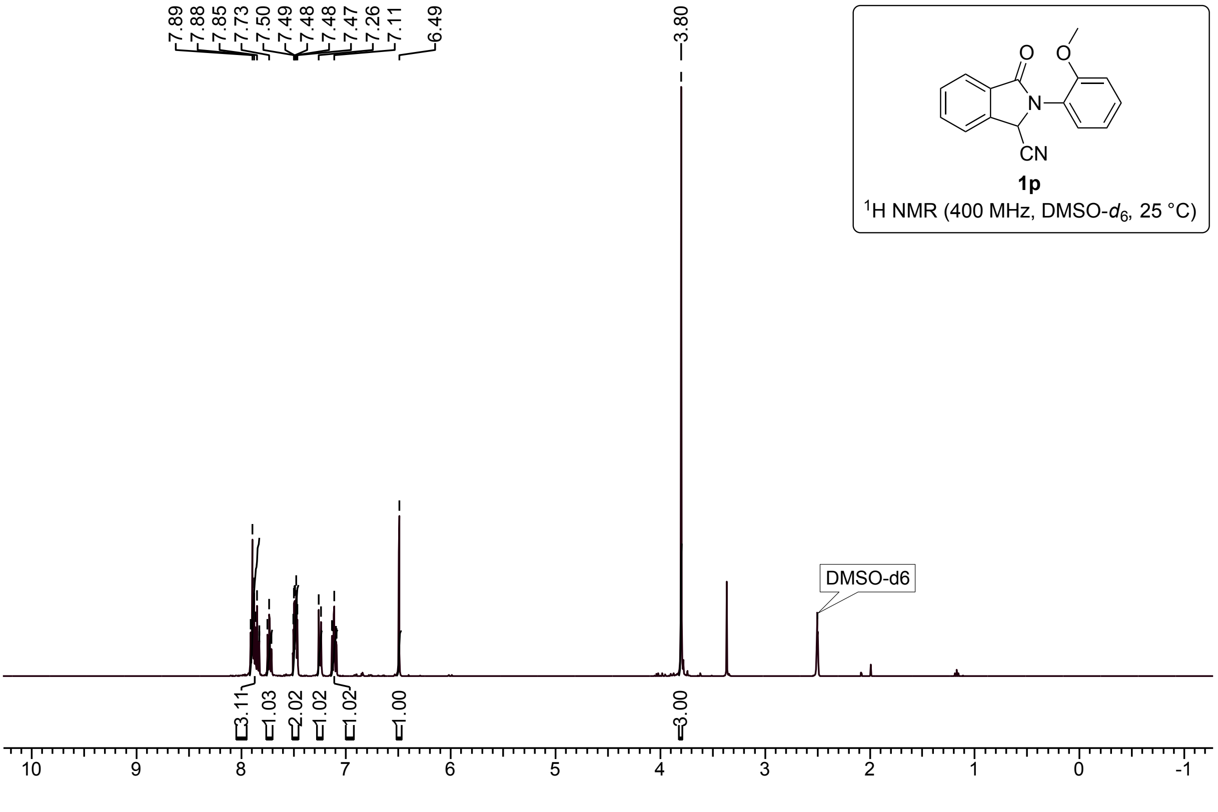

7.89
7.88
7.85
7.73
7.50
7.49
7.48
7.48
7.47
7.26
7.11
6.49
3.80
O
O
N
CN
1p
^{1}H NMR (400 MHz, DMSO-d_6, 25 °C)
DMSO-d6
3.11
1.03
2.02
1.02
1.02
1.00
3.00
10
9
8
7
6
5
4
3
2
1
0
-1

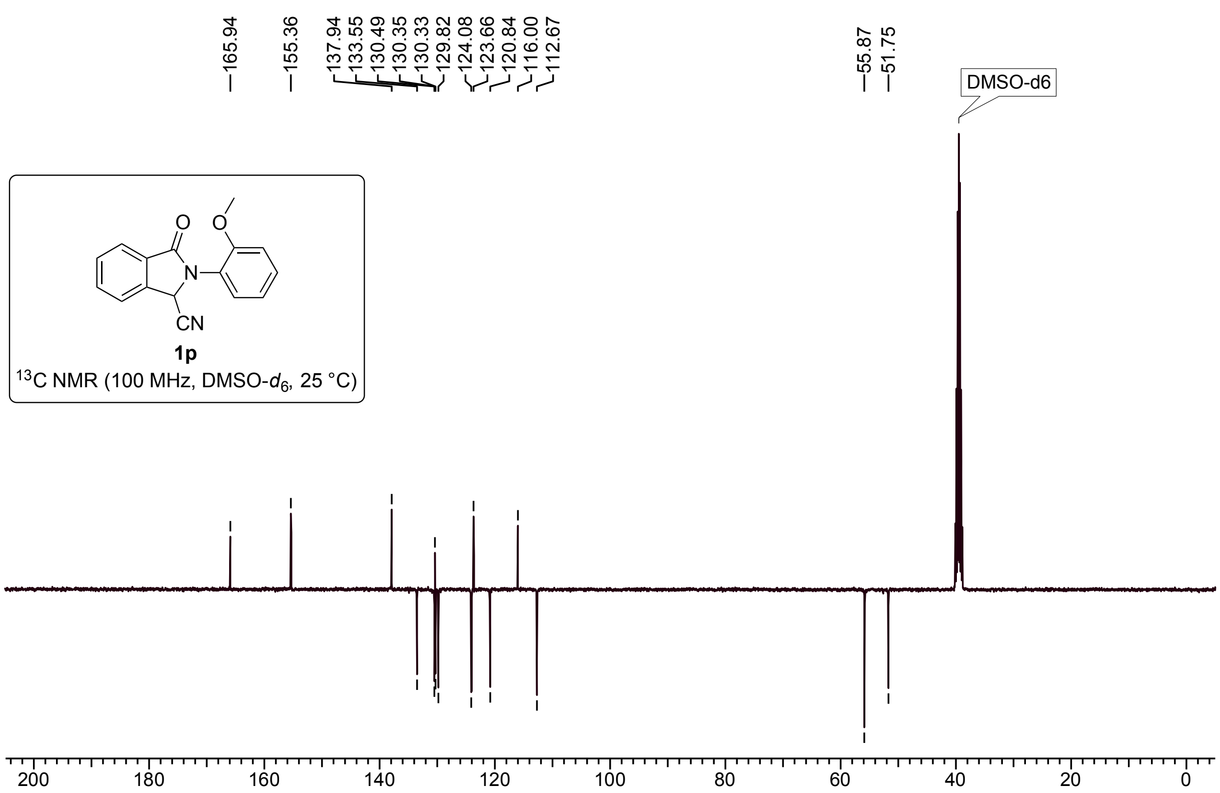
165.94
155.36
137.94
133.55
130.49
130.35
130.33
129.82
124.08
123.66
120.84
116.00
112.67
55.87
51.75
DMSO-d6
O
N
CN
1p
13C NMR (100 MHz, DMSO-d6, 25 °C)
200
180
160
140
120
100
80
60
40
20
0

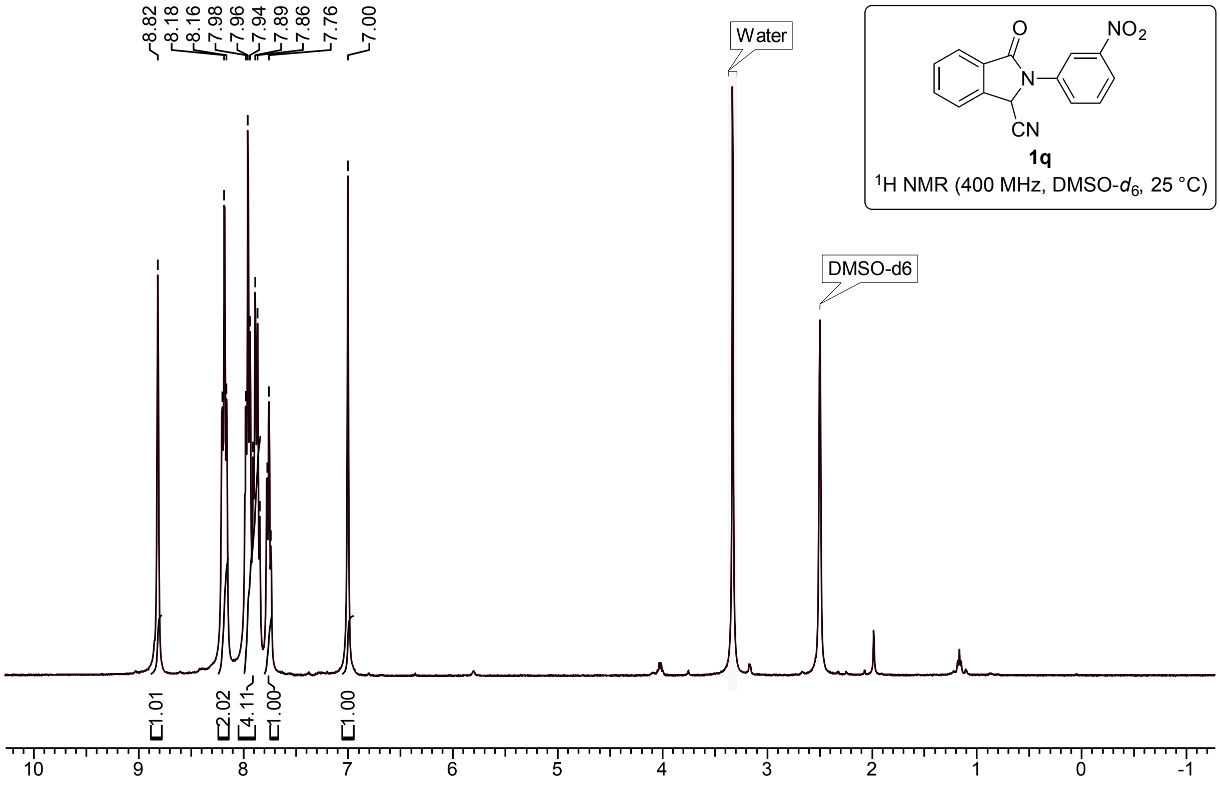
8.82
8.18
8.16
7.98
7.96
7.94
7.89
7.86
7.76
7.00
Water
O
NO2
N
CN
1q
1H NMR (400 MHz, DMSO-d6, 25 °C)
DMSO-d6
1.01
2.02
4.11
1.00
1.00
10
9
8
7
6
5
4
3
2
1
0
-1

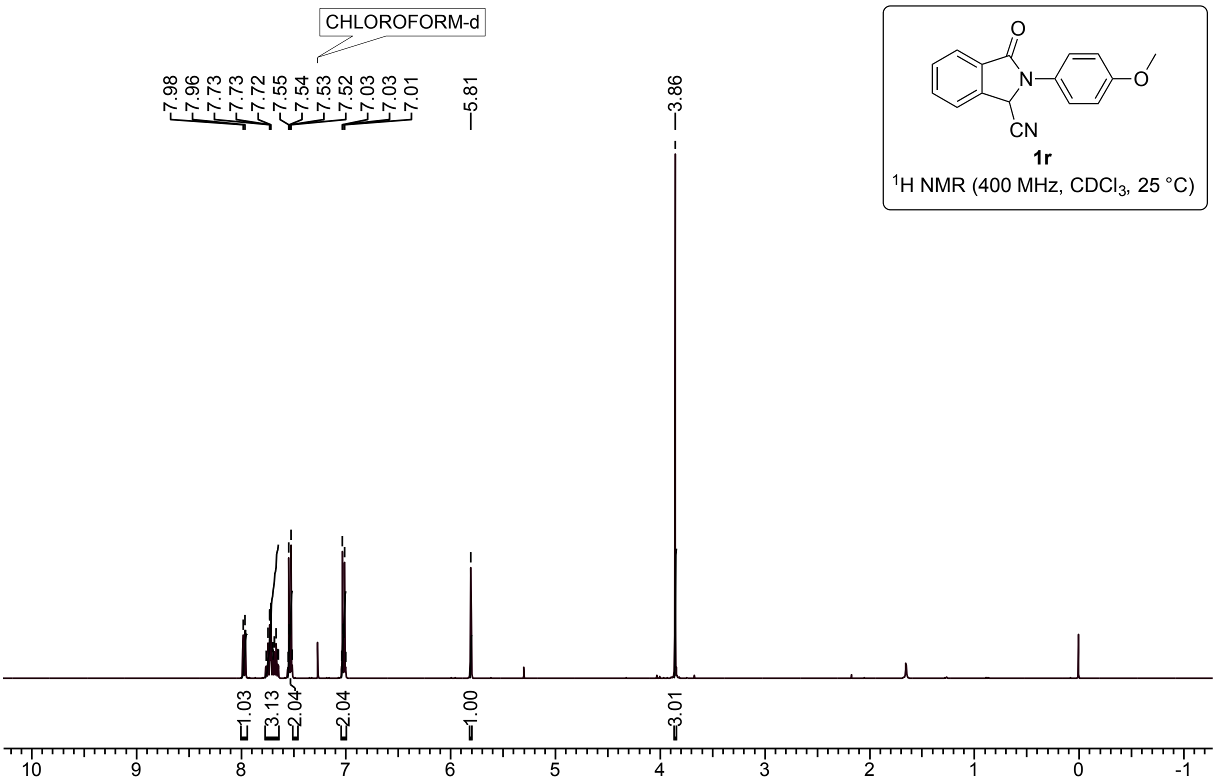
CHLOROFORM-d
7.98 7.96 7.73 7.73 7.72 7.55 7.54 7.53 7.52 7.03 7.03 7.01
5.81
3.86
O
N
O
CN
1r
1H NMR (400 MHz, CDCl3, 25 °C)
1.03 3.13 2.04 2.04 1.00 3.01
10 9 8 7 6 5 4 3 2 1 0 -1

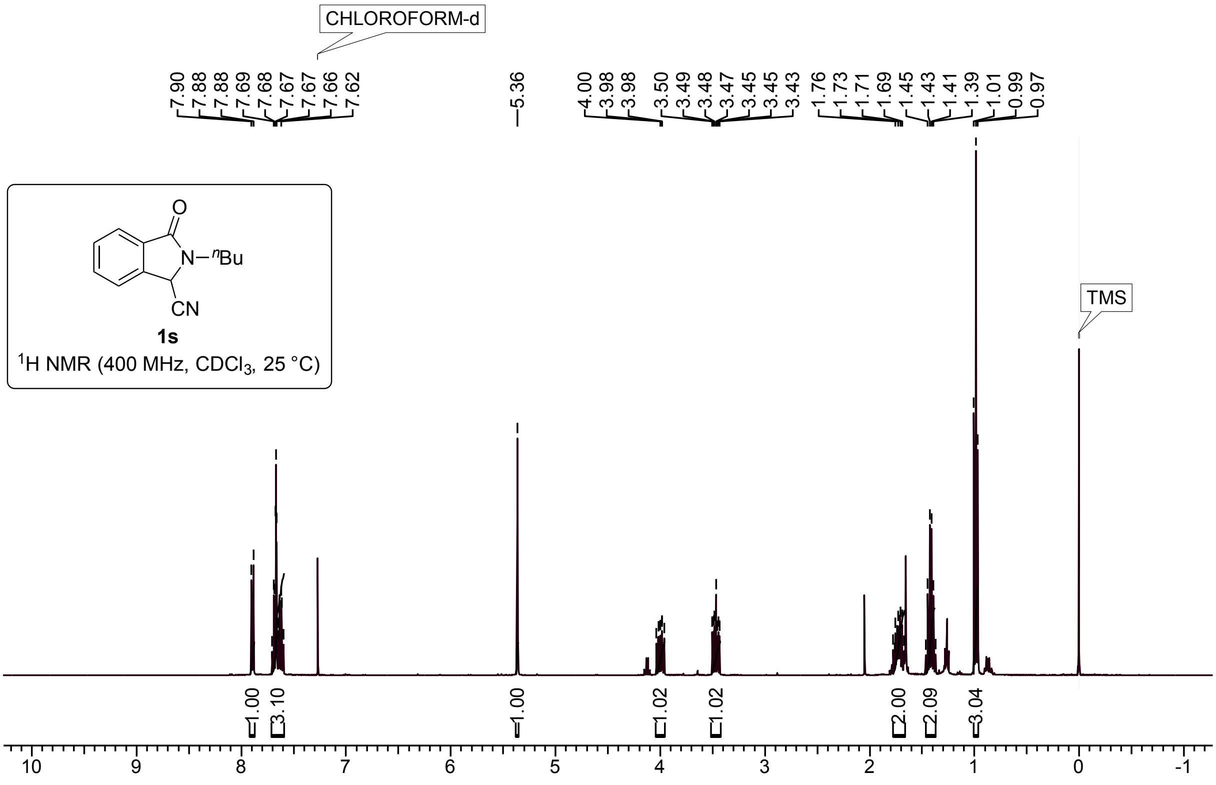
CHLOROFORM-d
7.90 7.88 7.69 7.68 7.67 7.67 7.66 7.62
5.36
4.00 3.98 3.98 3.50 3.49 3.48 3.47 3.45 3.45 3.43
1.76 1.73 1.71 1.69 1.45 1.43 1.41 1.39 1.01 0.99 0.97
O
N–nBu
CN
1s
1H NMR (400 MHz, CDCl3, 25 °C)
TMS
1.00 3.10 1.00 1.02 1.02 2.00 2.09 3.04
10 9 8 7 6 5 4 3 2 1 0 -1

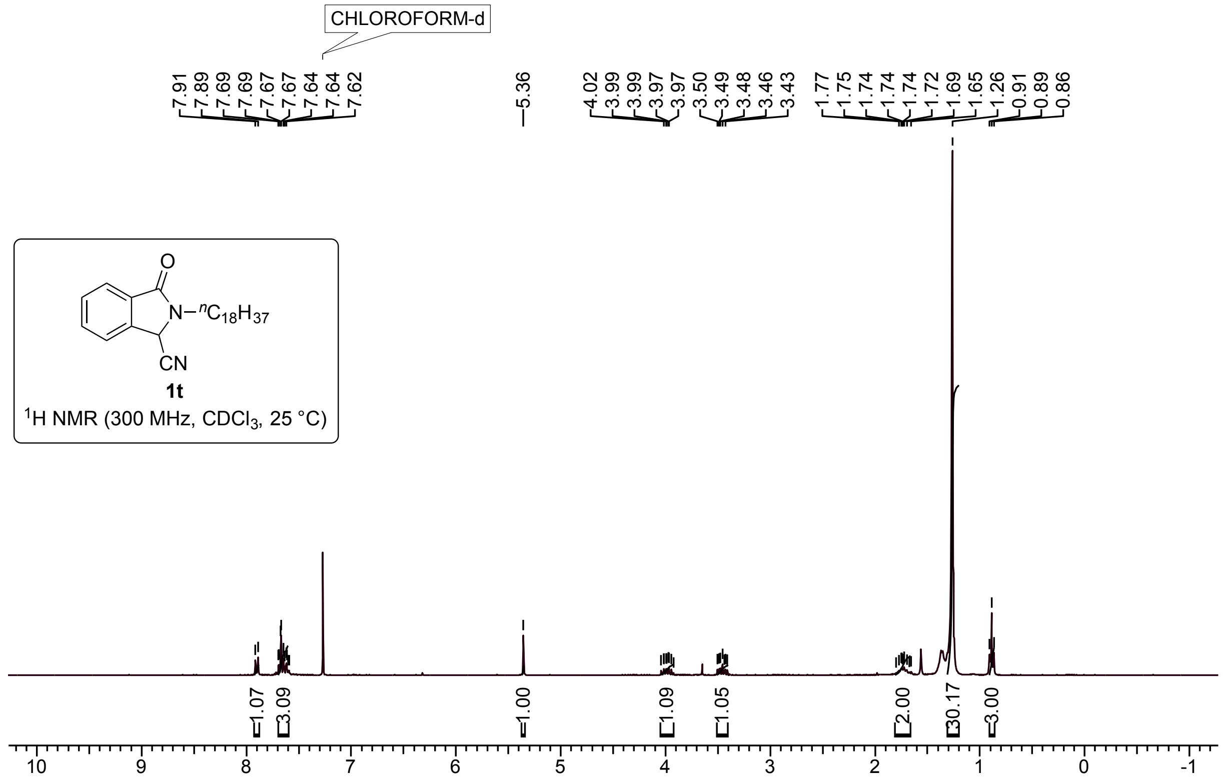
CHLOROFORM-d
7.91 7.89 7.69 7.69 7.67 7.67 7.64 7.64 7.62
5.36
4.02 3.99 3.99 3.97 3.97 3.50 3.49 3.48 3.46 3.43
1.77 1.75 1.74 1.74 1.74 1.72 1.69 1.65 1.26 0.91 0.89 0.86
1t
1H NMR (300 MHz, CDCl3, 25 °C)
1.07 3.09 1.00 1.09 1.05 2.00 30.17 3.00
10 9 8 7 6 5 4 3 2 1 0 -1

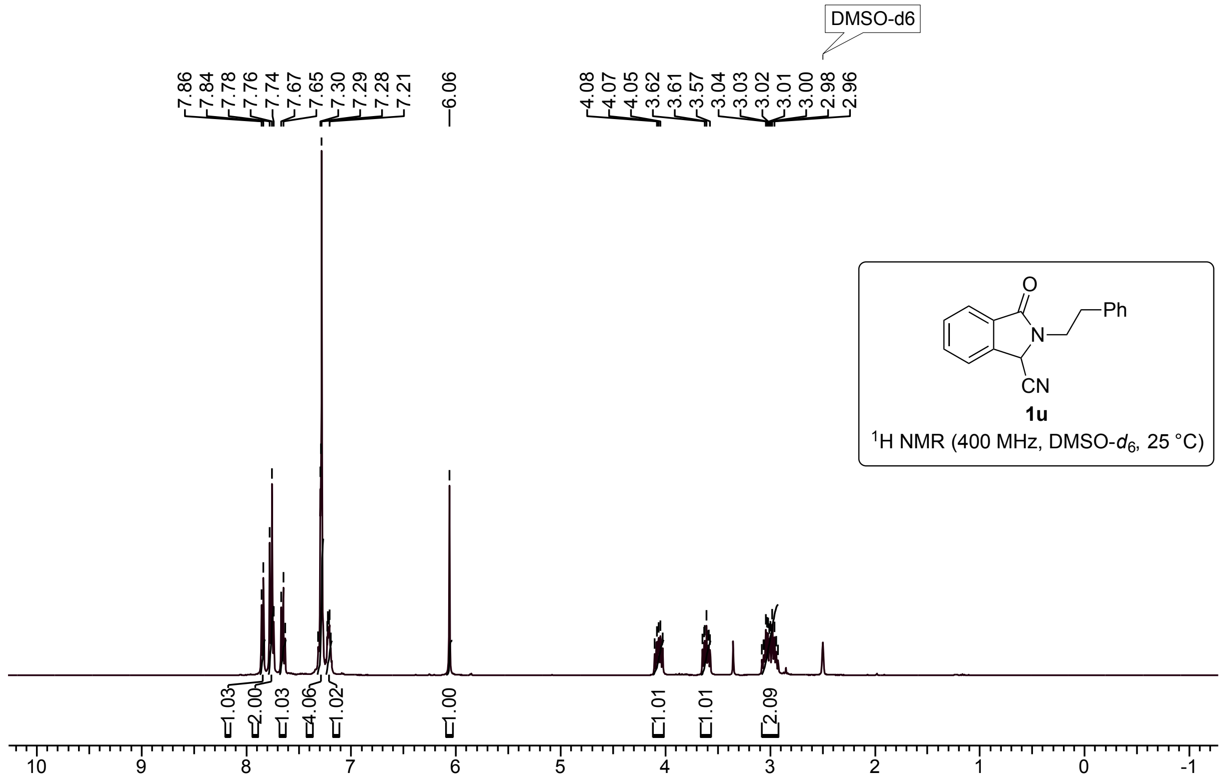
DMSO-d6
7.86 7.84 7.78 7.76 7.74 7.67 7.65 7.30 7.29 7.28 7.21
6.06
4.08 4.07 4.05 3.62 3.61 3.57 3.04 3.03 3.02 3.01 3.00 2.98 2.96
1u
1H NMR (400 MHz, DMSO-d6, 25 °C)
1.03 2.00 1.03 4.06 1.02 1.00 1.01 1.01 2.09
10 9 8 7 6 5 4 3 2 1 0 -1

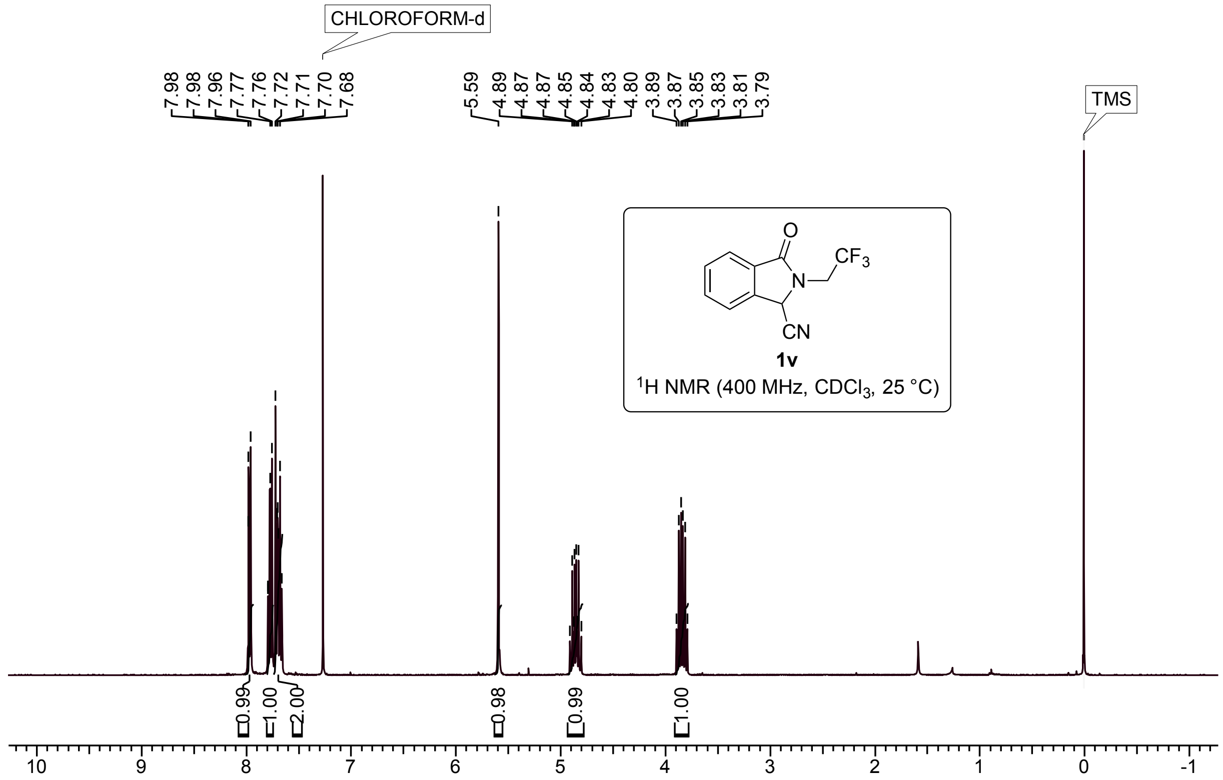

CHLOROFORM-d
7.98
7.98
7.96
7.77
7.76
7.72
7.71
7.70
7.68
5.59
4.89
4.87
4.87
4.85
4.84
4.83
4.80
3.89
3.87
3.85
3.83
3.81
3.79
TMS
O
CF3
N
CN
1v
1H NMR (400 MHz, CDCl3, 25 °C)
0.99
1.00
2.00
0.98
0.99
1.00
10
9
8
7
6
5
4
3
2
1
0
-1

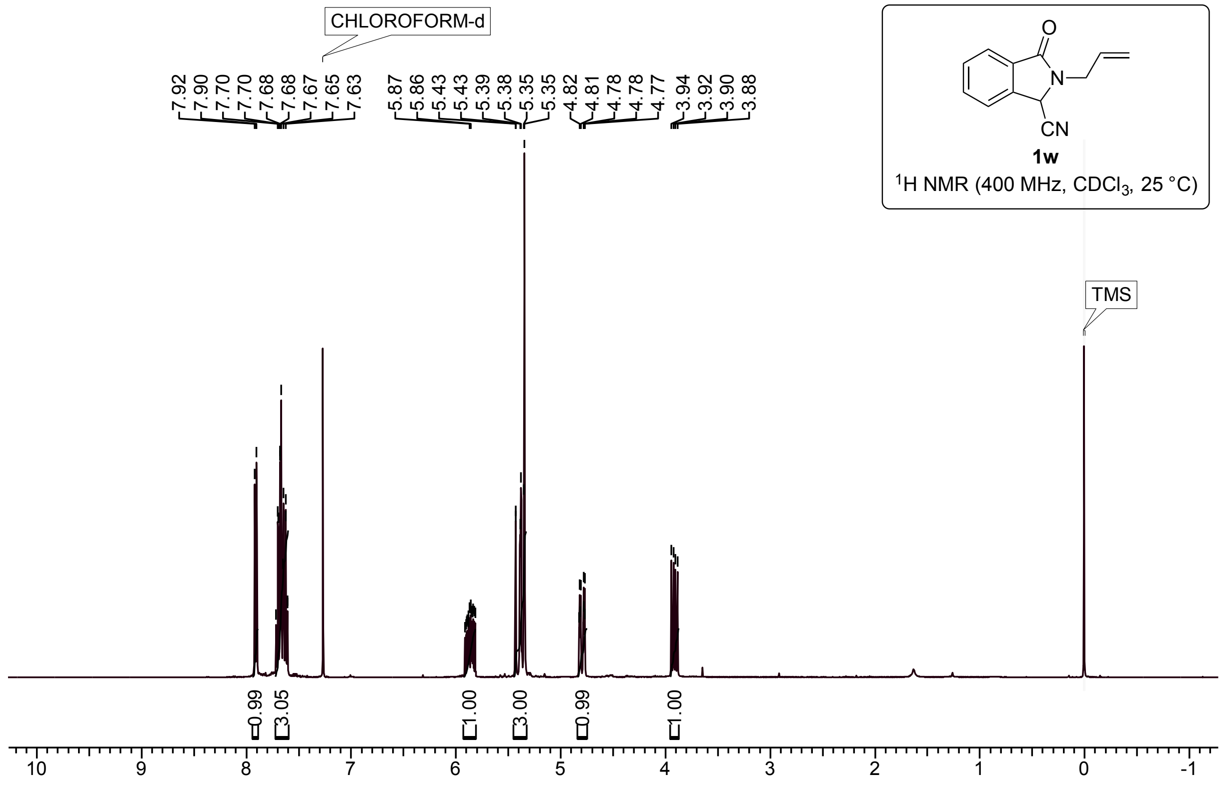

CHLOROFORM-d
7.92
7.90
7.70
7.70
7.68
7.68
7.67
7.65
7.63
5.87
5.86
5.43
5.43
5.39
5.38
5.35
5.35
4.82
4.81
4.78
4.77
3.94
3.92
3.90
3.88
O
N
CN
1w
1H NMR (400 MHz, CDCl3, 25 °C)
TMS
0.99
3.05
1.00
3.00
0.99
1.00
10
9
8
7
6
5
4
3
2
1
0
-1

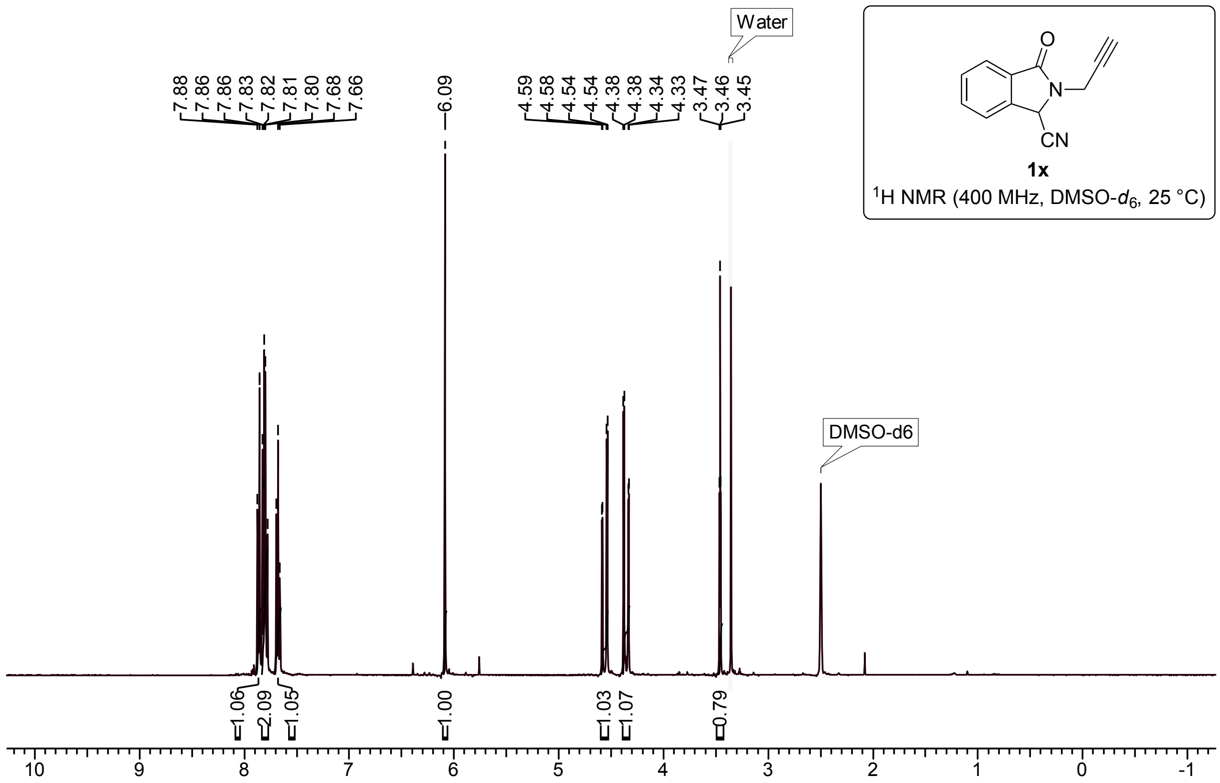
7.88
7.86
7.86
7.83
7.82
7.81
7.80
7.68
7.66
6.09
4.59
4.58
4.54
4.54
4.38
4.38
4.34
4.33
3.47
3.46
3.45
Water
1x
1H NMR (400 MHz, DMSO-d6, 25 °C)
DMSO-d6
1.06
2.09
1.05
1.00
1.03
1.07
0.79
10
9
8
7
6
5
4
3
2
1
0
-1

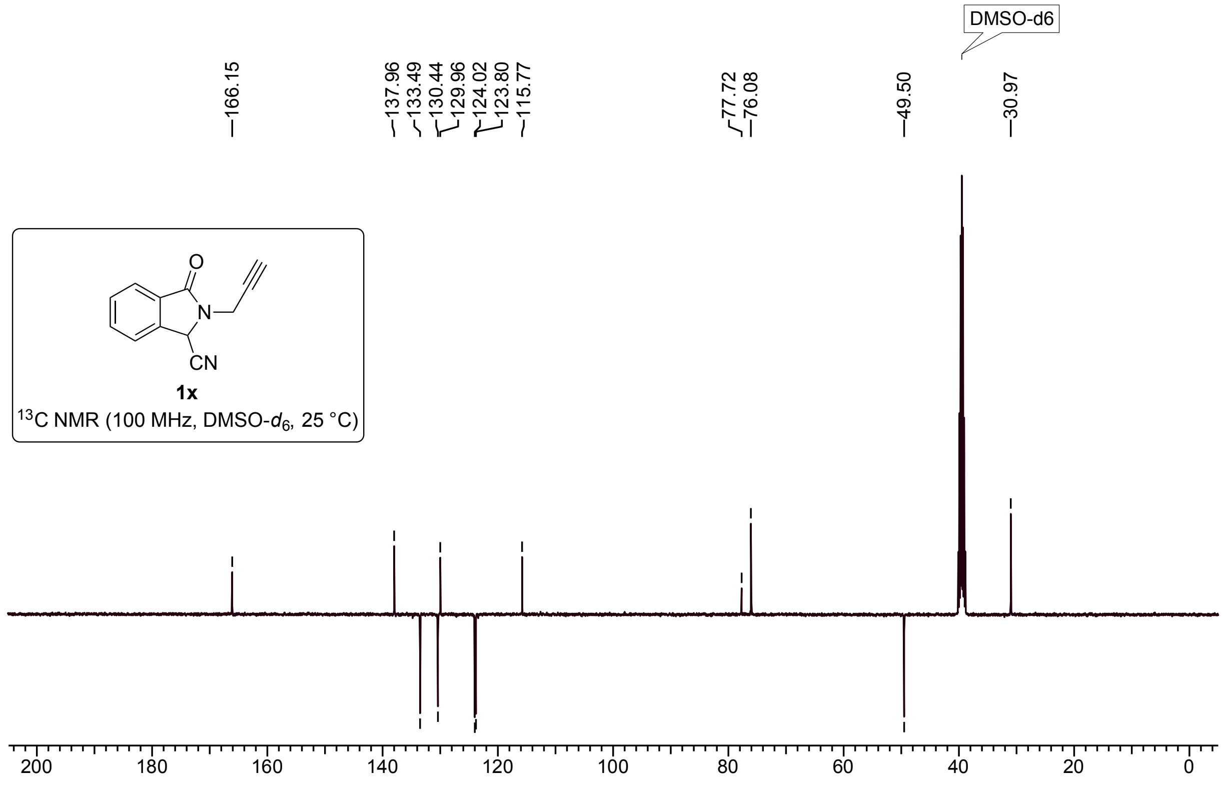
DMSO-d6
166.15
137.96
133.49
130.44
129.96
124.02
123.80
115.77
77.72
76.08
49.50
30.97
1x
13C NMR (100 MHz, DMSO-d6, 25 °C)
200
180
160
140
120
100
80
60
40
20
0

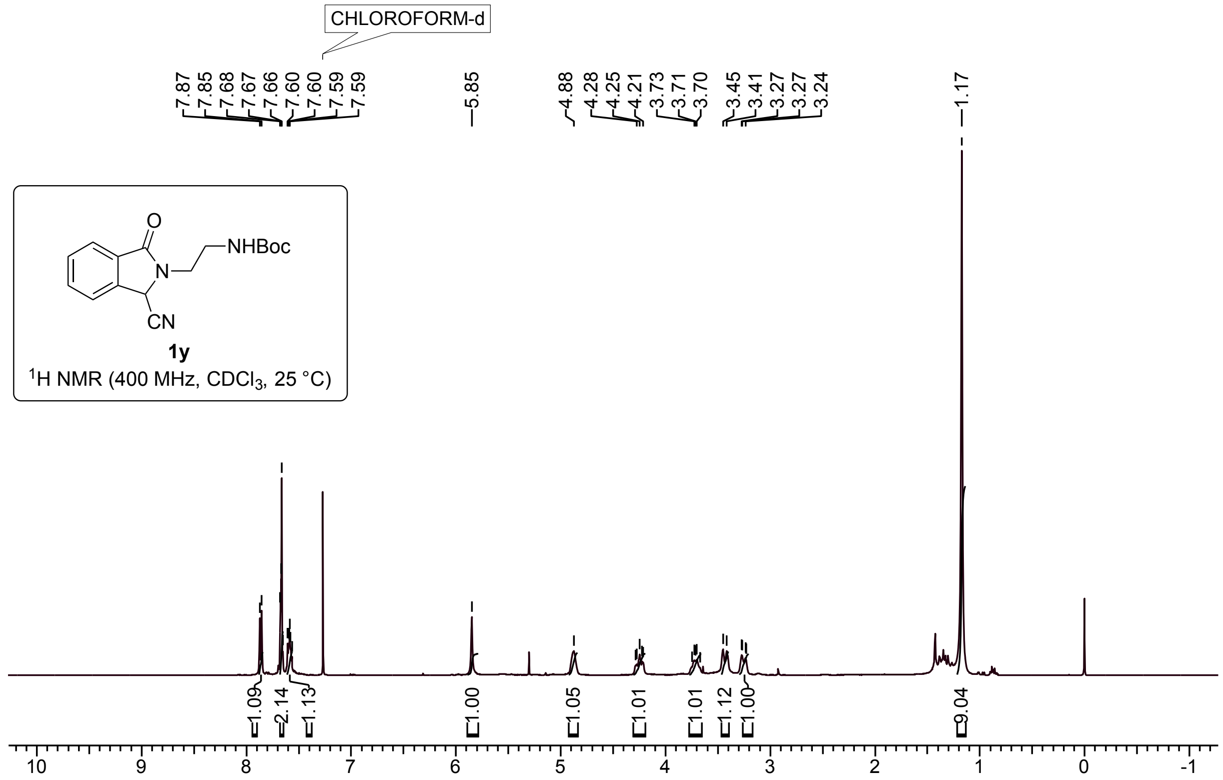
CHLOROFORM-d
7.87 7.85 7.68 7.67 7.66 7.60 7.60 7.59 7.59
5.85
4.88 4.28 4.25 4.21 3.73 3.71 3.70 3.45 3.41 3.27 3.27 3.24
1.17
O
NHBoc
N
CN
1y
¹H NMR (400 MHz, CDCl3, 25 °C)
1.09 2.14 1.13 1.00 1.05 1.01 1.01 1.12 1.00 9.04
10 9 8 7 6 5 4 3 2 1 0 -1

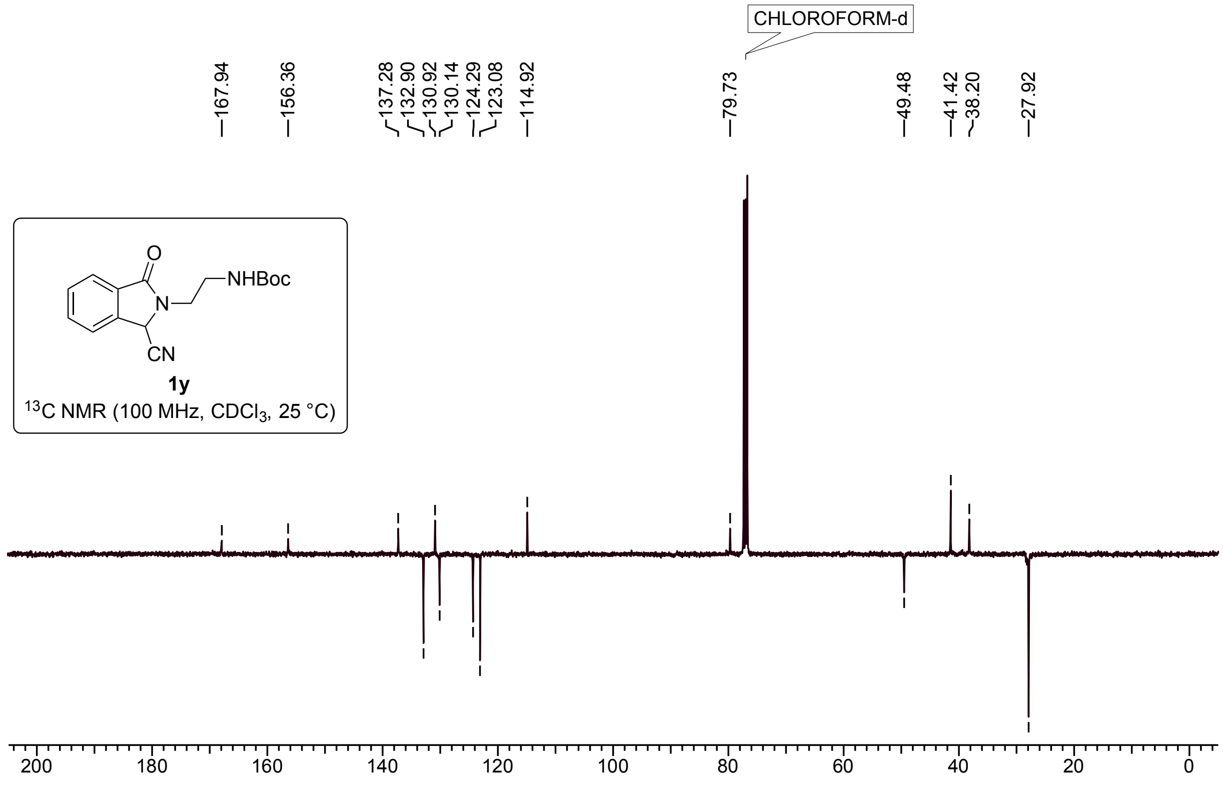
CHLOROFORM-d
167.94
156.36
137.28 132.90 130.92 130.14 124.29 123.08 114.92
79.73
49.48 41.42 38.20 27.92
O
NHBoc
N
CN
1y
¹³C NMR (100 MHz, CDCl3, 25 °C)
200 180 160 140 120 100 80 60 40 20 0

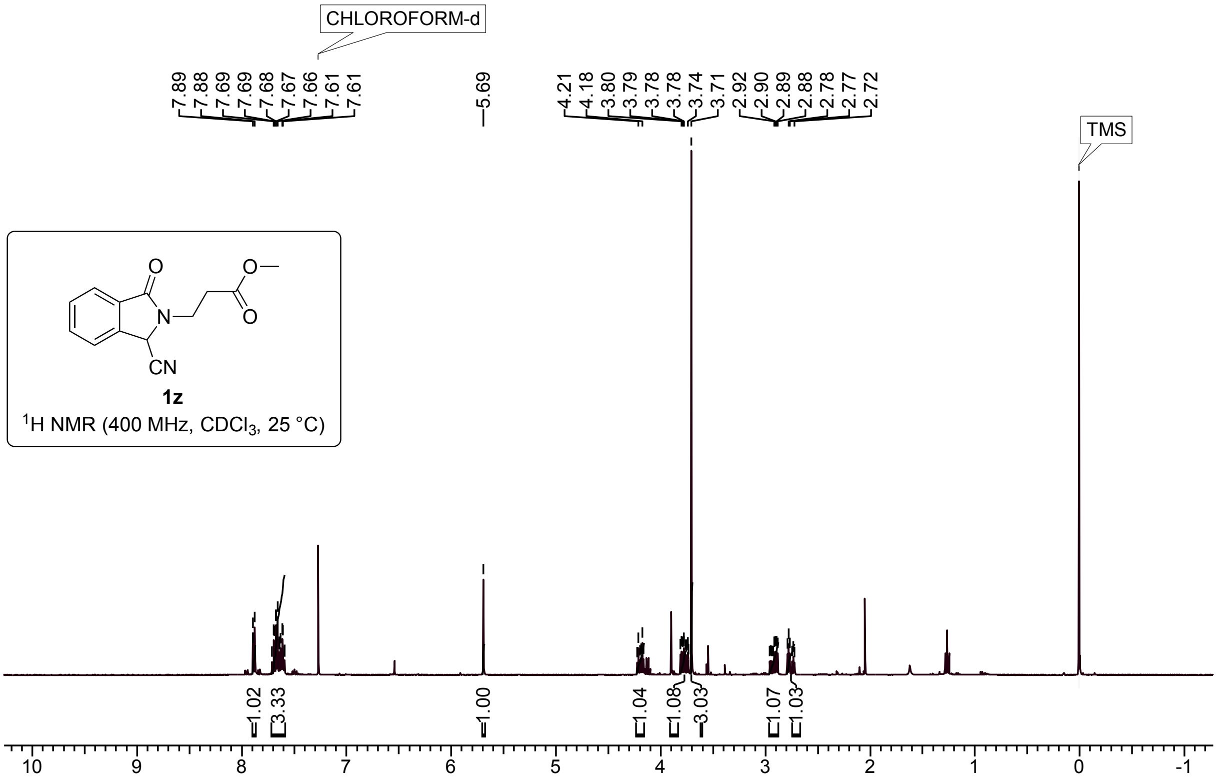
CHLOROFORM-d
7.89
7.88
7.69
7.69
7.68
7.67
7.66
7.61
7.61
5.69
4.21
4.18
3.80
3.79
3.78
3.78
3.74
3.71
2.92
2.90
2.89
2.88
2.78
2.77
2.72
TMS
O
O
N
O
CN
1z
1H NMR (400 MHz, CDCl3, 25 °C)
1.02
3.33
1.00
1.04
1.08
3.03
1.07
1.03
10
9
8
7
6
5
4
3
2
1
0
-1

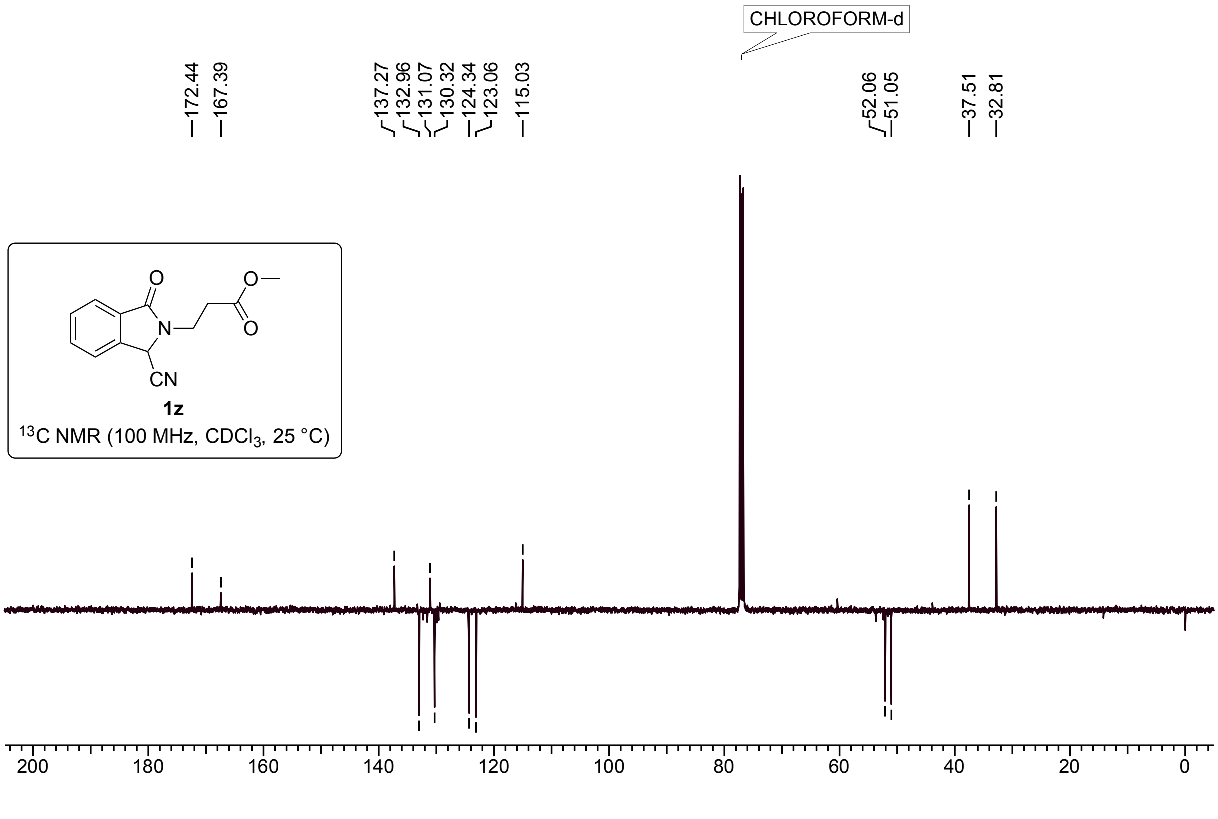
CHLOROFORM-d
172.44
167.39
137.27
132.96
131.07
130.32
124.34
123.06
115.03
52.06
51.05
37.51
32.81
O
O
N
O
CN
1z
13C NMR (100 MHz, CDCl3, 25 °C)
200
180
160
140
120
100
80
60
40
20
0

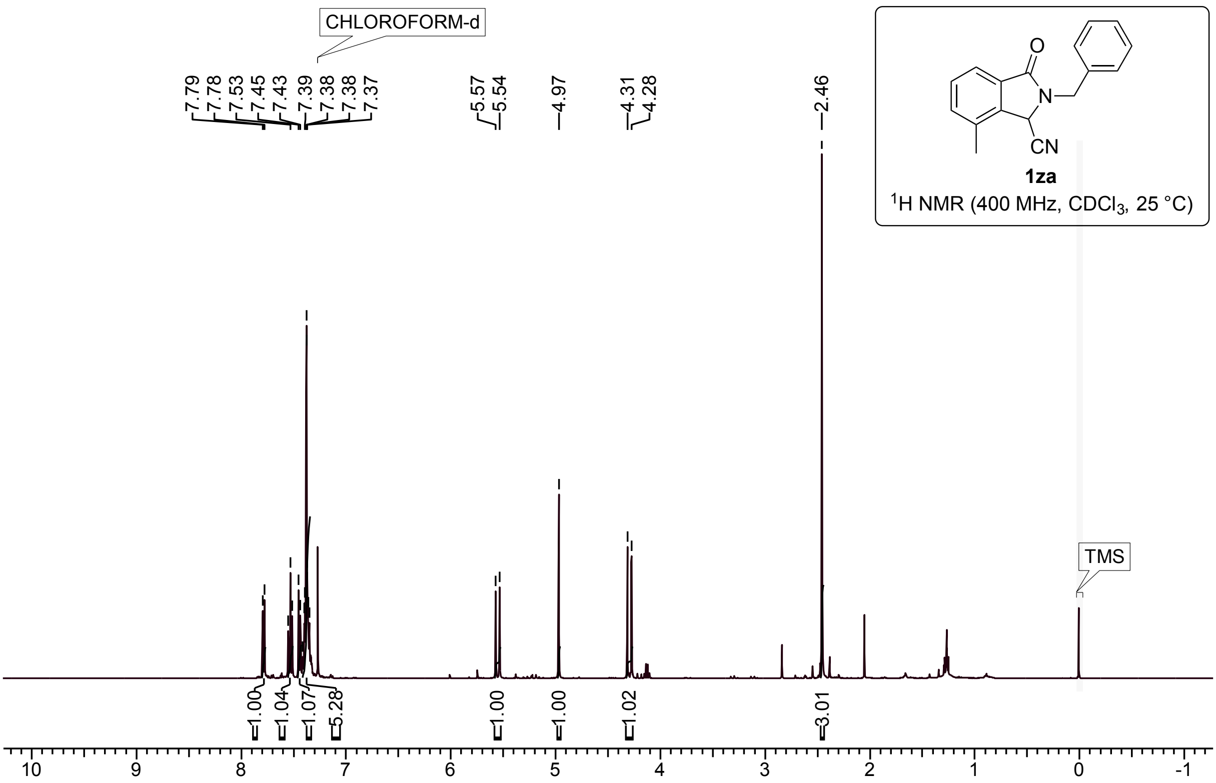
CHLOROFORM-d
7.79
7.78
7.53
7.45
7.43
7.39
7.38
7.38
7.37
5.57
5.54
4.97
4.31
4.28
2.46
TMS
1.00
1.04
1.07
5.28
1.00
1.00
1.02
3.01
1za
^{1}H NMR (400 MHz, $CDCl_3$, 25 °C)

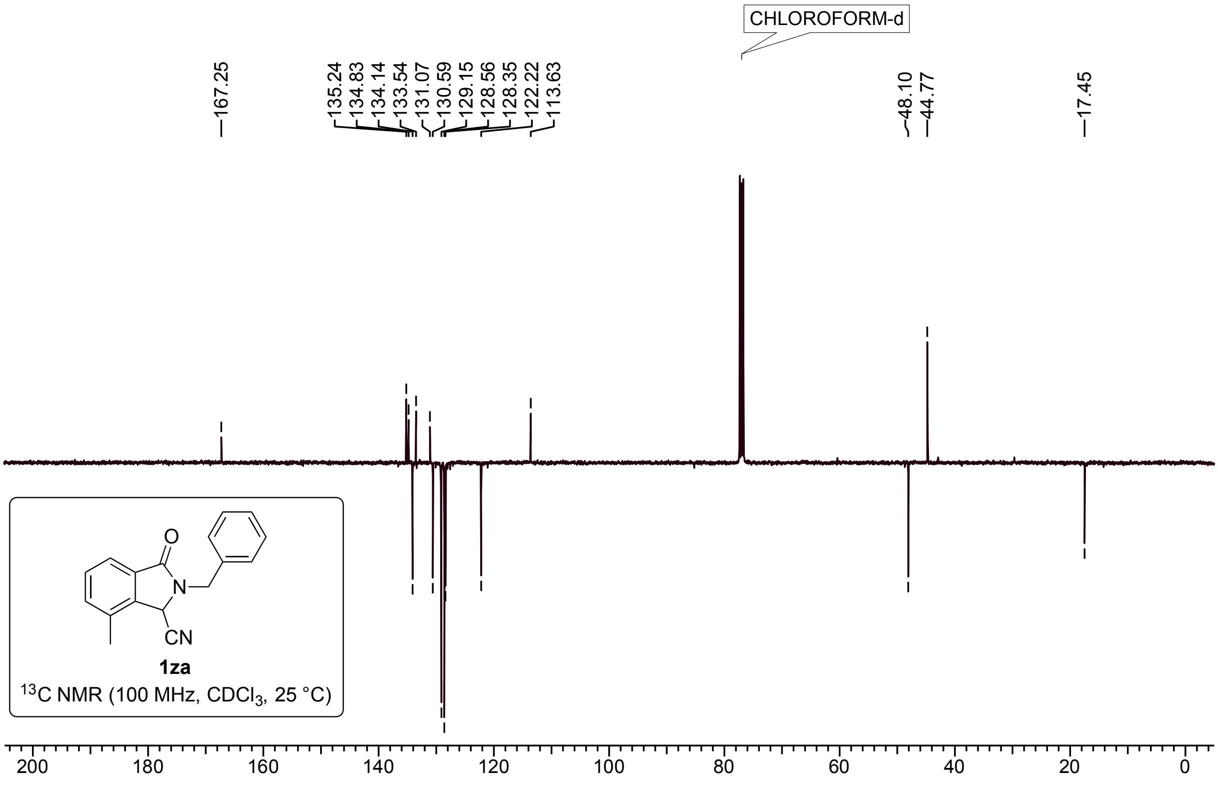
CHLOROFORM-d
167.25
135.24
134.83
134.14
133.54
131.07
130.59
129.15
128.56
128.35
122.22
113.63
48.10
44.77
17.45
1za
^{13}C NMR (100 MHz, $CDCl_3$, 25 °C)

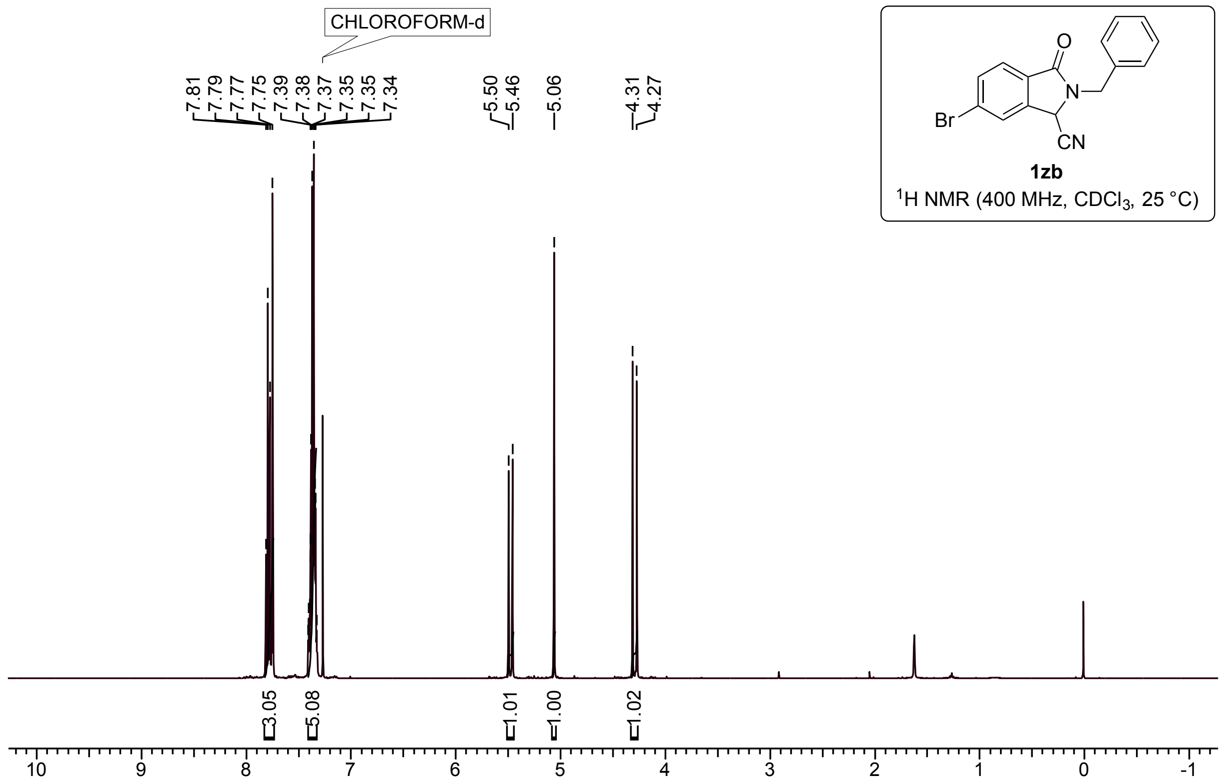
CHLOROFORM-d
7.81
7.79
7.77
7.75
7.39
7.38
7.37
7.35
7.35
7.34
5.50
5.46
5.06
4.31
4.27
O
N
Br
CN
1zb
^{1}H NMR (400 MHz, CDCl$_3$, 25 °C)
3.05
5.08
1.01
1.00
1.02
10 9 8 7 6 5 4 3 2 1 0 -1

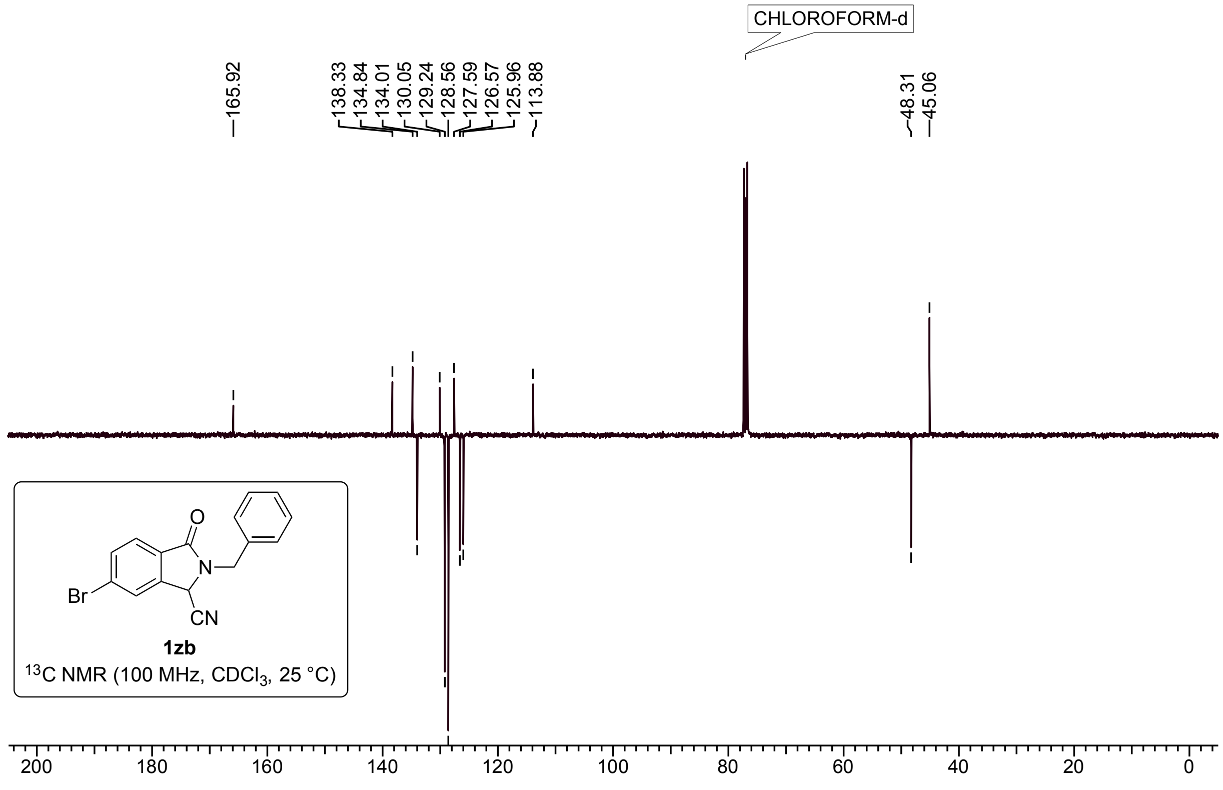
CHLOROFORM-d
165.92
138.33
134.84
134.01
130.05
129.24
128.56
127.59
126.57
125.96
113.88
48.31
45.06
O
N
Br
CN
1zb
^{13}C NMR (100 MHz, CDCl$_3$, 25 °C)
200 180 160 140 120 100 80 60 40 20 0

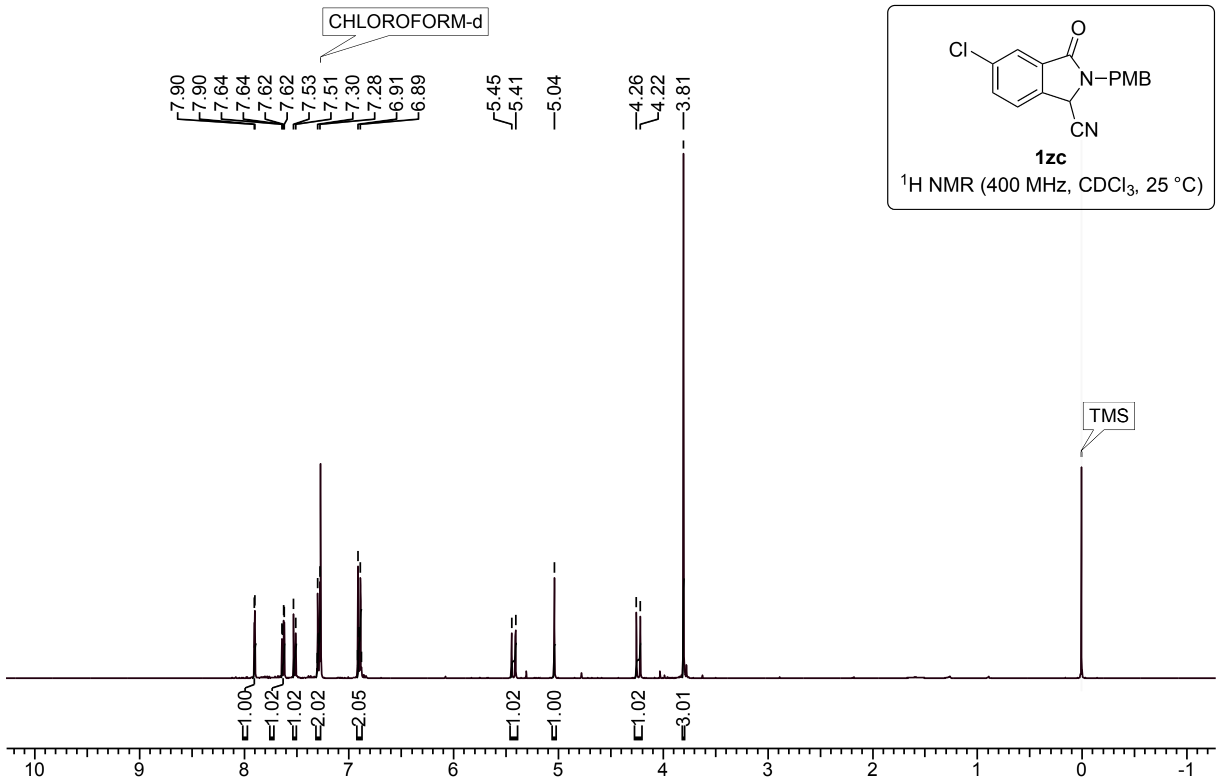

CHLOROFORM-d
7.90 7.90 7.64 7.64 7.62 7.62 7.53 7.51 7.30 7.28 6.91 6.89
5.45 5.41
5.04
4.26 4.22
3.81
TMS
Cl
O
N−PMB
CN
1zc
^{1}H NMR (400 MHz, CDCl$_3$, 25 °C)
1.00 1.02 1.02 2.02 2.05 1.02 1.00 1.02 3.01
10 9 8 7 6 5 4 3 2 1 0 -1

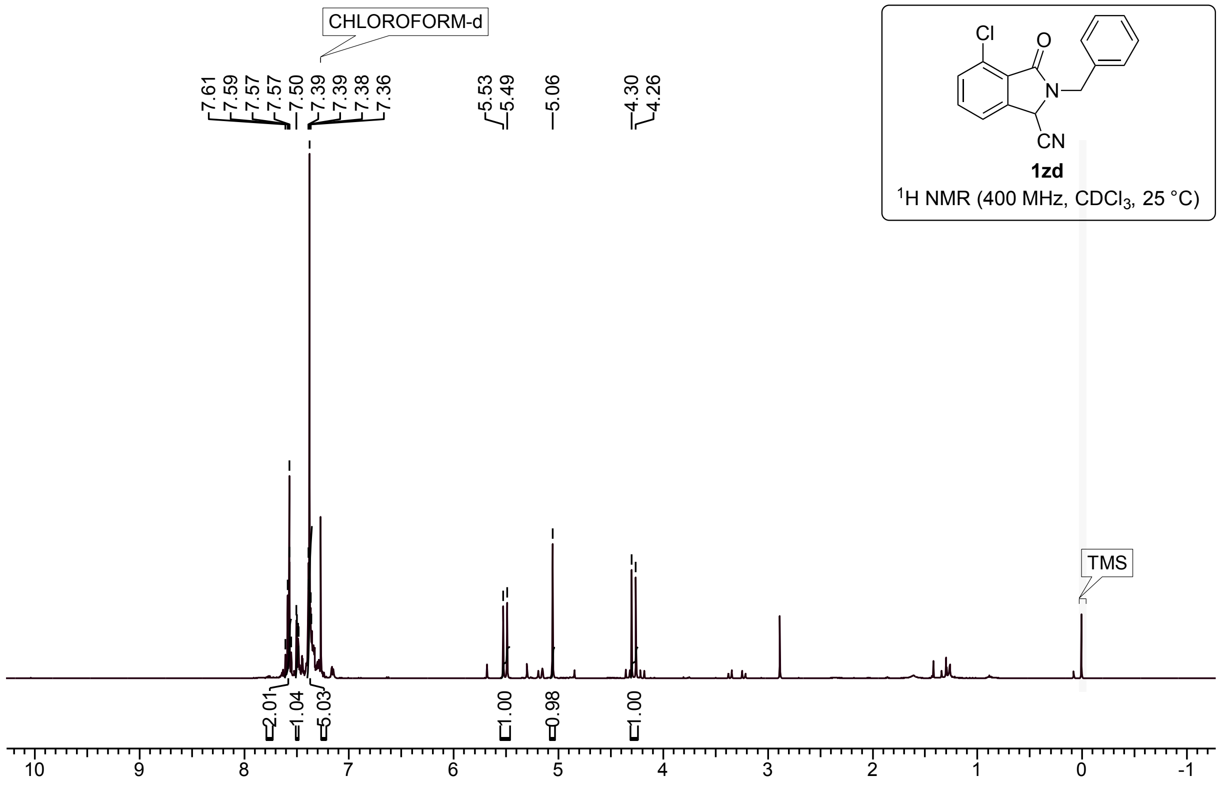

CHLOROFORM-d
7.61 7.59 7.57 7.57 7.50 7.39 7.39 7.38 7.36
5.53 5.49
5.06
4.30 4.26
TMS
Cl
O
N
CN
1zd
^{1}H NMR (400 MHz, CDCl$_3$, 25 °C)
2.01 1.04 5.03 1.00 0.98 1.00
10 9 8 7 6 5 4 3 2 1 0 -1

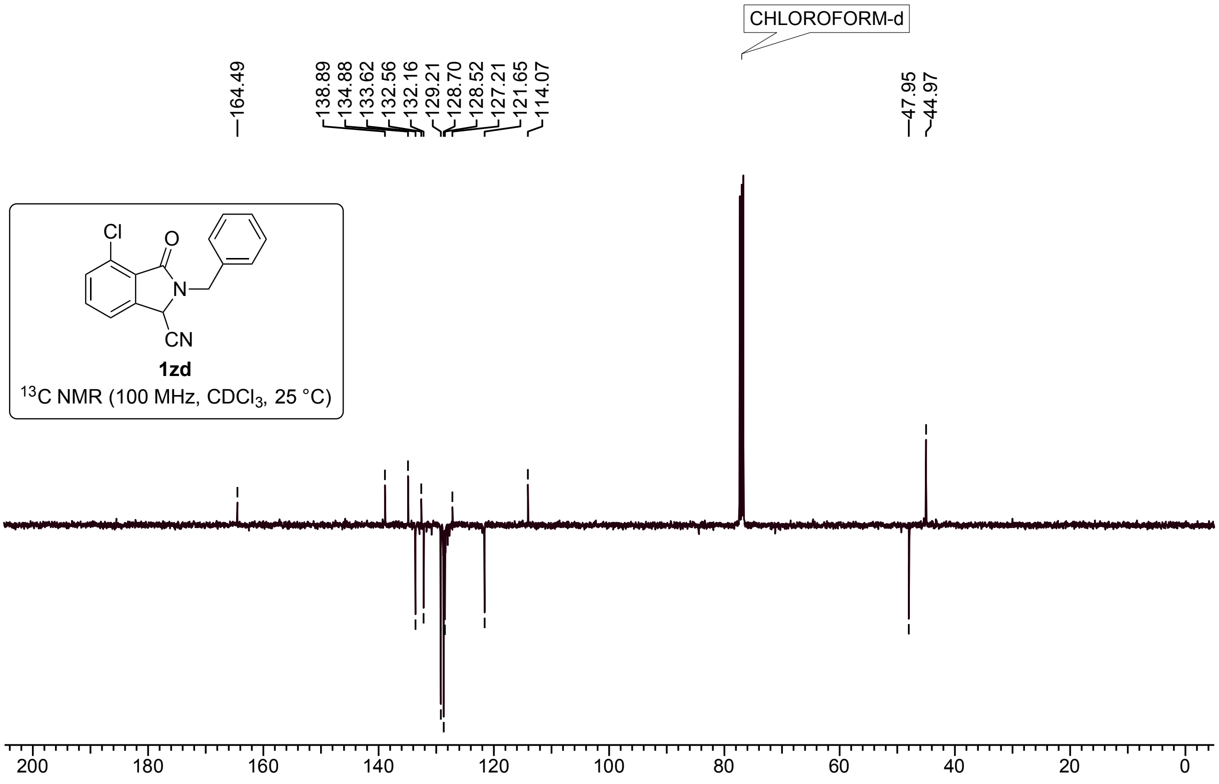
CHLOROFORM-d
164.49
138.89
134.88
133.62
132.56
132.16
129.21
128.70
128.52
127.21
121.65
114.07
47.95
44.97
Cl
O
N
CN
1zd
13C NMR (100 MHz, CDCl3, 25 °C)
200
180
160
140
120
100
80
60
40
20
0

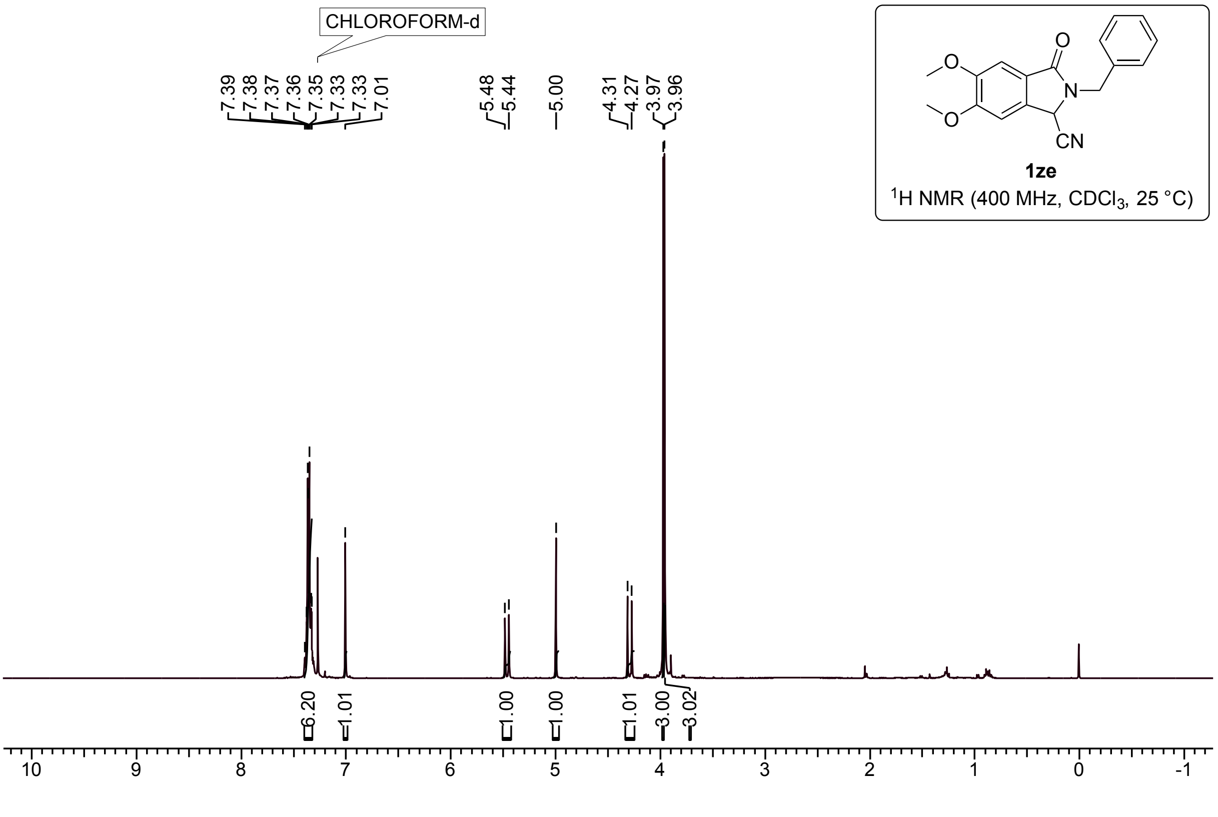
CHLOROFORM-d
7.39
7.38
7.37
7.36
7.35
7.33
7.33
7.01
5.48
5.44
5.00
4.31
4.27
3.97
3.96
O
O
O
N
CN
1ze
1H NMR (400 MHz, CDCl3, 25 °C)
6.20
1.01
1.00
1.00
1.01
3.00
3.02
10
9
8
7
6
5
4
3
2
1
0
-1

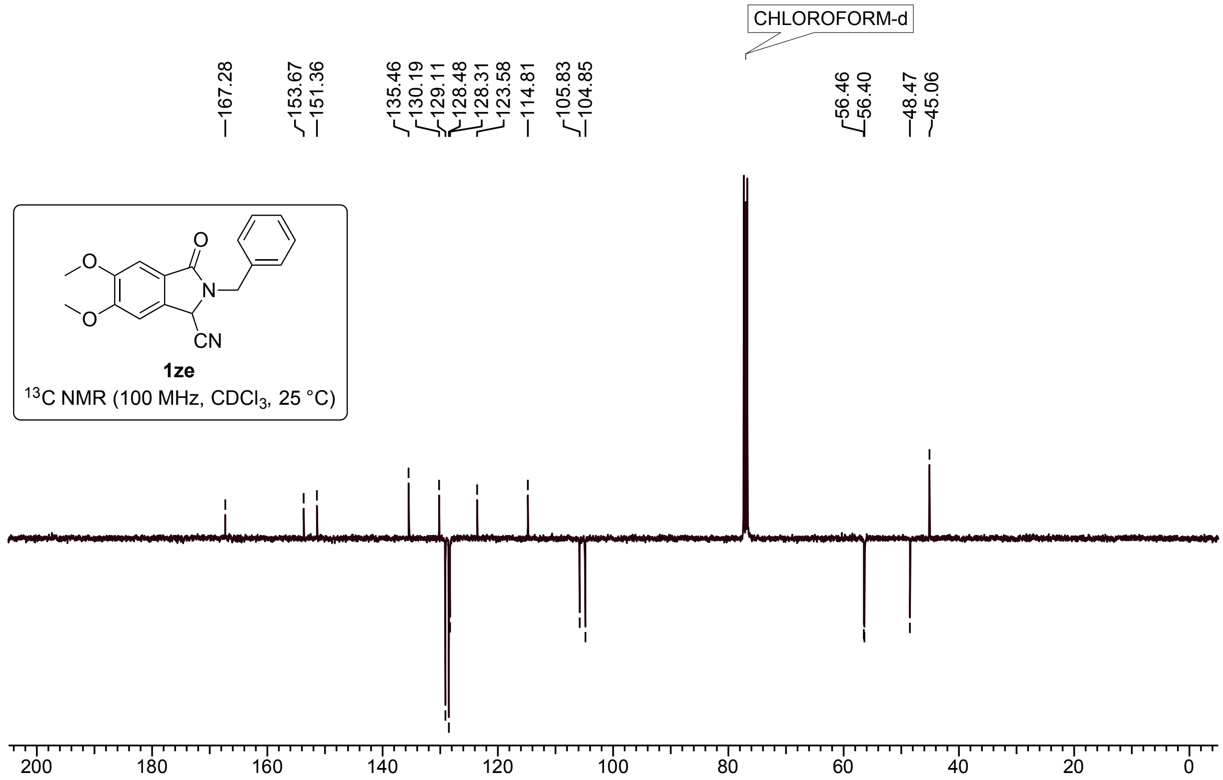
CHLOROFORM-d
167.28
153.67
151.36
135.46
130.19
129.11
128.48
128.31
123.58
114.81
105.83
104.85
56.46
56.40
48.47
45.06
O
N
O
O
CN
1ze
^{13}C NMR (100 MHz, CDCl$_3$, 25 °C)
200
180
160
140
120
100
80
60
40
20
0

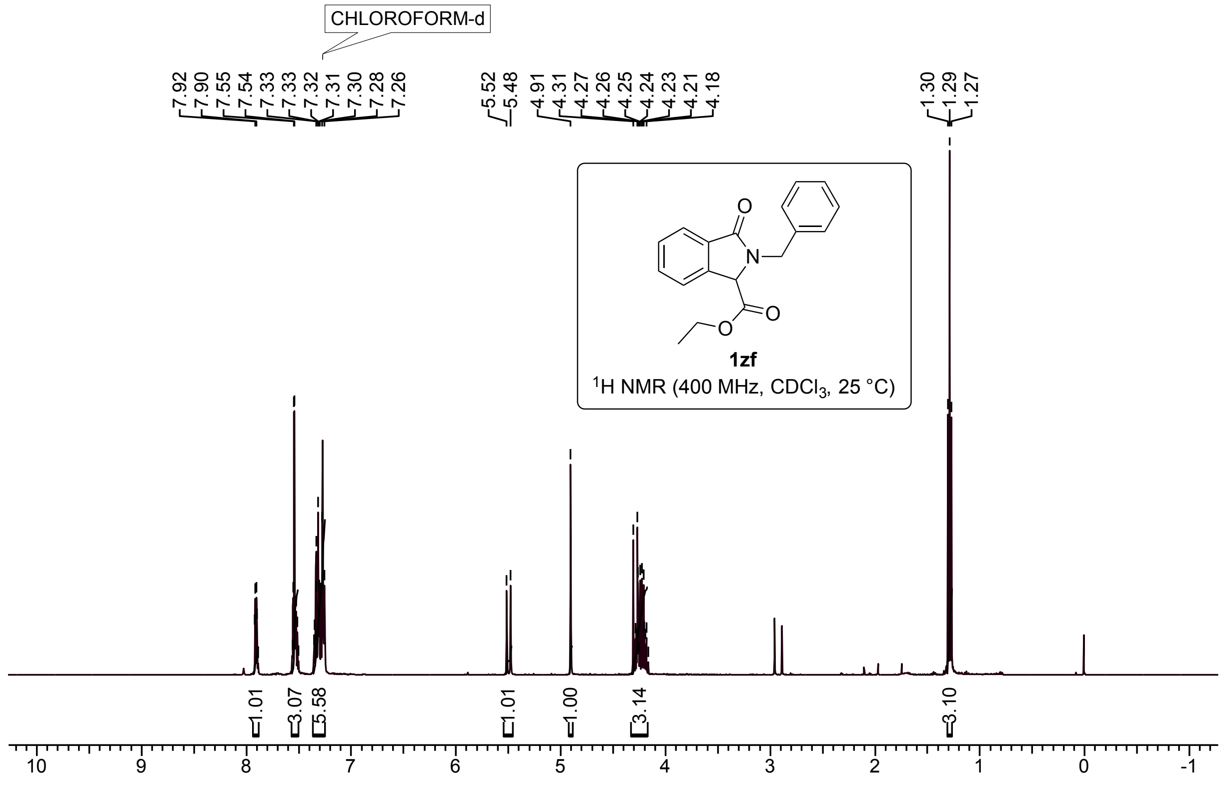
CHLOROFORM-d
7.92
7.90
7.55
7.54
7.33
7.33
7.32
7.31
7.30
7.28
7.26
5.52
5.48
4.91
4.31
4.27
4.26
4.25
4.24
4.23
4.21
4.18
1.30
1.29
1.27
O
N
O
O
1zf
^{1}H NMR (400 MHz, CDCl$_3$, 25 °C)
1.01
3.07
5.58
1.01
1.00
3.14
3.10
10
9
8
7
6
5
4
3
2
1
0
-1

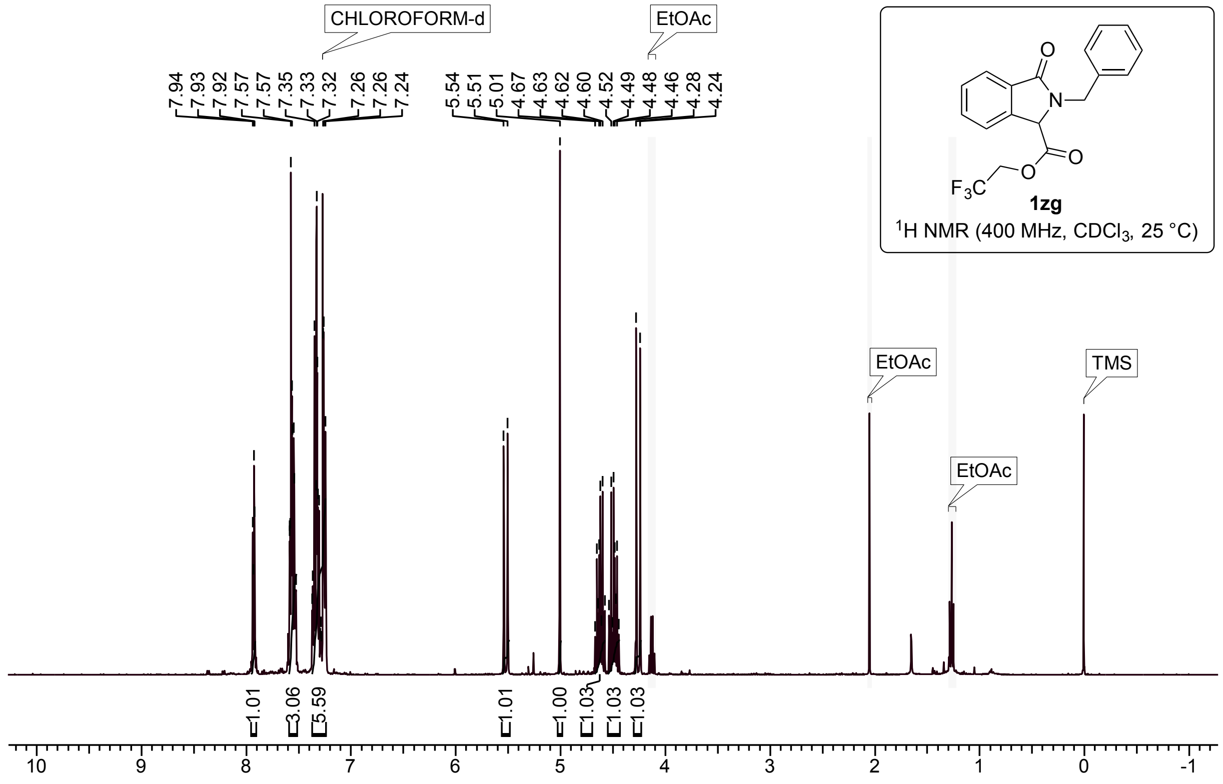
CHLOROFORM-d
EtOAc
7.94
7.93
7.92
7.57
7.57
7.35
7.33
7.32
7.26
7.26
7.24
5.54
5.51
5.01
4.67
4.63
4.62
4.60
4.52
4.49
4.48
4.46
4.28
4.24
1zg
^{1}H NMR (400 MHz, CDCl$_3$, 25 °C)
EtOAc
TMS
EtOAc
1.01
3.06
5.59
1.01
1.00
1.03
1.03
1.03
10
9
8
7
6
5
4
3
2
1
0
-1

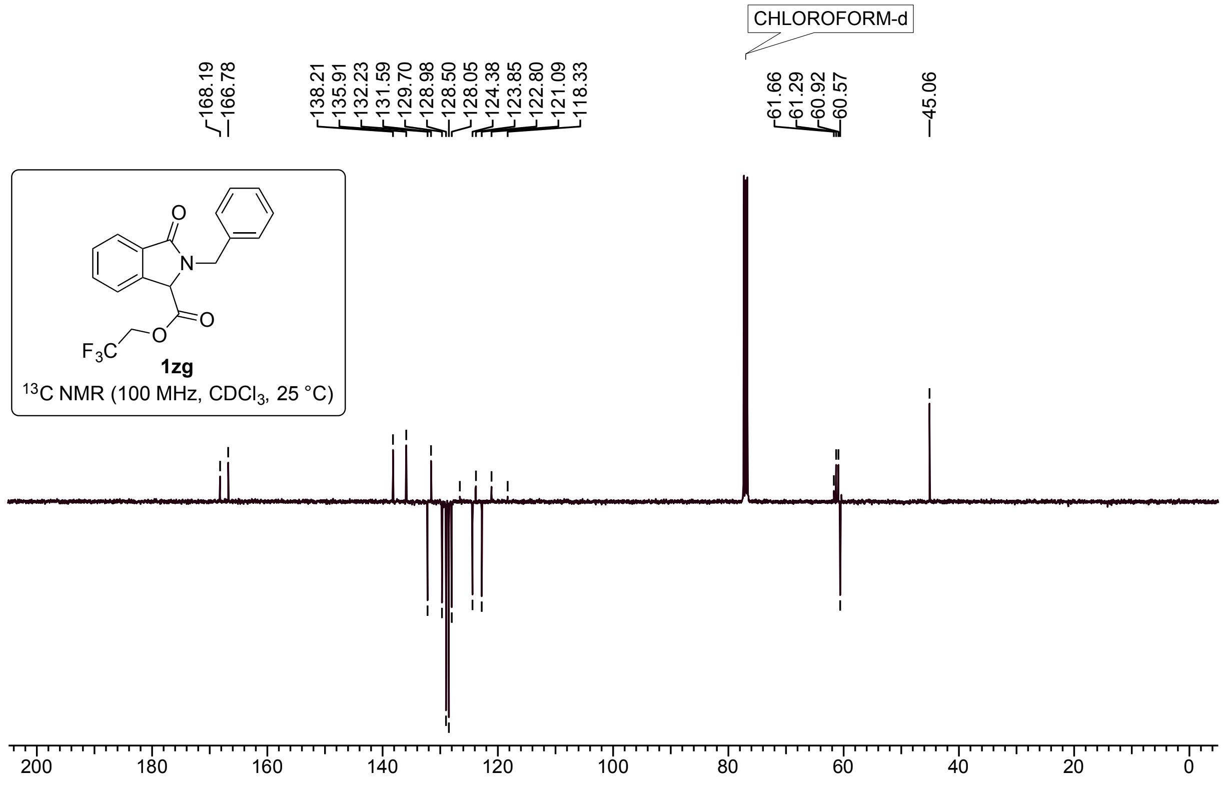
CHLOROFORM-d
168.19
166.78
138.21
135.91
132.23
131.59
129.70
128.98
128.50
128.05
124.38
123.85
122.80
121.09
118.33
61.66
61.29
60.92
60.57
45.06
1zg
^{13}C NMR (100 MHz, CDCl$_3$, 25 °C)
200
180
160
140
120
100
80
60
40
20
0

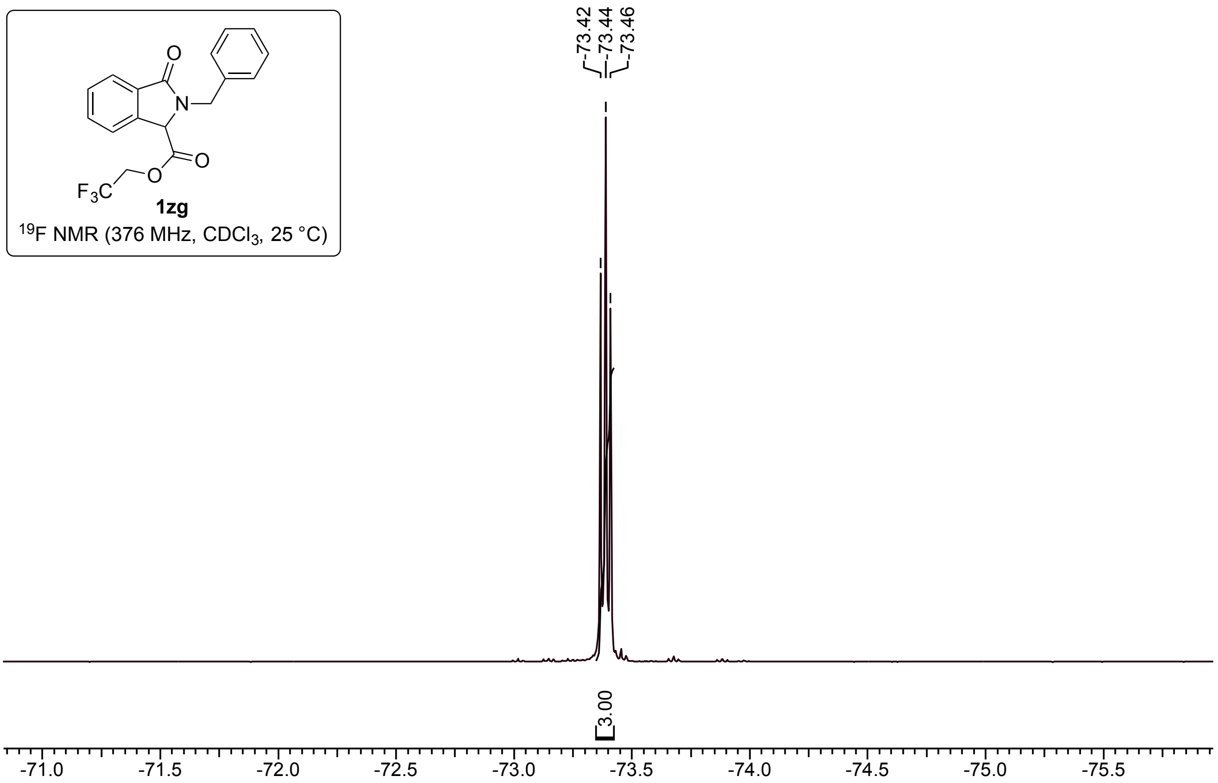
-73.42
-73.44
-73.46
1zg
^{19}F NMR (376 MHz, CDCl$_3$, 25 °C)
3.00
-71.0
-71.5
-72.0
-72.5
-73.0
-73.5
-74.0
-74.5
-75.0
-75.5

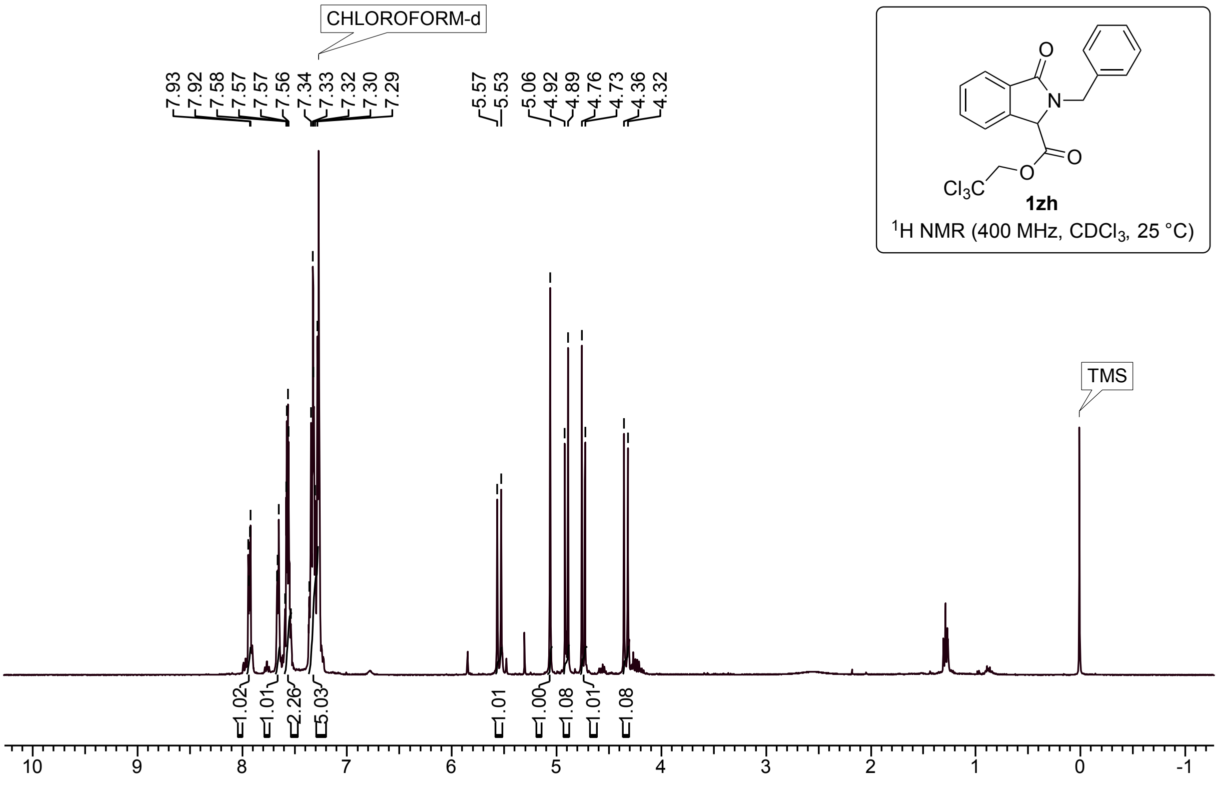
CHLOROFORM-d
7.93
7.92
7.58
7.57
7.57
7.56
7.34
7.33
7.32
7.30
7.29
5.57
5.53
5.06
4.92
4.89
4.76
4.73
4.36
4.32
1zh
^{1}H NMR (400 MHz, CDCl$_3$, 25 °C)
TMS
1.02
1.01
2.26
5.03
1.01
1.00
1.08
1.01
1.08
10
9
8
7
6
5
4
3
2
1
0
-1

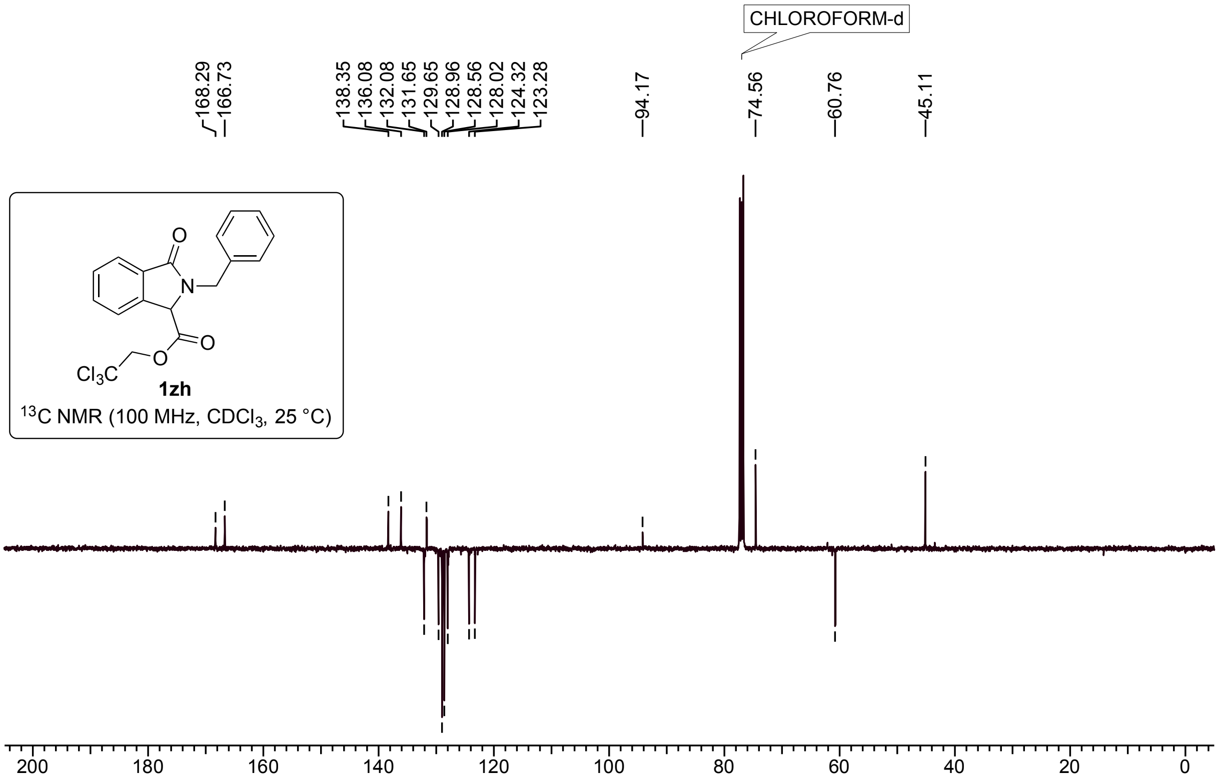
CHLOROFORM-d
168.29
166.73
138.35
136.08
132.08
131.65
129.65
128.96
128.56
128.02
124.32
123.28
94.17
74.56
60.76
45.11
O
N
O
O
Cl3C
1zh
13C NMR (100 MHz, CDCl3, 25 °C)
200
180
160
140
120
100
80
60
40
20
0

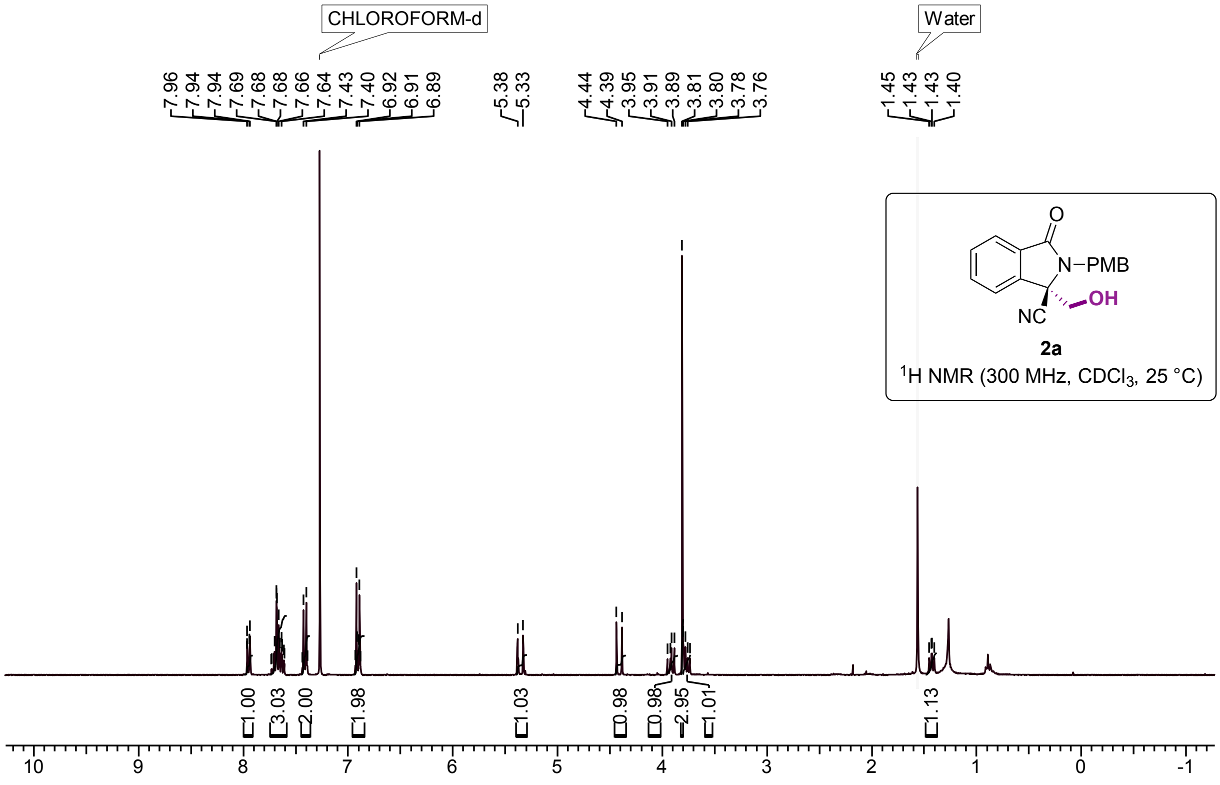
CHLOROFORM-d
Water
7.96
7.94
7.94
7.69
7.68
7.66
7.64
7.43
7.40
6.92
6.91
6.89
5.38
5.33
4.44
4.39
3.95
3.91
3.89
3.81
3.80
3.78
3.76
1.45
1.43
1.43
1.40
O
N−PMB
OH
NC
2a
1H NMR (300 MHz, CDCl3, 25 °C)
1.00
3.03
2.00
1.98
1.03
0.98
0.98
2.95
1.01
1.13
10
9
8
7
6
5
4
3
2
1
0
-1

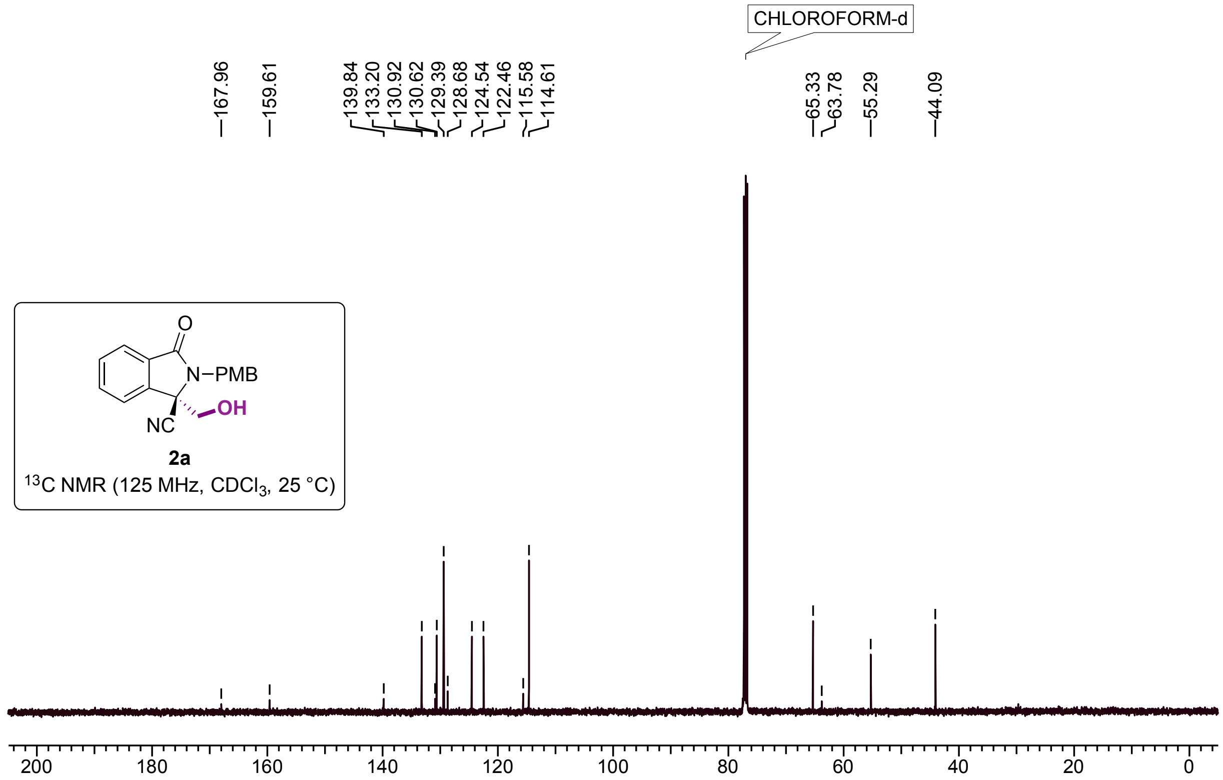

CHLOROFORM-d
167.96
159.61
139.84
133.20
130.92
130.62
129.39
128.68
124.54
122.46
115.58
114.61
65.33
63.78
55.29
44.09
O
N−PMB
OH
NC
2a
13C NMR (125 MHz, CDCl3, 25 °C)
200
180
160
140
120
100
80
60
40
20
0

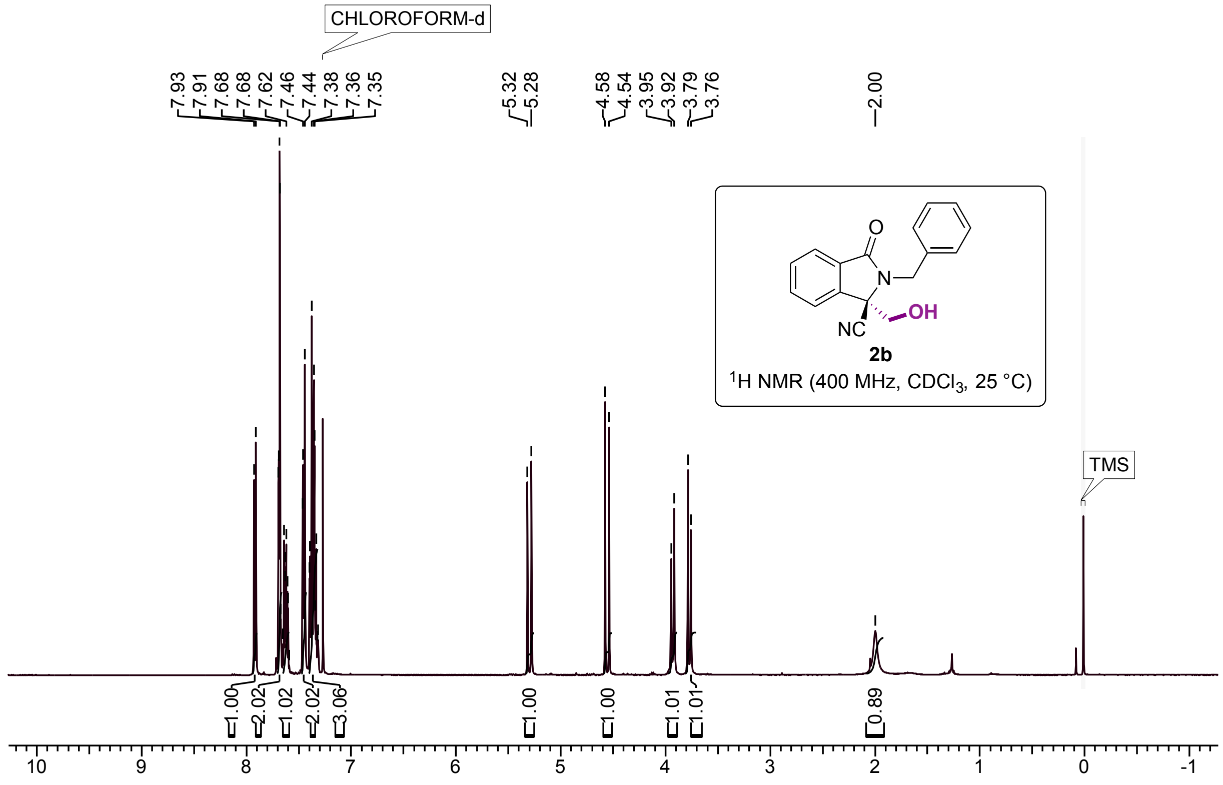

CHLOROFORM-d
7.93
7.91
7.68
7.68
7.62
7.46
7.44
7.38
7.36
7.35
5.32
5.28
4.58
4.54
3.95
3.92
3.79
3.76
2.00
O
N
OH
NC
2b
1H NMR (400 MHz, CDCl3, 25 °C)
TMS
1.00
2.02
1.02
2.02
3.06
1.00
1.00
1.01
1.01
0.89
10
9
8
7
6
5
4
3
2
1
0
-1

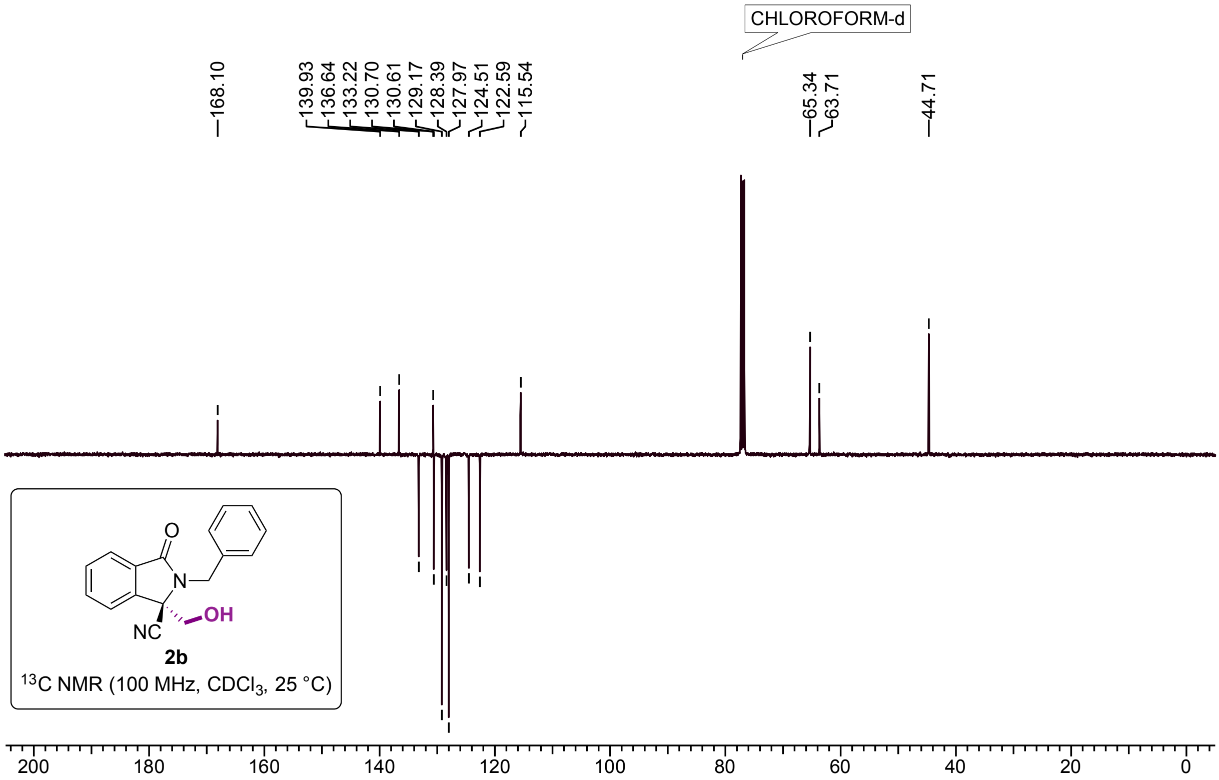
CHLOROFORM-d
168.10
139.93
136.64
133.22
130.70
130.61
129.17
128.39
127.97
124.51
122.59
115.54
65.34
63.71
44.71
O
N
OH
NC
2b
^{13}C NMR (100 MHz, $CDCl_3$, 25 °C)
200
180
160
140
120
100
80
60
40
20
0

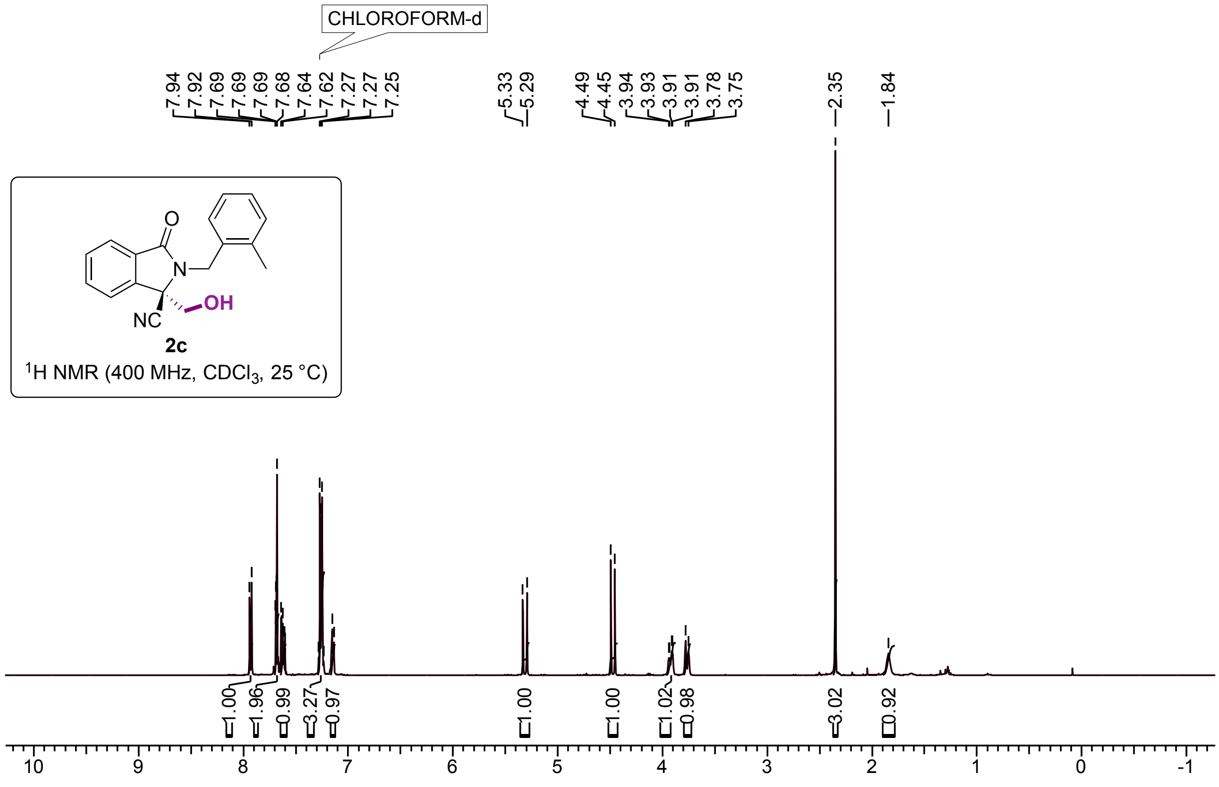
CHLOROFORM-d
7.94
7.92
7.69
7.69
7.69
7.68
7.64
7.62
7.27
7.27
7.25
5.33
5.29
4.49
4.45
3.94
3.93
3.91
3.91
3.78
3.75
2.35
1.84
O
N
OH
NC
2c
^{1}H NMR (400 MHz, $CDCl_3$, 25 °C)
1.00
1.96
0.99
3.27
0.97
1.00
1.00
1.02
0.98
3.02
0.92
10
9
8
7
6
5
4
3
2
1
0
-1

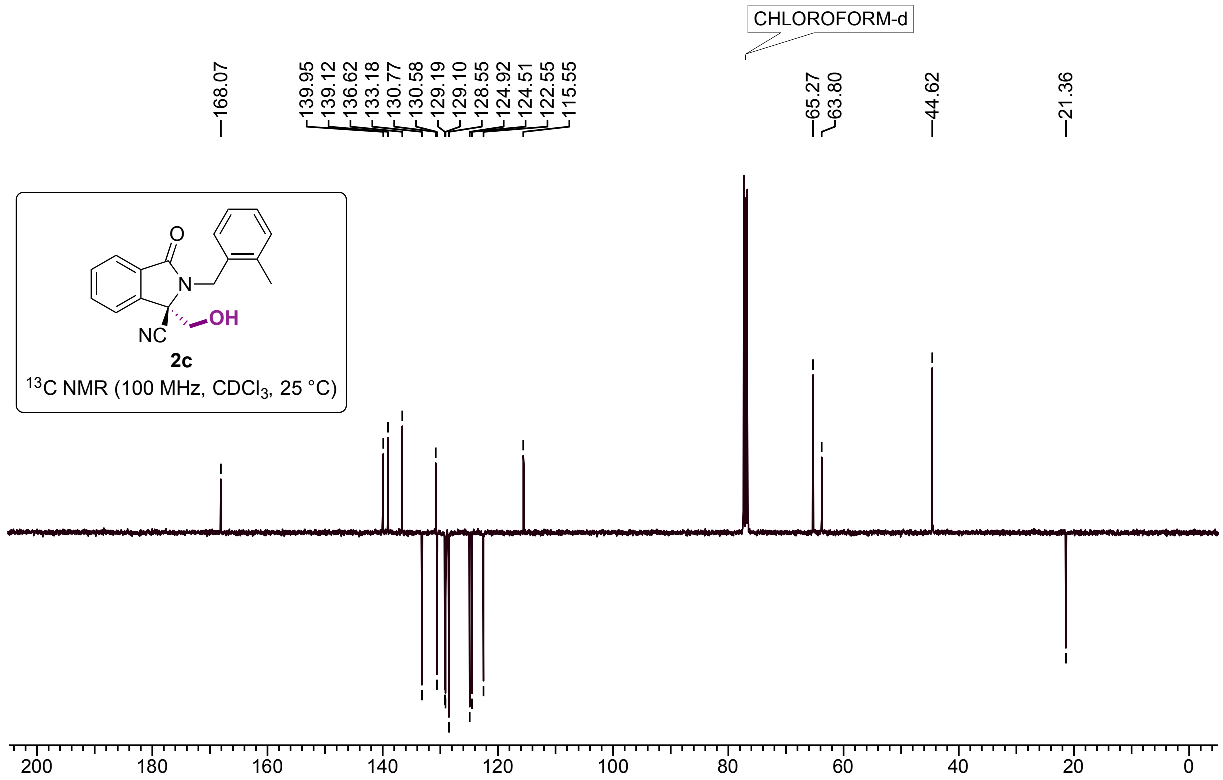
CHLOROFORM-d
168.07
139.95
139.12
136.62
133.18
130.77
130.58
129.19
129.10
128.55
124.92
124.51
122.55
115.55
65.27
63.80
44.62
21.36
2c
^{13}C NMR (100 MHz, CDCl$_3$, 25 °C)
O
N
NC
OH

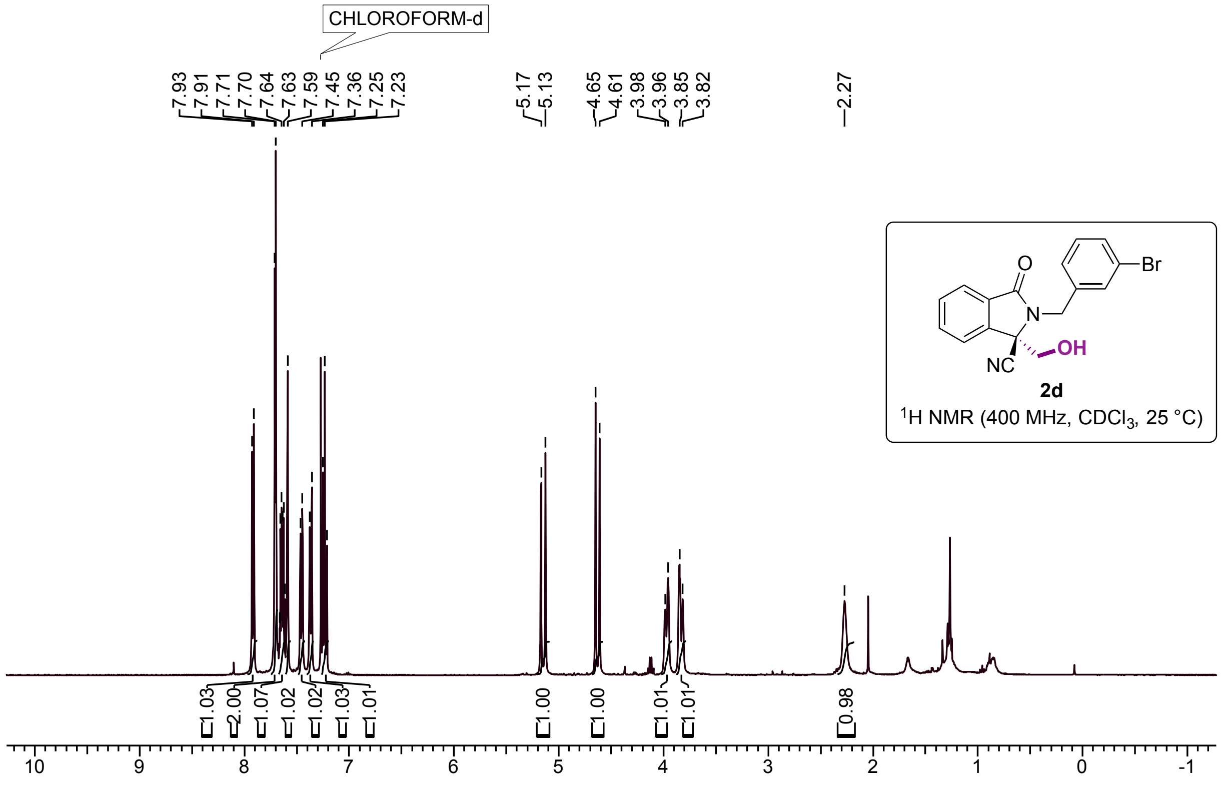
CHLOROFORM-d
7.93
7.91
7.71
7.70
7.64
7.63
7.59
7.45
7.36
7.25
7.23
5.17
5.13
4.65
4.61
3.98
3.96
3.85
3.82
2.27
2d
^{1}H NMR (400 MHz, CDCl$_3$, 25 °C)
O
N
Br
NC
OH
1.03
2.00
1.07
1.02
1.02
1.03
1.01
1.00
1.00
1.01
1.01
0.98

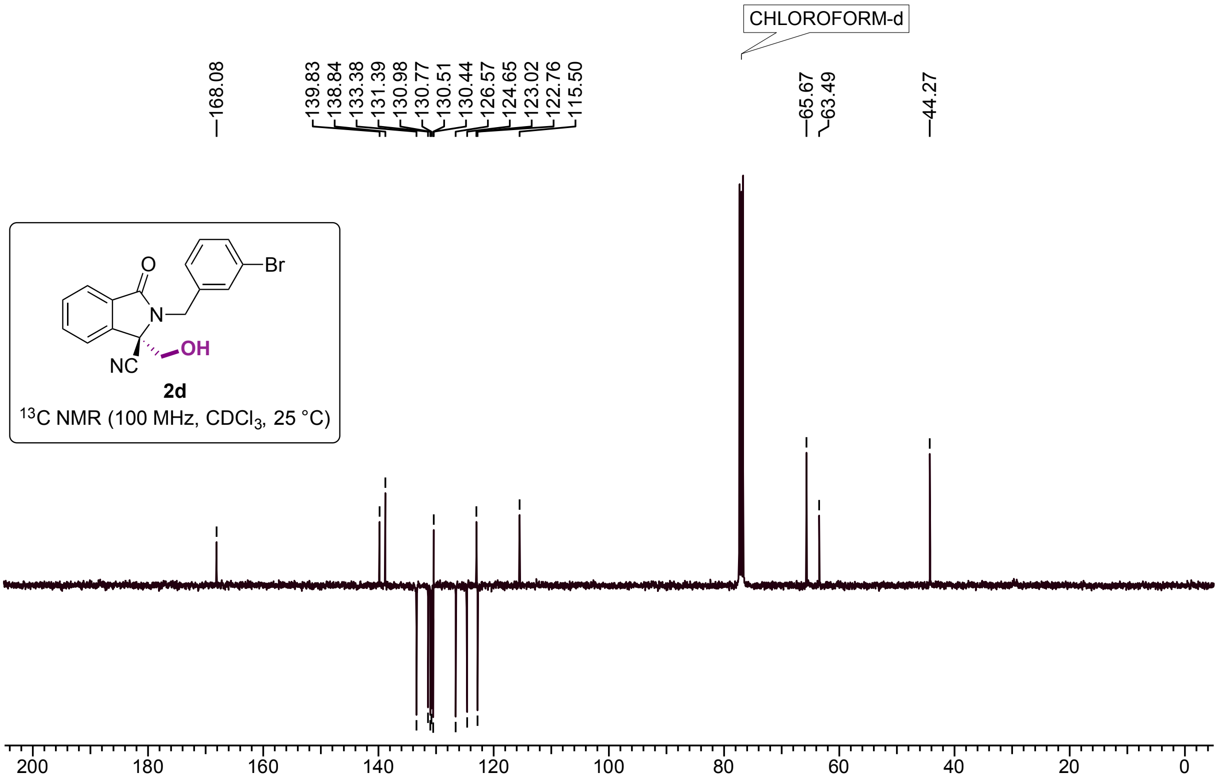
CHLOROFORM-d
168.08
139.83
138.84
133.38
131.39
130.98
130.77
130.51
130.44
126.57
124.65
123.02
122.76
115.50
65.67
63.49
44.27
O
Br
N
OH
NC
2d
^{13}C NMR (100 MHz, CDCl$_3$, 25 °C)
200
180
160
140
120
100
80
60
40
20
0

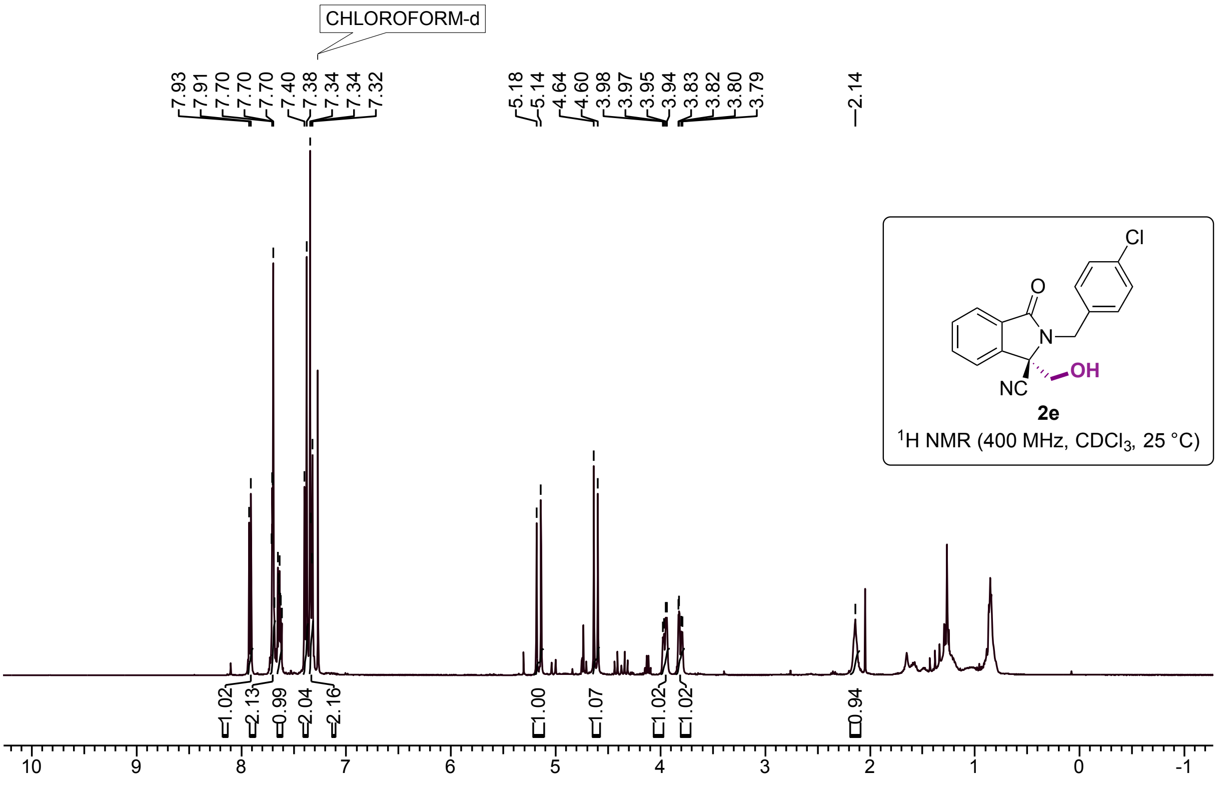
CHLOROFORM-d
7.93
7.91
7.70
7.70
7.70
7.40
7.38
7.34
7.34
7.32
5.18
5.14
4.64
4.60
3.98
3.97
3.95
3.94
3.83
3.82
3.80
3.79
2.14
Cl
O
N
OH
NC
2e
^{1}H NMR (400 MHz, CDCl$_3$, 25 °C)
1.02
2.13
0.99
2.04
2.16
1.00
1.07
1.02
1.02
0.94
10
9
8
7
6
5
4
3
2
1
0
-1

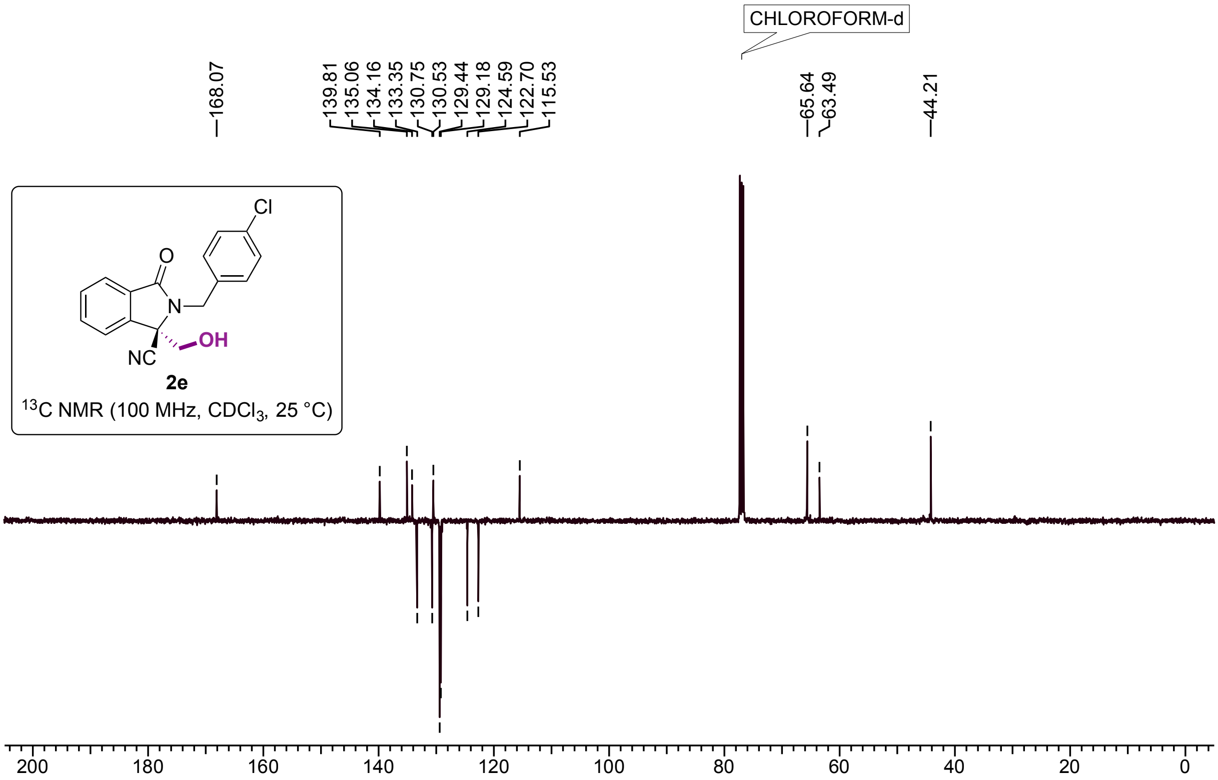
CHLOROFORM-d
168.07
139.81
135.06
134.16
133.35
130.75
130.53
129.44
129.18
124.59
122.70
115.53
65.64
63.49
44.21
Cl
O
N
OH
NC
2e
^{13}C NMR (100 MHz, CDCl$_3$, 25 °C)
200 180 160 140 120 100 80 60 40 20 0

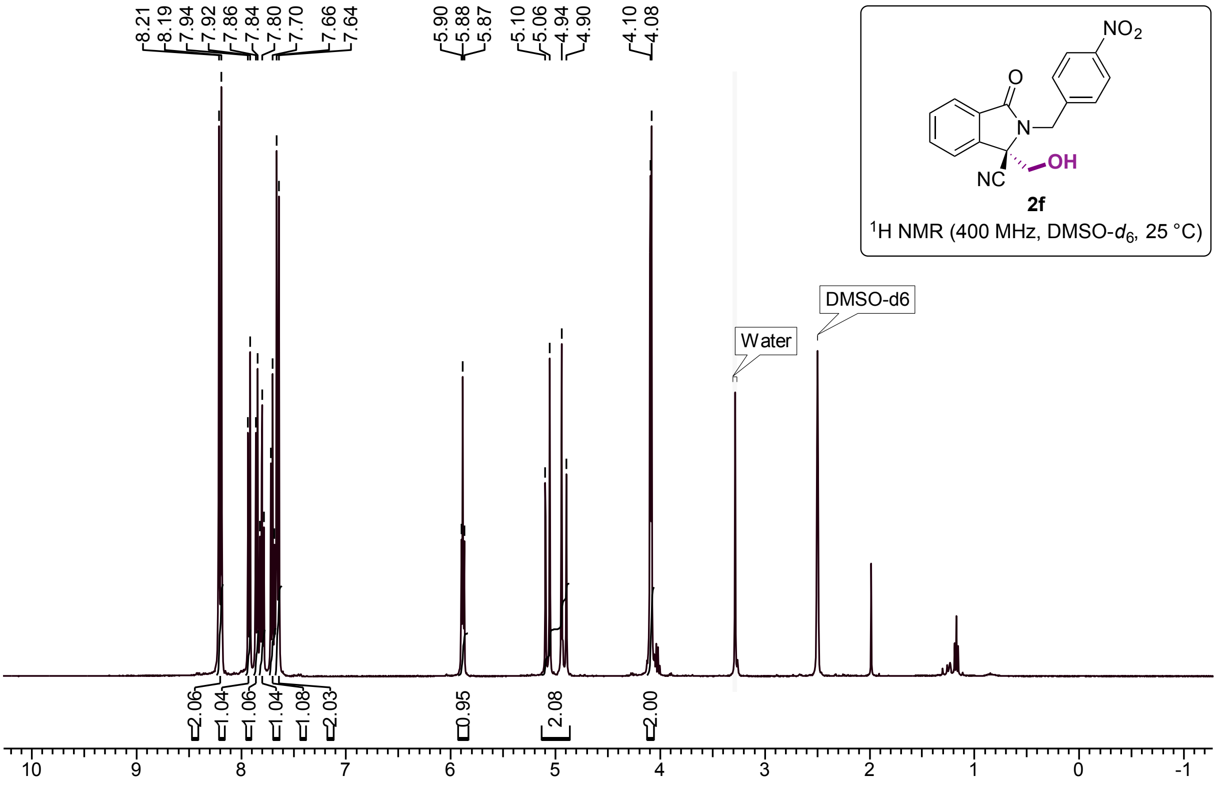
8.21
8.19
7.94
7.92
7.86
7.84
7.80
7.70
7.66
7.64
5.90
5.88
5.87
5.10
5.06
4.94
4.90
4.10
4.08
NO_2
O
N
OH
NC
2f
^{1}H NMR (400 MHz, DMSO-d_6, 25 °C)
DMSO-d6
Water
2.06
1.04
1.06
1.04
1.08
2.03
0.95
2.08
2.00
10 9 8 7 6 5 4 3 2 1 0 -1

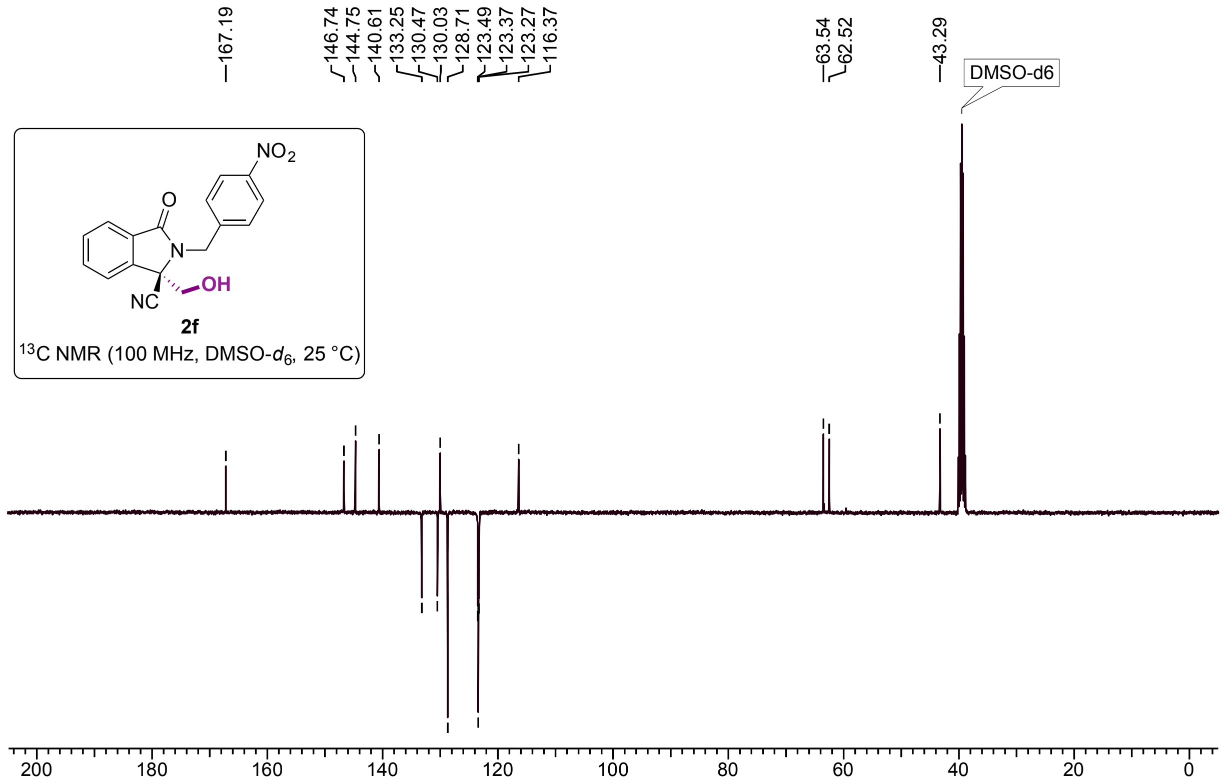
167.19
146.74
144.75
140.61
133.25
130.47
130.03
128.71
123.49
123.37
123.27
116.37
63.54
62.52
43.29
DMSO-d6
NO2
O
N
OH
NC
2f
13C NMR (100 MHz, DMSO-d6, 25 °C)
200
180
160
140
120
100
80
60
40
20
0

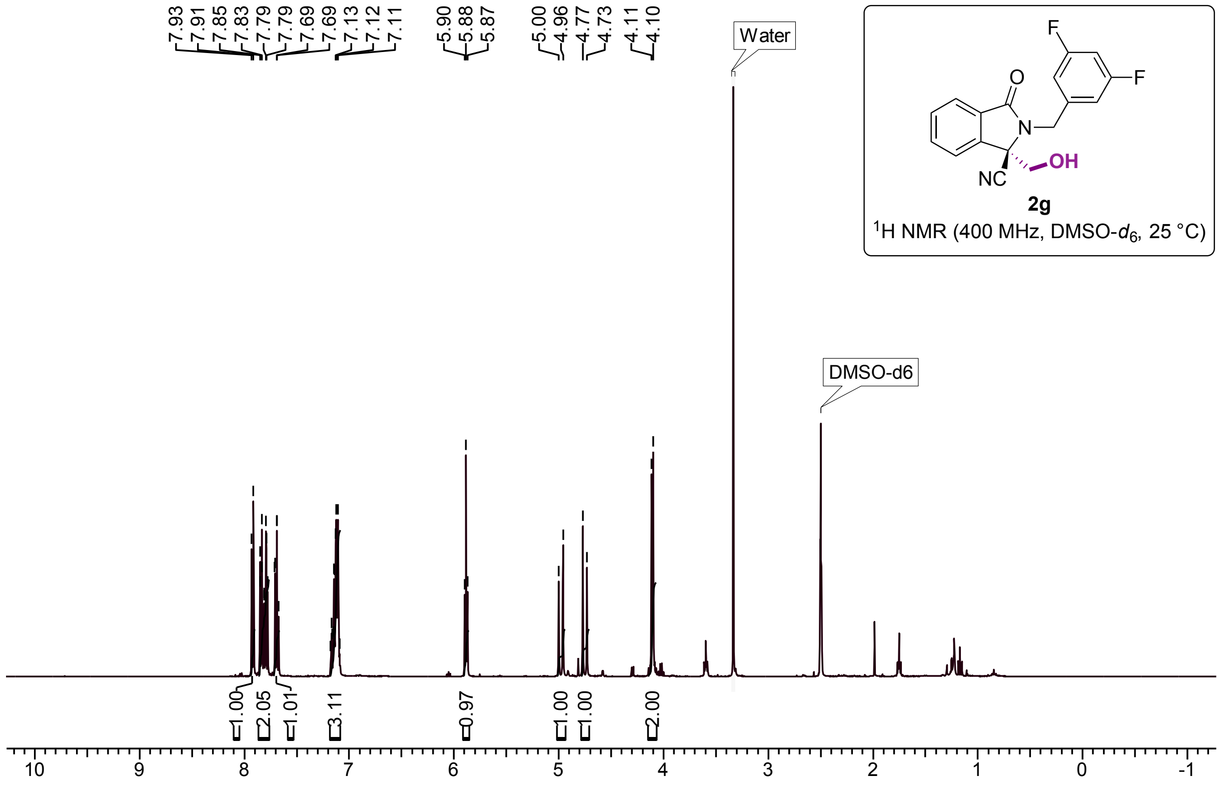
7.93
7.91
7.85
7.83
7.79
7.79
7.69
7.69
7.13
7.12
7.11
5.90
5.88
5.87
5.00
4.96
4.77
4.73
4.11
4.10
Water
F
F
O
N
OH
NC
2g
1H NMR (400 MHz, DMSO-d6, 25 °C)
DMSO-d6
1.00
2.05
1.01
3.11
0.97
1.00
1.00
2.00
10
9
8
7
6
5
4
3
2
1
0
-1

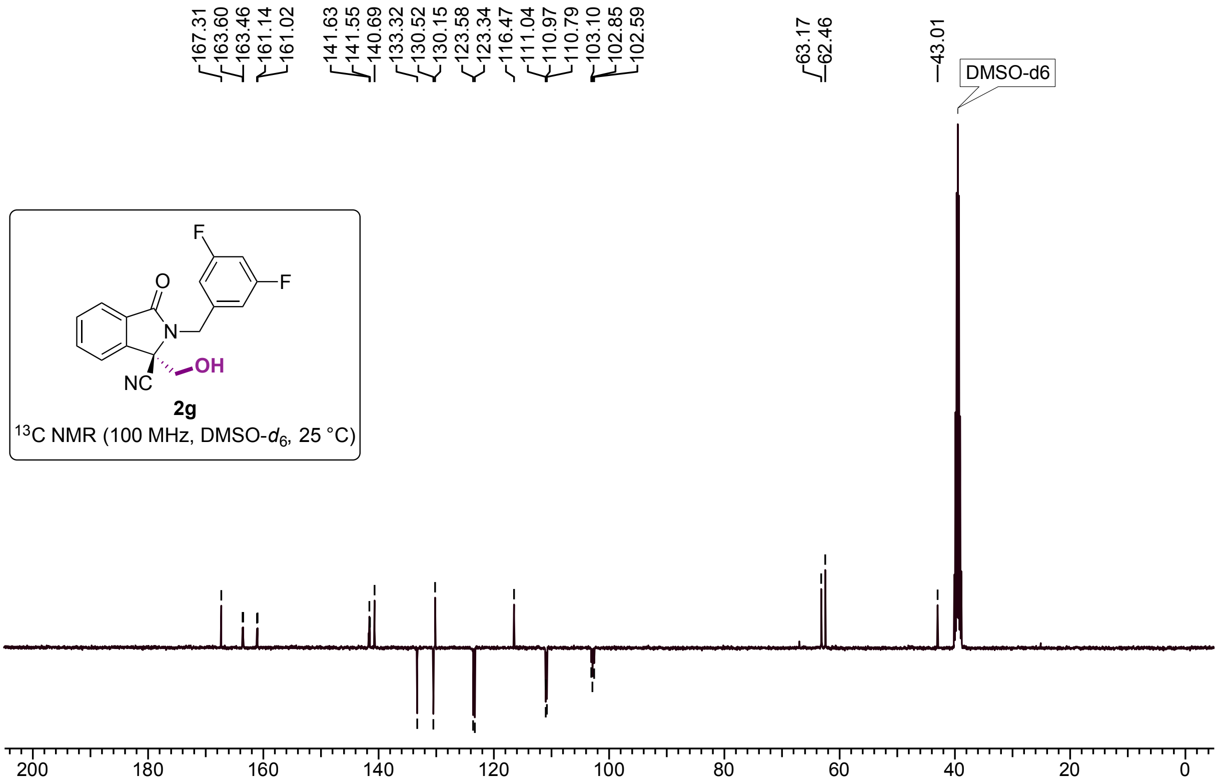
167.31
163.60
163.46
161.14
161.02
141.63
141.55
140.69
133.32
130.52
130.15
123.58
123.34
116.47
111.04
110.97
110.79
103.10
102.85
102.59
63.17
62.46
43.01
DMSO-d6
2g
^{13}C NMR (100 MHz, DMSO-d_6, 25 °C)
200 180 160 140 120 100 80 60 40 20 0

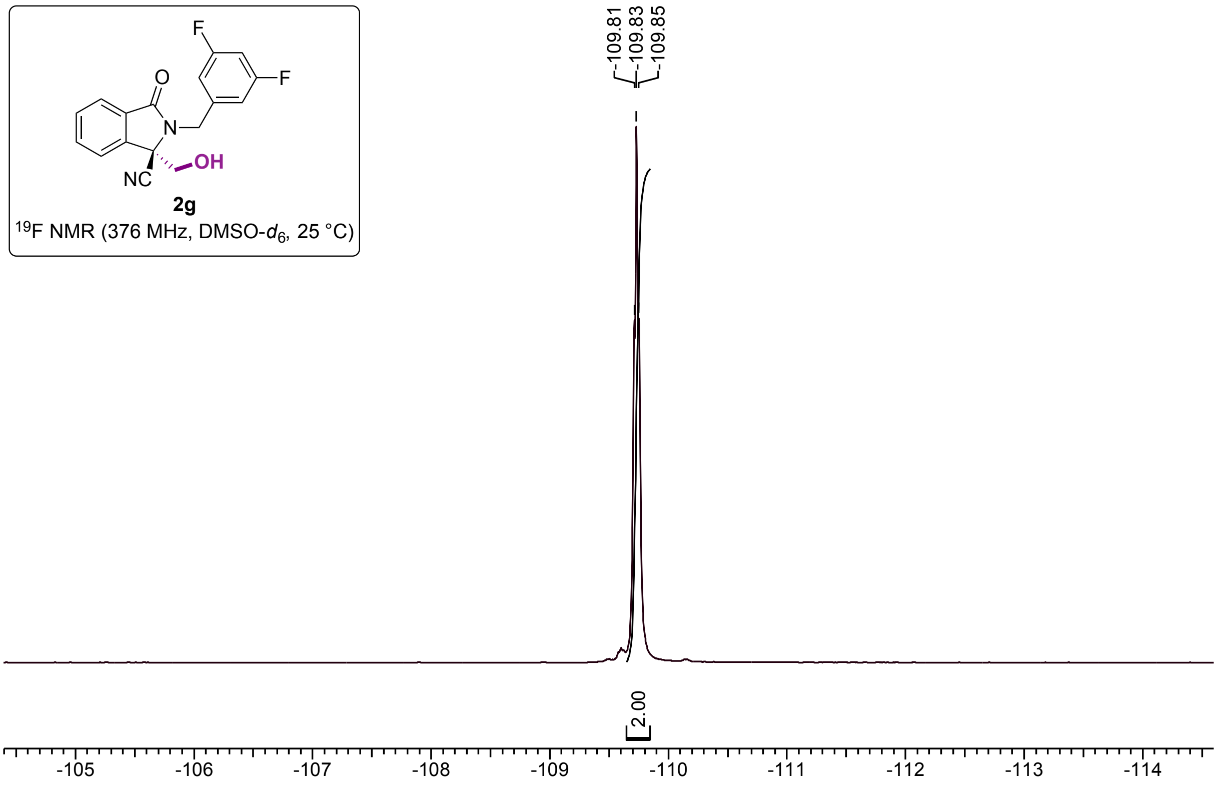
-109.81
-109.83
-109.85
2g
^{19}F NMR (376 MHz, DMSO-d_6, 25 °C)
2.00
-105 -106 -107 -108 -109 -110 -111 -112 -113 -114

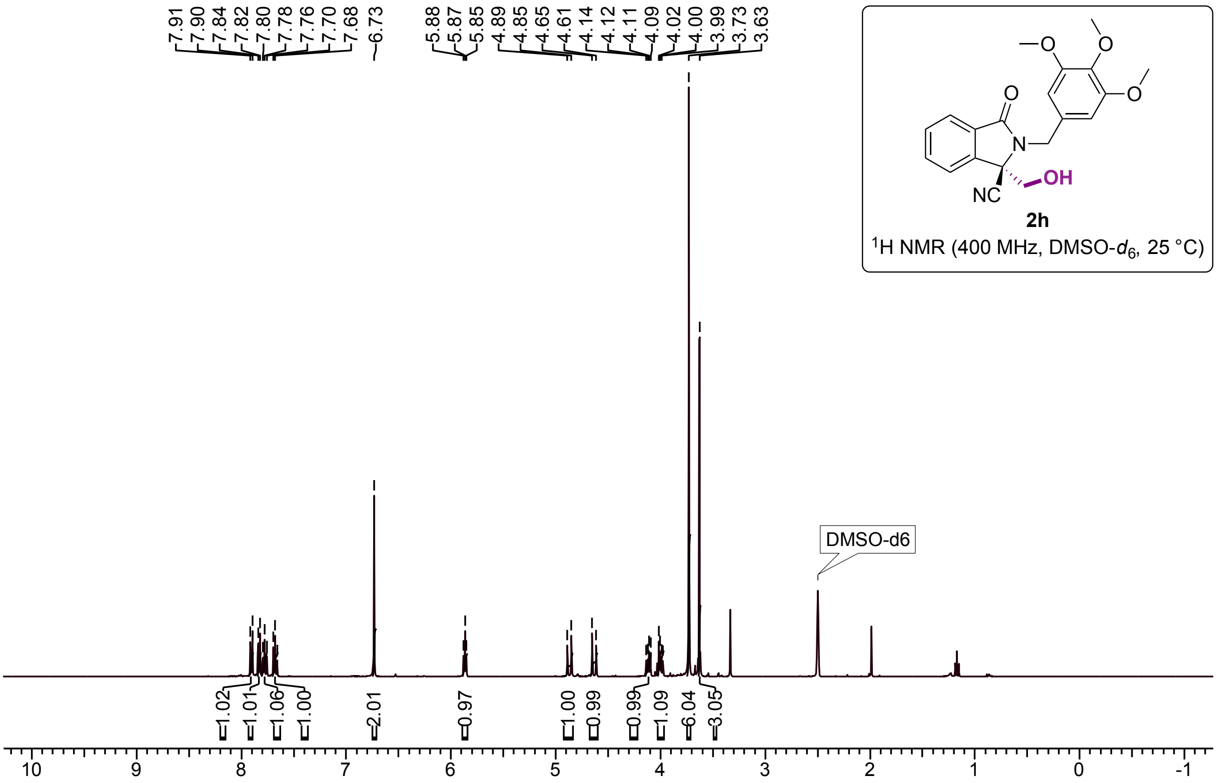
7.91 7.90 7.84 7.82 7.80 7.78 7.76 7.70 7.68
6.73
5.88 5.87 5.85 4.89 4.85 4.65 4.61 4.14 4.12 4.11 4.09 4.02 4.00 3.99 3.73 3.63
2h
1H NMR (400 MHz, DMSO-d6, 25 °C)
DMSO-d6
1.02 1.01 1.06 1.00 2.01 0.97 1.00 0.99 0.99 1.09 6.04 3.05
10 9 8 7 6 5 4 3 2 1 0 -1

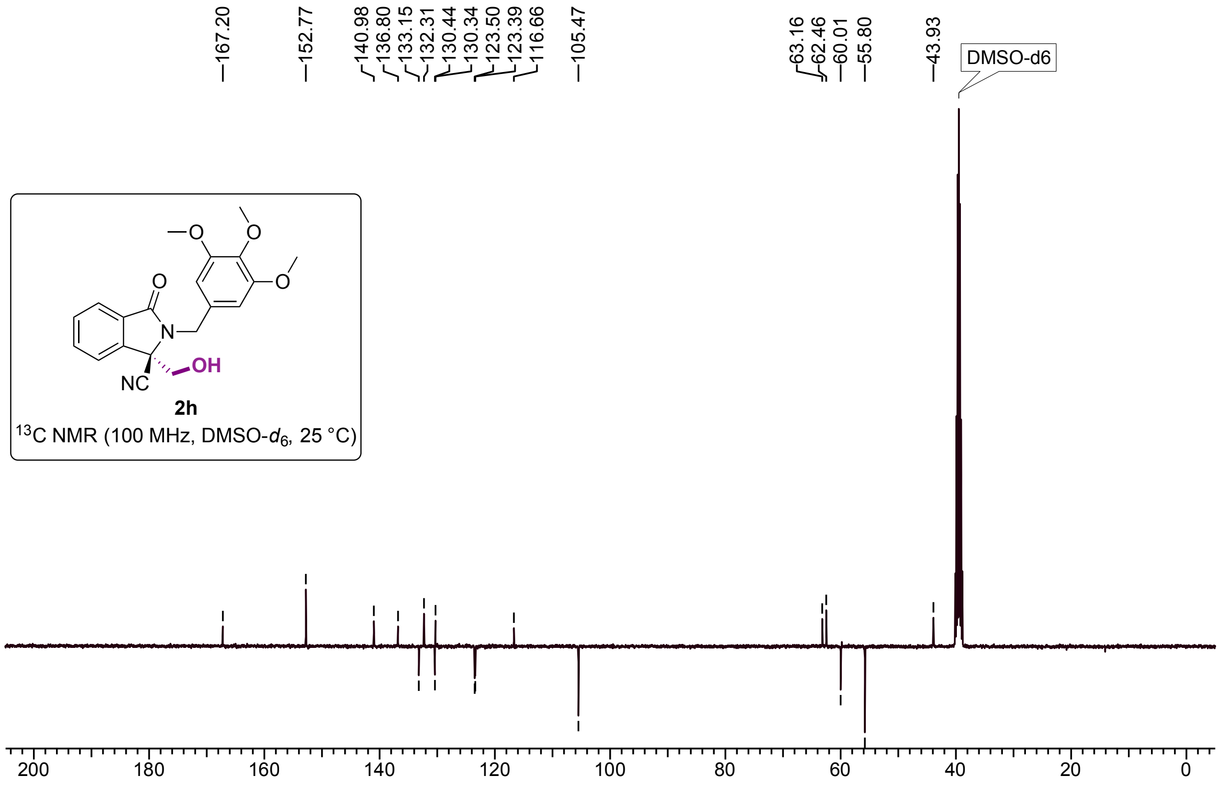
167.20
152.77
140.98 136.80 133.15 132.31 130.44 130.34 123.50 123.39 116.66
105.47
63.16 62.46 60.01 55.80
43.93
DMSO-d6
2h
13C NMR (100 MHz, DMSO-d6, 25 °C)
200 180 160 140 120 100 80 60 40 20 0

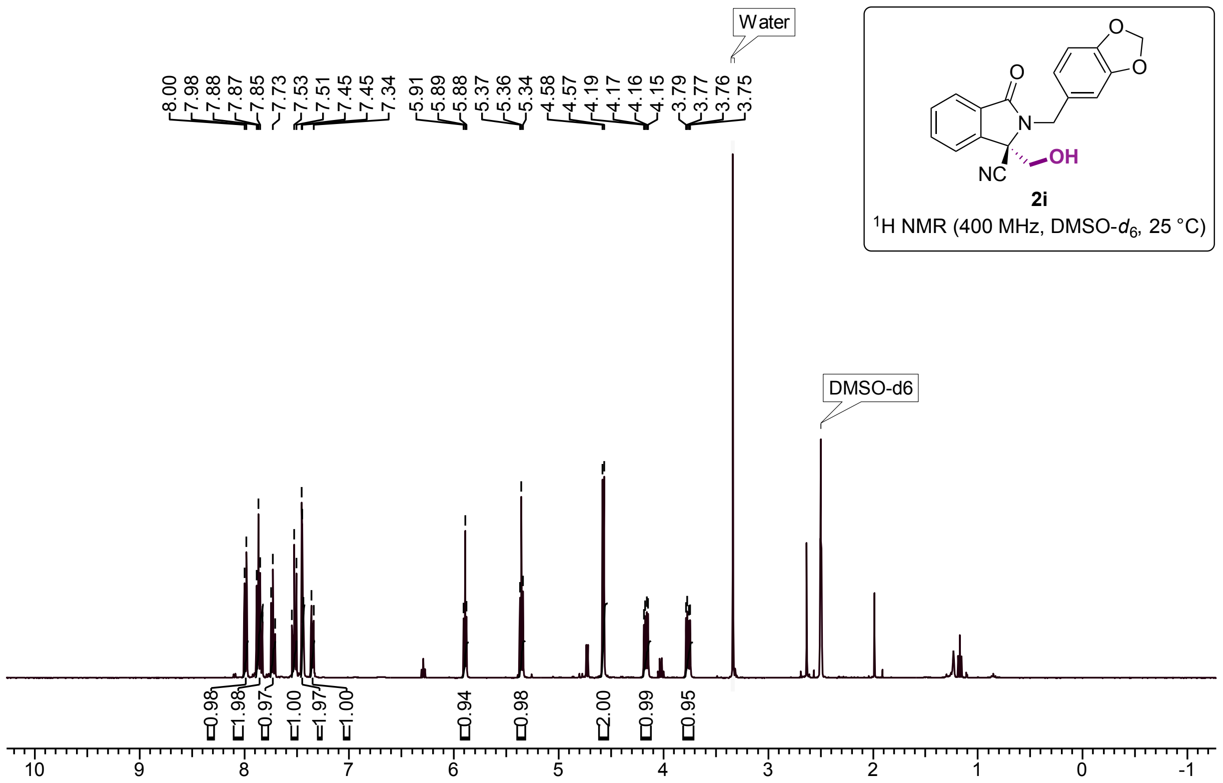
Water
8.00
7.98
7.88
7.87
7.85
7.73
7.53
7.51
7.45
7.45
7.34
5.91
5.89
5.88
5.37
5.36
5.34
4.58
4.57
4.19
4.17
4.16
4.15
3.79
3.77
3.76
3.75
DMSO-d6
0.98
1.98
0.97
1.00
1.97
1.00
0.94
0.98
2.00
0.99
0.95
10
9
8
7
6
5
4
3
2
1
0
-1
2i
1H NMR (400 MHz, DMSO-d6, 25 °C)

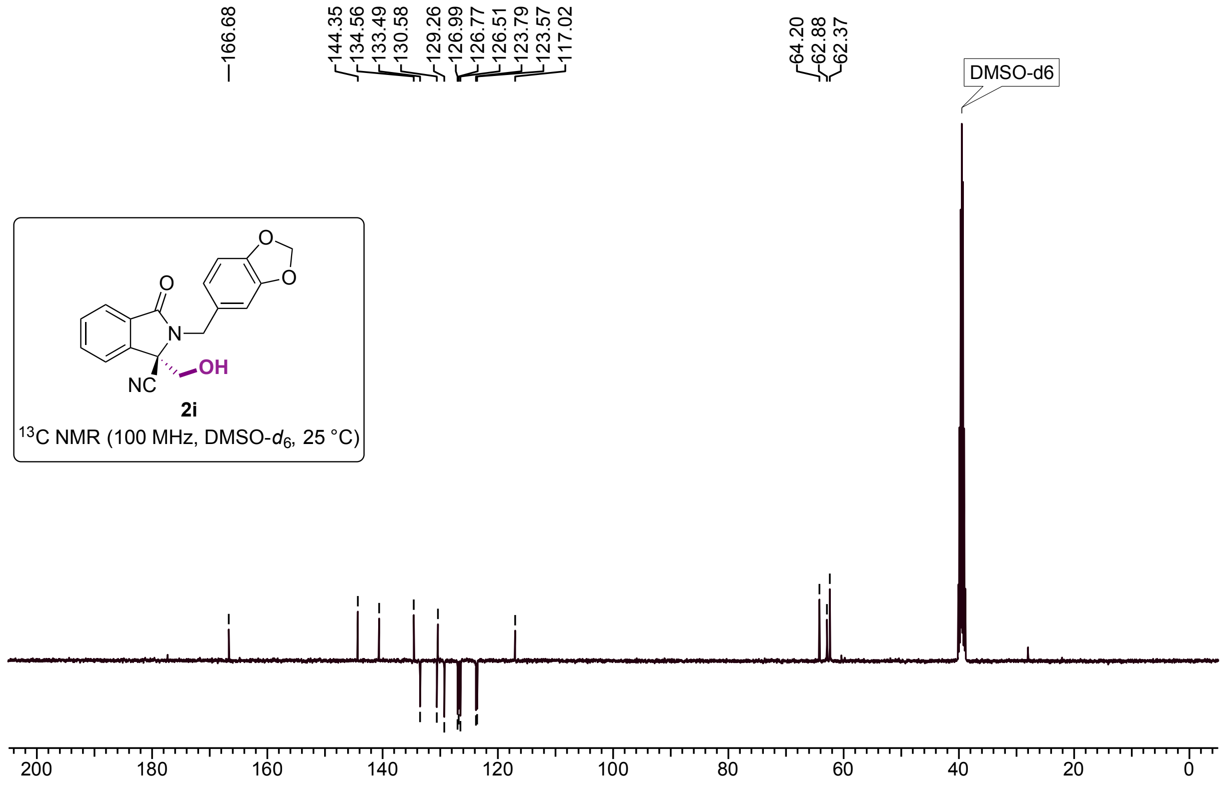
166.68
144.35
134.56
133.49
130.58
129.26
126.99
126.77
126.51
123.79
123.57
117.02
64.20
62.88
62.37
DMSO-d6
2i
13C NMR (100 MHz, DMSO-d6, 25 °C)
200
180
160
140
120
100
80
60
40
20
0

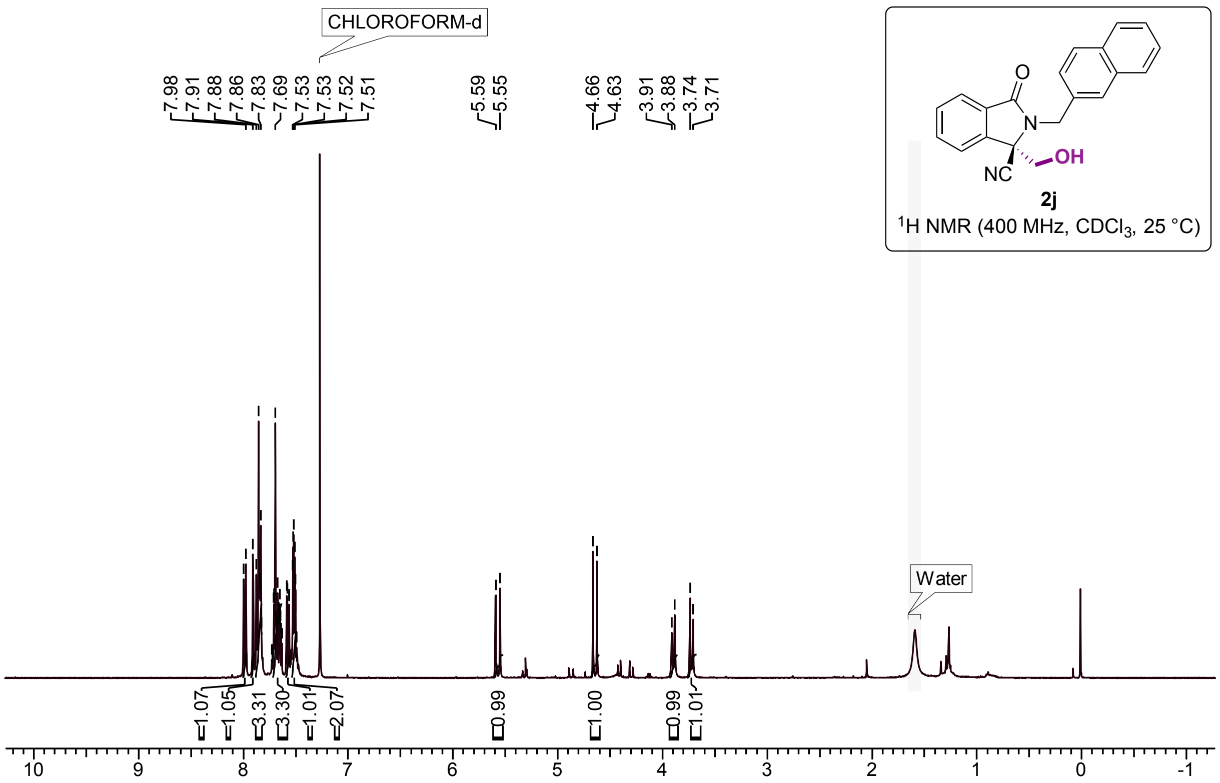
CHLOROFORM-d
7.98
7.91
7.88
7.86
7.83
7.69
7.53
7.53
7.52
7.51
5.59
5.55
4.66
4.63
3.91
3.88
3.74
3.71
Water
1.07
1.05
3.31
3.30
1.01
2.07
0.99
1.00
0.99
1.01
10
9
8
7
6
5
4
3
2
1
0
-1
O
N
OH
NC
2j
1H NMR (400 MHz, CDCl3, 25 °C)

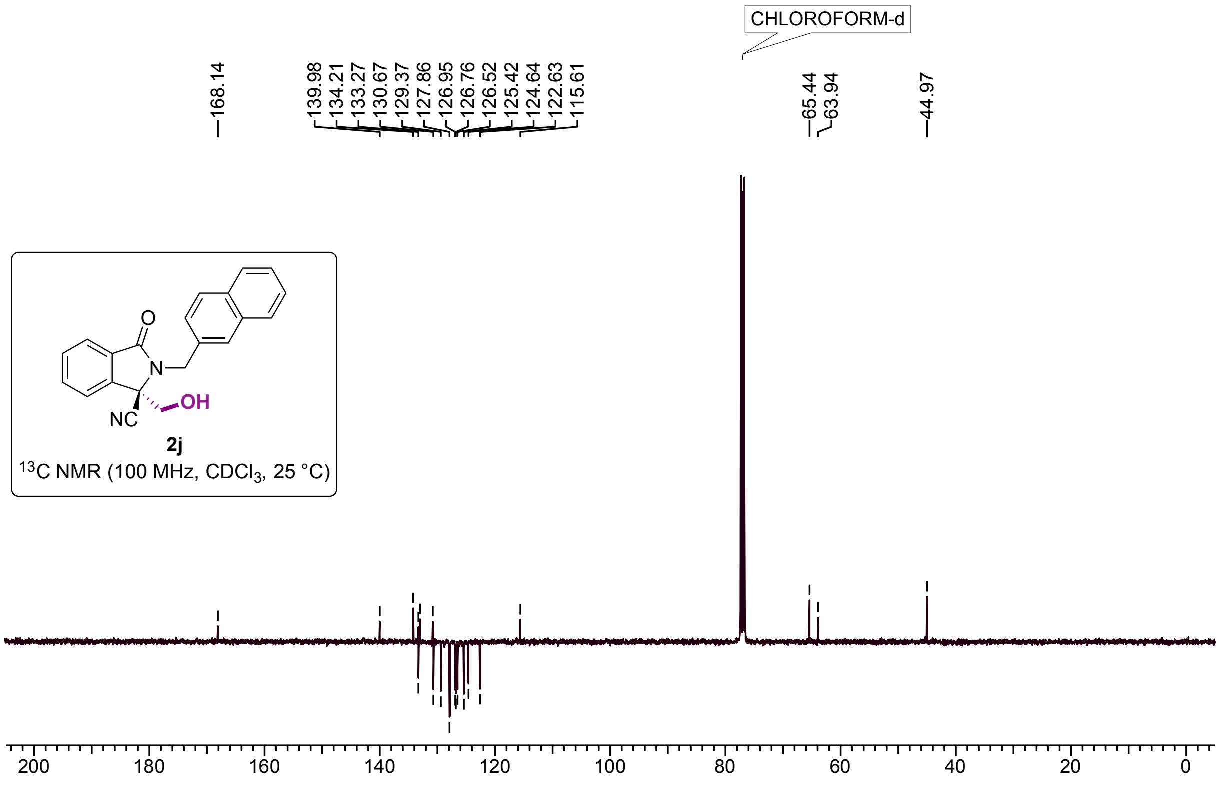
CHLOROFORM-d
168.14
139.98
134.21
133.27
130.67
129.37
127.86
126.95
126.76
126.52
125.42
124.64
122.63
115.61
65.44
63.94
44.97
O
N
OH
NC
2j
13C NMR (100 MHz, CDCl3, 25 °C)
200
180
160
140
120
100
80
60
40
20
0

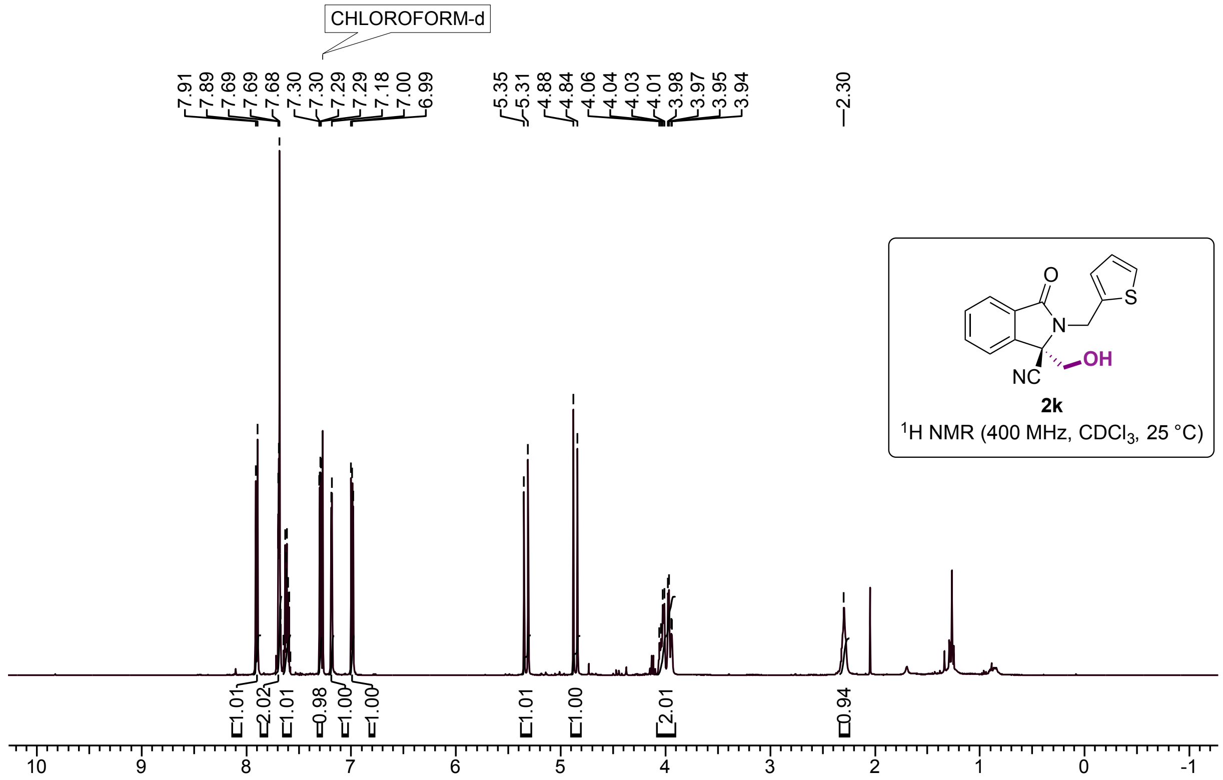
CHLOROFORM-d
7.91 7.89 7.69 7.69 7.69 7.68 7.30 7.30 7.29 7.29 7.18 7.00 6.99
5.35 5.31 4.88 4.84 4.06 4.04 4.03 4.01 3.98 3.97 3.95 3.94
2.30
1.01 2.02 1.01 0.98 1.00 1.00 1.01 1.00 2.01 0.94
O
N
S
OH
NC
2k
¹H NMR (400 MHz, CDCl₃, 25 °C)
10 9 8 7 6 5 4 3 2 1 0 -1

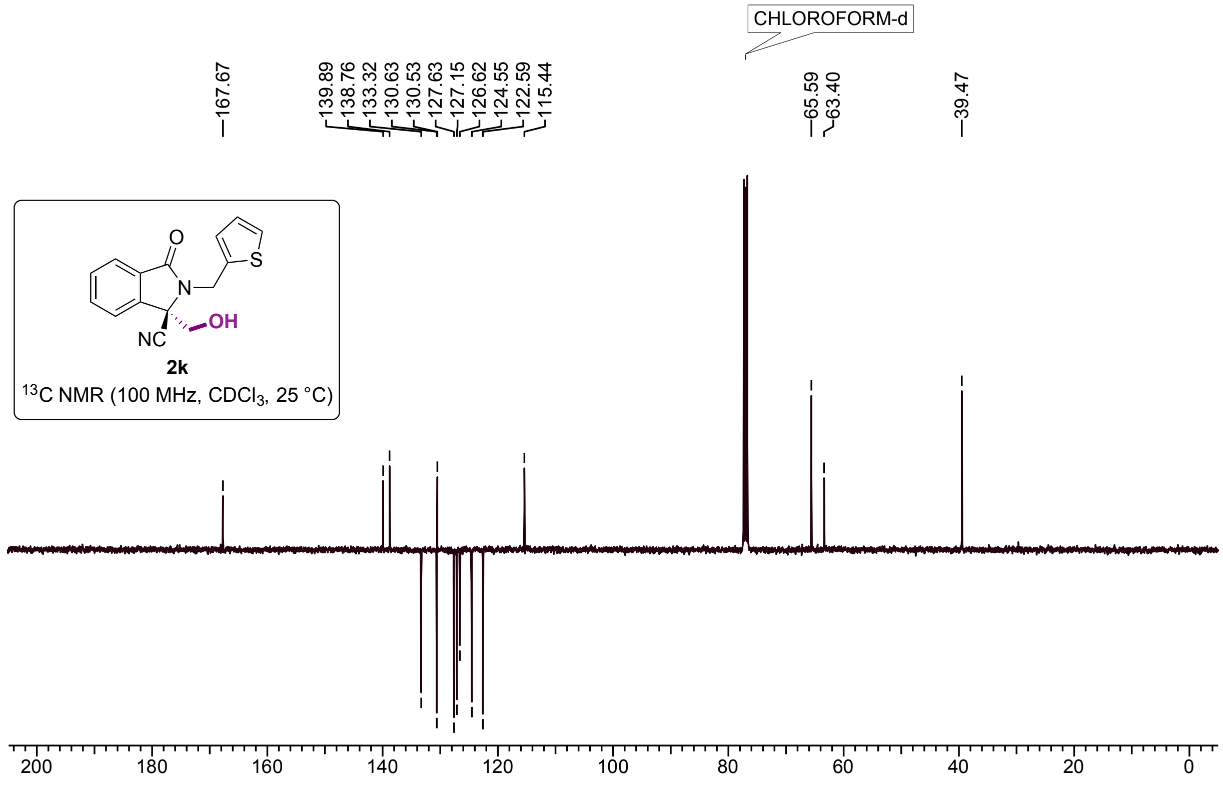
CHLOROFORM-d
167.67
139.89 138.76 133.32 130.63 130.53 127.63 127.15 126.62 124.55 122.59 115.44
65.59 63.40
39.47
O
N
S
OH
NC
2k
¹³C NMR (100 MHz, CDCl₃, 25 °C)
200 180 160 140 120 100 80 60 40 20 0

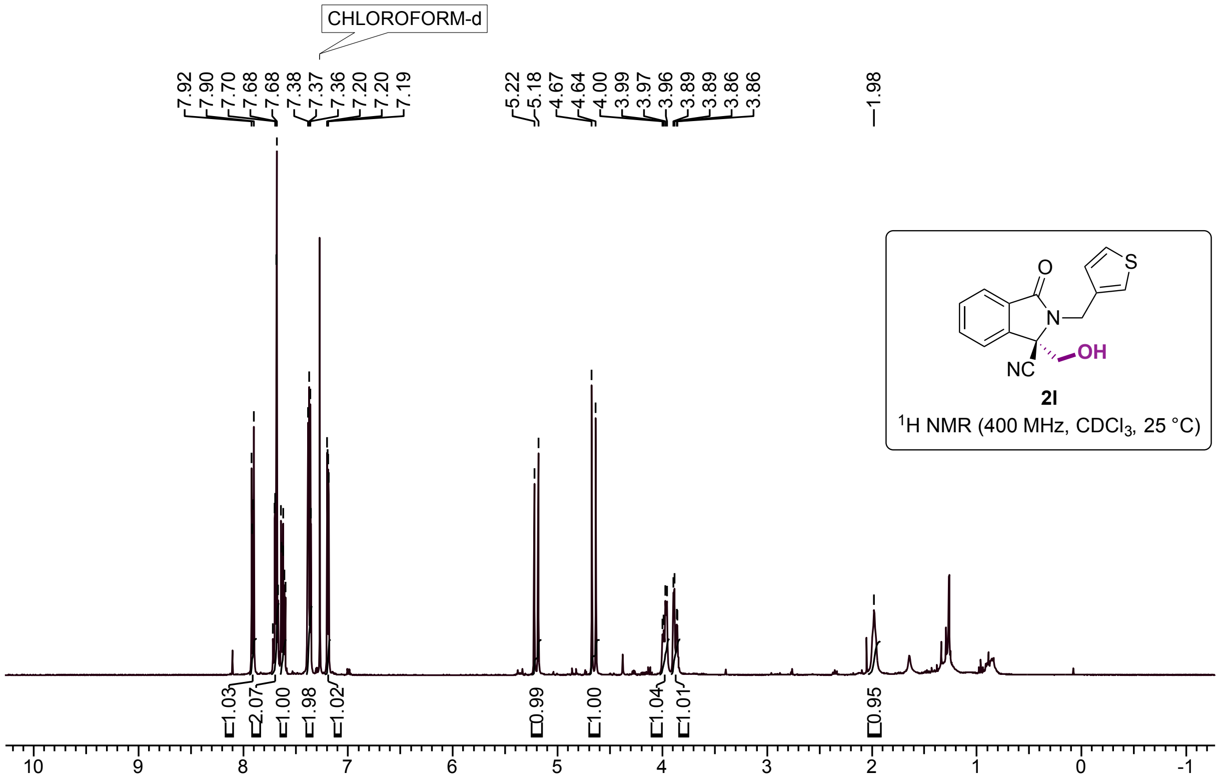
CHLOROFORM-d
7.92
7.90
7.70
7.68
7.68
7.38
7.37
7.36
7.20
7.20
7.19
5.22
5.18
4.67
4.64
4.00
3.99
3.97
3.96
3.89
3.86
3.86
1.98
O
S
N
OH
NC
2l
1H NMR (400 MHz, CDCl3, 25 °C)
1.03
2.07
1.00
1.98
1.02
0.99
1.00
1.04
1.01
0.95
10
9
8
7
6
5
4
3
2
1
0
-1

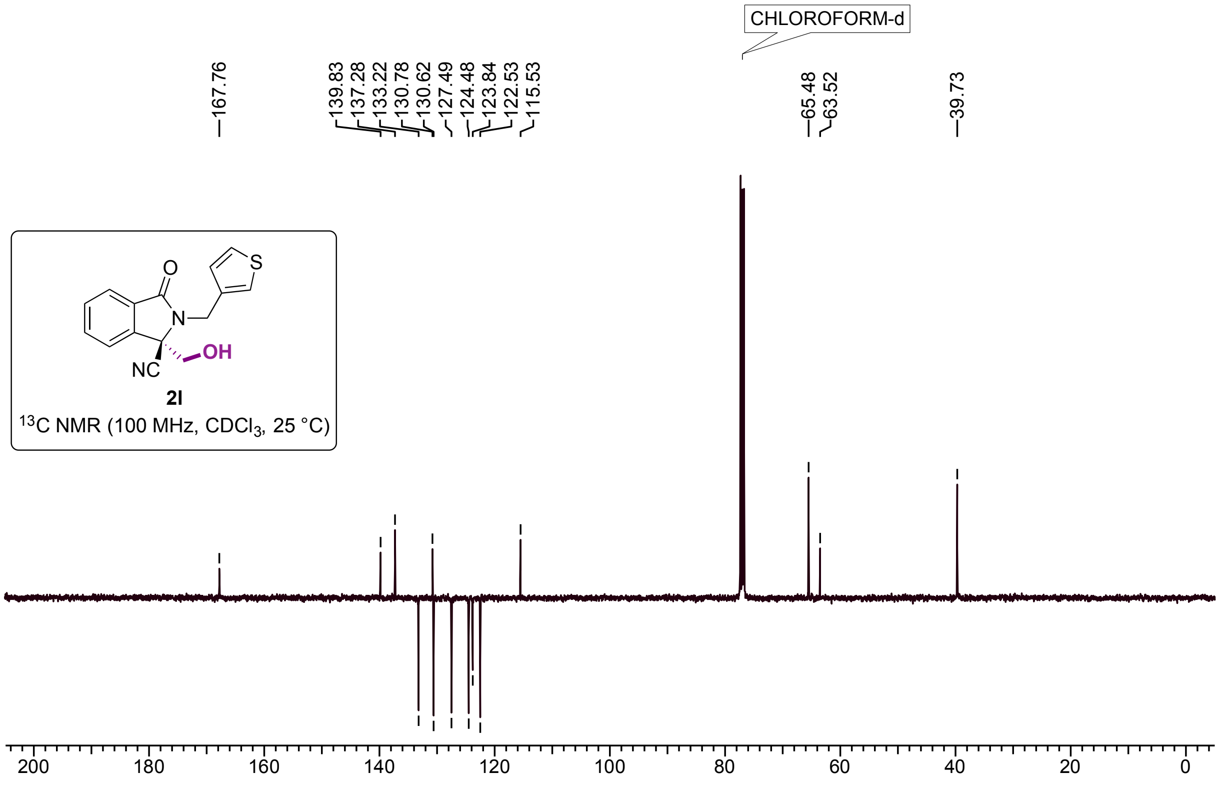
CHLOROFORM-d
167.76
139.83
137.28
133.22
130.78
130.62
127.49
124.48
123.84
122.53
115.53
65.48
63.52
39.73
O
S
N
OH
NC
2l
13C NMR (100 MHz, CDCl3, 25 °C)
200
180
160
140
120
100
80
60
40
20
0

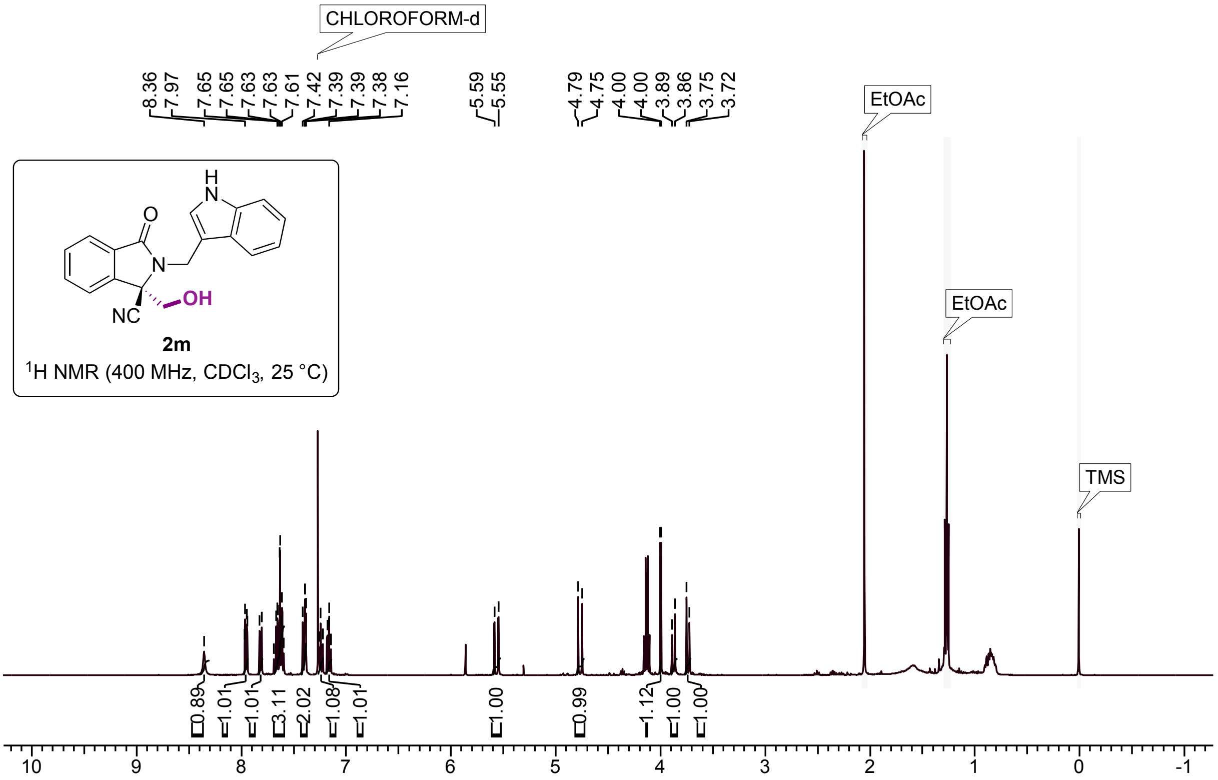
CHLOROFORM-d
8.36
7.97
7.65
7.65
7.63
7.63
7.61
7.42
7.39
7.39
7.38
7.16
5.59
5.55
4.79
4.75
4.00
4.00
3.89
3.86
3.75
3.72
EtOAc
EtOAc
TMS
2m
^{1}H NMR (400 MHz, $CDCl_3$, 25 °C)
0.89
1.01
1.01
3.11
2.02
1.08
1.01
1.00
0.99
1.12
1.00
1.00
10
9
8
7
6
5
4
3
2
1
0
-1

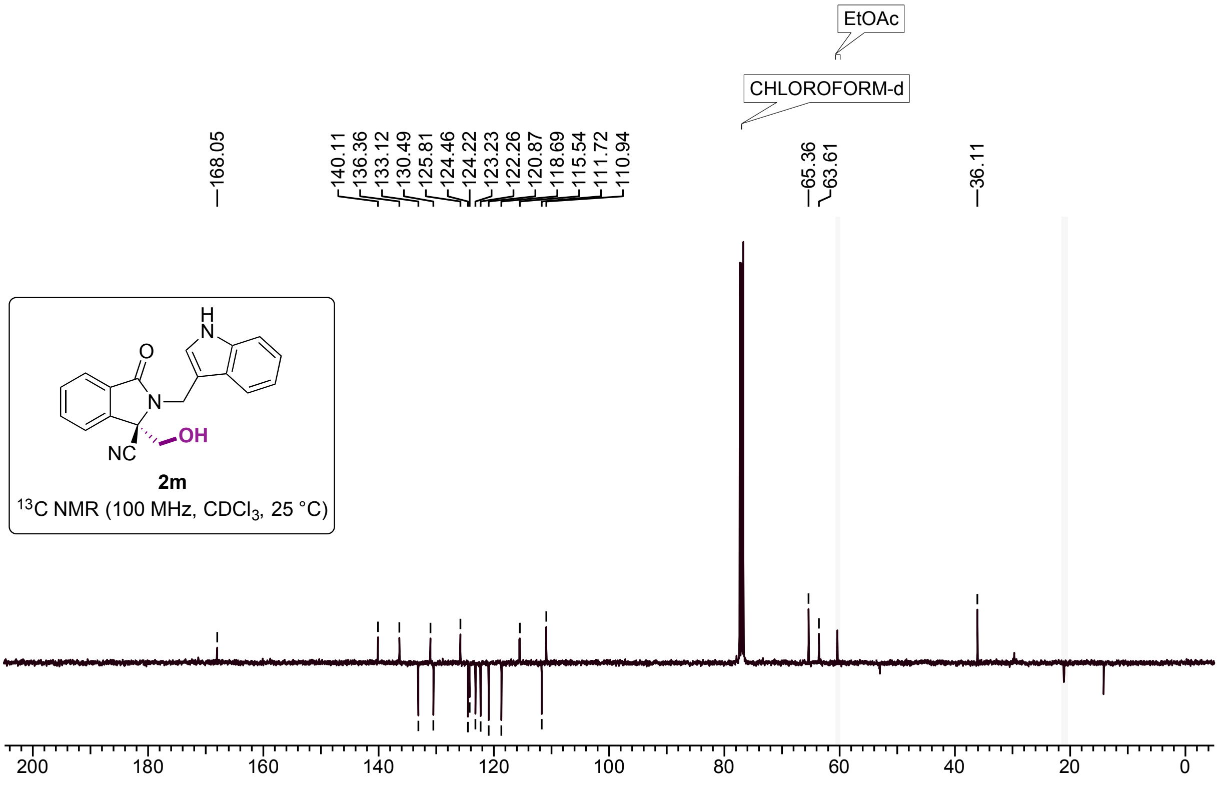
EtOAc
CHLOROFORM-d
168.05
140.11
136.36
133.12
130.49
125.81
124.46
124.22
123.23
122.26
120.87
118.69
115.54
111.72
110.94
65.36
63.61
36.11
2m
^{13}C NMR (100 MHz, $CDCl_3$, 25 °C)
200
180
160
140
120
100
80
60
40
20
0

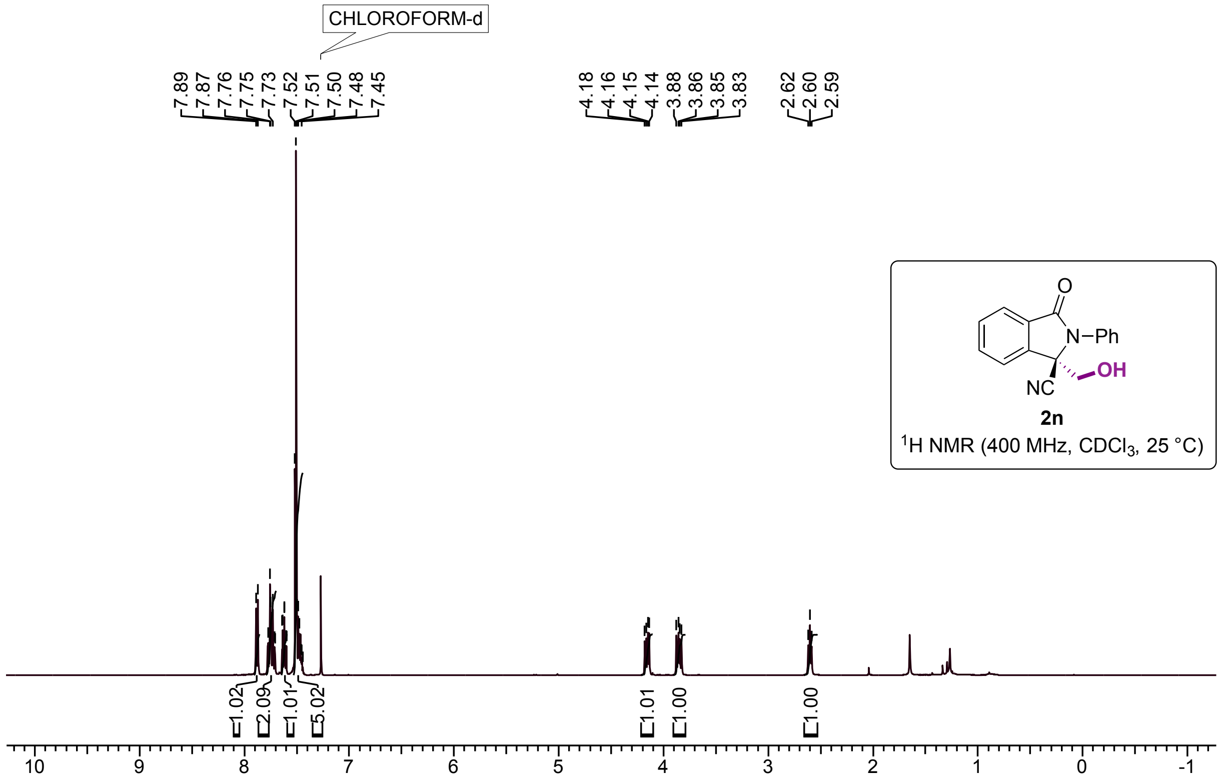

CHLOROFORM-d
7.89
7.87
7.76
7.75
7.73
7.52
7.51
7.50
7.48
7.45
4.18
4.16
4.15
4.14
3.88
3.86
3.85
3.83
2.62
2.60
2.59
N−Ph
OH
NC
O
2n
1H NMR (400 MHz, CDCl3, 25 °C)
1.02
2.09
1.01
5.02
1.01
1.00
1.00
10
9
8
7
6
5
4
3
2
1
0
-1

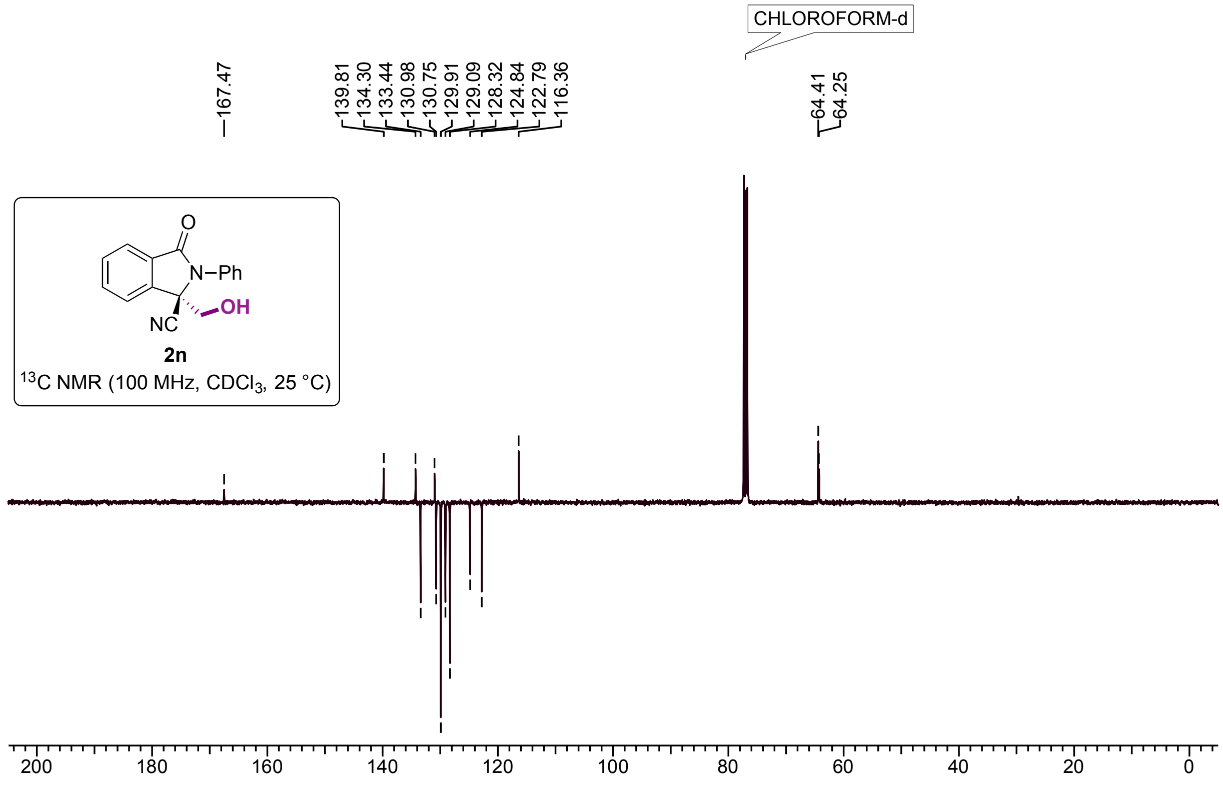

CHLOROFORM-d
167.47
139.81
134.30
133.44
130.98
130.75
129.91
129.09
128.32
124.84
122.79
116.36
64.41
64.25
O
N−Ph
OH
NC
2n
13C NMR (100 MHz, CDCl3, 25 °C)
200
180
160
140
120
100
80
60
40
20
0

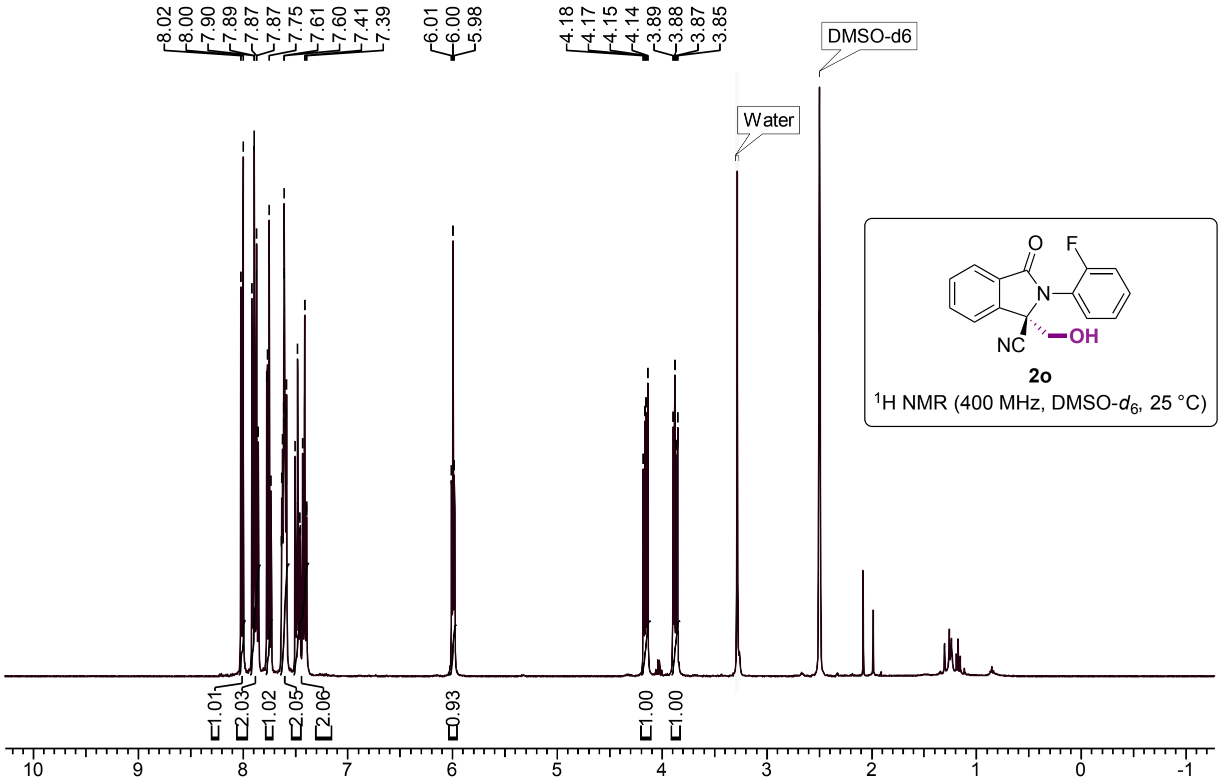
8.02
8.00
7.90
7.89
7.87
7.87
7.75
7.61
7.60
7.41
7.39
6.01
6.00
5.98
4.18
4.17
4.15
4.14
3.89
3.88
3.87
3.85
DMSO-d6
Water
O
F
N
NC
OH
2o
^{1}H NMR (400 MHz, DMSO-d_6, 25 °C)
1.01
2.03
1.02
2.05
2.06
0.93
1.00
1.00
10
9
8
7
6
5
4
3
2
1
0
-1

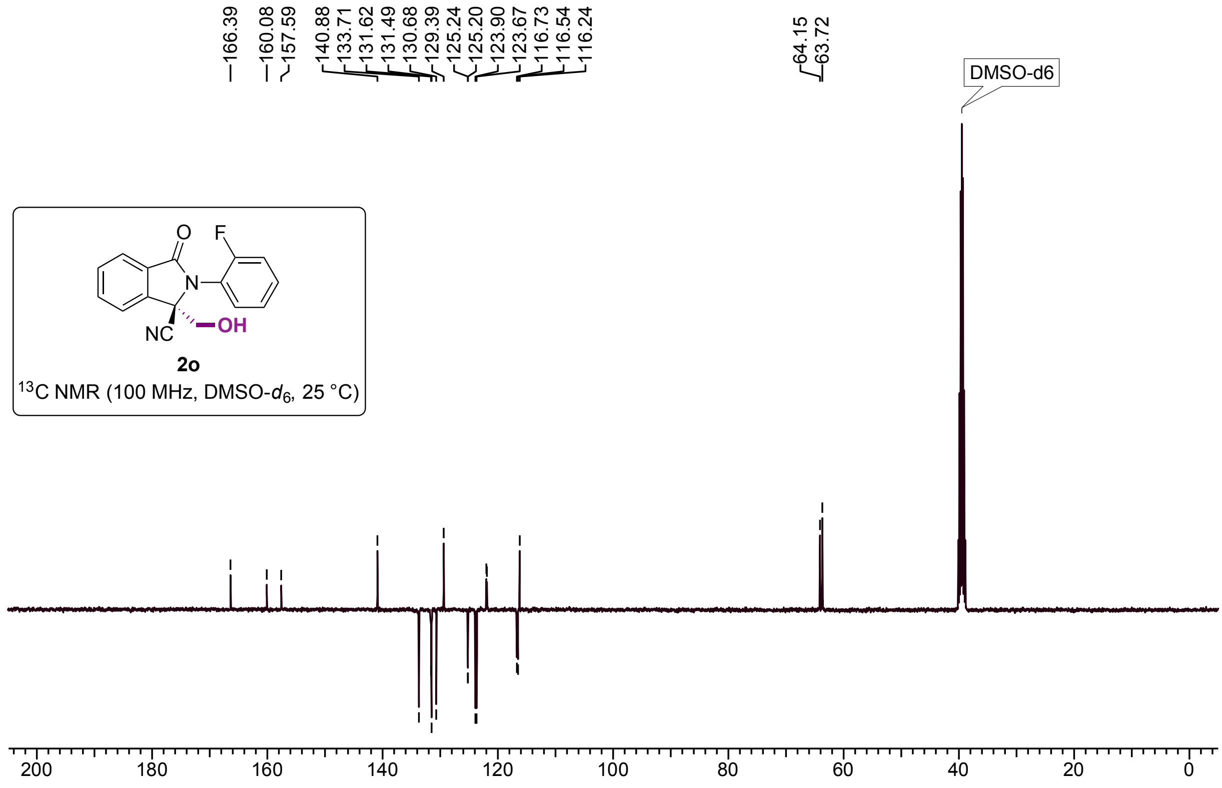
166.39
160.08
157.59
140.88
133.71
131.62
131.49
130.68
129.39
125.24
125.20
123.90
123.67
116.73
116.54
116.24
64.15
63.72
DMSO-d6
O
F
N
NC
OH
2o
^{13}C NMR (100 MHz, DMSO-d_6, 25 °C)
200
180
160
140
120
100
80
60
40
20
0

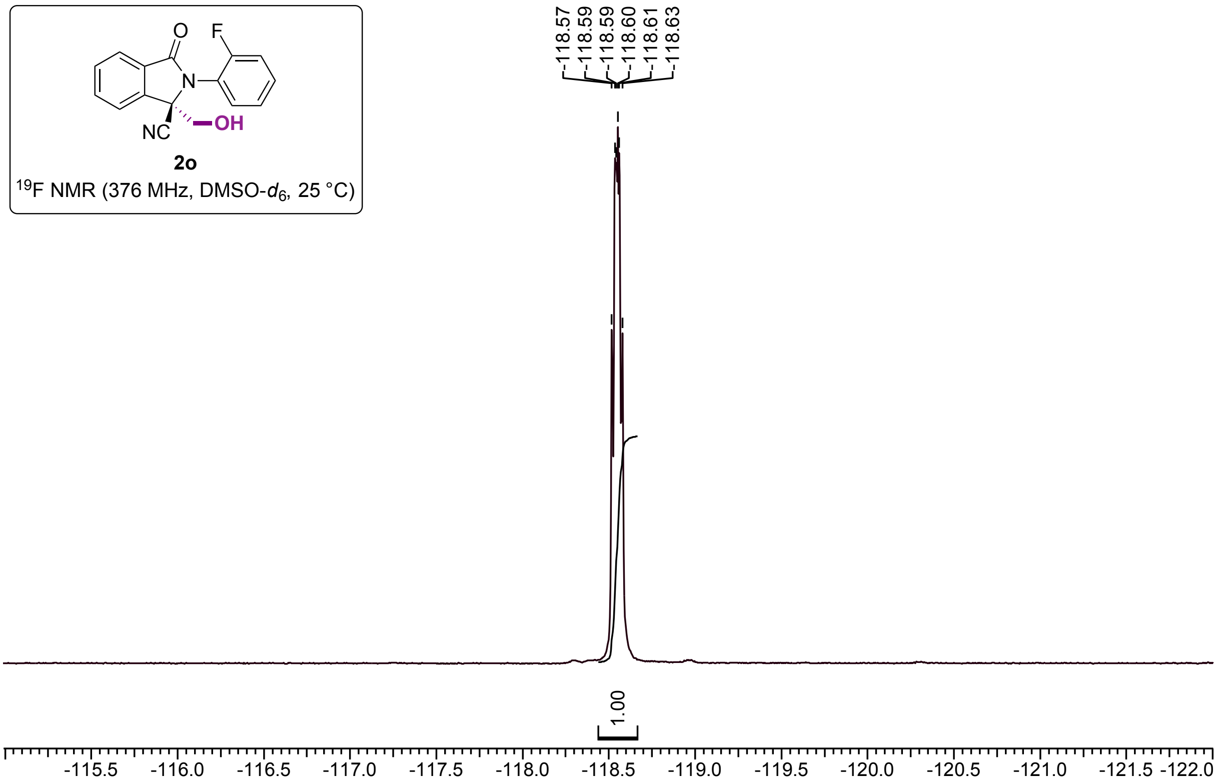

2o
^{19}F NMR (376 MHz, DMSO-d_6, 25 °C)
-118.57
-118.59
-118.59
-118.60
-118.61
-118.63
1.00
-115.5 -116.0 -116.5 -117.0 -117.5 -118.0 -118.5 -119.0 -119.5 -120.0 -120.5 -121.0 -121.5 -122.0

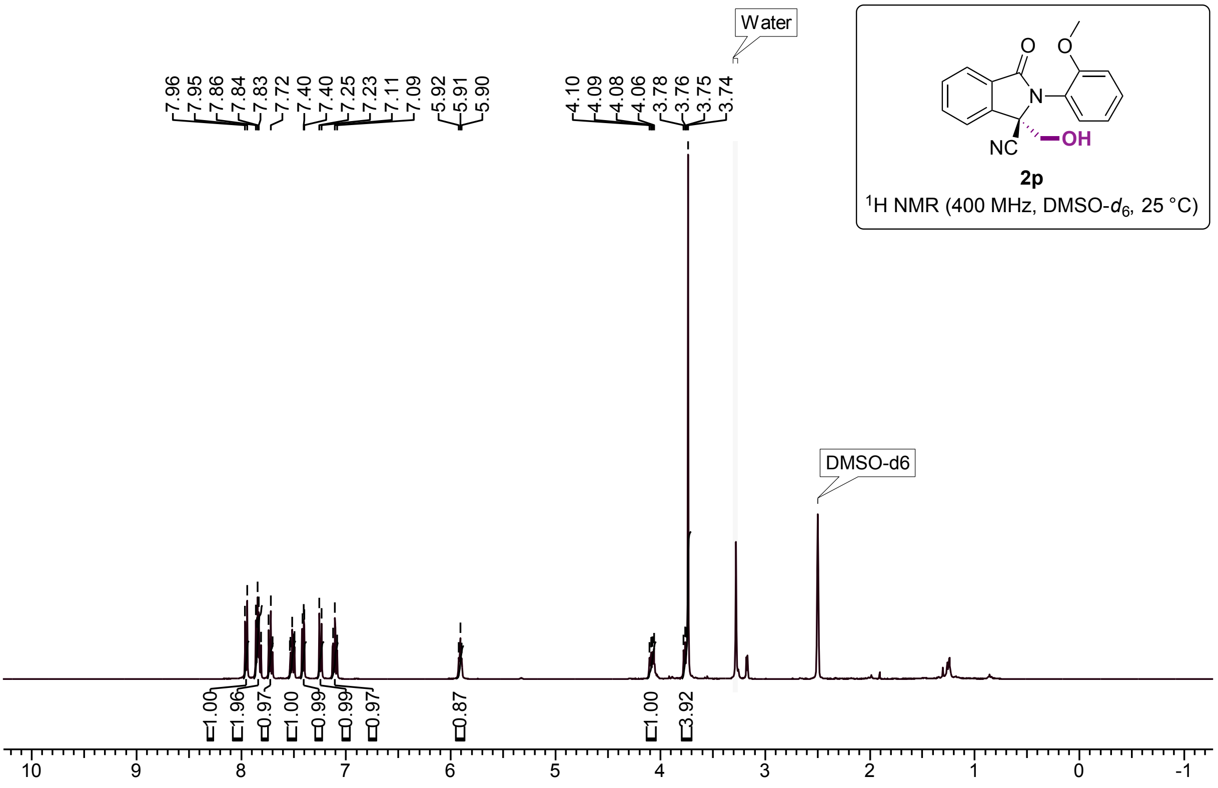

Water
DMSO-d6
2p
^{1}H NMR (400 MHz, DMSO-d_6, 25 °C)
7.96
7.95
7.86
7.84
7.83
7.72
7.40
7.40
7.25
7.23
7.11
7.09
5.92
5.91
5.90
4.10
4.09
4.08
4.06
3.78
3.76
3.75
3.74
1.00
1.96
0.97
1.00
0.99
0.99
0.97
0.87
1.00
3.92
10 9 8 7 6 5 4 3 2 1 0 -1

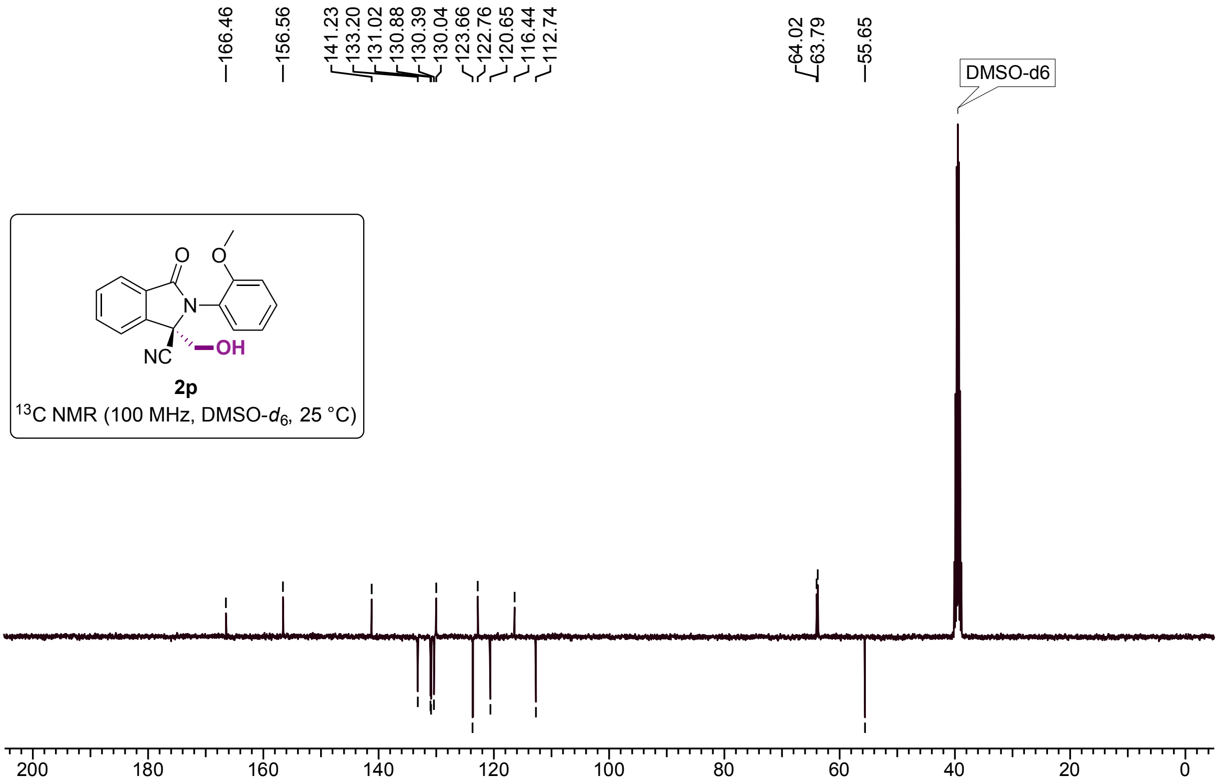
166.46
156.56
141.23
133.20
131.02
130.88
130.39
130.04
123.66
122.76
120.65
116.44
112.74
64.02
63.79
55.65
DMSO-d6
O
N
NC
OH
2p
^{13}C NMR (100 MHz, DMSO-d_6, 25 °C)
200
180
160
140
120
100
80
60
40
20
0

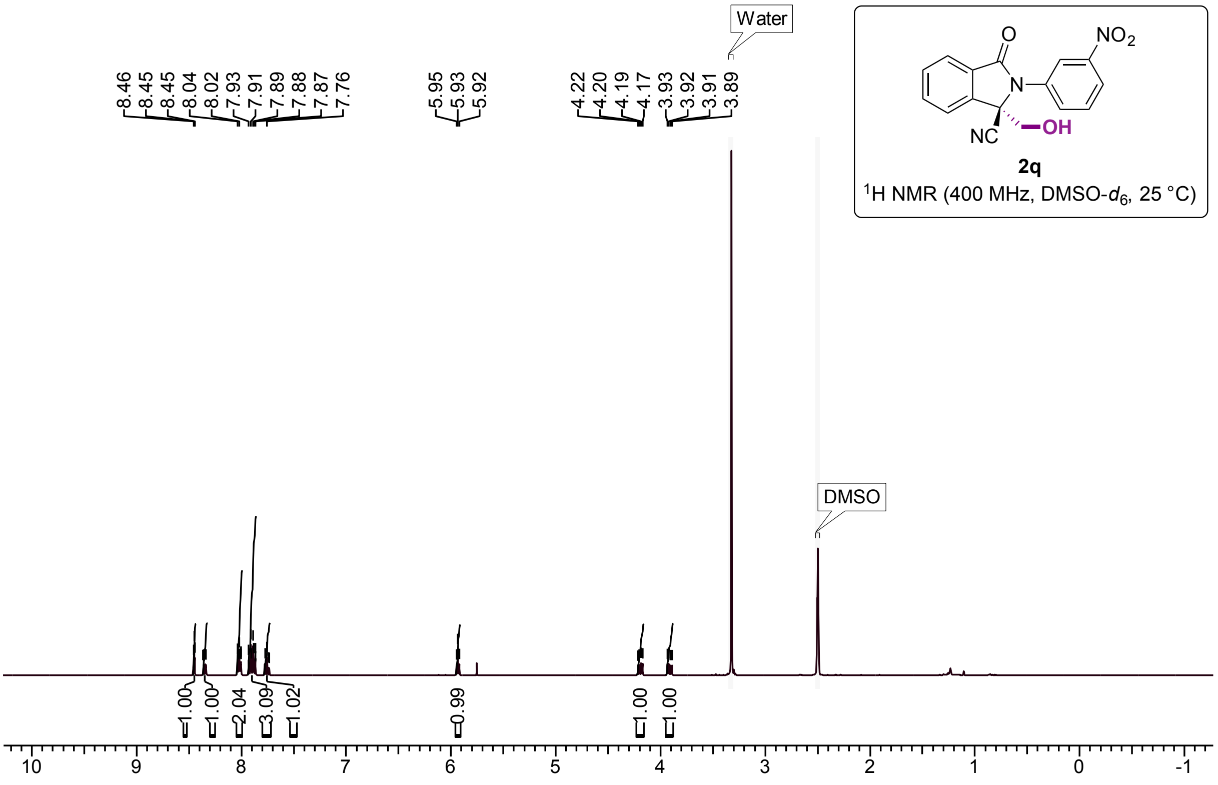
8.46
8.45
8.45
8.04
8.02
7.93
7.91
7.89
7.88
7.87
7.76
5.95
5.93
5.92
4.22
4.20
4.19
4.17
3.93
3.92
3.91
3.89
Water
DMSO
O
NO_2
N
NC
OH
2q
^{1}H NMR (400 MHz, DMSO-d_6, 25 °C)
1.00
1.00
2.04
3.09
1.02
0.99
1.00
1.00
10
9
8
7
6
5
4
3
2
1
0
-1

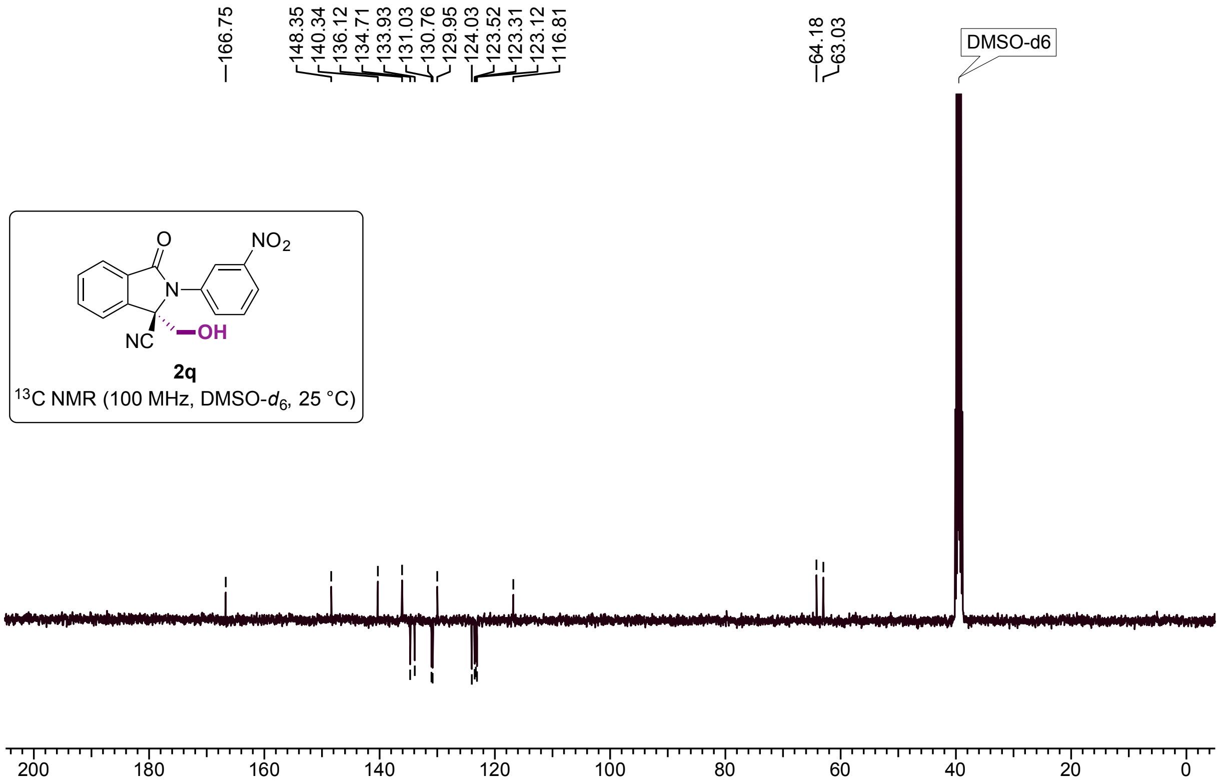
166.75
148.35
140.34
136.12
134.71
133.93
131.03
130.76
129.95
124.03
123.52
123.31
123.12
116.81
64.18
63.03
DMSO-d6
O
NO2
N
NC
OH
2q
13C NMR (100 MHz, DMSO-d6, 25 °C)
200
180
160
140
120
100
80
60
40
20
0

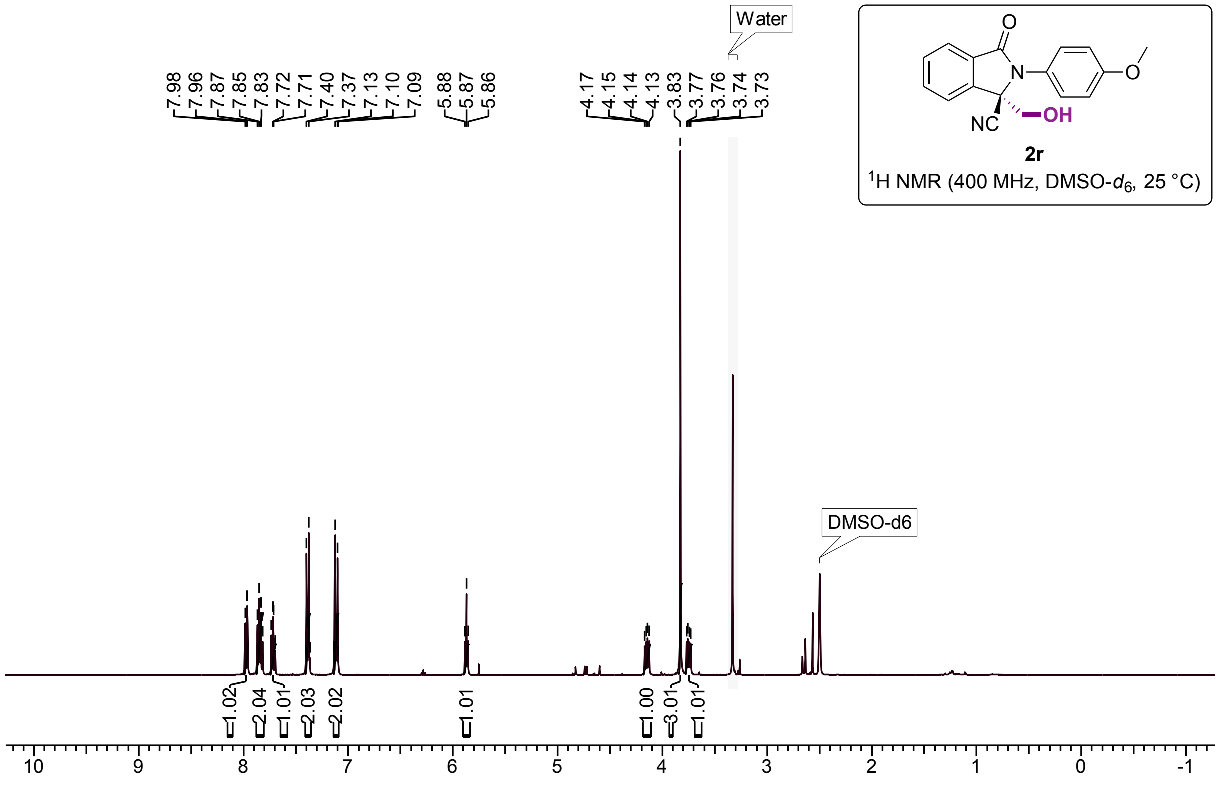
7.98
7.96
7.87
7.85
7.83
7.72
7.71
7.40
7.37
7.13
7.10
7.09
5.88
5.87
5.86
4.17
4.15
4.14
4.13
3.83
3.77
3.76
3.74
3.73
Water
DMSO-d6
O
N
O
NC
OH
2r
1H NMR (400 MHz, DMSO-d6, 25 °C)
1.02
2.04
1.01
2.03
2.02
1.01
1.00
3.01
1.01
10
9
8
7
6
5
4
3
2
1
0
-1

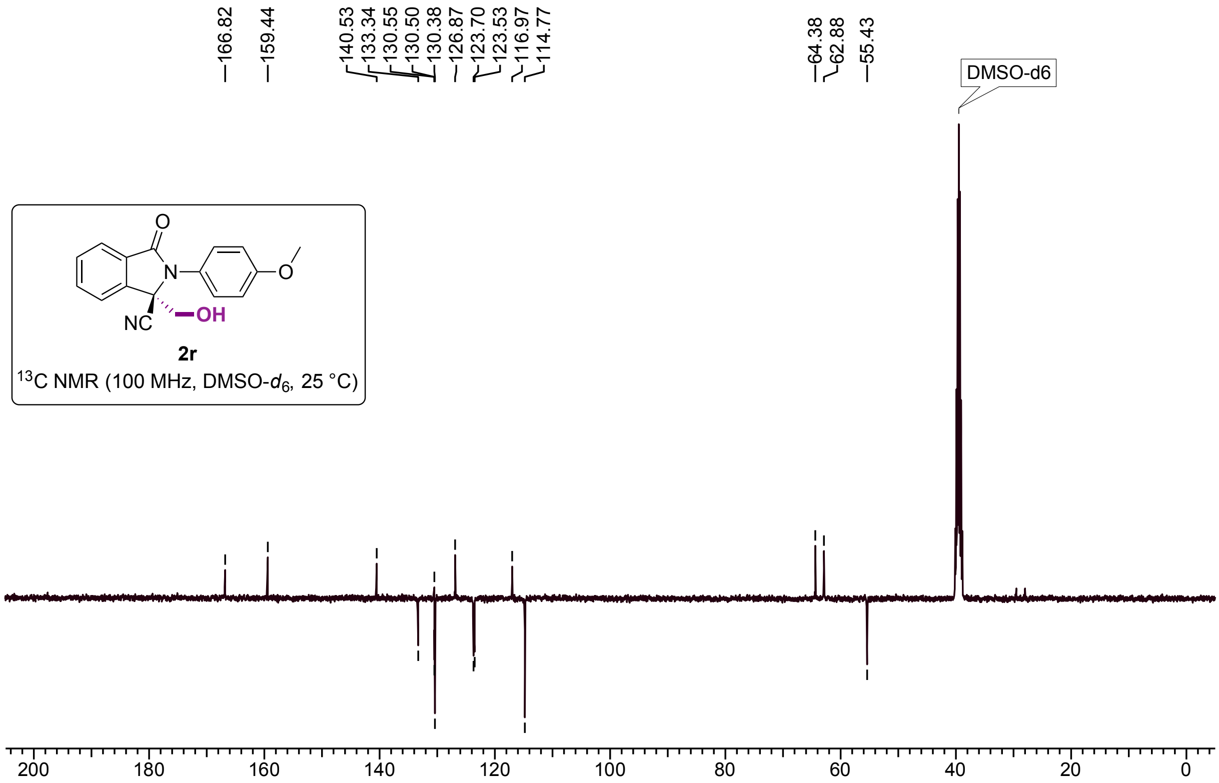
166.82
159.44
140.53
133.34
130.55
130.50
130.38
126.87
123.70
123.53
116.97
114.77
64.38
62.88
55.43
DMSO-d6
2r
^{13}C NMR (100 MHz, DMSO-d_6, 25 °C)
200
180
160
140
120
100
80
60
40
20
0

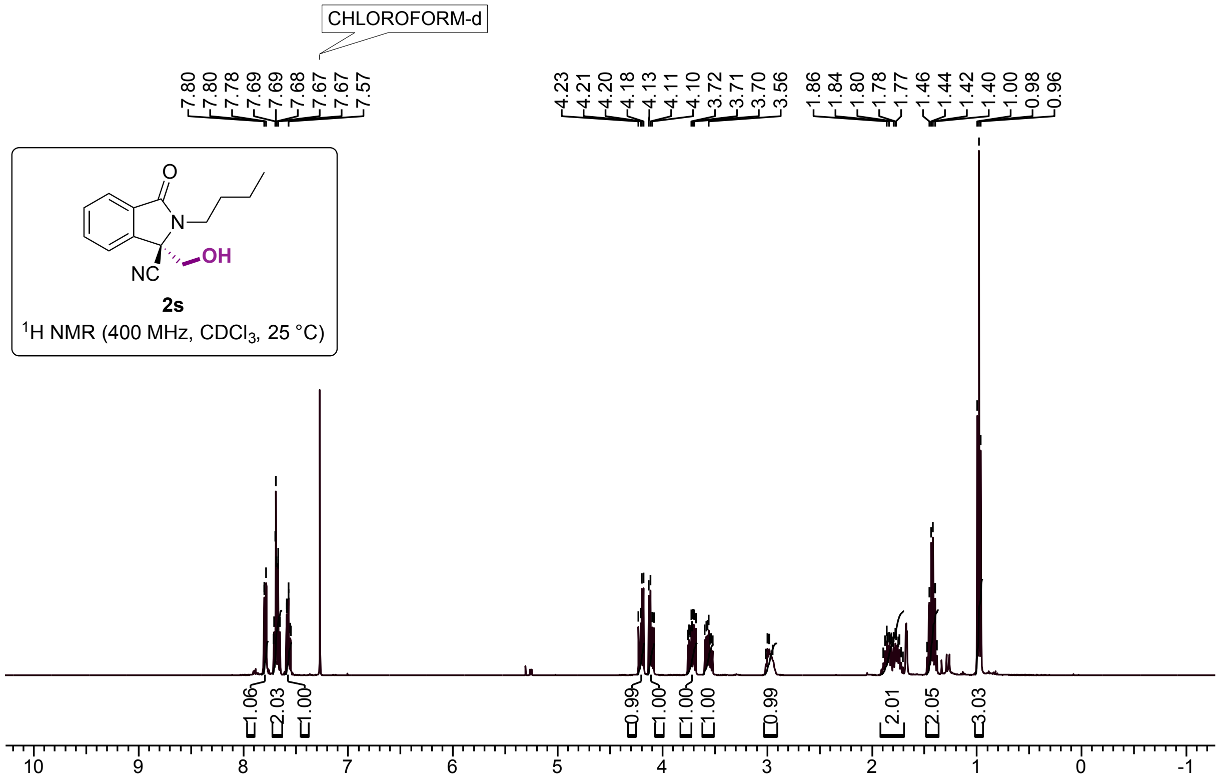
CHLOROFORM-d
7.80
7.80
7.78
7.69
7.69
7.68
7.67
7.67
7.57
4.23
4.21
4.20
4.18
4.13
4.11
4.10
3.72
3.71
3.70
3.56
1.86
1.84
1.80
1.78
1.77
1.46
1.44
1.42
1.40
1.00
0.98
0.96
2s
^{1}H NMR (400 MHz, CDCl$_3$, 25 °C)
1.06
2.03
1.00
0.99
1.00
1.00
1.00
0.99
2.01
2.05
3.03
10
9
8
7
6
5
4
3
2
1
0
-1

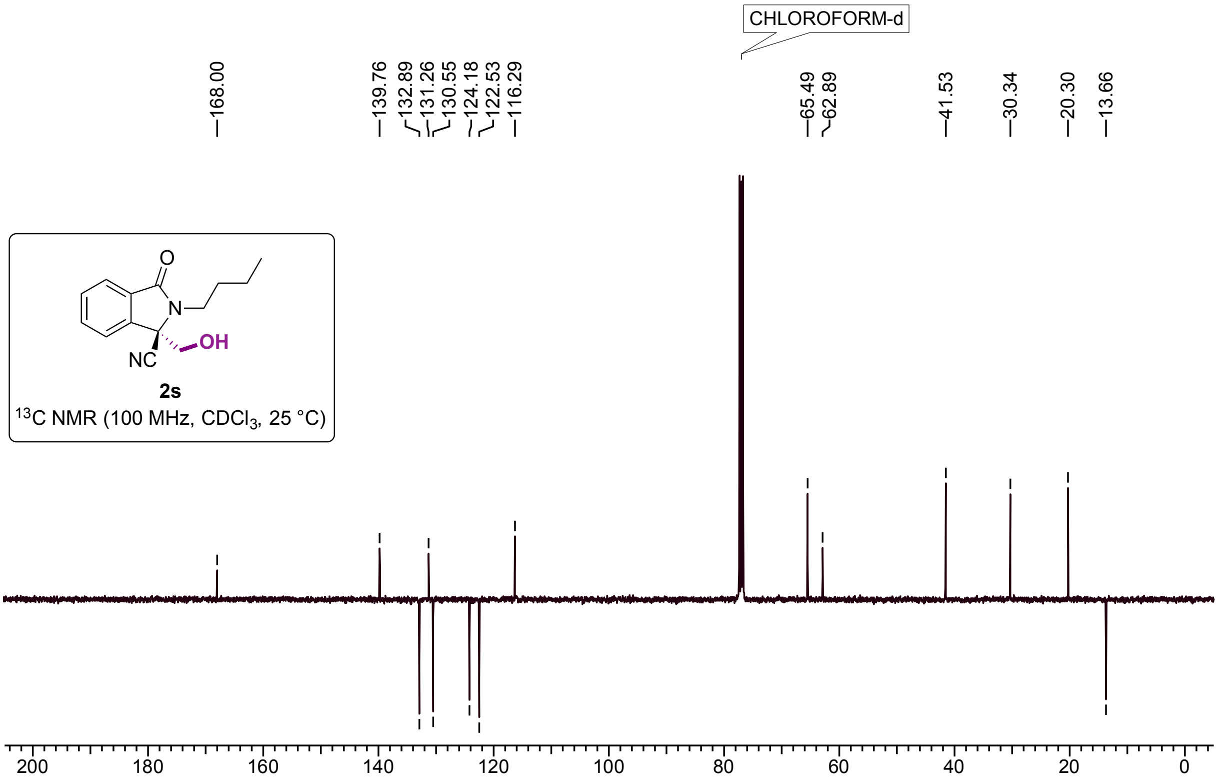

CHLOROFORM-d
168.00
139.76
132.89
131.26
130.55
124.18
122.53
116.29
65.49
62.89
41.53
30.34
20.30
13.66
O
N
NC
OH
2s
13C NMR (100 MHz, CDCl3, 25 °C)
200
180
160
140
120
100
80
60
40
20
0

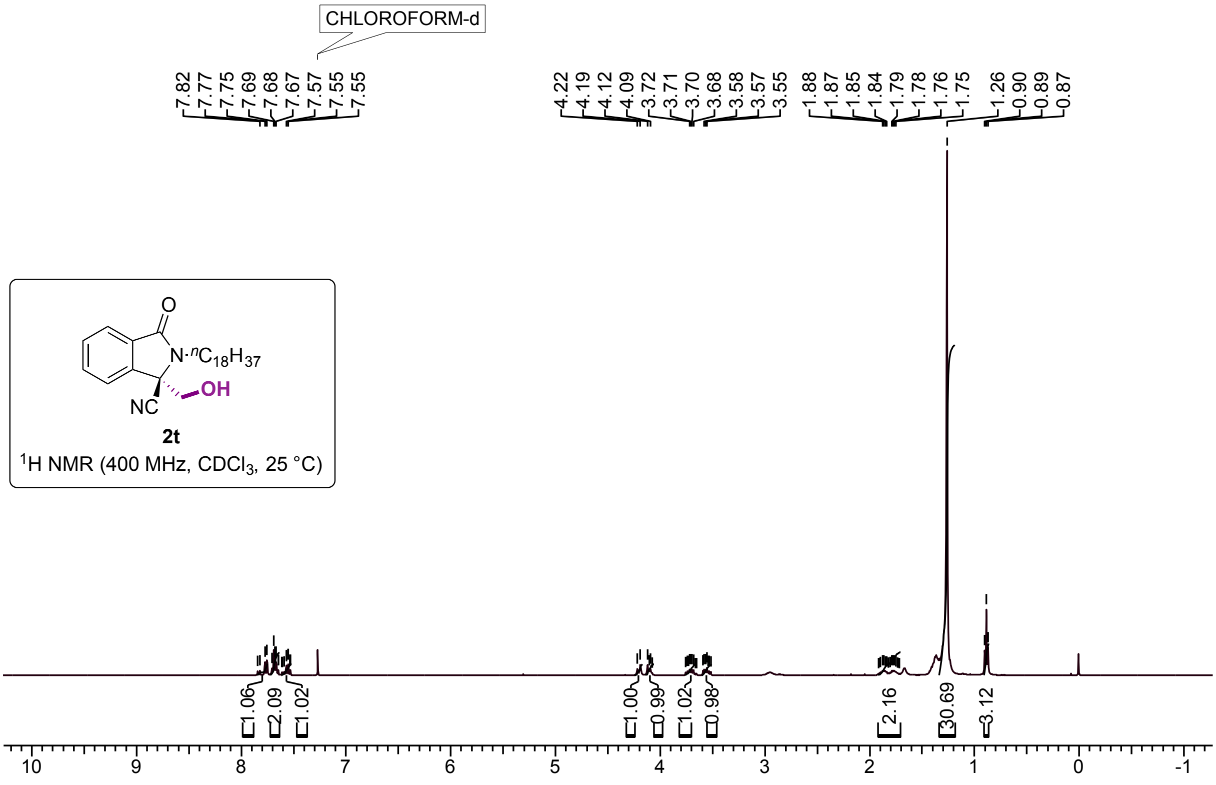

CHLOROFORM-d
7.82
7.77
7.75
7.69
7.68
7.67
7.57
7.55
7.55
4.22
4.19
4.12
4.09
3.72
3.71
3.70
3.68
3.58
3.57
3.55
1.88
1.87
1.85
1.84
1.79
1.78
1.76
1.75
1.26
0.90
0.89
0.87
O
N-nC18H37
NC
OH
2t
1H NMR (400 MHz, CDCl3, 25 °C)
1.06
2.09
1.02
1.00
0.99
1.02
0.98
2.16
30.69
3.12
10
9
8
7
6
5
4
3
2
1
0
-1

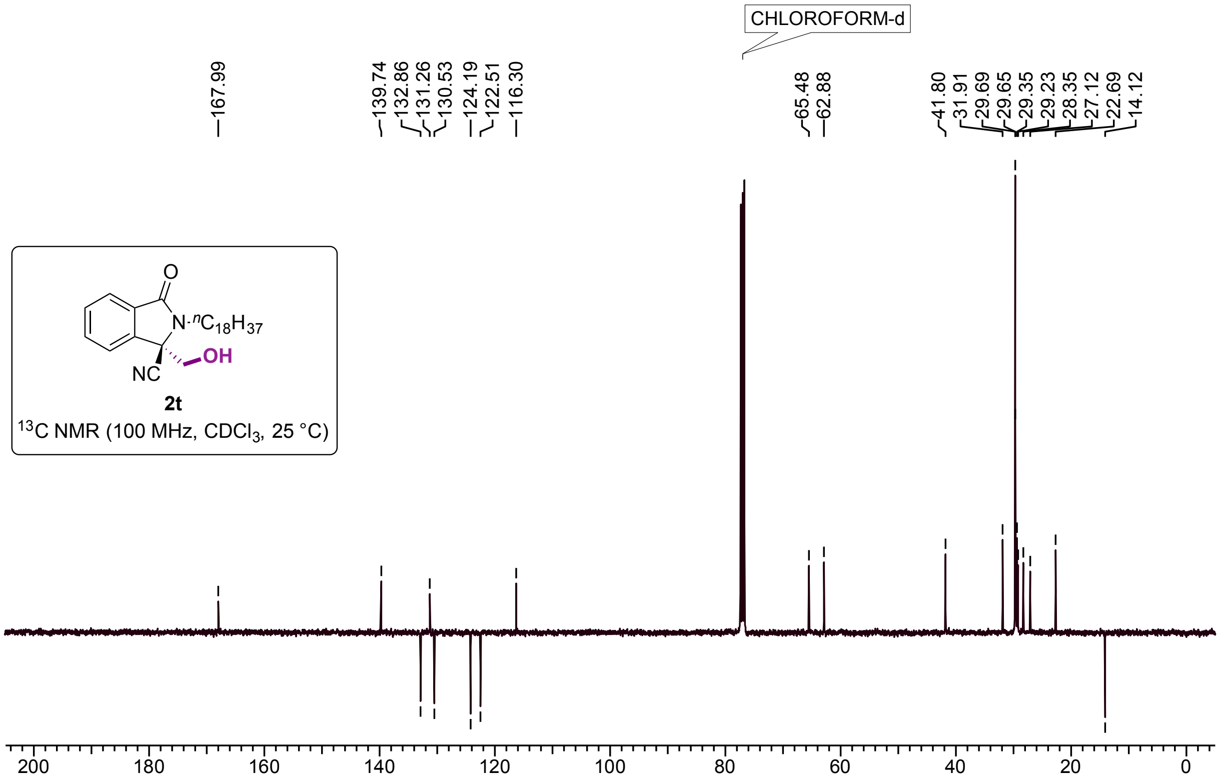
CHLOROFORM-d
167.99
139.74
132.86
131.26
130.53
124.19
122.51
116.30
65.48
62.88
41.80
31.91
29.69
29.65
29.35
29.23
28.35
27.12
22.69
14.12
2t
13C NMR (100 MHz, CDCl3, 25 °C)
200
180
160
140
120
100
80
60
40
20
0

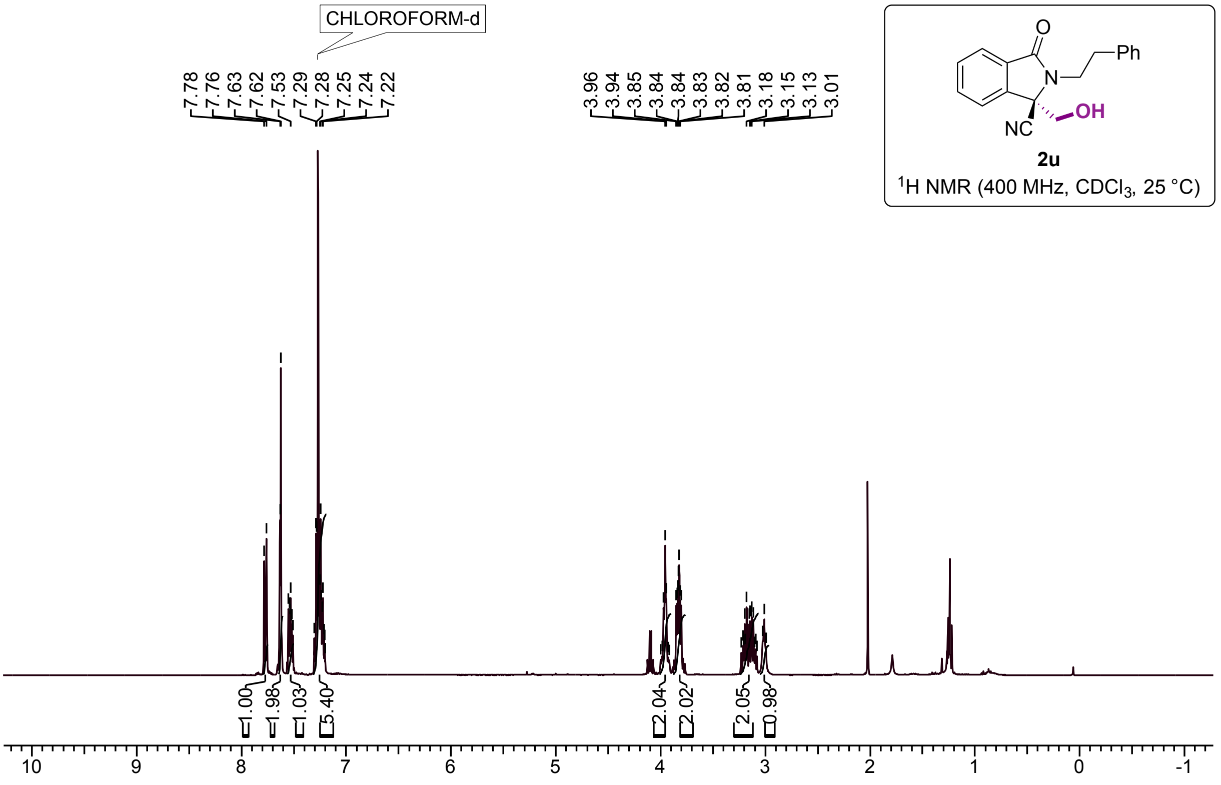
CHLOROFORM-d
7.78
7.76
7.63
7.62
7.53
7.29
7.28
7.25
7.24
7.22
3.96
3.94
3.85
3.84
3.84
3.83
3.82
3.81
3.18
3.15
3.13
3.01
2u
1H NMR (400 MHz, CDCl3, 25 °C)
1.00
1.98
1.03
5.40
2.04
2.02
2.05
0.98
10
9
8
7
6
5
4
3
2
1
0
-1

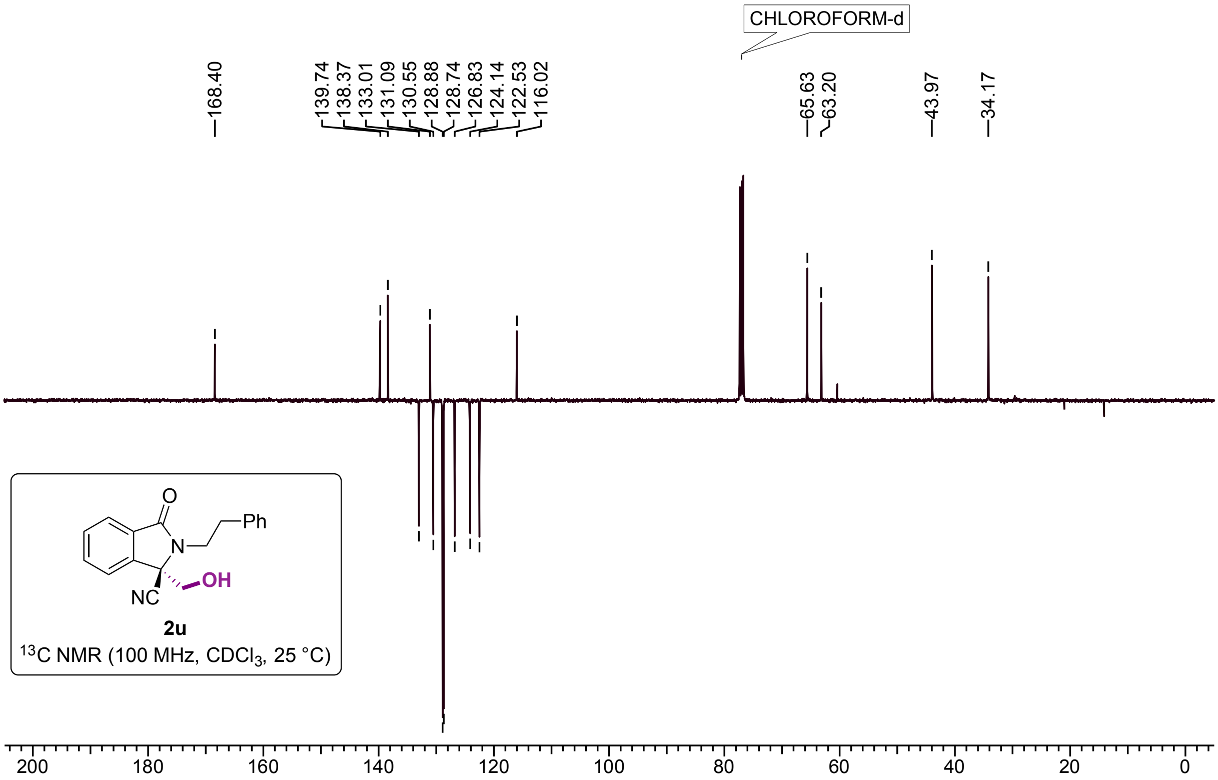
CHLOROFORM-d
168.40
139.74
138.37
133.01
131.09
130.55
128.88
128.74
126.83
124.14
122.53
116.02
65.63
63.20
43.97
34.17
O
N
Ph
NC
OH
2u
^{13}C NMR (100 MHz, $CDCl_3$, 25 °C)
200
180
160
140
120
100
80
60
40
20
0

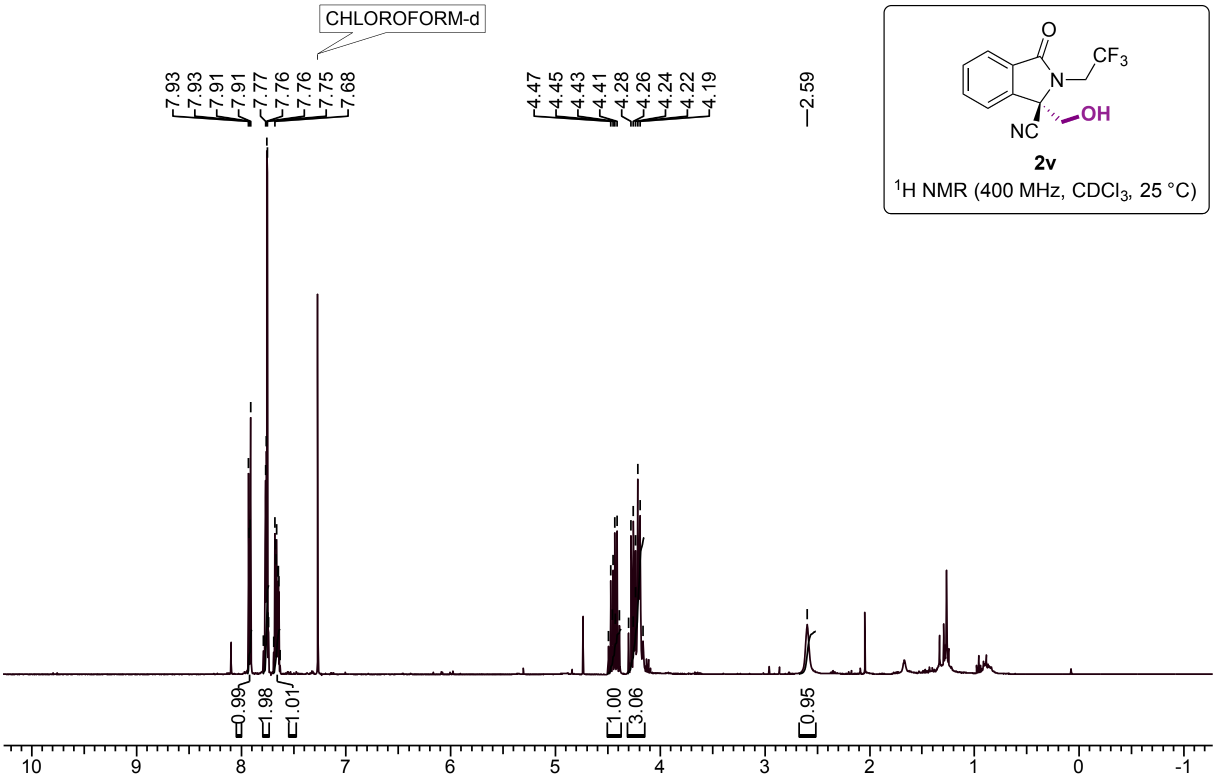
CHLOROFORM-d
7.93
7.93
7.91
7.91
7.77
7.76
7.76
7.75
7.68
4.47
4.45
4.43
4.41
4.28
4.26
4.24
4.22
4.19
2.59
O
CF_3
N
NC
OH
2v
^{1}H NMR (400 MHz, $CDCl_3$, 25 °C)
0.99
1.98
1.01
1.00
3.06
0.95
10
9
8
7
6
5
4
3
2
1
0
-1

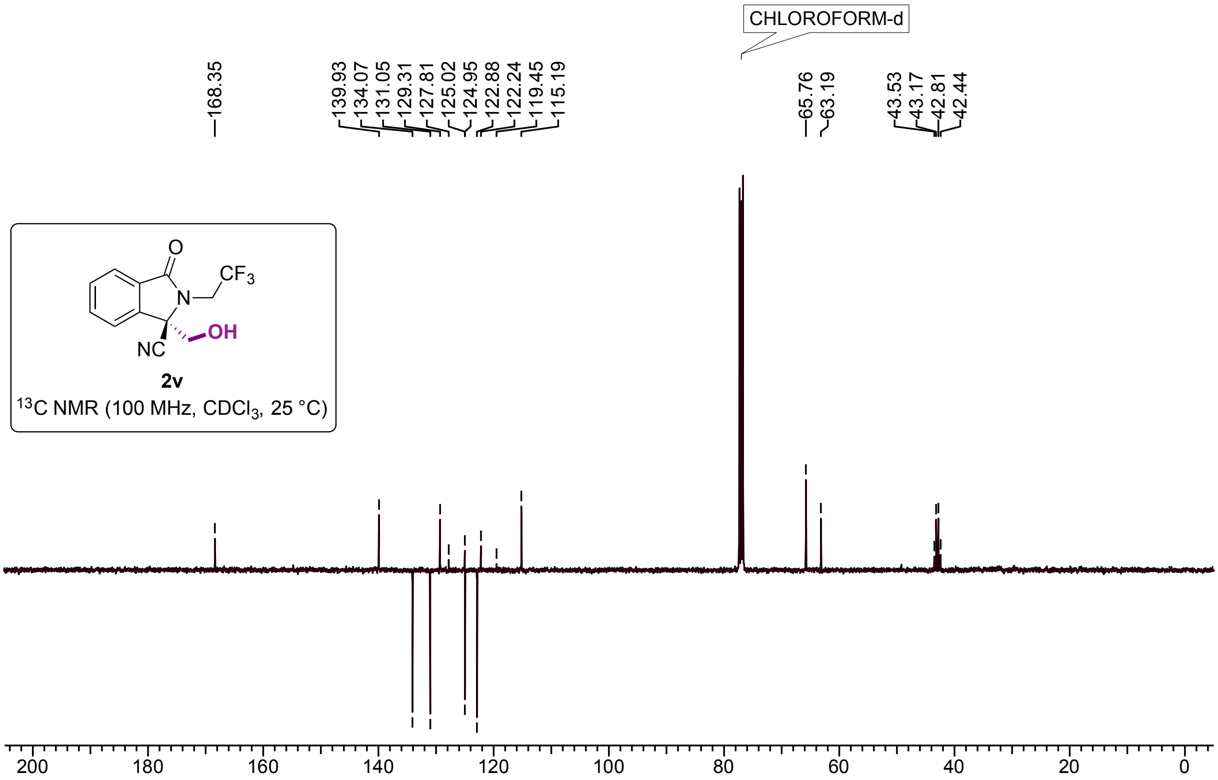
CHLOROFORM-d
168.35
139.93
134.07
131.05
129.31
127.81
125.02
124.95
122.88
122.24
119.45
115.19
65.76
63.19
43.53
43.17
42.81
42.44
O
CF3
N
OH
NC
2v
13C NMR (100 MHz, CDCl3, 25 °C)
200
180
160
140
120
100
80
60
40
20
0

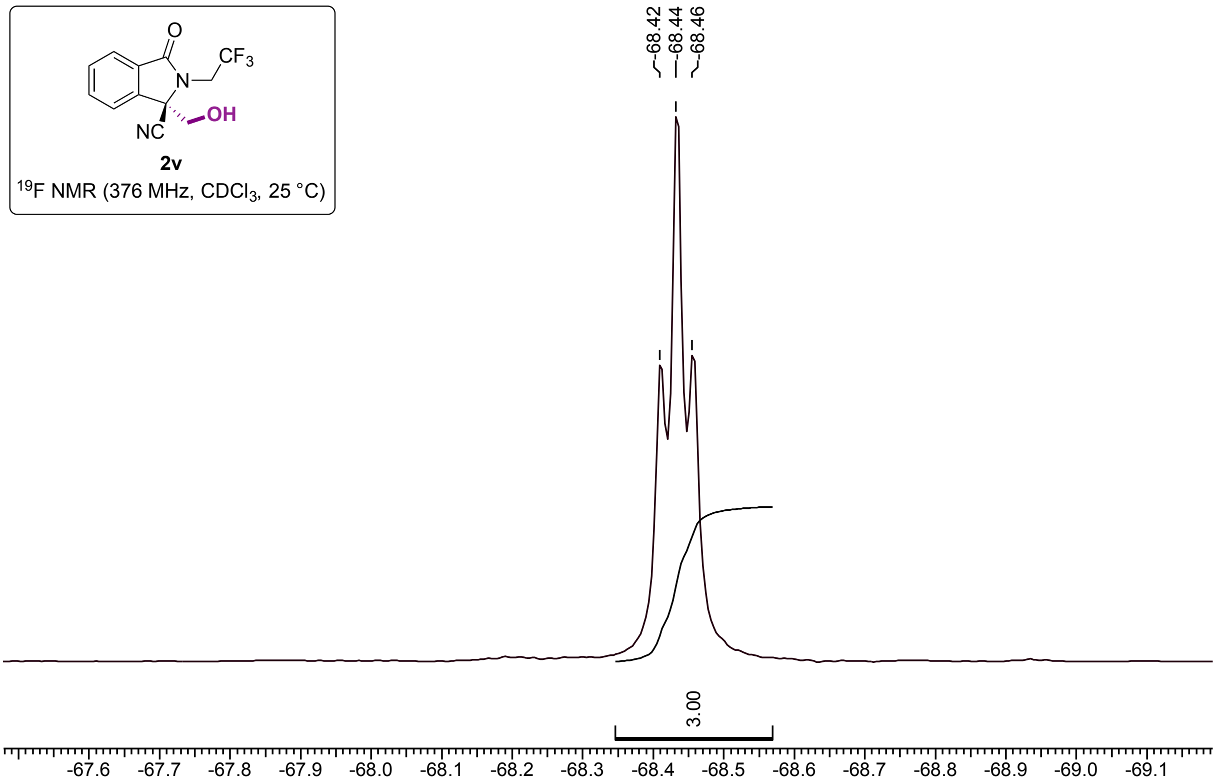
-68.42
-68.44
-68.46
O
CF3
N
OH
NC
2v
19F NMR (376 MHz, CDCl3, 25 °C)
3.00
-67.6
-67.7
-67.8
-67.9
-68.0
-68.1
-68.2
-68.3
-68.4
-68.5
-68.6
-68.7
-68.8
-68.9
-69.0
-69.1

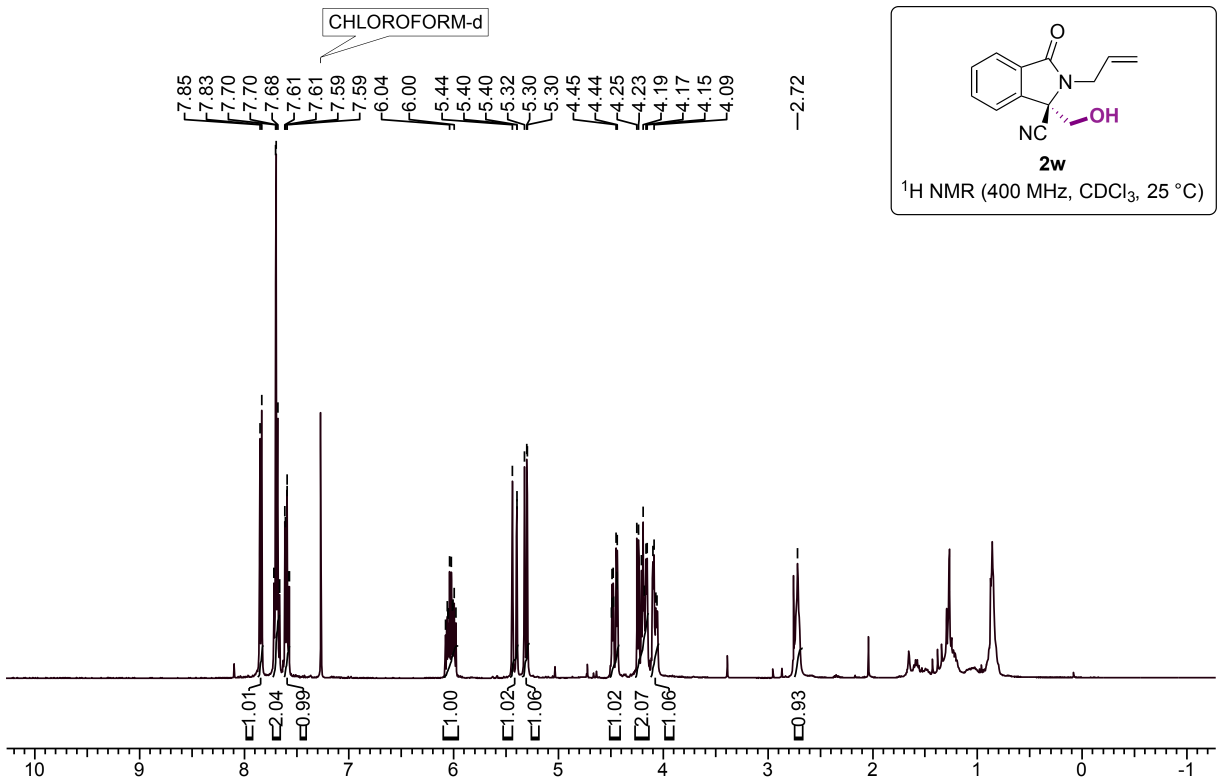
CHLOROFORM-d
7.85 7.83 7.70 7.70 7.68 7.61 7.61 7.59 7.59 6.04 6.00 5.44 5.40 5.40 5.32 5.30 5.30 4.45 4.44 4.25 4.23 4.19 4.17 4.15 4.09
2.72
O
N
NC
OH
2w
1H NMR (400 MHz, CDCl3, 25 °C)
1.01 2.04 0.99 1.00 1.02 1.06 1.02 2.07 1.06 0.93
10 9 8 7 6 5 4 3 2 1 0 -1

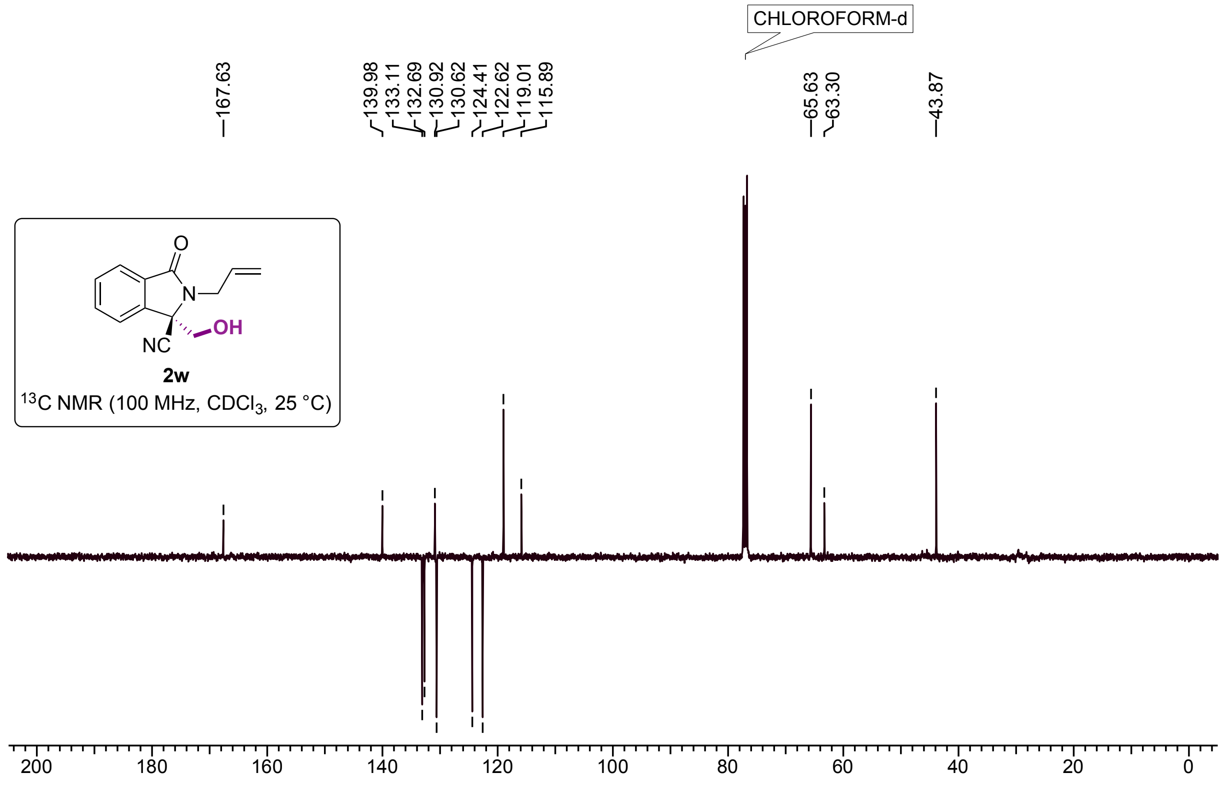
CHLOROFORM-d
167.63
139.98 133.11 132.69 130.92 130.62 124.41 122.62 119.01 115.89
65.63 63.30
43.87
O
N
NC
OH
2w
13C NMR (100 MHz, CDCl3, 25 °C)
200 180 160 140 120 100 80 60 40 20 0

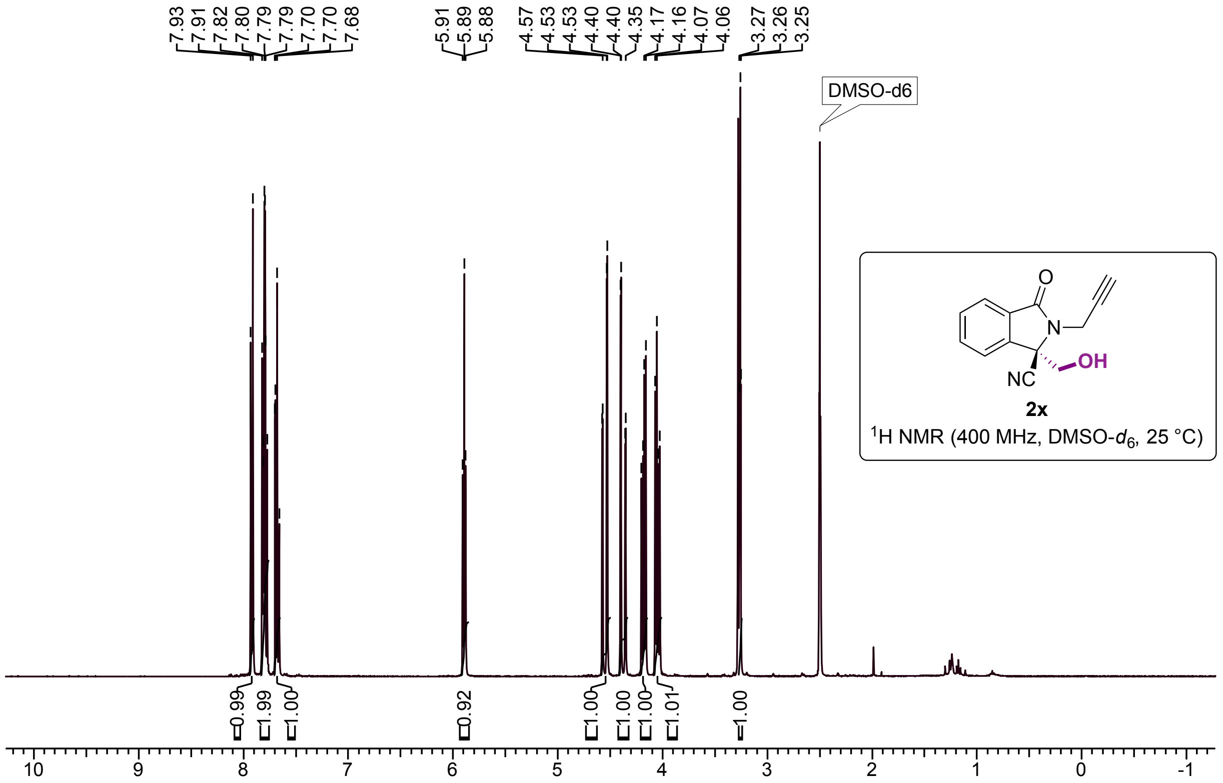
7.93 7.91 7.82 7.80 7.79 7.79 7.70 7.70 7.68
5.91 5.89 5.88
4.57 4.53 4.53 4.40 4.40 4.35 4.17 4.16 4.07 4.06
3.27 3.26 3.25
DMSO-d6
O
N
OH
NC
2x
1H NMR (400 MHz, DMSO-d6, 25 °C)
0.99 1.99 1.00 0.92 1.00 1.00 1.00 1.01 1.00
10 9 8 7 6 5 4 3 2 1 0 -1

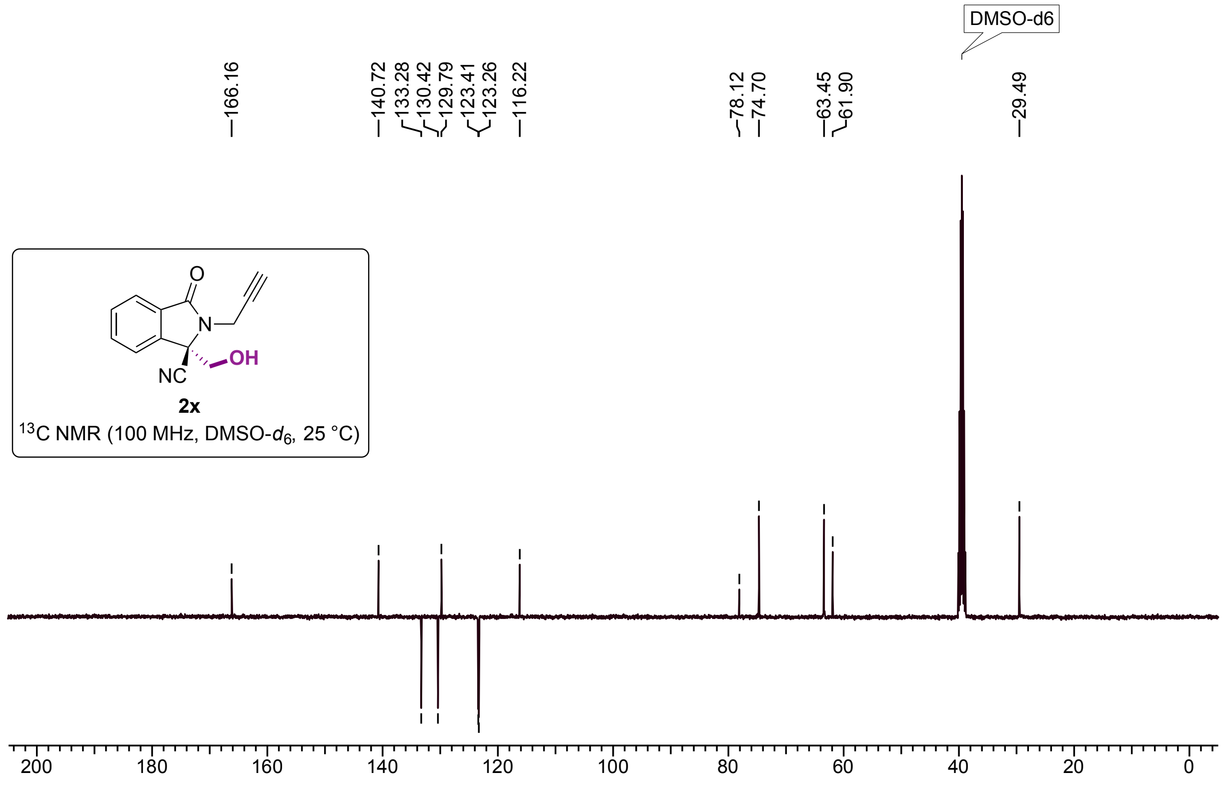
DMSO-d6
166.16
140.72 133.28 130.42 129.79 123.41 123.26 116.22
78.12 74.70
63.45 61.90
29.49
O
N
OH
NC
2x
13C NMR (100 MHz, DMSO-d6, 25 °C)
200 180 160 140 120 100 80 60 40 20 0

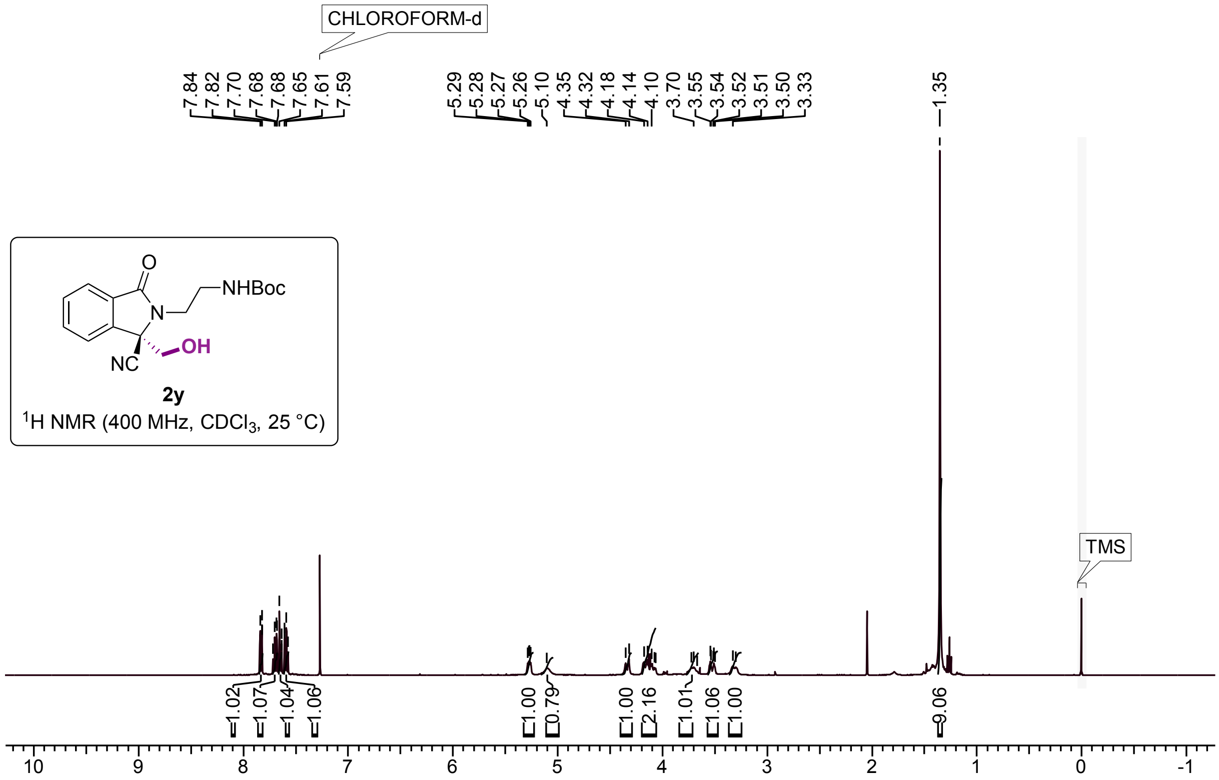
CHLOROFORM-d
7.84
7.82
7.70
7.68
7.68
7.65
7.61
7.59
5.29
5.28
5.27
5.26
5.10
4.35
4.32
4.18
4.14
4.10
3.70
3.55
3.54
3.52
3.51
3.50
3.33
1.35
TMS
O
NHBoc
N
OH
NC
2y
1H NMR (400 MHz, CDCl3, 25 °C)
1.02
1.07
1.04
1.06
1.00
0.79
1.00
2.16
1.01
1.06
1.00
9.06
10
9
8
7
6
5
4
3
2
1
0
-1

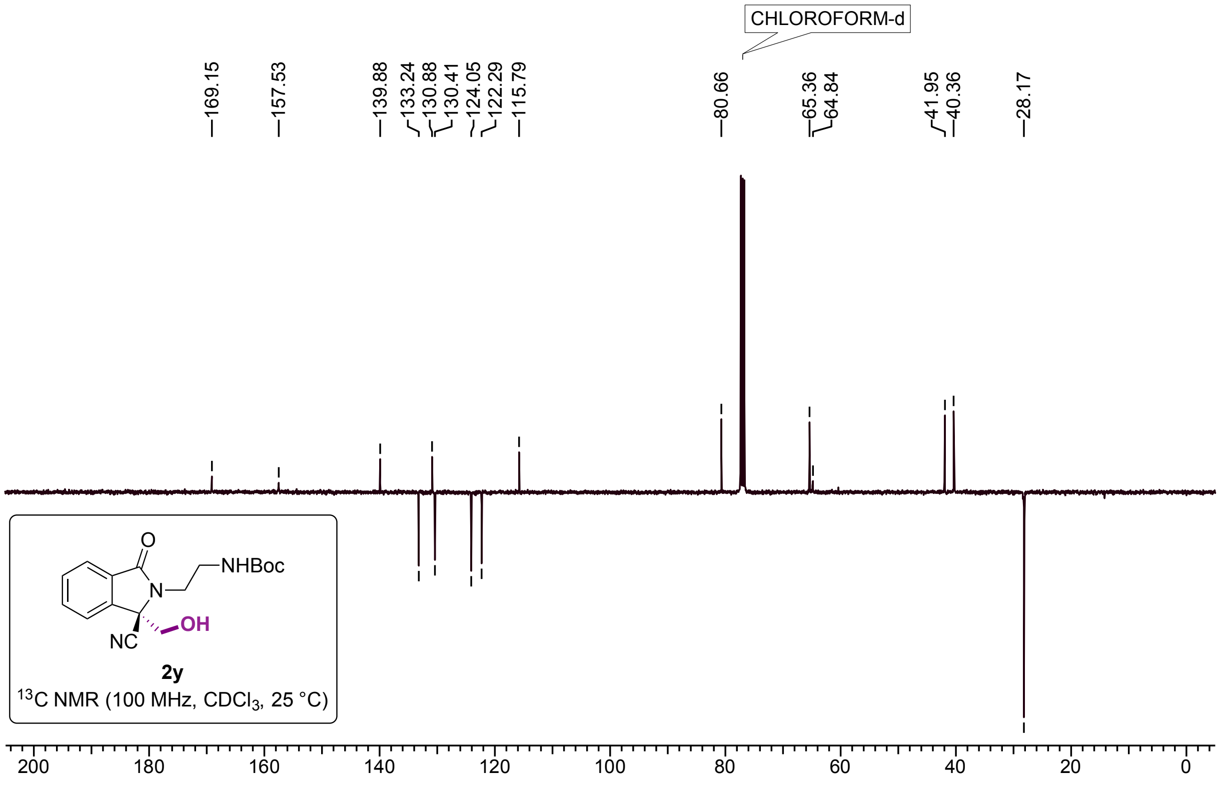
CHLOROFORM-d
169.15
157.53
139.88
133.24
130.88
130.41
124.05
122.29
115.79
80.66
65.36
64.84
41.95
40.36
28.17
O
NHBoc
N
OH
NC
2y
13C NMR (100 MHz, CDCl3, 25 °C)
200
180
160
140
120
100
80
60
40
20
0

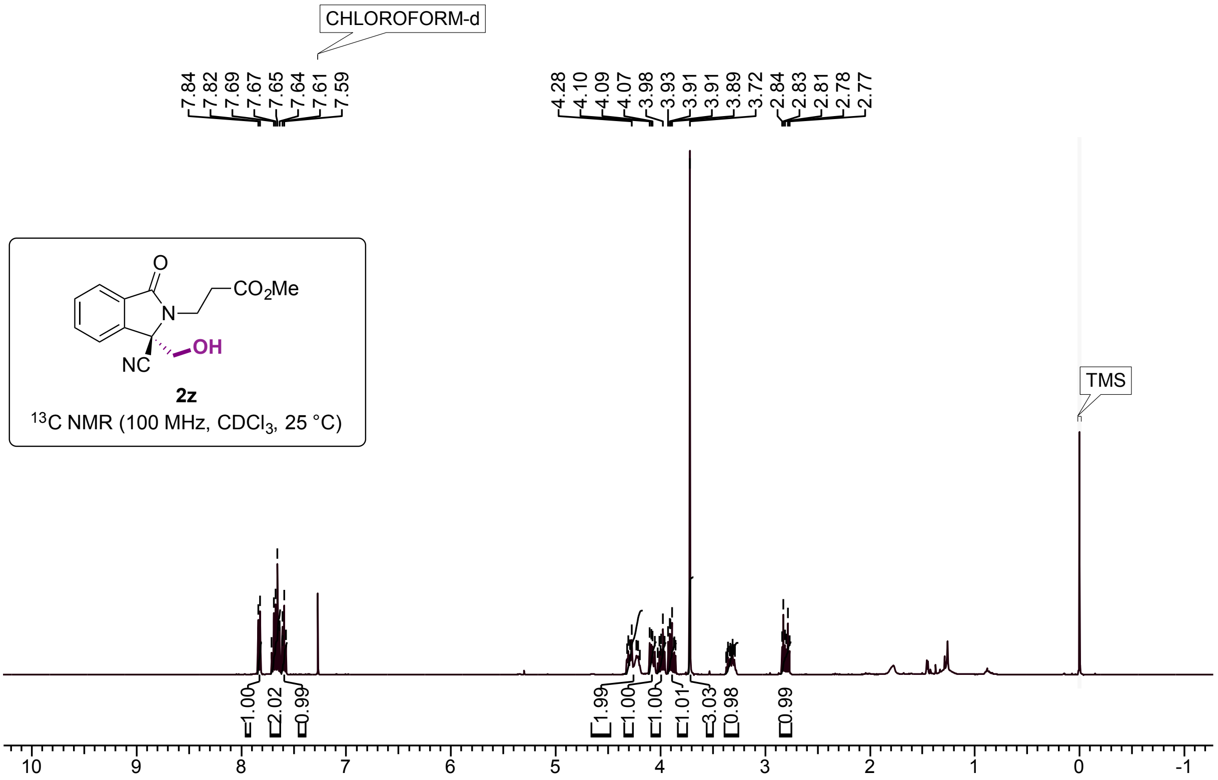
CHLOROFORM-d
7.84 7.82 7.69 7.67 7.65 7.64 7.61 7.59
4.28 4.10 4.09 4.07 3.98 3.93 3.91 3.91 3.89 3.72 2.84 2.83 2.81 2.78 2.77
O
CO_2Me
N
OH
NC
2z
^{13}C NMR (100 MHz, $CDCl_3$, 25 °C)
TMS
1.00 2.02 0.99
1.99 1.00 1.00 1.01 3.03 0.98 0.99
10 9 8 7 6 5 4 3 2 1 0 -1

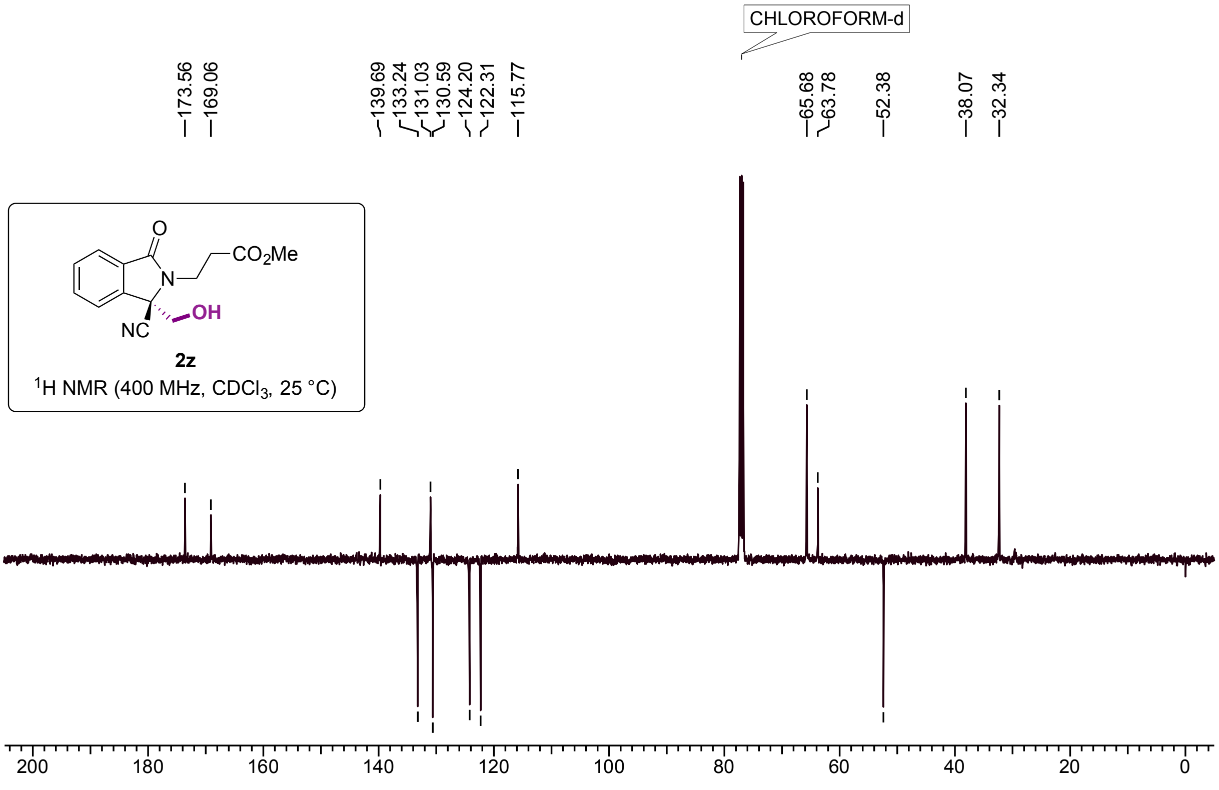
CHLOROFORM-d
173.56 169.06
139.69 133.24 131.03 130.59 124.20 122.31 115.77
65.68 63.78 52.38 38.07 32.34
O
CO_2Me
N
OH
NC
2z
^1H NMR (400 MHz, $CDCl_3$, 25 °C)
200 180 160 140 120 100 80 60 40 20 0

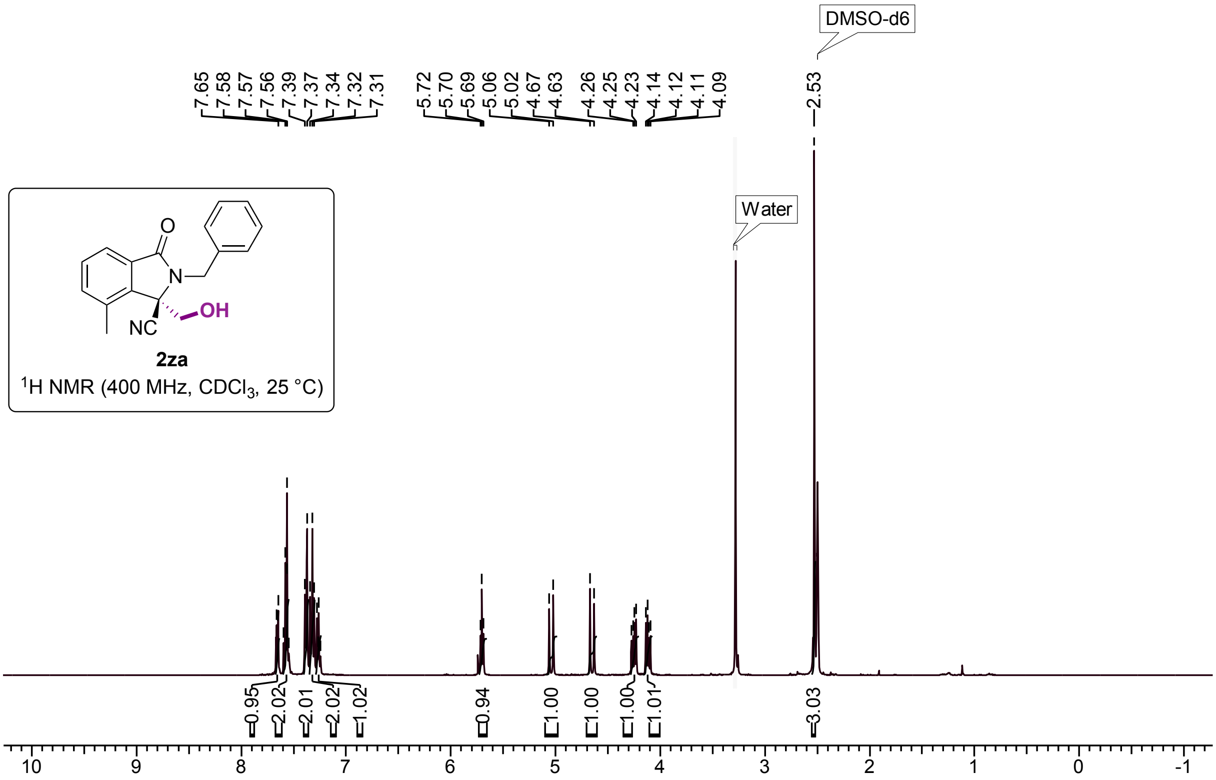
7.65
7.58
7.57
7.56
7.39
7.37
7.34
7.32
7.31
5.72
5.70
5.69
5.06
5.02
4.67
4.63
4.26
4.25
4.23
4.14
4.12
4.11
4.09
2.53
DMSO-d6
Water
O
N
OH
NC
2za
^{1}H NMR (400 MHz, $CDCl_3$, 25 °C)
0.95
2.02
2.01
2.02
1.02
0.94
1.00
1.00
1.00
1.01
3.03
10
9
8
7
6
5
4
3
2
1
0
-1

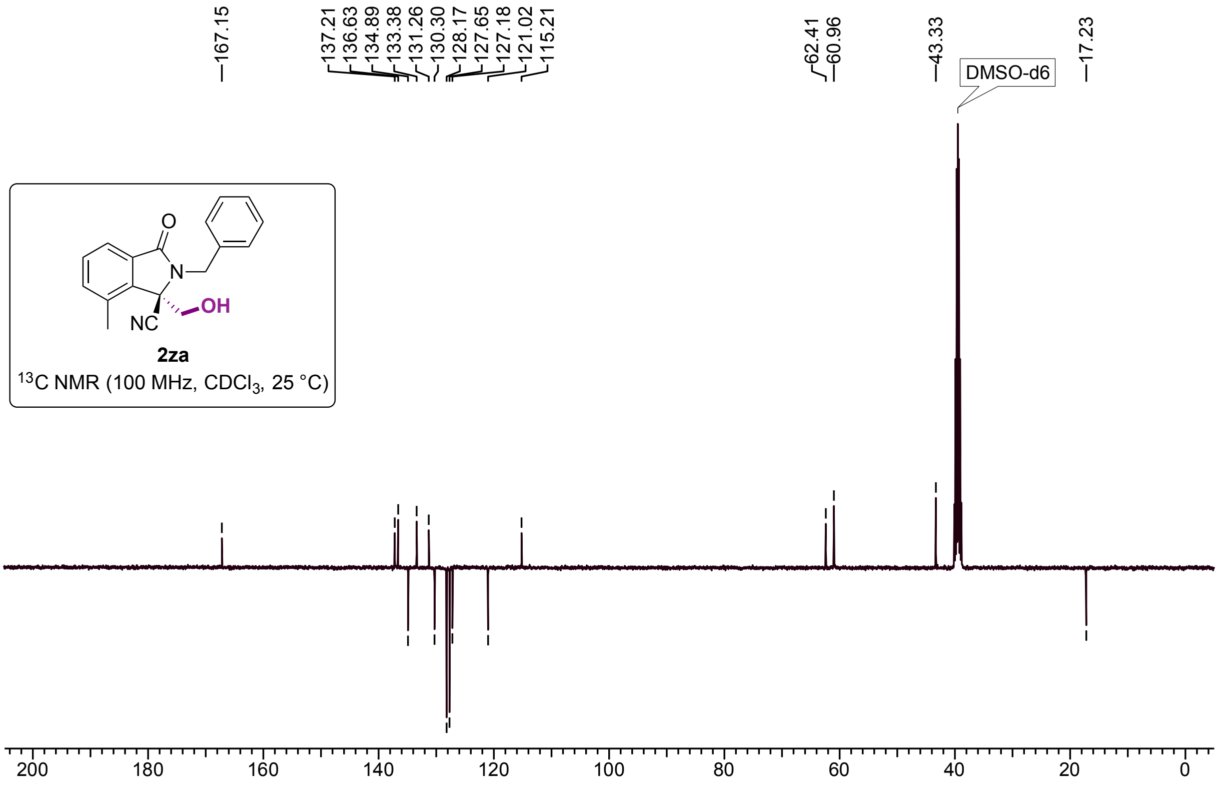
167.15
137.21
136.63
134.89
133.38
131.26
130.30
128.17
127.65
127.18
121.02
115.21
62.41
60.96
43.33
DMSO-d6
17.23
O
N
OH
NC
2za
^{13}C NMR (100 MHz, $CDCl_3$, 25 °C)
200
180
160
140
120
100
80
60
40
20
0

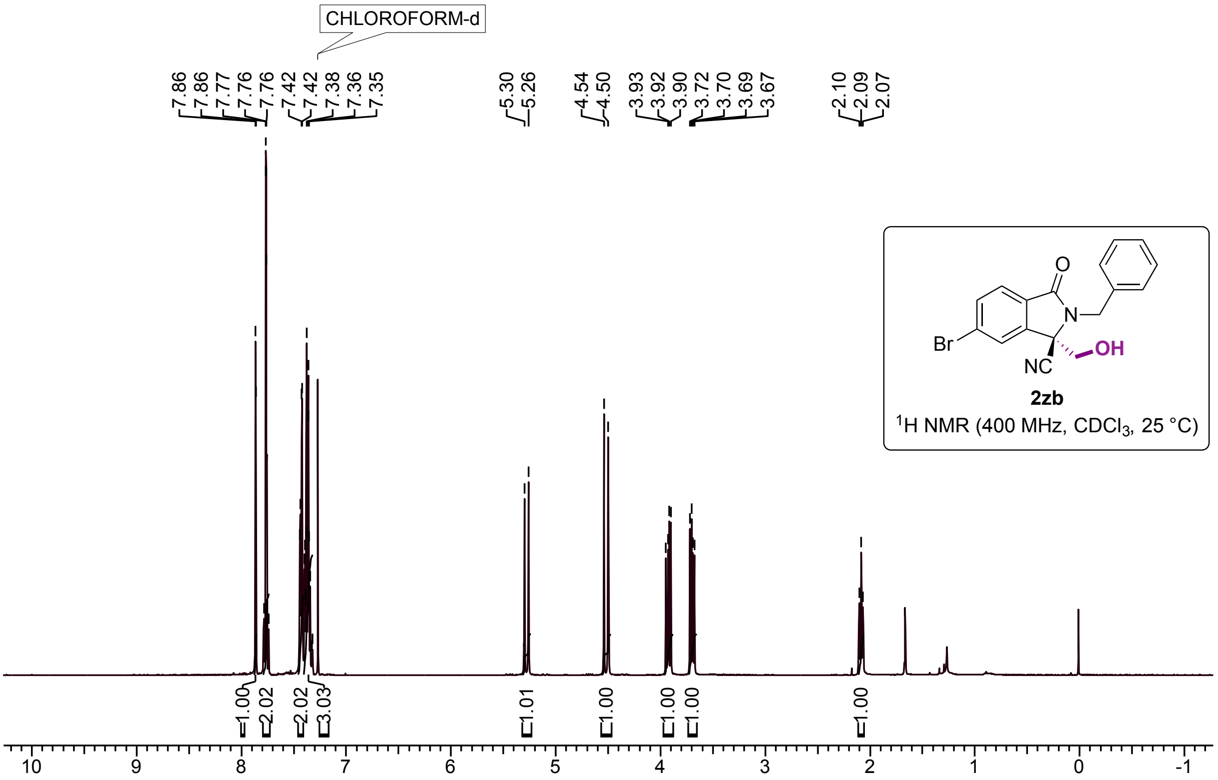
CHLOROFORM-d
7.86
7.86
7.77
7.76
7.76
7.42
7.42
7.38
7.36
7.35
5.30
5.26
4.54
4.50
3.93
3.92
3.90
3.72
3.70
3.69
3.67
2.10
2.09
2.07
O
N
Br
OH
NC
2zb
1H NMR (400 MHz, CDCl3, 25 °C)
1.00
2.02
2.02
3.03
1.01
1.00
1.00
1.00
1.00
10
9
8
7
6
5
4
3
2
1
0
-1

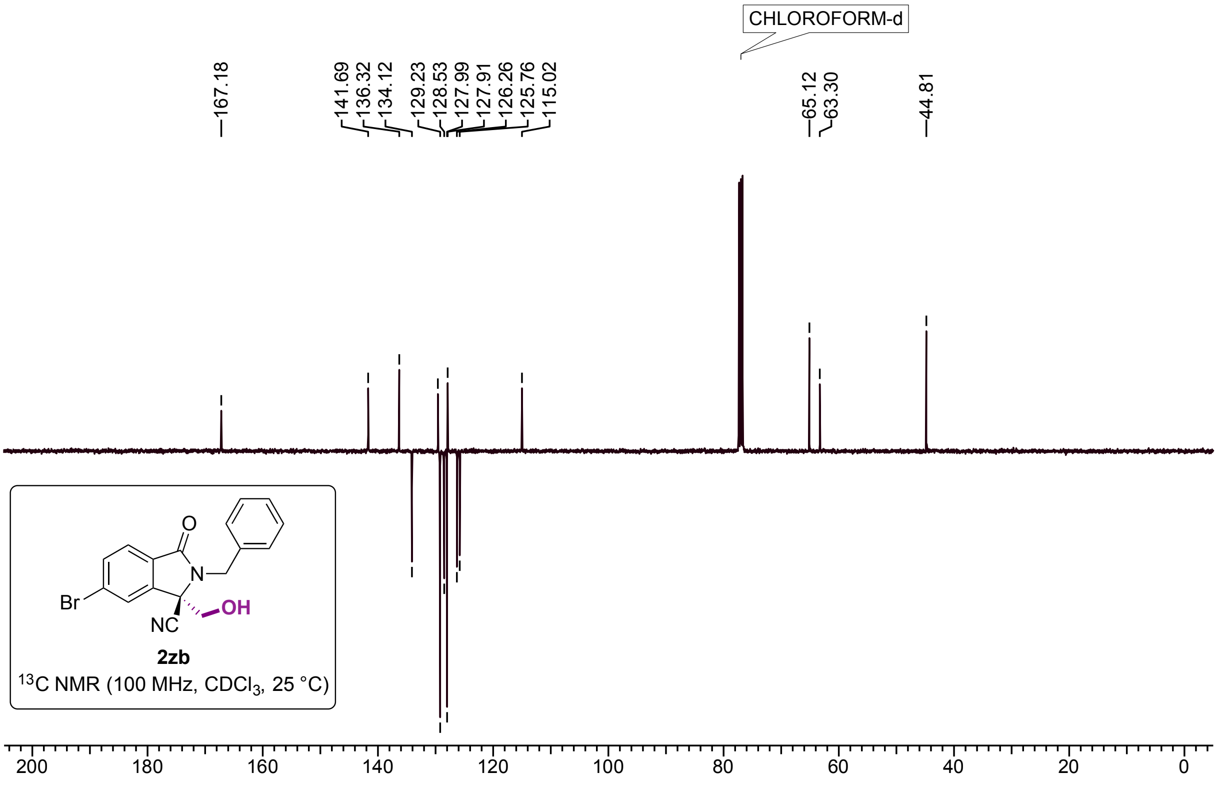
CHLOROFORM-d
167.18
141.69
136.32
134.12
129.23
128.53
127.99
127.91
126.26
125.76
115.02
65.12
63.30
44.81
O
N
Br
OH
NC
2zb
13C NMR (100 MHz, CDCl3, 25 °C)
200
180
160
140
120
100
80
60
40
20
0

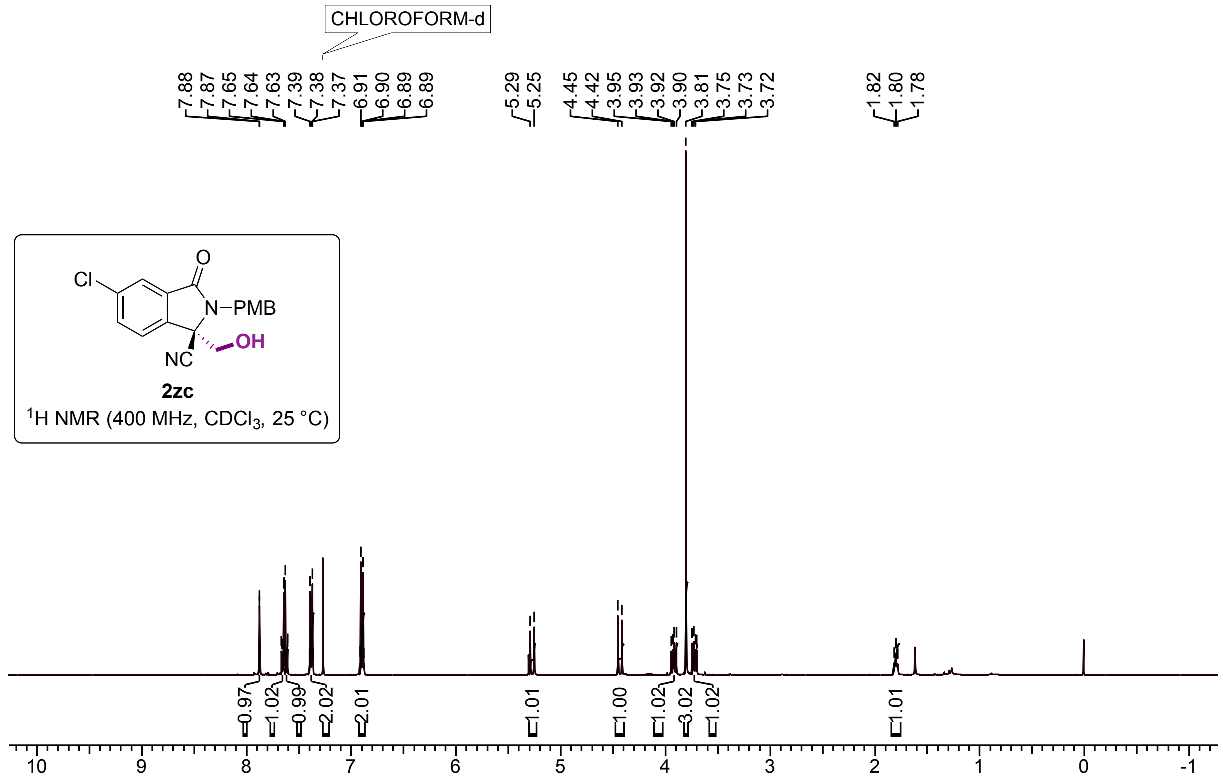
CHLOROFORM-d
7.88
7.87
7.65
7.64
7.63
7.39
7.38
7.37
6.91
6.90
6.89
6.89
5.29
5.25
4.45
4.42
3.95
3.93
3.92
3.90
3.81
3.75
3.73
3.72
1.82
1.80
1.78
Cl
O
N−PMB
NC
OH
2zc
^{1}H NMR (400 MHz, CDCl$_3$, 25 °C)
0.97
1.02
0.99
2.02
2.01
1.01
1.00
1.02
3.02
1.02
1.01
10
9
8
7
6
5
4
3
2
1
0
-1

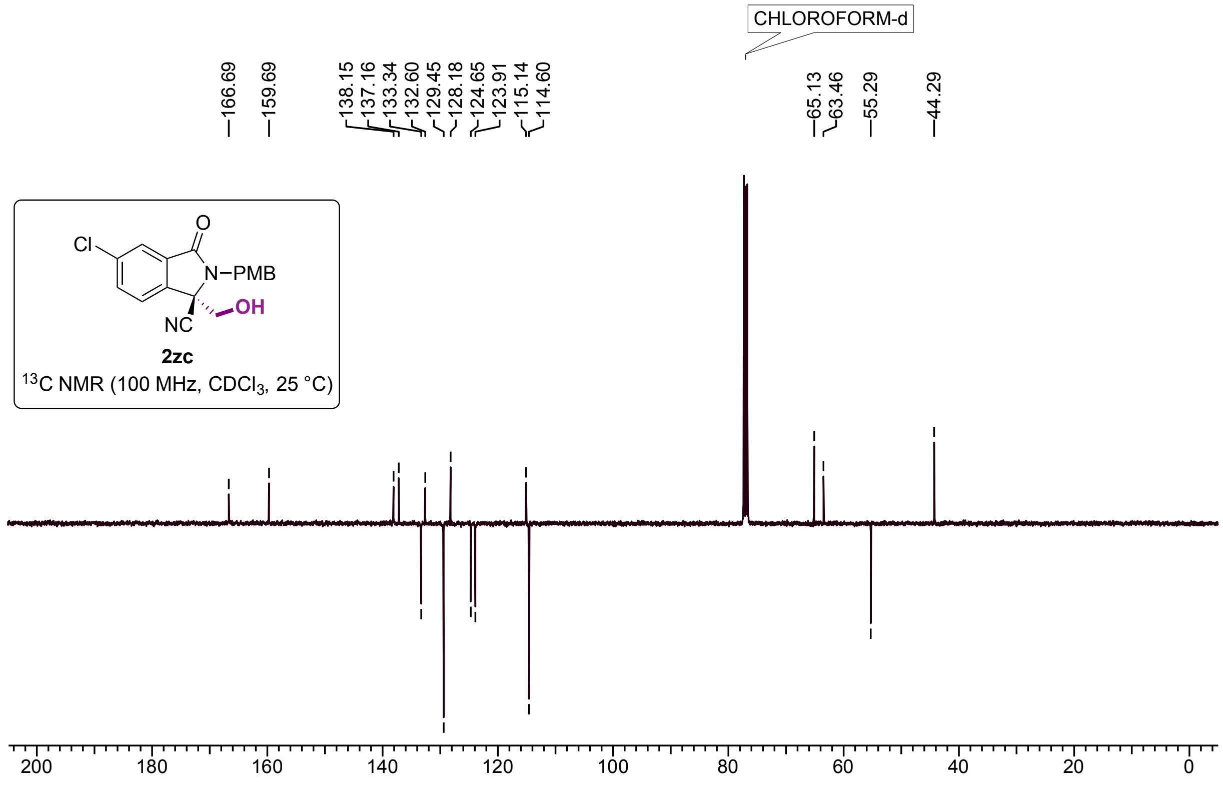
CHLOROFORM-d
166.69
159.69
138.15
137.16
133.34
132.60
129.45
128.18
124.65
123.91
115.14
114.60
65.13
63.46
55.29
44.29
Cl
O
N−PMB
NC
OH
2zc
^{13}C NMR (100 MHz, CDCl$_3$, 25 °C)
200
180
160
140
120
100
80
60
40
20
0

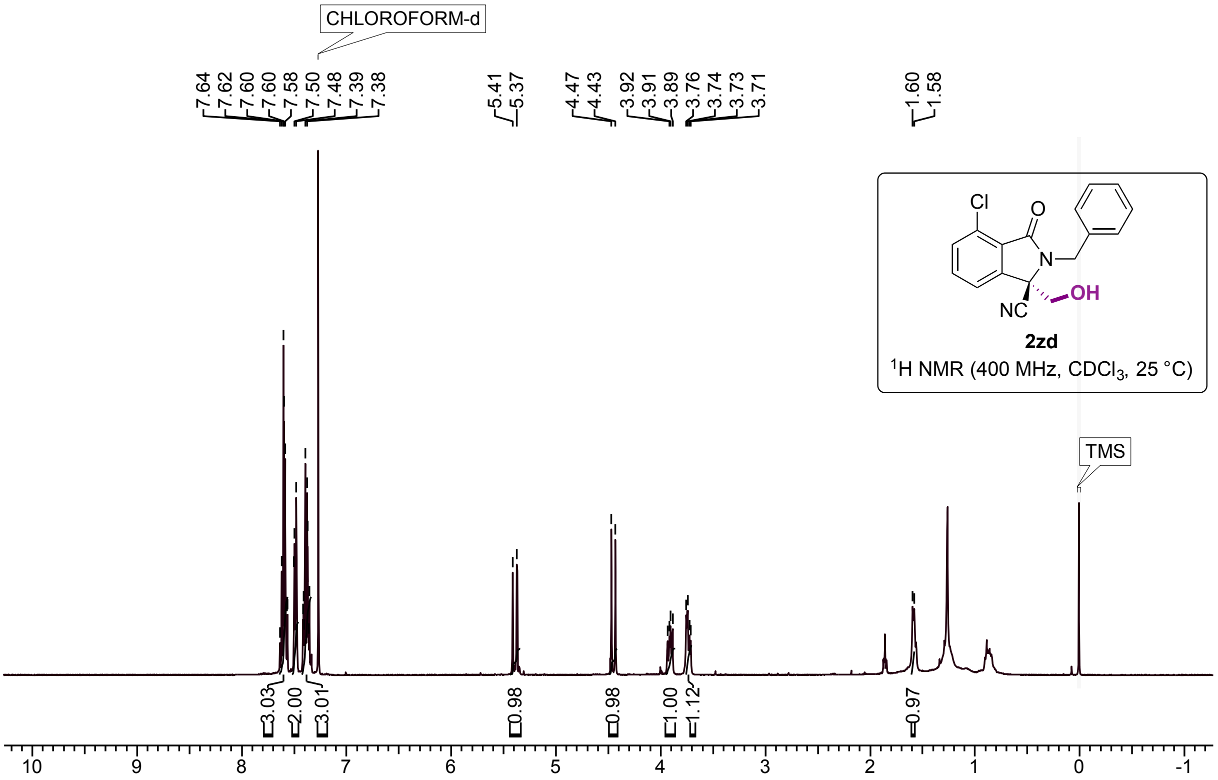
CHLOROFORM-d
7.64
7.62
7.60
7.60
7.58
7.50
7.48
7.39
7.38
5.41
5.37
4.47
4.43
3.92
3.91
3.89
3.76
3.74
3.73
3.71
1.60
1.58
Cl
O
N
NC
OH
2zd
¹H NMR (400 MHz, CDCl3, 25 °C)
TMS
3.03
2.00
3.01
0.98
0.98
1.00
1.12
0.97
10
9
8
7
6
5
4
3
2
1
0
-1

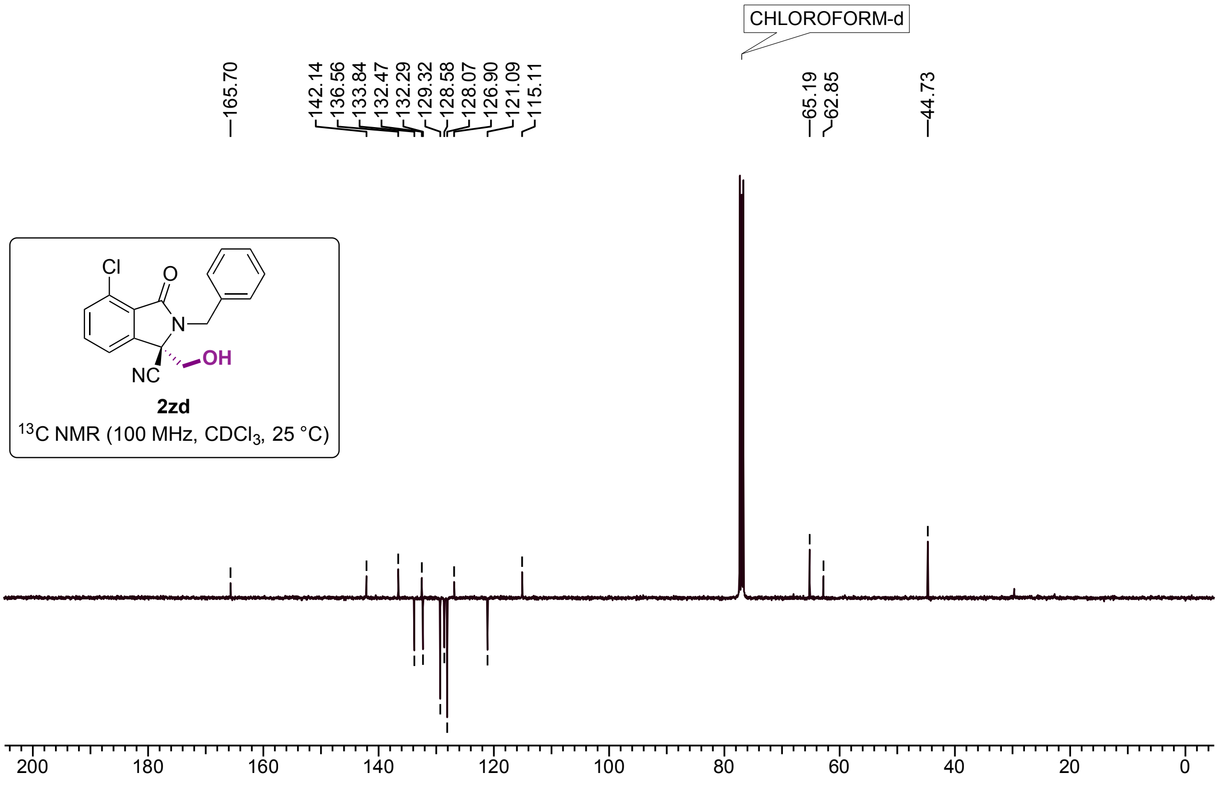
CHLOROFORM-d
165.70
142.14
136.56
133.84
132.47
132.29
129.32
128.58
128.07
126.90
121.09
115.11
65.19
62.85
44.73
Cl
O
N
NC
OH
2zd
¹³C NMR (100 MHz, CDCl3, 25 °C)
200
180
160
140
120
100
80
60
40
20
0

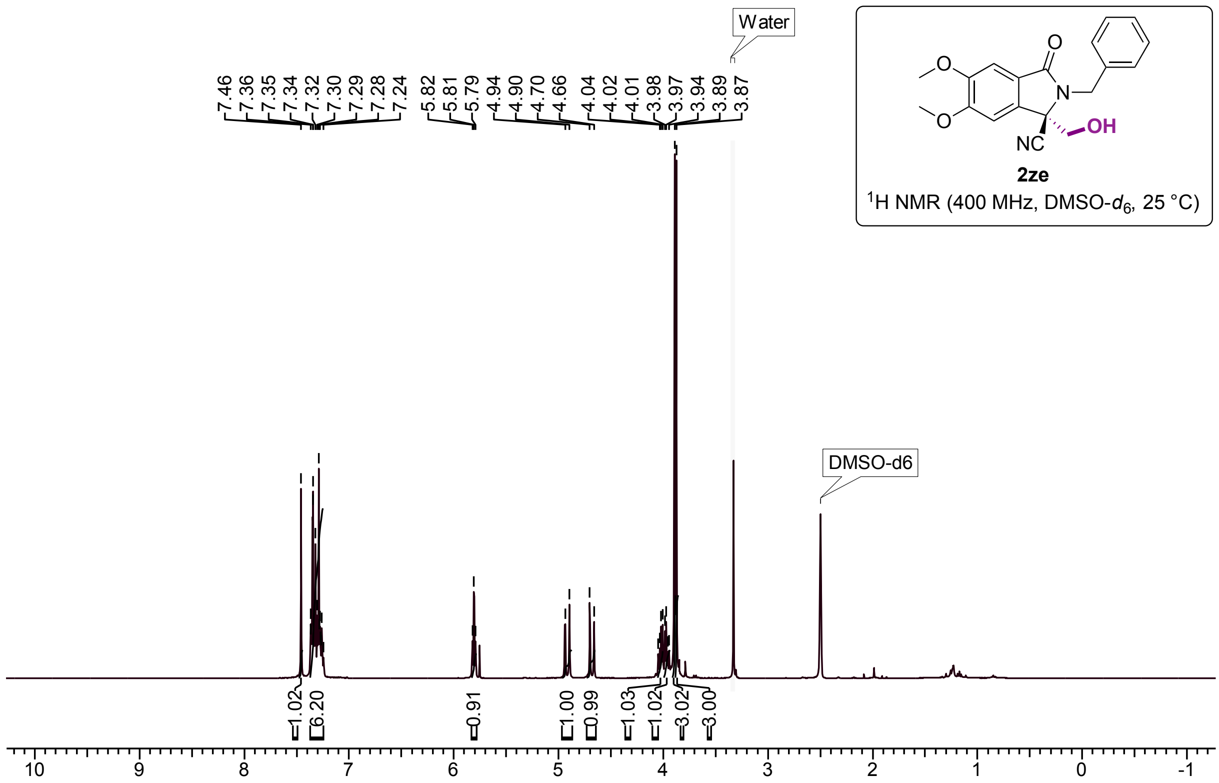
7.46
7.36
7.35
7.34
7.32
7.30
7.29
7.28
7.24
5.82
5.81
5.79
4.94
4.90
4.70
4.66
4.04
4.02
4.01
3.98
3.97
3.94
3.89
3.87
Water
DMSO-d6
1.02
6.20
0.91
1.00
0.99
1.03
1.02
3.02
3.00
10
9
8
7
6
5
4
3
2
1
0
-1
O
N
O
O
NC
OH
2ze
1H NMR (400 MHz, DMSO-d6, 25 °C)

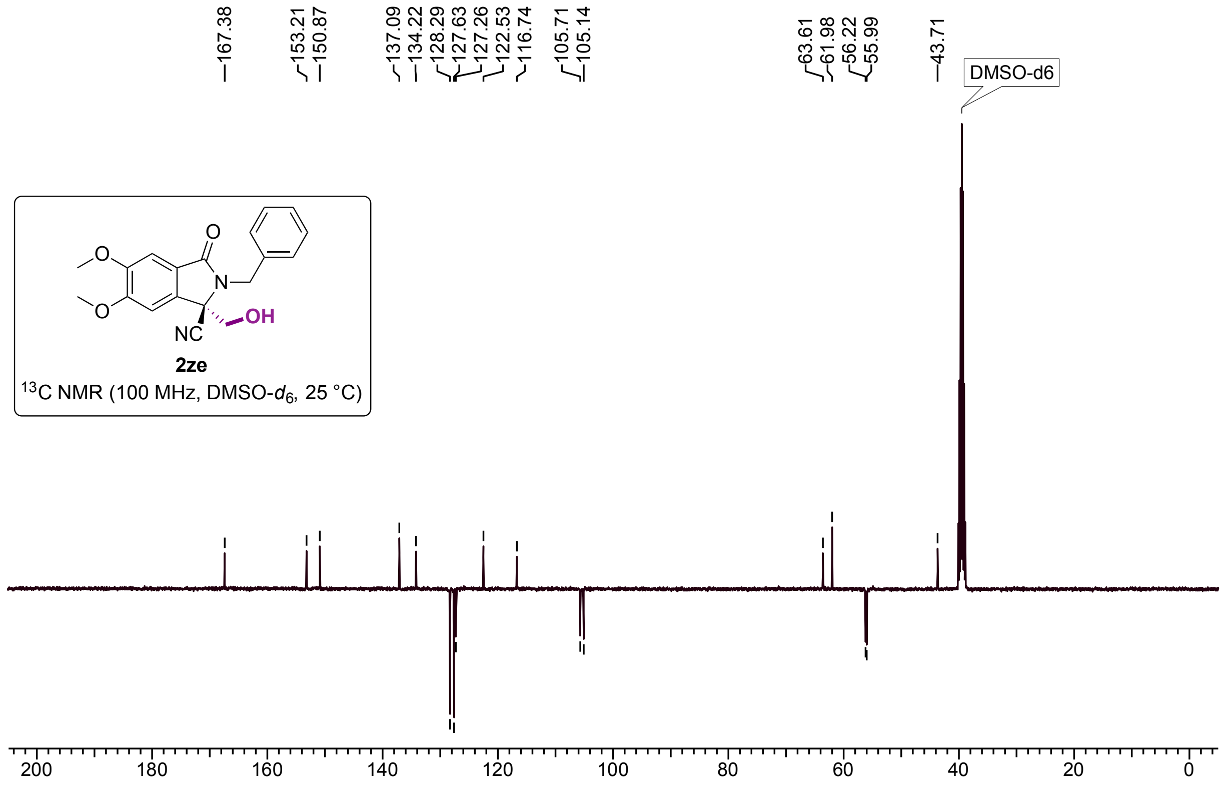
167.38
153.21
150.87
137.09
134.22
128.29
127.63
127.26
122.53
116.74
105.71
105.14
63.61
61.98
56.22
55.99
43.71
DMSO-d6
200
180
160
140
120
100
80
60
40
20
0
O
N
O
O
NC
OH
2ze
13C NMR (100 MHz, DMSO-d6, 25 °C)

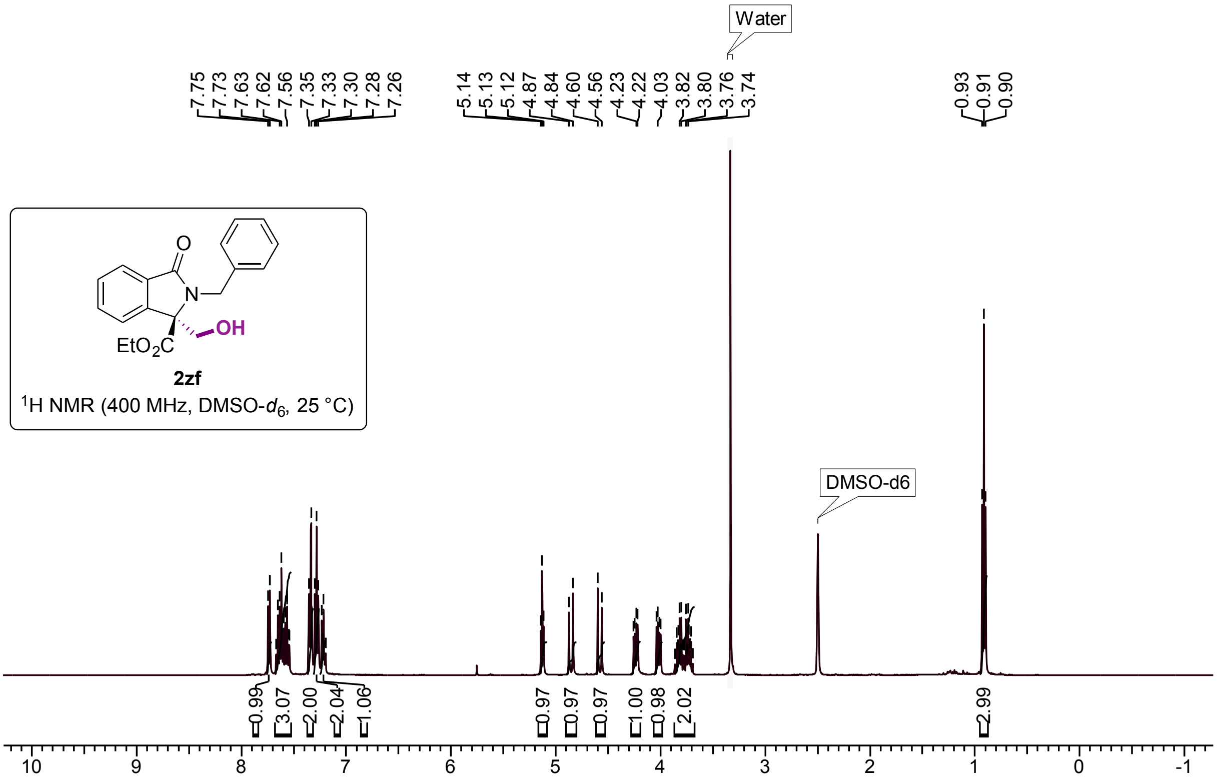
Water
7.75 7.73 7.63 7.62 7.56 7.35 7.33 7.30 7.28 7.26
5.14 5.13 5.12 4.87 4.84 4.60 4.56 4.23 4.22 4.03 3.82 3.80 3.76 3.74
0.93 0.91 0.90
O
N
OH
EtO2C
2zf
1H NMR (400 MHz, DMSO-d6, 25 °C)
DMSO-d6
0.99 3.07 2.00 2.04 1.06 0.97 0.97 0.97 1.00 0.98 2.02 2.99
10 9 8 7 6 5 4 3 2 1 0 -1

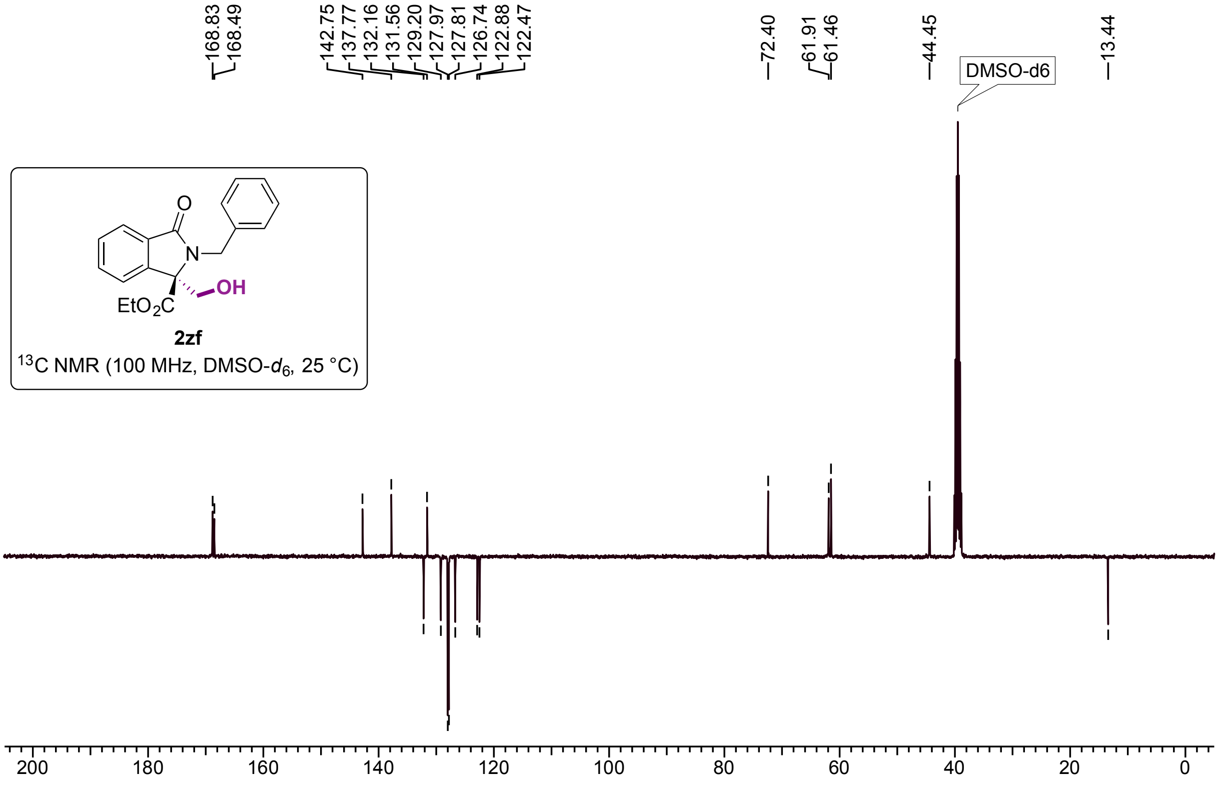
168.83 168.49
142.75 137.77 132.16 131.56 129.20 127.97 127.81 126.74 122.88 122.47
72.40 61.91 61.46 44.45 13.44
DMSO-d6
O
N
OH
EtO2C
2zf
13C NMR (100 MHz, DMSO-d6, 25 °C)
200 180 160 140 120 100 80 60 40 20 0

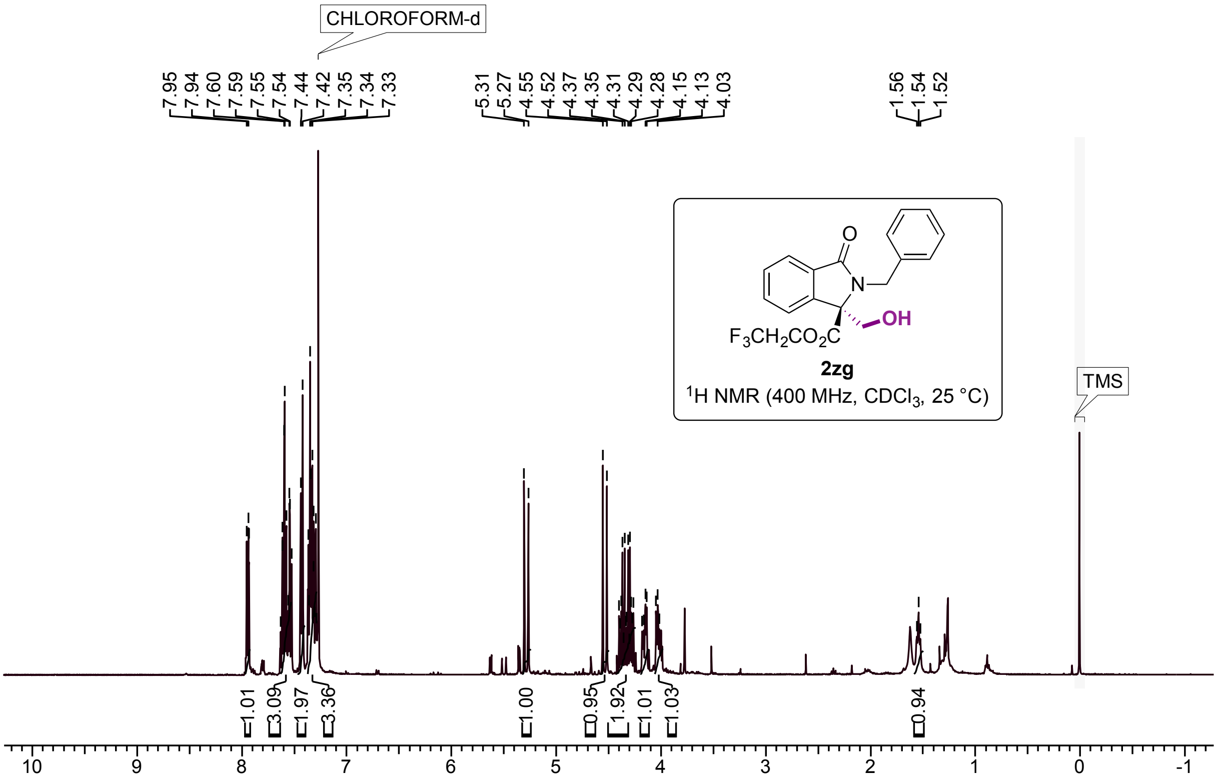
CHLOROFORM-d
7.95 7.94 7.60 7.59 7.55 7.54 7.44 7.42 7.35 7.34 7.33
5.31 5.27 4.55 4.52 4.37 4.35 4.31 4.29 4.28 4.15 4.13 4.03
1.56 1.54 1.52
O
N
OH
$F_3CH_2CO_2C$
2zg
^1H NMR (400 MHz, $CDCl_3$, 25 °C)
TMS
1.01 3.09 1.97 3.36 1.00 0.95 1.92 1.01 1.03 0.94
10 9 8 7 6 5 4 3 2 1 0 -1

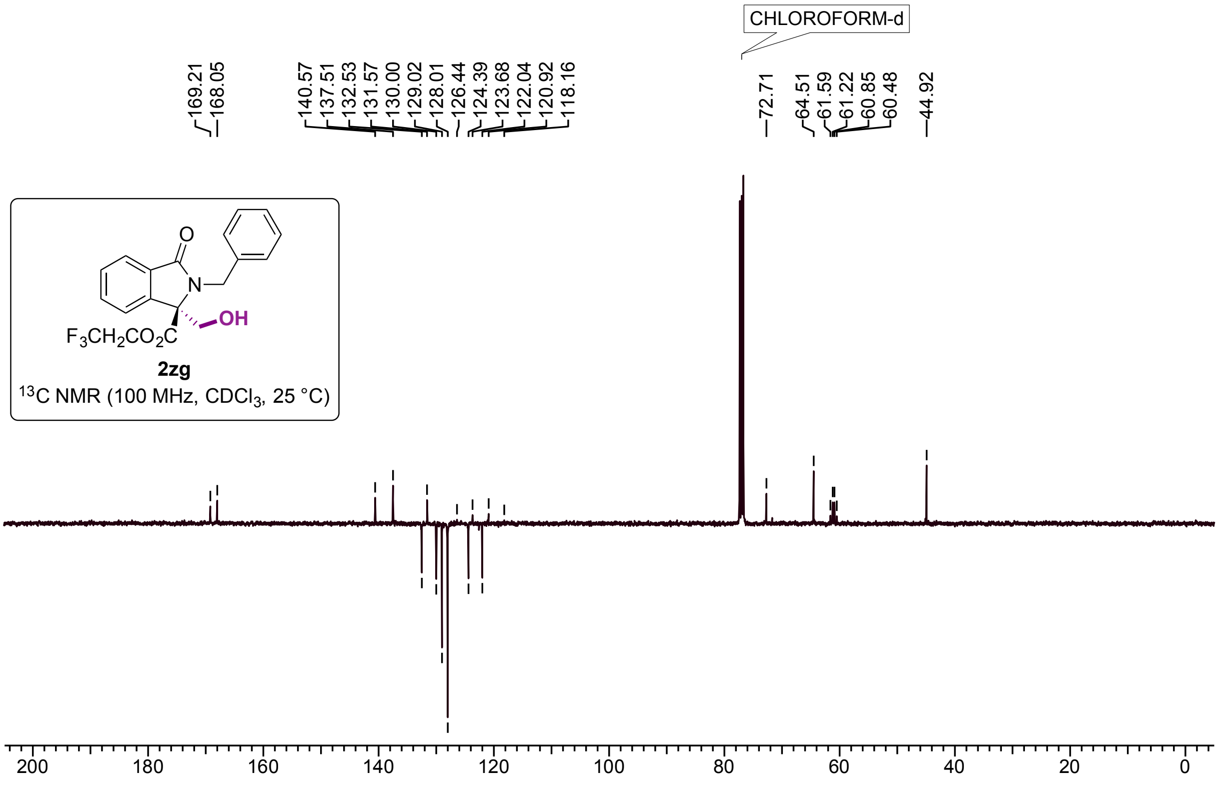
CHLOROFORM-d
169.21 168.05
140.57 137.51 132.53 131.57 130.00 129.02 128.01 126.44 124.39 123.68 122.04 120.92 118.16
72.71
64.51 61.59 61.22 60.85 60.48
44.92
O
N
OH
$F_3CH_2CO_2C$
2zg
^{13}C NMR (100 MHz, $CDCl_3$, 25 °C)
200 180 160 140 120 100 80 60 40 20 0

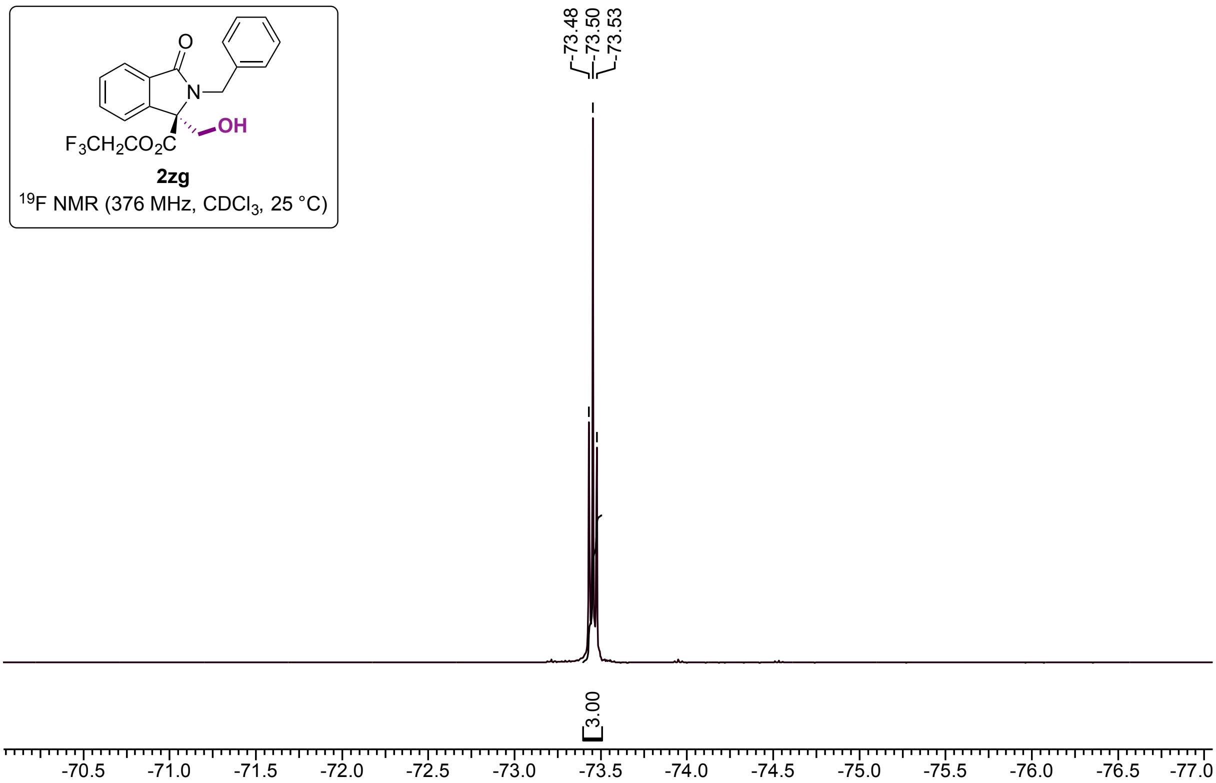
-73.48
-73.50
-73.53
F3CH2CO2C
OH
O
N
2zg
19F NMR (376 MHz, CDCl3, 25 °C)
3.00
-70.5 -71.0 -71.5 -72.0 -72.5 -73.0 -73.5 -74.0 -74.5 -75.0 -75.5 -76.0 -76.5 -77.0

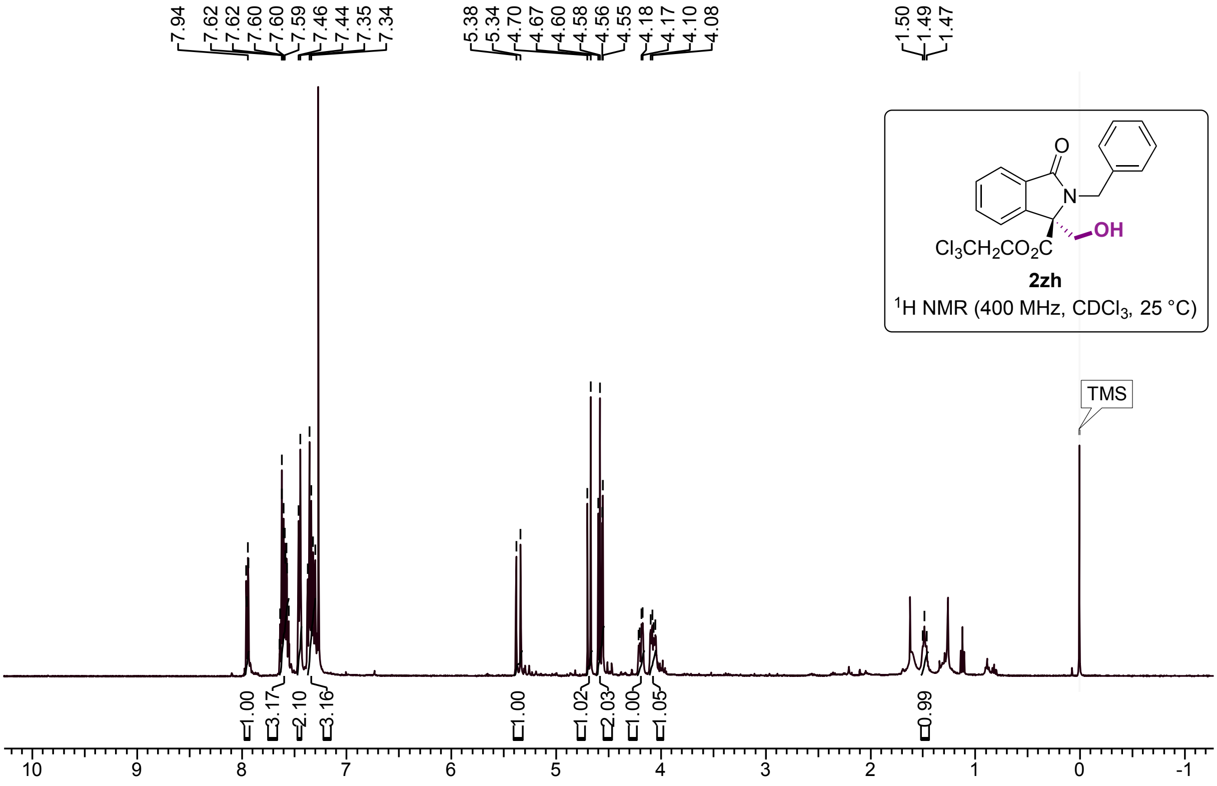
7.94 7.62 7.62 7.60 7.60 7.59 7.46 7.44 7.35 7.34
5.38 5.34 4.70 4.67 4.60 4.58 4.56 4.55 4.18 4.17 4.10 4.08
1.50 1.49 1.47
O
N
OH
Cl3CH2CO2C
2zh
1H NMR (400 MHz, CDCl3, 25 °C)
TMS
1.00 3.17 2.10 3.16 1.00 1.02 2.03 1.00 1.05 0.99
10 9 8 7 6 5 4 3 2 1 0 -1

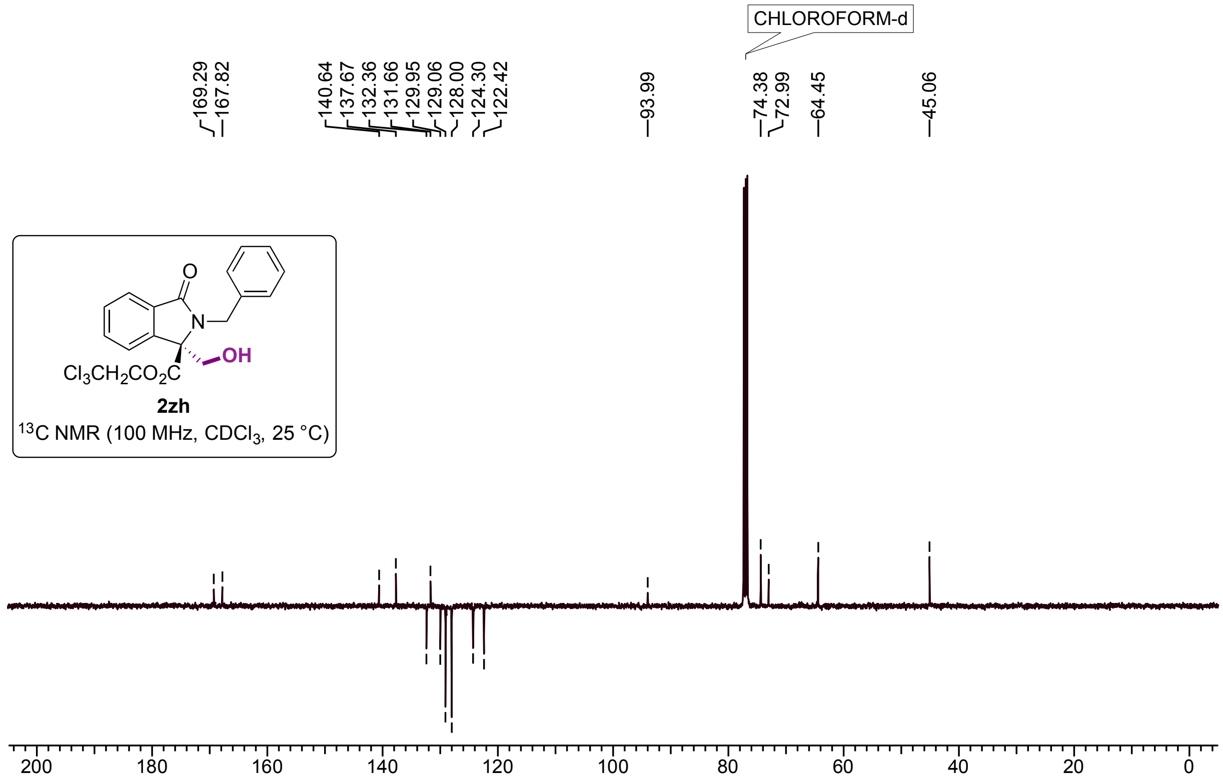
CHLOROFORM-d
169.29
167.82
140.64
137.67
132.36
131.66
129.95
129.06
128.00
124.30
122.42
93.99
74.38
72.99
64.45
45.06
2zh
^{13}C NMR (100 MHz, CDCl$_3$, 25 °C)
200
180
160
140
120
100
80
60
40
20
0

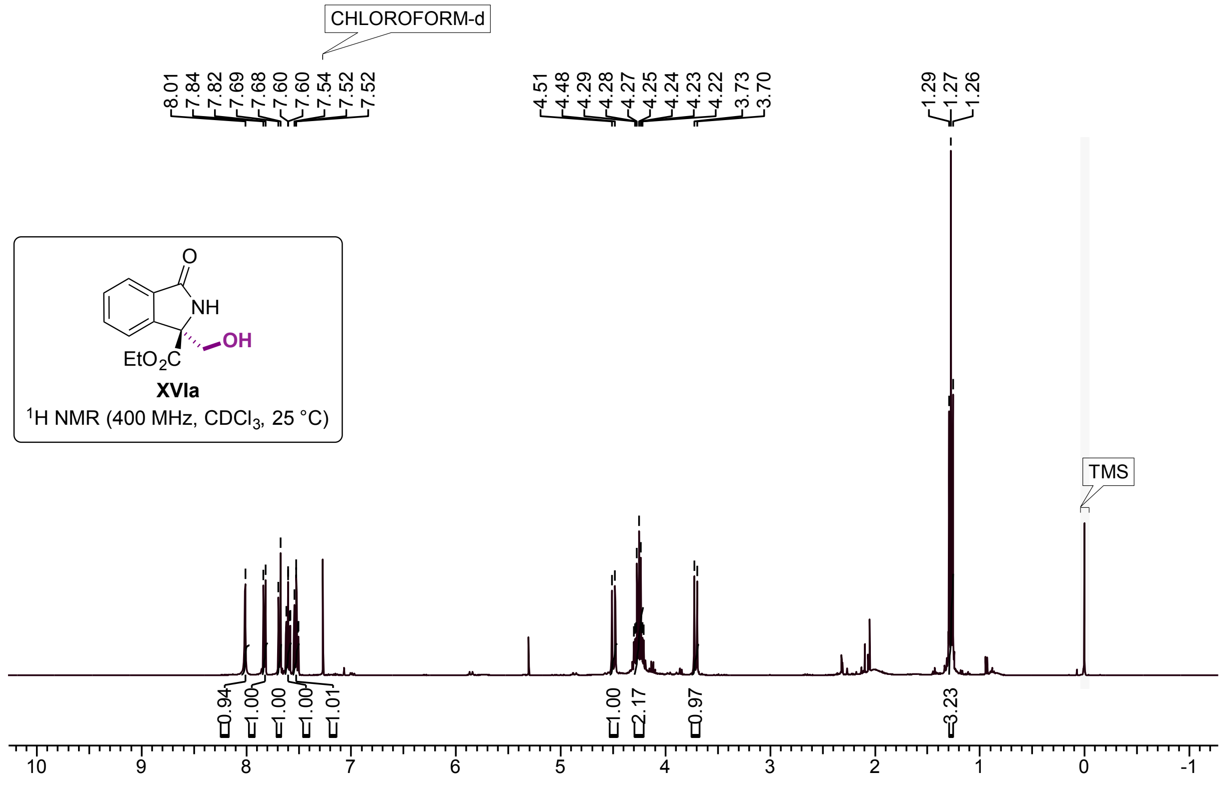
CHLOROFORM-d
8.01
7.84
7.82
7.69
7.68
7.60
7.60
7.54
7.52
7.52
4.51
4.48
4.29
4.28
4.27
4.25
4.24
4.23
4.22
3.73
3.70
1.29
1.27
1.26
XVIa
^{1}H NMR (400 MHz, CDCl$_3$, 25 °C)
TMS
0.94
1.00
1.00
1.00
1.01
1.00
2.17
0.97
3.23
10
9
8
7
6
5
4
3
2
1
0
-1

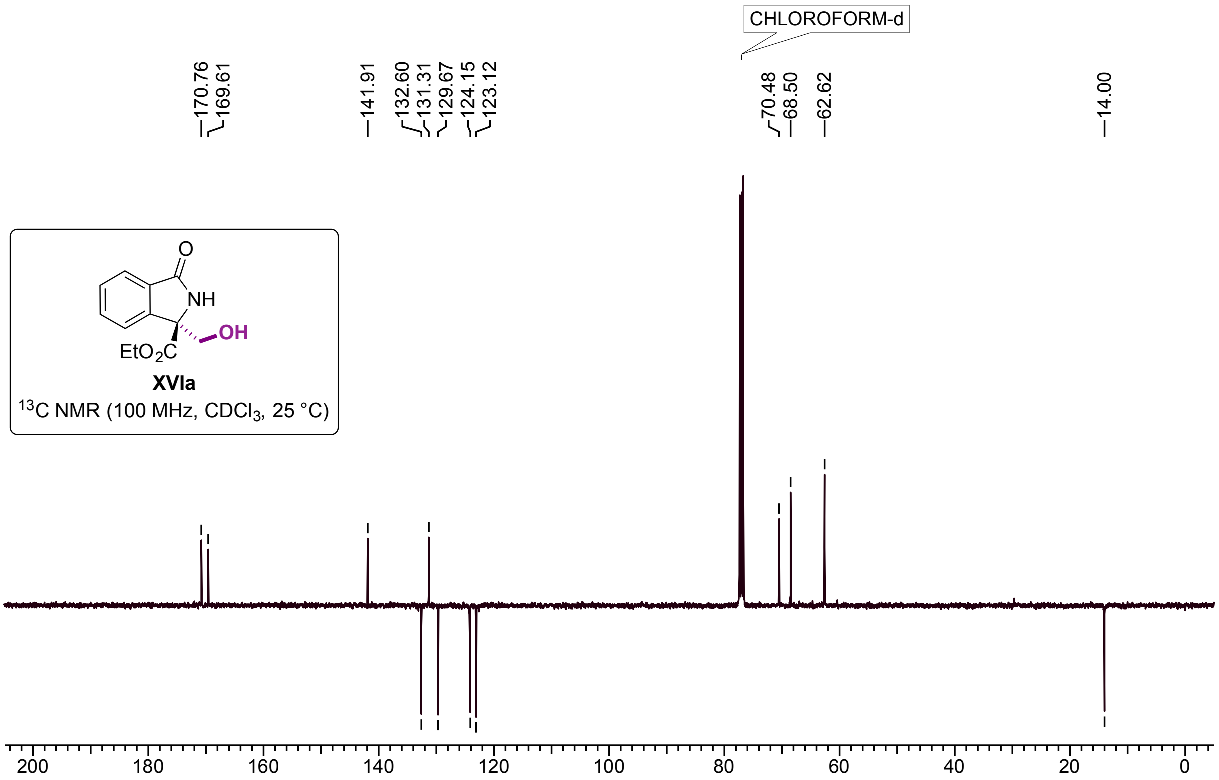
CHLOROFORM-d
170.76
169.61
141.91
132.60
131.31
129.67
124.15
123.12
70.48
68.50
62.62
14.00
O
NH
OH
EtO_2C
XVIa
^{13}C NMR (100 MHz, $CDCl_3$, 25 °C)
200
180
160
140
120
100
80
60
40
20
0

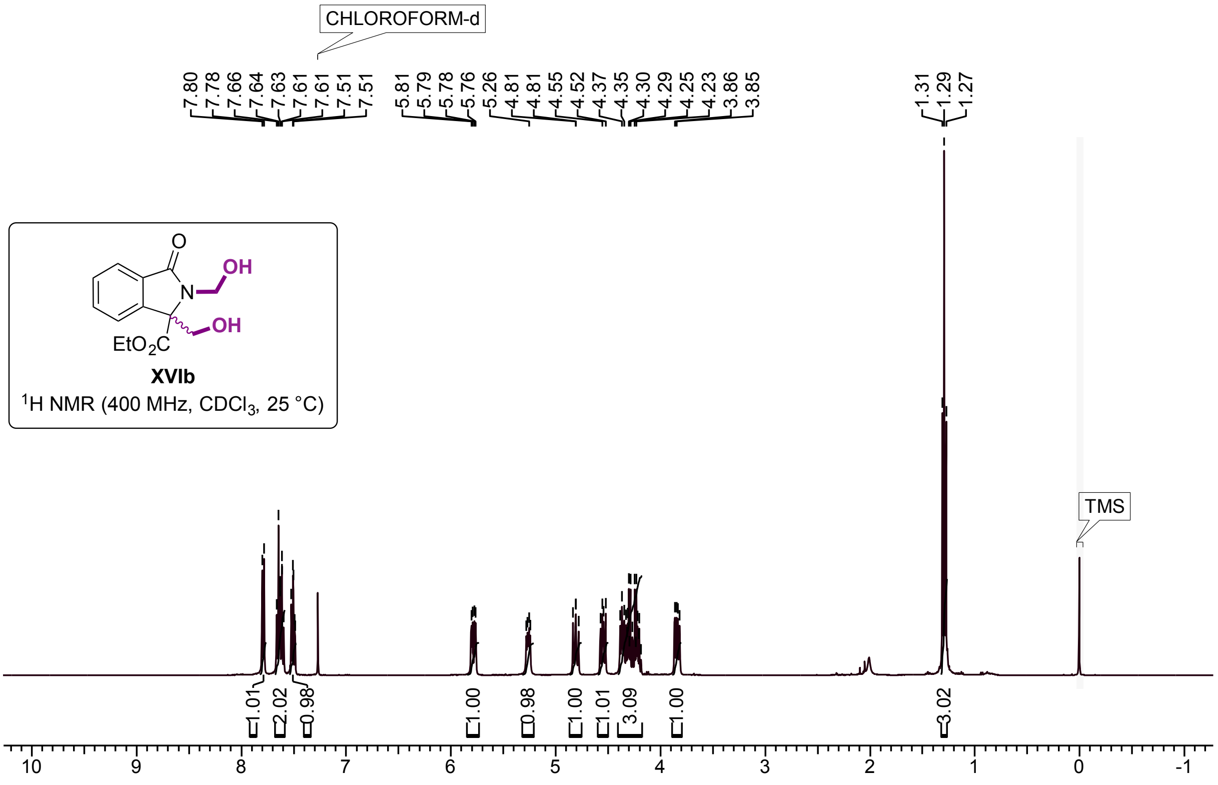
CHLOROFORM-d
7.80
7.78
7.66
7.64
7.63
7.61
7.51
7.51
5.81
5.79
5.78
5.76
5.26
4.81
4.81
4.55
4.52
4.37
4.35
4.30
4.29
4.25
4.23
3.86
3.85
1.31
1.29
1.27
O
N
OH
OH
EtO_2C
XVIb
^1H NMR (400 MHz, $CDCl_3$, 25 °C)
TMS
1.01
2.02
0.98
1.00
0.98
1.00
1.01
3.09
1.00
3.02
10
9
8
7
6
5
4
3
2
1
0
-1

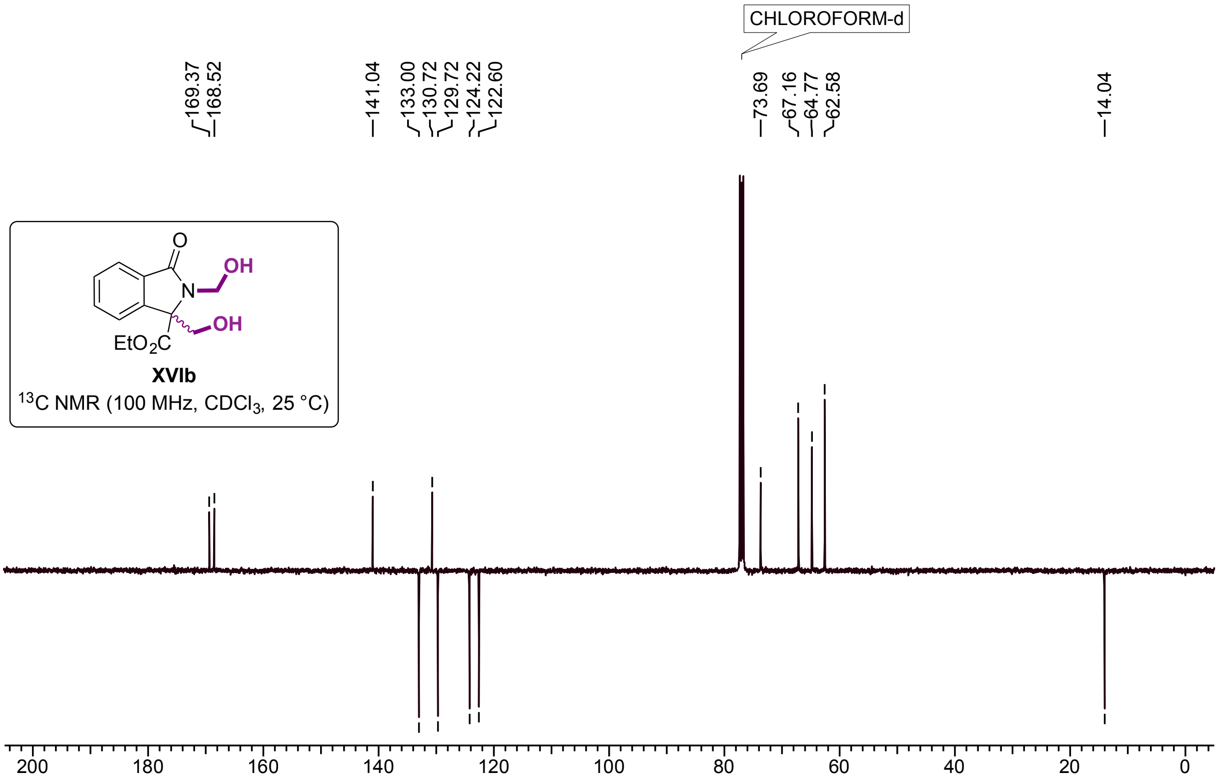
CHLOROFORM-d
169.37
168.52
141.04
133.00
130.72
129.72
124.22
122.60
73.69
67.16
64.77
62.58
14.04
OH
N
OH
O
EtO_2C
XVIb
^{13}C NMR (100 MHz, $CDCl_3$, 25 °C)
200
180
160
140
120
100
80
60
40
20
0

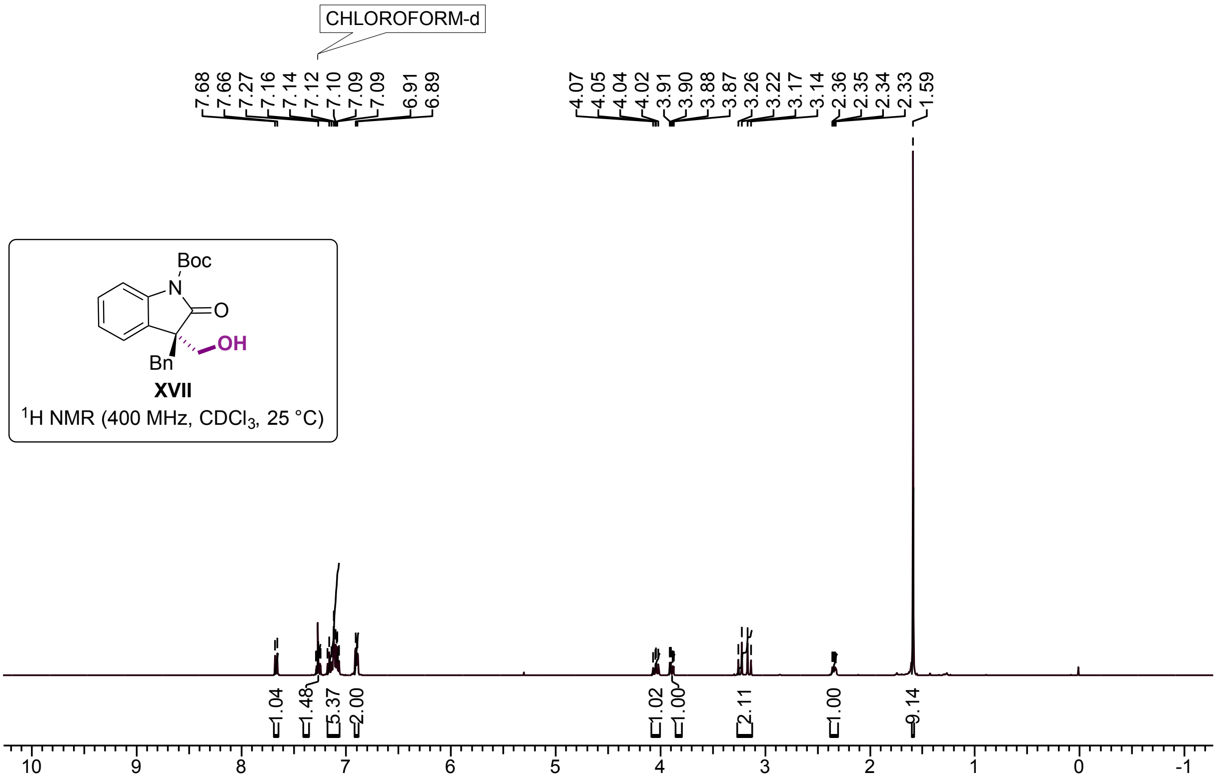
CHLOROFORM-d
7.68
7.66
7.27
7.16
7.14
7.12
7.10
7.09
7.09
6.91
6.89
4.07
4.05
4.04
4.02
3.91
3.90
3.88
3.87
3.26
3.22
3.17
3.14
2.36
2.35
2.34
2.33
1.59
Boc
N
O
OH
Bn
XVII
^{1}H NMR (400 MHz, $CDCl_3$, 25 °C)
1.04
1.48
5.37
2.00
1.02
1.00
2.11
1.00
9.14
10
9
8
7
6
5
4
3
2
1
0
-1

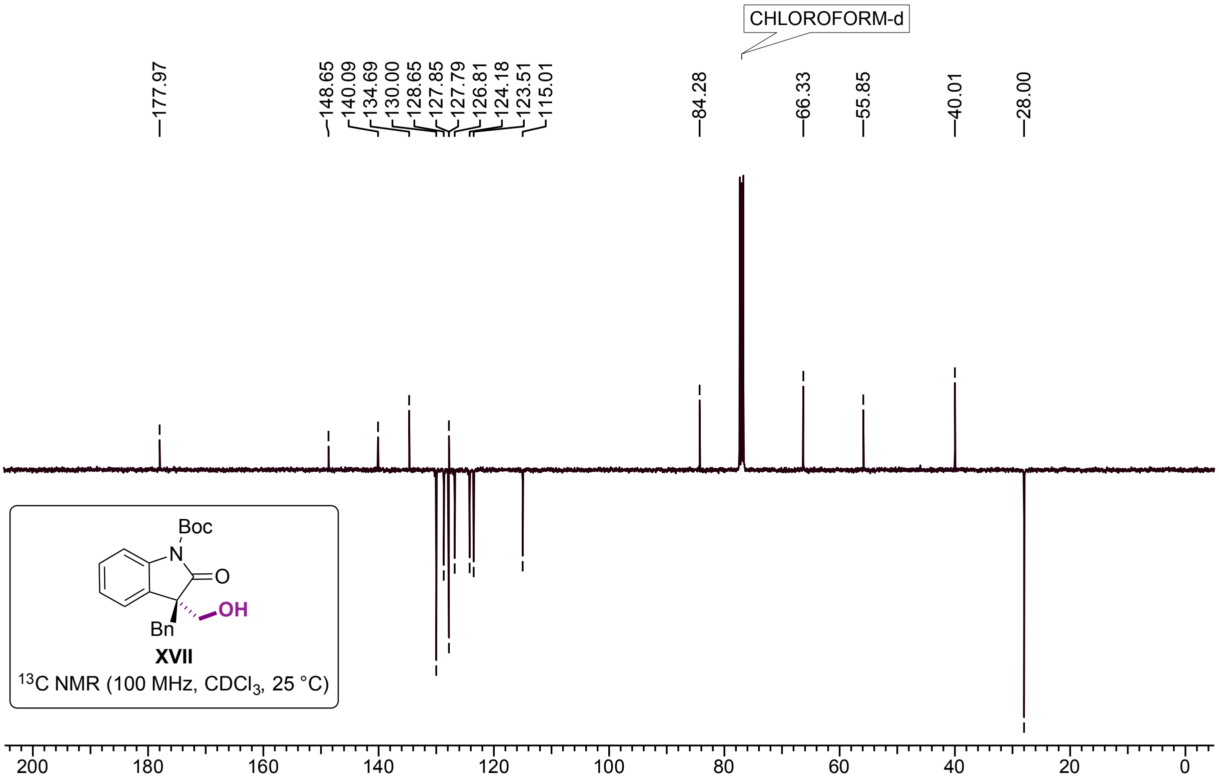
CHLOROFORM-d
177.97
148.65
140.09
134.69
130.00
128.65
127.85
127.79
126.81
124.18
123.51
115.01
84.28
66.33
55.85
40.01
28.00
Boc
N
O
OH
Bn
XVII
^{13}C NMR (100 MHz, CDCl$_3$, 25 °C)
200 180 160 140 120 100 80 60 40 20 0

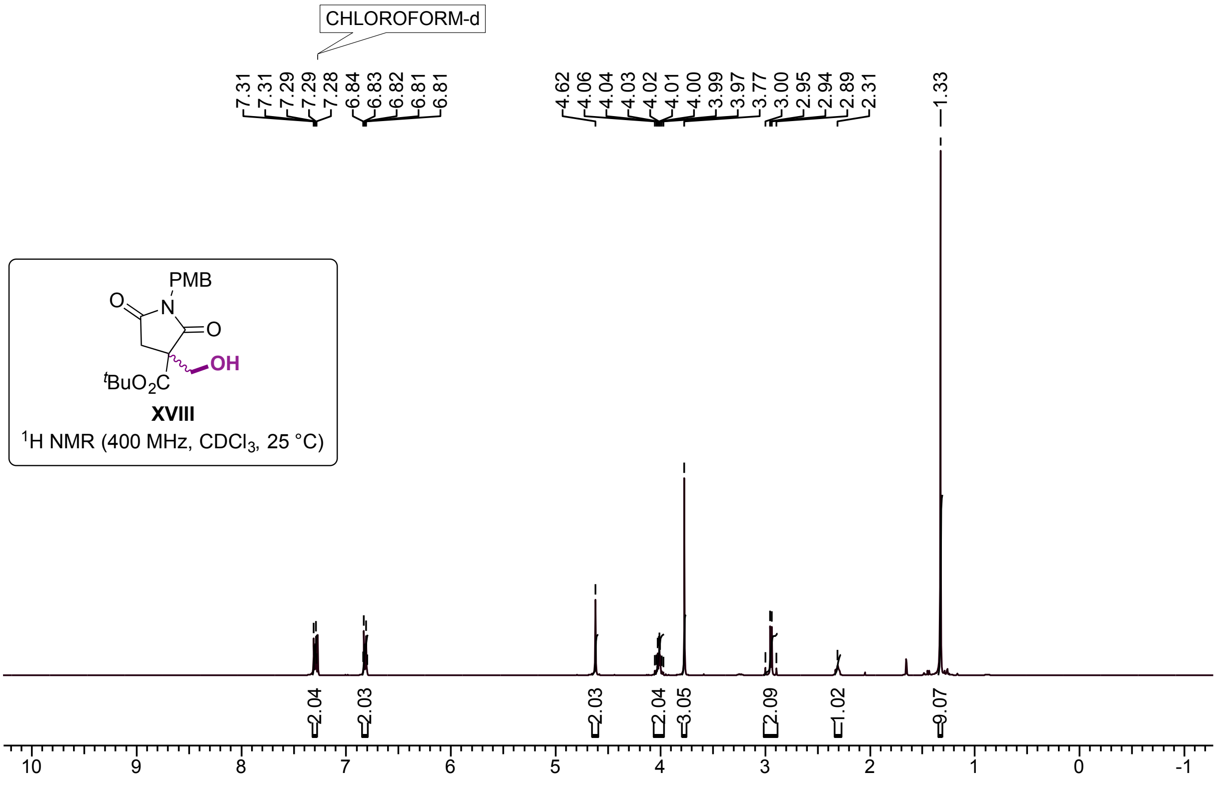
CHLOROFORM-d
7.31
7.31
7.29
7.29
7.28
6.84
6.83
6.82
6.81
6.81
4.62
4.06
4.04
4.03
4.02
4.01
4.00
3.99
3.97
3.77
3.00
2.95
2.94
2.89
2.31
1.33
PMB
O
N
O
OH
^{t}BuO$_2$C
XVIII
^{1}H NMR (400 MHz, CDCl$_3$, 25 °C)
2.04
2.03
2.03
2.04
3.05
2.09
1.02
9.07
10 9 8 7 6 5 4 3 2 1 0 -1

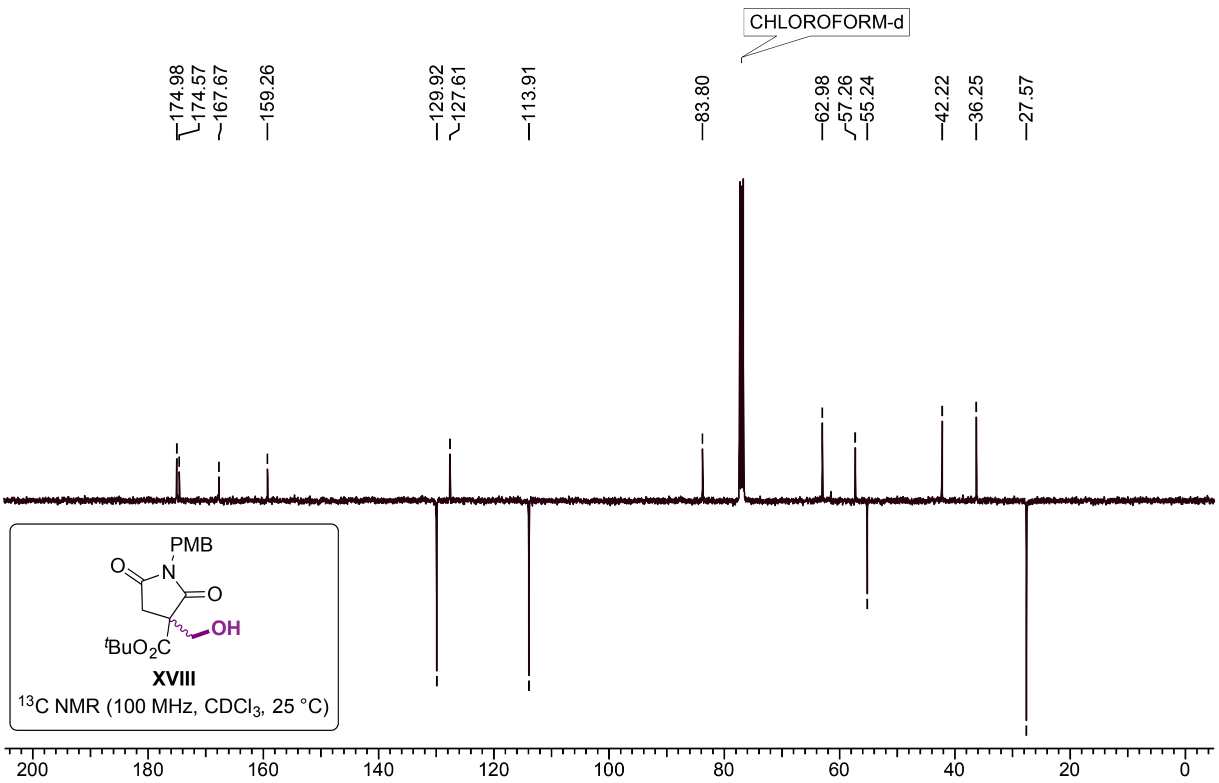
CHLOROFORM-d
174.98
174.57
167.67
159.26
129.92
127.61
113.91
83.80
62.98
57.26
55.24
42.22
36.25
27.57
PMB
N
O
O
OH
$^tBuO_2C$
XVIII
^{13}C NMR (100 MHz, $CDCl_3$, 25 °C)
200
180
160
140
120
100
80
60
40
20
0

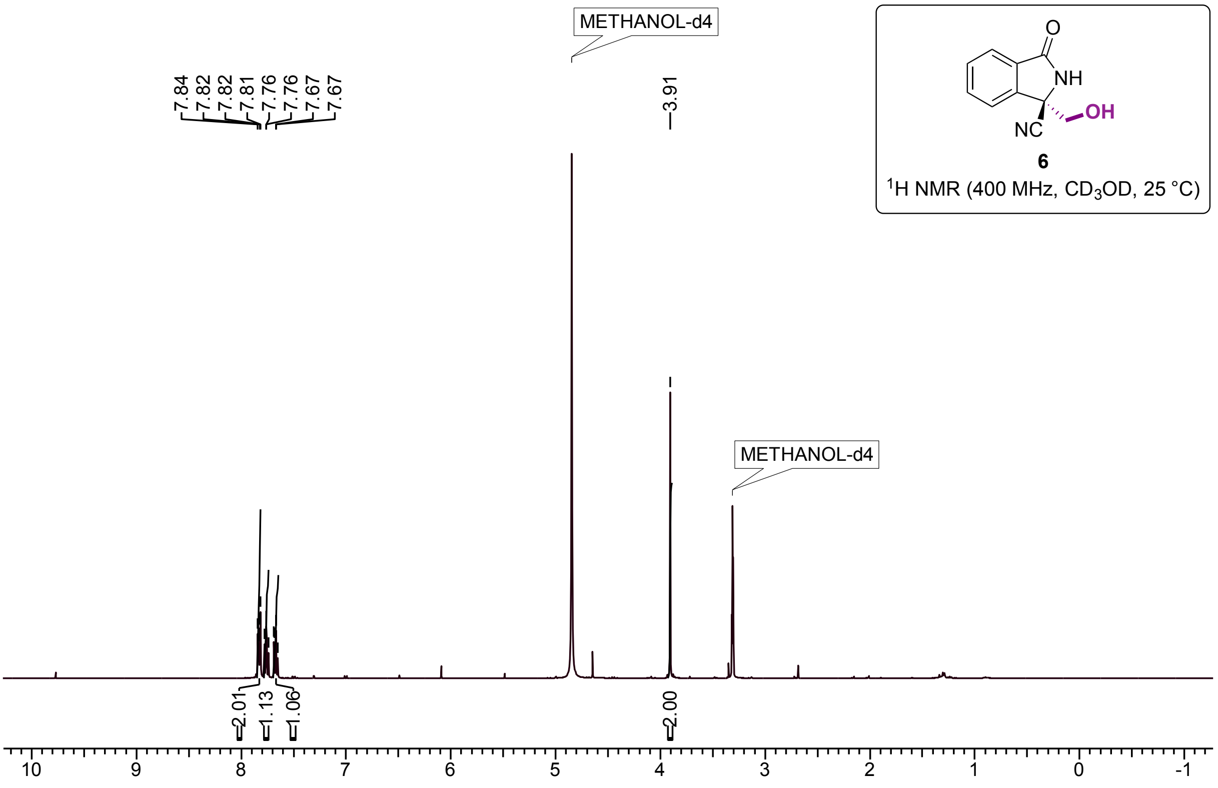
METHANOL-d4
7.84
7.82
7.82
7.81
7.76
7.76
7.67
7.67
3.91
O
NH
NC
OH
6
^1H NMR (400 MHz, CD_3OD, 25 °C)
METHANOL-d4
2.01
1.13
1.06
2.00
10
9
8
7
6
5
4
3
2
1
0
-1

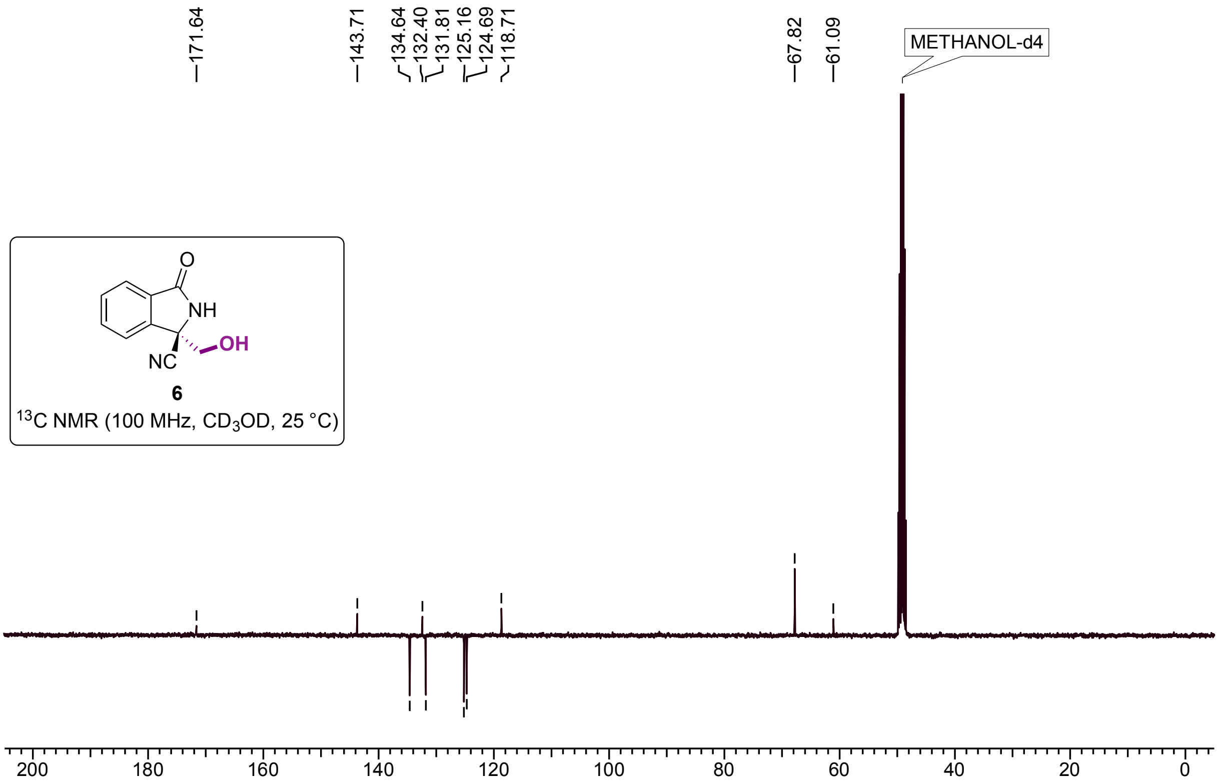
171.64
143.71
134.64
132.40
131.81
125.16
124.69
118.71
67.82
61.09
METHANOL-d4
O
NH
NC
OH
6
13C NMR (100 MHz, CD3OD, 25 °C)
200 180 160 140 120 100 80 60 40 20 0

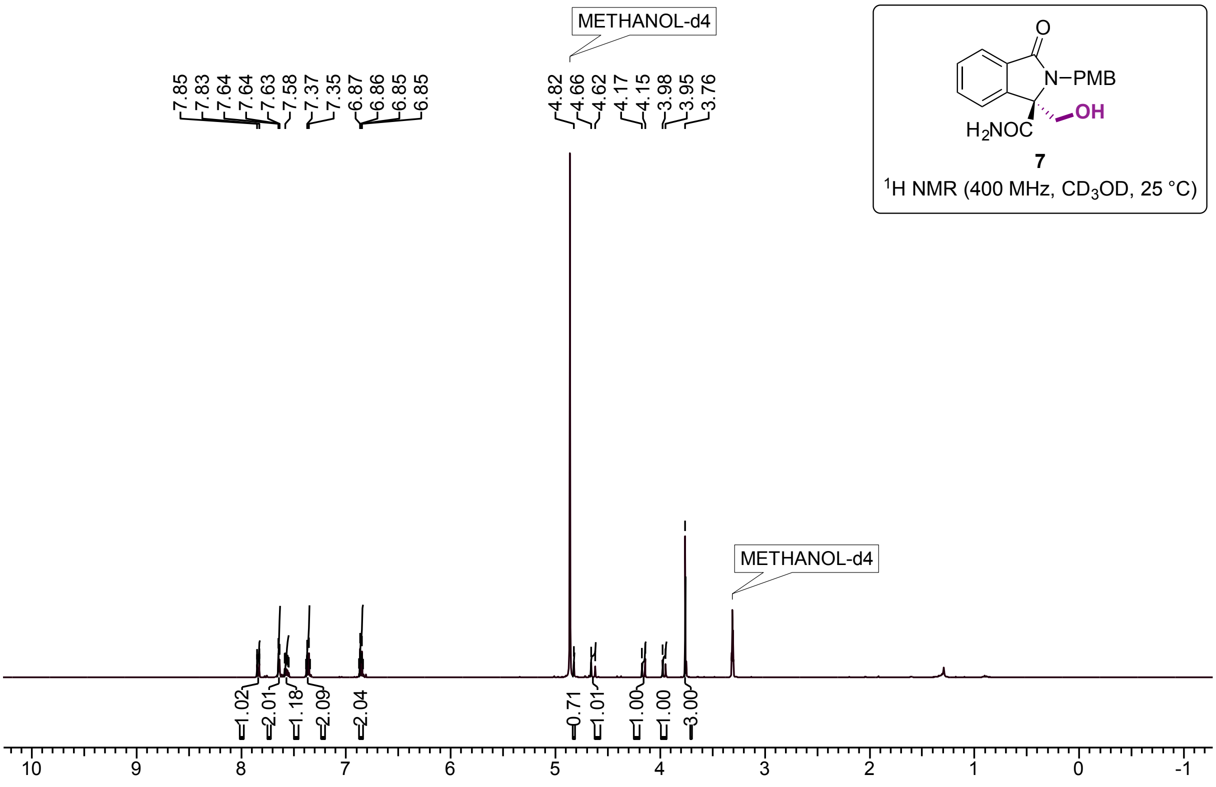
7.85
7.83
7.64
7.64
7.63
7.58
7.37
7.35
6.87
6.86
6.85
6.85
METHANOL-d4
4.82
4.66
4.62
4.17
4.15
3.98
3.95
3.76
O
N−PMB
H2NOC
OH
7
1H NMR (400 MHz, CD3OD, 25 °C)
METHANOL-d4
1.02
2.01
1.18
2.09
2.04
0.71
1.01
1.00
1.00
3.00
10 9 8 7 6 5 4 3 2 1 0 -1

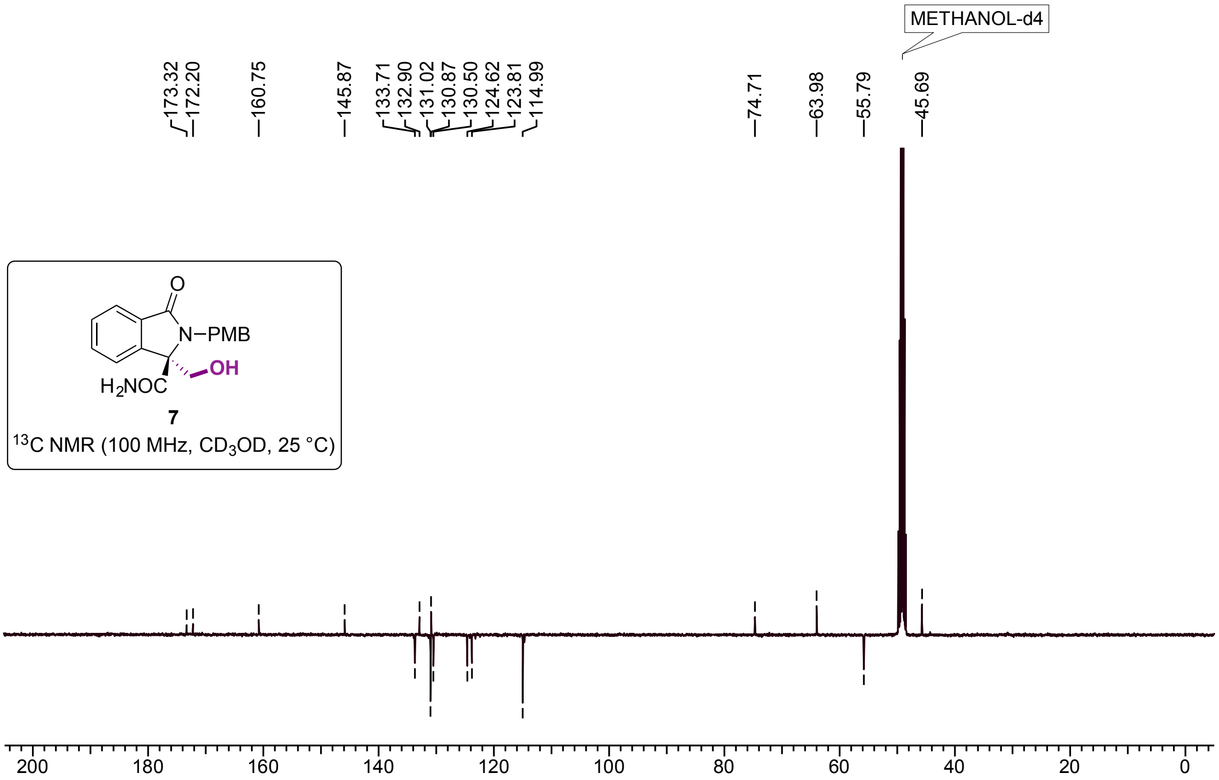
METHANOL-d4
173.32
172.20
160.75
145.87
133.71
132.90
131.02
130.87
130.50
124.62
123.81
114.99
74.71
63.98
55.79
45.69
O
N−PMB
OH
H2NOC
7
13C NMR (100 MHz, CD3OD, 25 °C)
200
180
160
140
120
100
80
60
40
20
0

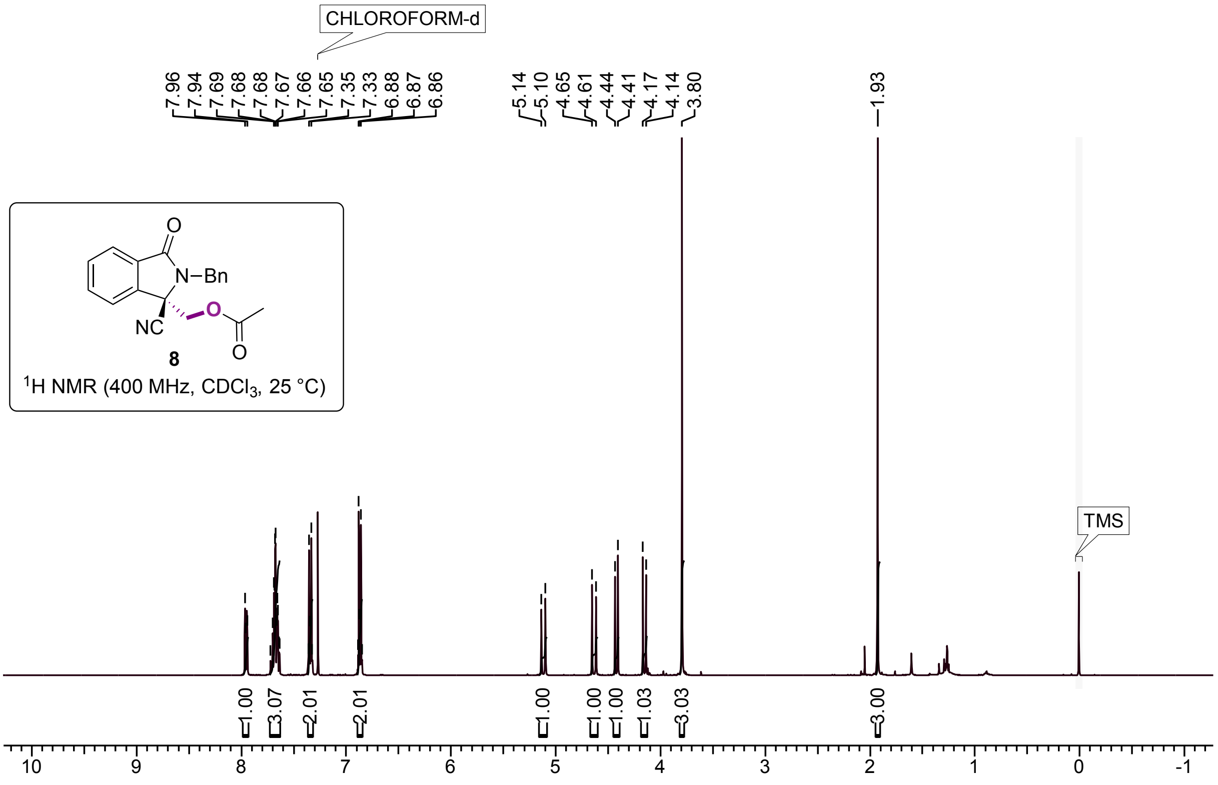
CHLOROFORM-d
7.96
7.94
7.69
7.68
7.68
7.67
7.66
7.65
7.35
7.33
6.88
6.87
6.86
5.14
5.10
4.65
4.61
4.44
4.41
4.17
4.14
3.80
1.93
O
N−Bn
NC
O
O
8
1H NMR (400 MHz, CDCl3, 25 °C)
TMS
1.00
3.07
2.01
2.01
1.00
1.00
1.00
1.03
3.03
3.00
10
9
8
7
6
5
4
3
2
1
0
-1

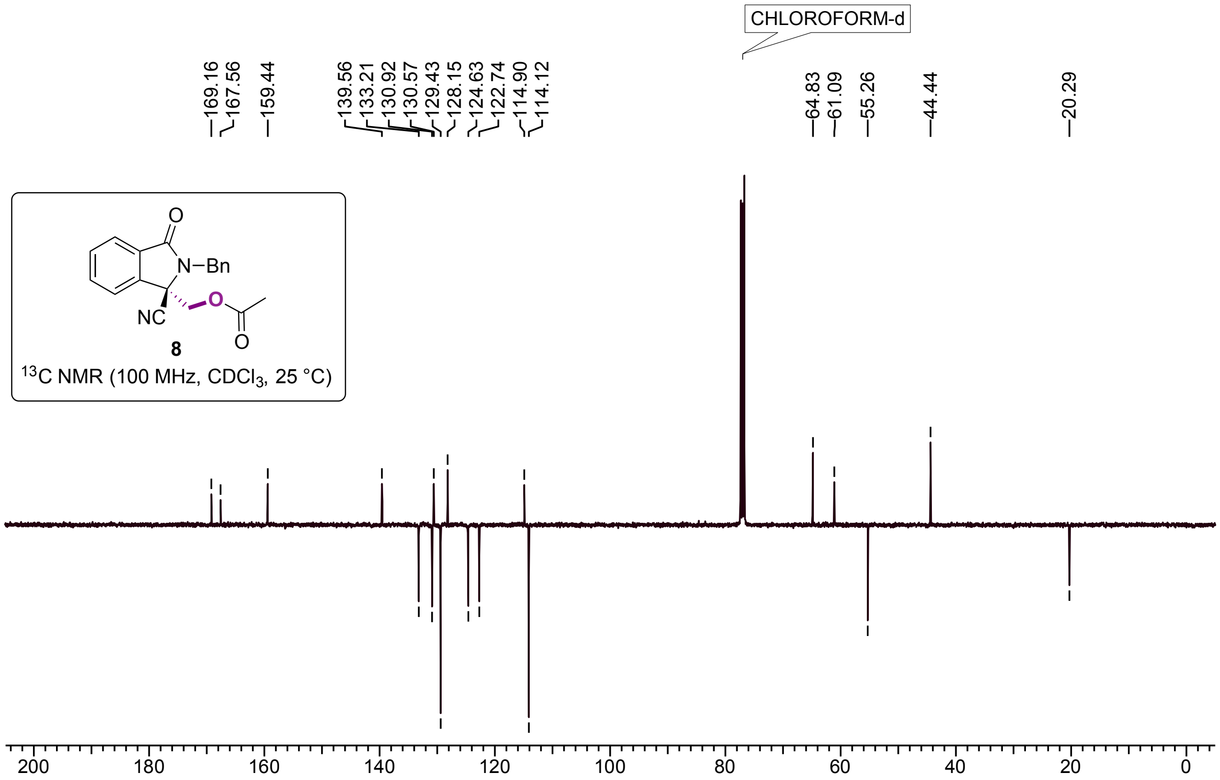
CHLOROFORM-d
169.16
167.56
159.44
139.56
133.21
130.92
130.57
129.43
128.15
124.63
122.74
114.90
114.12
64.83
61.09
55.26
44.44
20.29
N−Bn
O
NC
8
^{13}C NMR (100 MHz, CDCl$_3$, 25 °C)
200
180
160
140
120
100
80
60
40
20
0

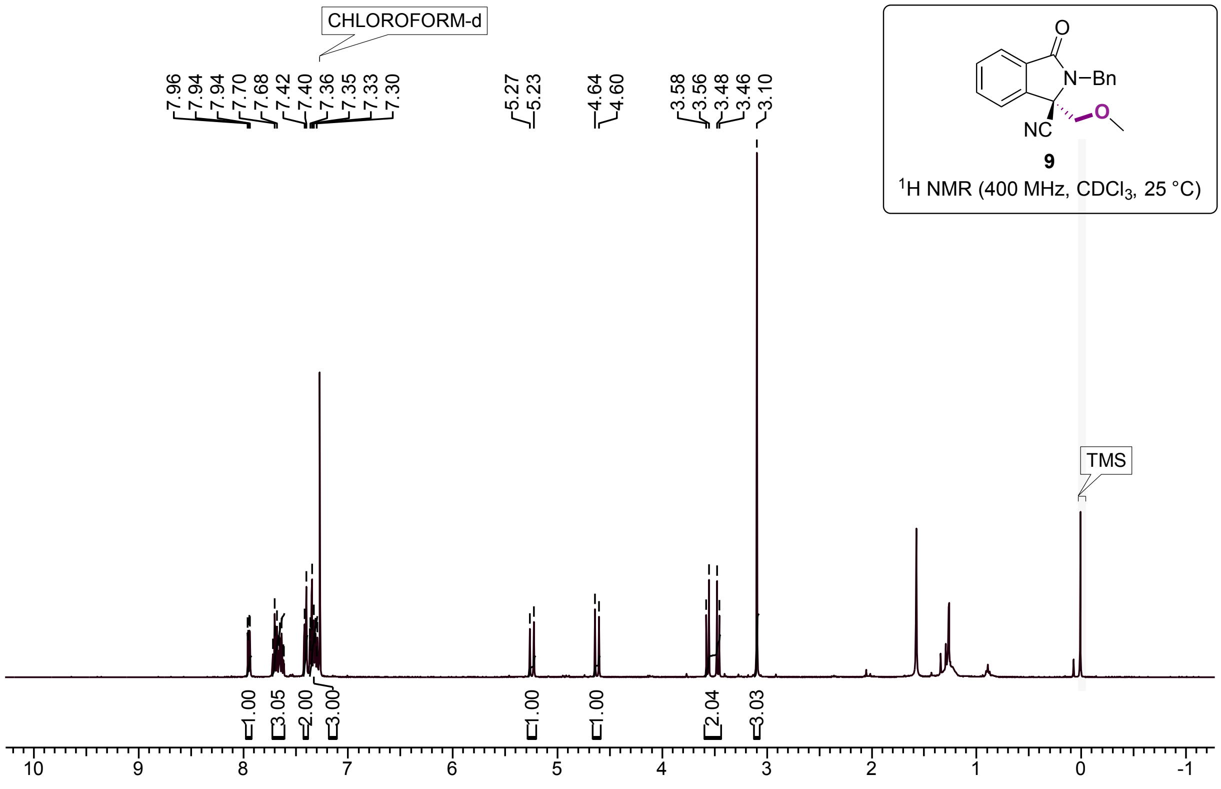
CHLOROFORM-d
7.96
7.94
7.94
7.70
7.68
7.42
7.40
7.36
7.35
7.33
7.30
5.27
5.23
4.64
4.60
3.58
3.56
3.48
3.46
3.10
O
N−Bn
NC
9
^{1}H NMR (400 MHz, CDCl$_3$, 25 °C)
TMS
1.00
3.05
2.00
3.00
1.00
1.00
2.04
3.03
10
9
8
7
6
5
4
3
2
1
0
-1

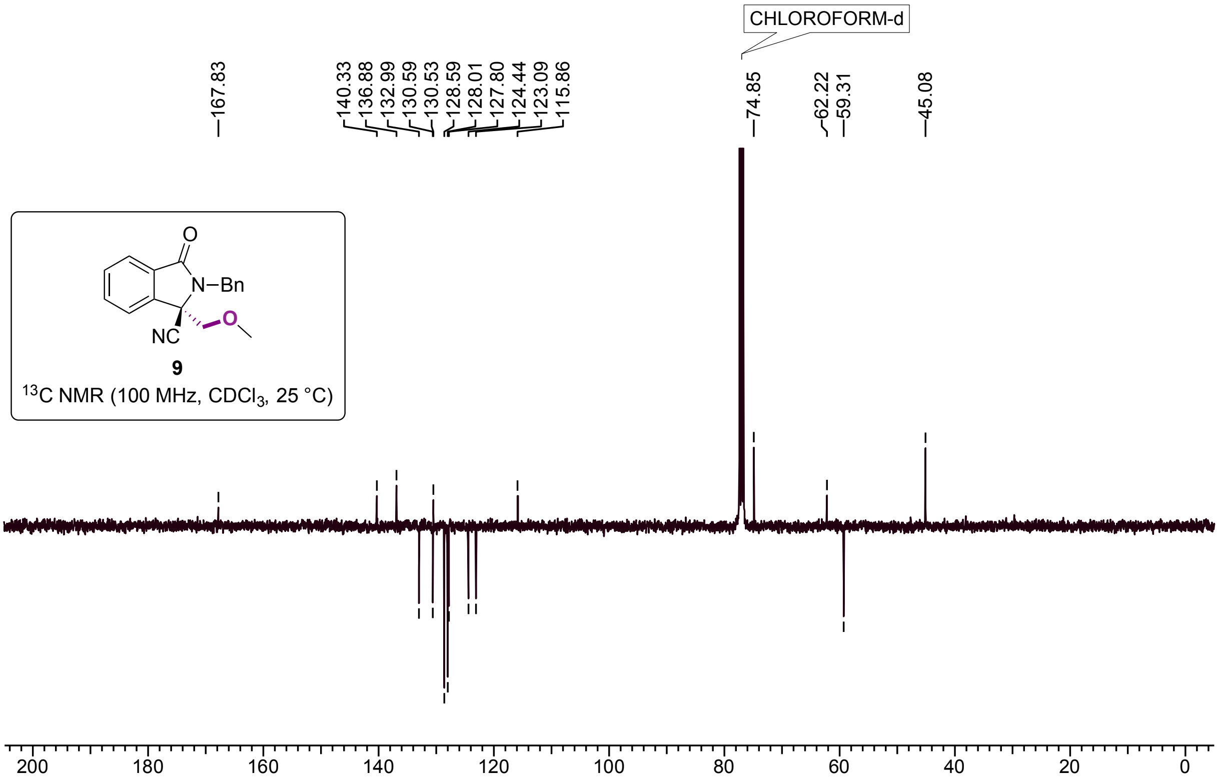
CHLOROFORM-d
167.83
140.33
136.88
132.99
130.59
130.53
128.59
128.01
127.80
124.44
123.09
115.86
74.85
62.22
59.31
45.08
O
N−Bn
NC
O
9
^{13}C NMR (100 MHz, $CDCl_3$, 25 °C)
200
180
160
140
120
100
80
60
40
20
0

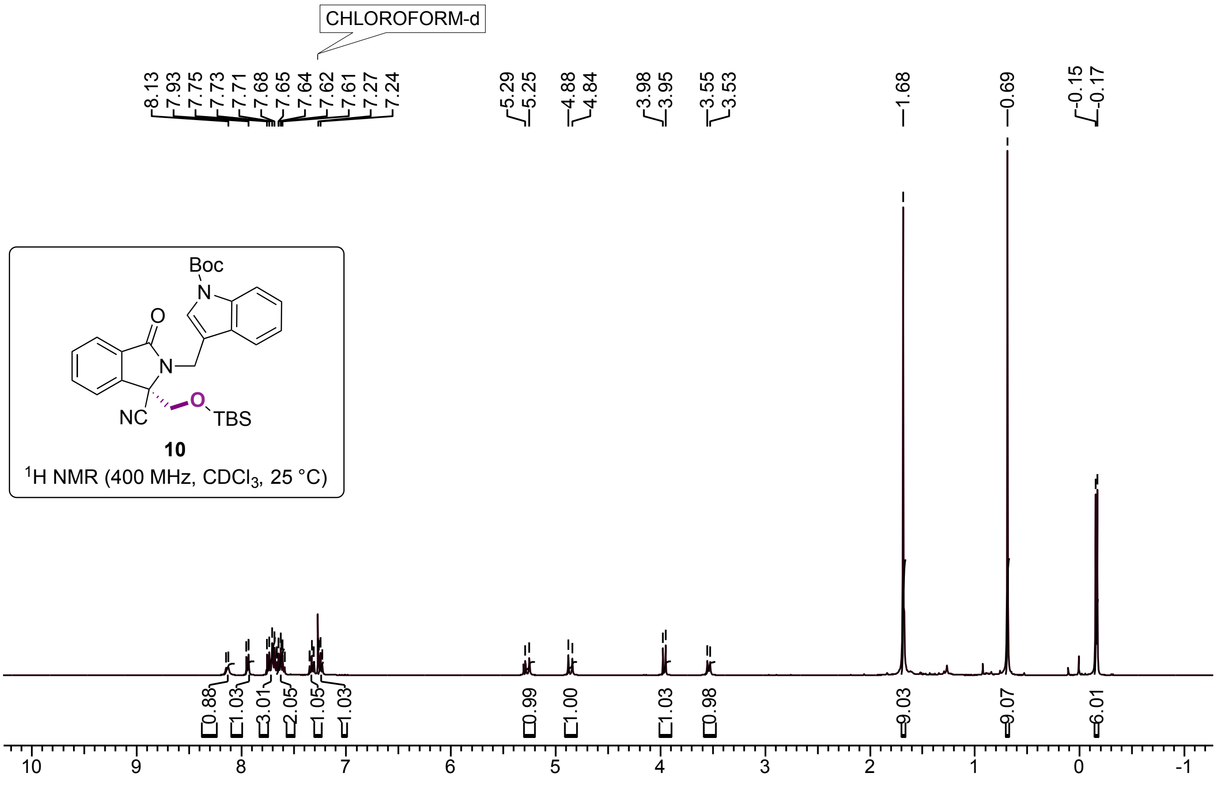
CHLOROFORM-d
8.13
7.93
7.75
7.73
7.71
7.68
7.65
7.64
7.62
7.61
7.27
7.24
5.29
5.25
4.88
4.84
3.98
3.95
3.55
3.53
1.68
0.69
-0.15
-0.17
Boc
N
O
N
NC
O
TBS
10
^{1}H NMR (400 MHz, $CDCl_3$, 25 °C)
0.88
1.03
3.01
2.05
1.05
1.03
0.99
1.00
1.03
0.98
9.03
9.07
6.01
10
9
8
7
6
5
4
3
2
1
0
-1

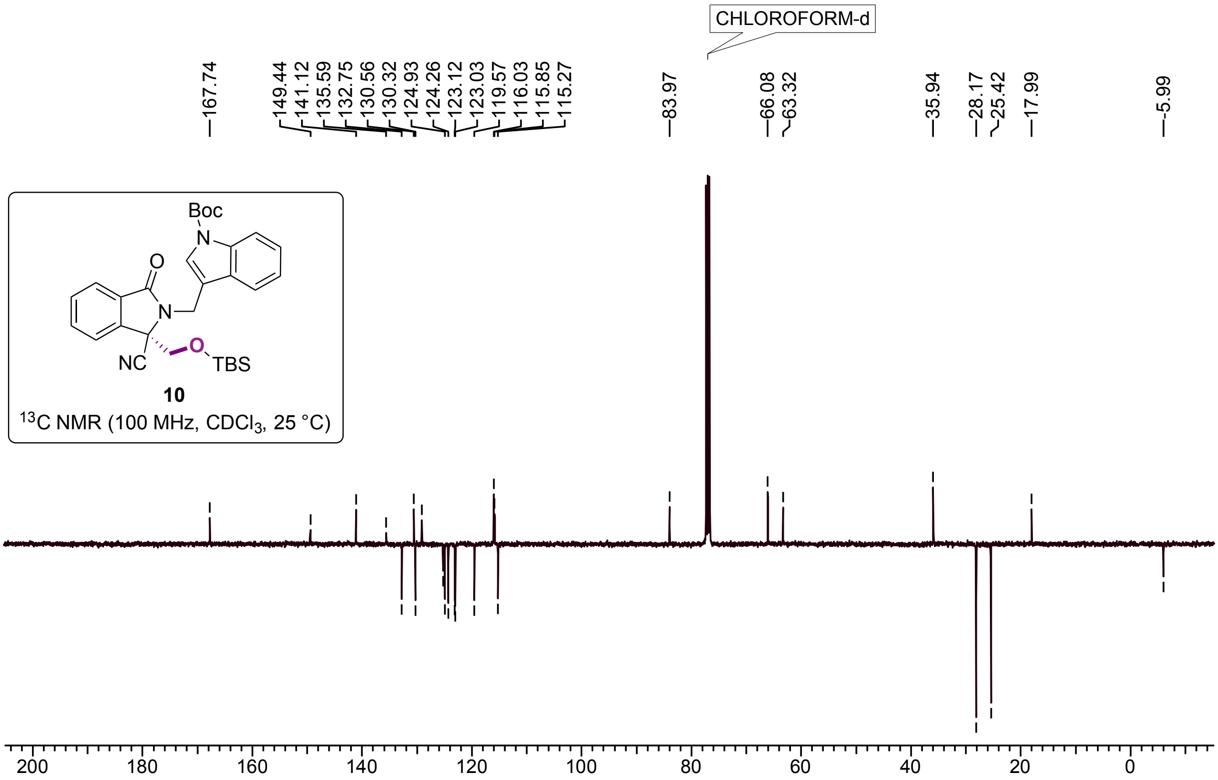

CHLOROFORM-d
167.74
149.44
141.12
135.59
132.75
130.56
130.32
124.93
124.26
123.12
123.03
119.57
116.03
115.85
115.27
83.97
66.08
63.32
35.94
28.17
25.42
17.99
-5.99
Boc
N
O
N
NC
O
TBS
10
13C NMR (100 MHz, CDCl3, 25 °C)
200
180
160
140
120
100
80
60
40
20
0

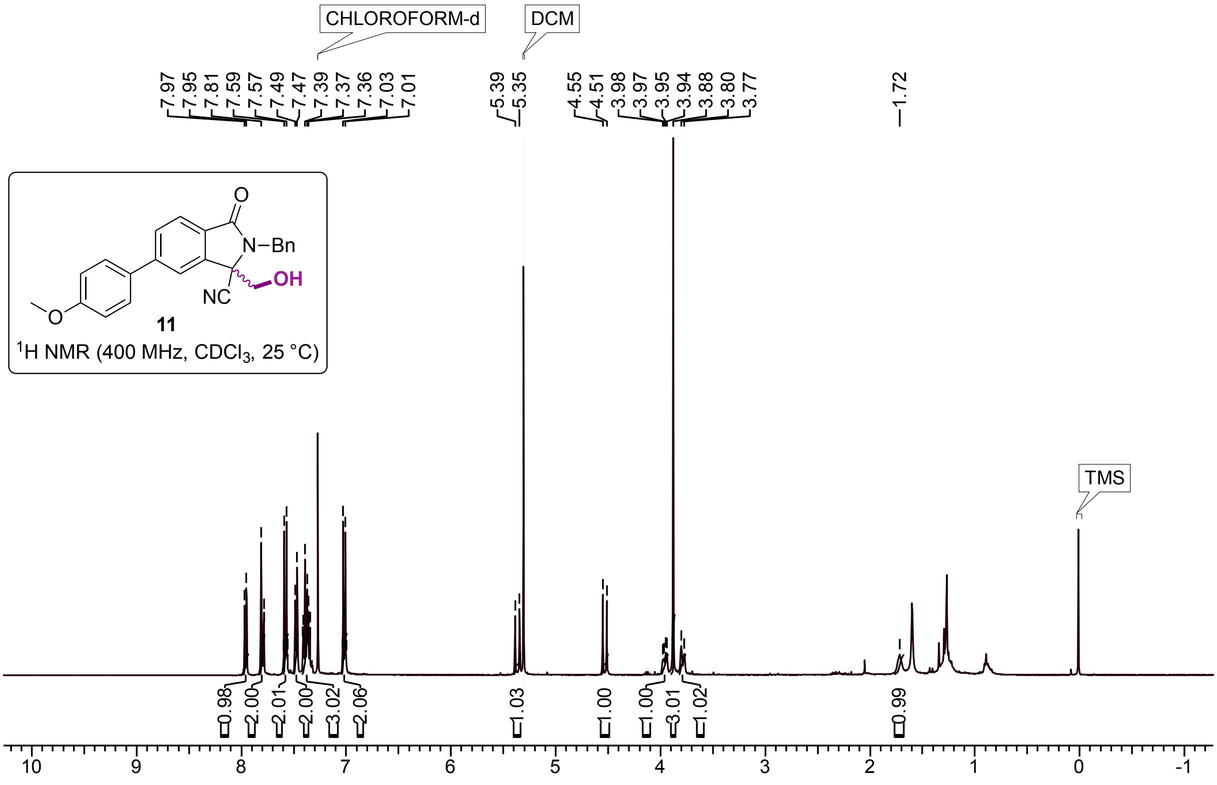

CHLOROFORM-d
DCM
7.97
7.95
7.81
7.59
7.57
7.49
7.47
7.39
7.37
7.36
7.03
7.01
5.39
5.35
4.55
4.51
3.98
3.97
3.95
3.94
3.88
3.80
3.77
1.72
O
N–Bn
OH
NC
O
11
1H NMR (400 MHz, CDCl3, 25 °C)
TMS
0.98
2.00
2.01
2.00
3.02
2.06
1.03
1.00
1.00
3.01
1.02
0.99
10
9
8
7
6
5
4
3
2
1
0
-1

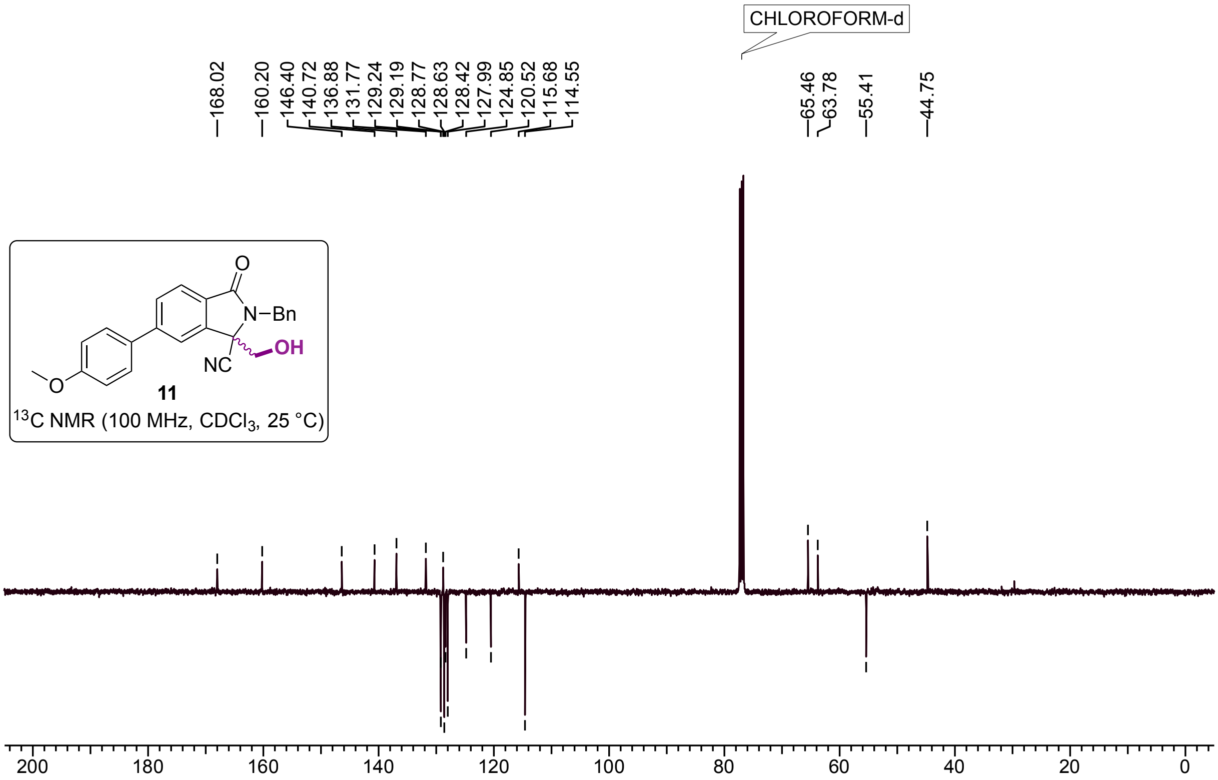

CHLOROFORM-d
168.02
160.20
146.40
140.72
136.88
131.77
129.24
129.19
128.77
128.63
128.42
127.99
124.85
120.52
115.68
114.55
65.46
63.78
55.41
44.75
O
N−Bn
OH
NC
O
11
13C NMR (100 MHz, CDCl3, 25 °C)
200
180
160
140
120
100
80
60
40
20
0

9. Copies of HPLC Chromatograms

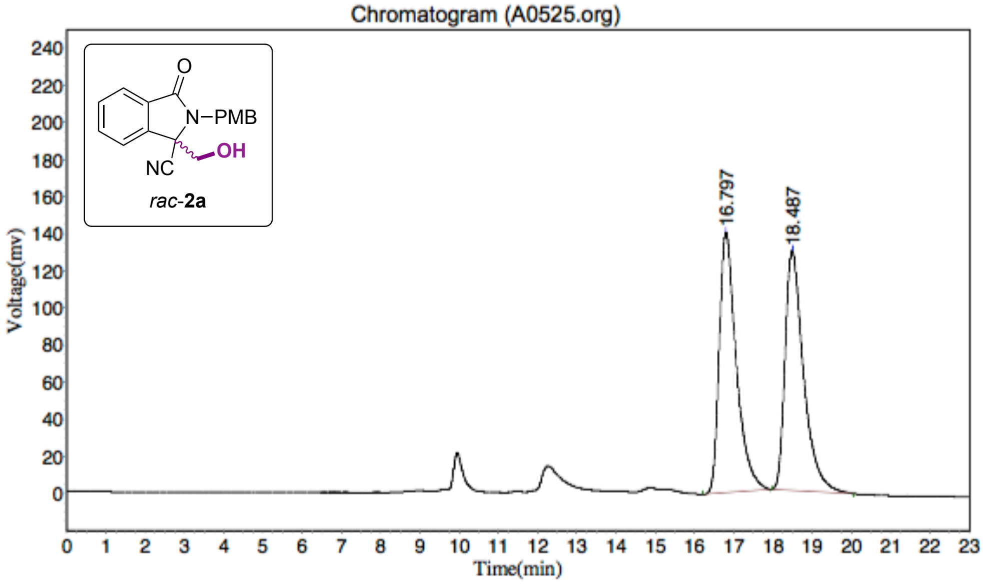

Results

Peak No.	Peak ID	Ret Time	Height	Area	Conc.
1		16.797	140190.047	4217351.500	50.0254
2		18.487	129228.219	4213062.500	49.9746
**Total**			269418.266	8430414.000	100.0000

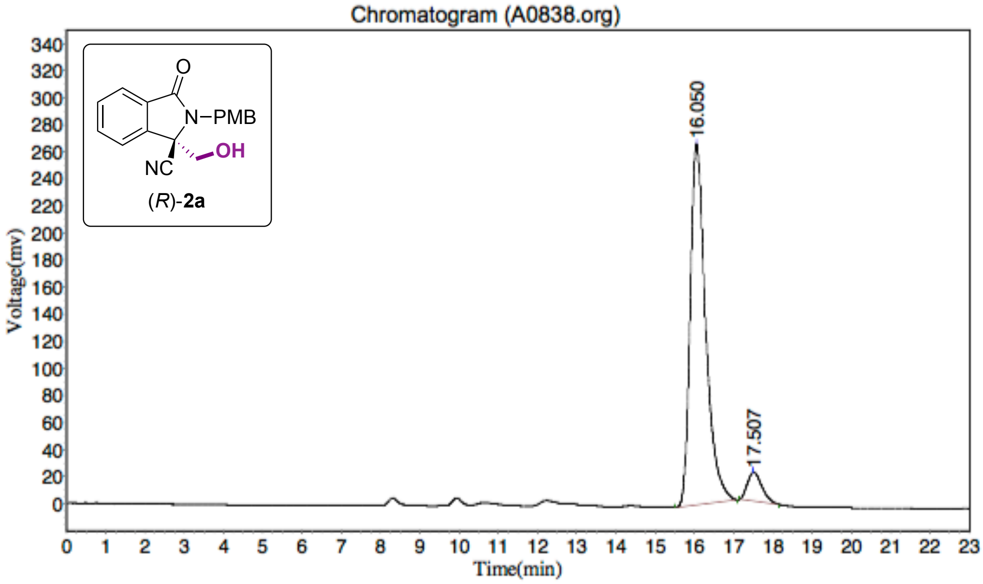

Results

Peak No.	Peak ID	Ret Time	Height	Area	Conc.
1		16.050	266805.969	7288845.000	92.9850
2		17.507	21129.840	549884.375	7.0150
**Total**			287935.809	7838729.375	100.0000

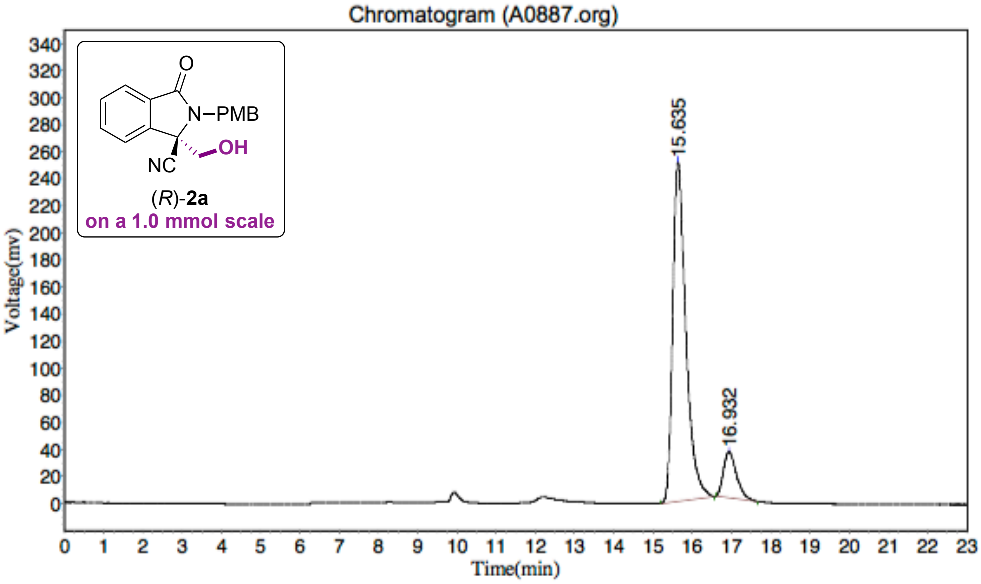

Results

Peak No.	Peak ID	Ret Time	Height	Area	Conc.
1		15.635	250713.188	5807995.500	88.0913
2		16.932	33893.848	785156.250	11.9087
**Total**			284607.035	6593151.750	100.0000

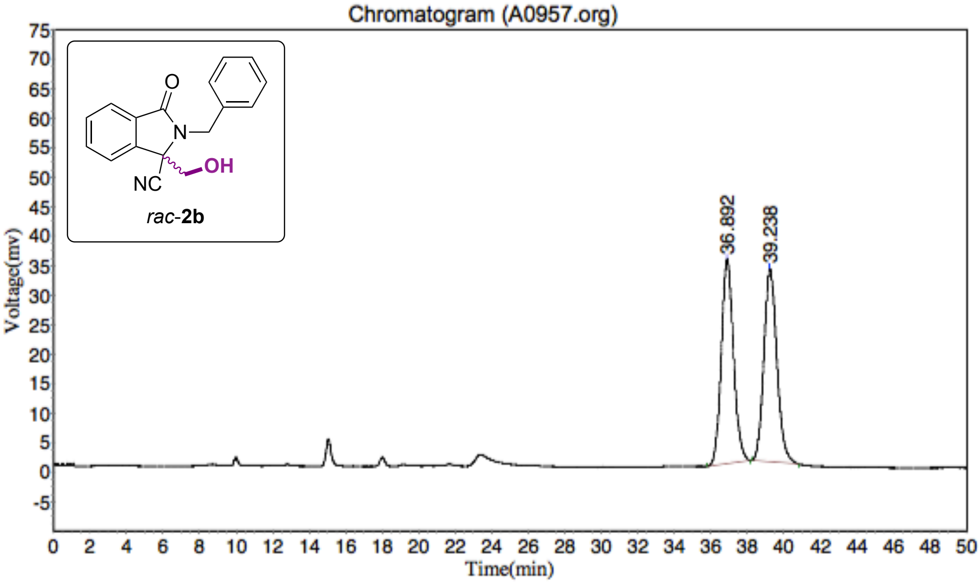

Results

Peak No.	Peak ID	Ret Time	Height	Area	Conc.
1		36.892	34676.227	1610188.500	49.8999
2		39.238	32733.707	1616651.625	50.1001
**Total**			67409.934	3226840.125	100.0000

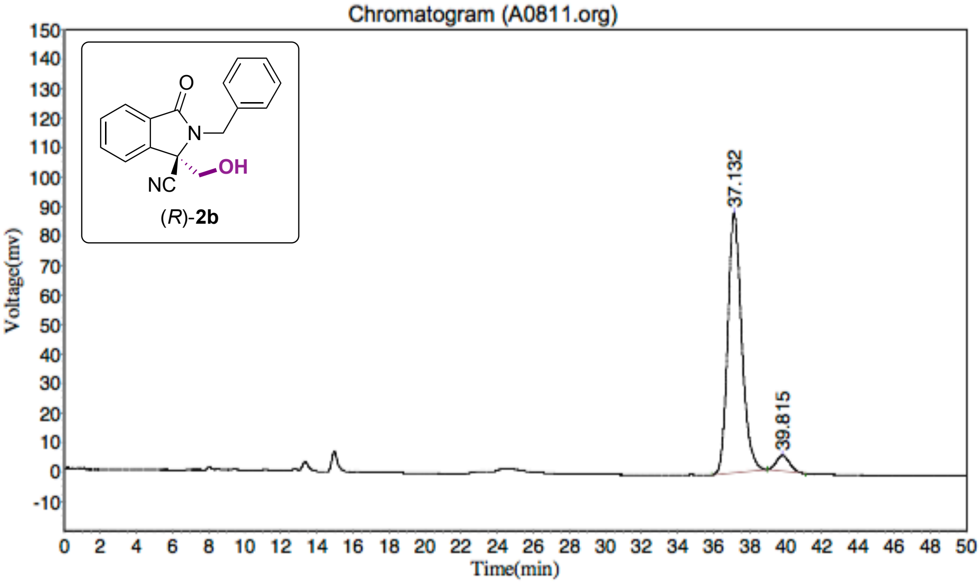

Results

Peak No.	Peak ID	Ret Time	Height	Area	Conc.
1		37.132	88230.031	4714544.000	94.5340
2		39.815	5293.982	272596.063	5.4660
**Total**			93524.014	4987140.063	100.0000

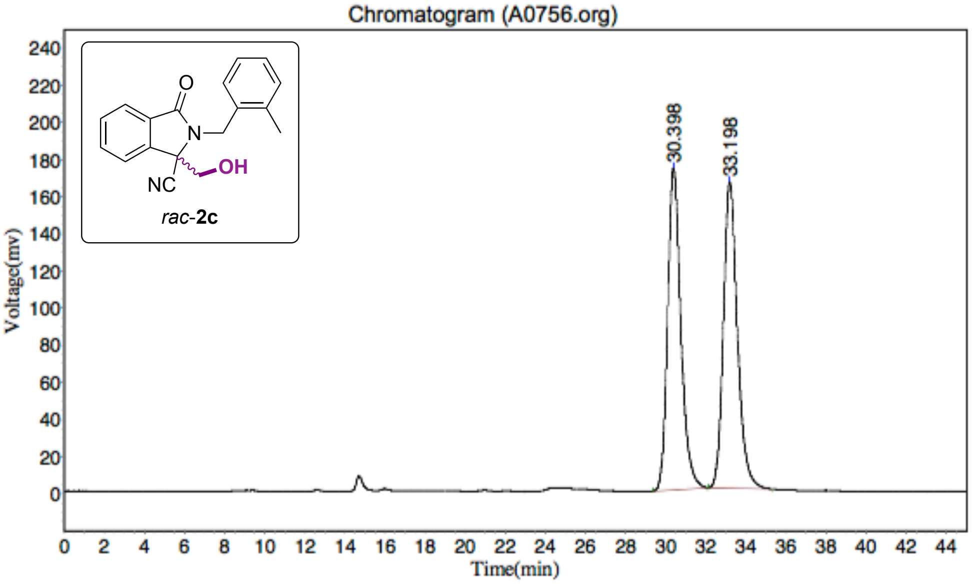

Results

Peak No.	Peak ID	Ret Time	Height	Area	Conc.
1		30.398	173665.484	7946921.500	49.8459
2		33.198	165094.109	7996063.500	50.1541
**Total**			338759.594	15942985.000	100.0000

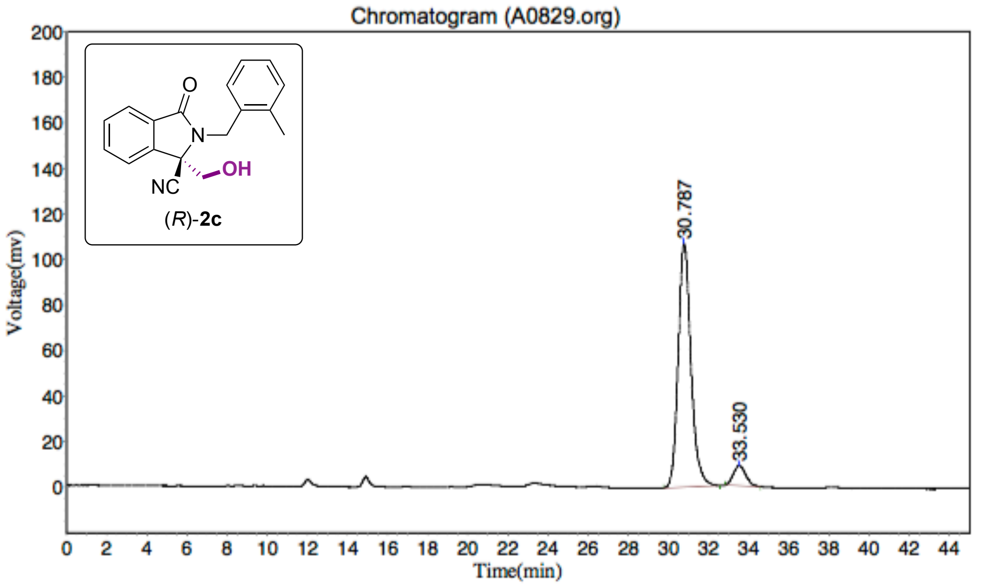

Results

Peak No.	Peak ID	Ret Time	Height	Area	Conc.
1		30.787	106839.531	4701437.500	92.4654
2		33.530	8852.943	383100.406	7.5346
**Total**			115692.475	5084537.906	100.0000

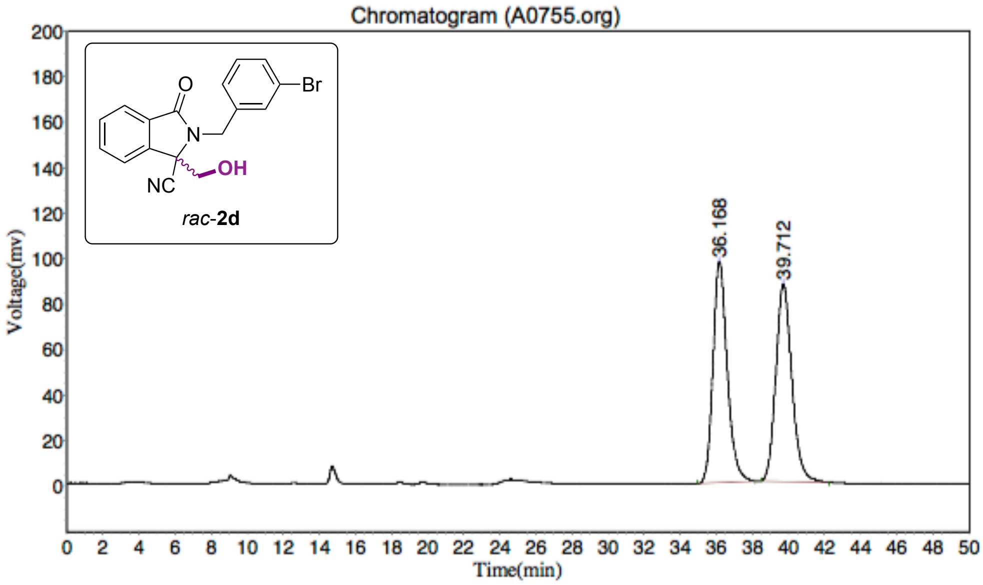

Results

Peak No.	Peak ID	Ret Time	Height	Area	Conc.
1		36.168	96912.453	5295746.000	49.8557
2		39.712	86634.281	5326399.000	50.1443
**Total**			183546.734	10622145.000	100.0000

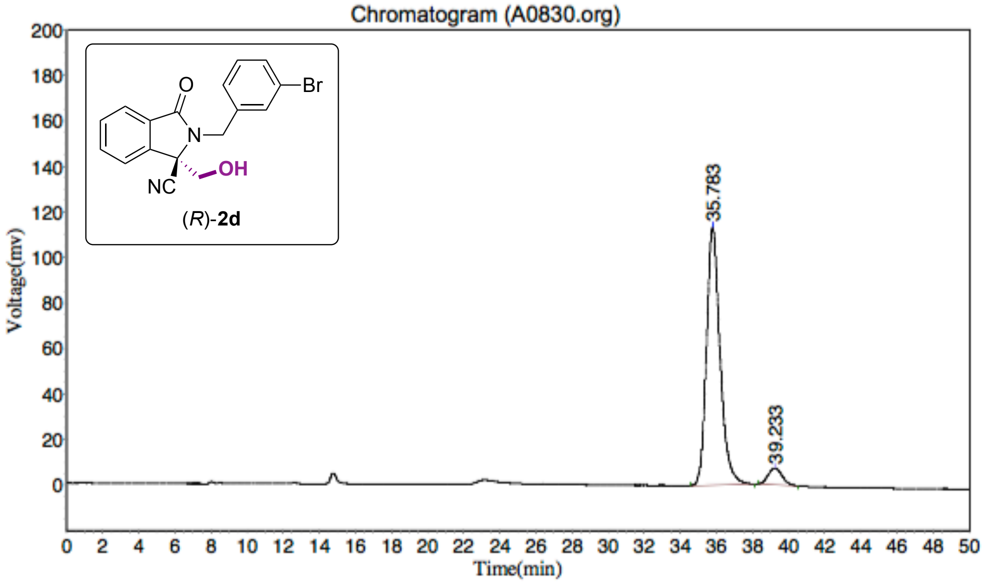

Results

Peak No.	Peak ID	Ret Time	Height	Area	Conc.
1		35.783	113188.727	5900379.000	93.7538
2		39.233	7286.004	393100.719	6.2462
**Total**			120474.731	6293479.719	100.0000

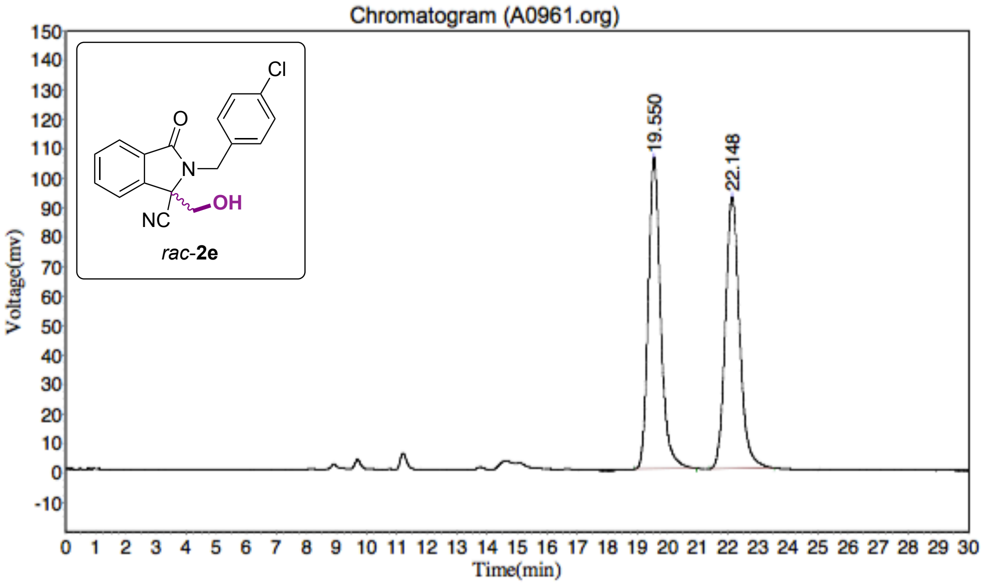

Results

Peak No.	Peak ID	Ret Time	Height	Area	Conc.
1		19.550	105623.617	3014137.500	49.9428
2		22.148	92134.453	3021036.250	50.0572
**Total**			197758.070	6035173.750	100.0000

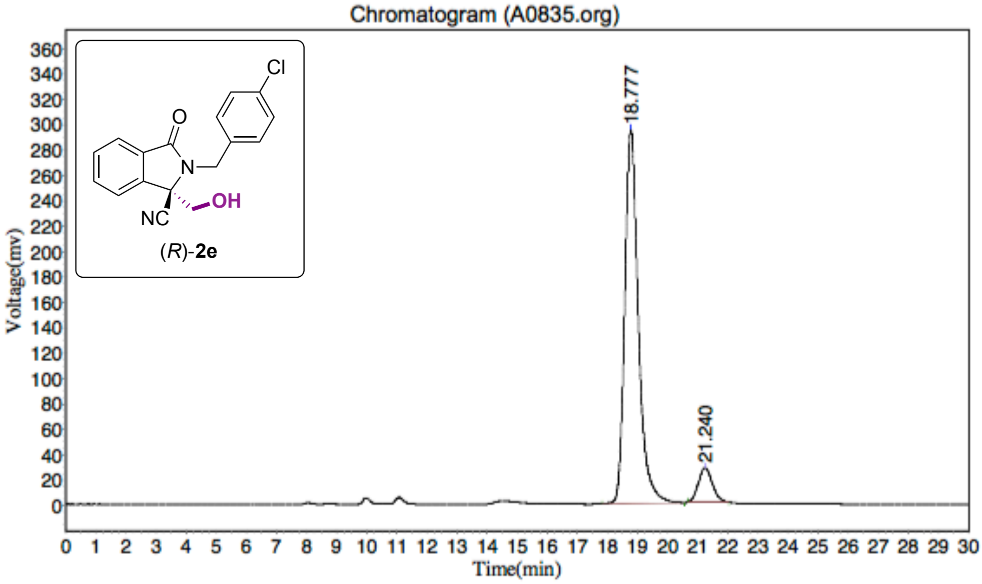

Results

Peak No.	Peak ID	Ret Time	Height	Area	Conc.
1		18.777	294763.656	9192952.000	91.3700
2		21.240	26848.654	868279.063	8.6299
**Total**			321612.311	10061231.063	100.0000

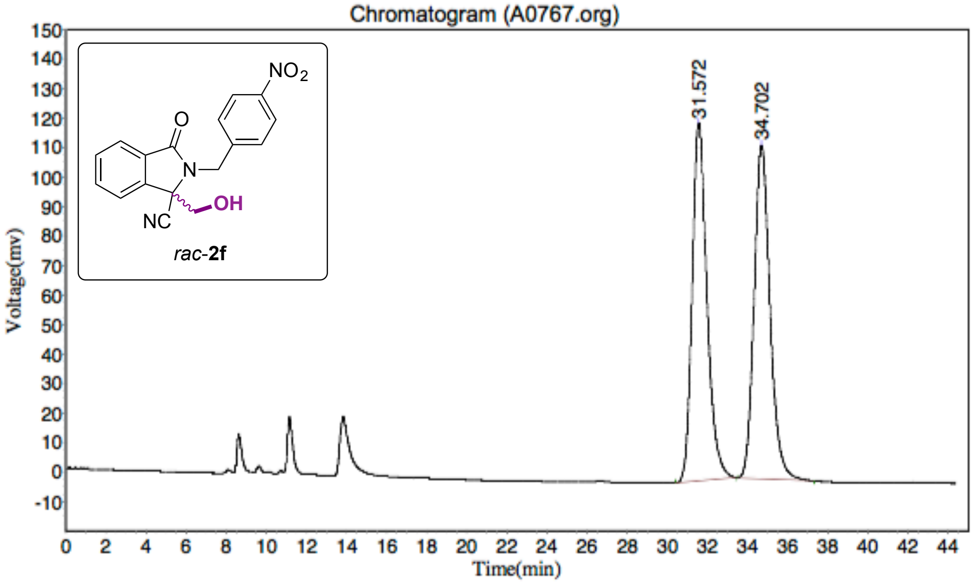

Results

Peak No.	Peak ID	Ret Time	Height	Area	Conc.
1		31.572	121271.234	6211134.000	49.8946
2		34.702	113173.758	6237381.500	50.1054
**Total**			234444.992	12448515.500	100.0000

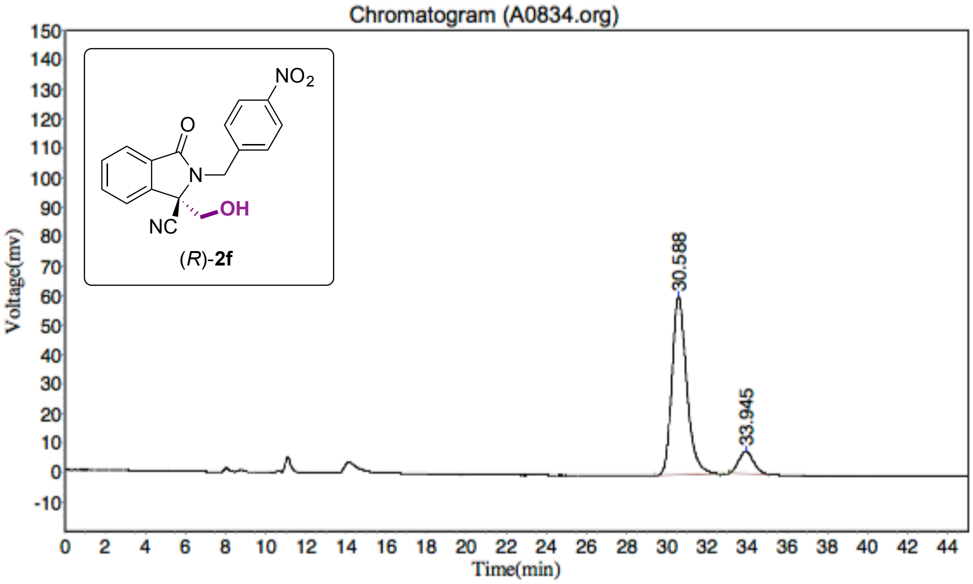

Results

Peak No.	Peak ID	Ret Time	Height	Area	Conc.
1		30.588	60651.125	3101986.500	88.9998
2		33.945	7540.388	383398.406	11.0002
**Total**			68191.513	3485384.906	100.0000

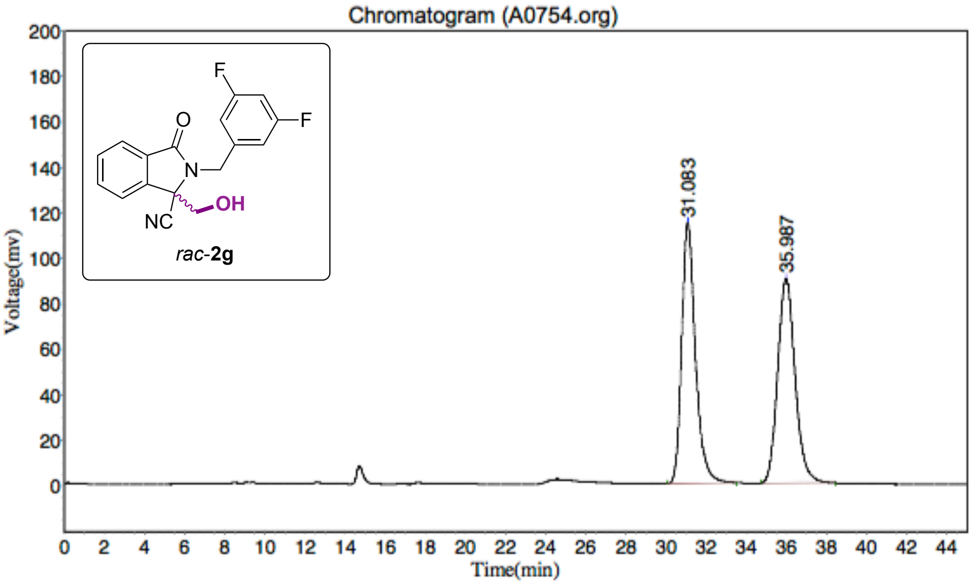

Results

Peak No.	Peak ID	Ret Time	Height	Area	Conc.
1		31.083	114763.508	5420200.000	50.0909
2		35.987	89823.695	5400535.500	49.9091
**Total**			204587.203	10820735.500	100.0000

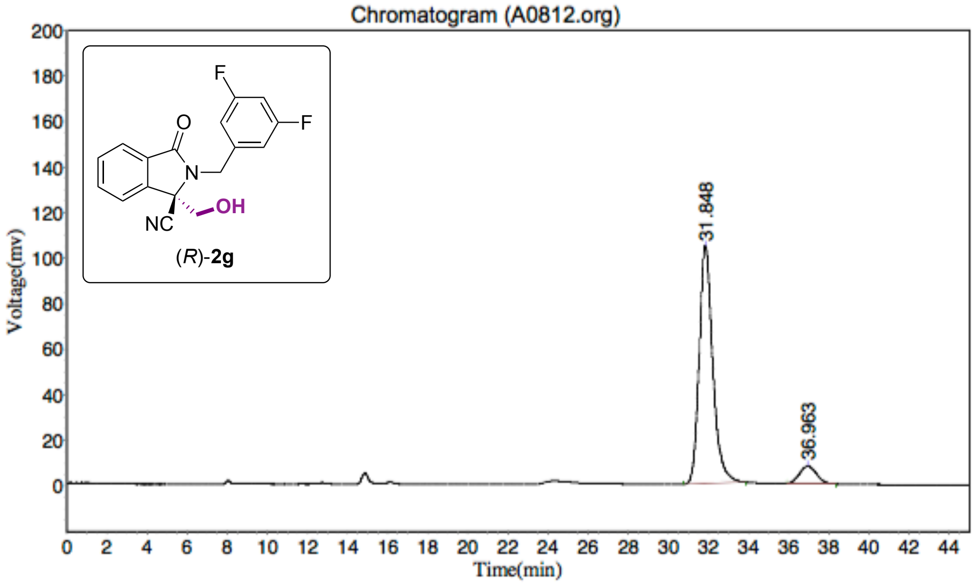

Results

Peak No.	Peak ID	Ret Time	Height	Area	Conc.
1		31.848	104314.188	4900048.500	91.5762
2		36.963	7709.595	450741.375	8.4238
**Total**			112023.782	5350789.875	100.0000

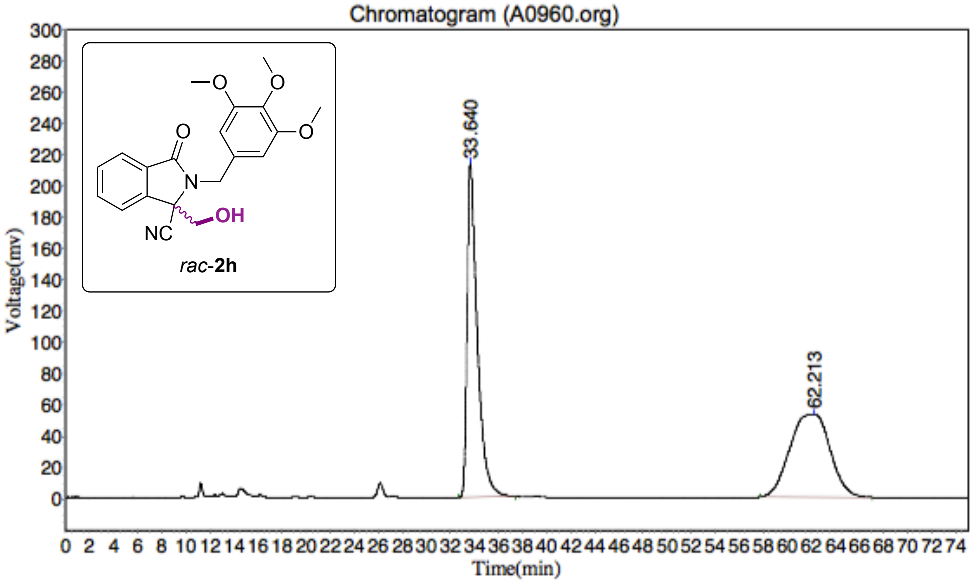

Results

Peak No.	Peak ID	Ret Time	Height	Area	Conc.
1		33.640	213600.953	12515621.000	50.4414
2		62.213	52848.664	12296556.000	49.5586
**Total**			266449.617	24812177.000	100.0000

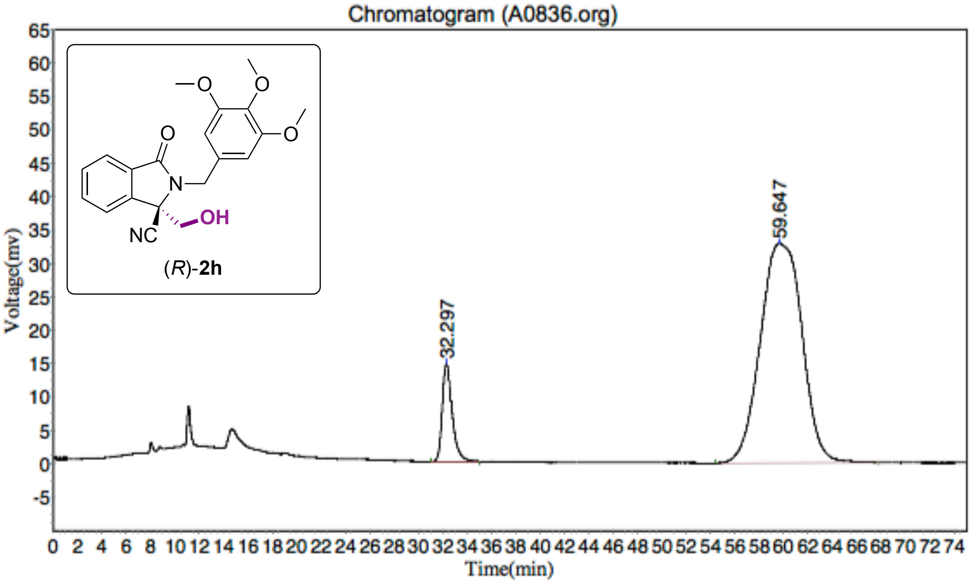

Results

Peak No.	Peak ID	Ret Time	Height	Area	Conc.
1		32.297	14676.595	822059.750	9.4839
2		59.647	32791.602	7845913.500	90.5161
**Total**			47468.196	8667973.250	100.0000

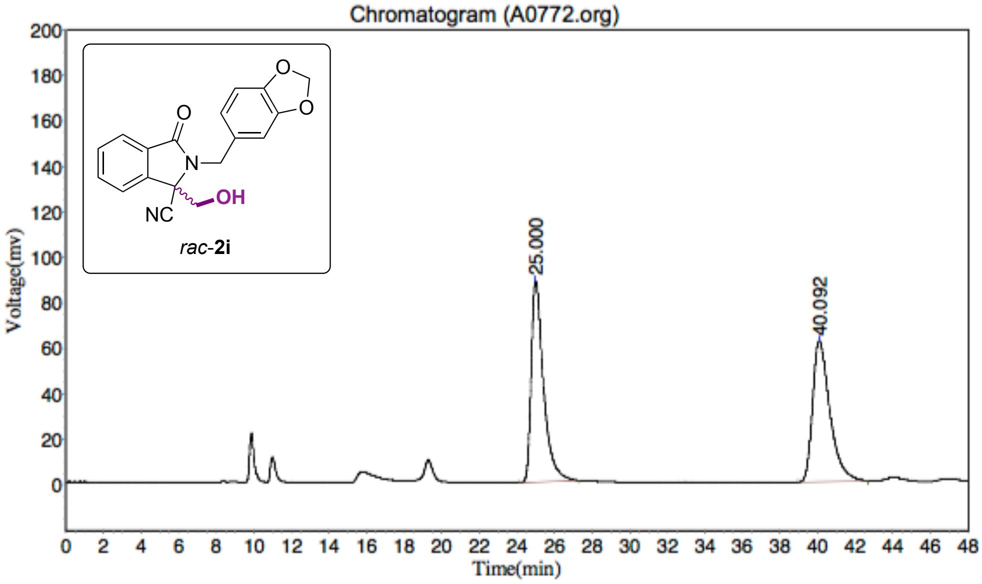

Results

Peak No.	Peak ID	Ret Time	Height	Area	Conc.
1		25.000	88206.867	3986668.000	50.1323
2		40.092	62001.242	3965631.000	49.8677
**Total**			150208.109	7952299.000	100.0000

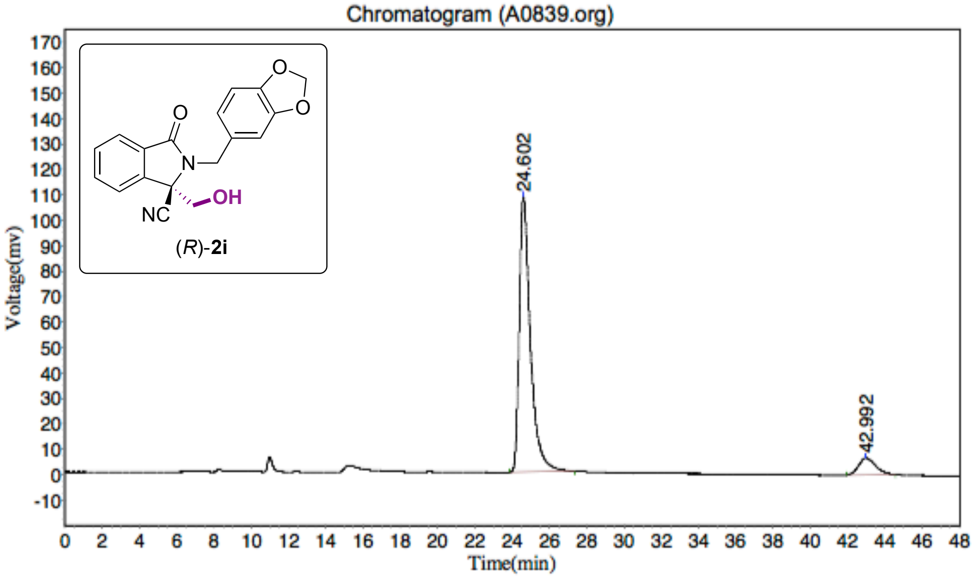

Results

Peak No.	Peak ID	Ret Time	Height	Area	Conc.
1		24.602	108367.648	4508518.000	91.5653
2		42.992	6613.124	415308.844	8.4347
**Total**			114980.772	4923826.844	100.0000

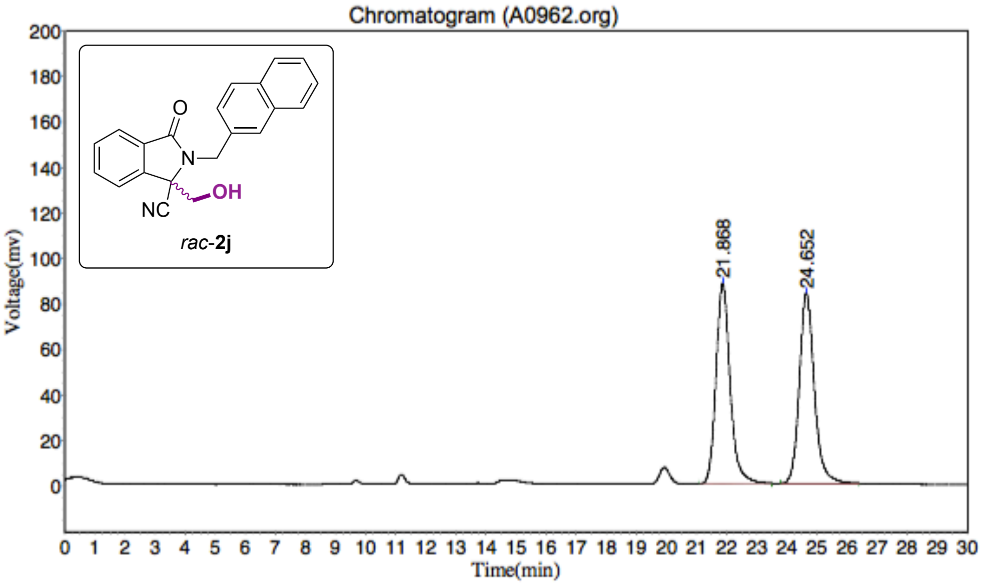

Results

Peak No.	Peak ID	Ret Time	Height	Area	Conc.
1		21.868	88177.234	2874062.750	49.9487
2		24.652	83682.648	2879970.000	50.0513
**Total**			171859.883	5754032.750	100.0000

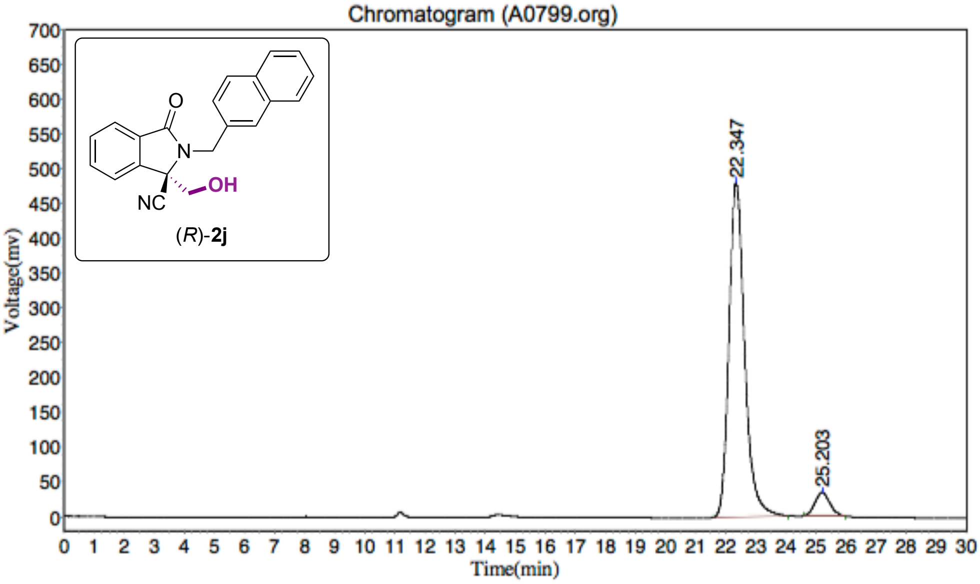

Results

Peak No.	Peak ID	Ret Time	Height	Area	Conc.
1		22.347	480671.000	17290450.000	93.7172
2		25.203	33706.242	1159158.875	6.2828
**Total**			514377.242	18449608.875	100.0000

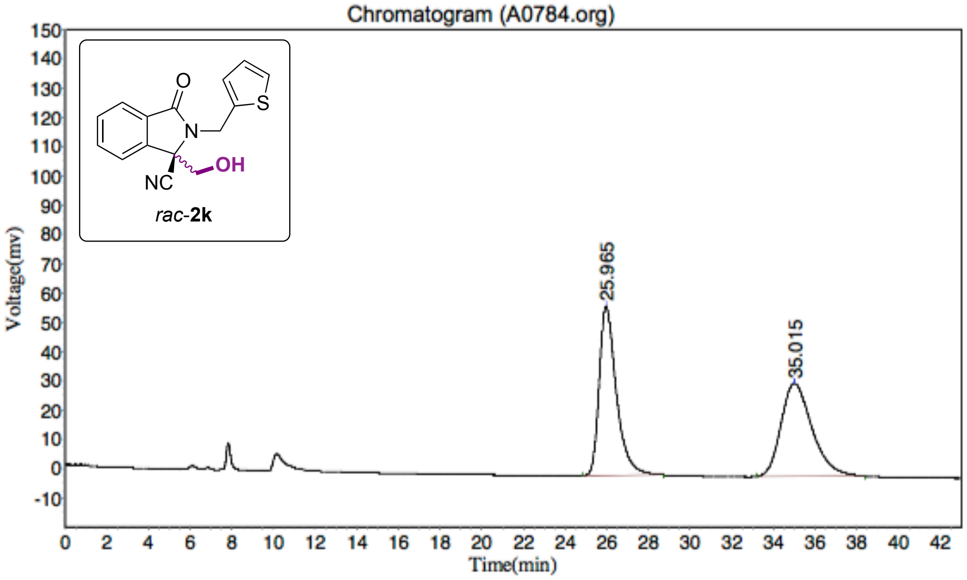

Results

Peak No.	Peak ID	Ret Time	Height	Area	Conc.
1		25.965	57966.598	3319458.250	50.1918
2		35.015	31830.410	3294090.000	49.8082
**Total**			89797.008	6613548.250	100.0000

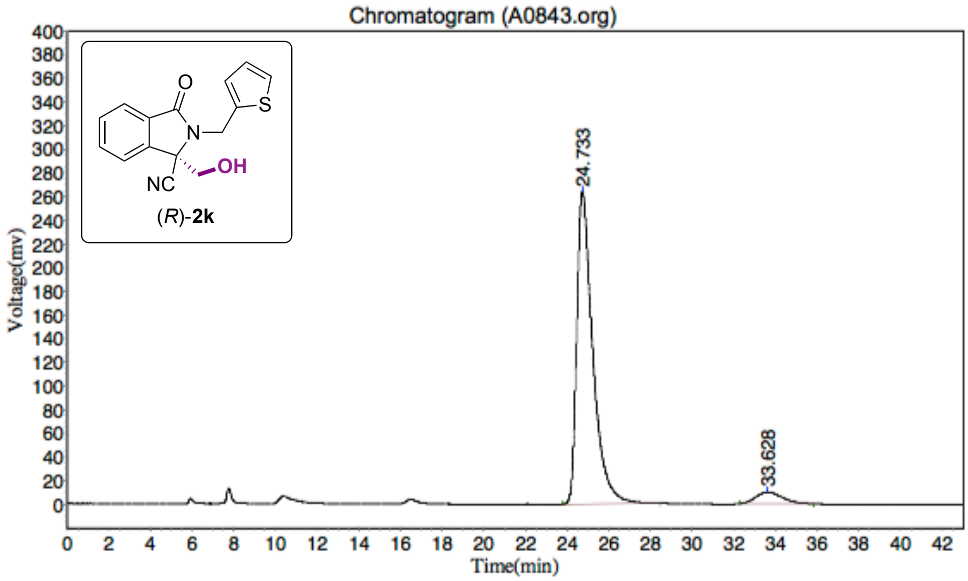

Results

Peak No.	Peak ID	Ret Time	Height	Area	Conc.
1		24.733	263877.688	14054636.000	94.0285
2		33.628	9942.692	892580.625	5.9716
**Total**			273820.380	14947216.625	100.0000

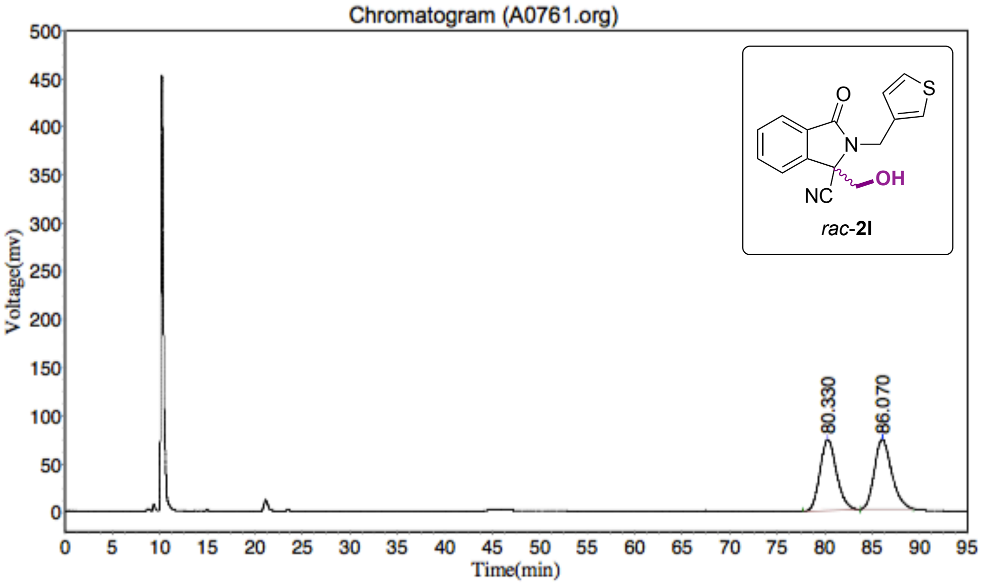

Results

Peak No.	Peak ID	Ret Time	Height	Area	Conc.
1		80.330	73810.969	8933852.000	49.4546
2		86.070	72723.000	9130902.000	50.5454
**Total**			146533.969	18064754.000	100.0000

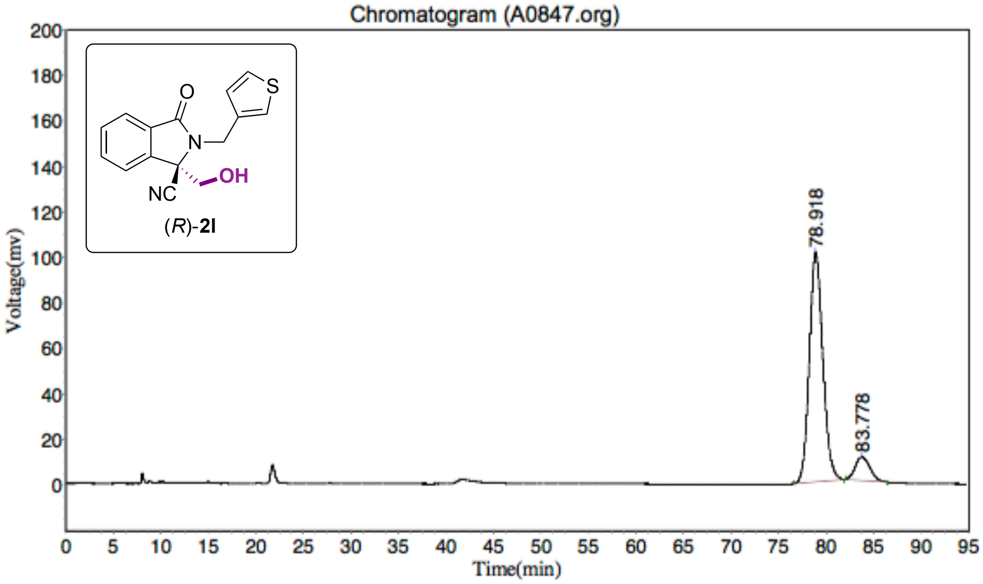

Results

Peak No.	Peak ID	Ret Time	Height	Area	Conc.
1		78.918	100620.828	9921948.000	89.9673
2		83.778	10401.838	1106441.625	10.0327
**Total**			111022.666	11028389.625	100.0000

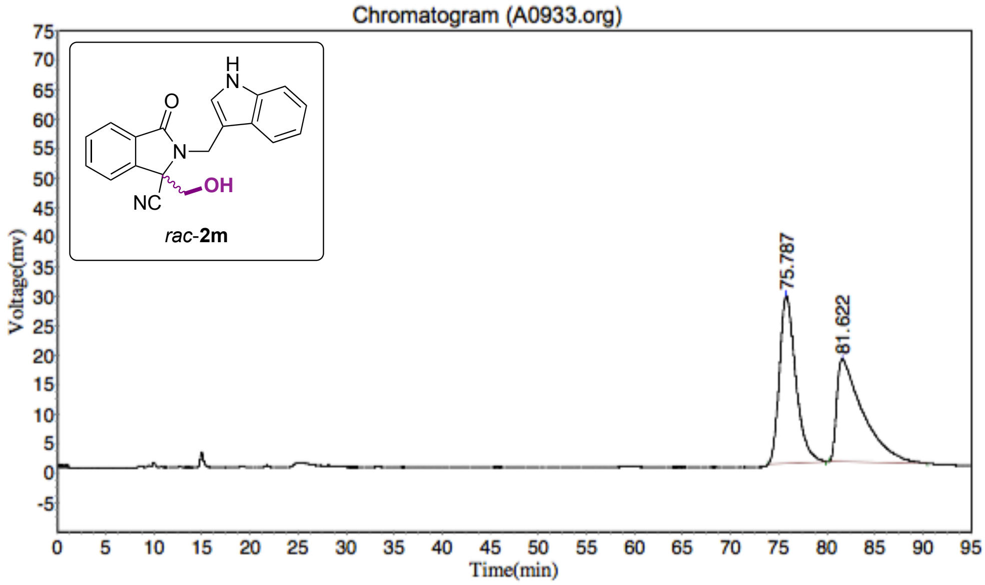

Results

Peak No.	Peak ID	Ret Time	Height	Area	Conc.
1		75.787	28419.234	3350220.000	50.4299
2		81.622	17374.340	3293106.500	49.5701
**Total**			45793.574	6643326.500	100.0000

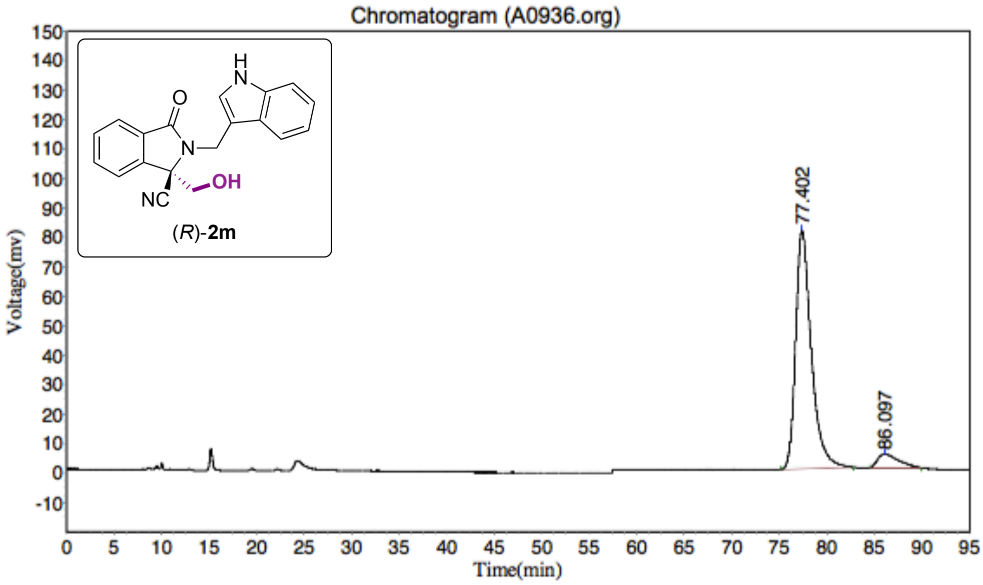

Results

Peak No.	Peak ID	Ret Time	Height	Area	Conc.
1		77.402	80971.359	9306112.000	92.9056
2		86.097	4554.412	710631.875	7.0944
**Total**			85525.771	10016743.875	100.0000

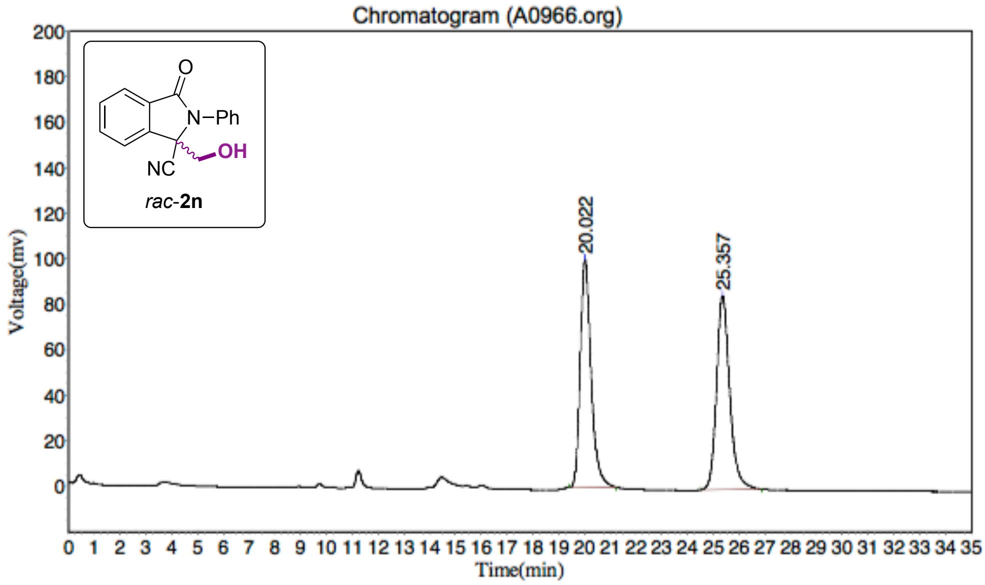

Results

Peak No.	Peak ID	Ret Time	Height	Area	Conc.
1		20.022	100252.938	2929840.000	49.3168
2		25.357	85075.906	3011012.000	50.6832
**Total**			185328.844	5940852.000	100.0000

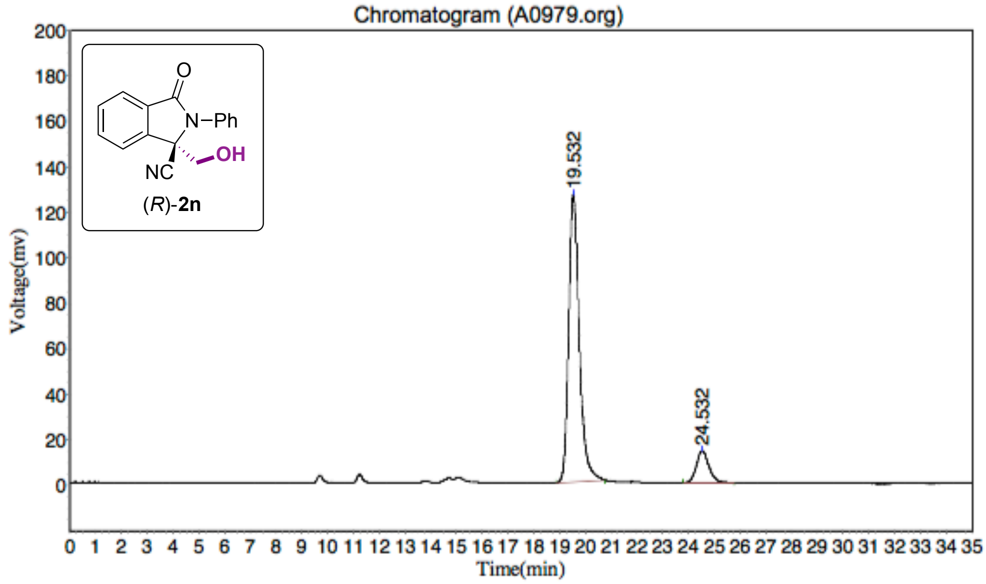

Results

Peak No.	Peak ID	Ret Time	Height	Area	Conc.
1		19.532	126192.383	3613204.000	88.3520
2		24.532	14042.730	476350.969	11.6480
**Total**			140235.113	4089554.969	100.0000

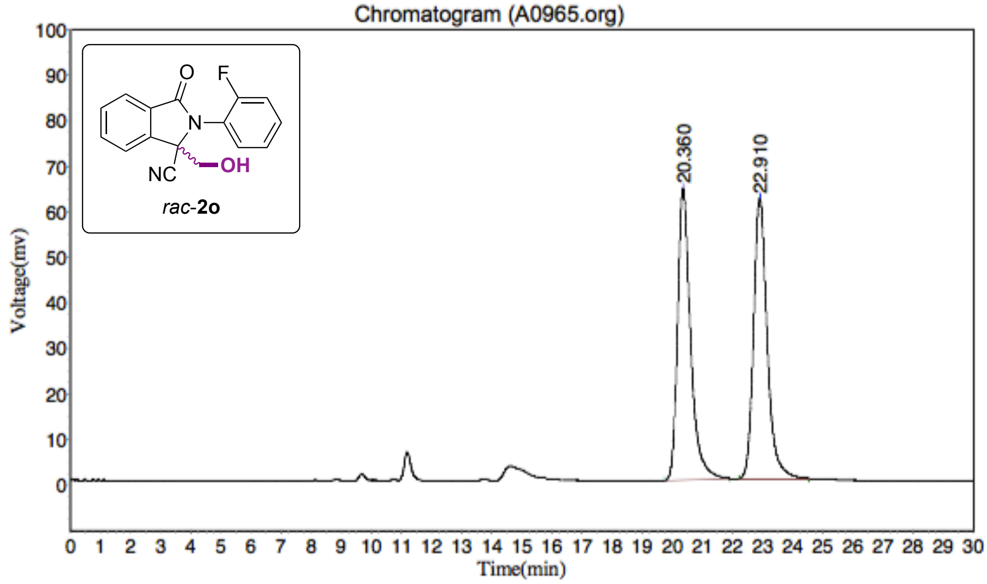

Results

Peak No.	Peak ID	Ret Time	Height	Area	Conc.
1		20.360	64051.609	1920619.375	49.9055
2		22.910	61549.941	1927895.375	50.0945
**Total**			125601.551	3848514.750	100.0000

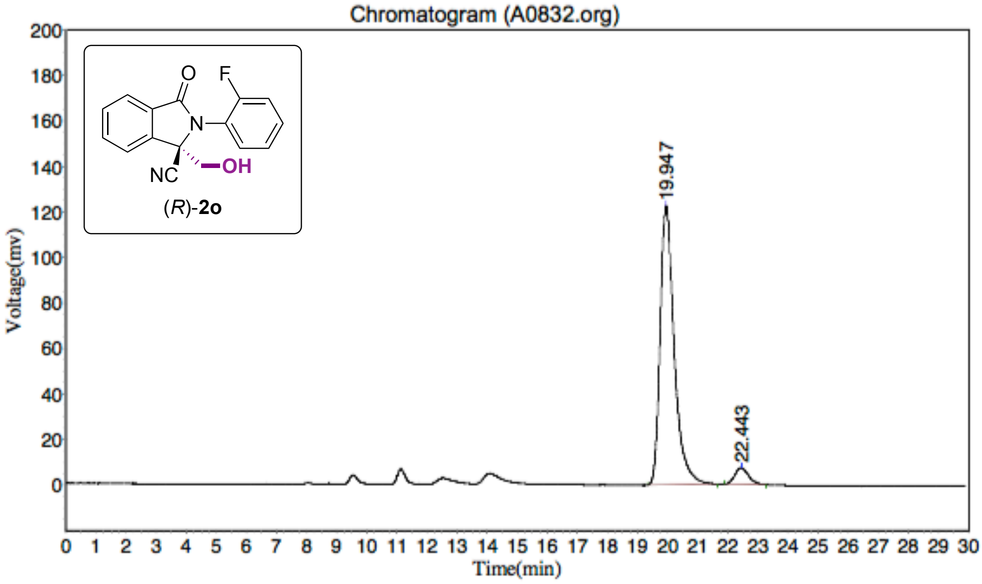

Results

Peak No.	Peak ID	Ret Time	Height	Area	Conc.
1		19.947	122607.484	3971848.000	94.5296
2		22.443	7251.713	229848.594	5.4704
**Total**			129859.198	4201696.594	100.0000

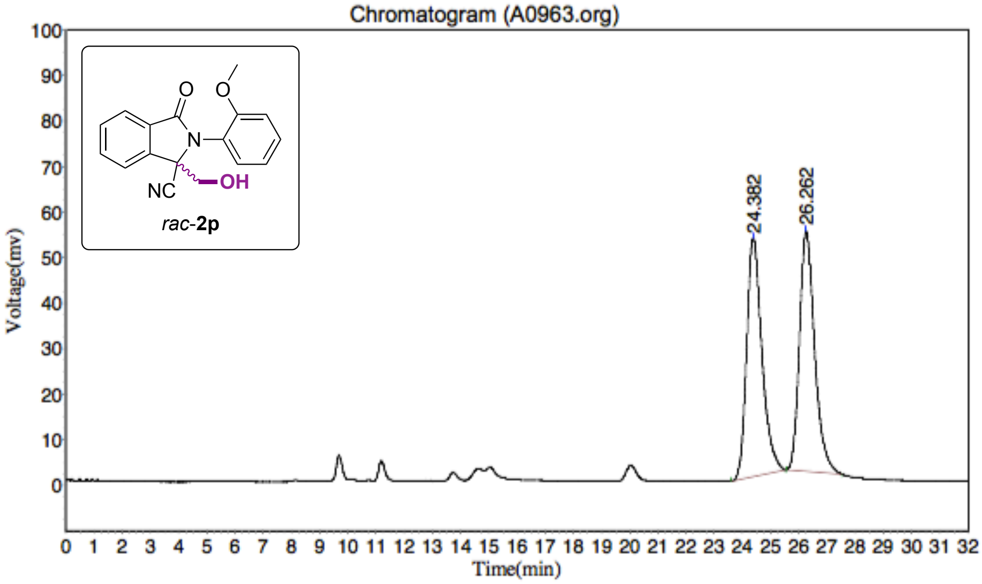

Results

Peak No.	Peak ID	Ret Time	Height	Area	Conc.
1		24.382	52383.145	1962074.375	49.8350
2		26.262	52738.781	1975070.125	50.1650
**Total**			105121.926	3937144.500	100.0000

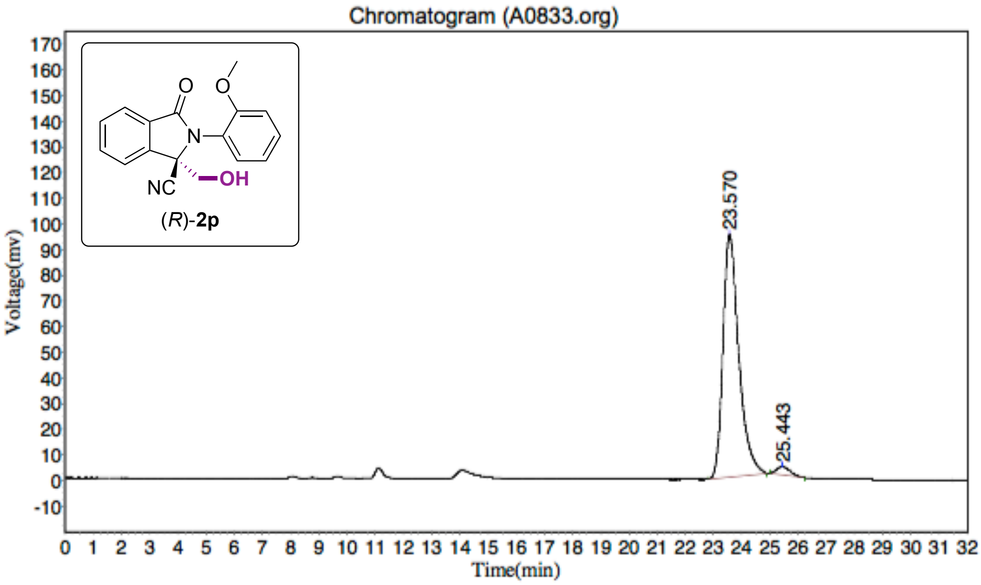

Results

Peak No.	Peak ID	Ret Time	Height	Area	Conc.
1		23.570	94471.758	3675589.000	97.2830
2		25.443	3162.911	102653.945	2.7170
**Total**			97634.668	3778242.945	100.0000

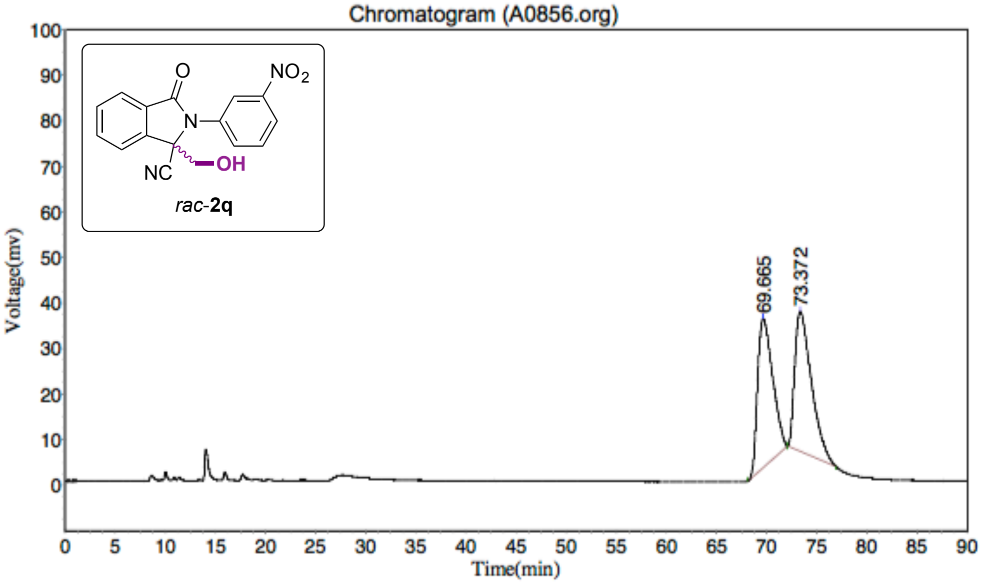

Results

Peak No.	Peak ID	Ret Time	Height	Area	Conc.
1		69.665	32629.166	3409428.500	49.0584
2		73.372	30547.525	3540310.250	50.9416
**Total**			63176.691	6949738.750	100.0000

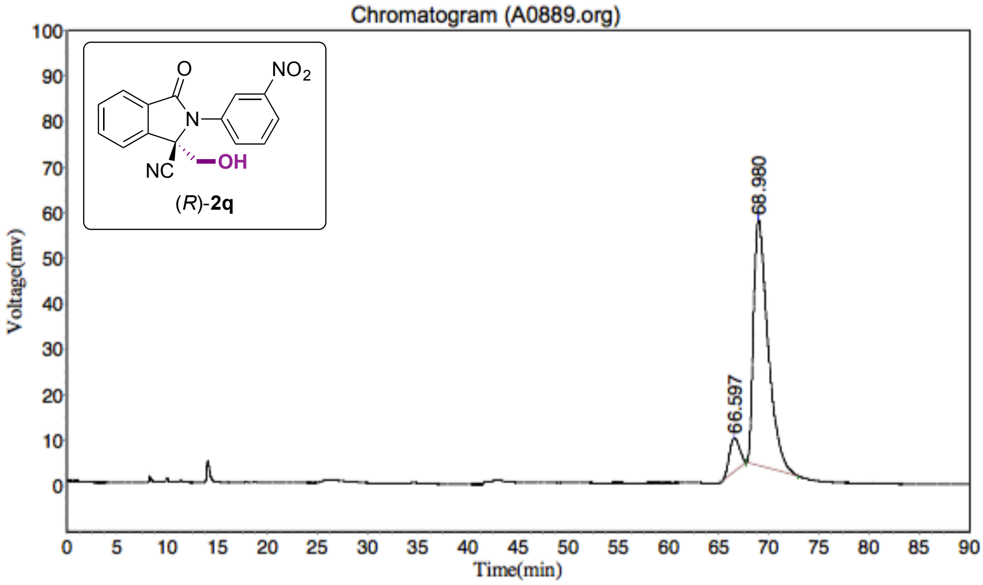

Results

Peak No.	Peak ID	Ret Time	Height	Area	Conc.
1		66.597	7319.977	519337.000	8.7986
2		68.980	53986.590	5383125.500	91.2013
**Total**			61306.567	5902462.500	100.0000

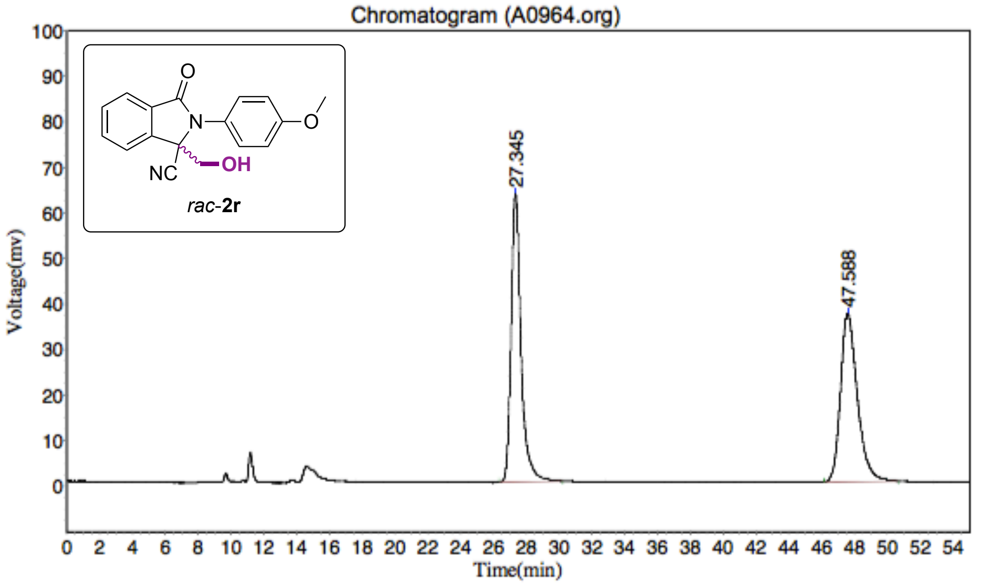

Results

Peak No.	Peak ID	Ret Time	Height	Area	Conc.
1		27.345	63348.598	2664700.000	50.4354
2		47.588	36914.586	2618688.000	49.5646
**Total**			100263.184	5283388.000	100.0000

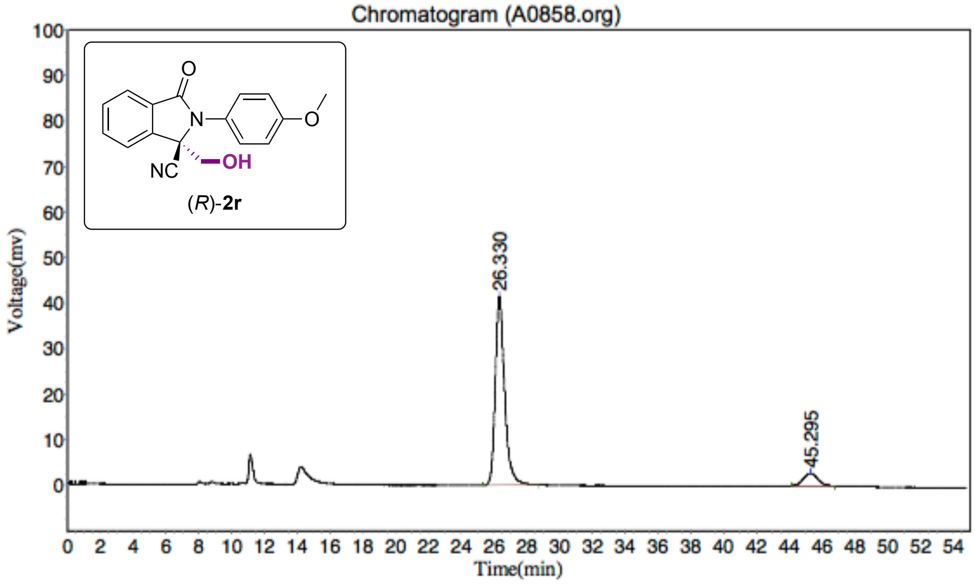

Results

Peak No.	Peak ID	Ret Time	Height	Area	Conc.
1		26.330	41519.648	1642283.250	90.4787
2		45.295	2805.697	172820.953	9.5213
**Total**			44325.345	1815104.203	100.0000

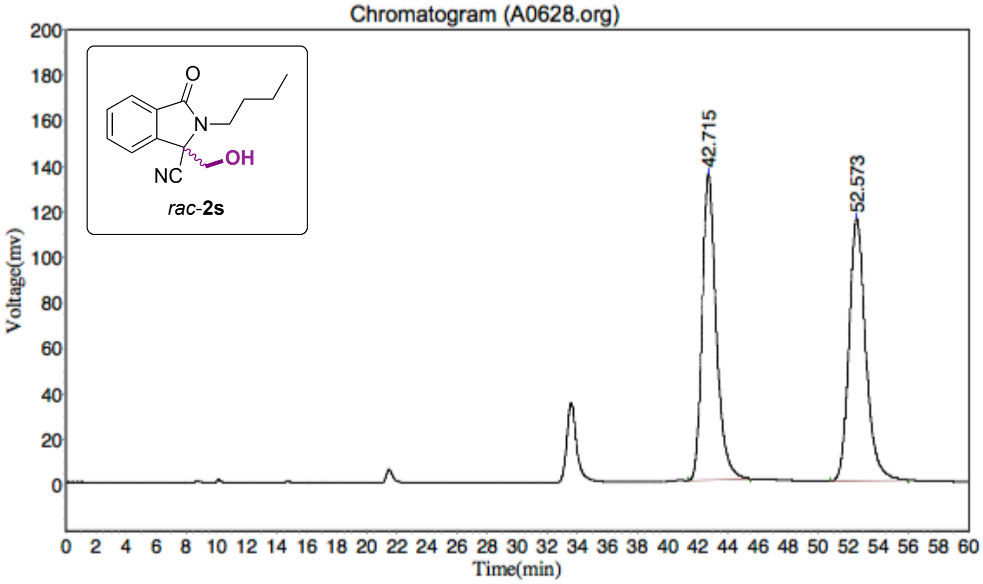

Results

Peak No.	Peak ID	Ret Time	Height	Area	Conc.
1		42.715	134351.688	8716123.000	49.5694
2		52.573	115313.625	8867554.000	50.4306
**Total**			249665.313	17583677.000	100.0000

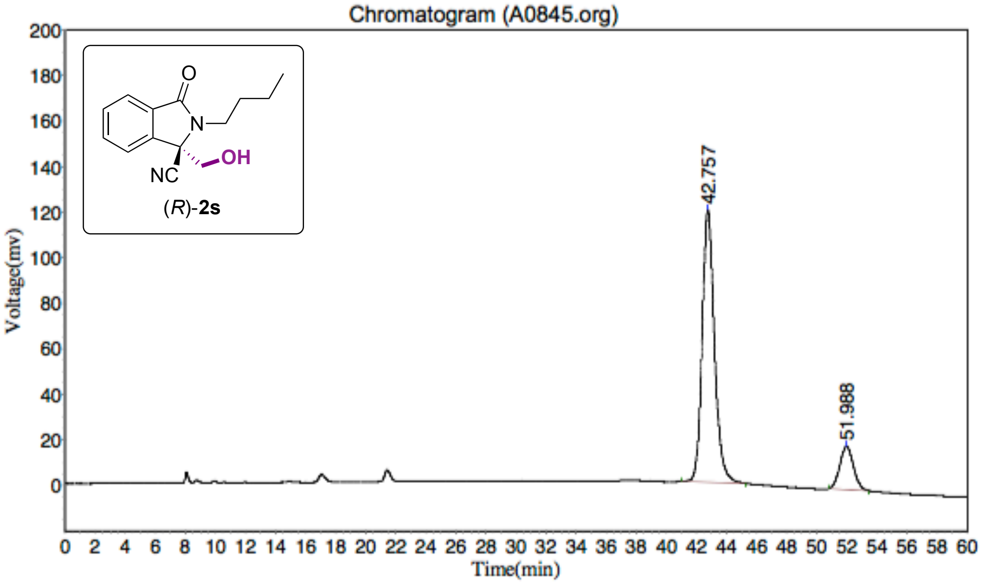

Results

Peak No.	Peak ID	Ret Time	Height	Area	Conc.
1		42.757	119620.258	6760169.500	84.9647
2		51.988	18991.357	1196276.750	15.0353
**Total**			138611.615	7956446.250	100.0000

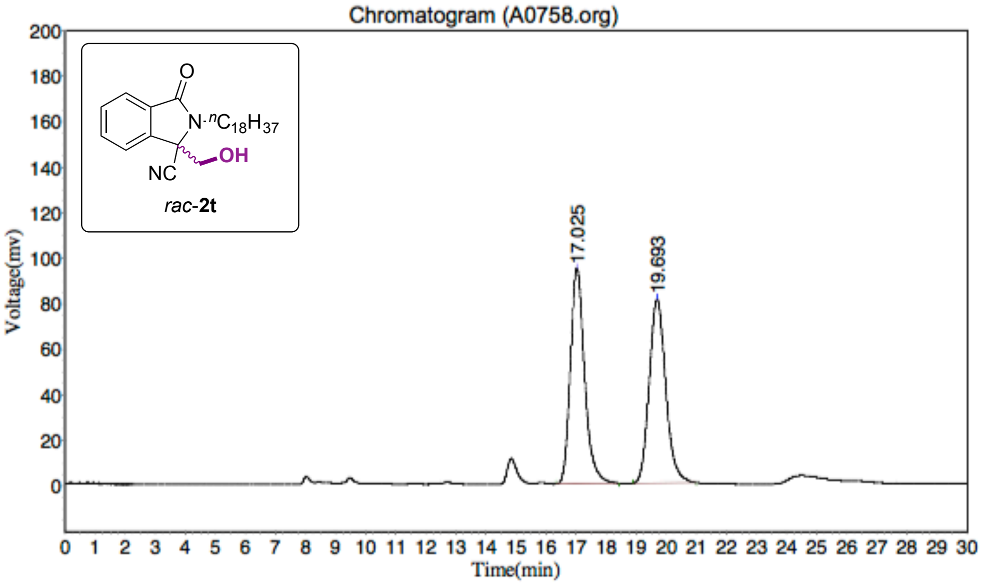

Results

Peak No.	Peak ID	Ret Time	Height	Area	Conc.
1		17.025	94341.563	3099644.000	50.1166
2		19.693	80712.844	3085223.750	49.8834
**Total**			175054.406	6184867.750	100.0000

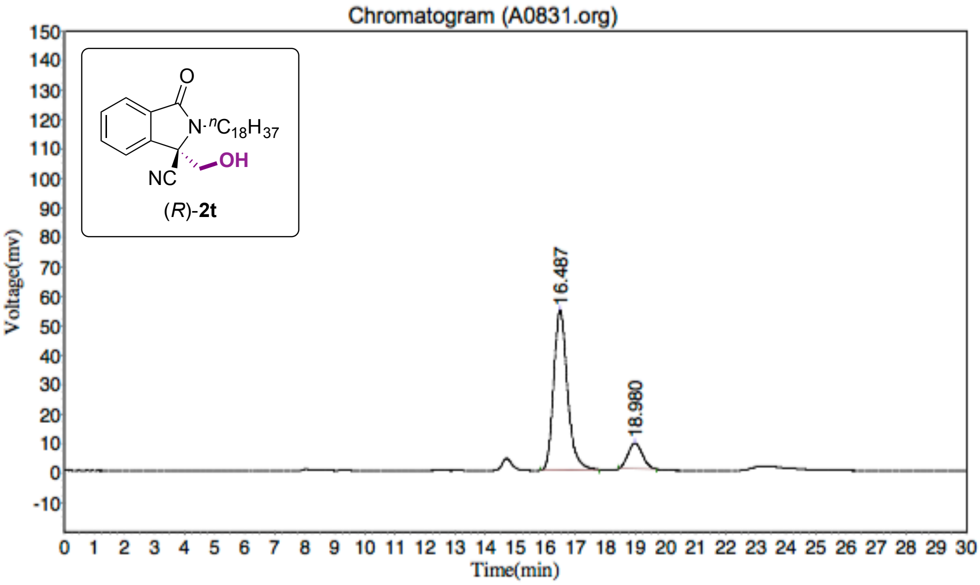

Results

Peak No.	Peak ID	Ret Time	Height	Area	Conc.
1		16.487	54311.145	1730044.250	85.6588
2		18.980	8638.674	289648.313	14.3412
**Total**			62949.818	2019692.563	100.0000

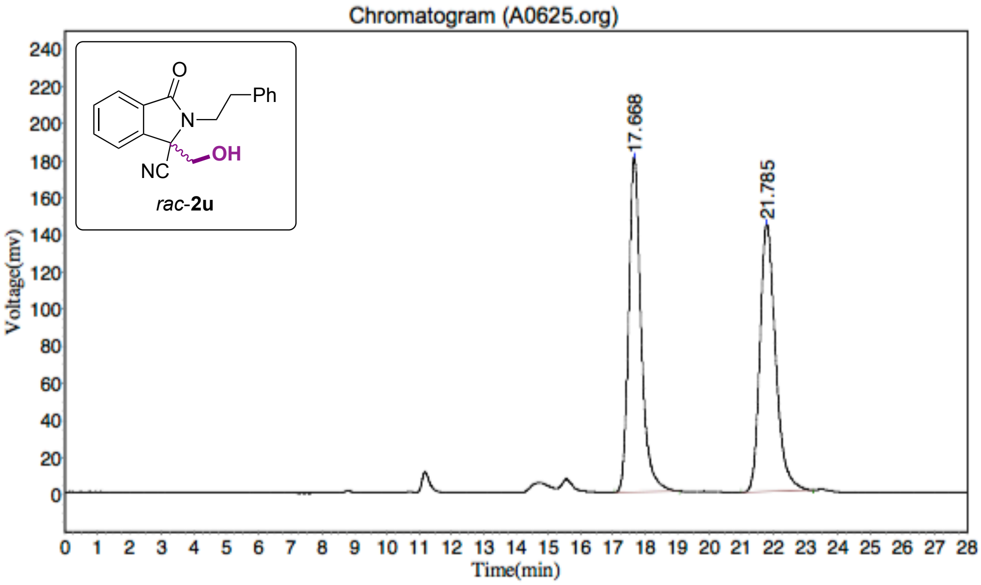

Results

Peak No.	Peak ID	Ret Time	Height	Area	Conc.
1		17.668	180170.734	4877846.000	50.0046
2		21.785	144132.484	4876953.000	49.9954
**Total**			324303.219	9754799.000	100.0000

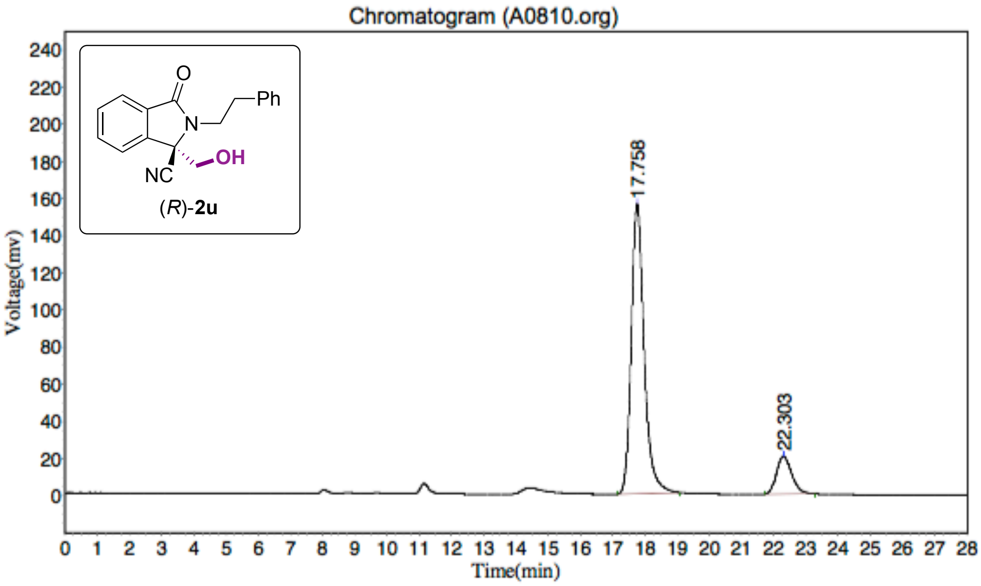

Results

Peak No.	Peak ID	Ret Time	Height	Area	Conc.
1		17.758	156236.422	4339542.500	86.6296
2		22.303	20448.877	669761.938	13.3704
**Total**			176685.299	5009304.438	100.0000

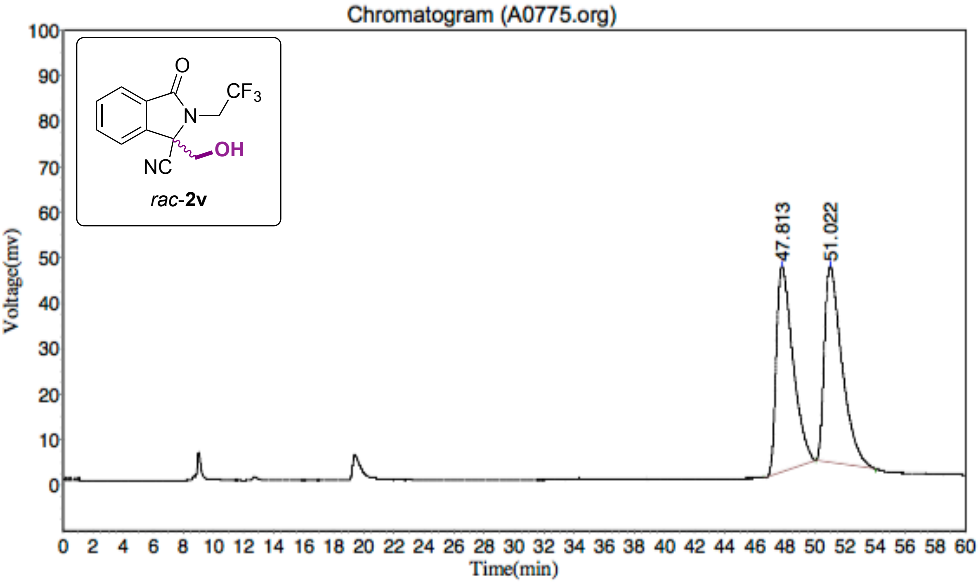

Results

Peak No.	Peak ID	Ret Time	Height	Area	Conc.
1		47.813	45238.805	3468110.250	49.6589
2		51.022	43016.098	3515749.250	50.3411
**Total**			88254.902	6983859.500	100.0000

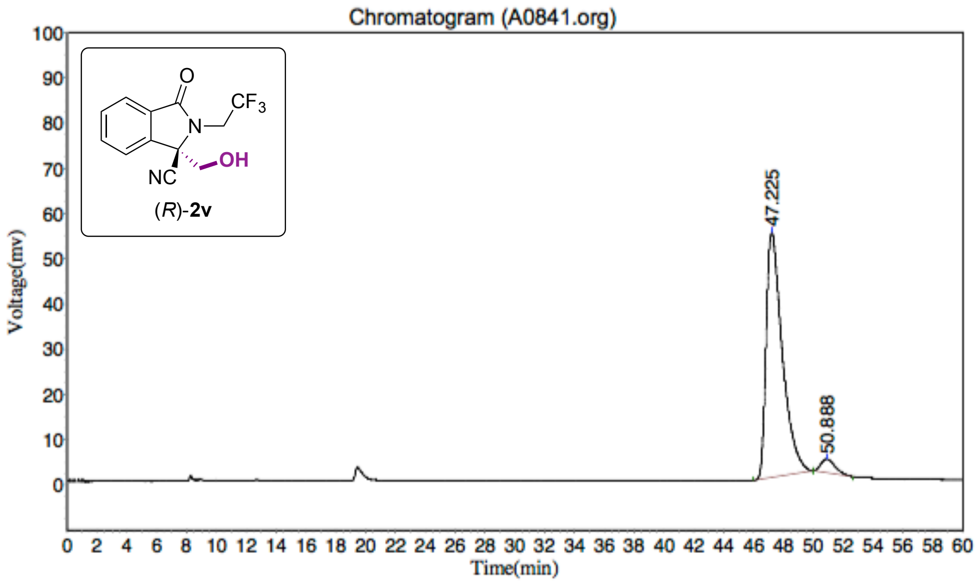

Results

Peak No.	Peak ID	Ret Time	Height	Area	Conc.
1		47.225	54134.359	4053877.750	95.1462
2		50.888	3066.611	206803.578	4.8538
**Total**			57200.970	4260681.328	100.0000

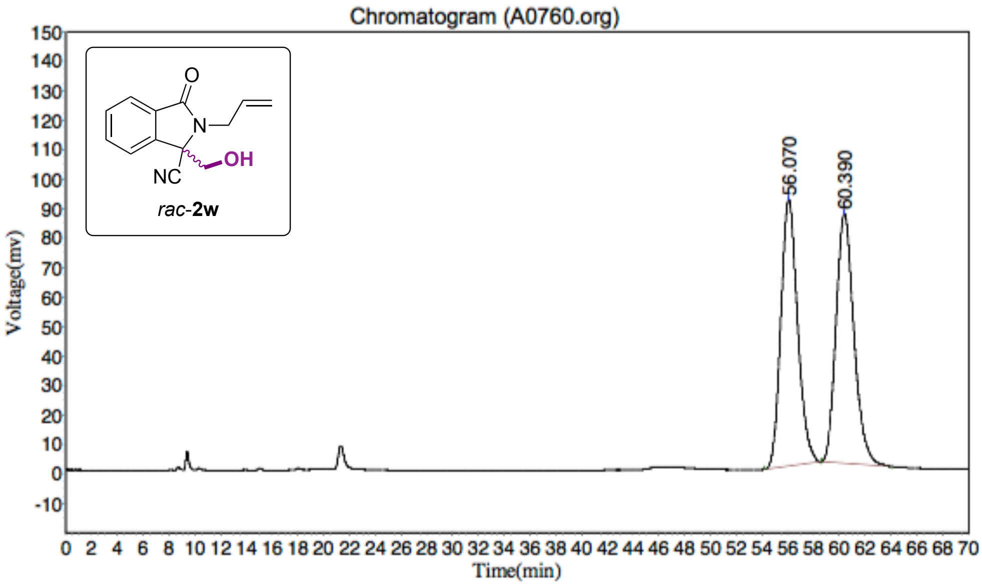

Results

Peak No.	Peak ID	Ret Time	Height	Area	Conc.
1		56.070	90269.117	8138907.000	49.8795
2		60.390	84555.227	8178220.500	50.1205
**Total**			174824.344	16317127.500	100.0000

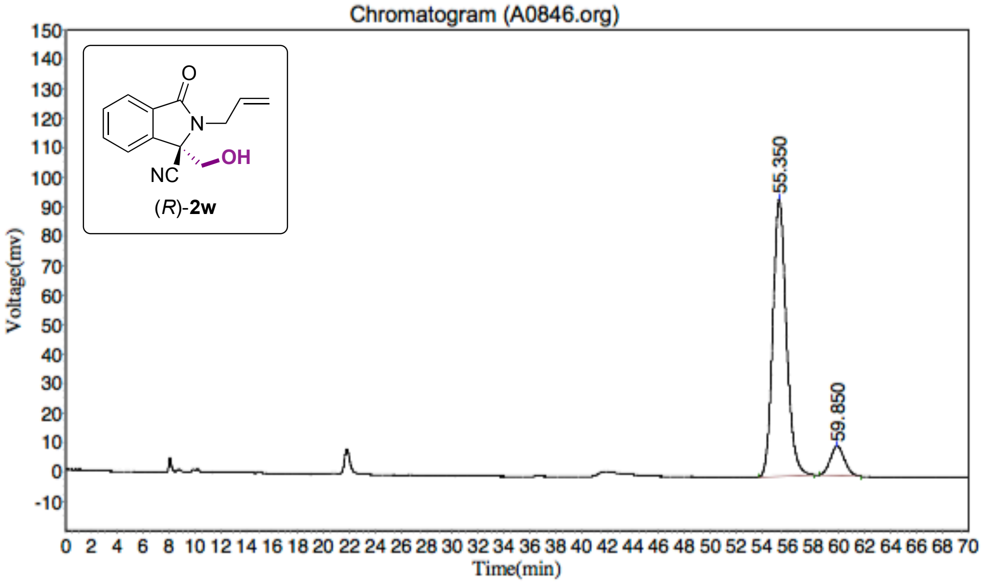

Results

Peak No.	Peak ID	Ret Time	Height	Area	Conc.
1		55.350	94010.813	6869257.000	90.0950
2		59.850	9911.615	755205.688	9.9050
**Total**			103922.428	7624462.688	100.0000

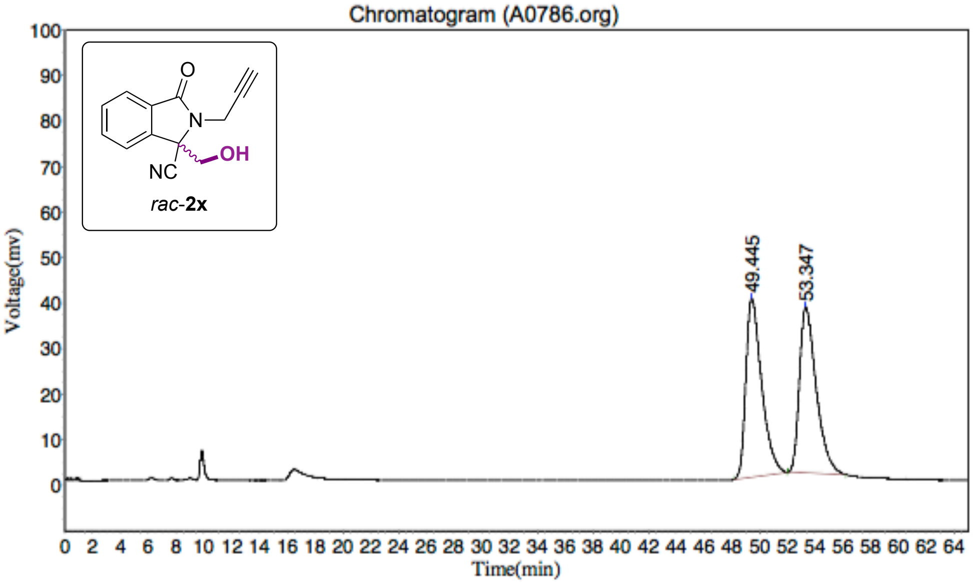

Results

Peak No.	Peak ID	Ret Time	Height	Area	Conc.
1		49.445	39153.223	3082500.250	50.1973
2		53.347	36411.371	3058269.500	49.8027
**Total**			75564.594	6140769.750	100.0000

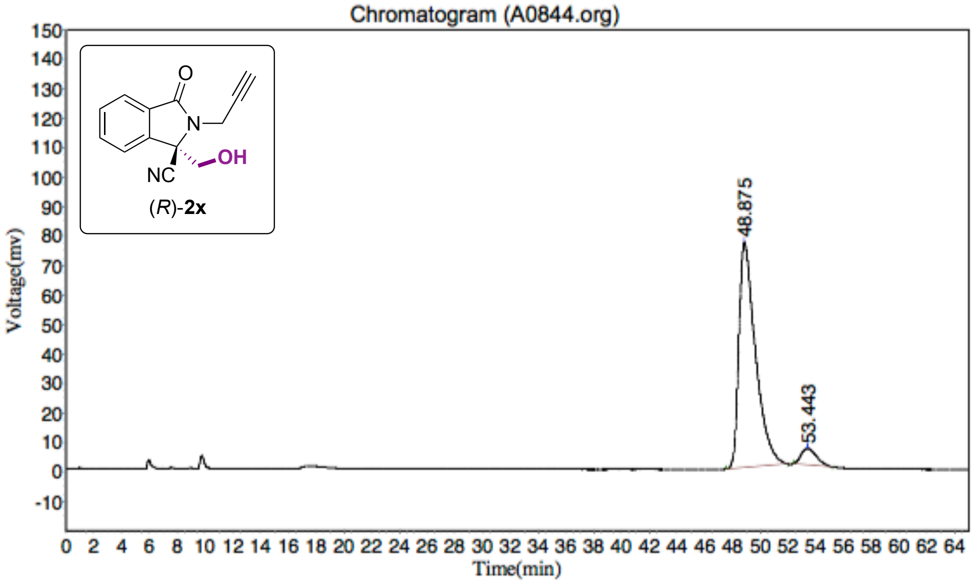

Results

Peak No.	Peak ID	Ret Time	Height	Area	Conc.
1		48.875	76426.383	6326964.500	93.7235
2		53.443	5523.270	423703.844	6.2765
**Total**			81949.652	6750668.344	100.0000

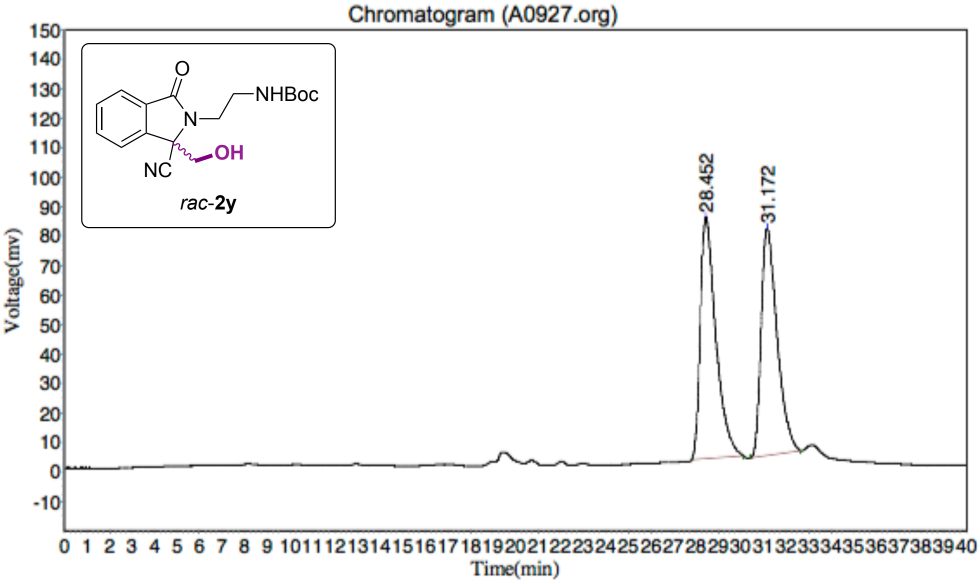

Results

Peak No.	Peak ID	Ret Time	Height	Area	Conc.
1		28.452	81858.422	3907142.250	50.8480
2		31.172	77162.289	3776827.000	49.1520
**Total**			159020.711	7683969.250	100.0000

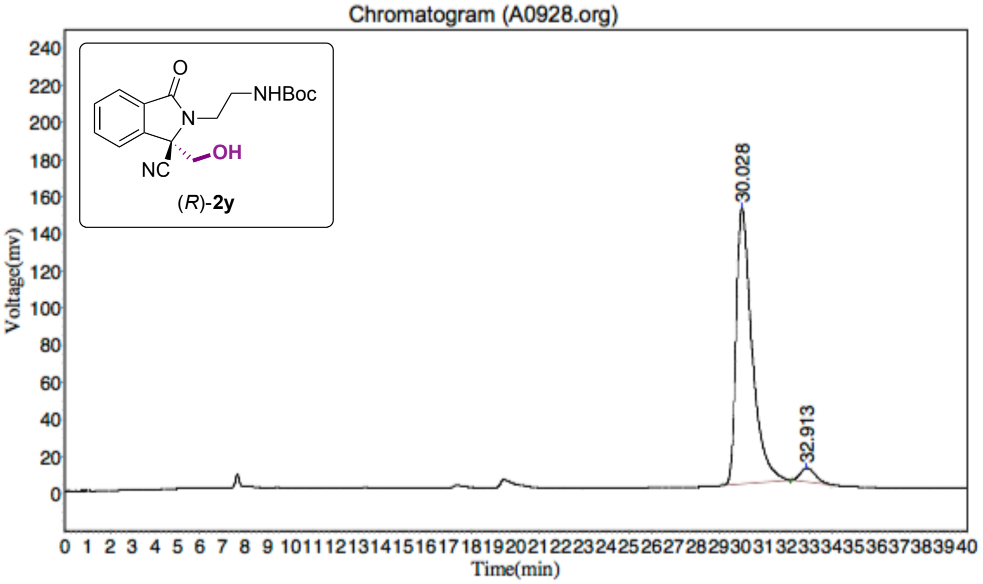

Results

Peak No.	Peak ID	Ret Time	Height	Area	Conc.
1		30.028	148757.750	7242076.000	95.5807
2		32.913	7304.205	334847.844	4.4193
**Total**			156061.955	7576923.844	100.0000

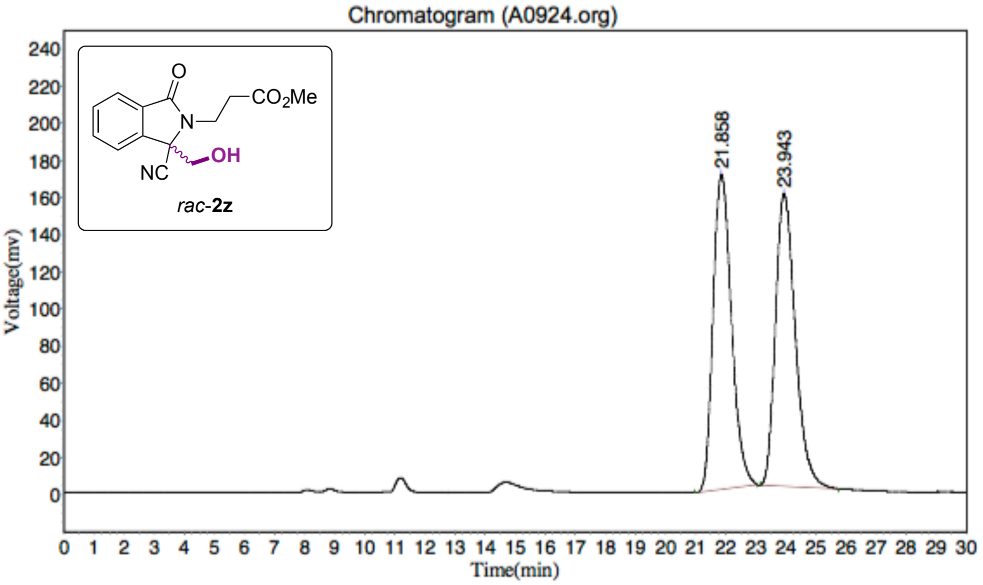

Results

Peak No.	Peak ID	Ret Time	Height	Area	Conc.
1		21.858	169634.125	7198754.000	49.9878
2		23.943	157568.703	7202270.000	50.0122
**Total**			327202.828	14401024.000	100.0000

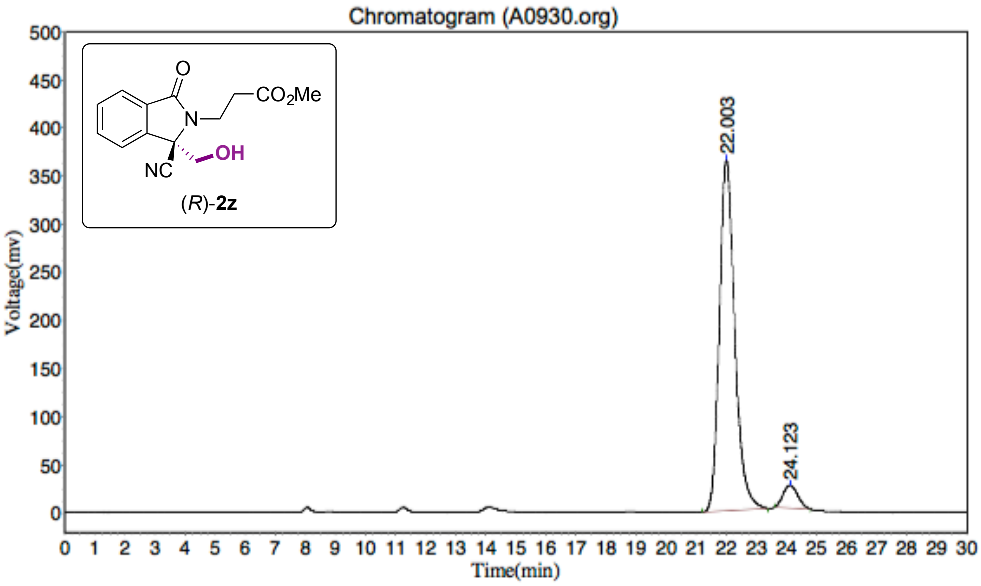

Results

Peak No.	Peak ID	Ret Time	Height	Area	Conc.
1		22.003	364086.219	12960148.000	94.2175
2		24.123	23425.711	795409.125	5.7825
**Total**			387511.930	13755557.125	100.0000

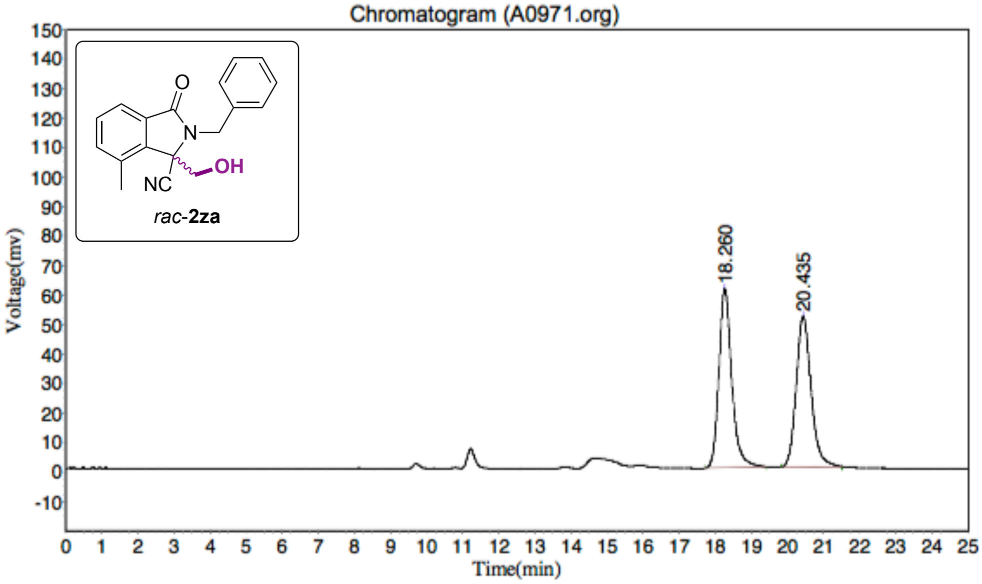

Results

Peak No.	Peak ID	Ret Time	Height	Area	Conc.
1		18.260	60692.000	1507722.875	50.5732
2		20.435	51122.684	1473548.750	49.4269
**Total**			111814.684	2981271.625	100.0000

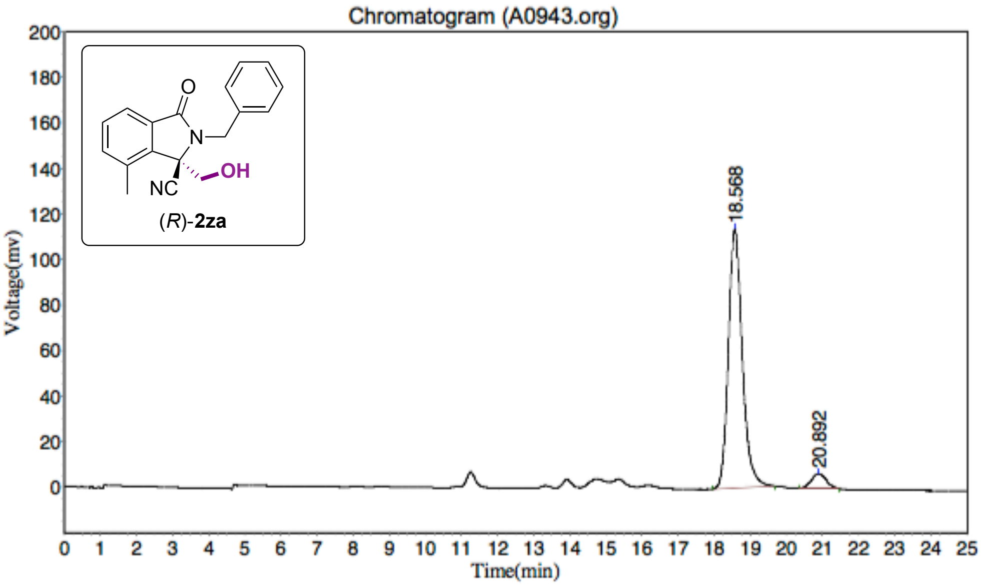

Results

Peak No.	Peak ID	Ret Time	Height	Area	Conc.
1		18.568	113697.680	3027656.750	94.1729
2		20.892	6517.042	187342.609	5.8271
**Total**			120214.722	3214999.359	100.0000

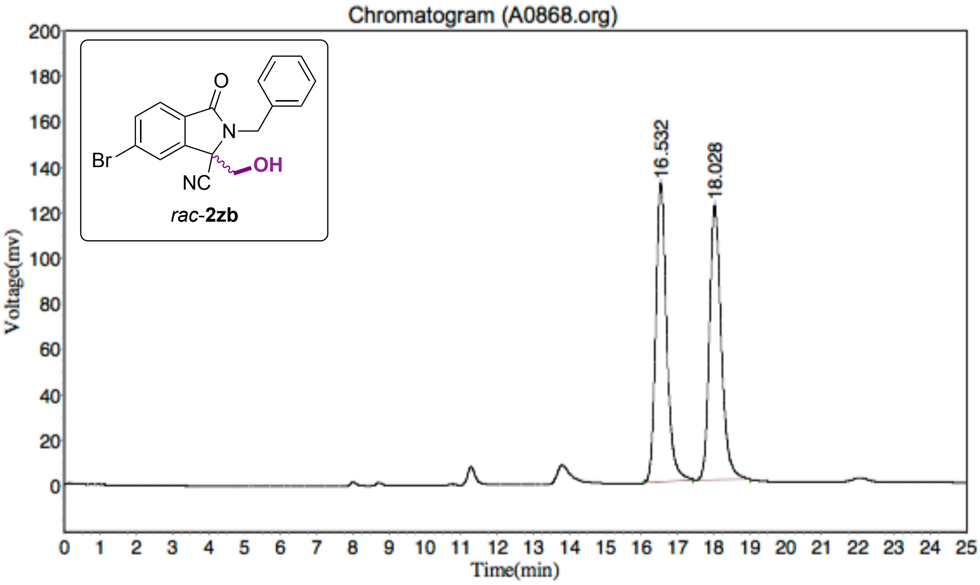

Results

Peak No.	Peak ID	Ret Time	Height	Area	Conc.
1		16.532	130972.844	2730167.000	50.1205
2		18.028	120274.047	2717040.750	49.8795
**Total**			251246.891	5447207.750	100.0000

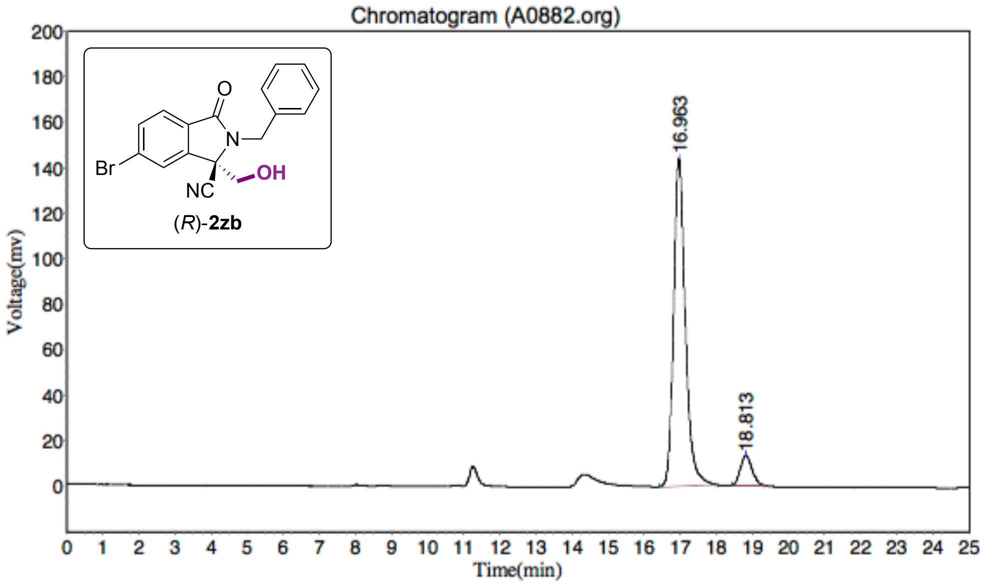

Results

Peak No.	Peak ID	Ret Time	Height	Area	Conc.
1		16.963	143780.078	3249208.000	91.7914
2		18.813	13033.358	290565.188	8.2086
**Total**			156813.437	3539773.188	100.0000

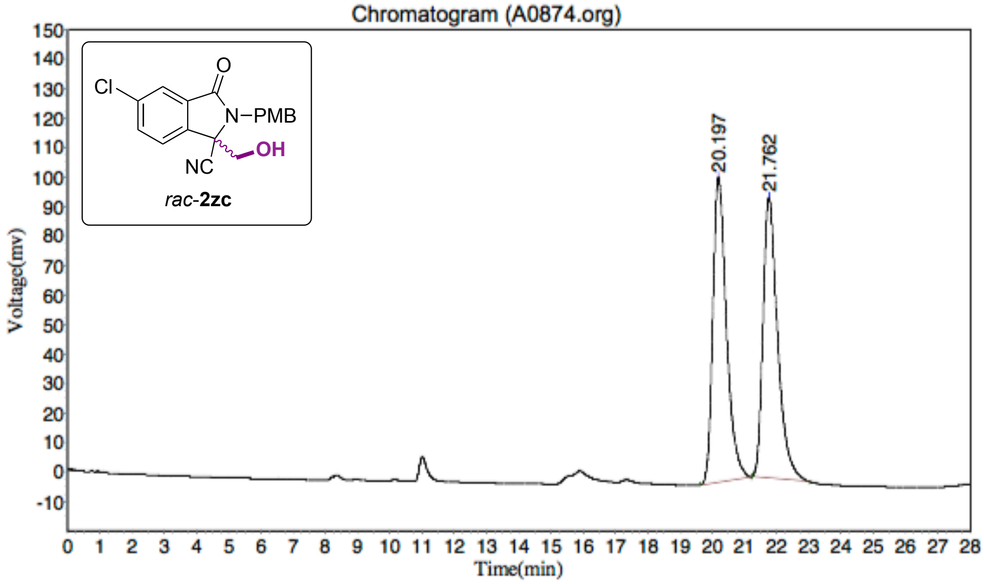

Results

Peak No.	Peak ID	Ret Time	Height	Area	Conc.
1		20.197	103460.375	3017411.000	50.2179
2		21.762	95013.641	2991228.250	49.7821
**Total**			198474.016	6008639.250	100.0000

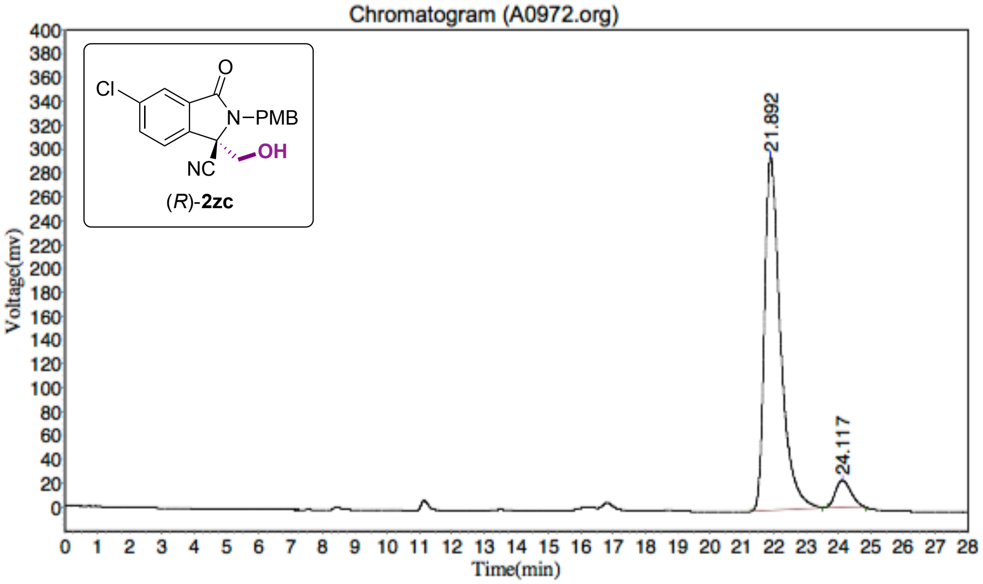

Results

Peak No.	Peak ID	Ret Time	Height	Area	Conc.
1		21.892	296620.594	10194383.000	93.0948
2		24.117	22484.084	756152.750	6.9052
**Total**			319104.678	10950535.750	100.0000

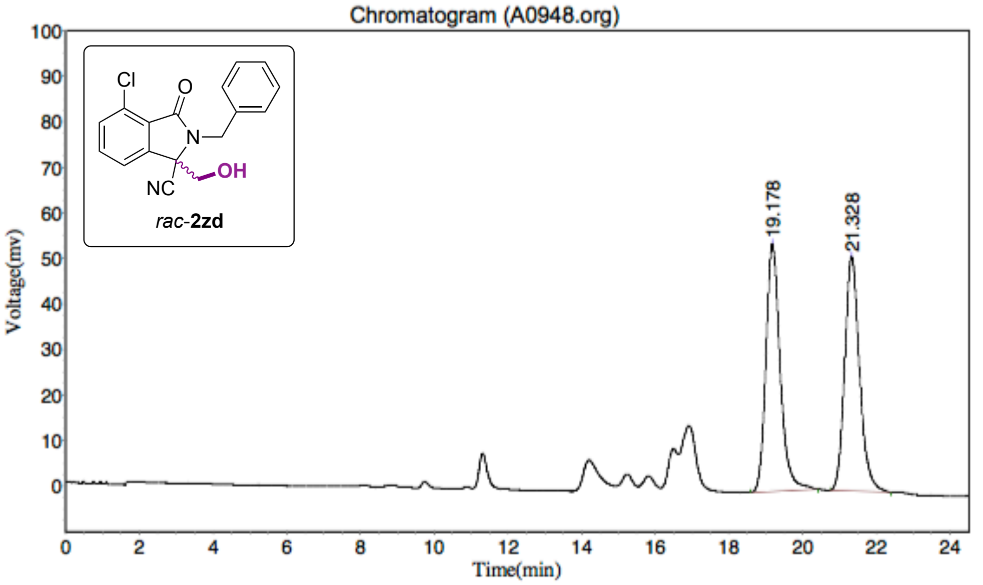

Results

Peak No.	Peak ID	Ret Time	Height	Area	Conc.
1		19.178	54401.152	1462110.000	50.3340
2		21.328	51419.563	1442708.125	49.6660
**Total**			105820.715	2904818.125	100.0000

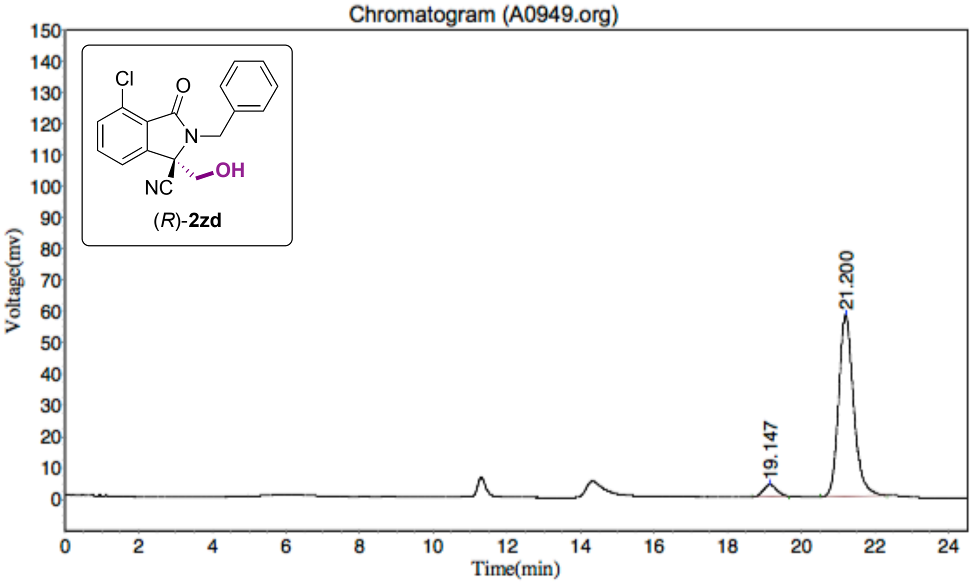

Results

Peak No.	Peak ID	Ret Time	Height	Area	Conc.
1		19.147	3778.066	94407.898	5.4622
2		21.200	57939.664	1633987.875	94.5378
**Total**			61717.730	1728395.773	100.0000

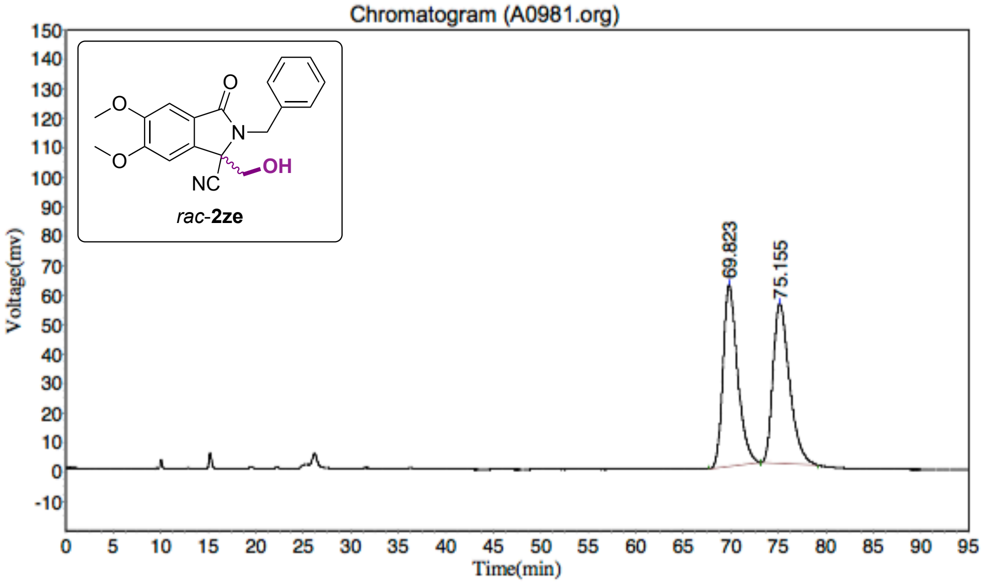

Results

Peak No.	Peak ID	Ret Time	Height	Area	Conc.
1		69.823	61636.063	6571169.000	50.1239
2		75.155	54196.922	6538691.000	49.8761
**Total**			115832.984	13109860.000	100.0000

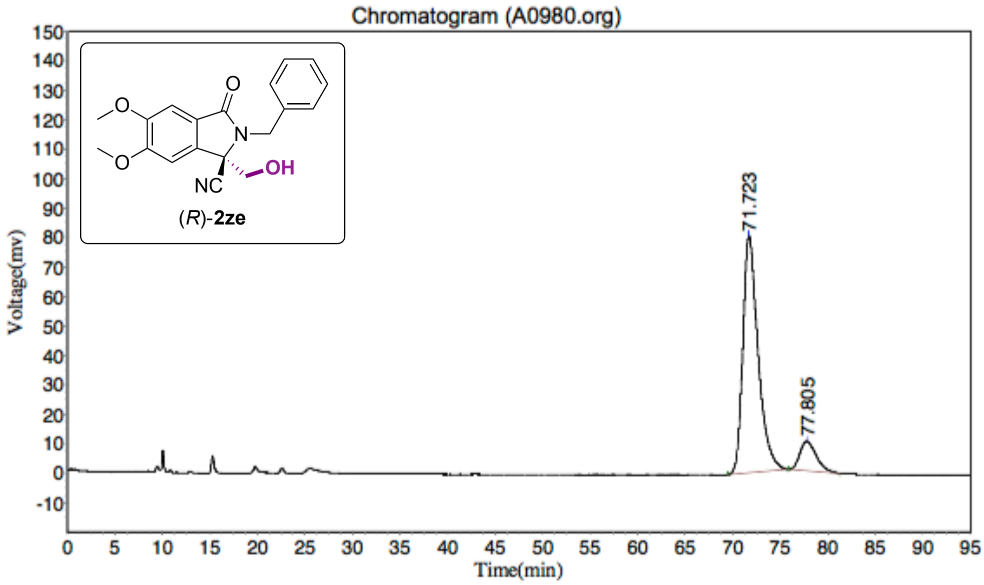

Results

Peak No.	Peak ID	Ret Time	Height	Area	Conc.
1		71.723	80382.227	8928708.000	88.2440
2		77.805	9903.241	1189498.250	11.7560
**Total**			90285.468	10118206.250	100.0000

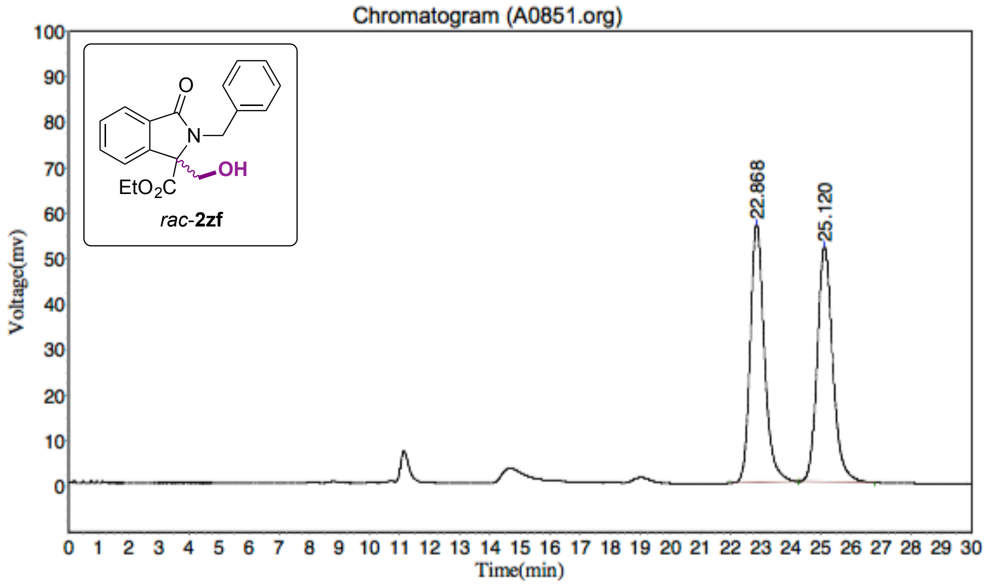

Results

Peak No.	Peak ID	Ret Time	Height	Area	Conc.
1		22.868	56636.582	1838790.000	49.9625
2		25.120	51718.816	1841553.750	50.0375
**Total**			108355.398	3680343.750	100.0000

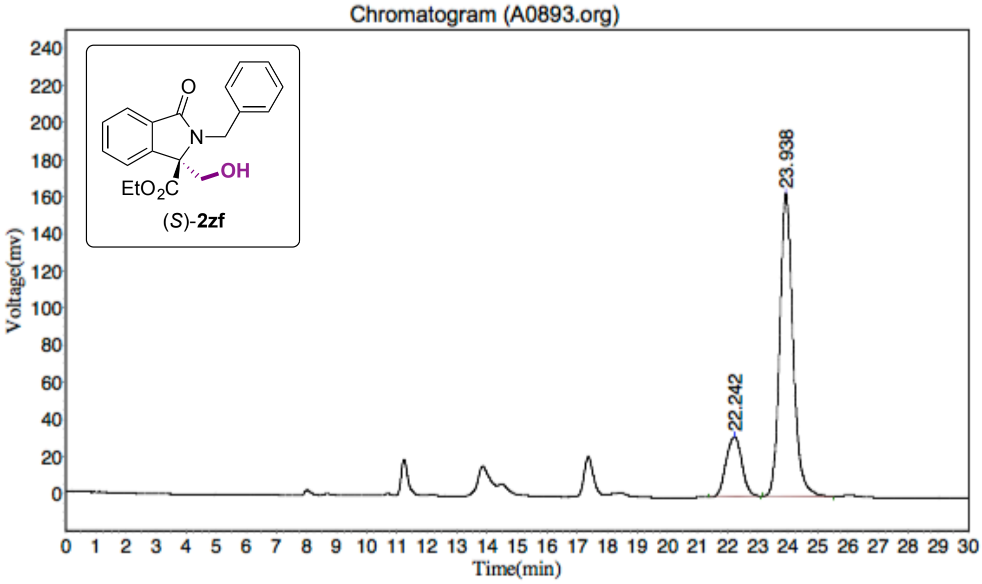

Results

Peak No.	Peak ID	Ret Time	Height	Area	Conc.
1		22.242	32344.689	1183204.000	19.1895
2		23.938	163186.563	4982693.500	80.8105
**Total**			195531.252	6165897.500	100.0000

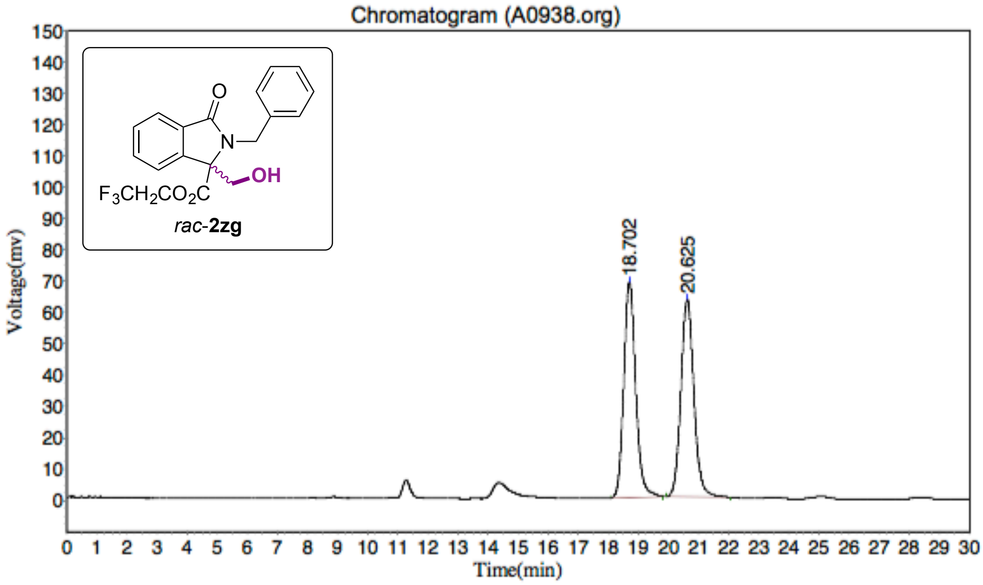

Results

Peak No.	Peak ID	Ret Time	Height	Area	Conc.
1		18.702	68738.484	1880444.250	49.9788
2		20.625	63057.719	1882041.500	50.0212
**Total**			131796.203	3762485.750	100.0000

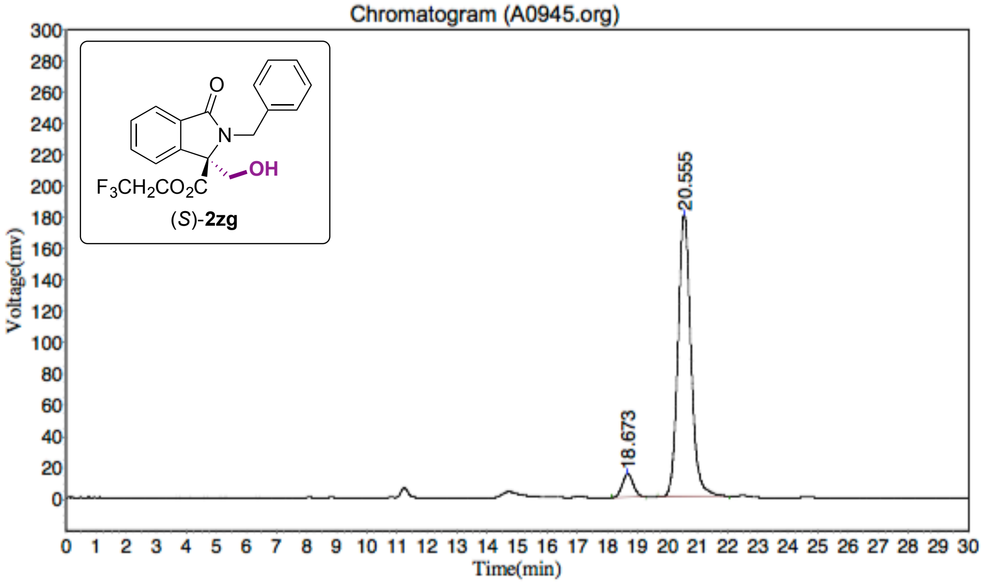

Results

Peak No.	Peak ID	Ret Time	Height	Area	Conc.
1		18.673	14878.756	379996.594	6.6021
2		20.555	179827.938	5375677.000	93.3979
**Total**			194706.693	5755673.594	100.0000

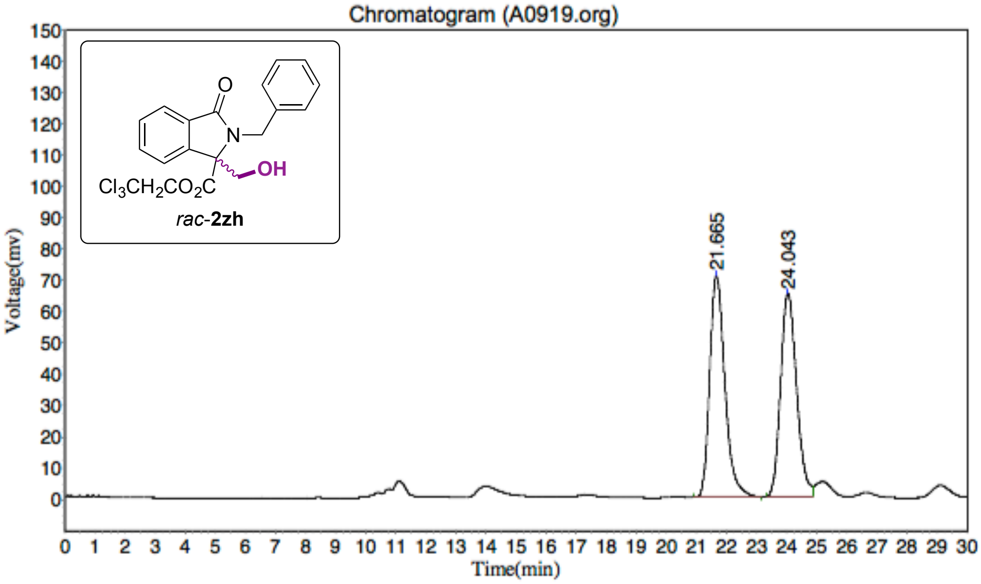

Results

Peak No.	Peak ID	Ret Time	Height	Area	Conc.
1		21.665	70613.820	2472562.000	50.8740
2		24.043	64778.715	2387604.750	49.1260
**Total**			135392.535	4860166.750	100.0000

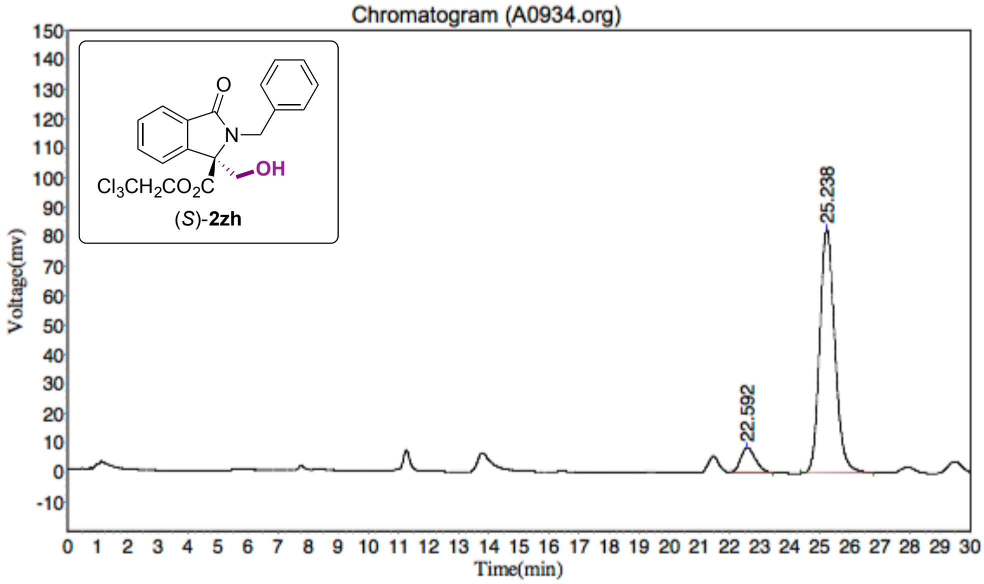

Results

Peak No.	Peak ID	Ret Time	Height	Area	Conc.
1		22.592	8363.431	280109.688	8.8414
2		25.238	82700.992	2888063.500	91.1586
**Total**			91064.423	3168173.188	100.0000

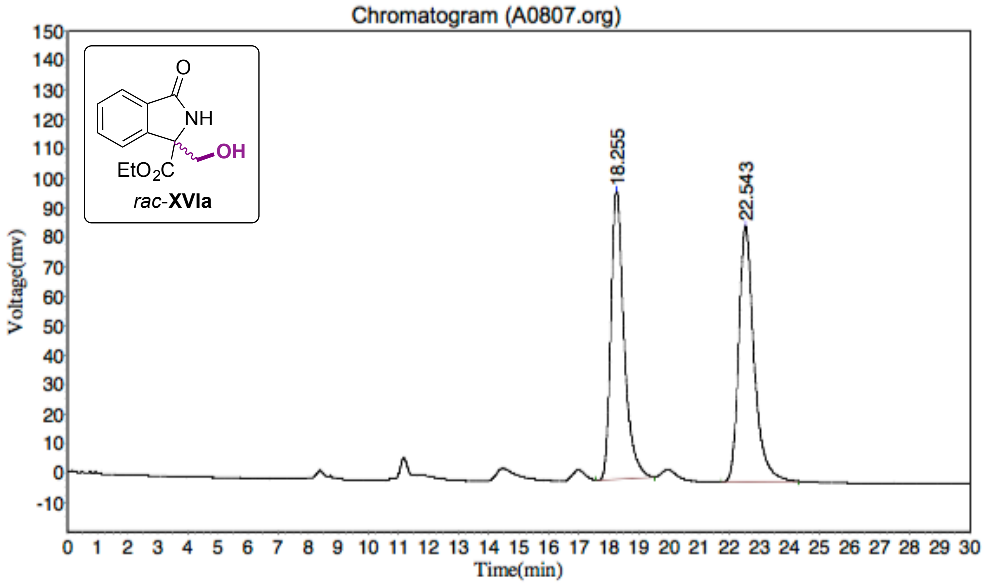

Results

Peak No.	Peak ID	Ret Time	Height	Area	Conc.
1		18.255	97706.977	2959670.250	49.0990
2		22.543	86912.320	3068290.000	50.9010
**Total**			184619.297	6027960.250	100.0000

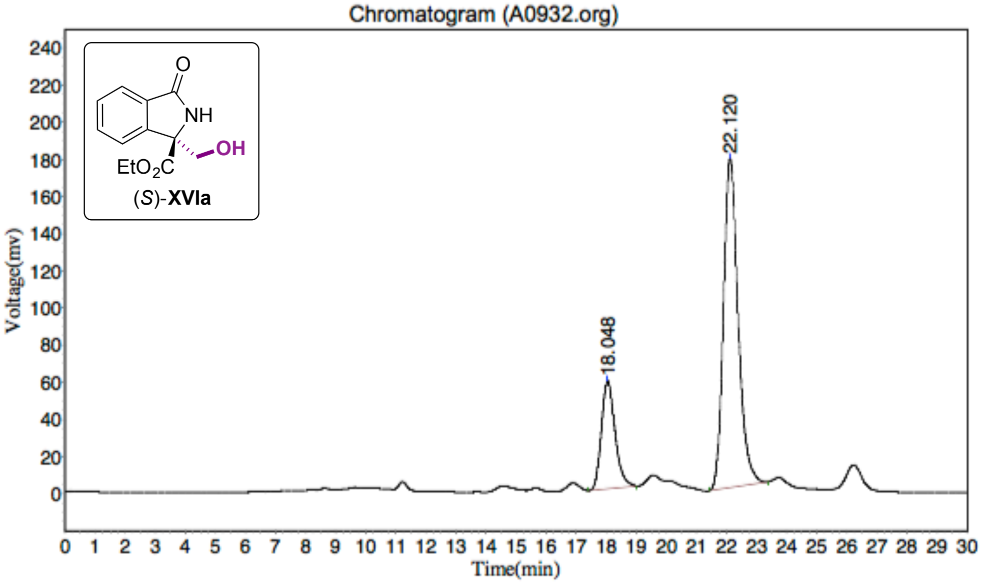

Results

Peak No.	Peak ID	Ret Time	Height	Area	Conc.
1		18.048	57941.691	1825563.250	23.3210
2		22.120	177268.172	6002419.000	76.6790
**Total**			235209.863	7827982.250	100.0000

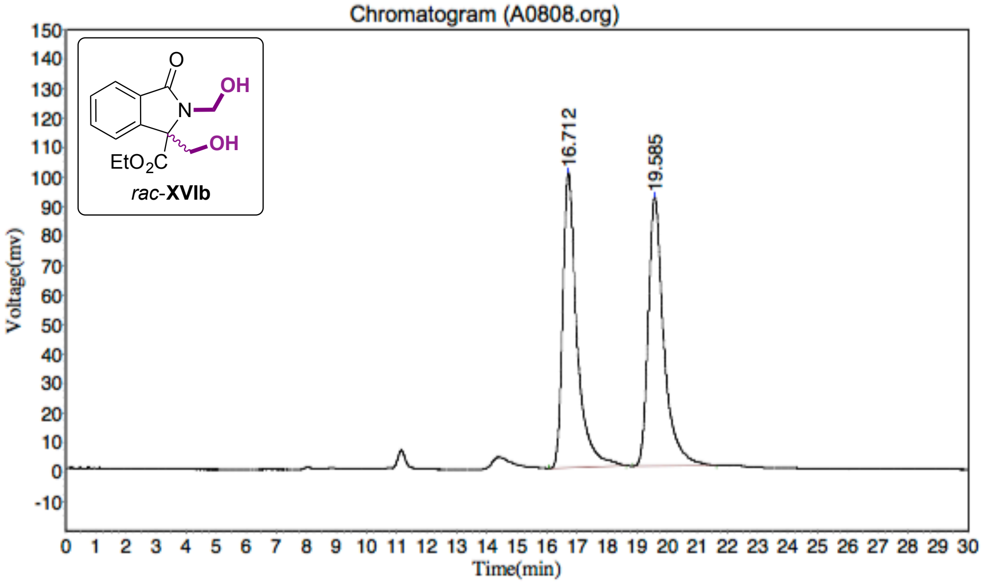

Results

Peak No.	Peak ID	Ret Time	Height	Area	Conc.
1		16.712	100038.734	3281676.500	50.0584
2		19.585	91131.867	3274014.000	49.9416
**Total**			191170.602	6555690.500	100.0000

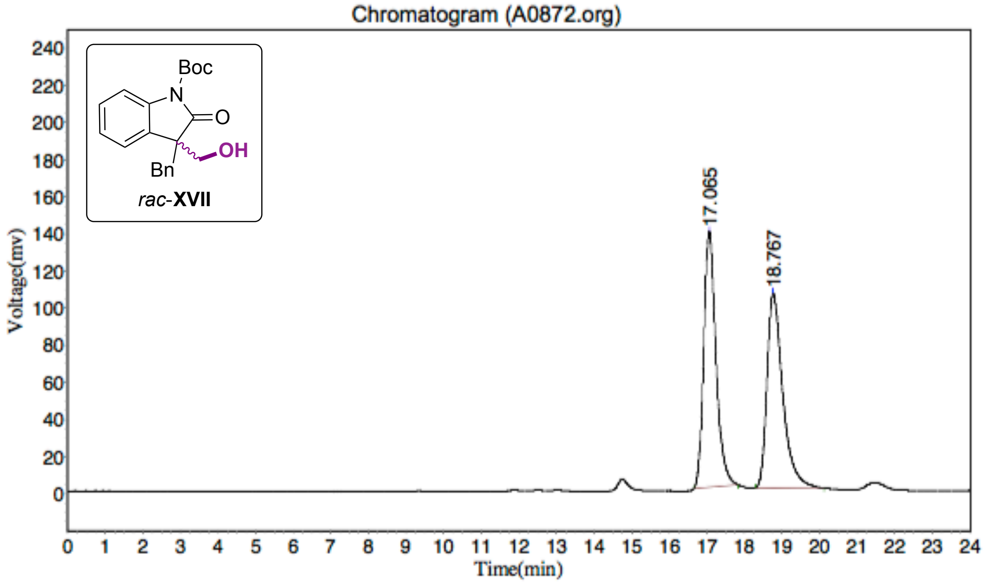

Results

Peak No.	Peak ID	Ret Time	Height	Area	Conc.
1		17.065	137943.453	3111545.500	50.5339
2		18.767	104982.211	3045795.000	49.4661
**Total**			242925.664	6157340.500	100.0000

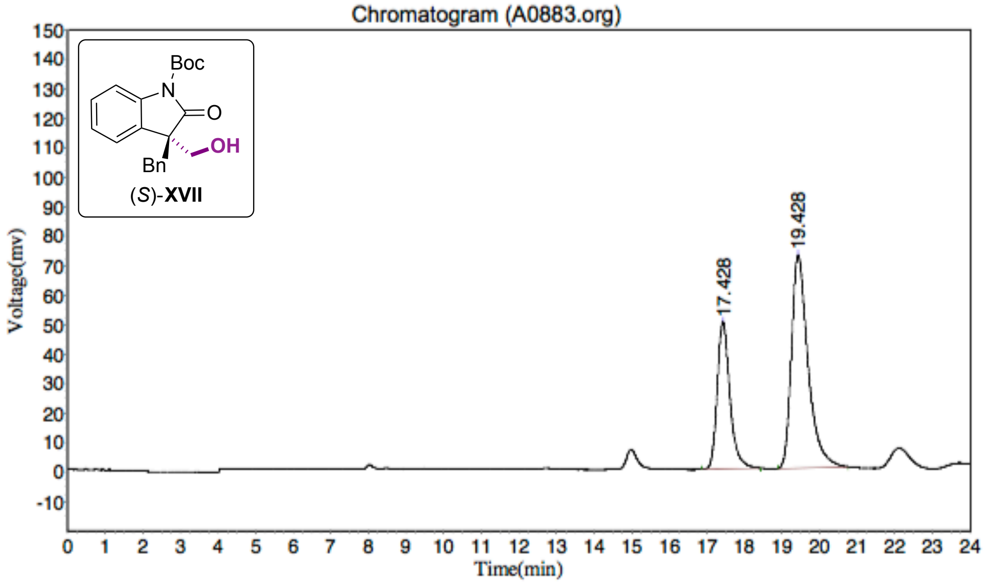

Results

Peak No.	Peak ID	Ret Time	Height	Area	Conc.
1		17.428	49906.859	1163338.625	34.3611
2		19.428	72248.297	2222291.500	65.6389
**Total**			122155.156	3385630.125	100.0000

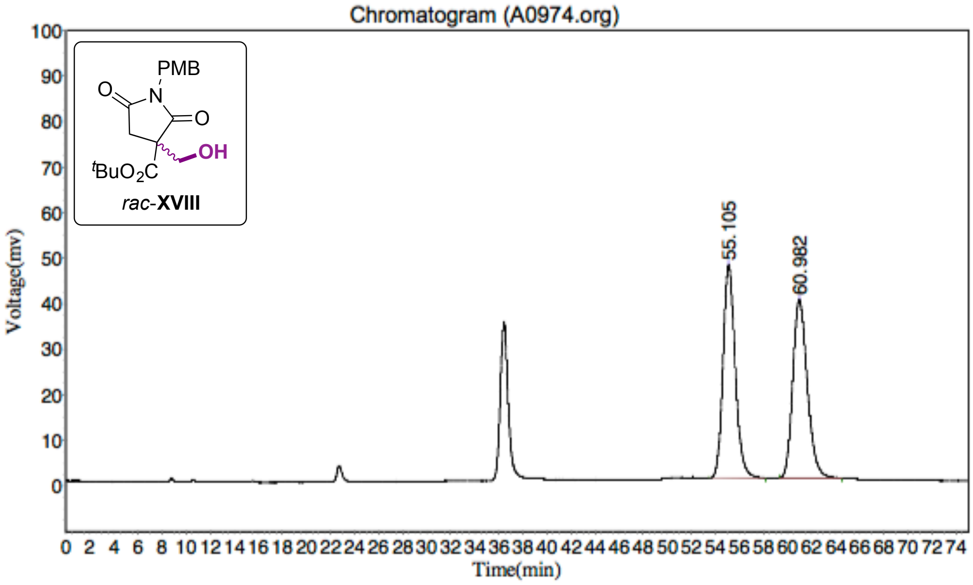

Results

Peak No.	Peak ID	Ret Time	Height	Area	Conc.
1		55.105	46873.469	3321535.000	49.9412
2		60.982	39237.164	3329353.750	50.0588
**Total**			86110.633	6650888.750	100.0000

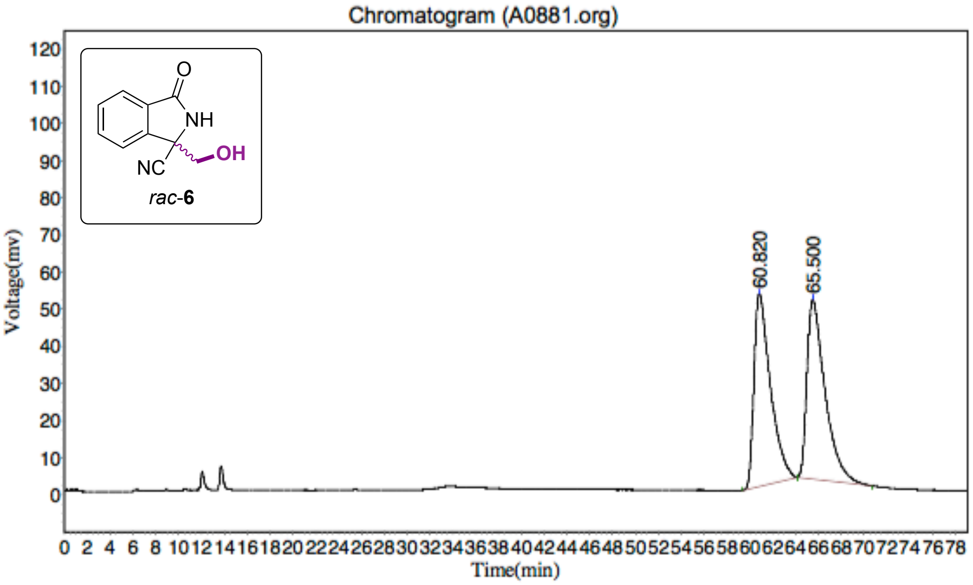

Results

Peak No.	Peak ID	Ret Time	Height	Area	Conc.
1		60.820	51883.340	5203111.000	49.4995
2		65.500	48293.289	5308338.500	50.5005
**Total**			100176.629	10511449.500	100.0000

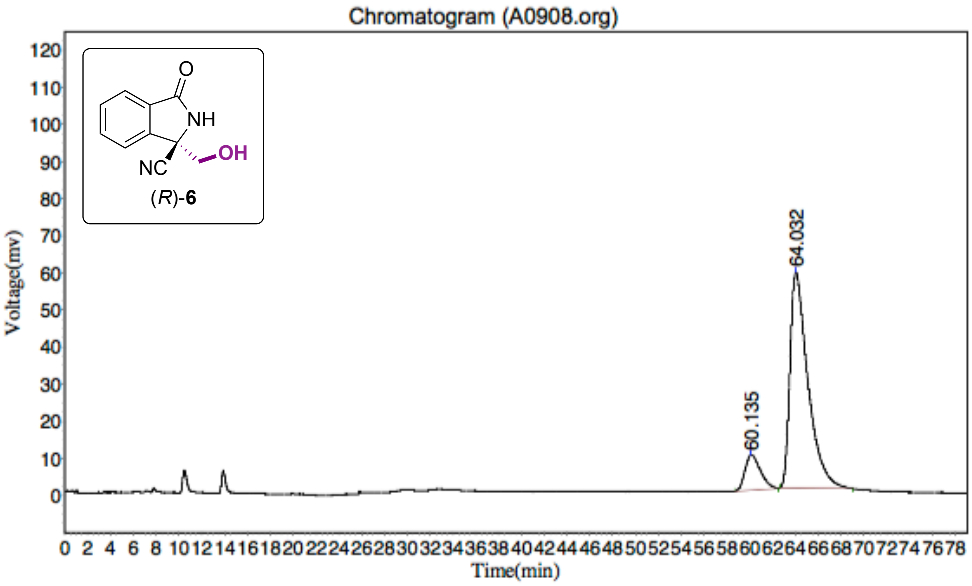

Results

Peak No.	Peak ID	Ret Time	Height	Area	Conc.
1		60.135	9483.971	888578.813	12.2618
2		64.032	58098.715	6358166.500	87.7382
**Total**			67582.686	7246745.313	100.0000

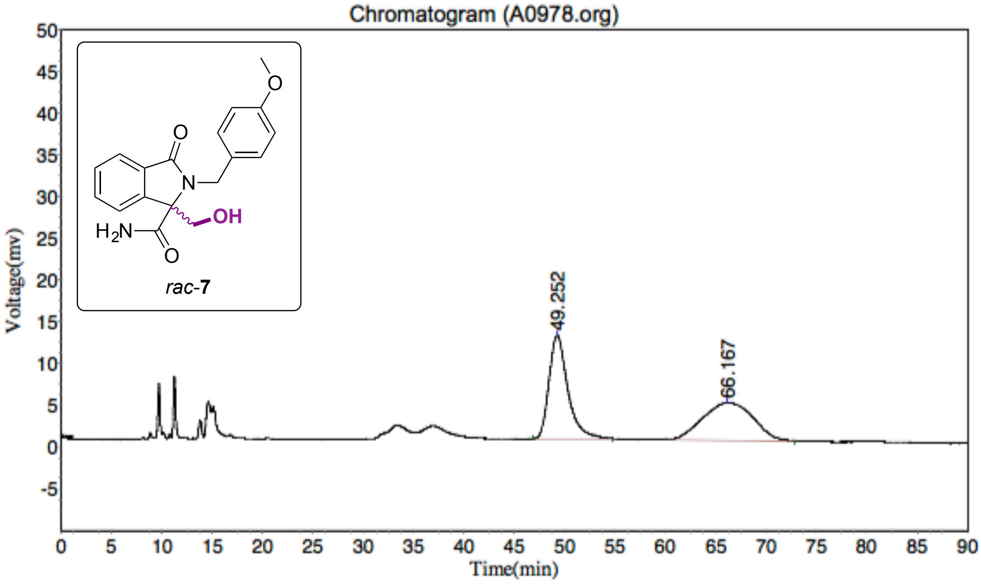

Results

Peak No.	Peak ID	Ret Time	Height	Area	Conc.
1		49.252	12360.783	1605799.375	50.7383
2		66.167	4511.298	1559066.875	49.2617
**Total**			16872.081	3164866.250	100.0000

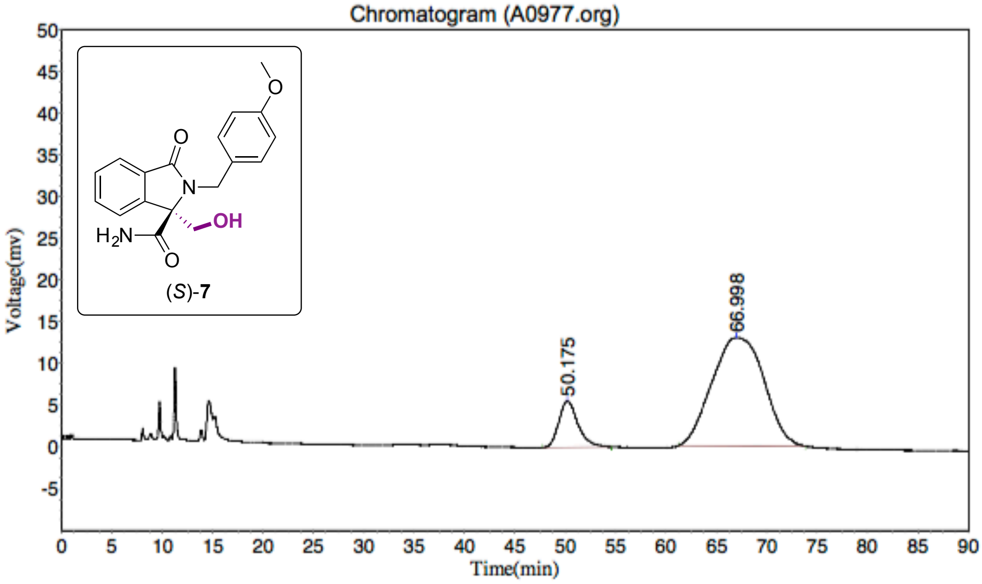

Results

Peak No.	Peak ID	Ret Time	Height	Area	Conc.
1		50.175	5561.641	730580.625	13.5662
2		66.998	12978.434	4654710.500	86.4338
**Total**			18540.074	5385291.125	100.0000

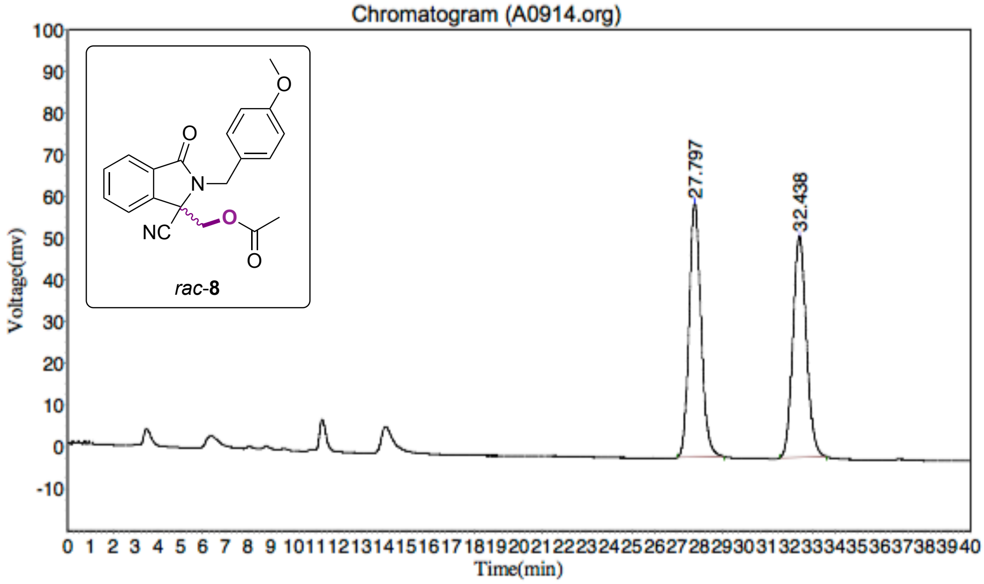

Results

Peak No.	Peak ID	Ret Time	Height	Area	Conc.
1		27.797	60760.723	2226950.500	50.2728
2		32.438	52897.500	2202783.500	49.7272
**Total**			113658.223	4429734.000	100.0000

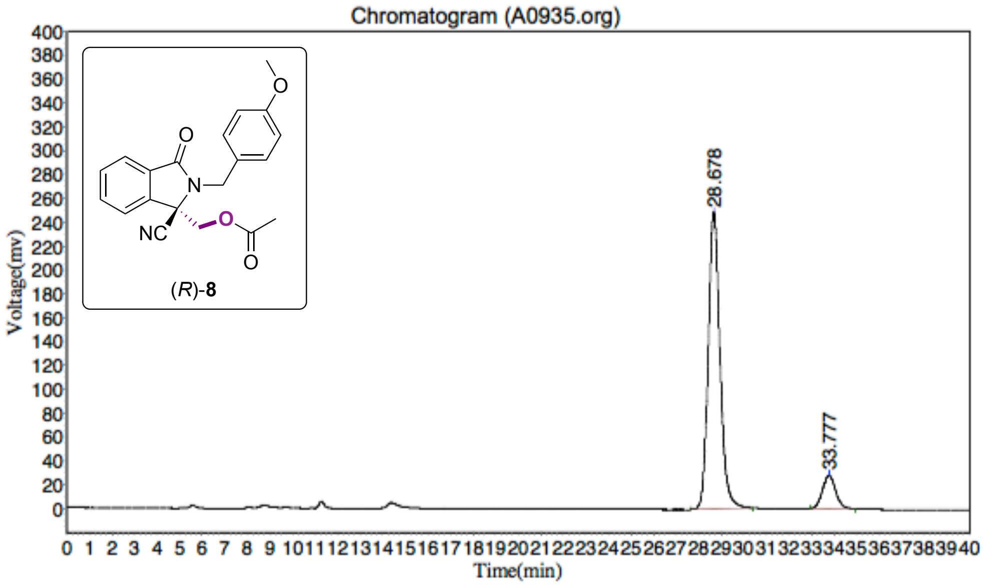

Results

Peak No.	Peak ID	Ret Time	Height	Area	Conc.
1		28.678	248498.250	8990189.000	88.7177
2		33.777	27971.727	1143287.875	11.2823
**Total**			276469.977	10133476.875	100.0000

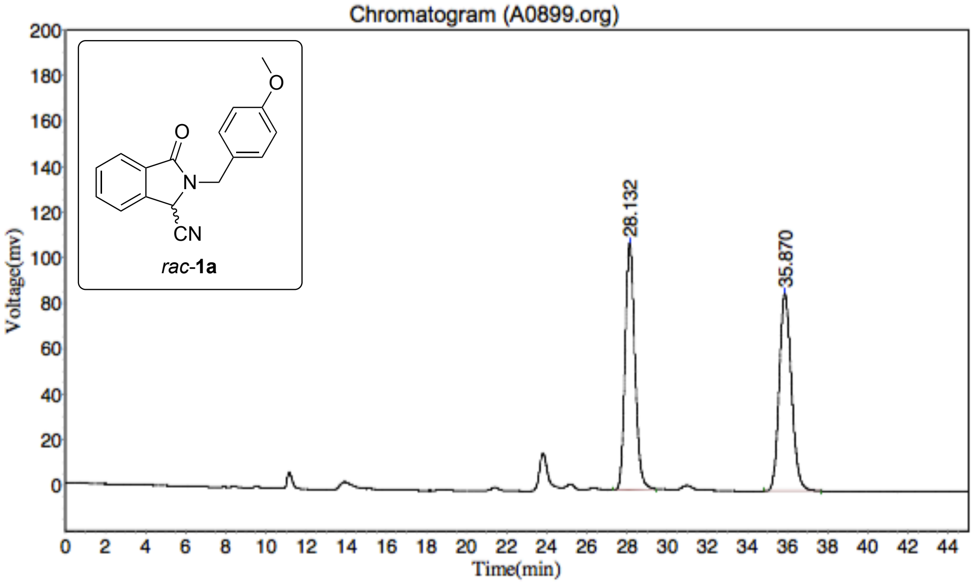

Results

Peak No.	Peak ID	Ret Time	Height	Area	Conc.
1		28.132	108374.633	3652556.250	49.7847
2		35.870	86844.281	3684152.750	50.2153
**Total**			195218.914	7336709.000	100.0000

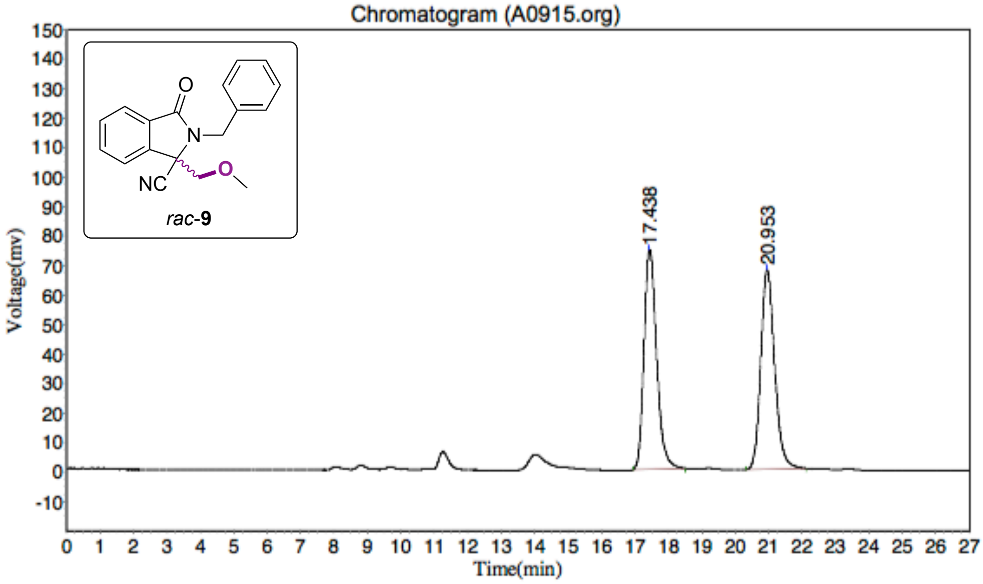

Results

Peak No.	Peak ID	Ret Time	Height	Area	Conc.
1		17.438	74526.141	1944268.250	49.8992
2		20.953	67660.078	1952119.875	50.1008
**Total**			142186.219	3896388.125	100.0000

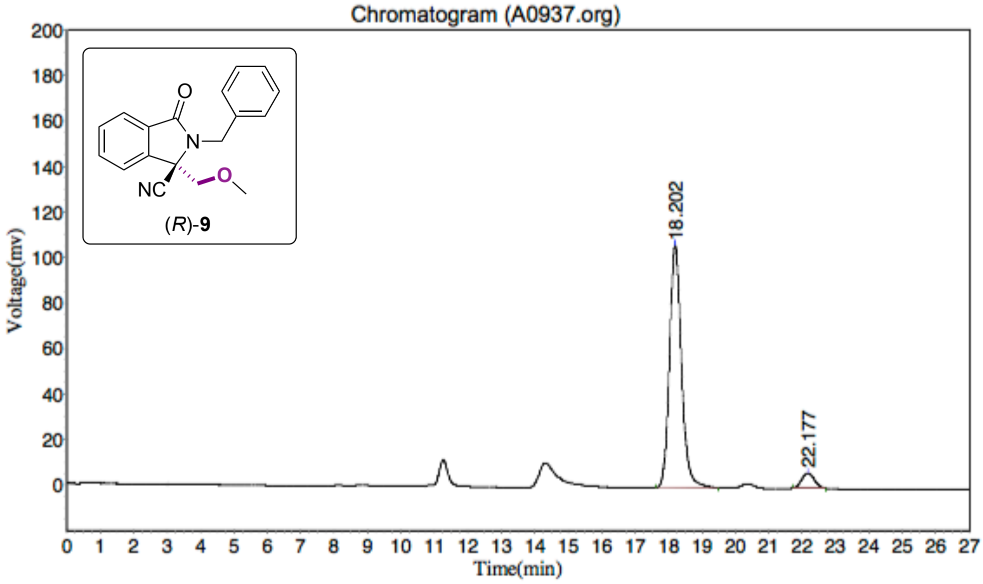

Results

Peak No.	Peak ID	Ret Time	Height	Area	Conc.
1		18.202	106624.125	2678352.250	94.0411
2		22.177	6560.387	169712.750	5.9589
**Total**			113184.512	2848065.000	100.0000

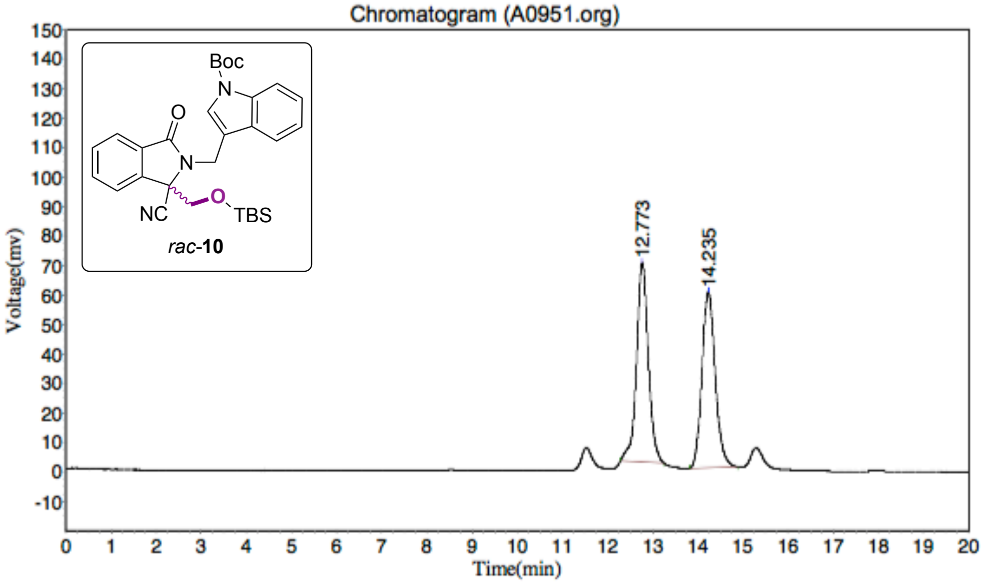

Results

Peak No.	Peak ID	Ret Time	Height	Area	Conc.
1		12.773	67541.836	1233049.875	50.1119
2		14.235	59688.828	1227544.125	49.8881
**Total**			127230.664	2460594.000	100.0000

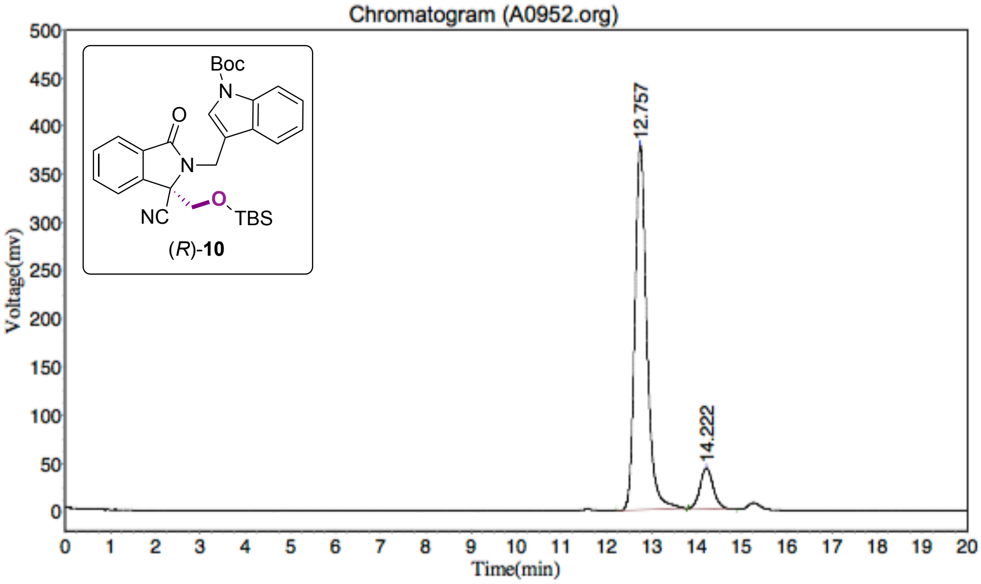

Results

Peak No.	Peak ID	Ret Time	Height	Area	Conc.
1		12.757	378181.656	6967093.000	89.0574
2		14.222	42109.555	856057.063	10.9426
**Total**			420291.211	7823150.063	100.0000

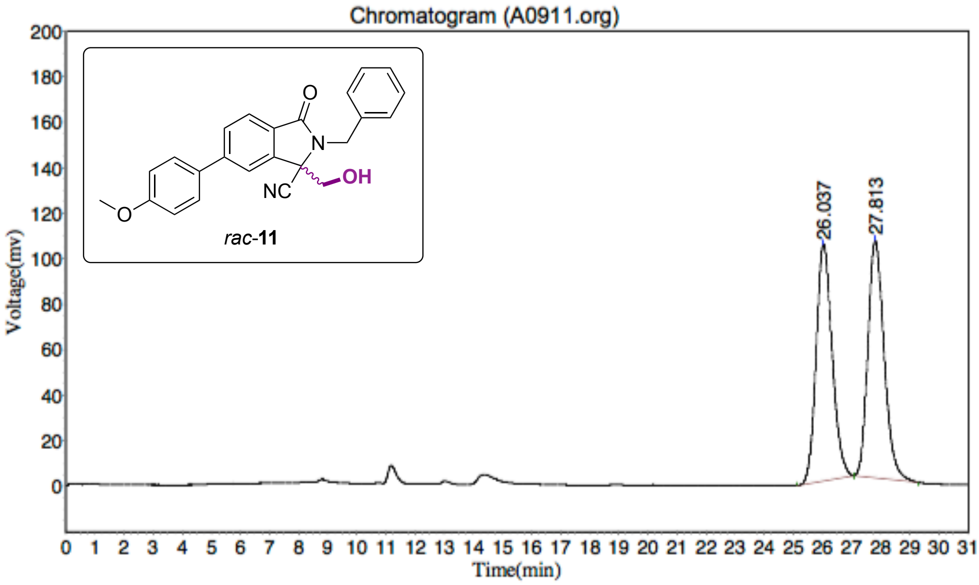

Results

Peak No.	Peak ID	Ret Time	Height	Area	Conc.
1		26.037	103896.555	3966576.000	49.5860
2		27.813	104236.844	4032817.250	50.4140
**Total**			208133.398	7999393.250	100.0000

10. Copies of the Product MS Spectra

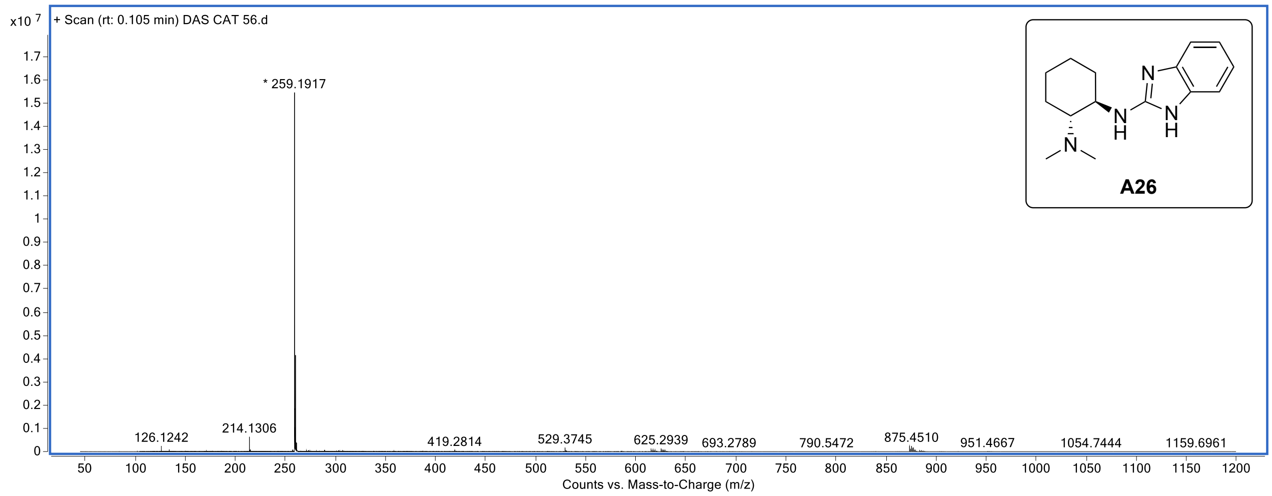

Compound **A26** HRMS (ESI-Orbitrap) *m/z*: $[M + H]^+$ calcd for $C_{15}H_{23}N_4$ 259.1917; found 259.1917.

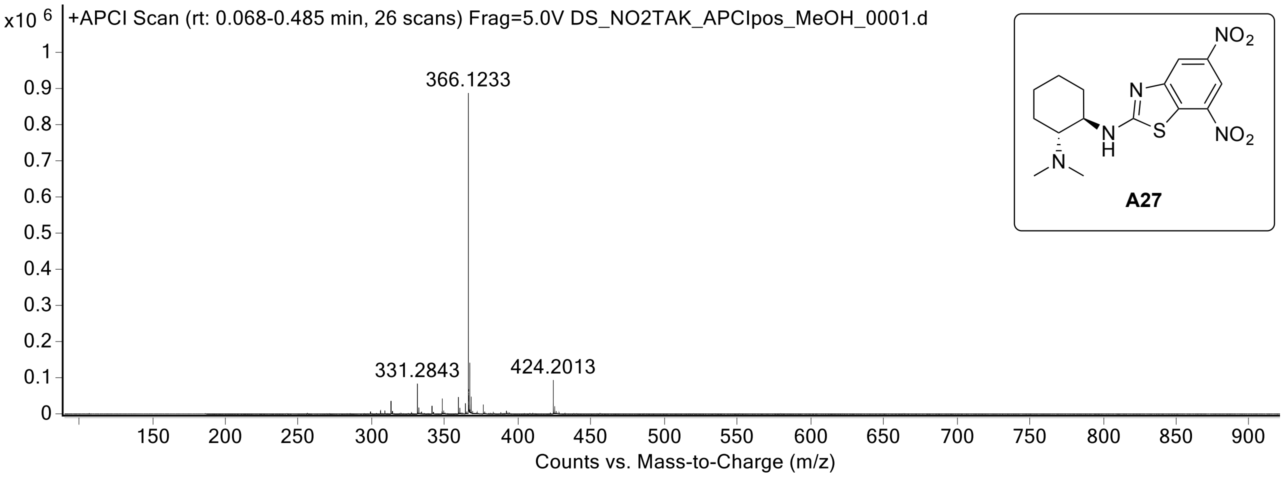

Compound **A27** HRMS (APCI) *m/z*: $[M + H]^+$ calcd for $C_{15}H_{20}N_5O_4S$ 366.1231, found 366.1233.

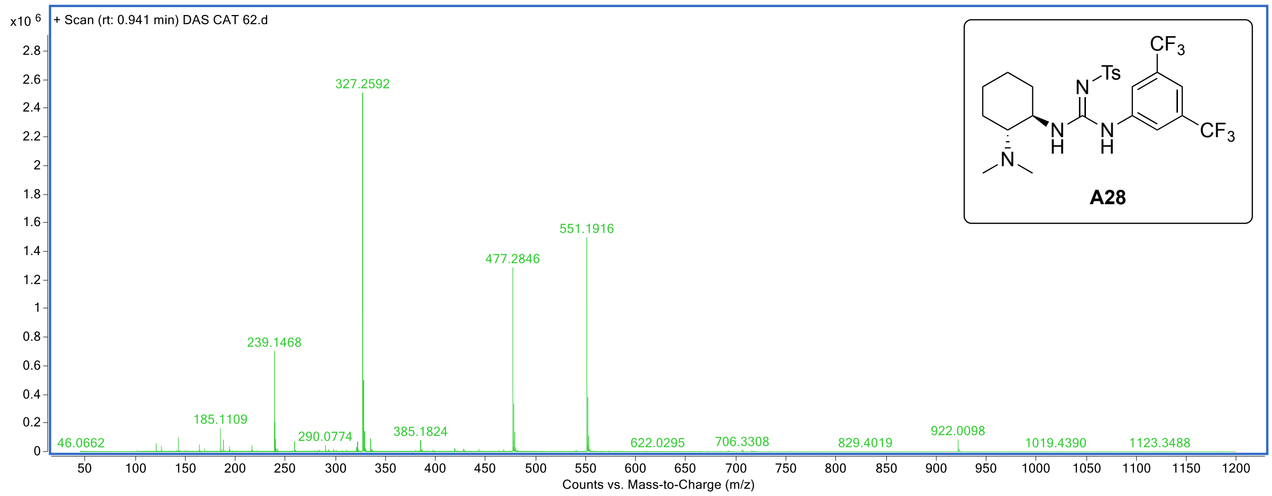

Compound **A28** HRMS (ESI-Orbitrap) *m/z*: $[M + H]^+$ calcd for $C_{24}H_{29}F_6N_4O_2S$ 551.1910, found 551.1916.

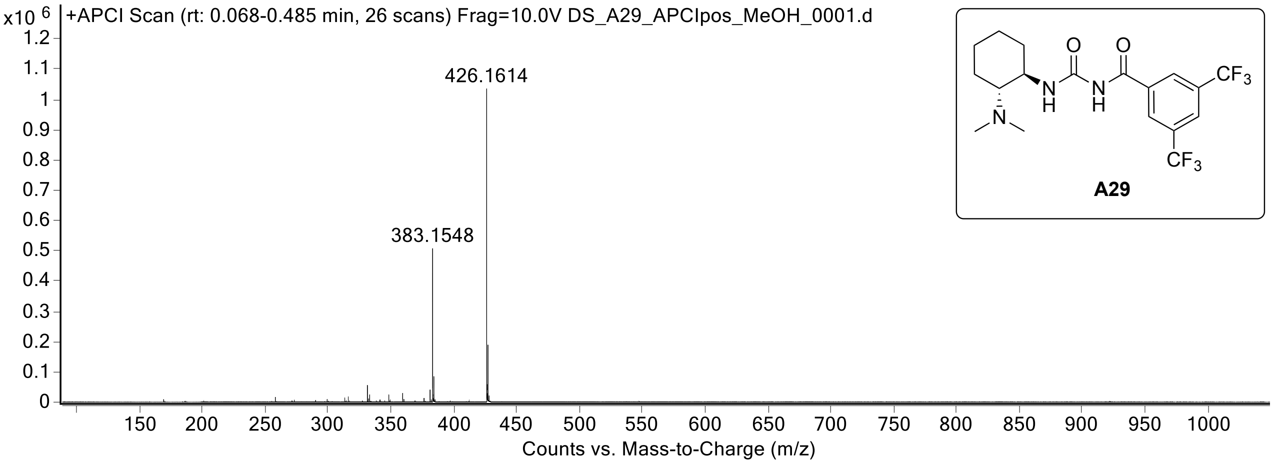

Compound **A29** HRMS (APCI) *m/z*: $[M + H]^+$ calcd for $C_{18}H_{22}F_6N_3O_2$ 426.1611, found 426.1614.

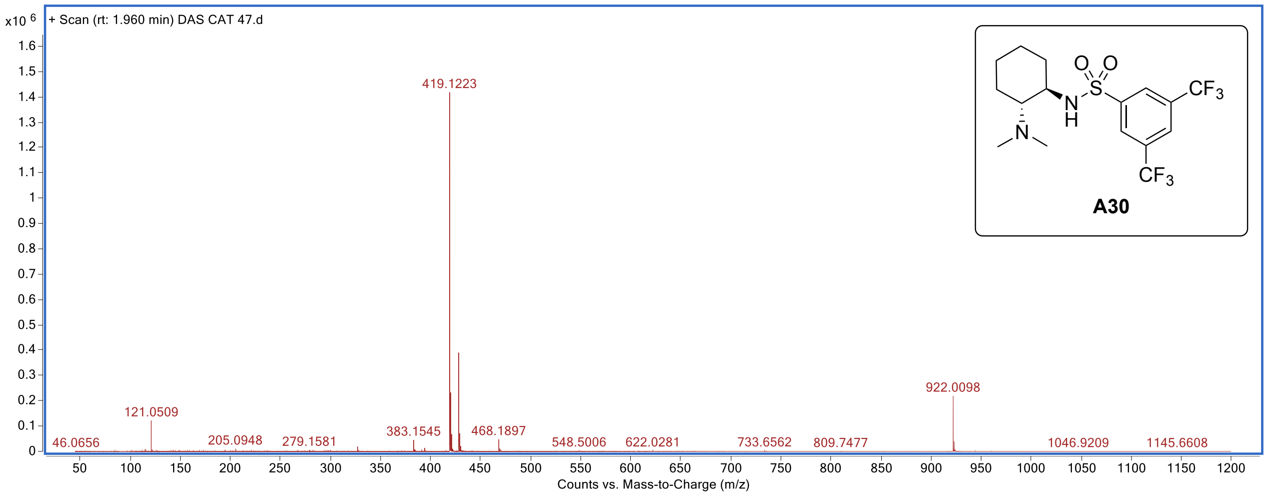

Compound **A30** HRMS (ESI-Orbitrap) *m/z*: $[M + H]^+$ calcd for $C_{16}H_{21}F_6N_2O_2S$ 419.1222, found 419.1223.

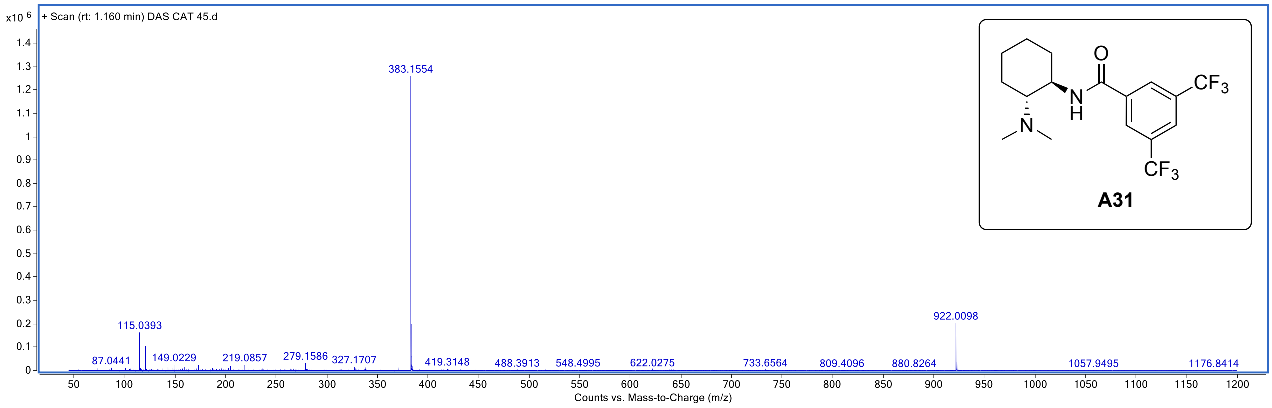

Compound **A31** HRMS (ESI-Orbitrap) *m/z*: $[M + H]^+$ calcd for $C_{17}H_{21}F_6N_2O$ 383.1553, found 383.1554.

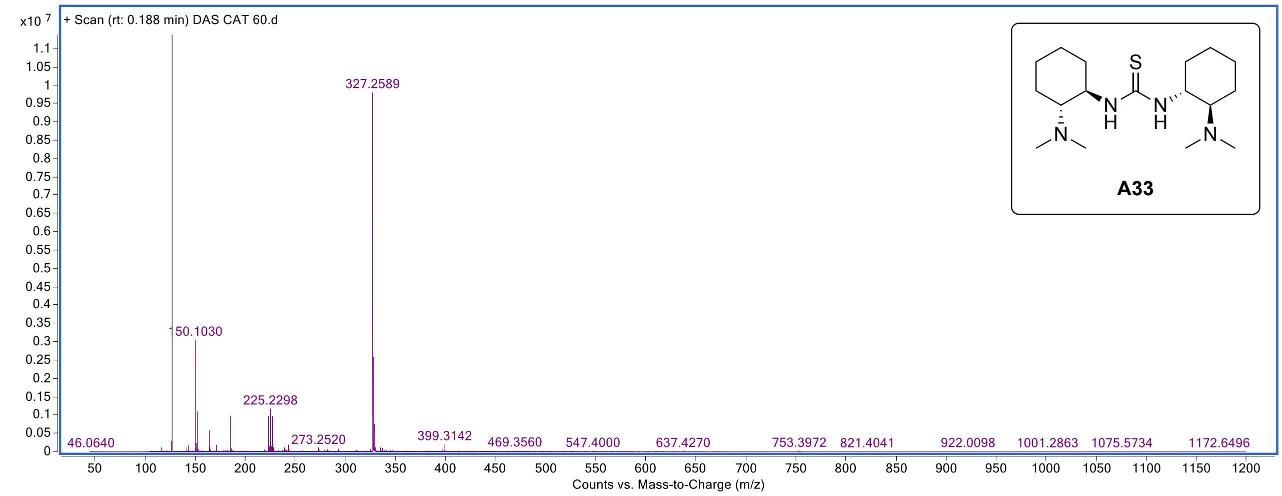

Compound **A33** HRMS (ESI-Orbitrap) *m/z*: $[M + H]^+$ calcd for $C_{17}H_{35}N_4S$ 327.2577, found 327.2589.

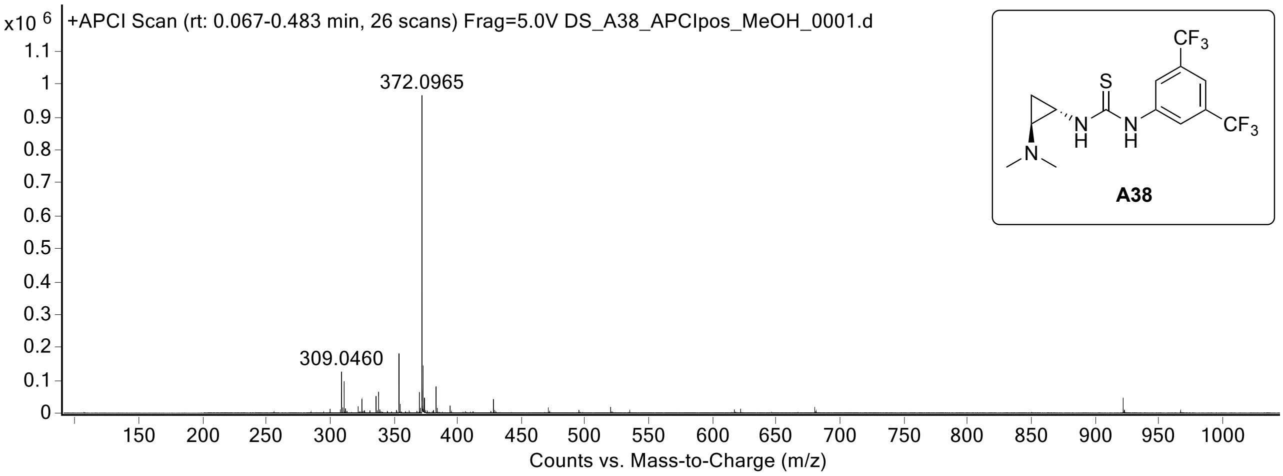

Compound **A38** HRMS (APCI) *m/z*: $[M + H]^+$ calcd for $C_{14}H_{16}F_6N_3S$ 372.0964, found 372.0965.

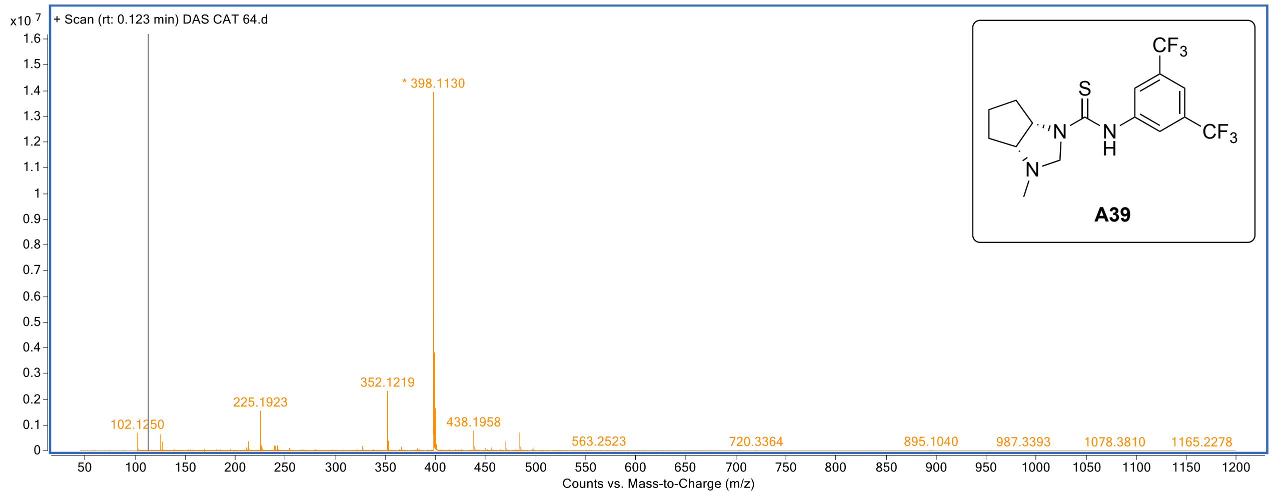

Compound **A39** HRMS (ESI-Orbitrap) *m/z*: $[M + H]^+$ calcd for $C_{16}H_{18}F_6N_3S$ 398.1120, found 398.1130.

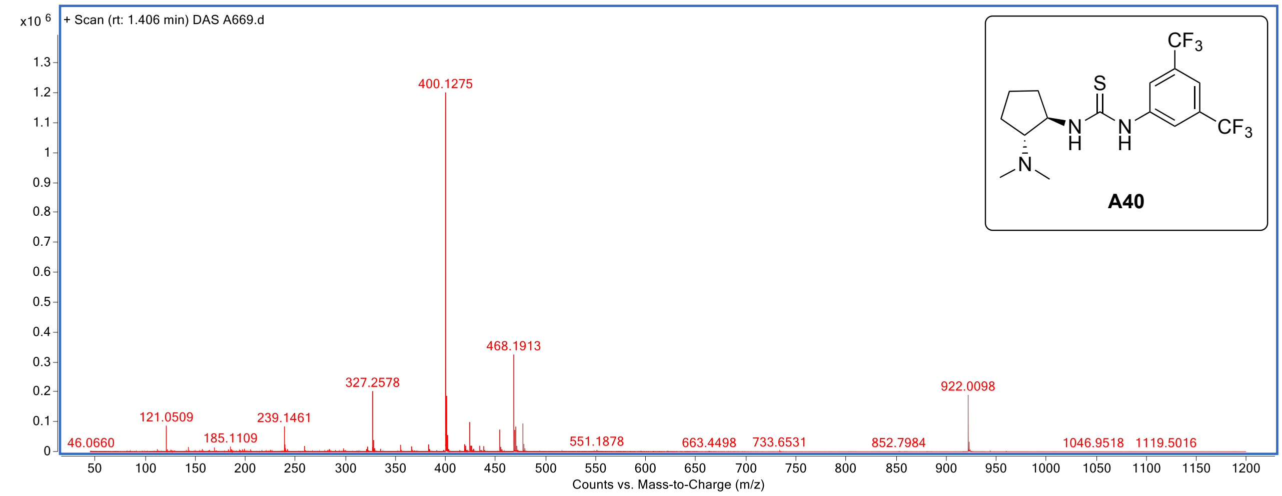

Compound **A40** HRMS (ESI-Orbitrap) *m/z*: $[M + H]^+$ calcd for $C_{16}H_{19}F_6N_3S$ 400.1277, found 400.1275.

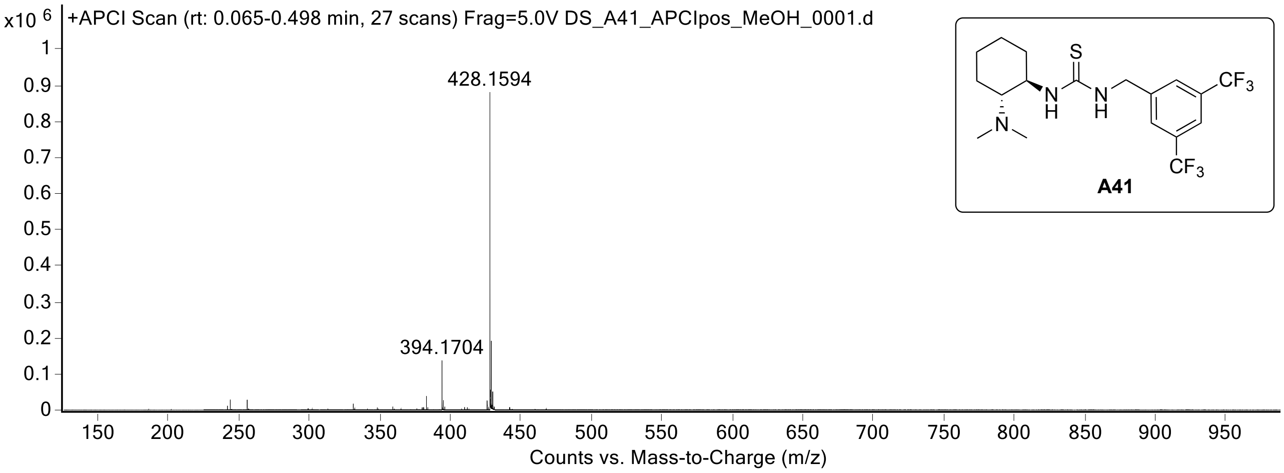

Compound **A41** HRMS (APCI) *m/z*: $[M + H]^+$ calcd for $C_{18}H_{24}F_6N_3S$ 428.1590, found 428.1594.

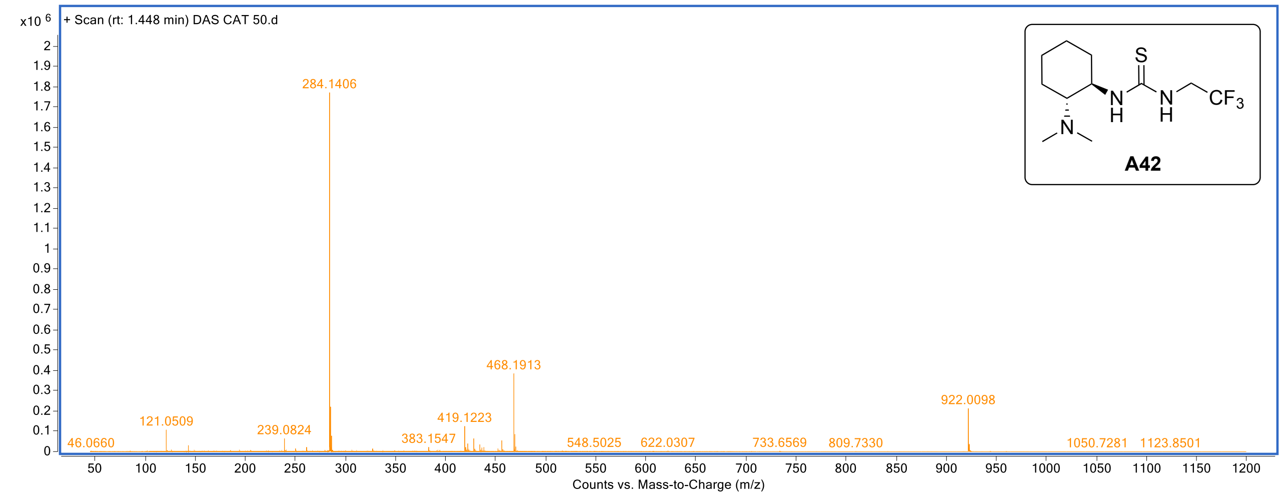

Compound **A42** HRMS (ESI-Orbitrap) *m/z*: $[M + H]^+$ calcd for $C_{11}H_{21}F_3N_3S$ 284.1403, found 284.1406.

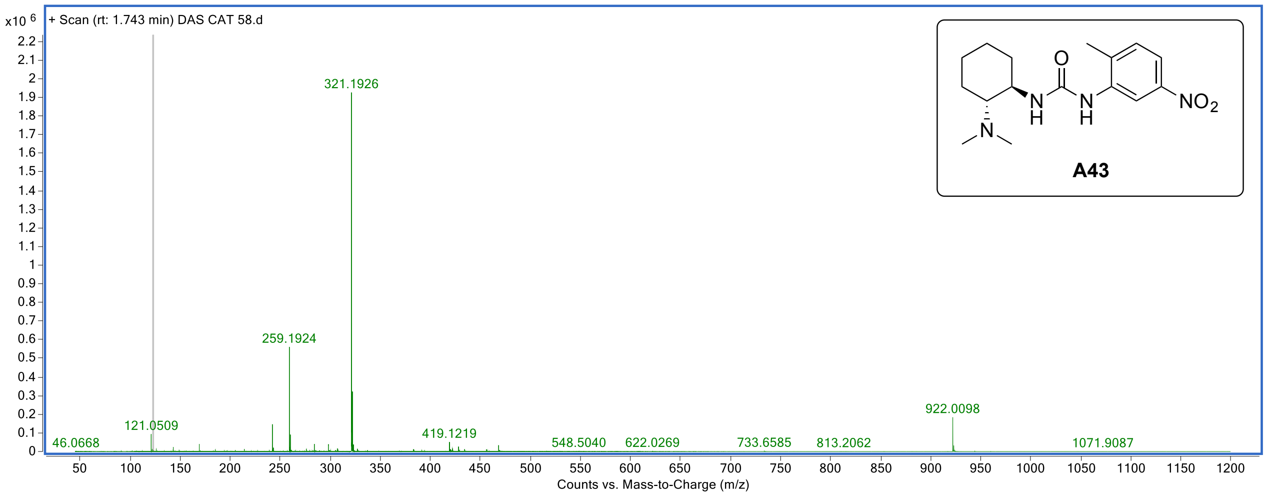

Compound **A43** HRMS (ESI-Orbitrap) *m/z*: $[M + H]^+$ calcd for $C_{16}H_{25}N_4O_3$ 321.1921, found 321.1926.

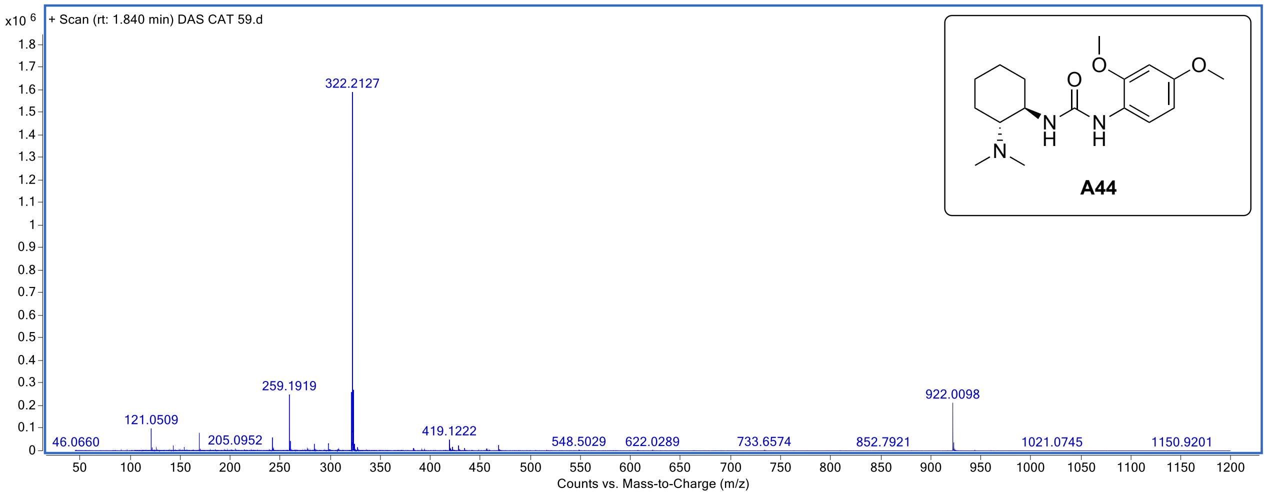

Compound **A44** HRMS (ESI-Orbitrap) *m/z*: $[M + H]^+$ calcd for $C_{17}H_{28}N_3O_3$ 322.2125, found 322.2127.

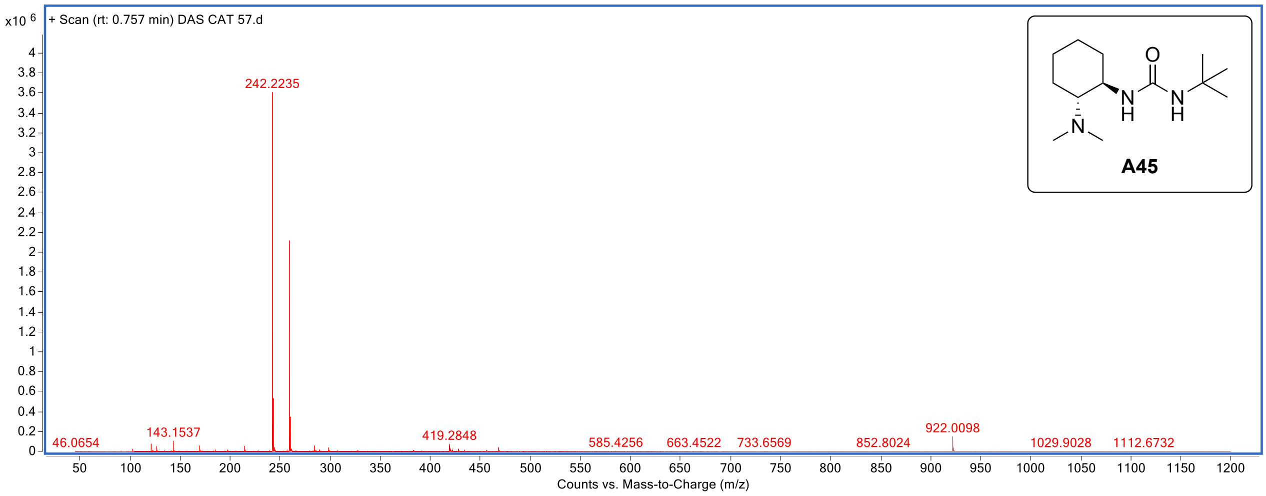

Compound **A45** HRMS (ESI-Orbitrap) *m/z*: $[M + H]^+$ calcd for $C_{13}H_{28}N_3O$ 242.2227, found 242.2235.

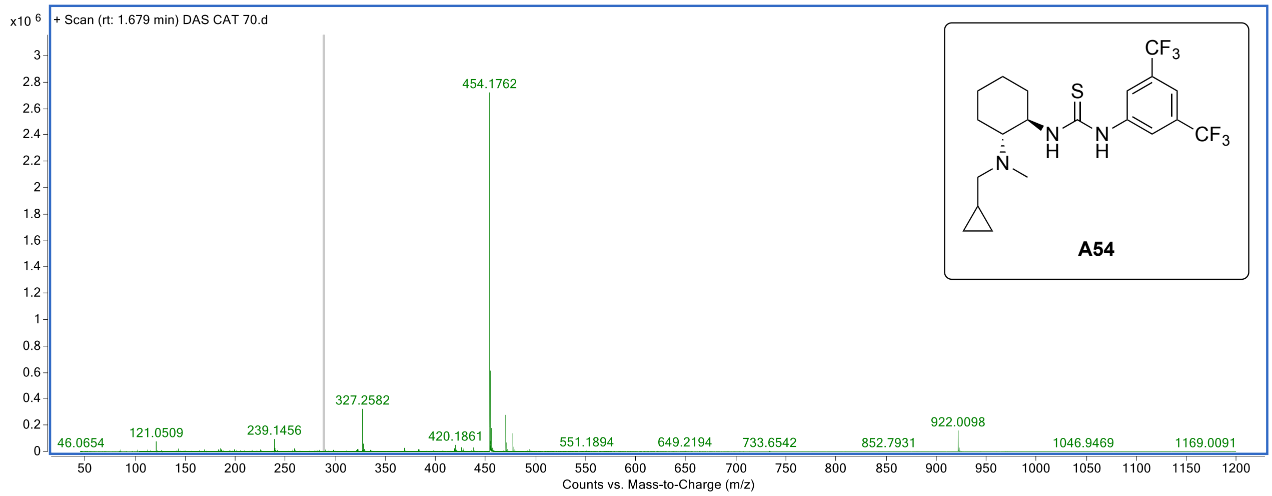

Compound **A54** HRMS (ESI-Orbitrap) *m/z*: $[M + H]^+$ calcd for $C_{20}H_{26}F_6N_3S$ 454.1746, found 454.1762.

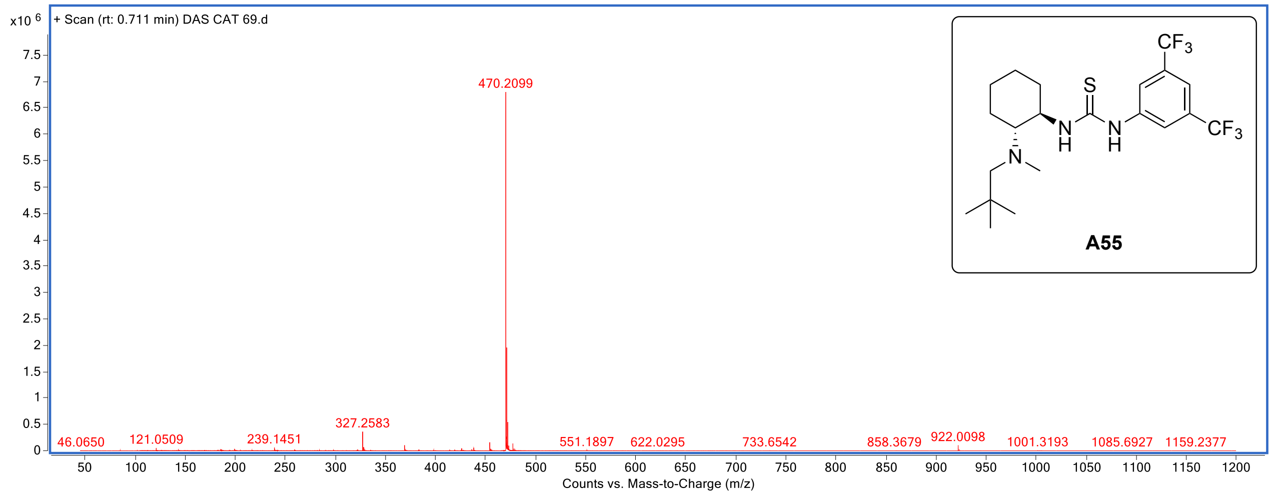

Compound **A55** HRMS (ESI-Orbitrap) *m/z*: $[M + H]^+$ calcd for $C_{21}H_{30}F_6N_3S$ 470.2059, found 470.2099.

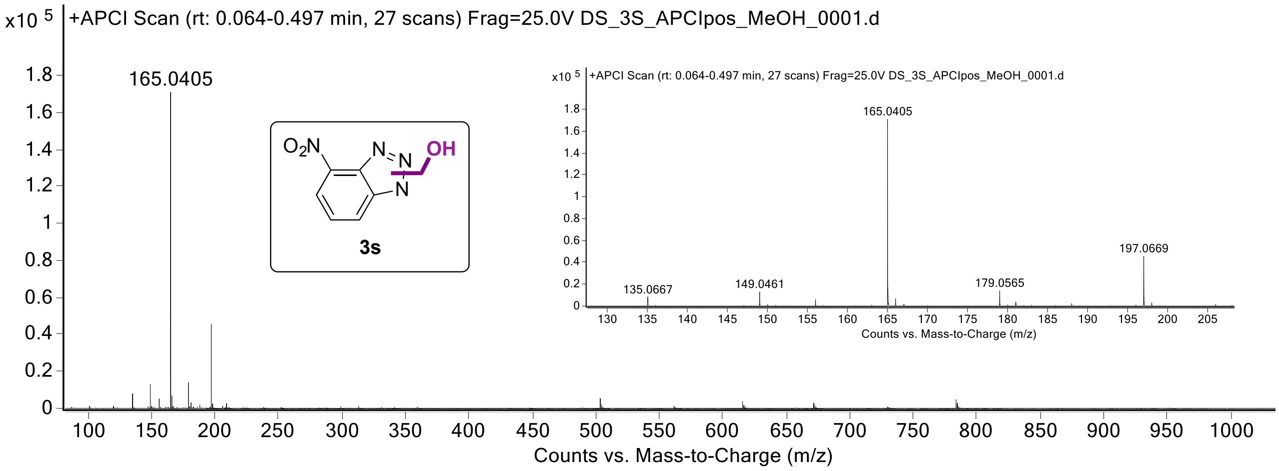

Compound **3s** HRMS (APCI) *m/z*: $[M – CH_2O + H]^+$ calcd for $C_6H_5N_4O_2$ 165.0407, found 165.0405.

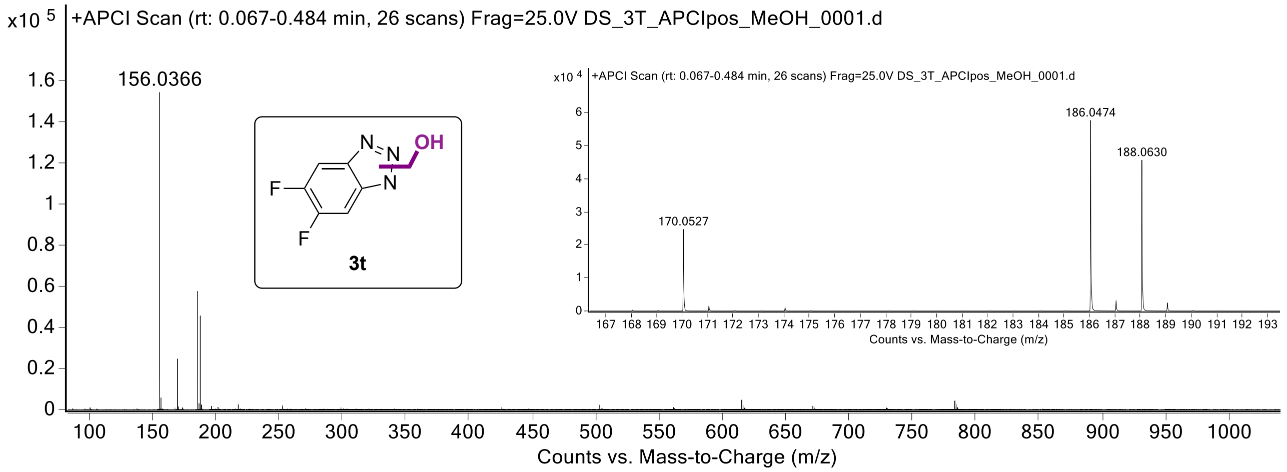

Compound **3t** HRMS (APCI) *m/z*: $[M + H]^+$ calcd for $C_7H_6F_2N_3O$ 186.0473, found 186.0474.

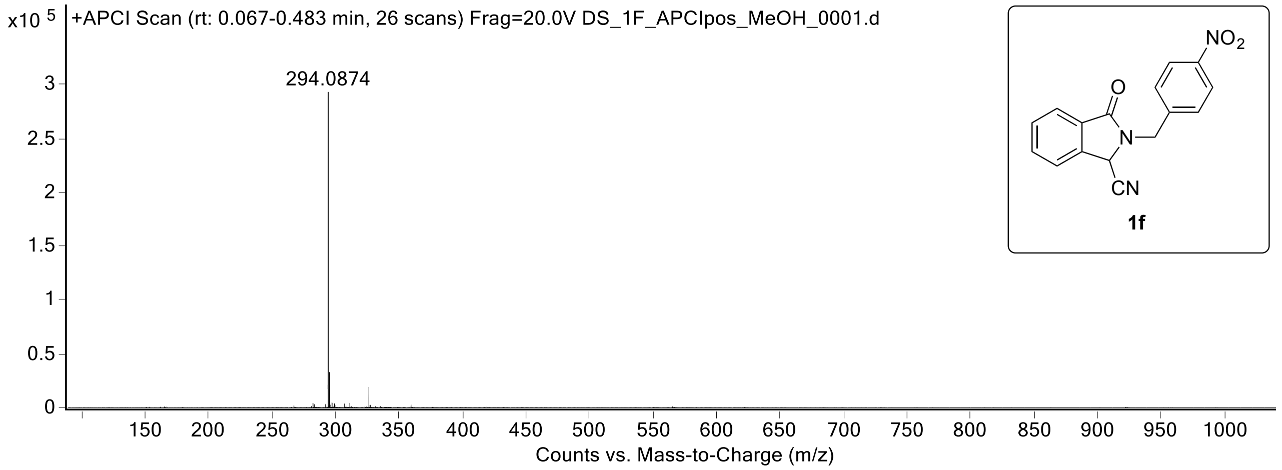

Compound **1f** HRMS (APCI) *m/z*: $[M + H]^+$ calcd for $C_{16}H_{12}N_3O_3$ 294.0873, found 294.0874.

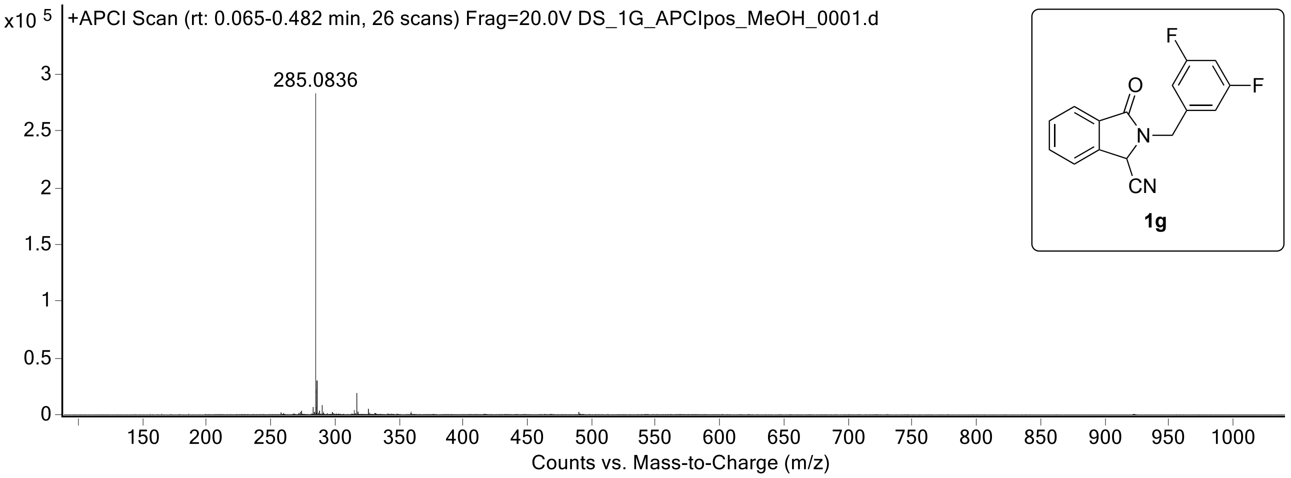

Compound **1g** HRMS (APCI) *m/z*: $[M + H]^+$ calcd for $C_{16}H_{11}F_2N_2O$ 285.0834, found 285.0836.

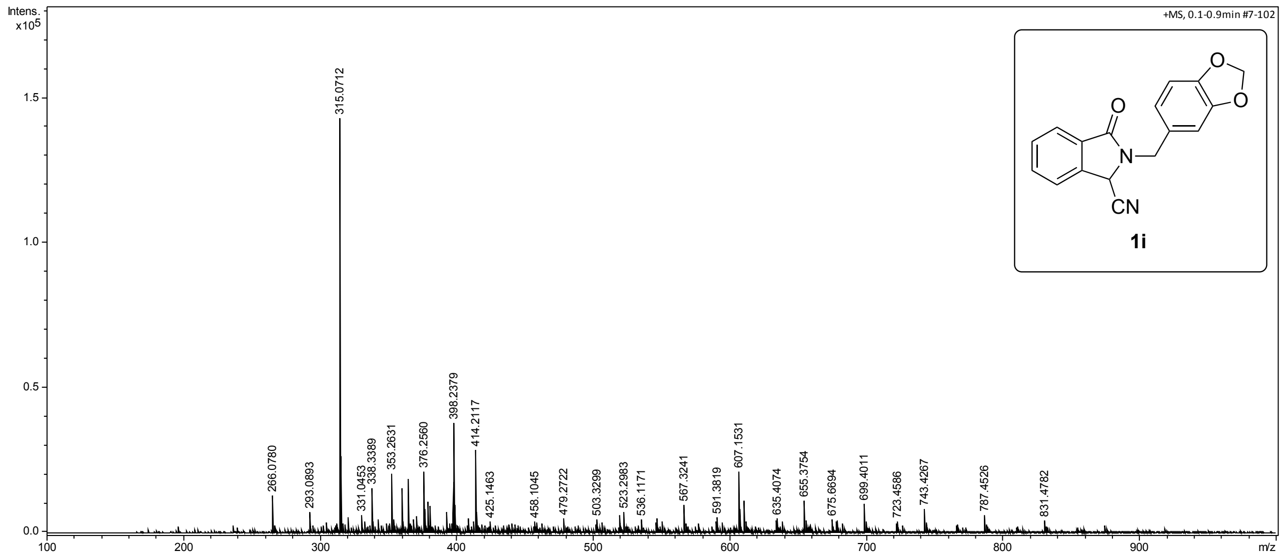

Compound **1i** HRMS (ESI-Q-TOF) *m/z*: $[M + H]^+$ calcd for $C_{17}H_{13}N_2O_3$ 293.0921, found 293.0893.

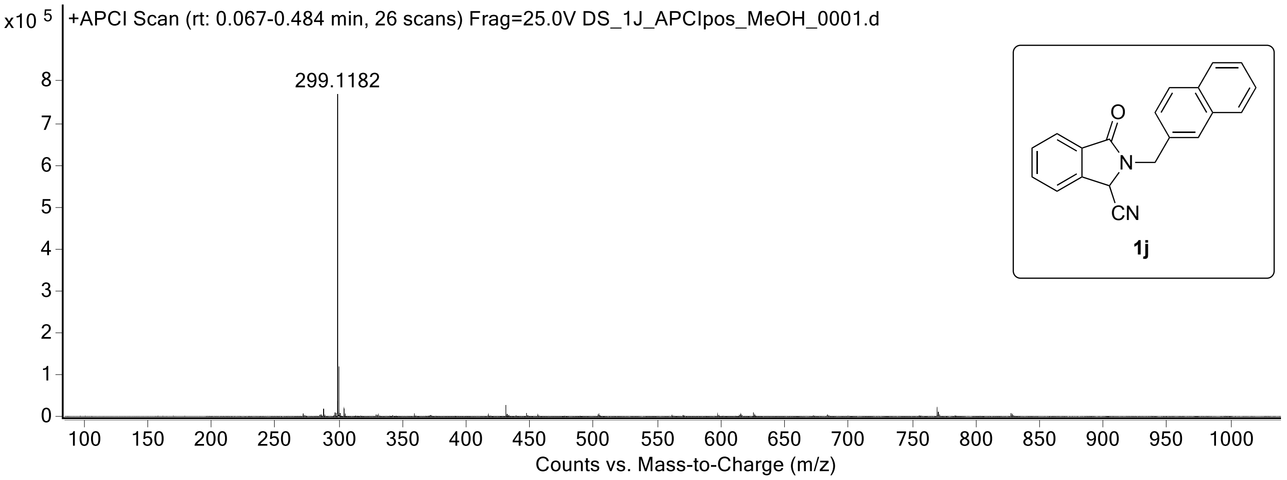

Compound **1j** HRMS (APCI) *m/z*: $[M + H]^+$ calcd for $C_{20}H_{15}N_2O$ 299.1179, found 299.1182.

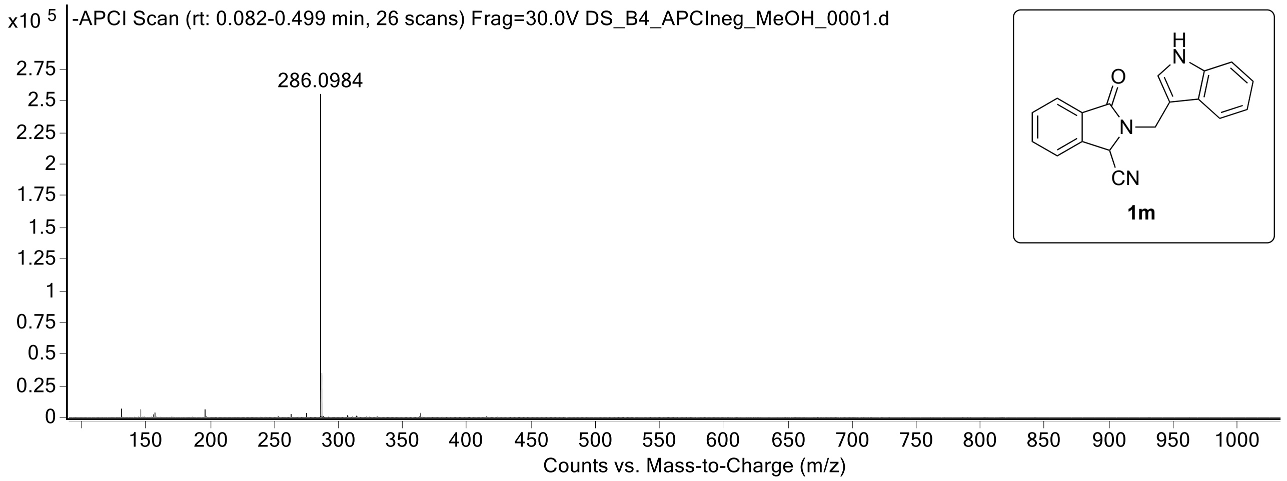

Compound **1m** HRMS (APCI) *m/z*: $[M - H]^-$ calcd for $C_{18}H_{12}N_3O$ 286.0986, found 286.0984.

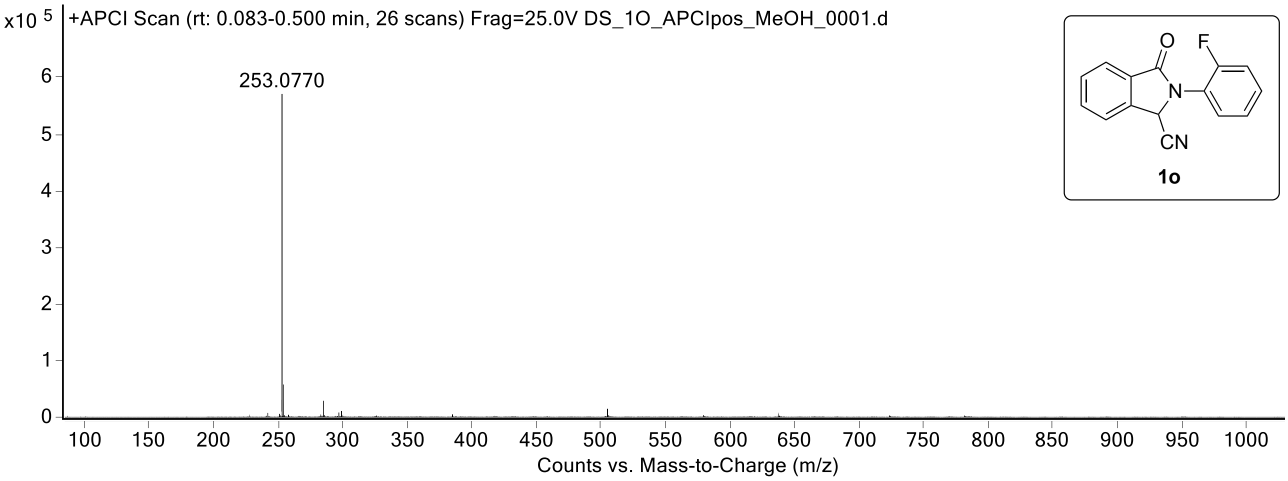

Compound **1o** HRMS (APCI) *m/z*: $[M + H]^+$ calcd for $C_{15}H_{10}FN_2O$ 253.0772, found 253.0770.

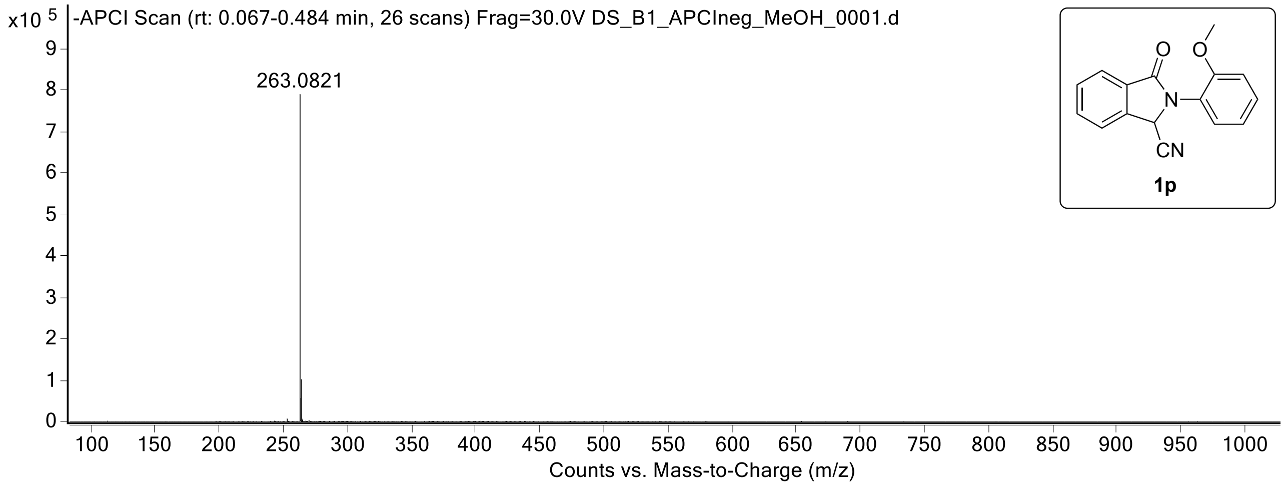

Compound **1p** HRMS (APCI) *m/z*: $[M{-}H]^-$ calcd for $C_{16}H_{11}N_2O_2$ 263.0823, found 263.0821.

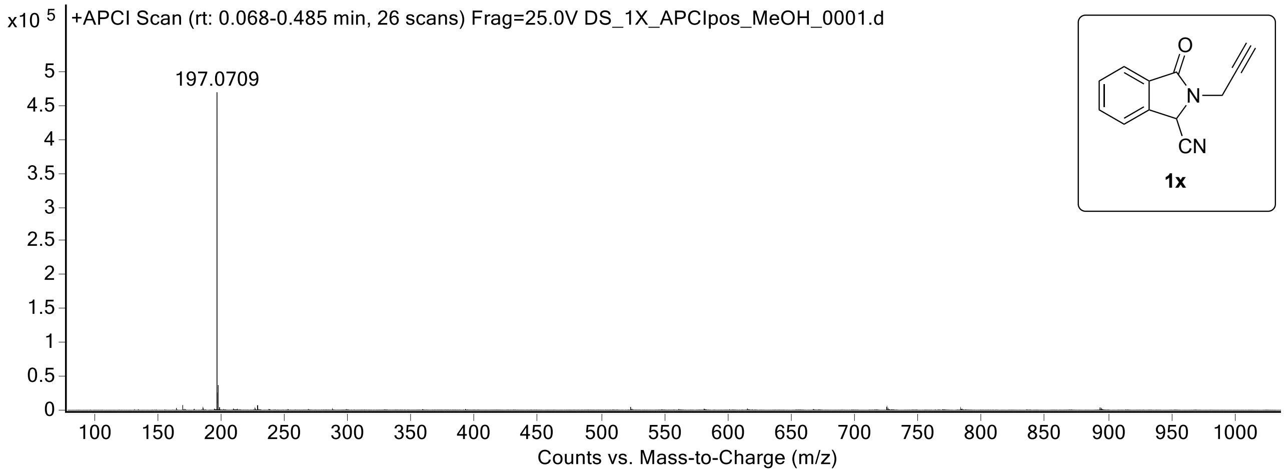

Compound **1x** HRMS (APCI) *m/z*: $[M + H]^+$ calcd for $C_{12}H_9N_2O$ 197.0709, found 197.0709.

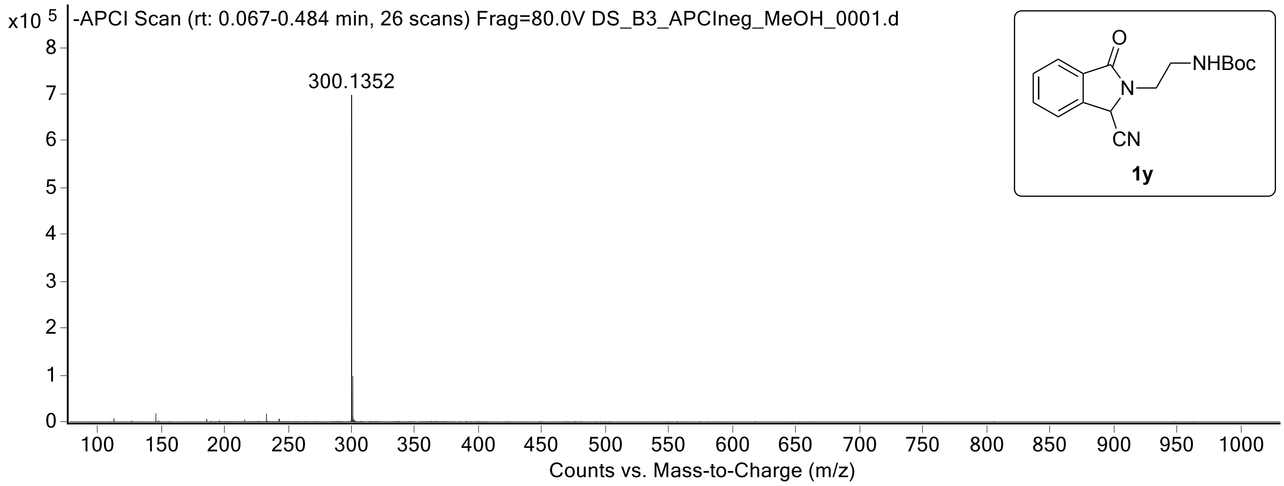

Compound **1y** HRMS (APCI) *m/z*: $[M - H]^-$ calcd for $C_{16}H_{18}N_3O_3$ 300.1354, found 300.1352.

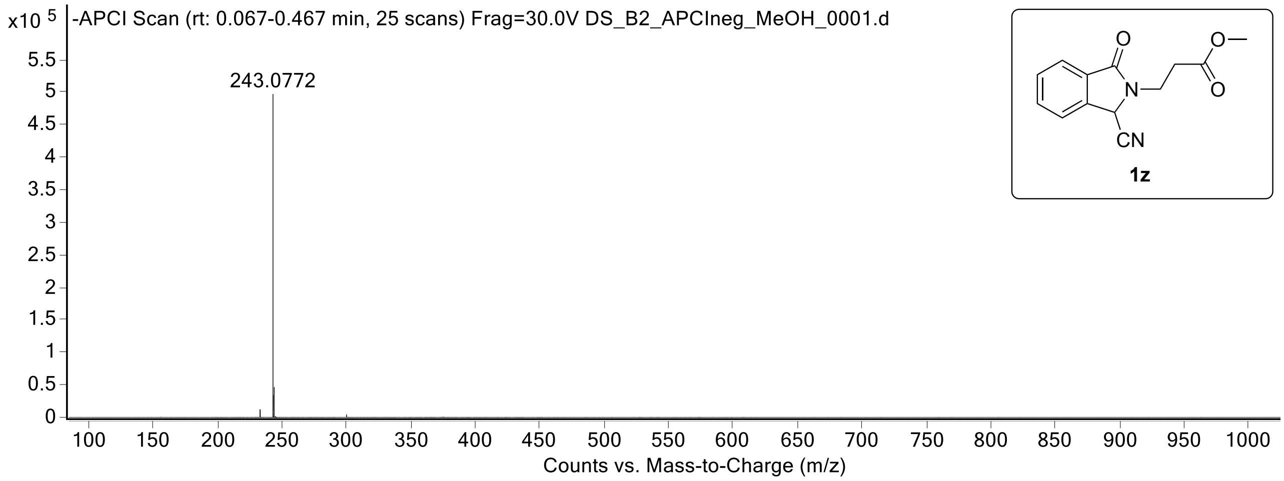

Compound **1z** HRMS (APCI) *m/z*: $[M - H]^-$ calcd for $C_{13}H_{11}N_2O_3$ 243.0775, found 243.0772.

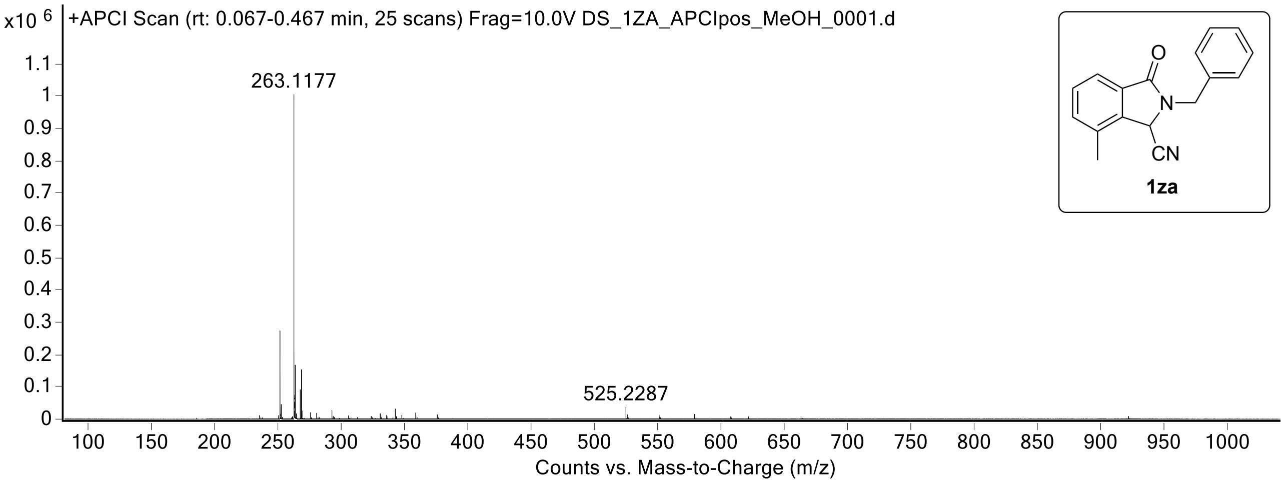

Compound **1za** HRMS (APCI) *m/z*: $[M + H]^+$ calcd for $C_{17}H_{15}N_2O$ 263.1179, found 263.1177.

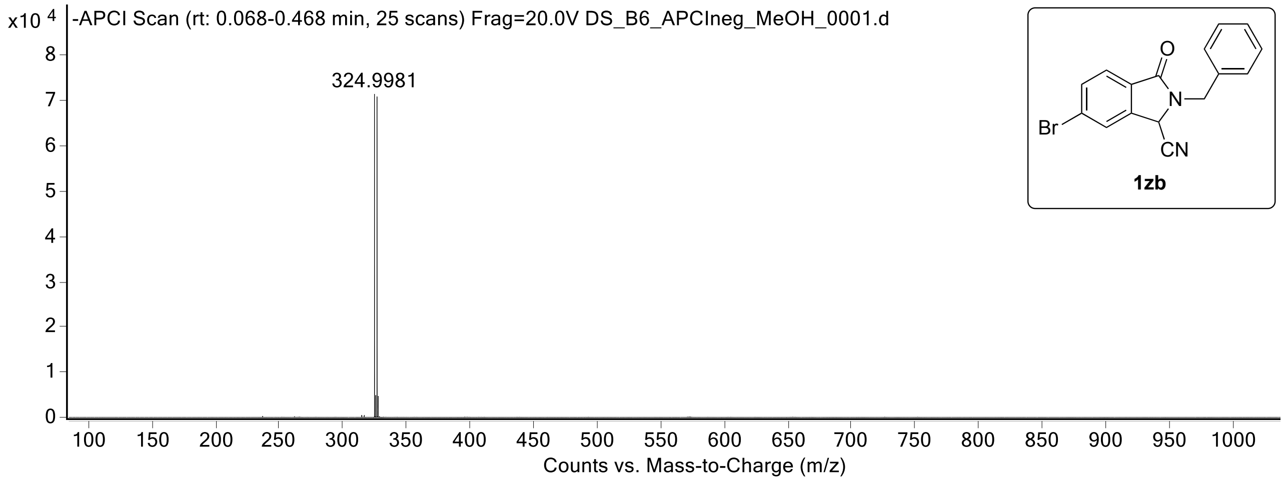

Compound **1zb** HRMS (APCI) *m/z*: $[M - H]^-$ calcd for $C_{16}H_{10}BrN_2O$ 324.9982, found 324.9981.

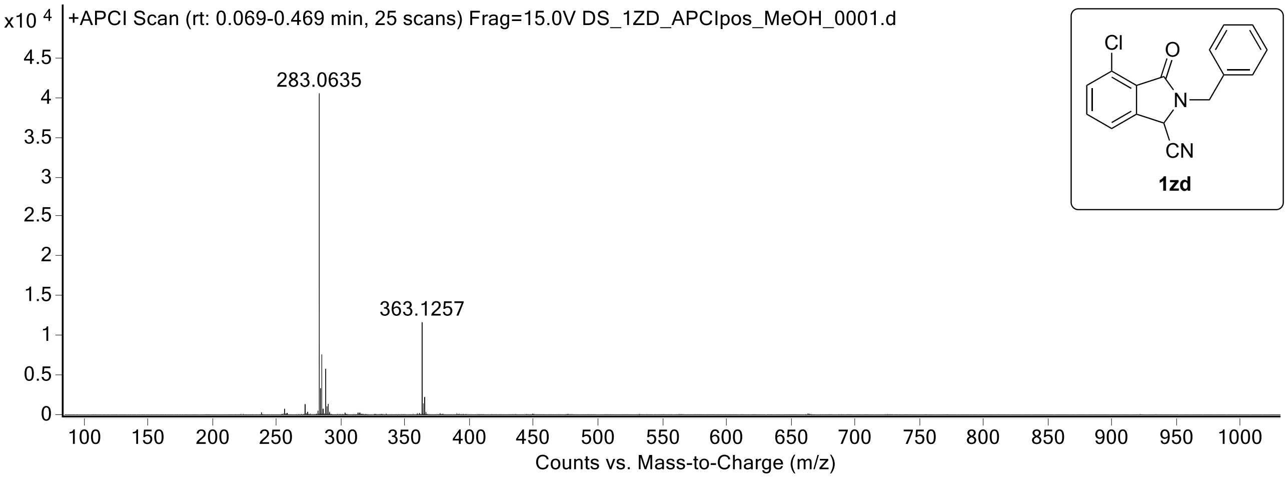

Compound **1zd** HRMS (APCI) *m/z*: $[M + H]^+$ calcd for $C_{16}H_{12}ClN_2O$ 283.0633, found 283.0635.

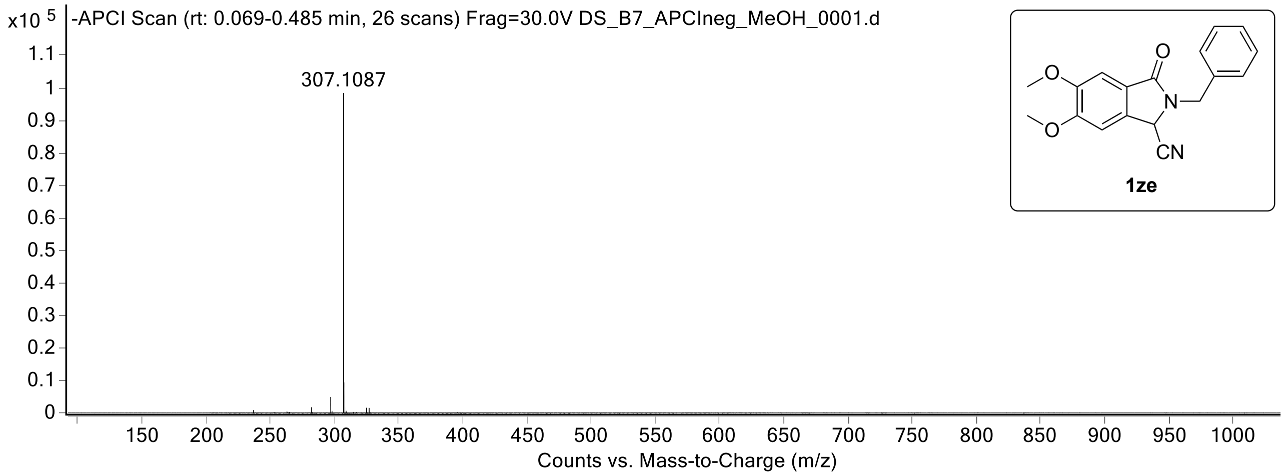

Compound **1ze** HRMS (APCI) *m/z*: $[M - H]^-$ calcd for $C_{18}H_{15}N_2O_3$ 307.1088, found 307.1087.

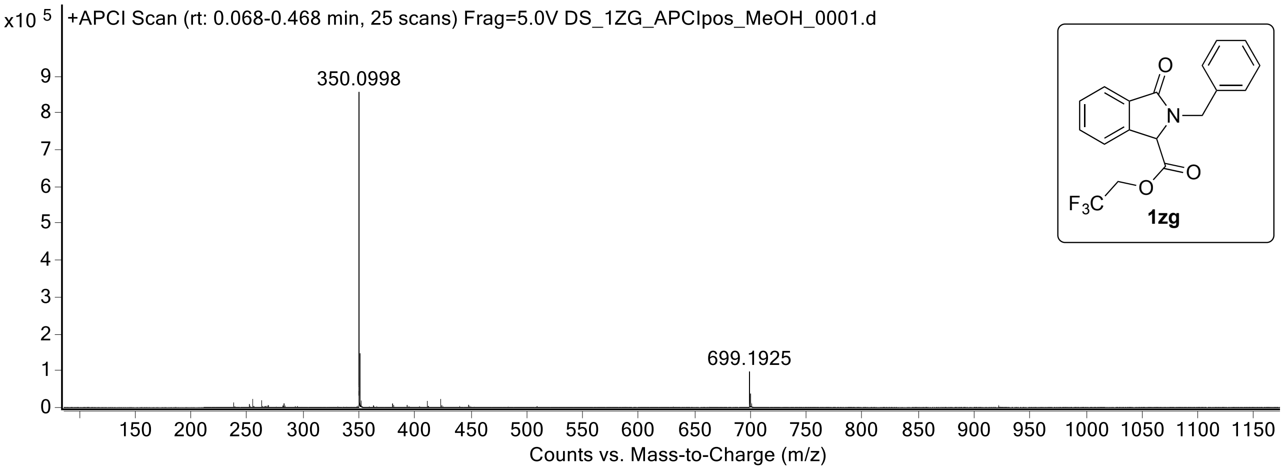

Compound **1zg** HRMS (APCI) *m/z*: $[M + H]^+$ calcd for $C_{18}H_{15}F_3NO_3$ 350.0999, found 350.0998.

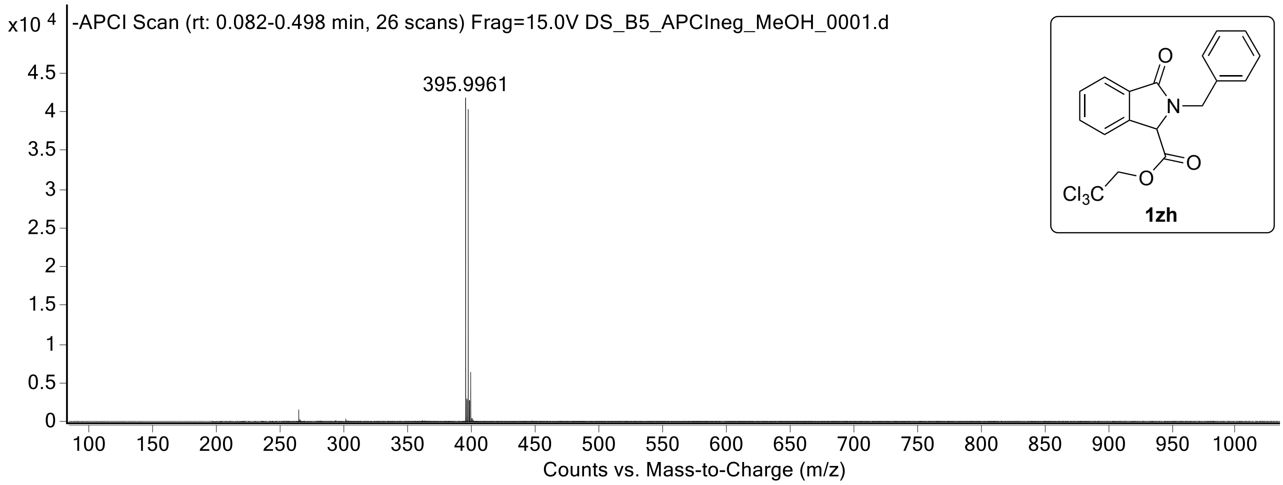

Compound **1zh** HRMS (APCI) *m/z*: $[M - H]^-$ calcd for $C_{18}H_{13}Cl_3NO_3$ 395.9966, found 395.9961.

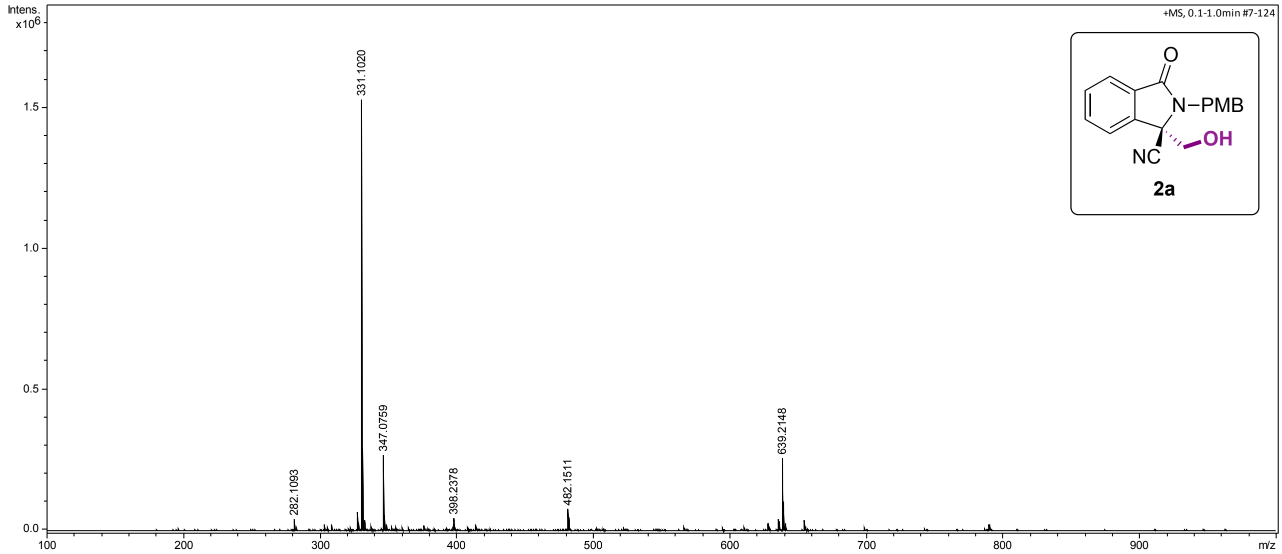

Compound **2a** HRMS (ESI-Q-TOF) *m/z*: $[M + Na]^+$ calcd for $C_{18}H_{16}N_2O_3Na$ 331.1053, found 331.1020.

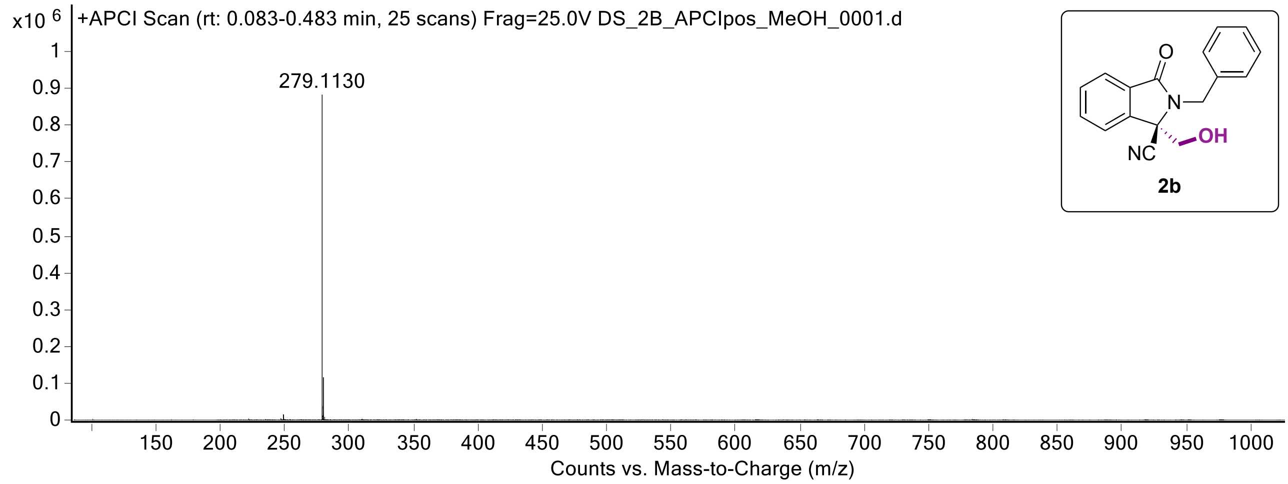

Compound **2b** HRMS (APCI) *m/z*: $[M + H]^+$ calcd for $C_{17}H_{15}N_2O_2$ 279.1128, found 279.1130.

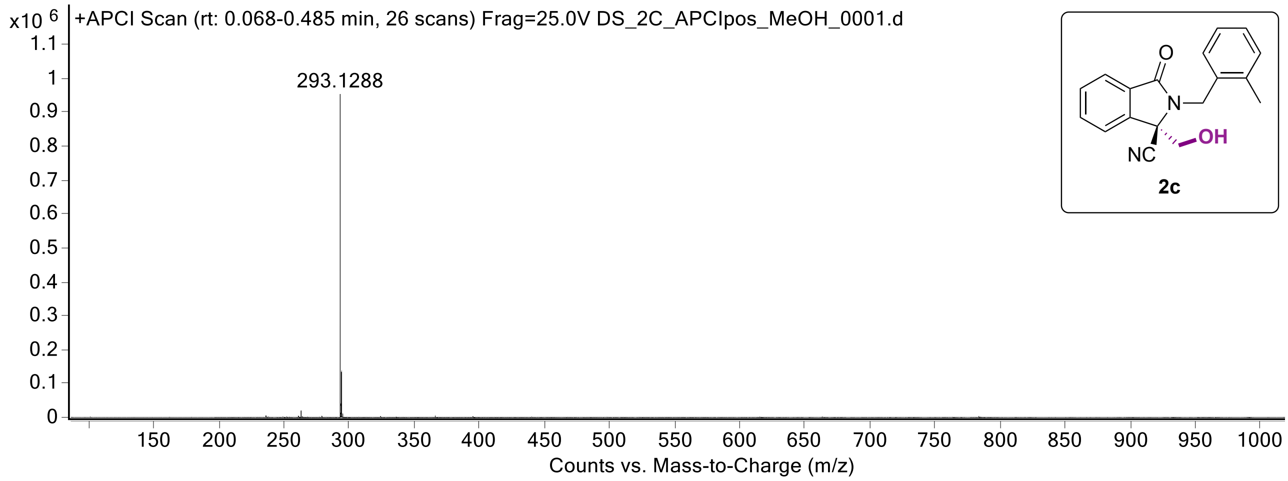

Compound **2c** HRMS (APCI) *m/z*: $[M + H]^+$ calcd for $C_{18}H_{17}N_2O_2$ 293.1285, found 293.1288.

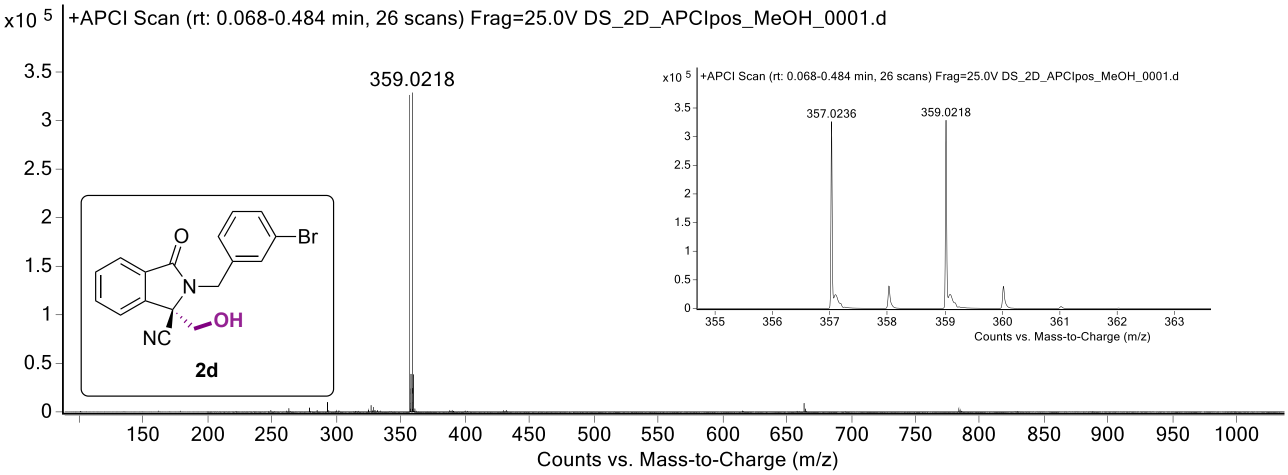

Compound **2d** HRMS (APCI) *m/z*: $[M + H]^+$ calcd for $C_{17}H_{14}BrN_2O_2$ 357.0233, found 357.0236.

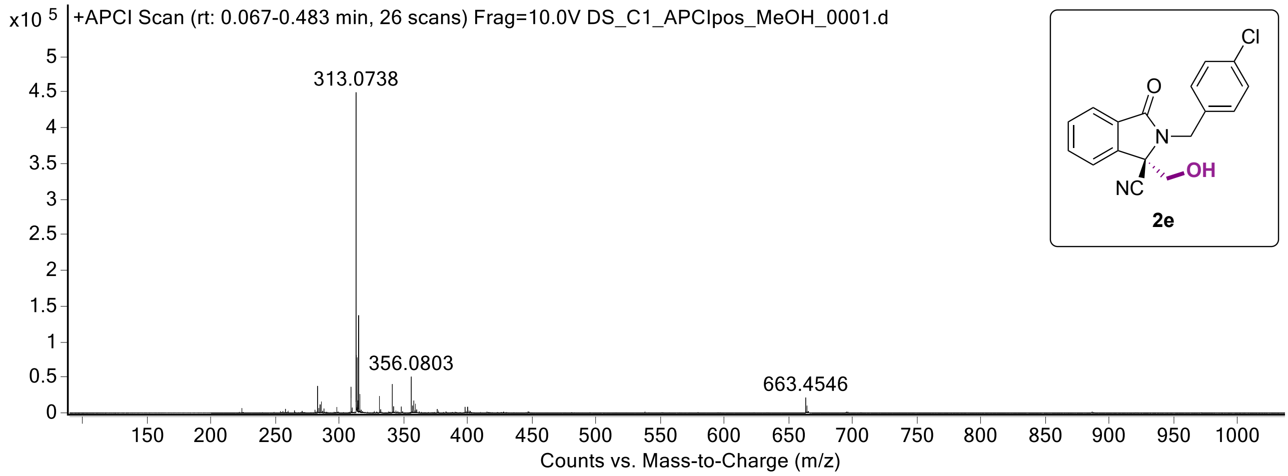

Compound **2e** HRMS (APCI) *m/z*: $[M + H]^+$ calcd for $C_{17}H_{14}ClN_2O_2$ 313.0738, found 313.0738.

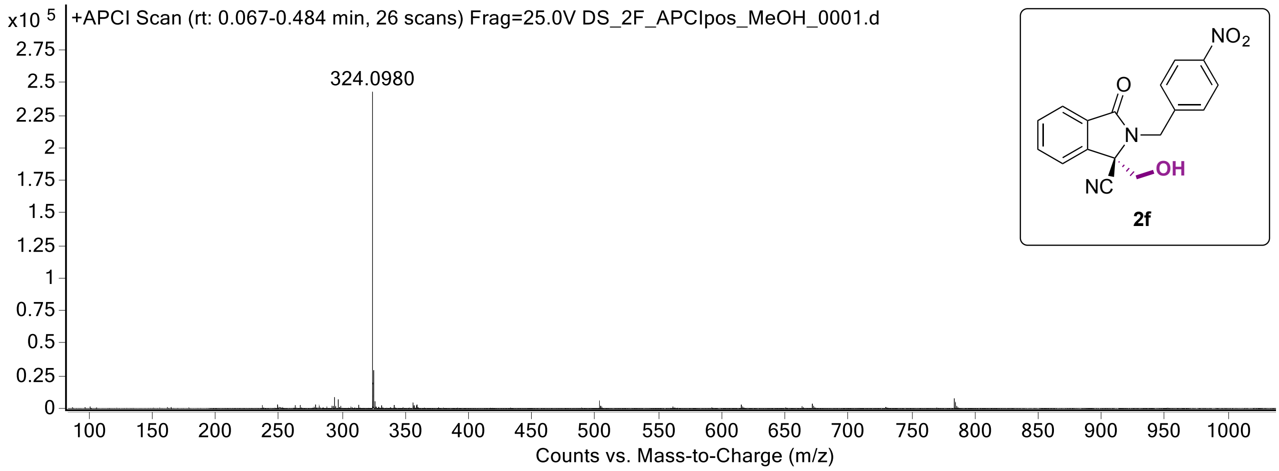

Compound **2f** HRMS (APCI) *m/z*: $[M + H]^+$ calcd for $C_{17}H_{14}N_3O_4$ 324.0979, found 324.0980.

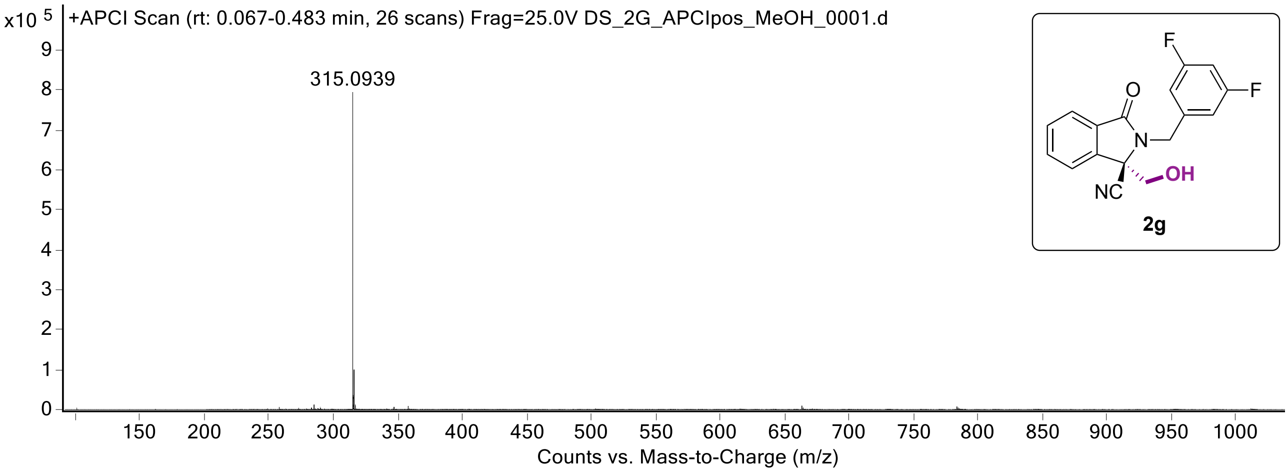

Compound **2g** HRMS (APCI) *m/z*: $[M + H]^+$ calcd for $C_{17}H_{13}F_2N_2O_2$ 315.0940, found 315.0939.

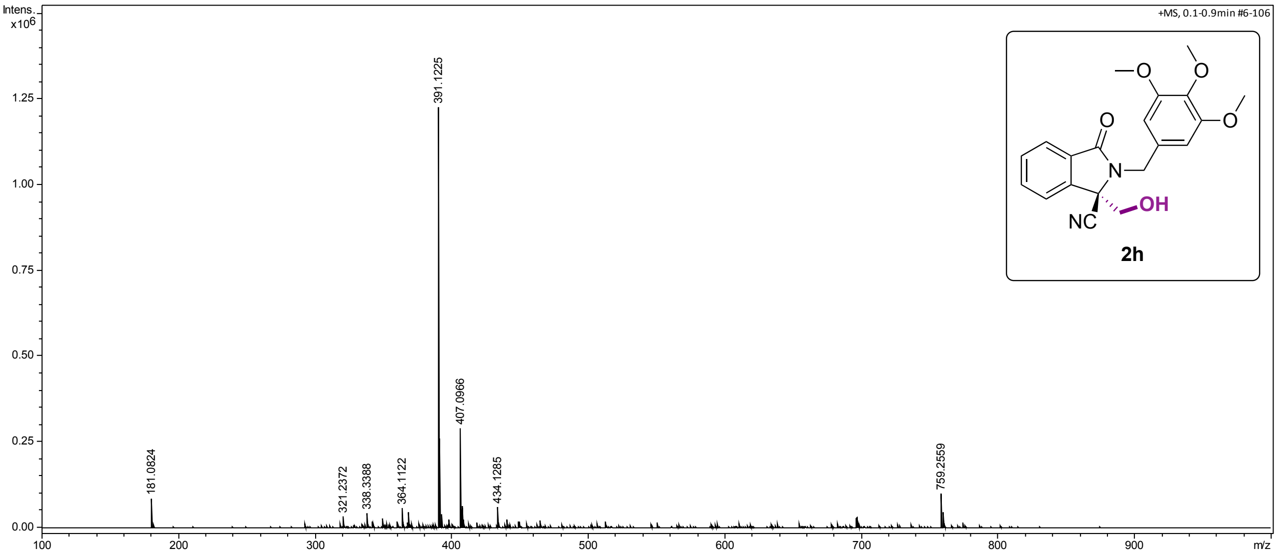

Compound **2h** HRMS (ESI-Q-TOF) *m/z*: $[M + Na]^+$ calcd for $C_{20}H_{20}N_2O_5Na$ 391.1264, found 391.1225.

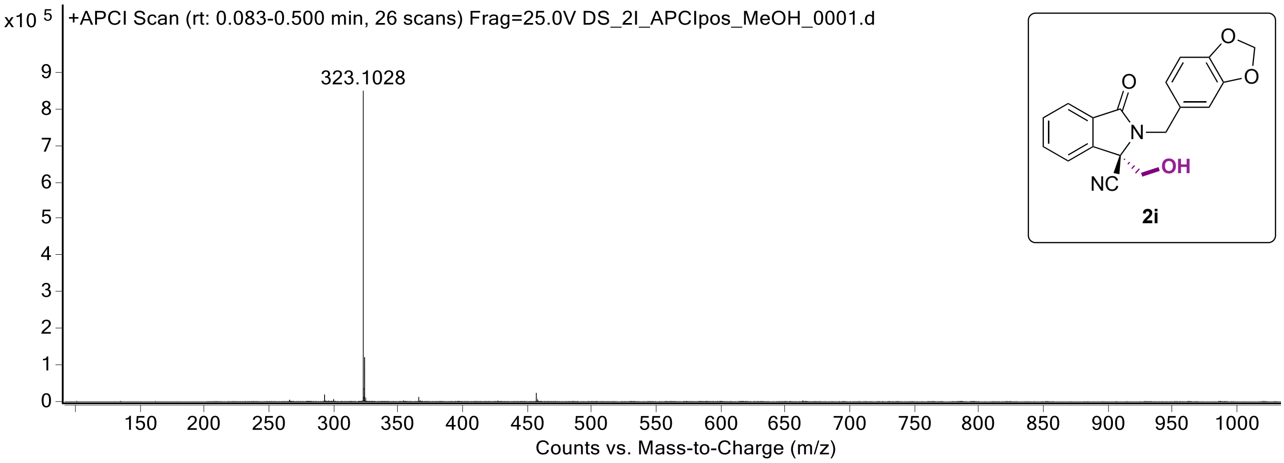

Compound **2i** HRMS (APCI) *m/z*: $[M + H]^+$ calcd for $C_{18}H_{15}N_2O_4$ 323.1026, found 323.1028.

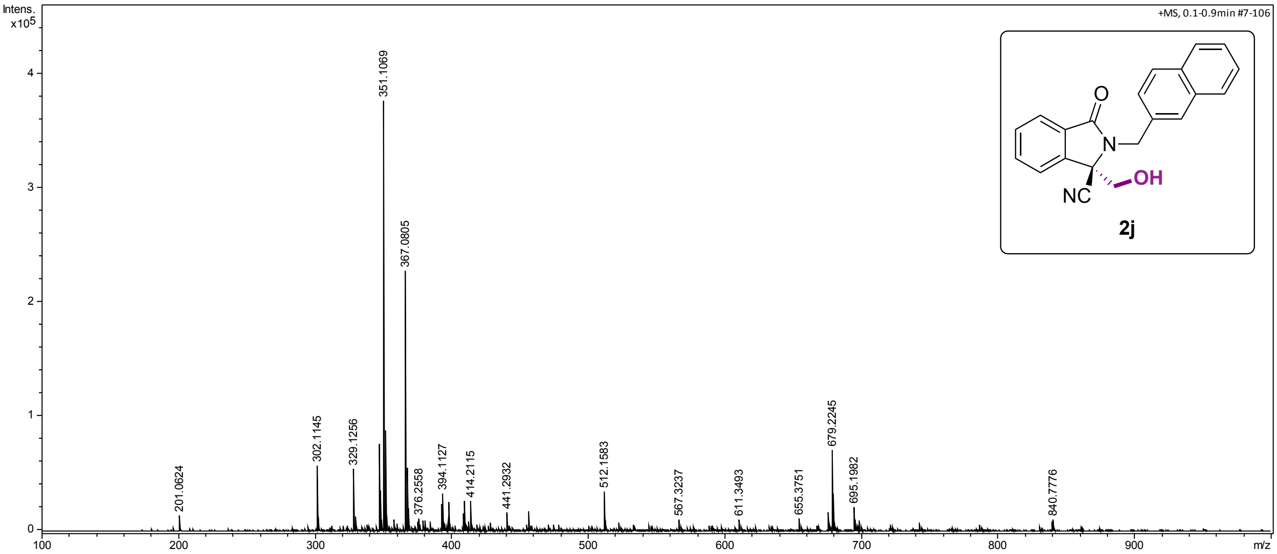

Compound **2j** HRMS (ESI-Q-TOF) *m/z*: $[M + H]^+$ calcd for $C_{21}H_{17}N_2O_2$ 329.1285, found 329.1256.

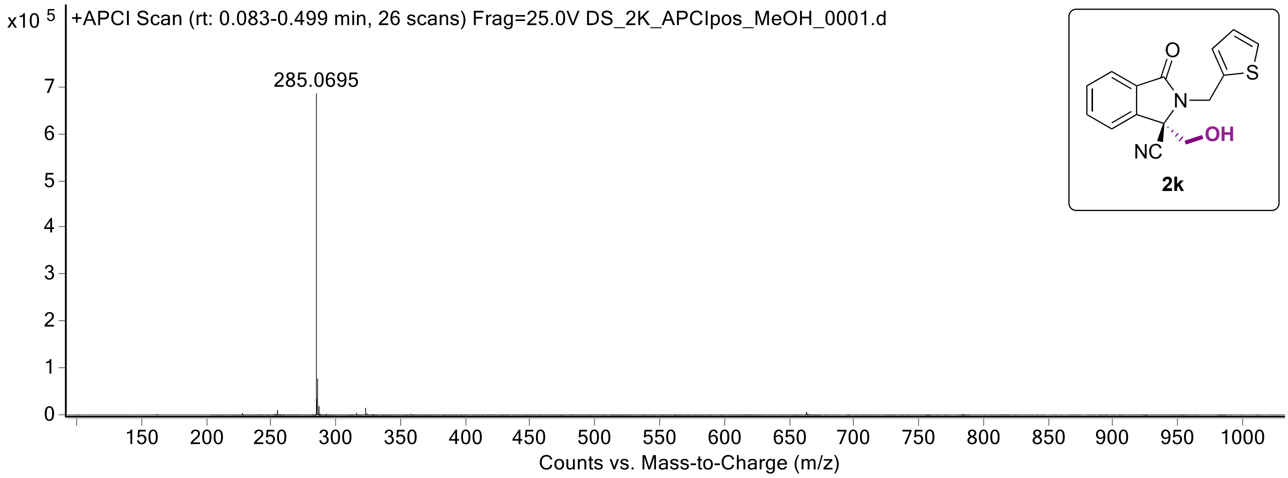

Compound **2k** HRMS (APCI) *m/z*: $[M + H]^+$ calcd for $C_{15}H_{13}N_2O_2S$ 285.0692, found 285.0695.

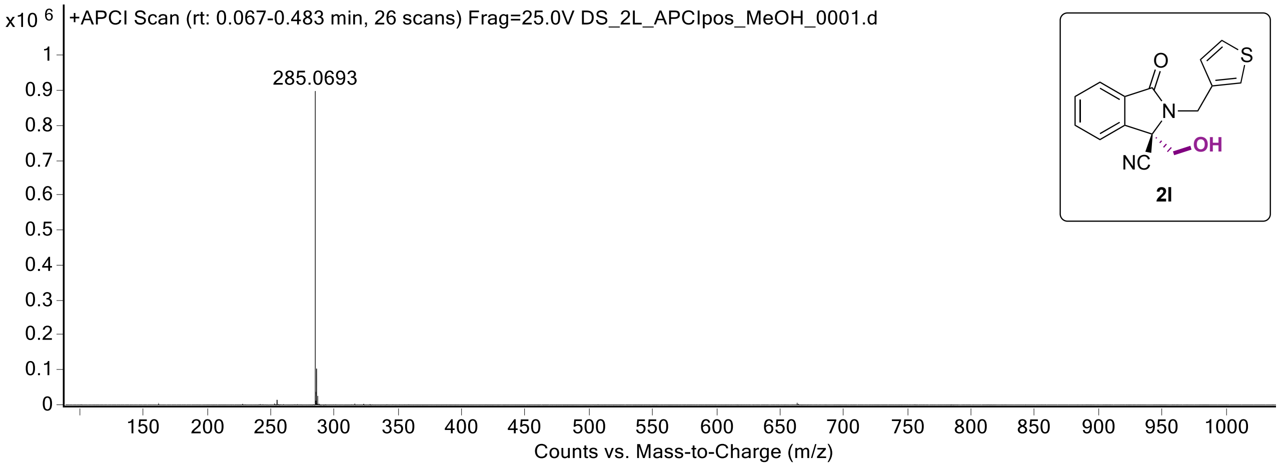

Compound **2l** HRMS (APCI) *m/z*: $[M + H]^+$ calcd for $C_{15}H_{13}N_2O_2S$ 285.0692, found 285.0693.

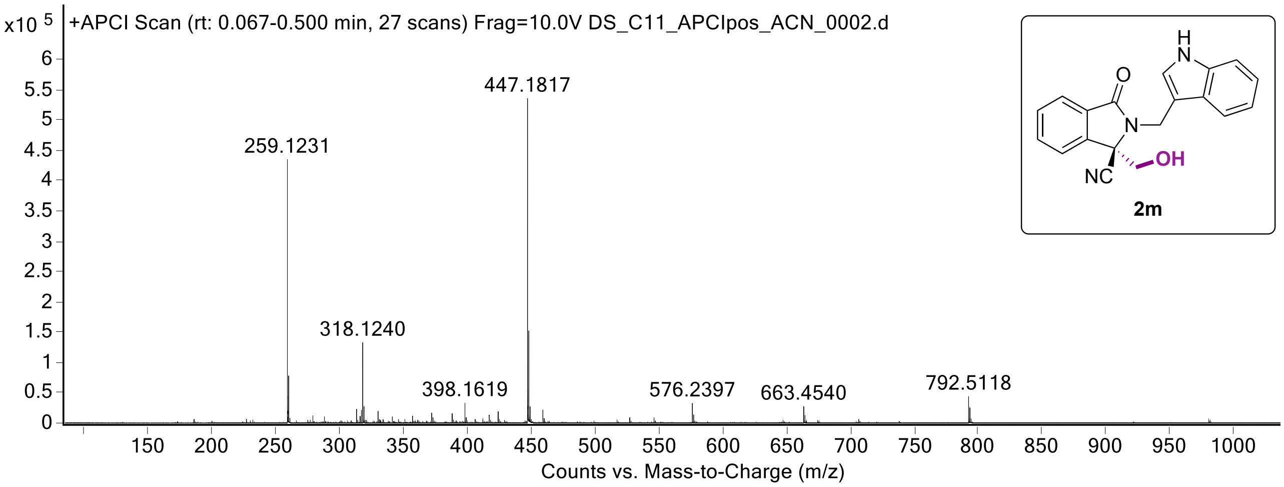

Compound **2m** HRMS (APCI) *m/z*: $[M + H]^+$ calcd for $C_{19}H_{16}N_3O_2$ 318.1237, found 318.1240.

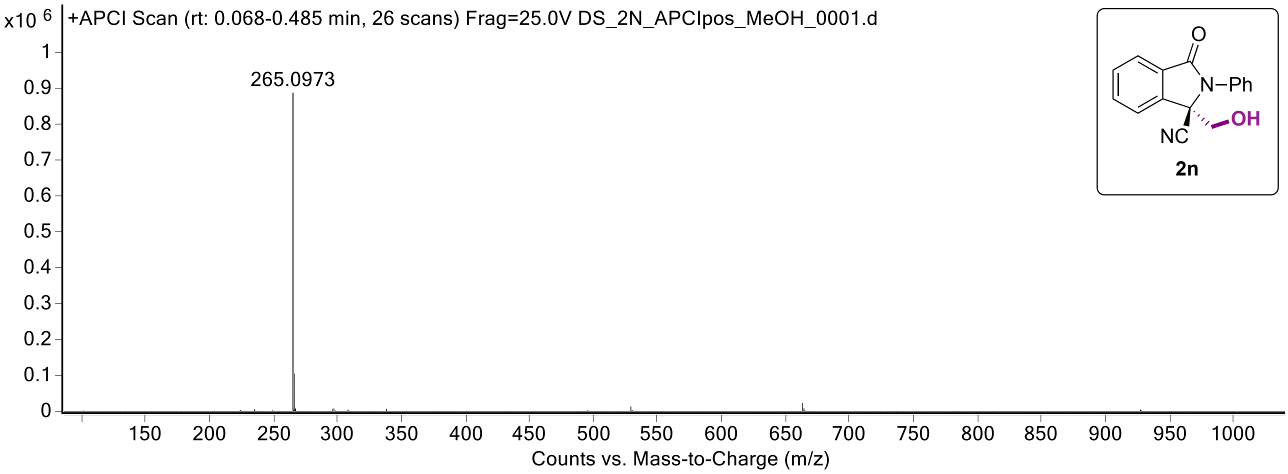

Compound **2n** HRMS (APCI) *m/z*: $[M + H]^+$ calcd for $C_{16}H_{13}N_2O_2$ 265.0972, found 265.0973.

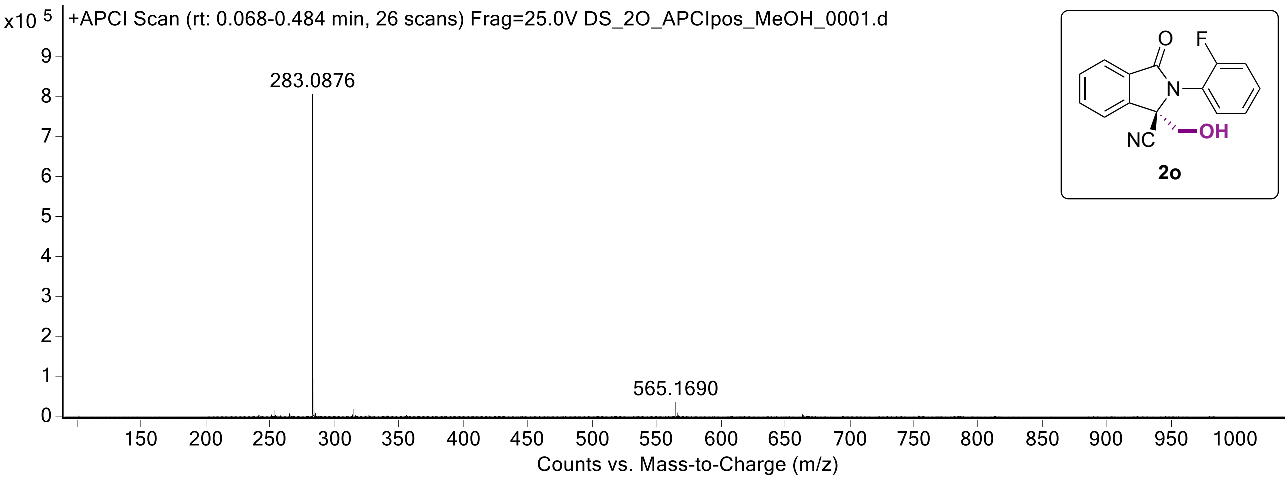

Compound **2o** HRMS (APCI) *m/z*: $[M + H]^+$ calcd for $C_{16}H_{12}FN_2O_2$ 283.0877, found 283.0876.

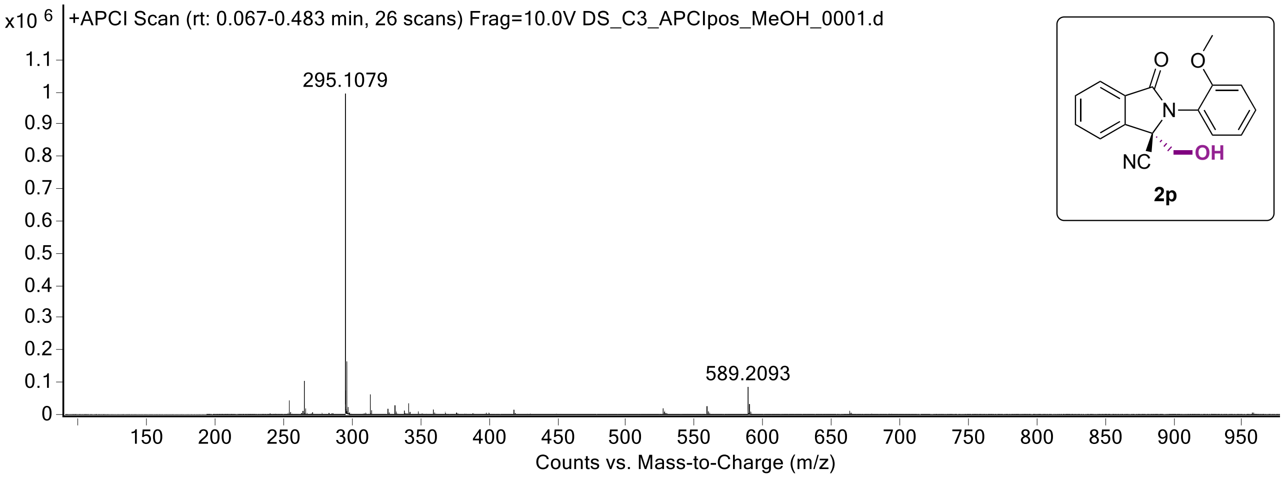

Compound **2p** HRMS (APCI) *m/z*: $[M + H]^+$ calcd for $C_{17}H_{15}N_2O_3$ 295.1077, found 295.1079.

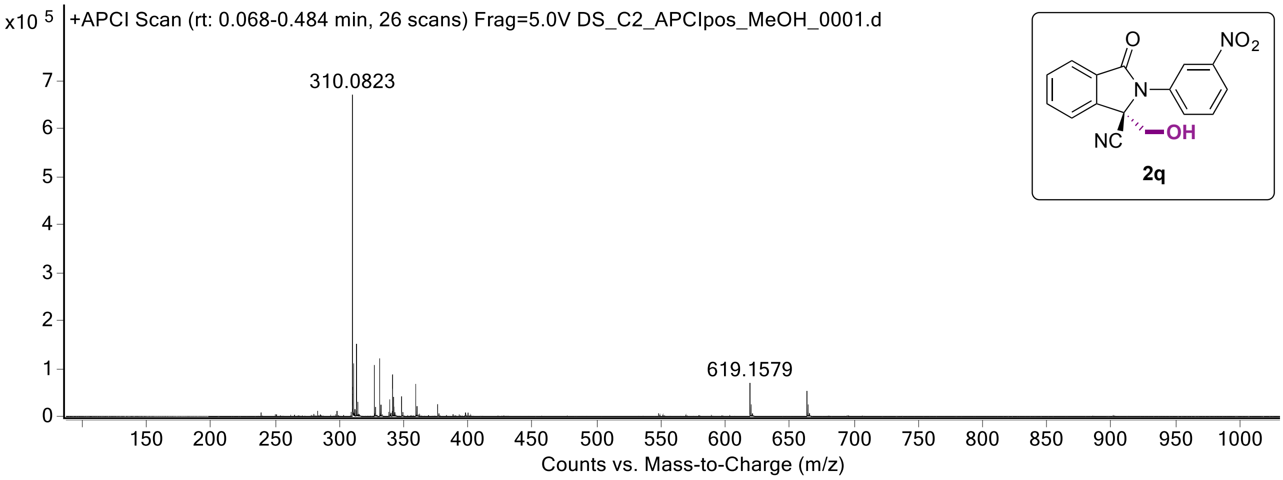

Compound **2q** HRMS (APCI) *m/z*: $[M + H]^+$ calcd for $C_{16}H_{12}N_3O_4$ 310.0822, found 310.0823.

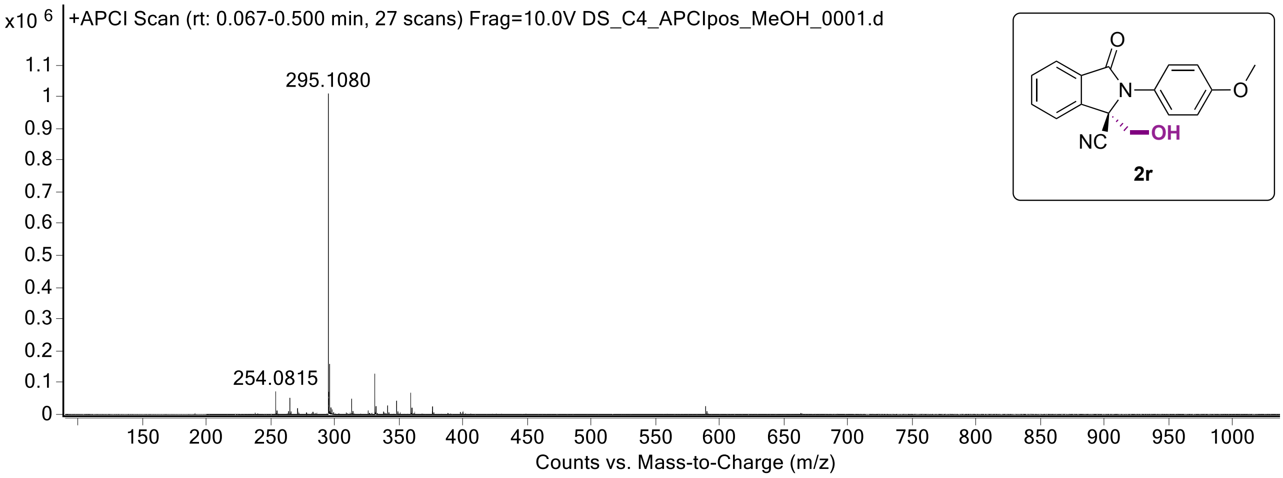

Compound **2r** HRMS (APCI) *m/z*: $[M + H]^+$ calcd for $C_{17}H_{15}N_2O_3$ 295.1077, found 295.1080.

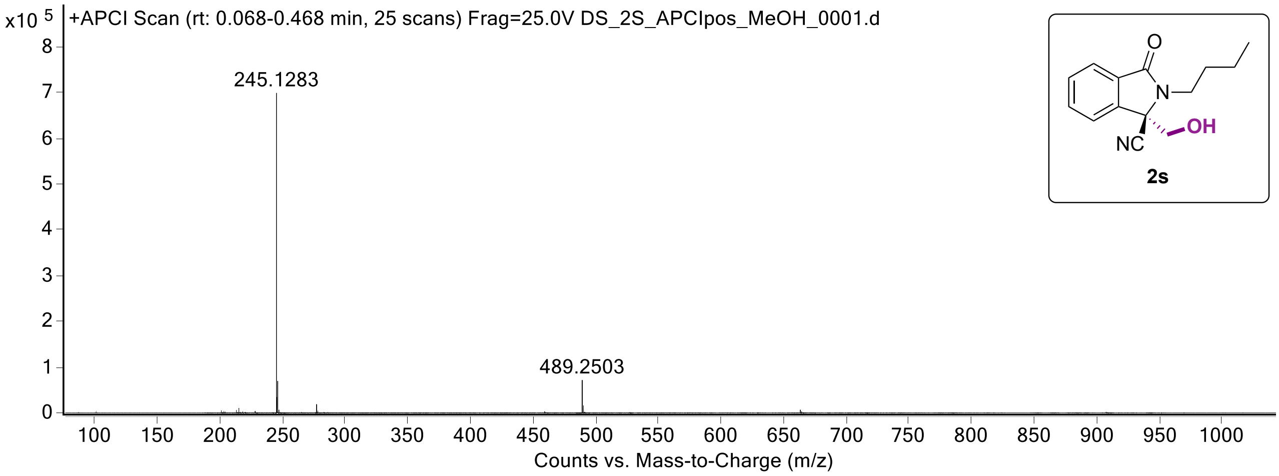

Compound **2s** HRMS (APCI) *m/z*: $[M + H]^+$ calcd for $C_{14}H_{17}N_2O_2$ 245.1285, found 245.1283.

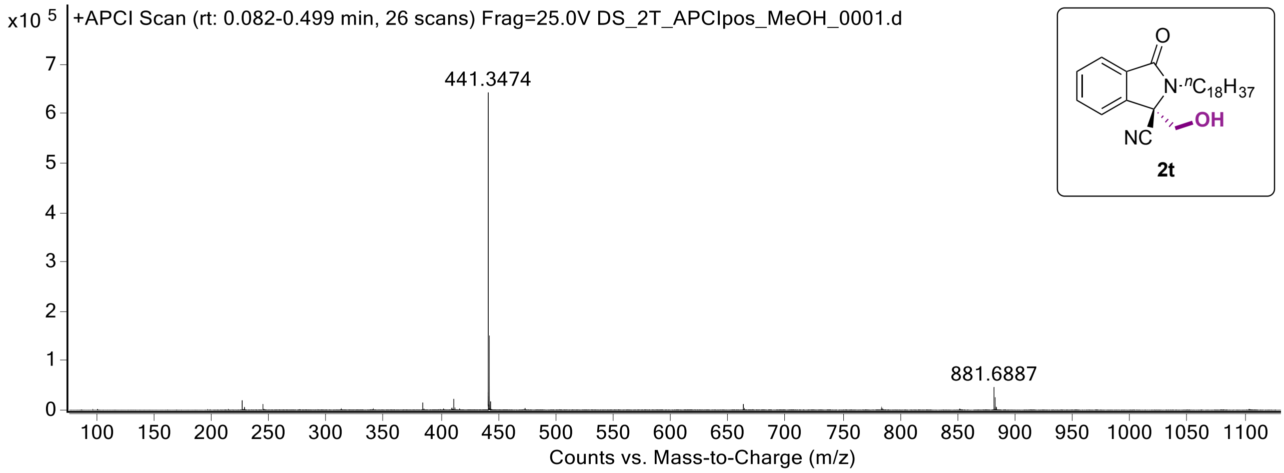

Compound **2t** HRMS (APCI) *m/z*: $[M + H]^+$ calcd for $C_{28}H_{45}N_2O_2$ 441.3476, found 441.3474.

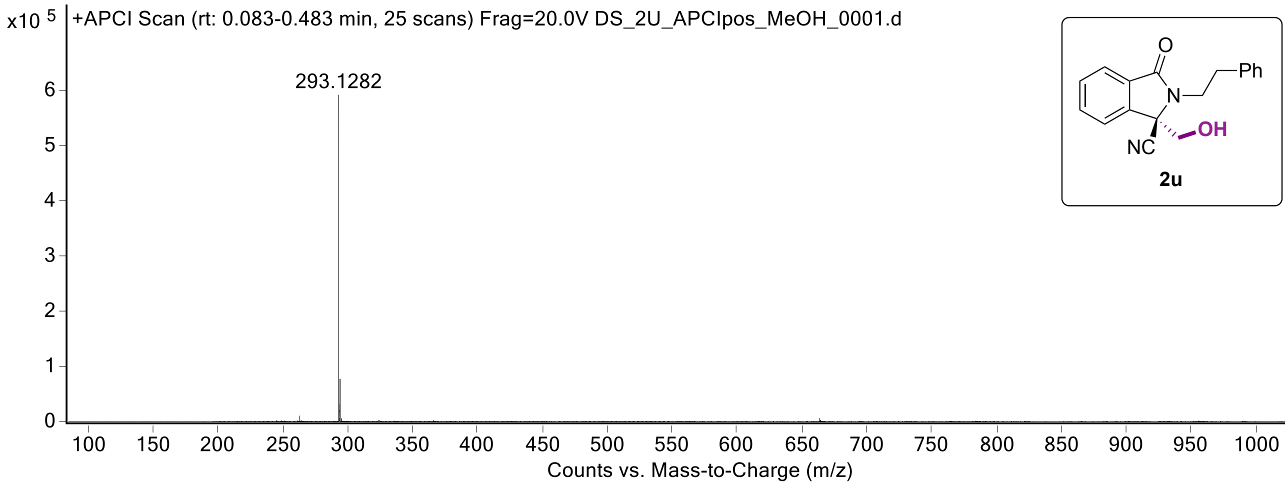

Compound **2u** HRMS (APCI) *m/z*: $[M + H]^+$ calcd for $C_{18}H_{17}N_2O_2$ 293.1285, found 293.1282.

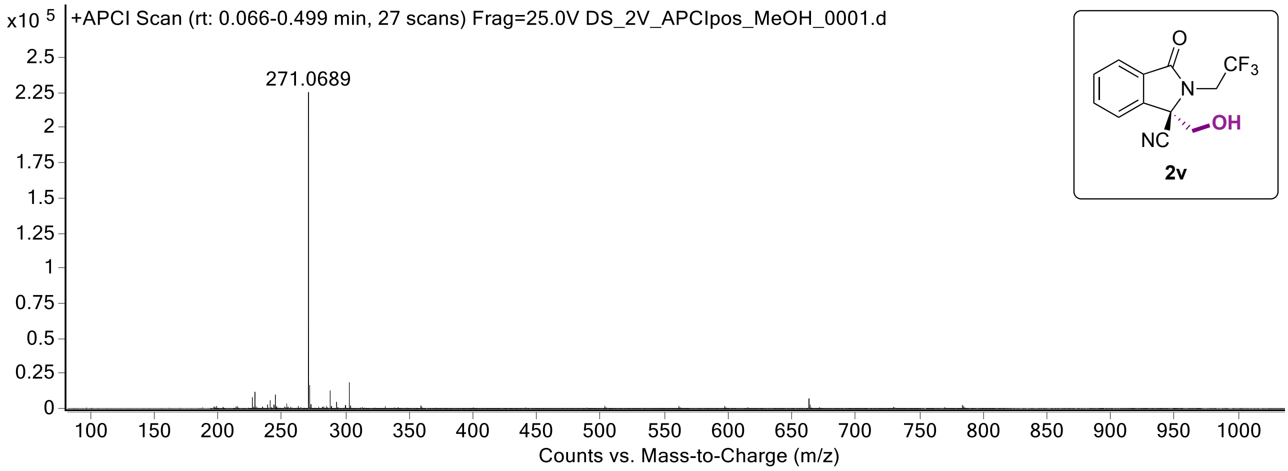

Compound **2v** HRMS (APCI) *m/z*: $[M + H]^+$ calcd for $C_{12}H_{10}F_3N_2O_2$ 271.0689, found 271.0689.

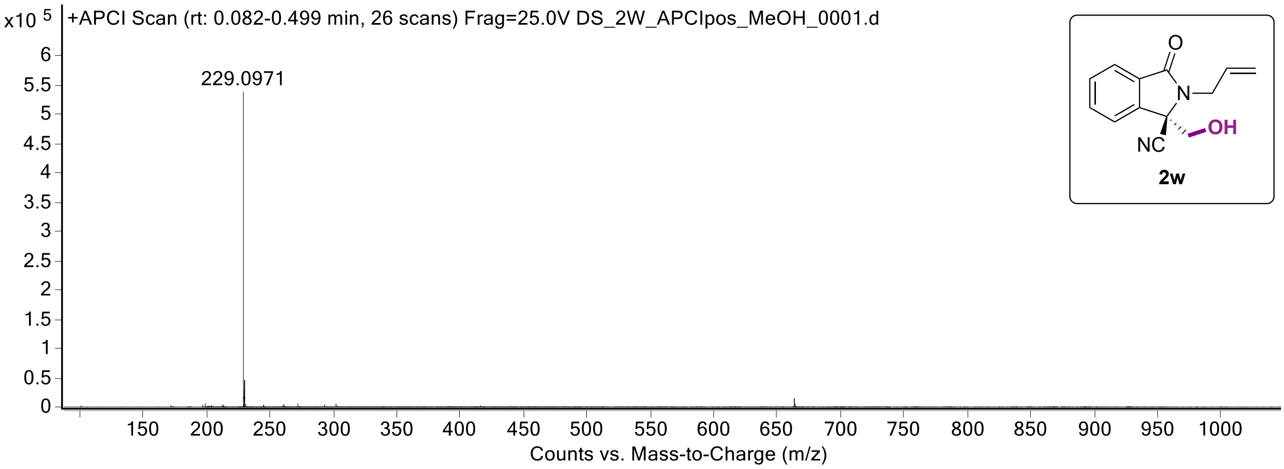

Compound **2w** HRMS (APCI) *m/z*: $[M + H]^+$ calcd for $C_{13}H_{13}N_2O_2$ 229.0972, found 229.0971.

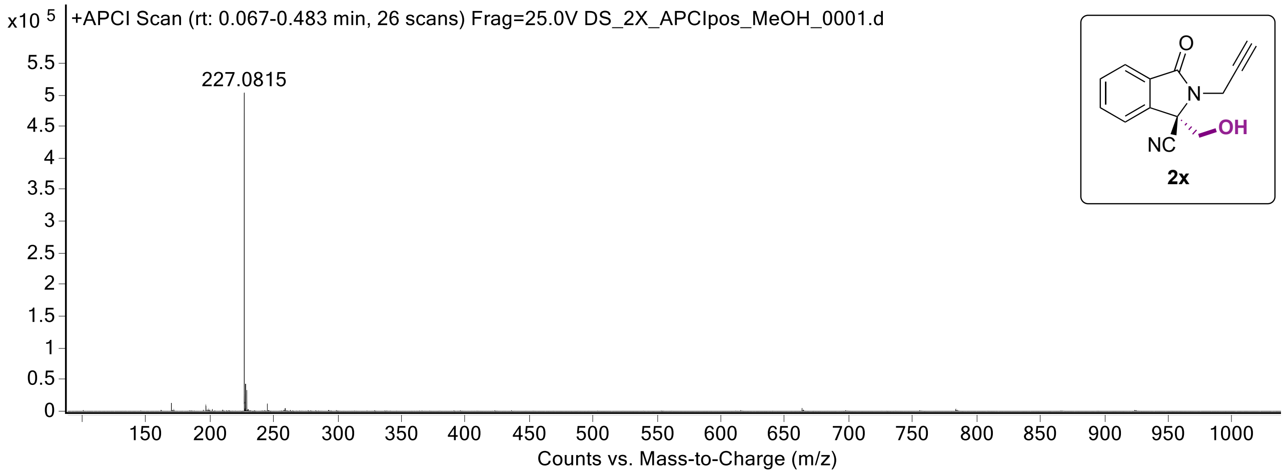

Compound **2x** HRMS (APCI) *m/z*: $[M + H]^+$ calcd for $C_{13}H_{11}N_2O_2$ 227.0815, found 227.0815.

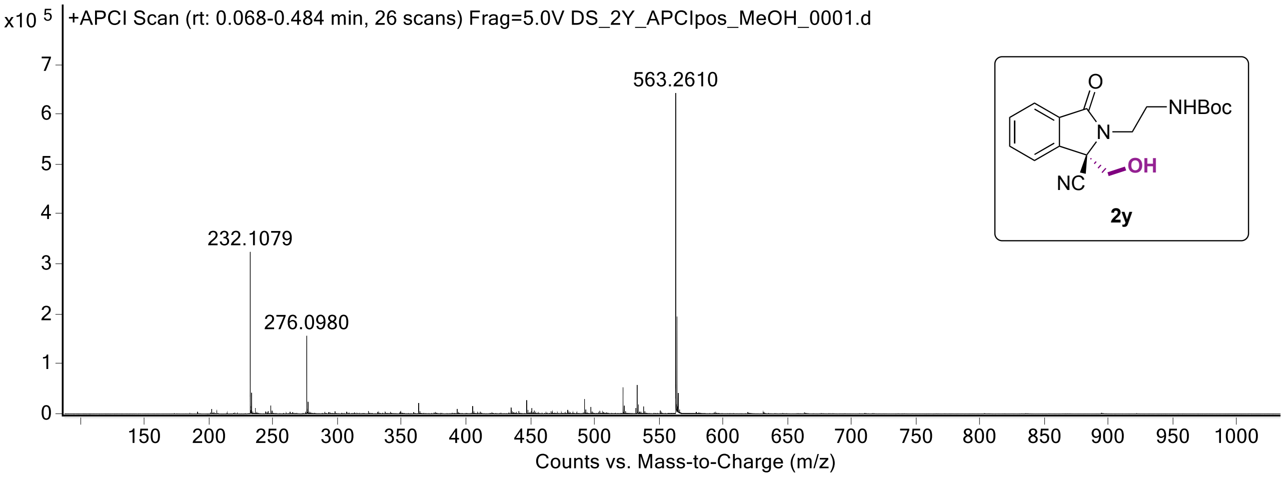

Compound **2y** HRMS (APCI) *m/z*: $[M + H - Boc + H]^+$ calcd for $C_{12}H_{14}N_3O_2$ 232.1081, found 232.1079.

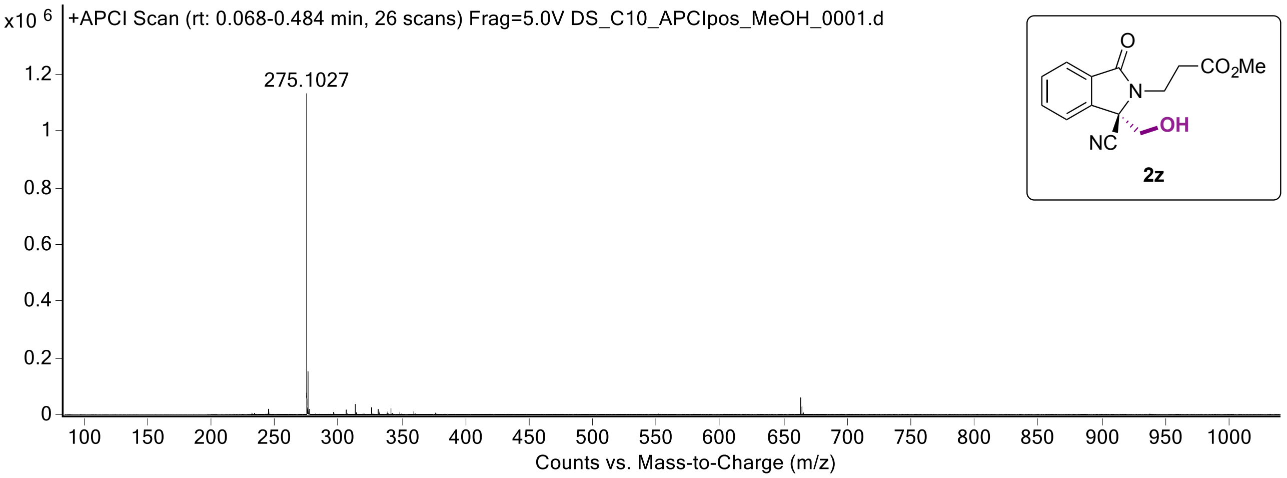

Compound **2z** HRMS (APCI) *m/z*: $[M + H]^+$ calcd for $C_{14}H_{15}N_2O_4$ 275.1026, found 275.1027.

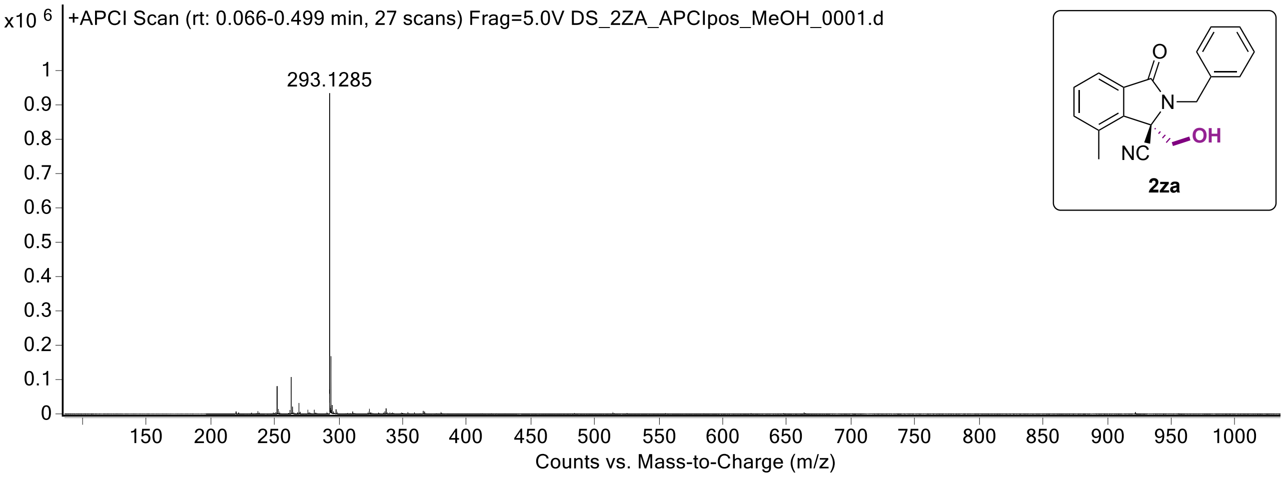

Compound **2za** HRMS (APCI) *m/z*: $[M + H]^+$ calcd for $C_{18}H_{17}N_2O_2$ 293.1285, found 293.1285.

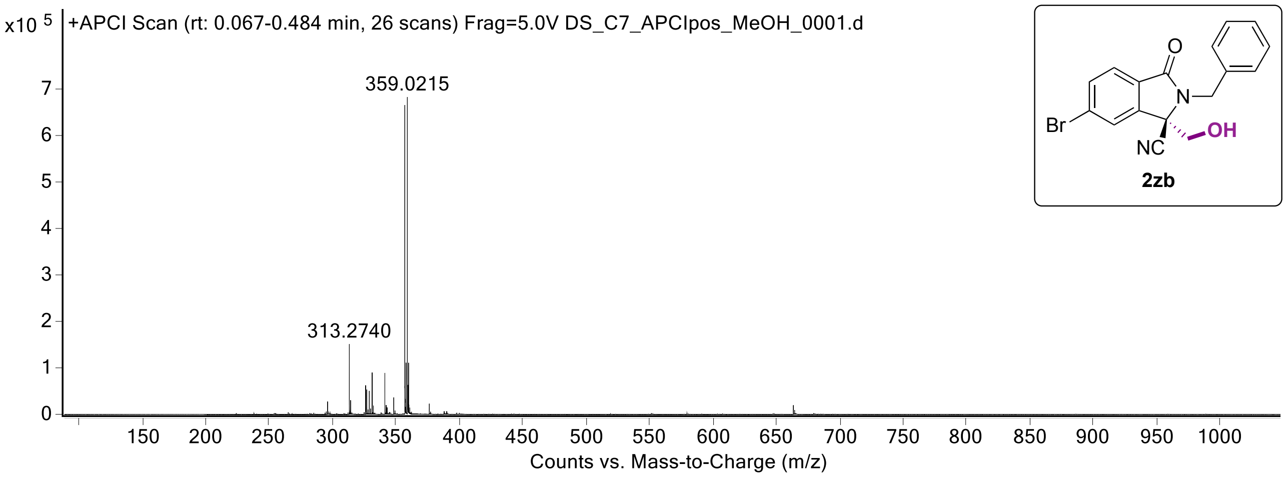

Compound **2zb** HRMS (APCI) *m/z*: $[M + H]^+$ calcd for $C_{17}H_{14}BrN_2O_2$ 357.0233, found 357.0234.

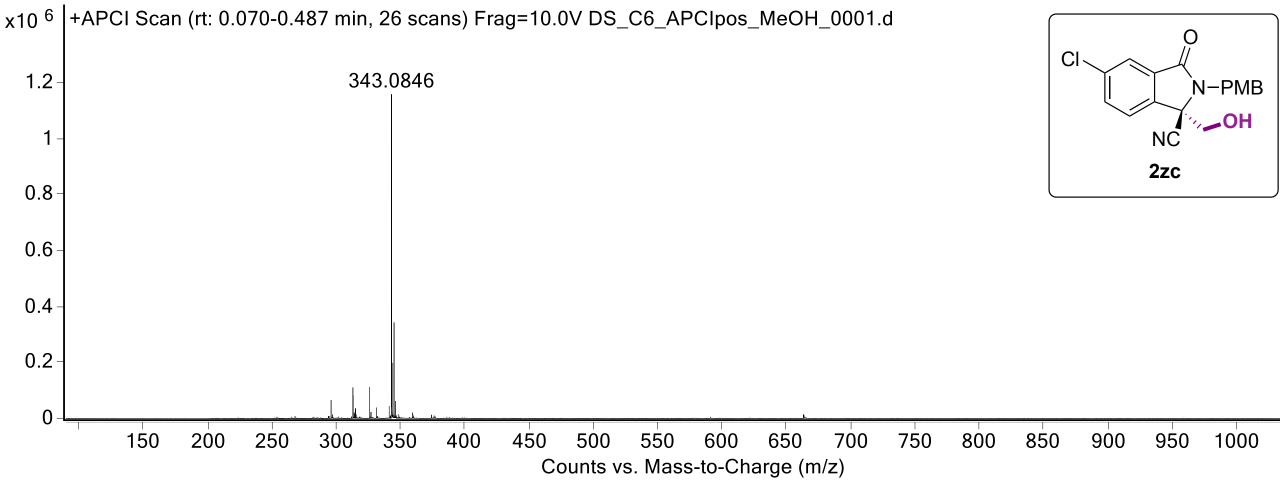

Compound **2zc** HRMS (APCI) *m/z*: $[M + H]^+$ calcd for $C_{18}H_{16}ClN_2O_3$ 343.0844, found 343.0846.

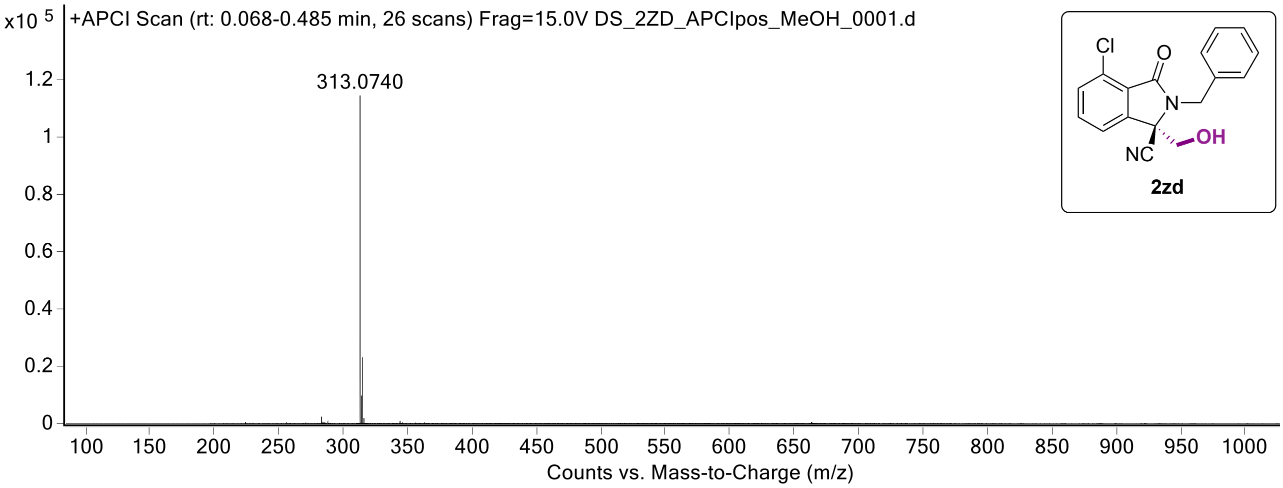

Compound **2zd** HRMS (APCI) *m/z*: $[M + H]^+$ calcd for $C_{17}H_{14}ClN_2O_2$ 313.0738, found 313.0740.

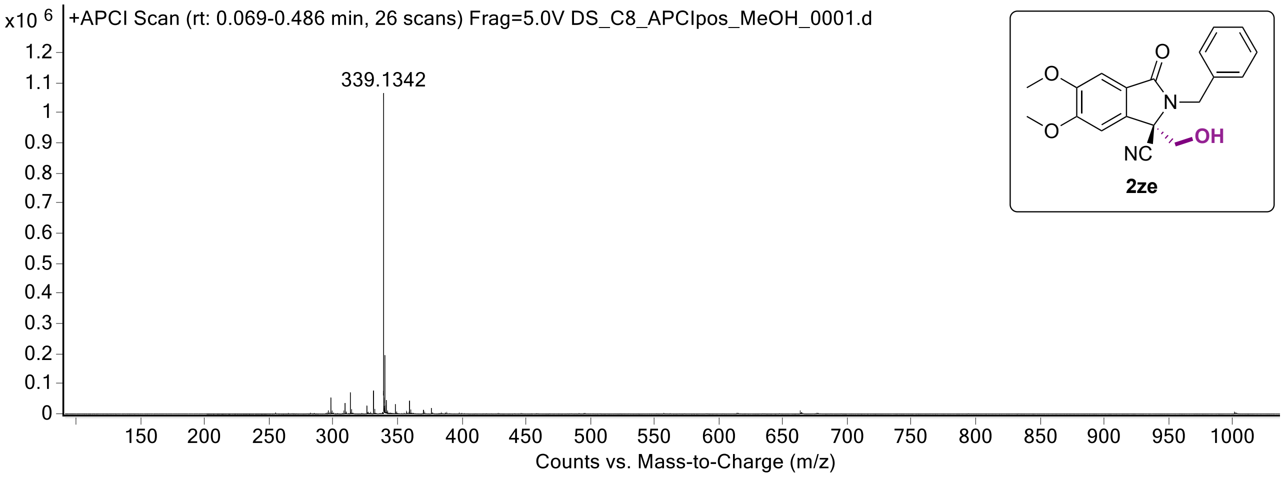

Compound **2ze** HRMS (APCI) *m/z*: $[M + H]^+$ calcd for $C_{19}H_{19}N_2O_4$ 339.1339, found 339.1342.

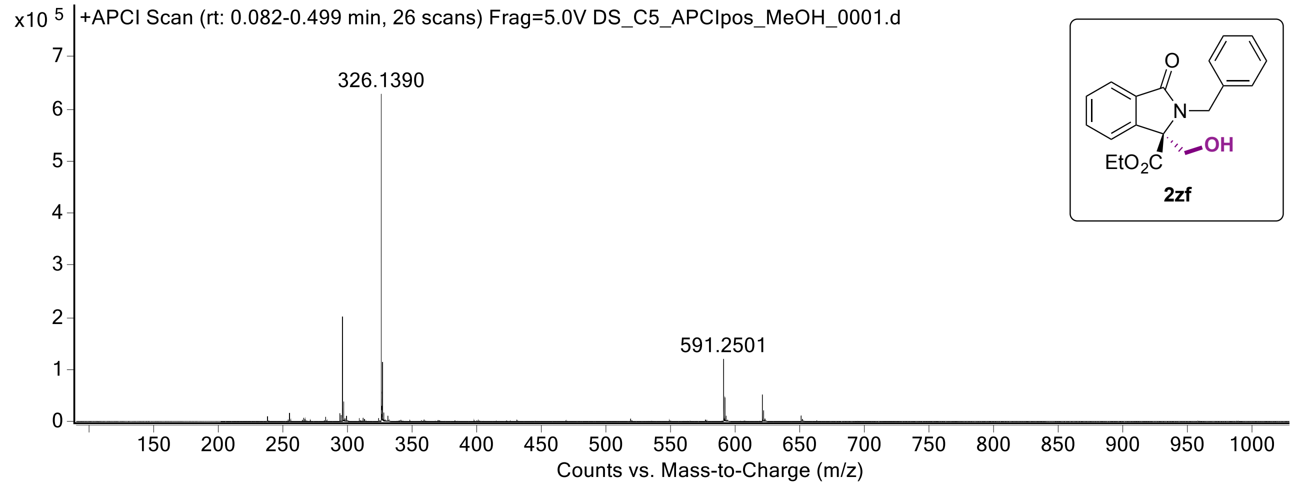

Compound **2zf** HRMS (APCI) *m/z*: $[M + H]^+$ calcd for $C_{19}H_{20}NO_4$ 326.1387, found 326.1390.

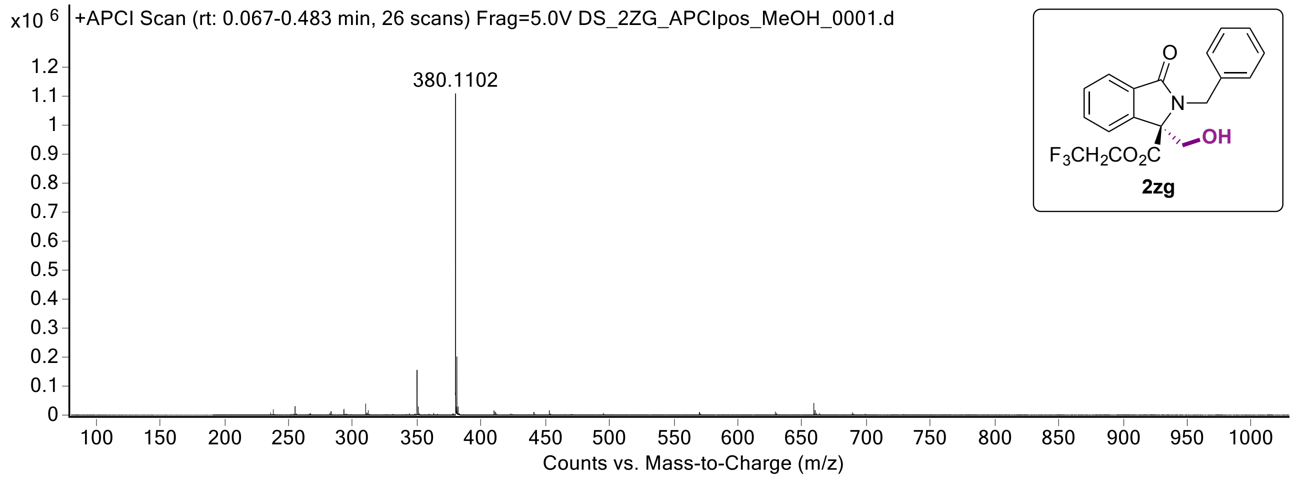

Compound **2zg** HRMS (APCI) *m/z*: $[M + H]^+$ calcd for $C_{19}H_{17}F_3NO_4$ 380.1104, found 380.1102.

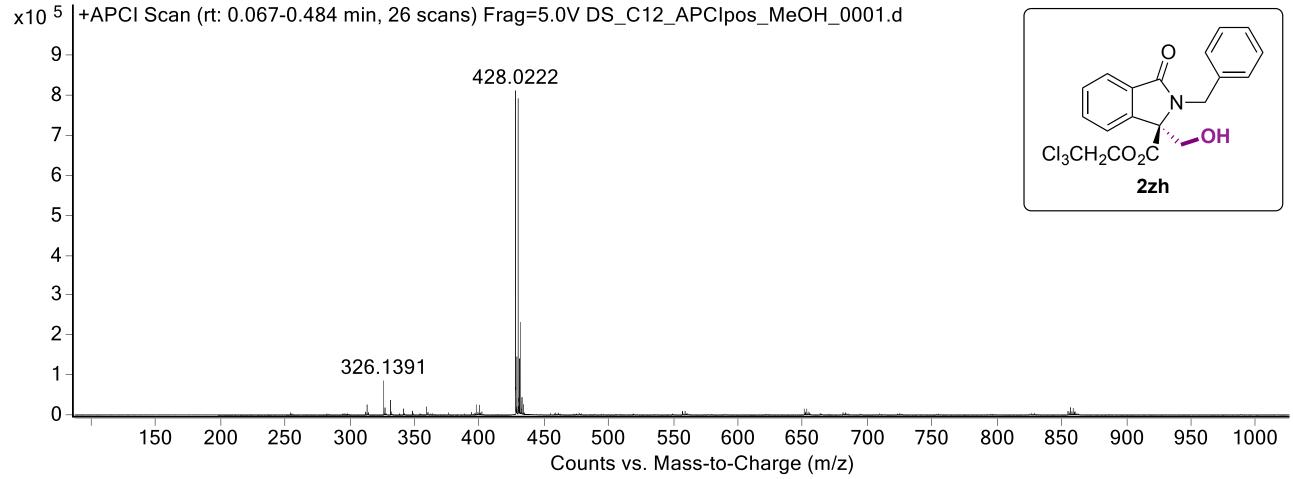

Compound **2zh** HRMS (APCI) *m/z*: $[M + H]^+$ calcd for $C_{19}H_{17}Cl_3NO_4$ 428.0218, found 428.0222.

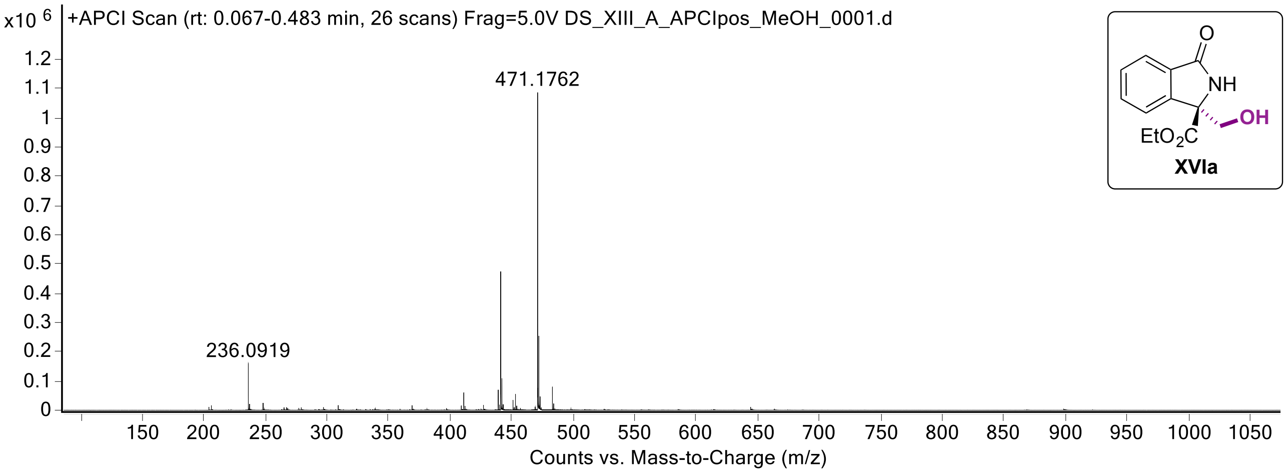

Compound **XVIa** HRMS (APCI) *m/z*: $[M + H]^+$ calcd for $C_{12}H_{14}NO_4$ 236.0917, found 236.0919.

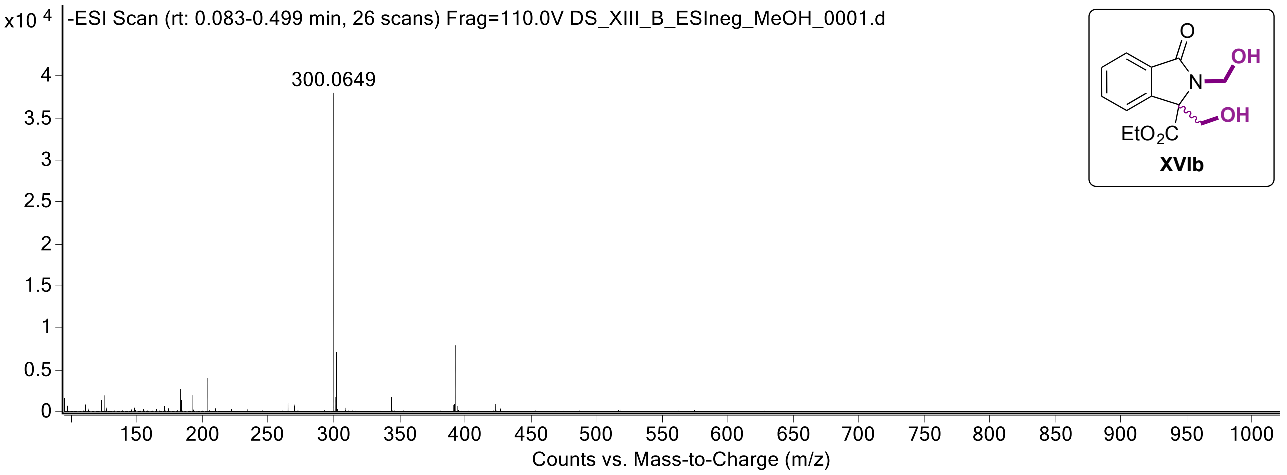

Compound **XVIb** HRMS (APCI) *m/z*: $[M + Cl]^-$ calcd for $C_{13}H_{15}ClNO_5$ 300.0644, found 300.0649.

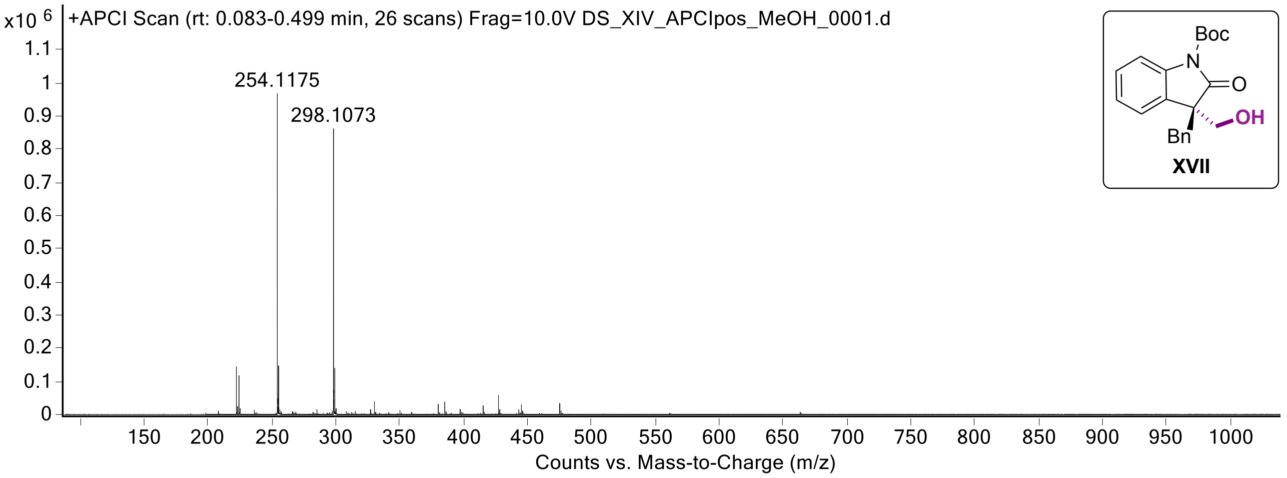

Compound **XVII** HRMS (APCI) *m/z*: $[M + H - Boc + H]^+$ calcd for $C_{16}H_{16}NO_2$ 254.1176, found 254.1175.

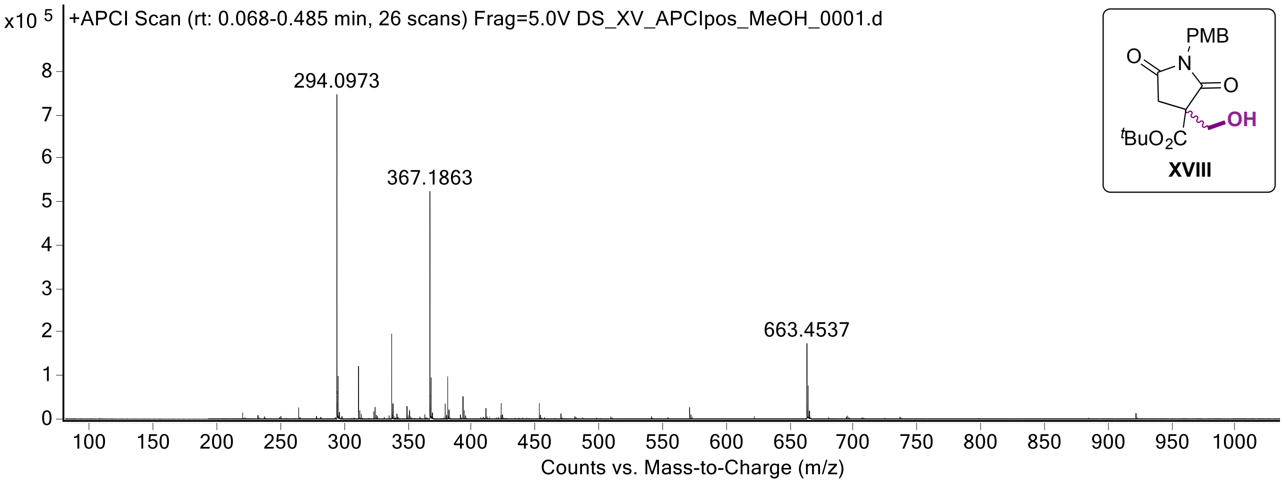

Compound **XVIII** HRMS (APCI) *m/z*: $[M + NH_4]^+$ calcd for $C_{18}H_{27}N_2O_6$ 367.1864, found 367.1863.

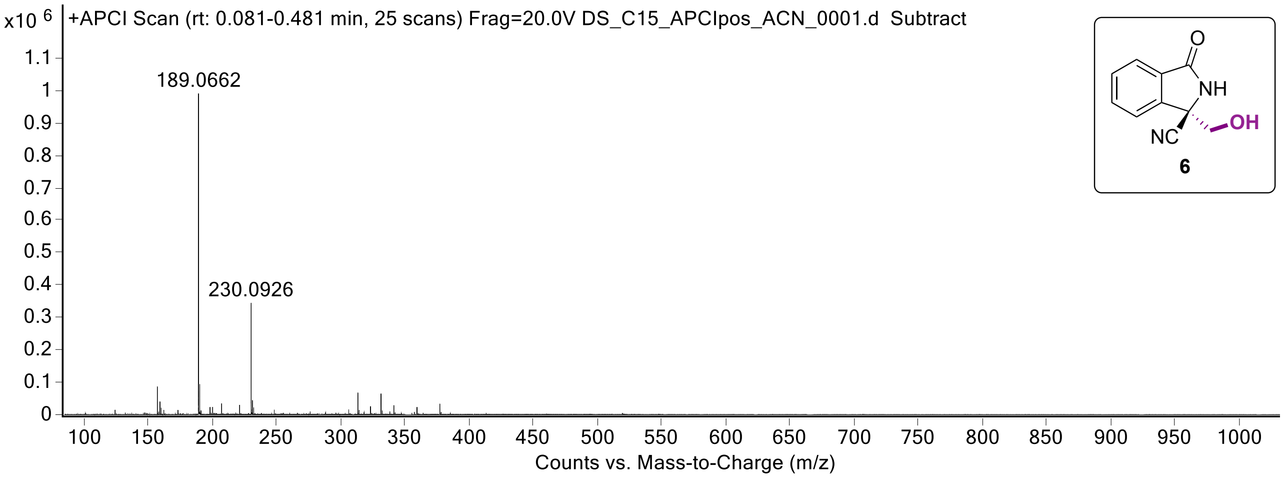

Compound **6** HRMS (APCI) *m/z*: $[M + H]^+$ calcd for $C_{10}H_9N_2O_2$ 189.0659, found 189.0662.

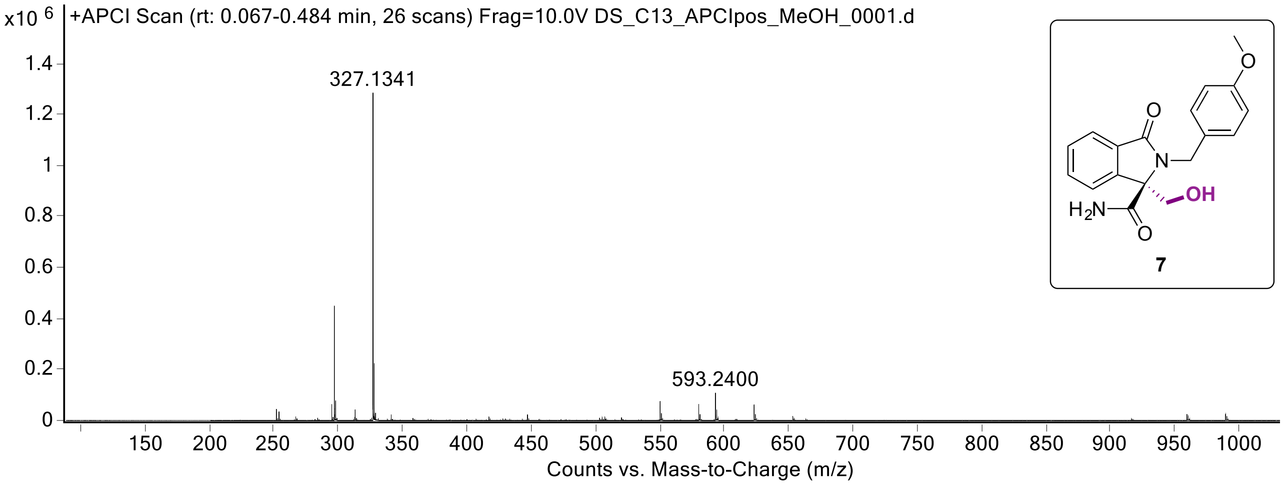

Compound **7** HRMS (APCI) *m/z*: $[M + H]^+$ calcd for $C_{18}H_{19}N_2O_4$ 327.1339, found 327.1341.

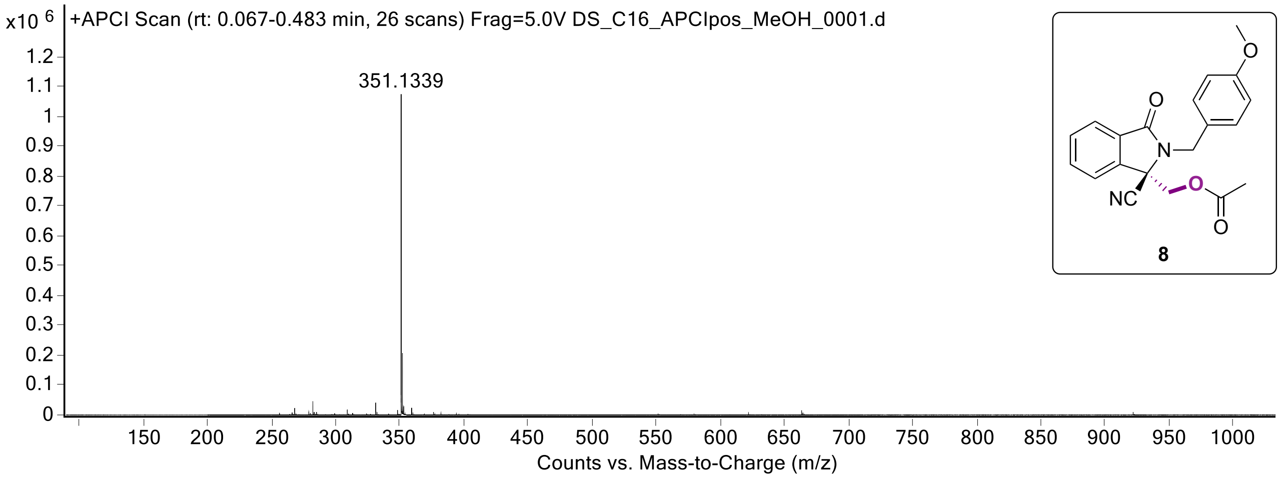

Compound **8** HRMS (APCI) *m/z*: $[M + H]^+$ calcd for $C_{20}H_{19}N_2O_4$ 351.1339, found 351.1339.

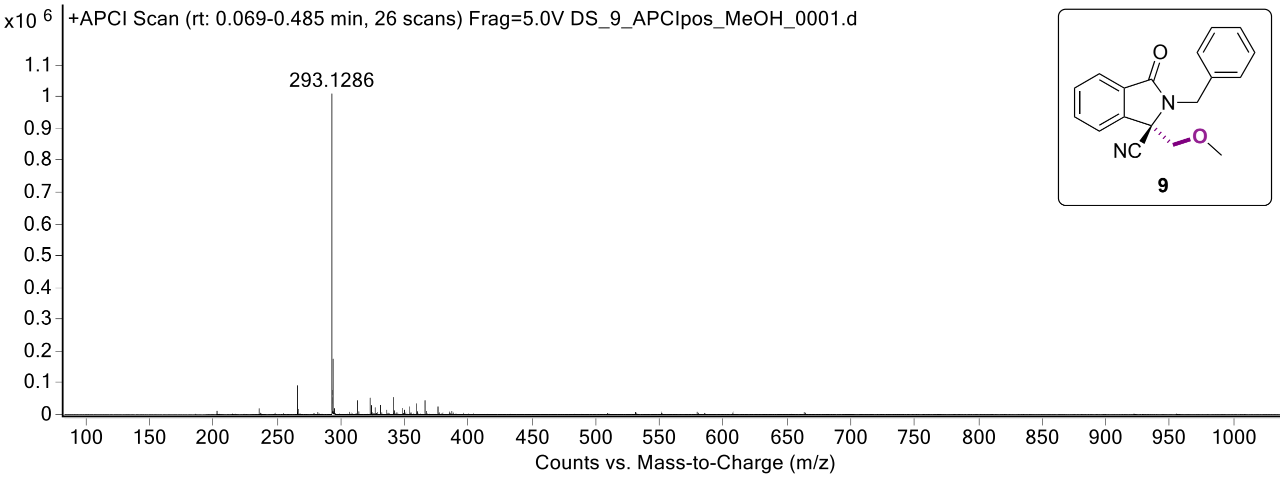

Compound **9** HRMS (APCI) *m/z*: $[M + H]^+$ calcd for $C_{18}H_{17}N_2O_2$ 293.1285, found 293.1286.

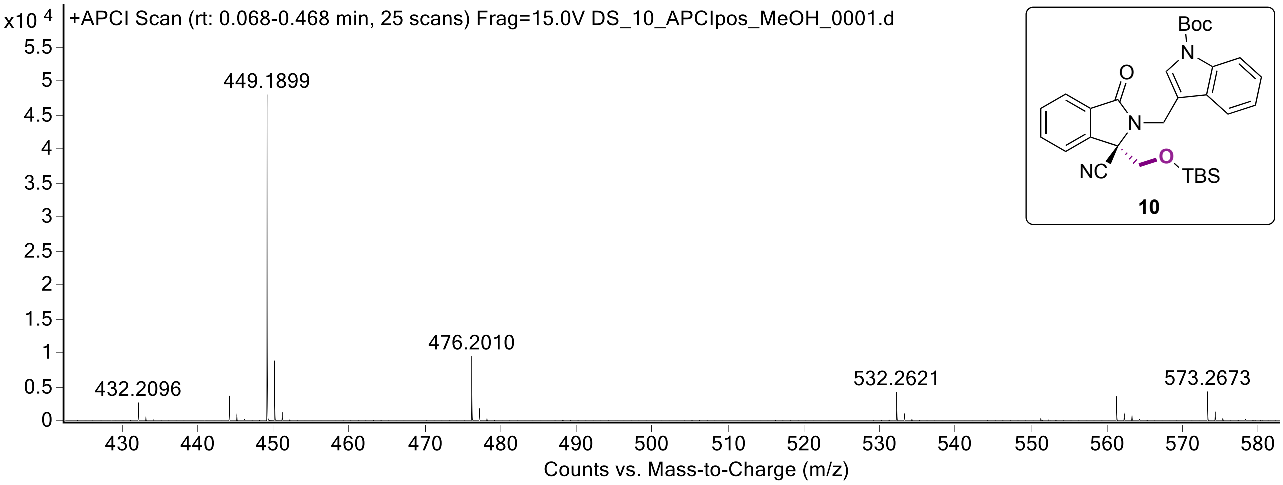

Compound **10** HRMS (APCI) *m/z*: $[M + H]^+$ calcd for $C_{30}H_{38}N_3O_4Si$ 532.2626, found 532.2621.

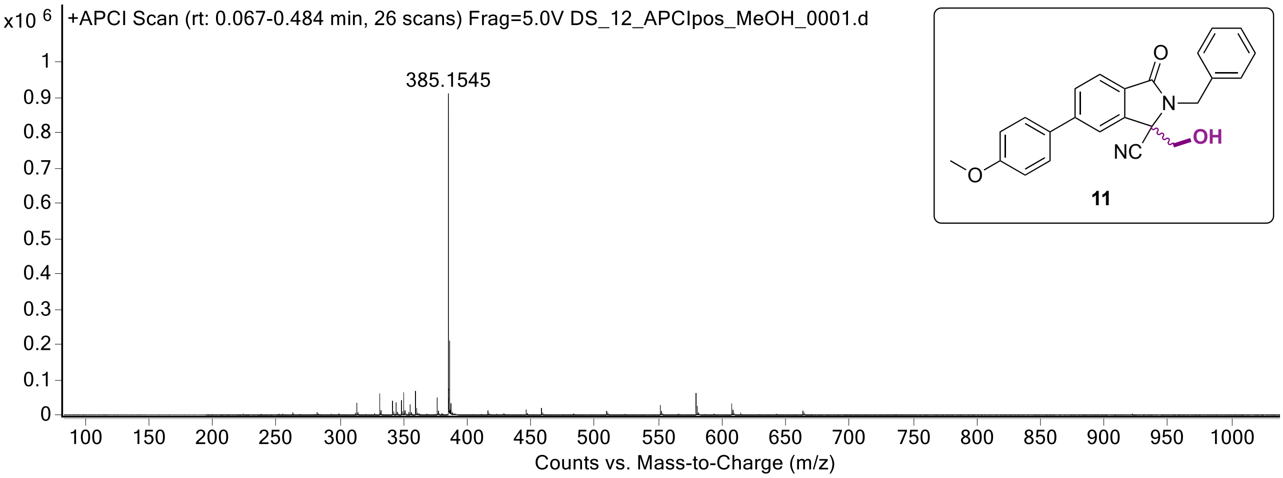

Compound **11** HRMS (APCI) *m/z*: $[M + H]^+$ calcd for $C_{24}H_{21}N_2O_3$ 385.1547, found 385.1545.

11. References

[1] Otevrel, J.; Bobal, P. Biphenyl-Based Bis(thiourea) Organocatalyst for Asymmetric and *syn*-Selective Henry Reaction. Synthesis **2017**, *49*, 593.

[2] Hofstetter, C.; Stone Wilkinson, P.; Pochapsky, T. C. NMR Structure Determination of Ion Pairs Derived from Quinine: A Model for Templating in Asymmetric Phase-Transfer Reductions by BH_4^- with Implications for Rational Design of Phase-Transfer Catalysts. *Org. Chem.* **1999**, *64*, 8794.

]3] Claraz, A.; Oudeyer, S., Levacher, V. Chiral Quaternary Ammonium Aryloxide/*N,O*-Bis(trimethyl-silyl)acetamide Combination as Efficient Organocatalytic System for the Direct Vinylogous Aldol Reaction of (*5H*)-Furan-2-one Derivatives. *Adv. Synth. Catal.* **2013**, *355*, 841.

[4] Otevrel, J.; Svestka, D.; Bobal, P. Bianthryl-Based Organocatalysts for the Asymmetric Henry Reaction of Fluoroketones. *Org. Biomol. Chem.* **2019**, *17*, 5244.

[5] Otevrel, J.; Bobal, P. Diamine-Tethered Bis(thiourea) Organocatalyst for Asymmetric Henry Reaction. *J. Org. Chem.* **2017**, *82*, 8342.

[6] Svestka, D.; Otevrel, J.; Bobal, P. Asymmetric Organocatalyzed Friedel–Crafts Reaction of Trihaloacetaldehydes and Phenols. *Adv. Synth. Catal.* **2022**, *364*, 2174.

[7] Zhang, Z.-H.; Dong, X.-Q.; Chen, D.; Wang, C.-J. Fine-Tunable Organocatalysts Bearing Multiple Hydrogen-Bonding Donors for Construction of Adjacent Quaternary and Tertiary Stereocenters *via* a Michael Reaction. *Chem. Eur. J.* **2008**, *14*, 8780.

[8] Berkessel, A.; Mukherjee, S.; Muller, T. N.; Cleemann, F.; Roland, K.; Brandenburg, M.; Neudorfl, J.-M.; Lex, J. Structural Optimization of Thiourea-Based Bifunctional Organocatalysts for the Highly Enantioselective Dynamic Kinetic Resolution of Azlactones. *Org. Biomol. Chem.* **2006**, *4*, 4319.

[9] McLeod, D. A.; Thøgersen, M. K.; Barløse, C. L.; Skipper, M. L.; Obregón, E. B.; Jørgensen, K. A. Enantioselective (8+3) Cycloadditions by Activation of Donor–Acceptor Cyclopropanes Employing Chiral Brønsted Base Catalysis. *Angew. Chem. Int. Ed.* **2022**, *61*, e202206096.

[10] Ni, D.; Wei, Y.; Ma, D. Thiourea-Catalyzed Asymmetric Michael Addition of Carbazolones to 2-Chloroacrylonitrile: Total Synthesis of 5,22-Dioxokopsane, Kopsinidine C, and Demethoxycarbonylkopsin. *Angew. Chem. Int. Ed.* **2018**, *32*, 10207.

[11] Jing, Z.; Bai, X.; Chen, W.; Zhang, G.; Zhu, B.; Jiang, Z. Organocatalytic Enantioselective Vinylogous Aldol Reaction of Allyl Aryl Ketones to Activated Acyclic Ketones. *Org. Lett.* **2016**, *18*, 260.

[12] Priede, M.; Kazak, M.; Kalnins, T.; Shubin, K.; Suna, E. Diastereoselective Hydroxymethylation of Cyclic *N-tert*-Butanesulfinylketimines Using Methoxymethanol as Formaldehyde Source, *J. Org. Chem.* **2014**, *79*, 3715

[13] Bontemps, S.; Sabo-Etienne, S. Trapping Formaldehyde in the Homogeneous Catalytic Reduction of Carbon Dioxide. *Angew. Chem. Int. Ed.* **2013**, *52*, 10253.

[14] Almasi, D.; Alonso, D. A.; Gomez-Bengoa, E.; Najera, C. Chiral 2-Aminobenzimidazoles as Recoverable Organocatalysts for the Addition of 1,3-Dicarbonyl Compounds to Nitroalkenes. *J. Org. Chem.* **2009**, *74*, 6163.

[15] Thai, K; Gravel, M. Design, Synthesis, and Application of Chiral Electron-Poor Guanidines as Hydrogen-Bonding Catalysts for the Michael Reaction. *Tetrahedron: Asymmetry*. **2010**, *21*, 751.

[16] Schmitt, E.; Schiffers, I.; Bolm, C. Highly Enantioselective Desymmetrizations of *meso*-Anhydrides. *Tetrahedron.* **2010**, *66*, 6349.

[17] Witiak, D. T; Lee, H. J.; Hart, R. W.; Gibson, R. E. Study of *trans*-Cyclopropylbis(diketopiperazine) and Chelating Agents Related to ICRF 159. Cytotoxicity, Mutagenicity, and Effects on Scheduled and Unscheduled DNA Synthesis. *J. Med. Chem.* **1977**, *20*, 630.

[18] Furuta, K.; Iwanaga, K.; Yamamoto, H. Condensation of (−)-Dimenthyl Succinate Dianion with 1,ω-Dihalides: (+)-(1*S*,2*S*)-Cyclopropane-1,2-dicarboxylic Acid. *Org. Synth.* **1989**, *67*, 76.

[19] Pokorski, J. K.; Myers, M. C.; Appella, D. H. Cyclopropane PNA: Observable Triplex Melting in a PNA Constrained with a 3-Membered Ring. *Tetrahedron Lett.* **2005**, *46*, 915.

[20] (a) Daly, A. M.; Gilheany, D. G. The Synthesis and Use in Asymmetric Epoxidation of Metal Salen Complexes Derived from Enantiopure *trans*-Cyclopentane- and Cyclobutane-1,2-diamine. *Tetrahedron: Asymmetry*. **2003**, *14*, 127. (b) Toftlund, H.; Pedersen, E. The Preparation and Optical Activity of the Isomers of the 1,2-Cyclopentanediamine Cobalt(III) and Chromium(III) Complexes. *Acta Chem. Scand.* **1972**, *26*, 4019.

[21] Yang, C.; Zhang, E.-G.; Li, X.; Cheng, J.-P. Asymmetric Conjugate Addition of Benzofuran-2-ones to Alkyl 2-Phthalimidoacrylates: Modeling Structure–Stereoselectivity Relationships with Steric and Electronic Parameters. *Angew. Chem. Int. Ed.* **2016**, *55*, 6506.
[22] Li, X.; Deng, H.; Zhang, B.; Li, J.; Zhang, L.; Luo, S.; Cheng, J.-P. Physical Organic Study of Structure–Activity–Enantioselectivity Relationships in Asymmetric Bifunctional Thiourea Catalysis: Hints for the Design of New Organocatalysts. *Chem. Eur. J.* **2010**, *16*, 450.
[23] Choudhury, A. R.; Mukherjee, S. A Catalytic Michael/Horner–Wadsworth–Emmons Cascade Reaction for Enantioselective Synthesis of Thiochromenes. *Adv. Synth. Catal.* **2013**, *355*, 1989.
[24] Cherbuliez, E.; Sulzer, G. Dérivés Formaldéhydiques de la Succinimide. *Helv. Chim. Acta.* **1925**, *8*, 568.
[25] Ermakov, A. S.; Bulatov, P. V.; Vinogradov, D. B.; Tartakovskii, V. A. Synthesis of Nitroesters Based on Methylol Derivatives of Parabanic and Isocyanuric Acids. *Russian J. Org. Chem.* **2005**, *41*, 255.
[26] Böhme, H.; Driesen, G.; Schünemann, D. Über Umsetzungen von *N*-Hydroxymethyl-und *N*-Chlormethyl-lactamen. *Arch. Pharm.* **1961**, *294*, 344.
[27] Taggart, M. S.; Richter, G. H. Synthesis of Pyrrole Alcohols and Their Derivatives *J. Am. Chem. Soc.* **1934**, *56*, 1385.
[28] Hüttel, R.; Jochum, P. Die Mannichsche Reaktion der Pyrazole. *Chem. Ber.* **1952**, *85*, 820.
[29] Majumdar, S.; Spaeth, M. M.; Sivendran, S.; Juntunen, J.; Thomas, J. D.; Sloan, K. B. α-(1*H*-Imidazol-1-yl)alkyl (IMIDA) Carboxylic Acid Esters as Prodrugs of Carboxylic Acid Containing Drugs. *Tetrahedron Lett.* **2007**, *48*, 4609.
[30] Vereshchagin, L. I.; Maksikova, A. V.; Tikhonova, L. G.; Buzilova, S. R.; Sakovich, G. V. Synthesis of Polynuclear Uncondensed Tri- and Tetrazoles. *Chem. Heterocycl. Compd.* **1981**, *17*, 510.
[31] Kalisiak, J.; Sharpless, K. B.; Fokin, V. V. Efficient Synthesis of 2-Substituted-1,2,3-Triazoles. *Org. Lett.* **2008**, *10*, 3171.
[32] Maier, L.; Kunz, W.; Rist, G. Organische Phosphorverbindungen 80 Herstellung von Triazolylmethyl-Phosphonaten und von Triazolylmethylphosphoniumsalzen und Deren Verwendung in der Wittig–Horner Reaktion. *Phosphorus Sulfur Silicon Relat. Elem.* **1987**, *33*, 41.
[33] Tselinskii, I. V.; Melnikov, A. A.; Varyagina, L. G.; Zhigadlova, I. G. Synthesis of *N*-Hydroxymethyl Derivatives of 5-Substituted Tetrazoles. *Chem. Heterocycl. Compd.* **1983**, *19*, 341.
[34] Sinderhauf, K.; Schwack, W. Photolysis Experiments on Phosmet, an Organophosphorus Insecticide. *J. Agric. Food Chem.* **2003**, 51, 5990.
[35] Bohme, H.; Schwartz, H. Uber *N*-[Imido-methyl]-thiocyanate und -isothiocyanate. *Arch. Pharm.* **1974**, *307*, 775.
[36] Choi, S.-Y.; Lee, S.-G.; Yoon, Y.-J.; Kim, K.-W. Reaction of *N*-Hydroxymethylsaccharin with Aliphatic Carboxylic Acid Derivatives: Synthesis of *N*-Acylsaccharins and *N*-(Saccharinylmethyl) Aliphatic Carboxylates. *J. Het. Chem.* **1989**, *26*, 1073.
[37] Alkorta, I.; Elguero, J.; Jagerovic, N.; Fruchier, A.; Yap. G. P. A. Study of the Structure of 1-Hydroxymethylindazole and 1-Hydroxymethylbenzotriazole by X-Ray Crystallography, Multinuclear NMR in Solution and DFT Calculations. *J. Heterocyclic Chem.* **2004**, *41*, 285.
[38] Abdireimov, K. B.; Mukhamedov, N. S.; Aiymbetov M. Z.; Shakhidoyatov, K. M. Benzazoles 5. Synthesis and Arylsulfonylation of 1-Hydroxymethylbenzimidazole. *Chem. Heterocycl. Compd.* **2012**, *48*, 458.
[39] Zhang, J.-P.; Li, Q.; Zhang, C.; Li, P.; Chen, L.-J.; Wang, Y.-H.; Ruan, X.-H.; Xiao, W.; Xue, W. Synthesis, Antibacterial, and Antiviral Activities of Novel Penta-1,4-dien-3-one Derivatives Containing a Benzotriazin-4(3*H*)-one Moiety. *Chem. Pap.* **2018**, *72*, 2193.
[40] *Patent* NL6400564, **1964**; *Chem.Abstr.* **1965**, *62*, 58972.
[41] Burton, D. J.; Wiemers, D. M. A Practical Synthesis of Fluoromethyltriphenylphosphonium Salts. *J. Fluorine Chem.* **1985**, *27*, 85.
[42] Liu, S.; Prevost, S. Palladium-Catalyzed *ortho*-C–H Alkoxycarbonylation of Aromatic Aldehydes *via* a Transient Directing Group Strategy. *Org.Lett.* **2023**, *25*, 1380.
[43] Bisai, V.; Unhale, R. A.; Suneja, A.; Dhanesekaran, S.; Singh, V. K. An Efficient Entry to *syn*- and *anti*-Selective Isoindolinones *via* an Organocatalytic Direct Mannich/Lactamization Sequence. *Org. Lett.* **2015**, *17*, 2102.
[44] Zhang, Y.-H.; Shi, B.-F.; Yu, J.-Q. Palladium(II)-Catalyzed *ortho*-Alkylation of Benzoic Acids with Alkyl Halides. *Angew. Chem. Int. Ed.* **2009**, *48*, 6097.
[45] *Patent* US20140256734, **2014**; *Chem. Abstr.* **2014**, *161*, 459972.

[46] Ghosh, U.; Bhattacharyya, R.; Keche, A. Mild and Efficient Syntheses of Diverse Isoindolinones from *ortho*-Phthaldehydic Acid Methylthiomethyl Ester. *Tetrahedron*. **2010**, *66*, 2148.

[47] Dhanesekaran, S.; Suneja, A.; Bisai, V.; Singh, V. K. Approach to Isoindolinones, Isoquinolinones, and THIQs *via* Lewis Acid-Catalyzed Domino Strecker-Lactamization/Alkylations. *Org. Lett.* **2016**, *18*, 634.

[48] Chen, T.; Cai, C. $Sc(OTf)_3$-Catalyzed Three-Component Cascade Reaction: One-Pot Synthesis of Substituted 3-Oxoisoindoline-1-carbonitrile Derivatives. *Catal. Commun.* **2016**, *74*, 119.

[49] Eitzinger, A.; Otevrel, J.; Haider, V.; Macchia, A.; Massa, A.; Faust,K.; Spingler, B. Berkessel, A.; Waser, M. Enantioselective Bifunctional Ammonium Salt-Catalyzed Syntheses of 3-CF_3S-, 3-RS-, and 3-F-Substituted Isoindolinones. *Adv. Synth. Catal.* **2021**, *363*, 1955.

[50] (a) Othman, M.; Pigeon, P.; Decroix, B. New Fused Lactones from lndolizinediones *via N*-Acyliminium Ions. *Tetrahedron*. **1998,** *54*, 8737. (b) Scorzelli, F.; Di Mola, A.; De Piano, F.; Tedesco, C.; Palombi, L.; Filosa, R.; Waser, M.; Massa, A. systematic study on the use of different organocatalytic activation modes for asymmetric conjugated addition reactions of isoindolinones. *Tetrahedron*. **2017**, *73*, 819.

[51] Scorzelli, F.; Di Mola, A.; Filosa, R.; Massa, A. Organocatalytic Asymmetric Hydroxymethylation of Isoindolinones with Paraformaldehyde. *Monatsh. Chem.* **2018**, *149*, 723.

[52] Liu, X.-L.; Liao, Y.-H.; Wu, Z.-J.; Cun, L.-F.; Zhang, X.-M.; Yuan, W.-C. Organocatalytic Enantioselective Hydroxymethylation of Oxindoles with Paraformaldehyde as C1 Unit. *J. Org. Chem.* **2010**, *75*, 4872.

[53] *Aldrich Technical Bulletin AL180* Diazald® and Diazomethane Generators, www.sigmaaldrich.com/content/dam/sigma-aldrich/docs/Aldrich/Bulletin/al_techbull_al180.pdf (*Accessed:* Jan 9, 2024).

[54] Hajra, S.; Maity, S.; Maity, R. Efficient Synthesis of 3,3′-Mixed Bisindoles *via* Lewis Acid Catalyzed Reaction of Spiro-epoxyoxindoles and Indoles. *Org. Lett.* **2015**, *17*, 3430.

[55] Ričko, S.; Bitsch, R. S.; Kaasik M.; Otevrel, J.; Madsen, M. H. Keimer, A.; Jørgensen, K. A. Enantioconvergent 6π Electrocyclization Enabled by Photoredox Racemization. *J. Am. Chem. Soc.* **2023**, *145*, 20913.

[56] Sheldrick, G. SHELXT – Integrated Space-Group and Crystal-Structure Determination. *Acta Crystallogr. A.* **2015**, *71*, 3.

[57] Sheldrick, G. Crystal Structure Refinement with SHELXL. *Acta Crystallogr. C.* **2015**, *71*, 3.

[58] Parsons, S.; Flack, H. D.; Wagner, T. Use of Intensity Quotients and Differences in Absolute Structure Refinement. *Acta Crystallogr. B.* **2013**, *69*, 249.

[59] These data can be obtained free of charge from The Cambridge Crystallographic Data Centre at www.ccdc.cam.ac.uk.
